# Supplementary material for: Interferon-induced PARP14-mediated ADP-ribosylation in p62 bodies requires the ubiquitin-proteasome system
Source: EMBO J. 2025 Apr 7;44(10):2741–73. doi: 10.1038/s44318-025-00421-4 (PMC12084362; doi:10.1038/s44318-025-00421-4)
Supplement: Supplementary file 4 — Movie EV1 [file 44318_2025_421_MOESM4_ESM.zip › Movie EV1/Expanded View Movie 1 Legend.docx]

**Expanded View Movie 1**

3D representation of ADPr (green) condensate with p62 (blue) and PARP14 (red) after 24-h IFNγ (500 IU/ml) treatment by surface detection module (Imaris), Scale bar = 1µm.
