# Supplementary figures and images for: Interferon-induced PARP14-mediated ADP-ribosylation in p62 bodies requires the ubiquitin-proteasome system (part 1 of 4)
Source: EMBO J. 2025 Apr 7;44(10):2741–73. doi: 10.1038/s44318-025-00421-4 (PMC12084362; doi:10.1038/s44318-025-00421-4)

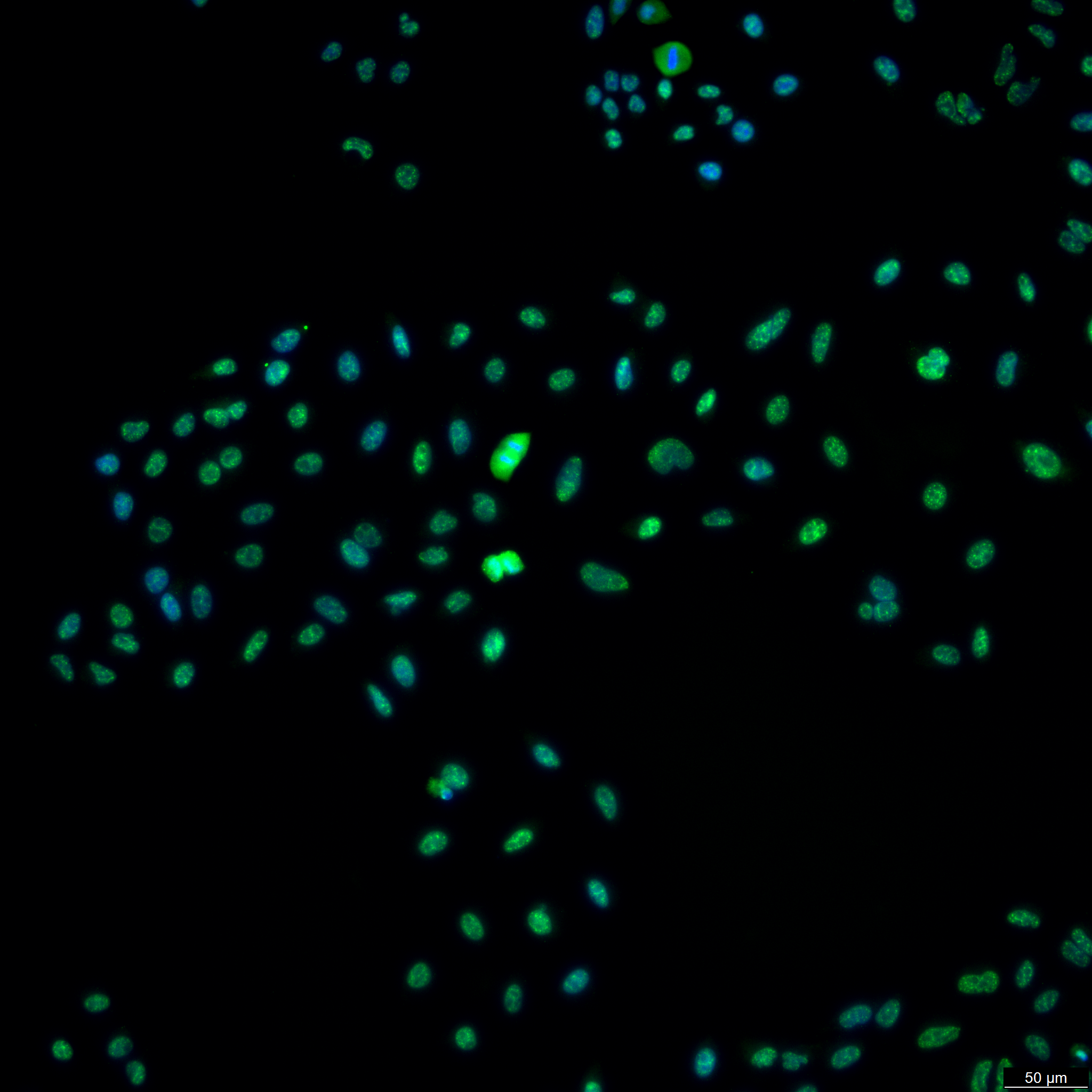

Supplement: Supplementary file 5 — Source data Fig. 1 [file 44318_2025_421_MOESM5_ESM.zip › Figure 1/Figure 1A/IFN 0 h.tif]

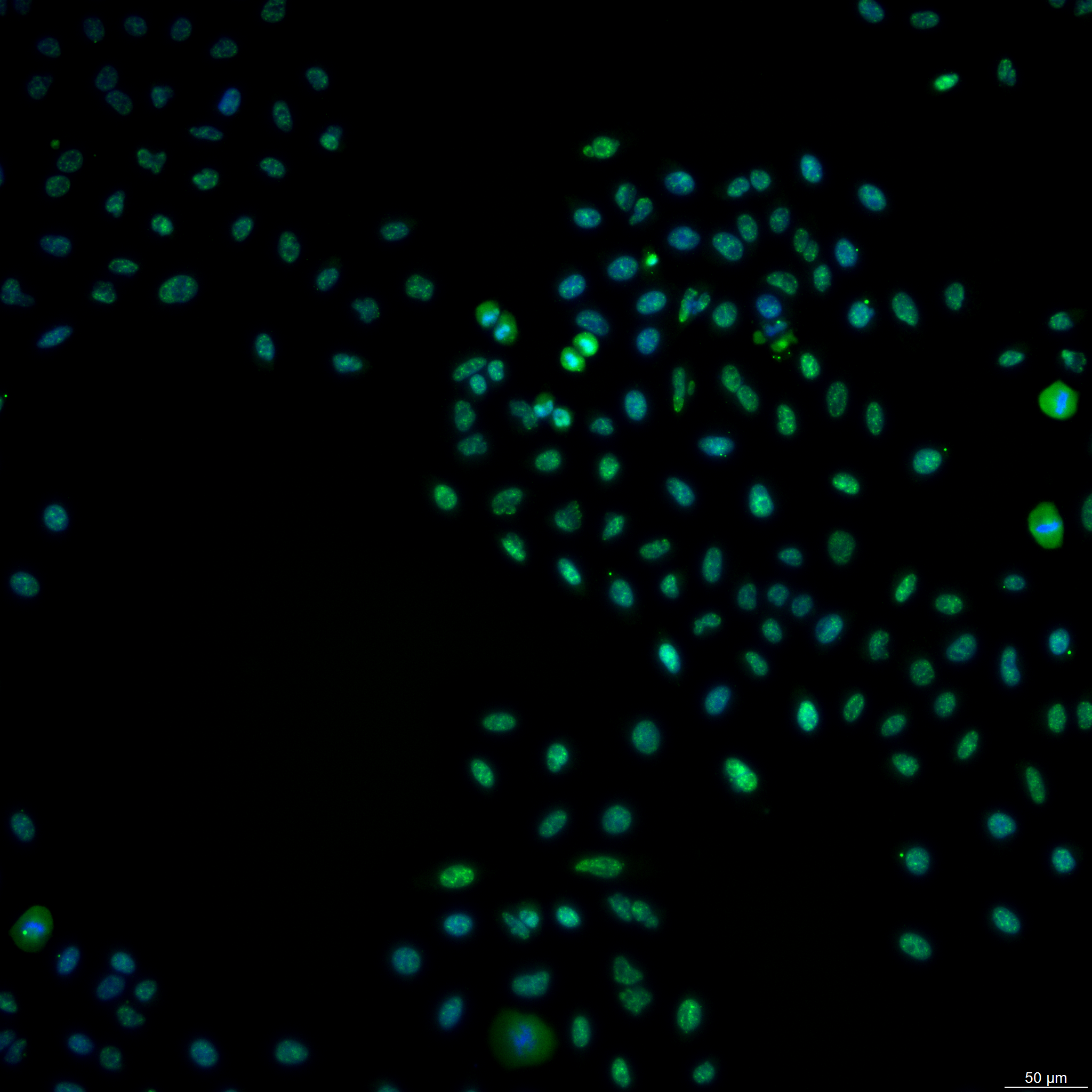

Supplement: Supplementary file 5 — Source data Fig. 1 [file 44318_2025_421_MOESM5_ESM.zip › Figure 1/Figure 1A/IFN 1 hr 1.tif]

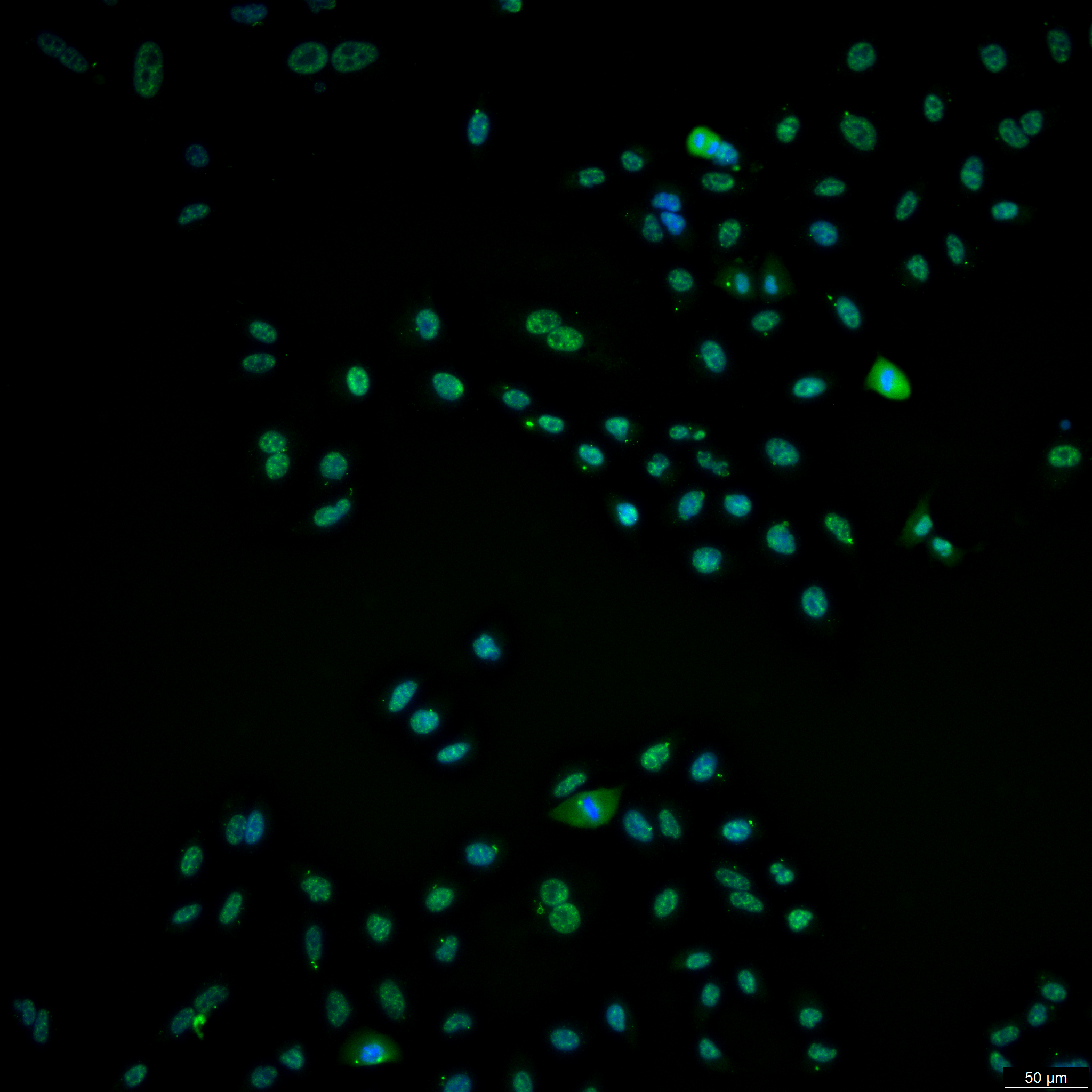

Supplement: Supplementary file 5 — Source data Fig. 1 [file 44318_2025_421_MOESM5_ESM.zip › Figure 1/Figure 1A/IFN 10 HR 1.tif]

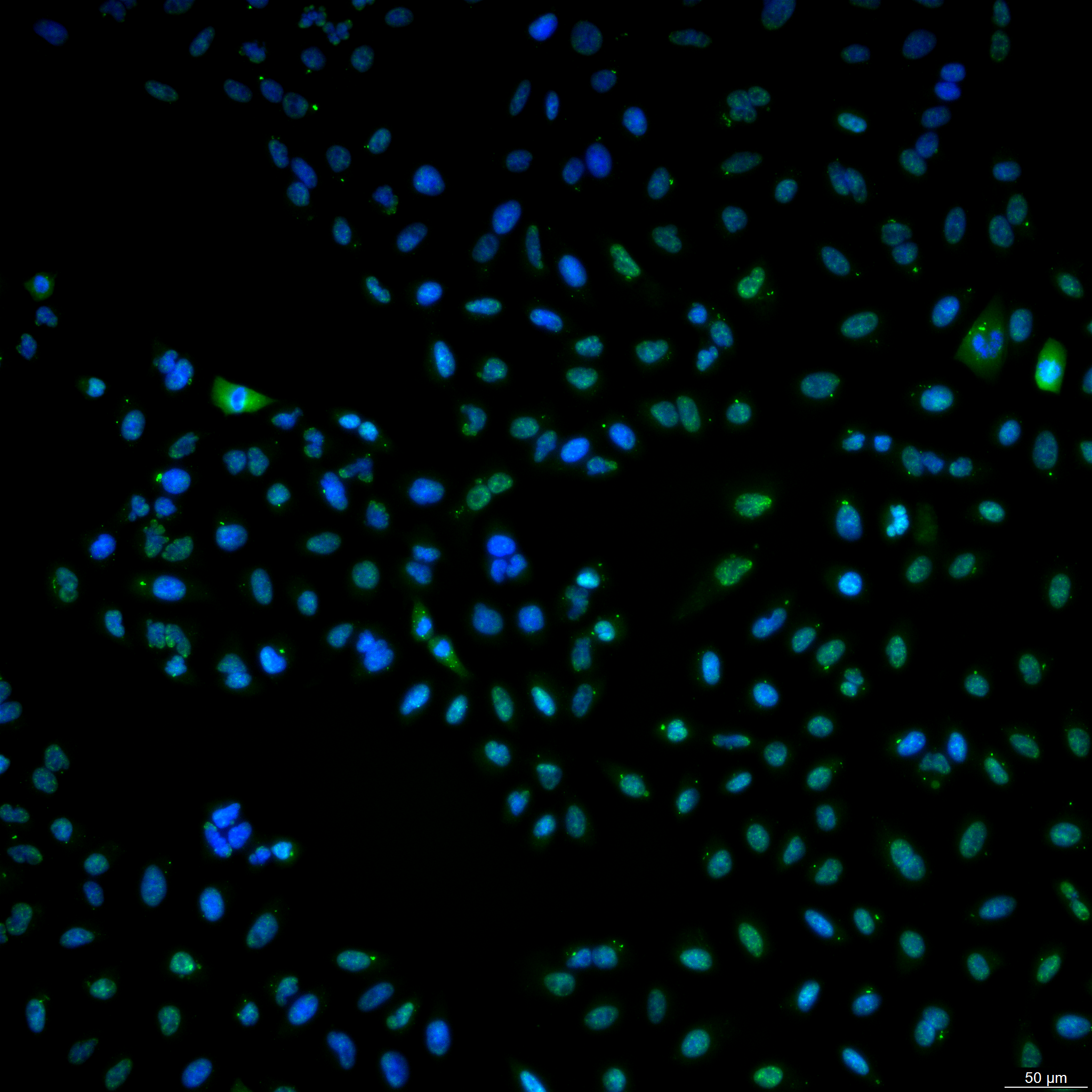

Supplement: Supplementary file 5 — Source data Fig. 1 [file 44318_2025_421_MOESM5_ESM.zip › Figure 1/Figure 1A/IFN 16 HR 1.tif]

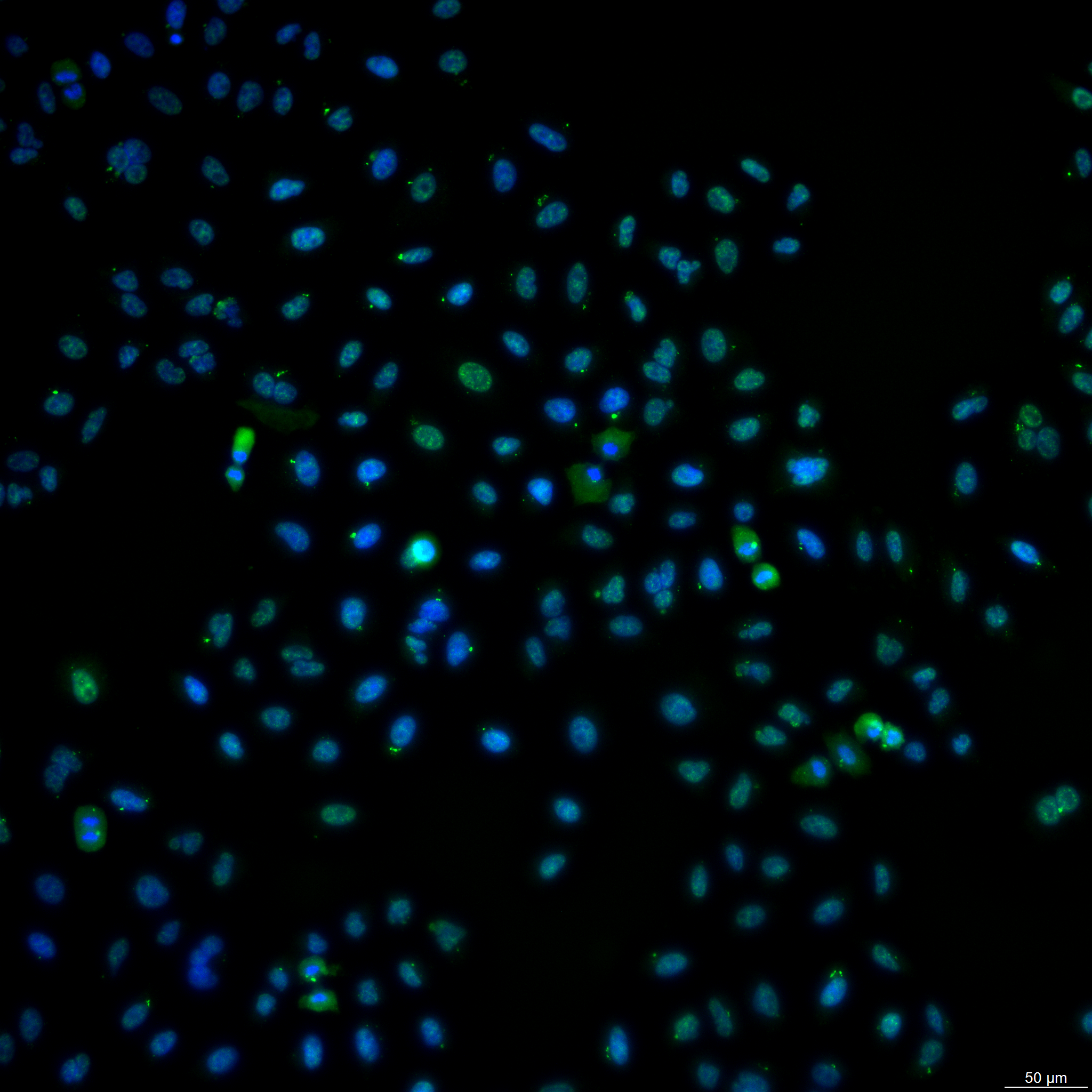

Supplement: Supplementary file 5 — Source data Fig. 1 [file 44318_2025_421_MOESM5_ESM.zip › Figure 1/Figure 1A/IFN 24 HR 1.tif]

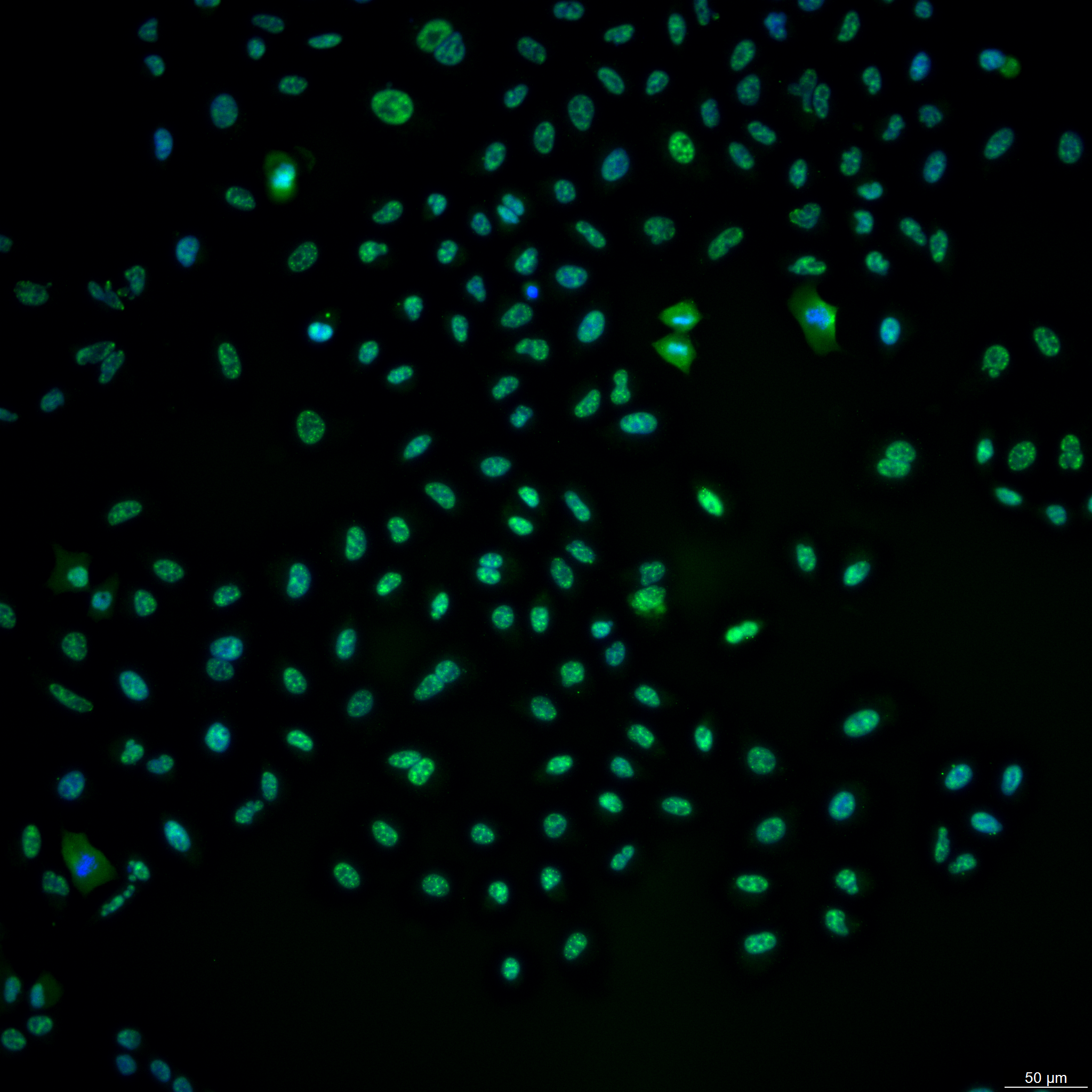

Supplement: Supplementary file 5 — Source data Fig. 1 [file 44318_2025_421_MOESM5_ESM.zip › Figure 1/Figure 1A/IFN 3 HR 1.tif]

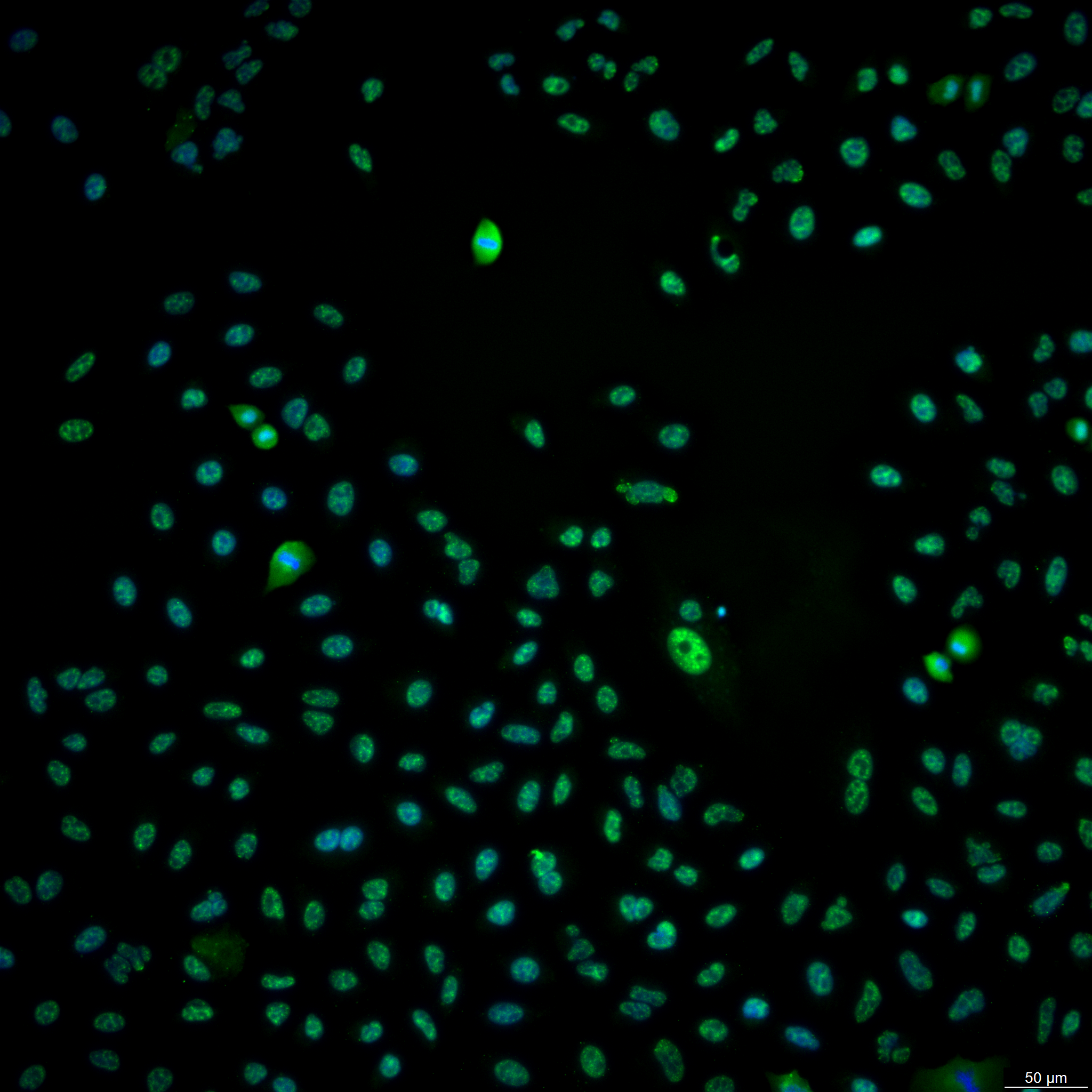

Supplement: Supplementary file 5 — Source data Fig. 1 [file 44318_2025_421_MOESM5_ESM.zip › Figure 1/Figure 1A/IFN 6HR 1.tif]

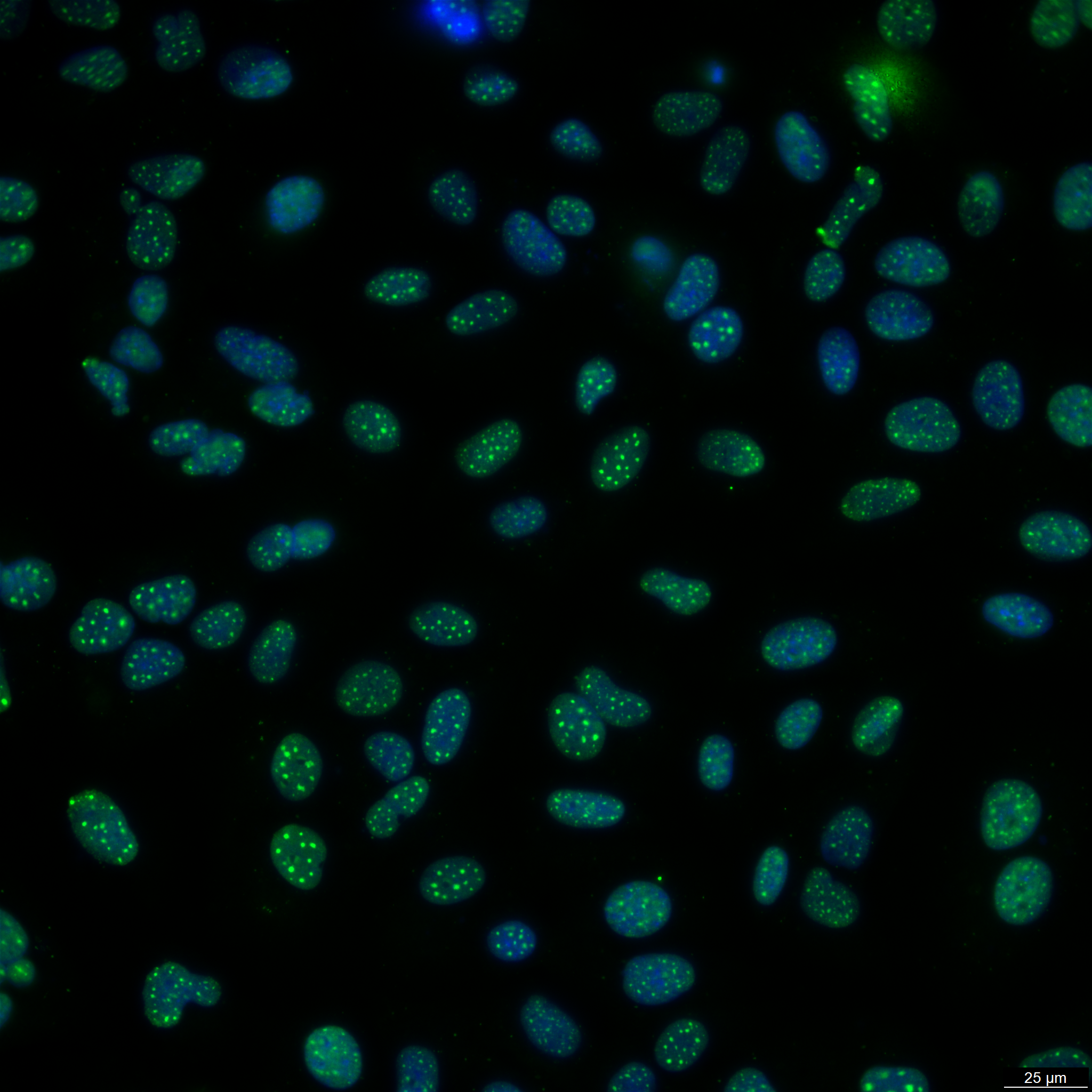

Supplement: Supplementary file 5 — Source data Fig. 1 [file 44318_2025_421_MOESM5_ESM.zip › Figure 1/Figure 1B/Act D .tif]

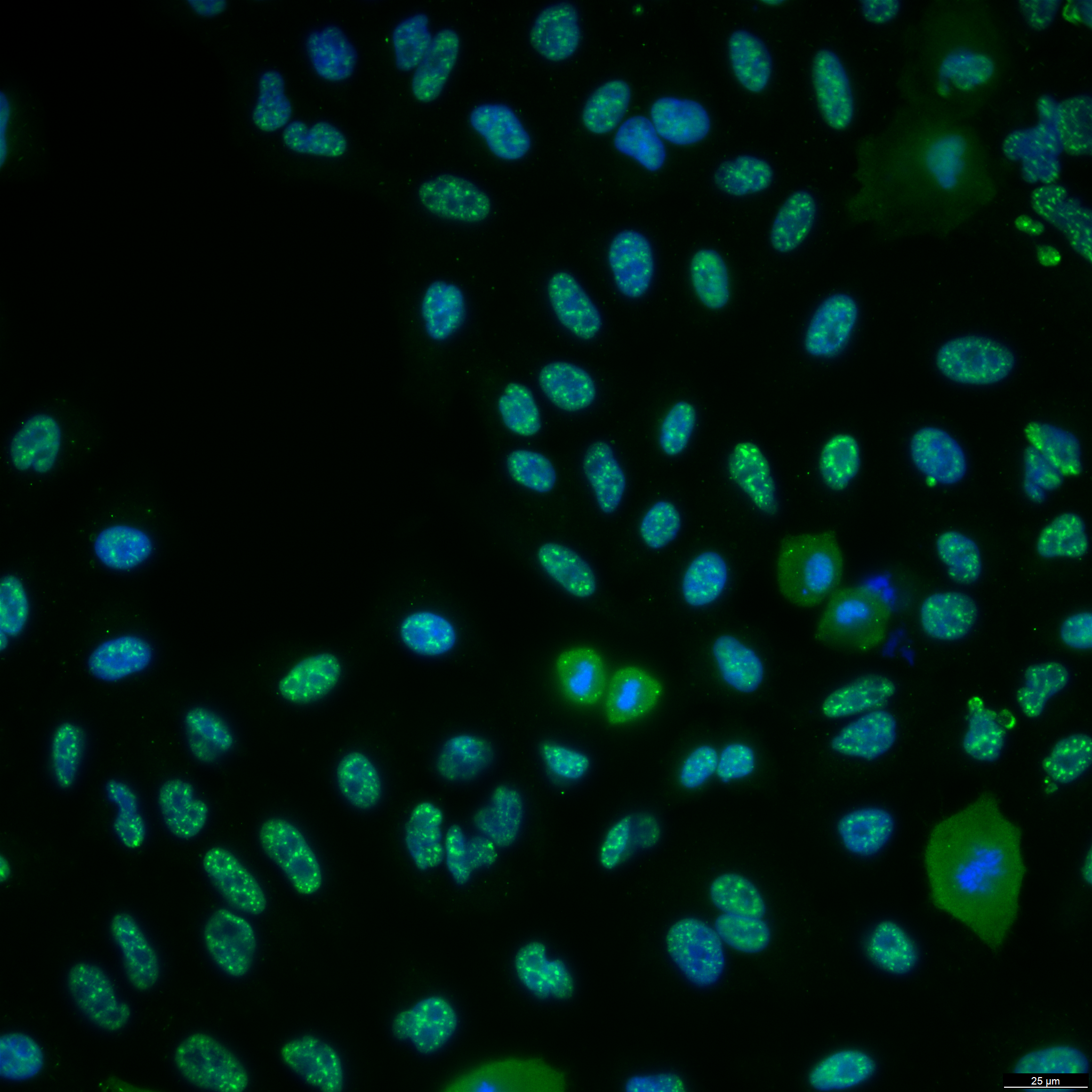

Supplement: Supplementary file 5 — Source data Fig. 1 [file 44318_2025_421_MOESM5_ESM.zip › Figure 1/Figure 1B/Control .tif]

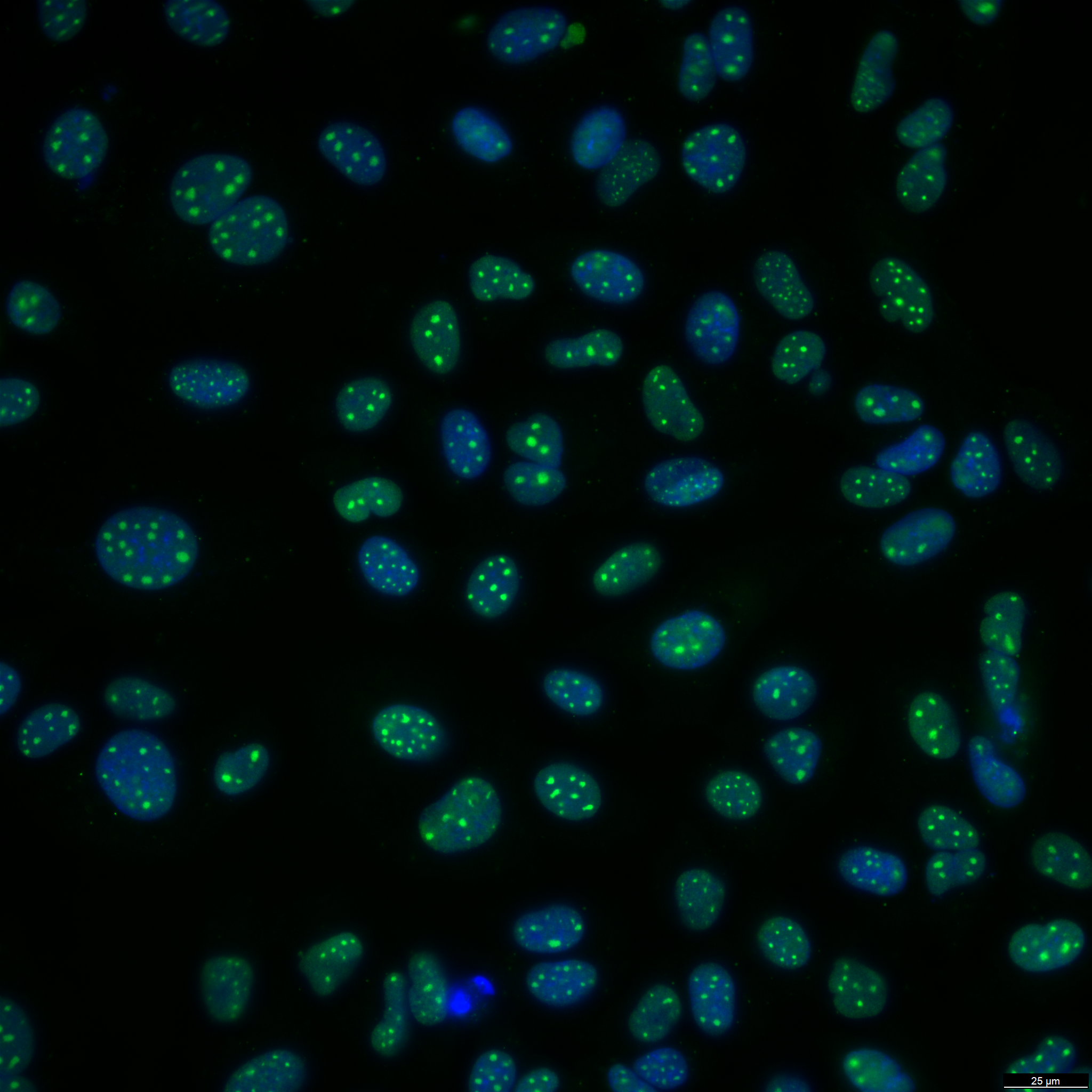

Supplement: Supplementary file 5 — Source data Fig. 1 [file 44318_2025_421_MOESM5_ESM.zip › Figure 1/Figure 1B/IFN+ Act D.tif]

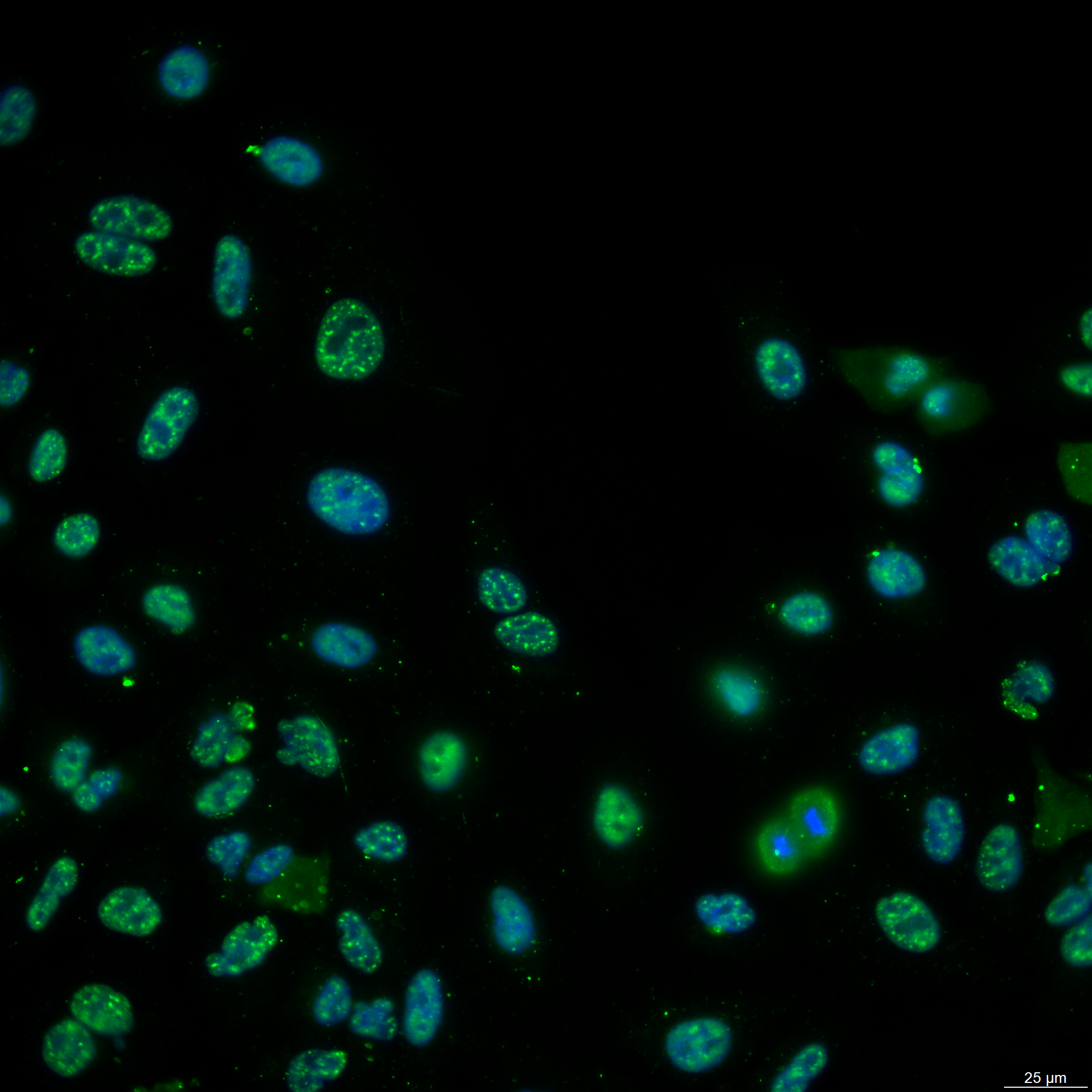

Supplement: Supplementary file 5 — Source data Fig. 1 [file 44318_2025_421_MOESM5_ESM.zip › Figure 1/Figure 1B/IFNγ.tif]

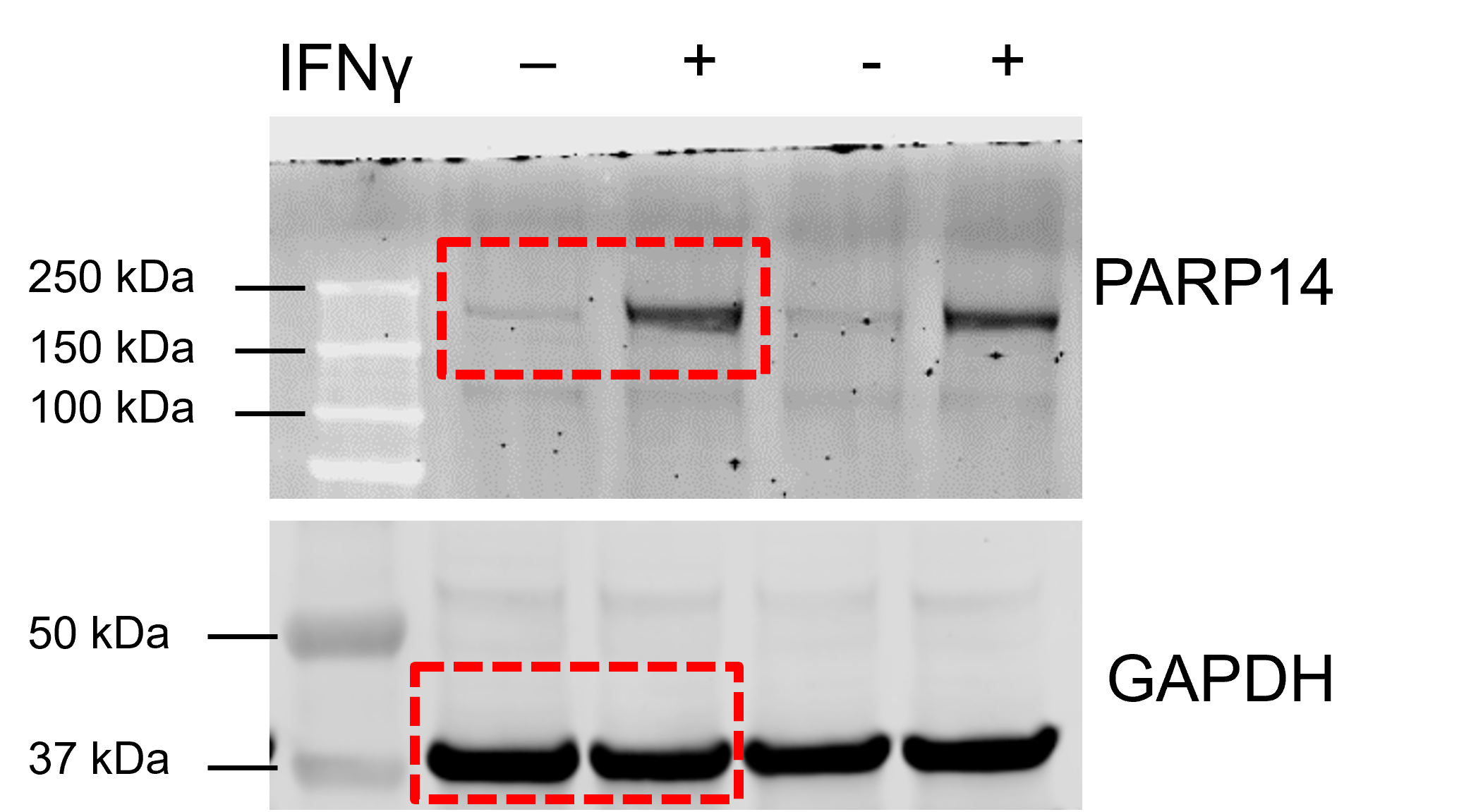

Supplement: Supplementary file 5 — Source data Fig. 1 [file 44318_2025_421_MOESM5_ESM.zip › Figure 1/Figure 1C.tif]

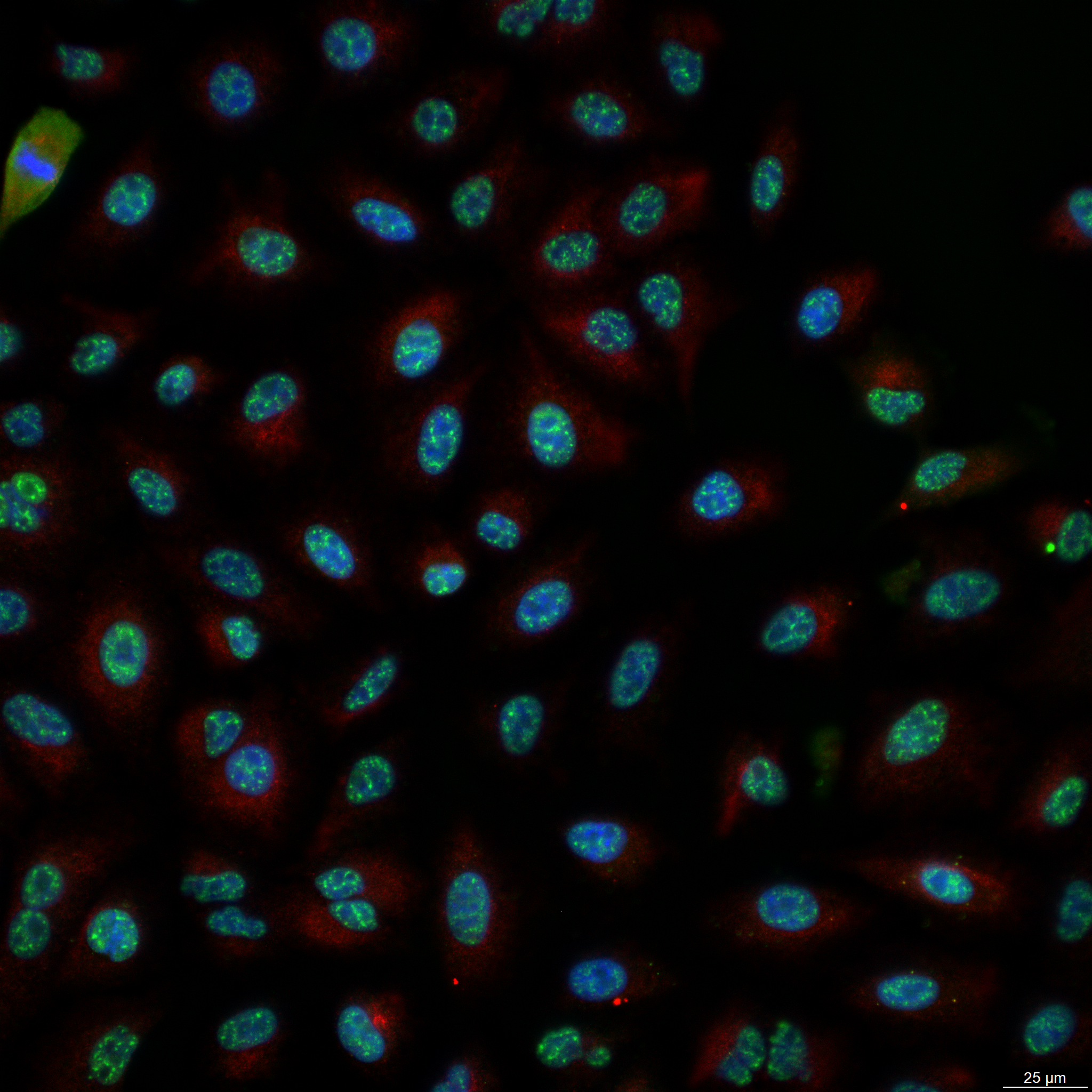

Supplement: Supplementary file 5 — Source data Fig. 1 [file 44318_2025_421_MOESM5_ESM.zip › Figure 1/Figure 1D/Control.tif]

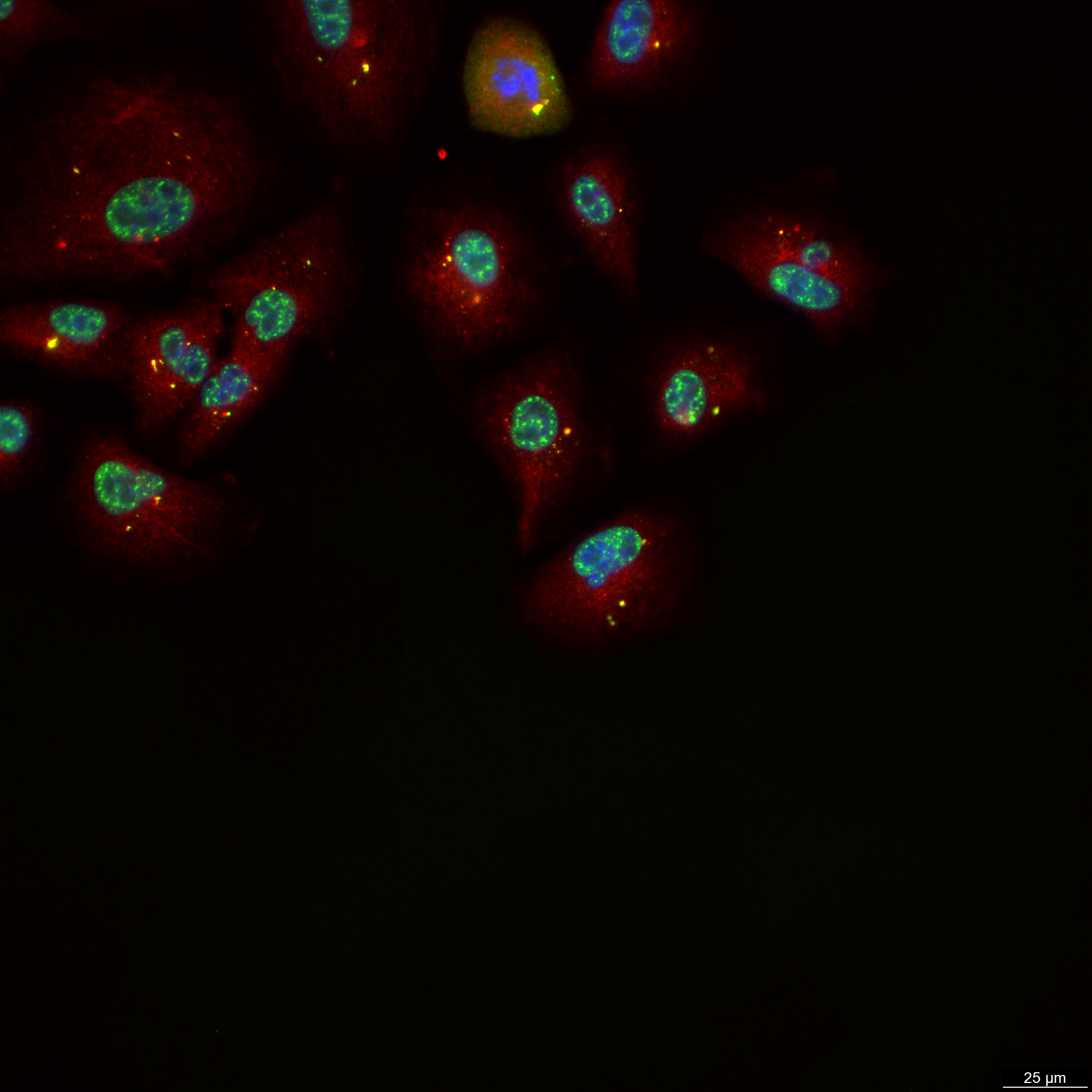

Supplement: Supplementary file 5 — Source data Fig. 1 [file 44318_2025_421_MOESM5_ESM.zip › Figure 1/Figure 1D/IFNγ.tif]

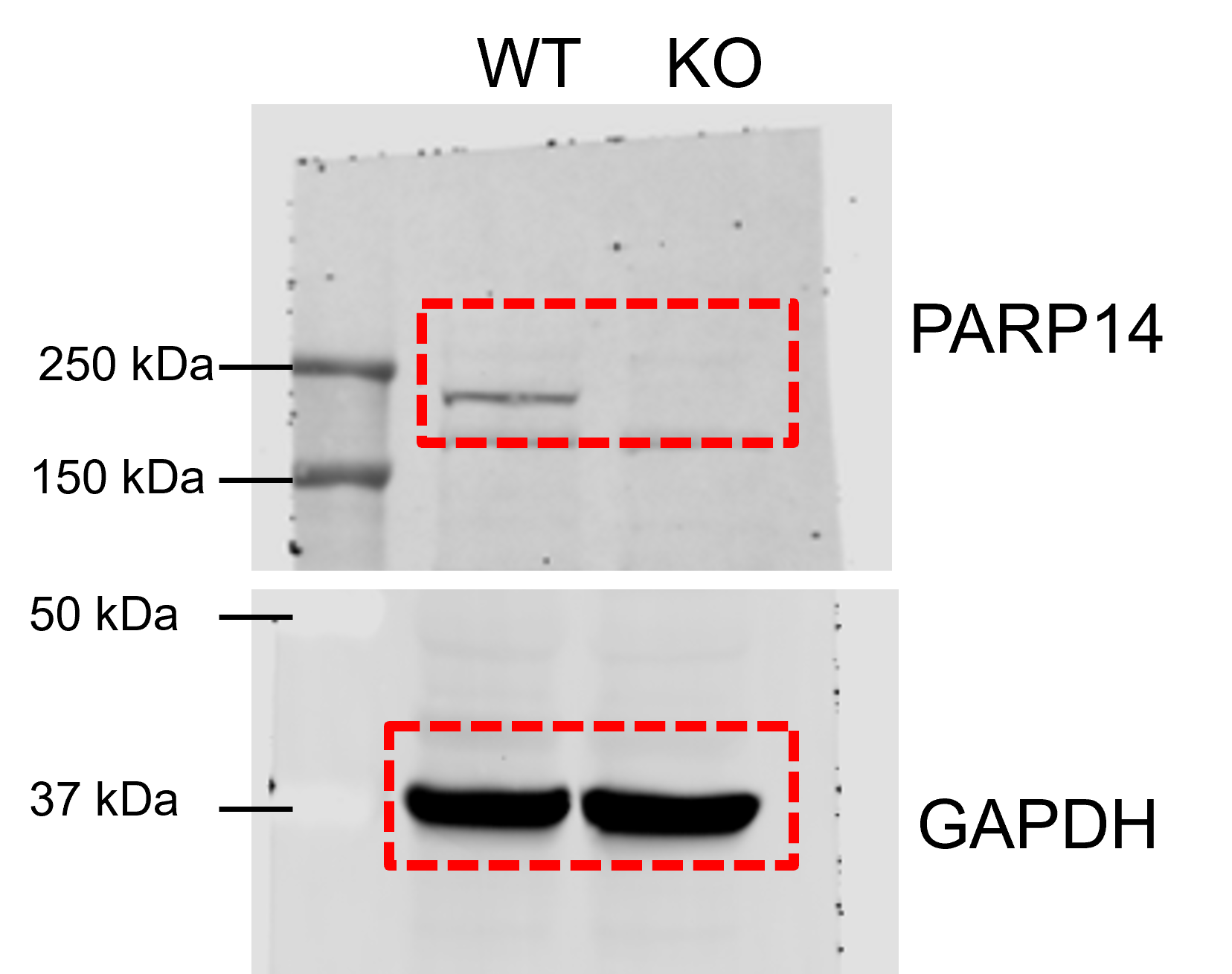

Supplement: Supplementary file 5 — Source data Fig. 1 [file 44318_2025_421_MOESM5_ESM.zip › Figure 1/Figure 1E.tif]

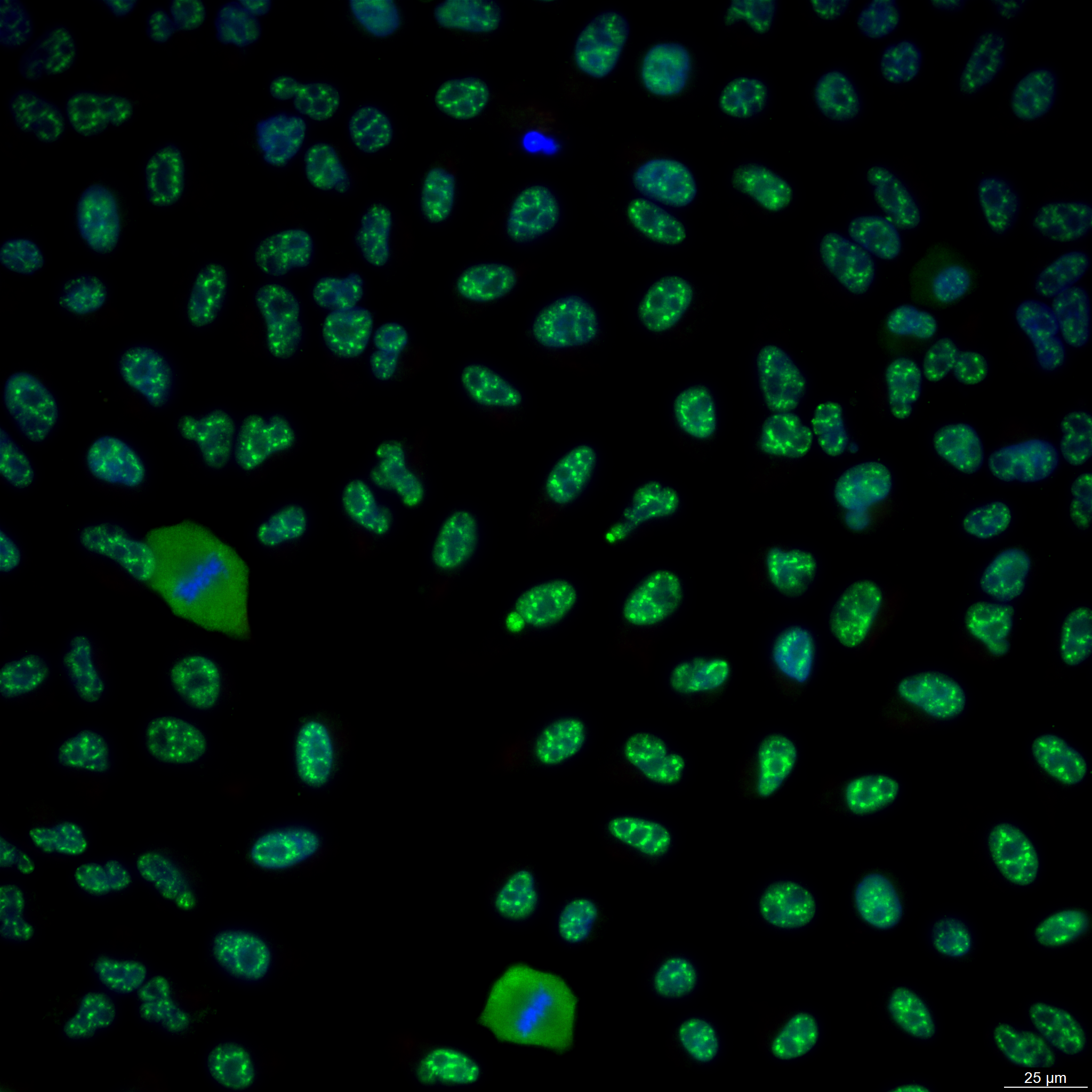

Supplement: Supplementary file 5 — Source data Fig. 1 [file 44318_2025_421_MOESM5_ESM.zip › Figure 1/Figure 1F/P14KO+IFNy.tif]

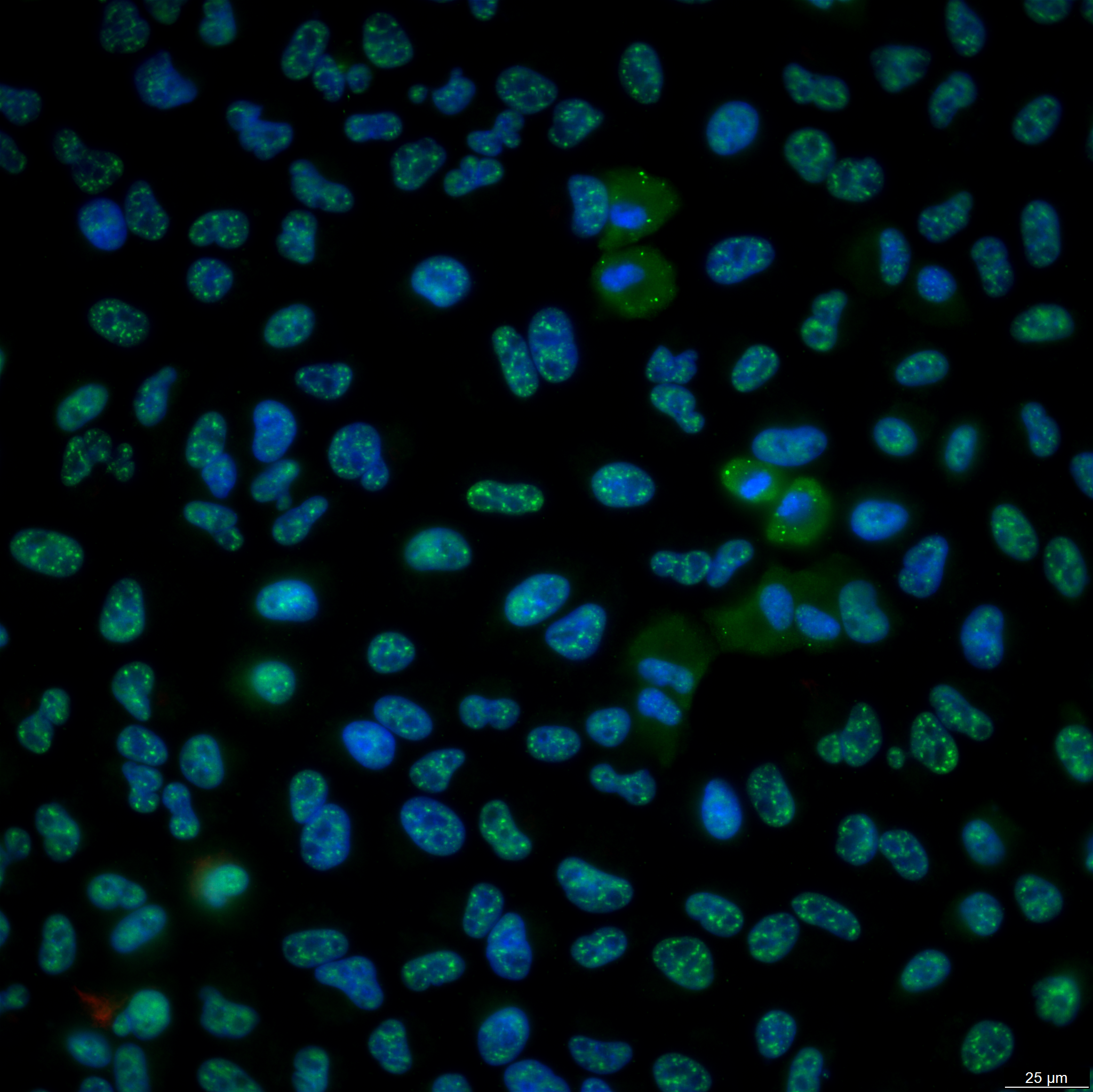

Supplement: Supplementary file 5 — Source data Fig. 1 [file 44318_2025_421_MOESM5_ESM.zip › Figure 1/Figure 1F/P14KO.tif]

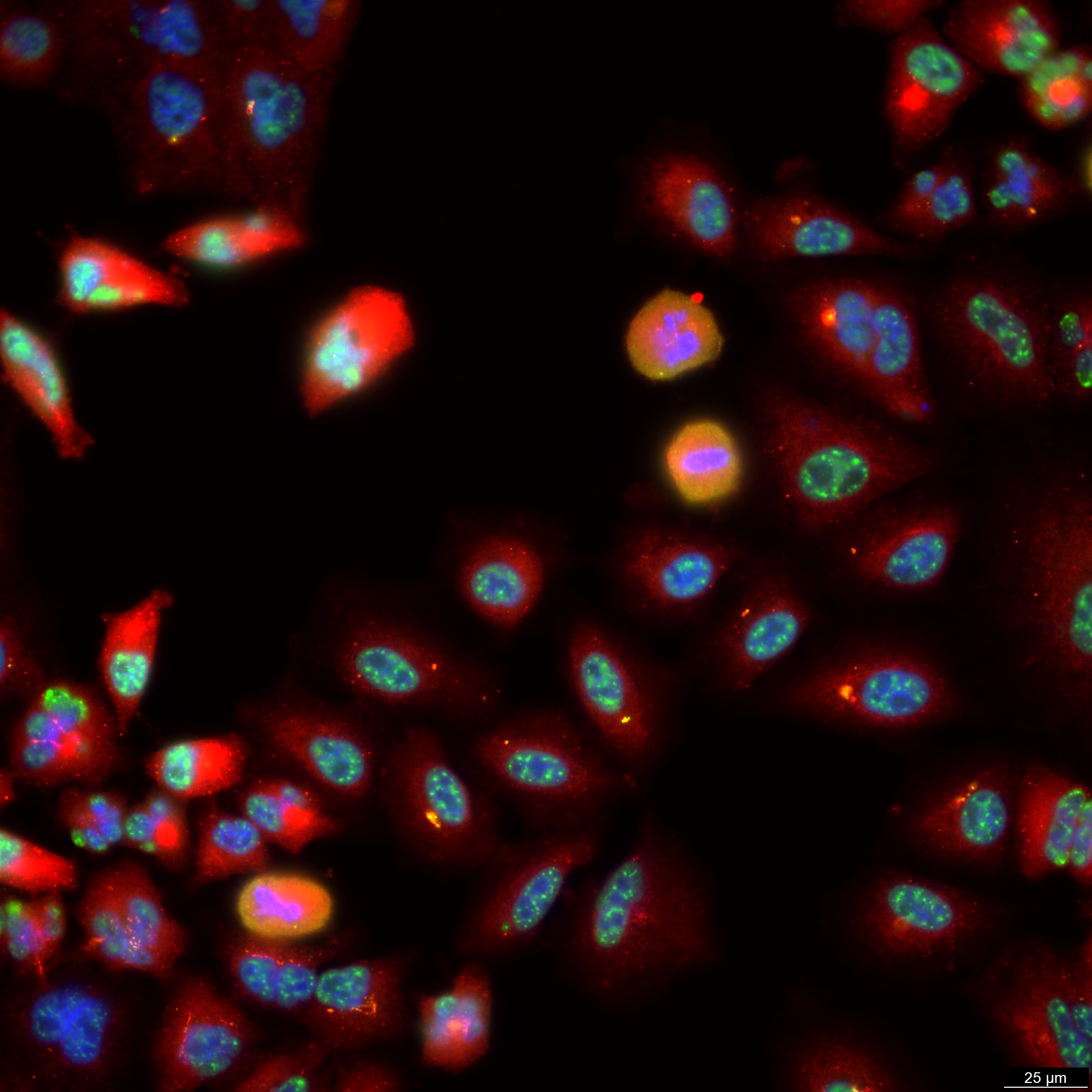

Supplement: Supplementary file 5 — Source data Fig. 1 [file 44318_2025_421_MOESM5_ESM.zip › Figure 1/Figure 1F/WT+IFNy.tif]

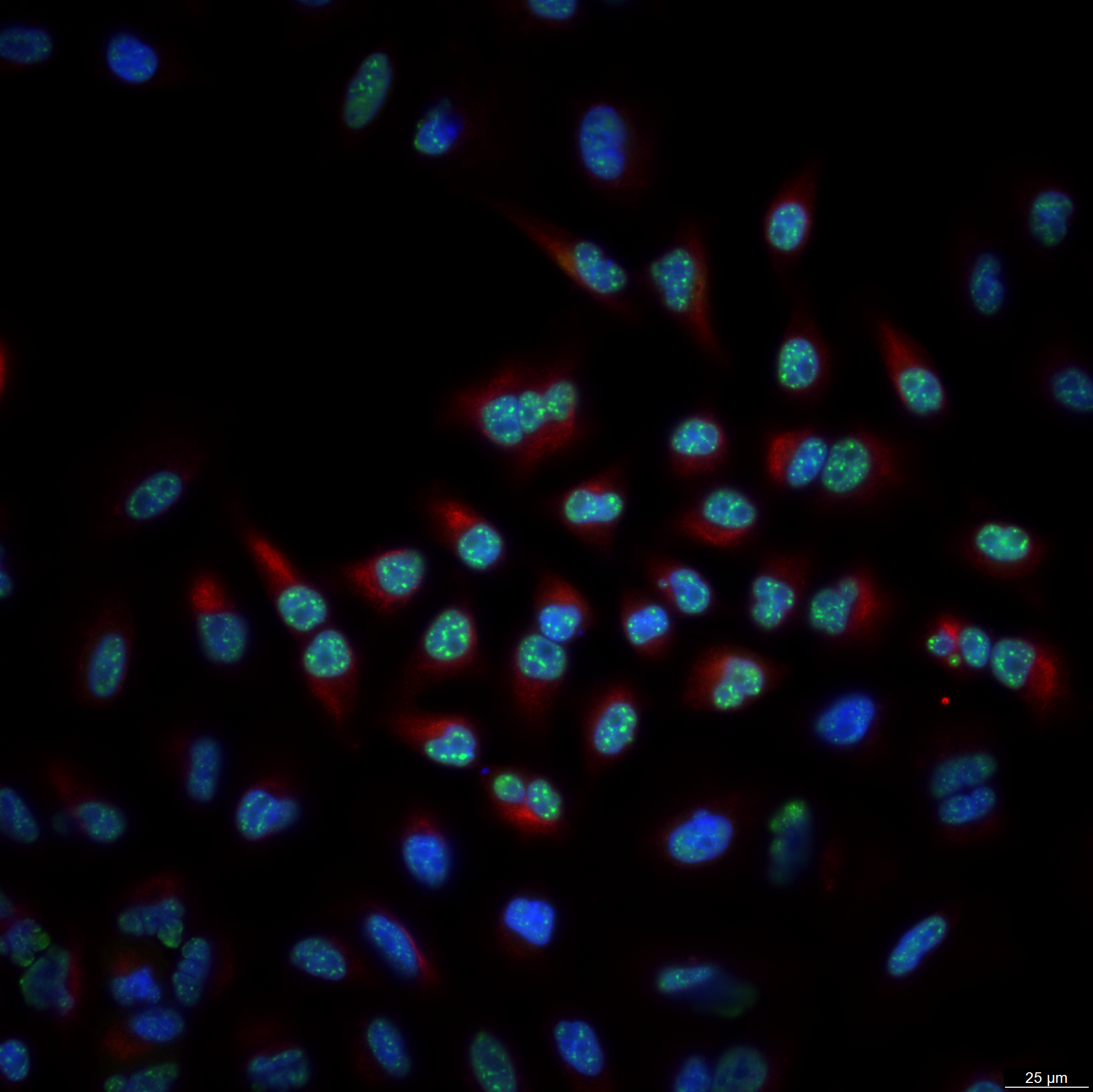

Supplement: Supplementary file 5 — Source data Fig. 1 [file 44318_2025_421_MOESM5_ESM.zip › Figure 1/Figure 1F/WT.tif]

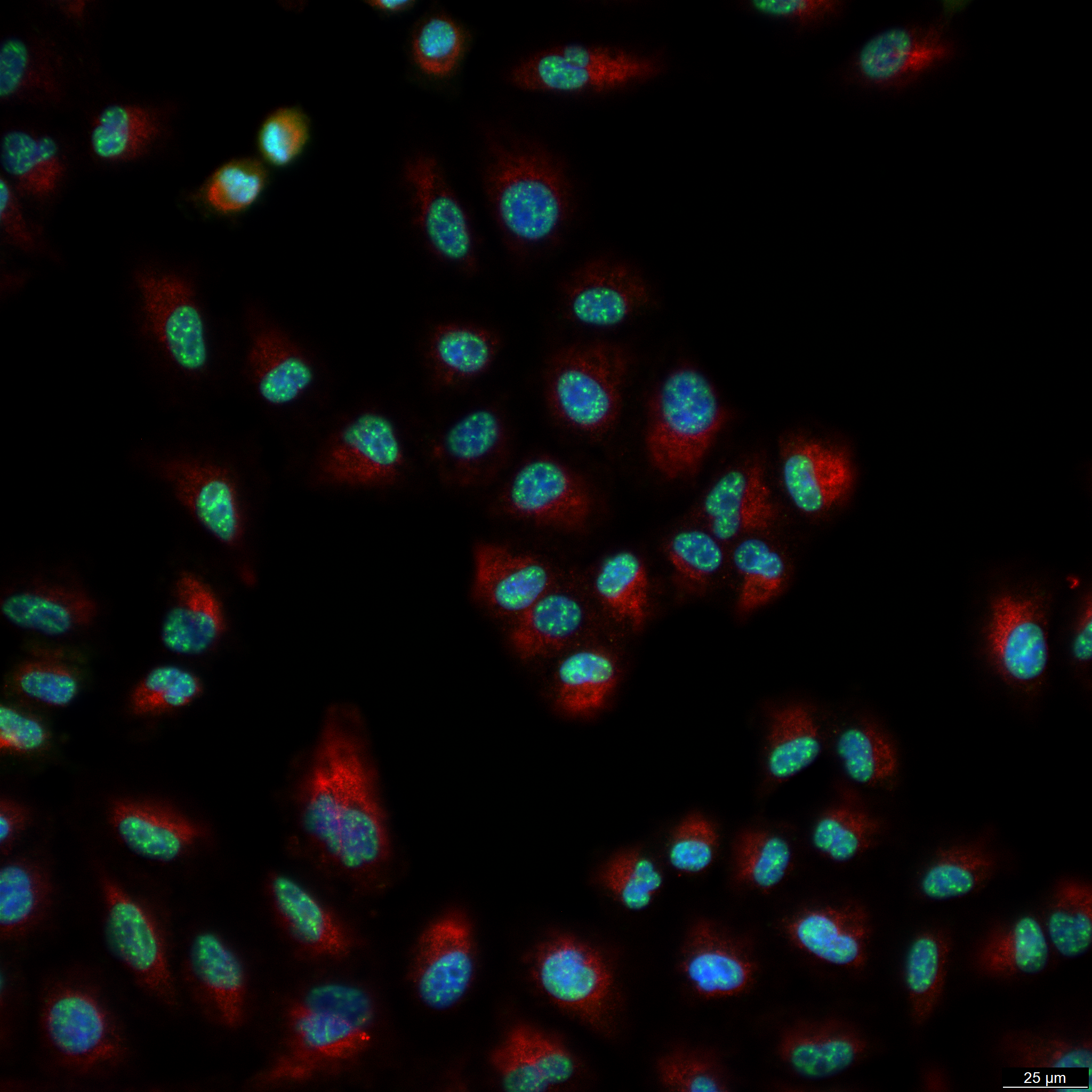

Supplement: Supplementary file 5 — Source data Fig. 1 [file 44318_2025_421_MOESM5_ESM.zip › Figure 1/Figure 1G/Control 1_overlay.tif]

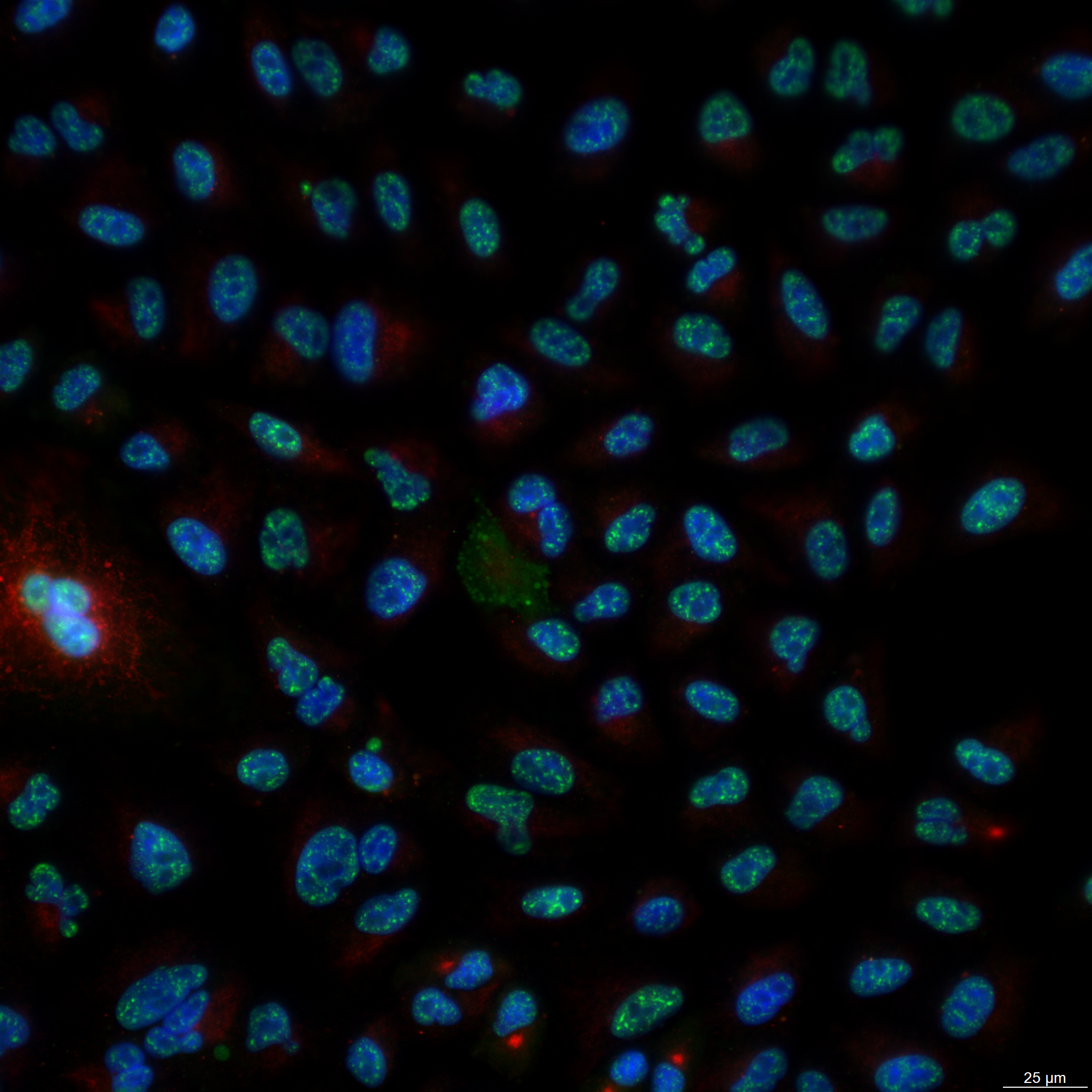

Supplement: Supplementary file 5 — Source data Fig. 1 [file 44318_2025_421_MOESM5_ESM.zip › Figure 1/Figure 1G/IFN γ+RBN012811 1 h.tif]

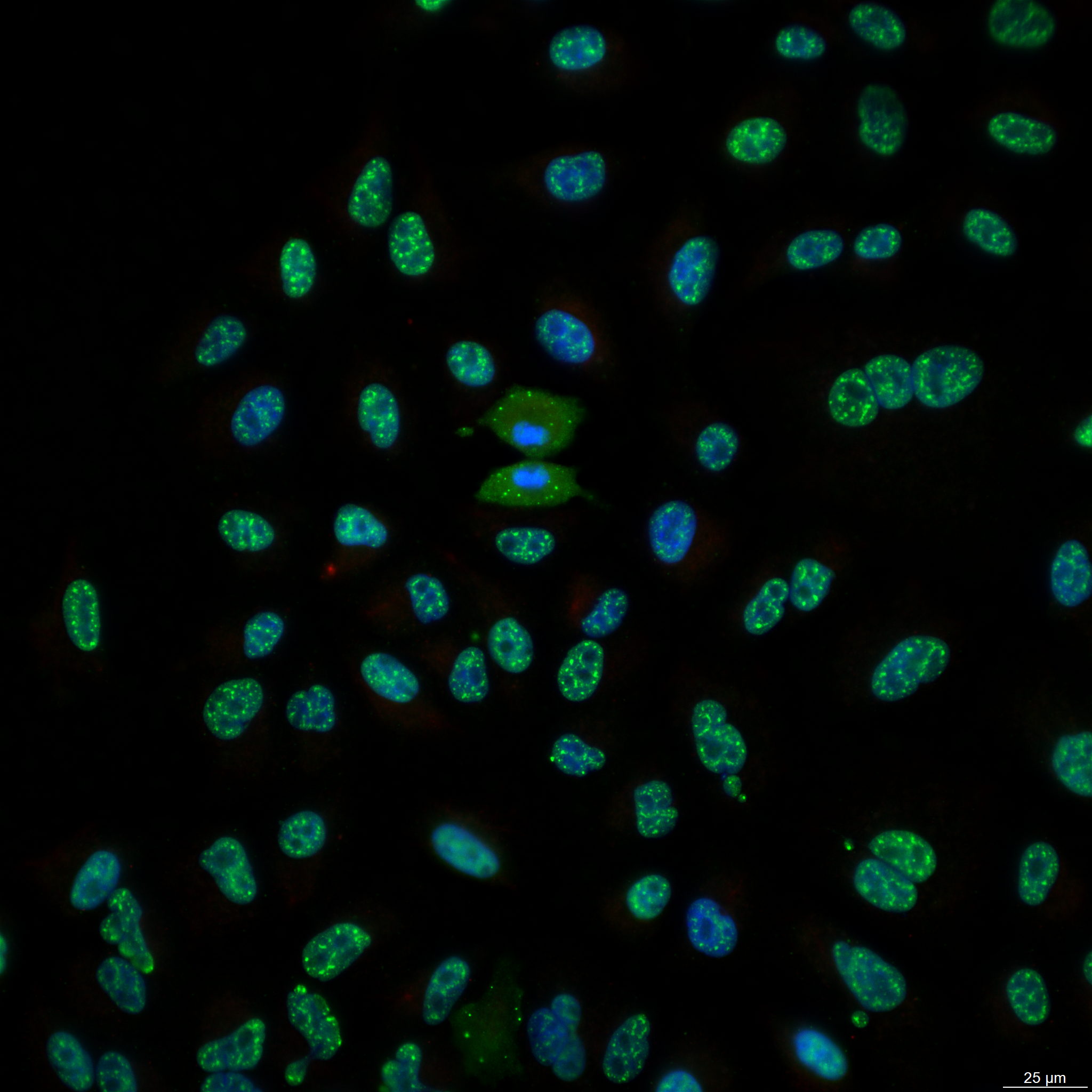

Supplement: Supplementary file 5 — Source data Fig. 1 [file 44318_2025_421_MOESM5_ESM.zip › Figure 1/Figure 1G/IFN γ+RBN012811 3 h.tif]

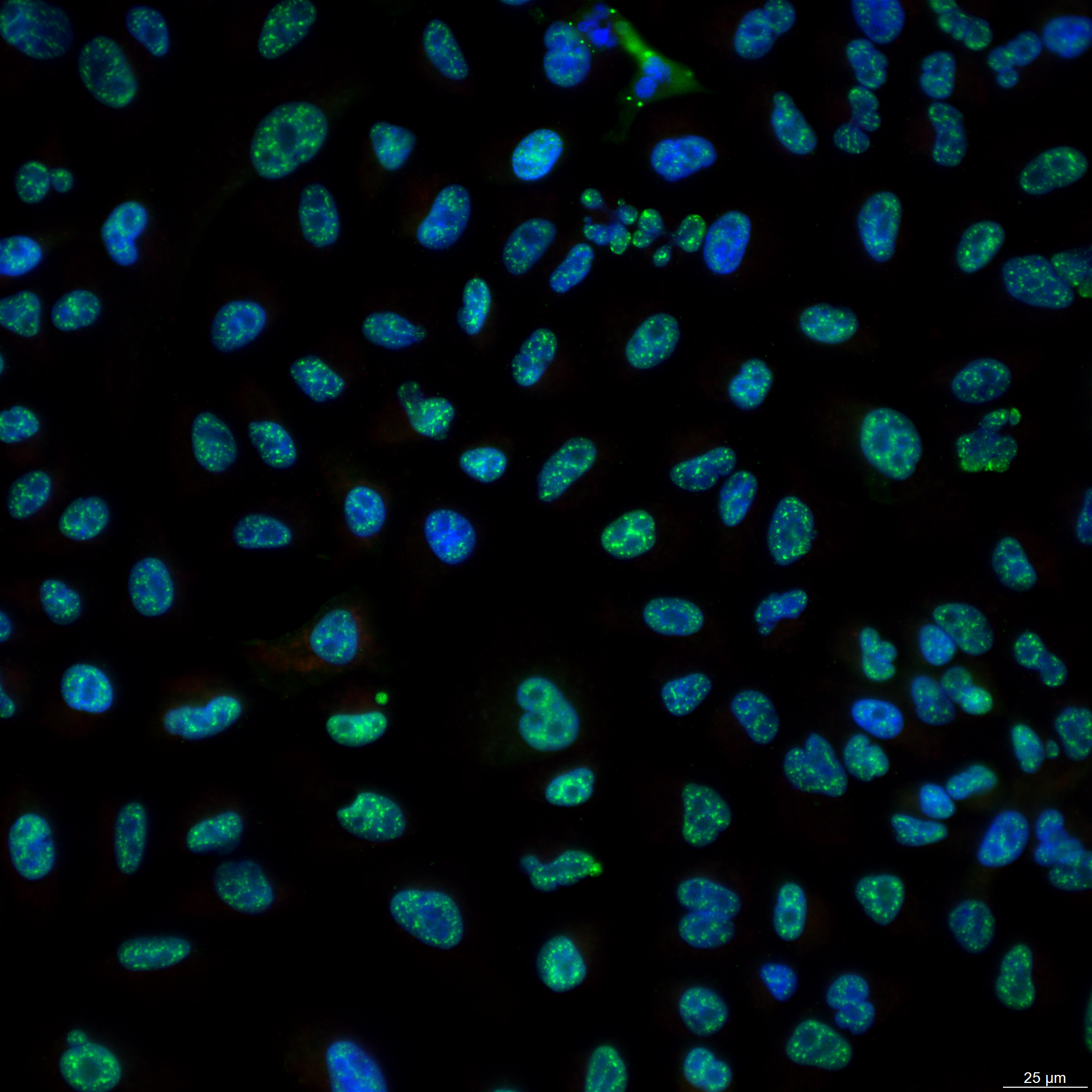

Supplement: Supplementary file 5 — Source data Fig. 1 [file 44318_2025_421_MOESM5_ESM.zip › Figure 1/Figure 1G/IFN γ+RBN012811 6 h.tif]

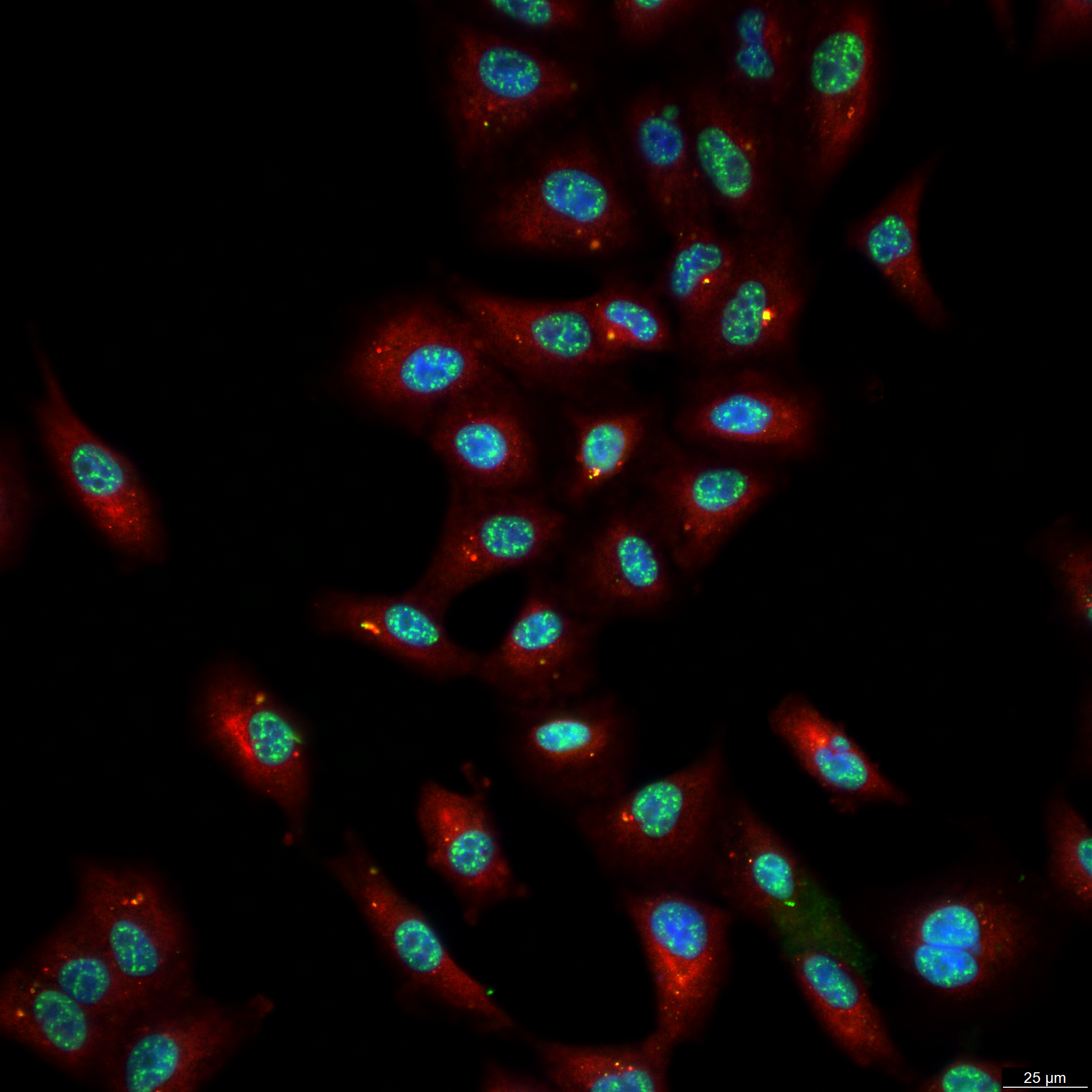

Supplement: Supplementary file 5 — Source data Fig. 1 [file 44318_2025_421_MOESM5_ESM.zip › Figure 1/Figure 1G/IFN γ+RBN013527 1 h.tif]

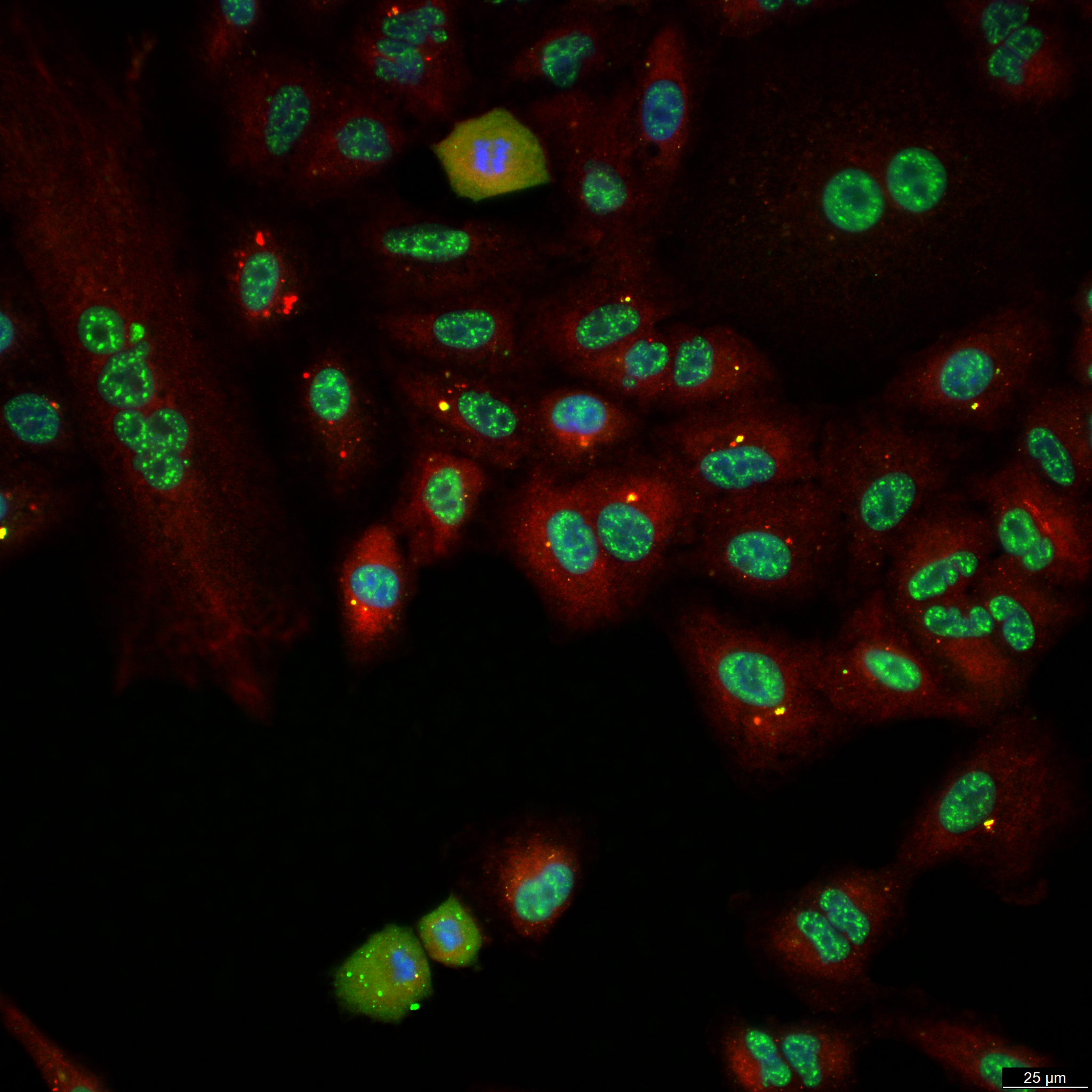

Supplement: Supplementary file 5 — Source data Fig. 1 [file 44318_2025_421_MOESM5_ESM.zip › Figure 1/Figure 1G/IFN γ+RBN013527 3 h.tif]

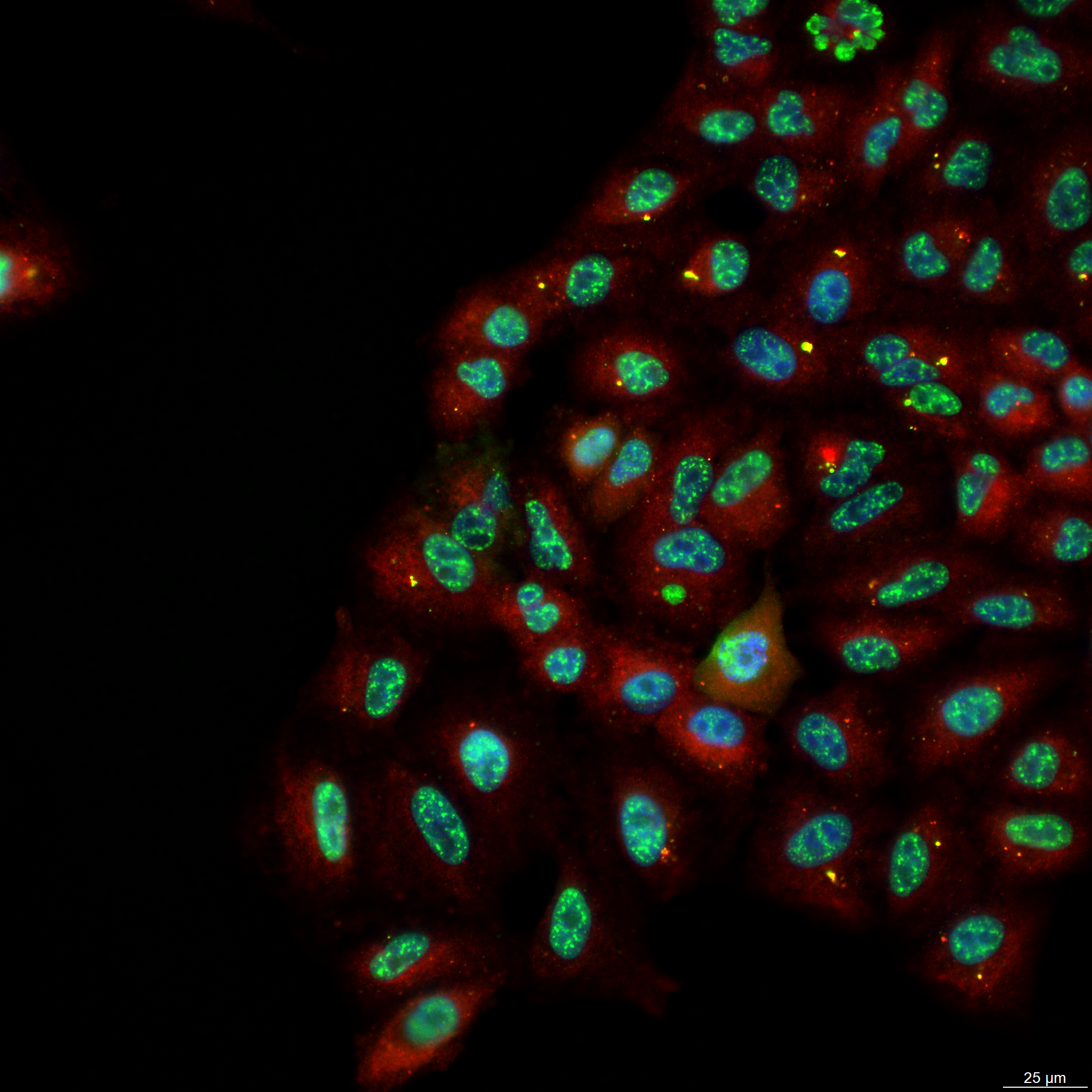

Supplement: Supplementary file 5 — Source data Fig. 1 [file 44318_2025_421_MOESM5_ESM.zip › Figure 1/Figure 1G/IFN γ+RBN013527 6 h.tif]

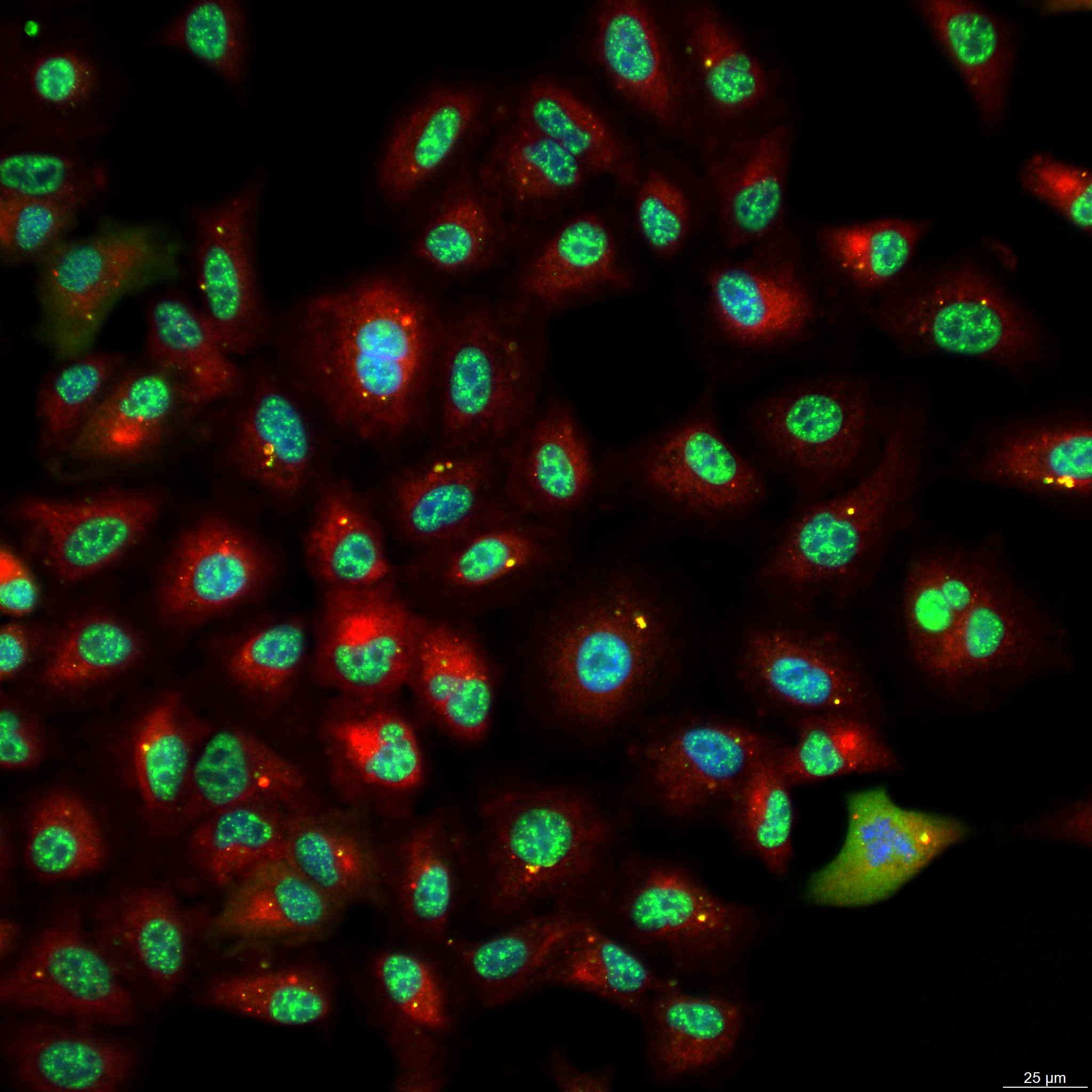

Supplement: Supplementary file 5 — Source data Fig. 1 [file 44318_2025_421_MOESM5_ESM.zip › Figure 1/Figure 1G/IFN γ.tif]

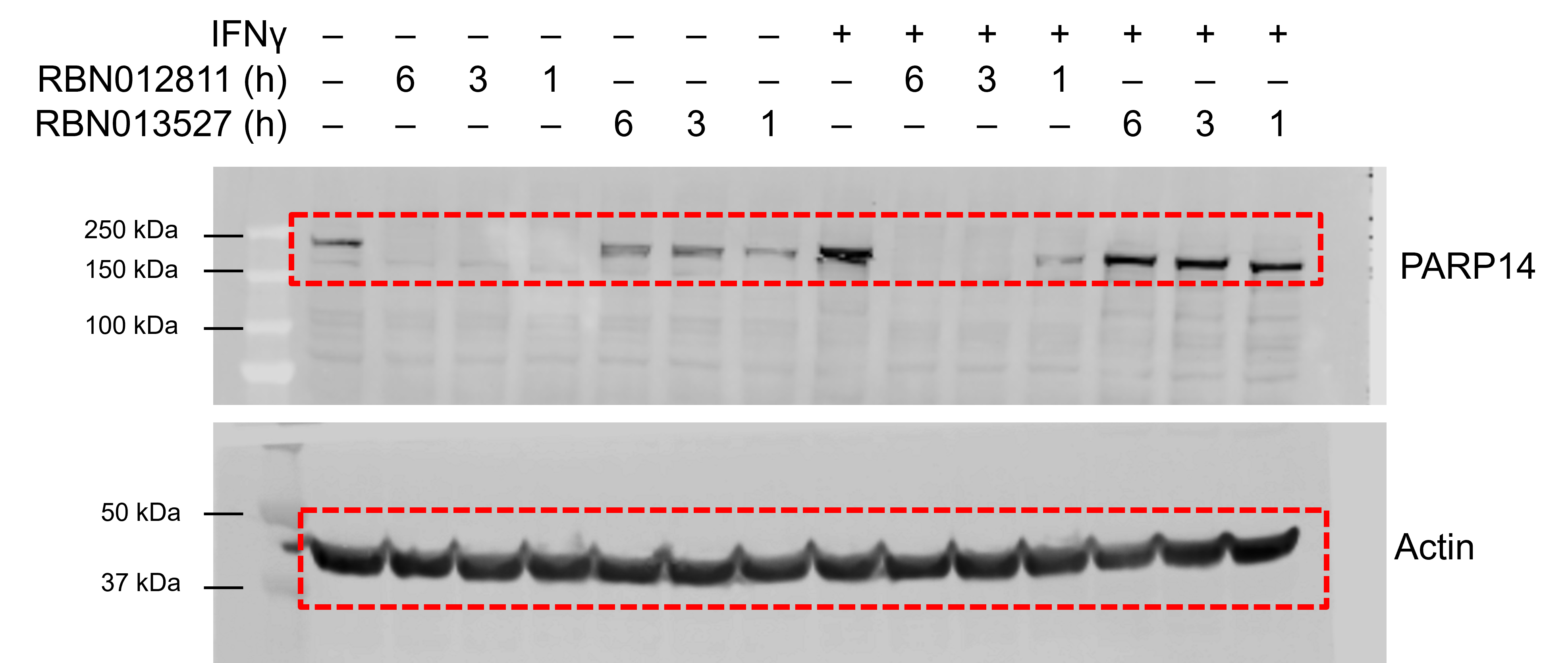

Supplement: Supplementary file 5 — Source data Fig. 1 [file 44318_2025_421_MOESM5_ESM.zip › Figure 1/Figure 1H.tif]

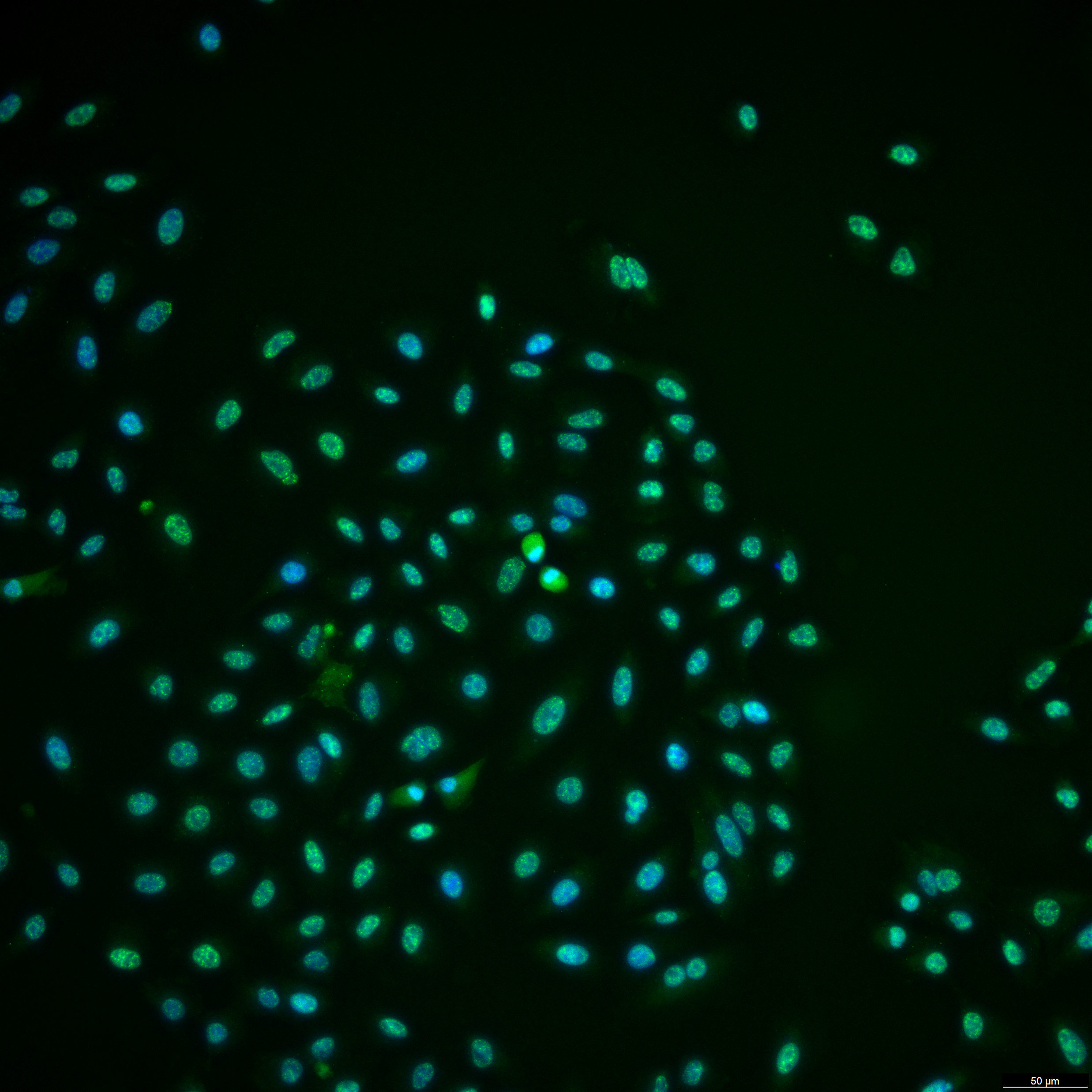

Supplement: Supplementary file 6 — Source data Fig. 2 [file 44318_2025_421_MOESM6_ESM.zip › Figure 2/Figure 2B/Control.tif]

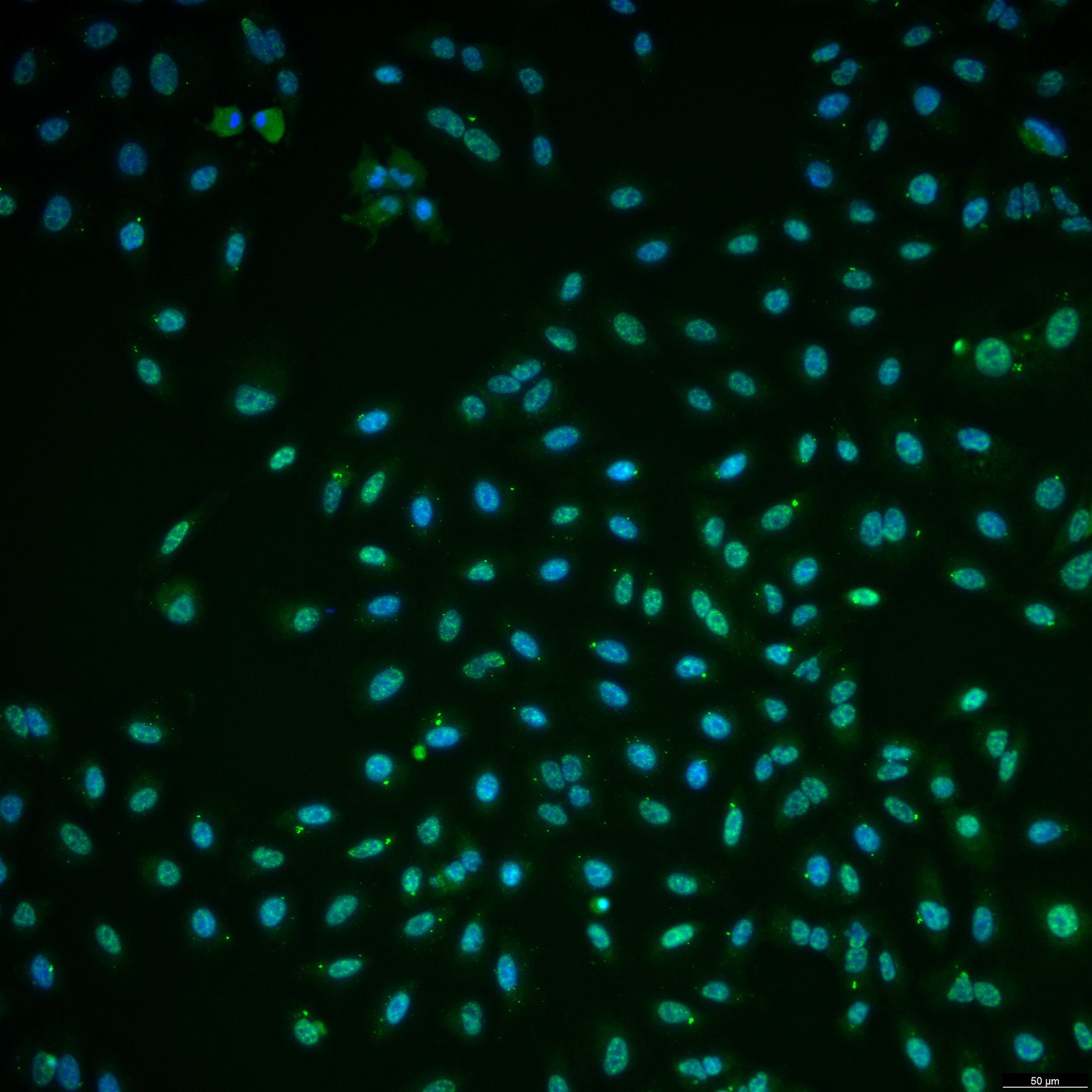

Supplement: Supplementary file 6 — Source data Fig. 2 [file 44318_2025_421_MOESM6_ESM.zip › Figure 2/Figure 2B/IFN γ+ RBN 0μM.tif]

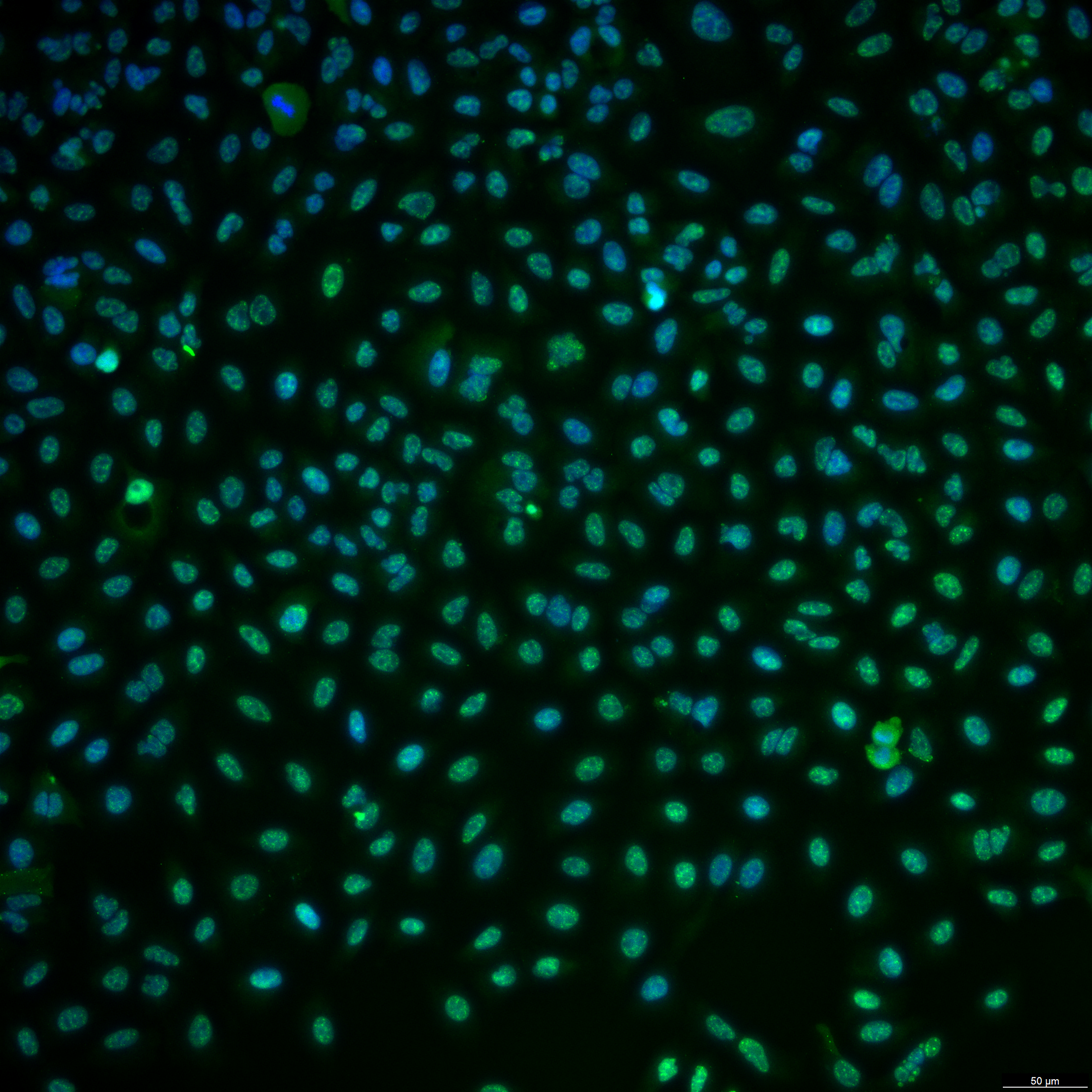

Supplement: Supplementary file 6 — Source data Fig. 2 [file 44318_2025_421_MOESM6_ESM.zip › Figure 2/Figure 2B/IFN γ+ RBN 100 nM.tif]

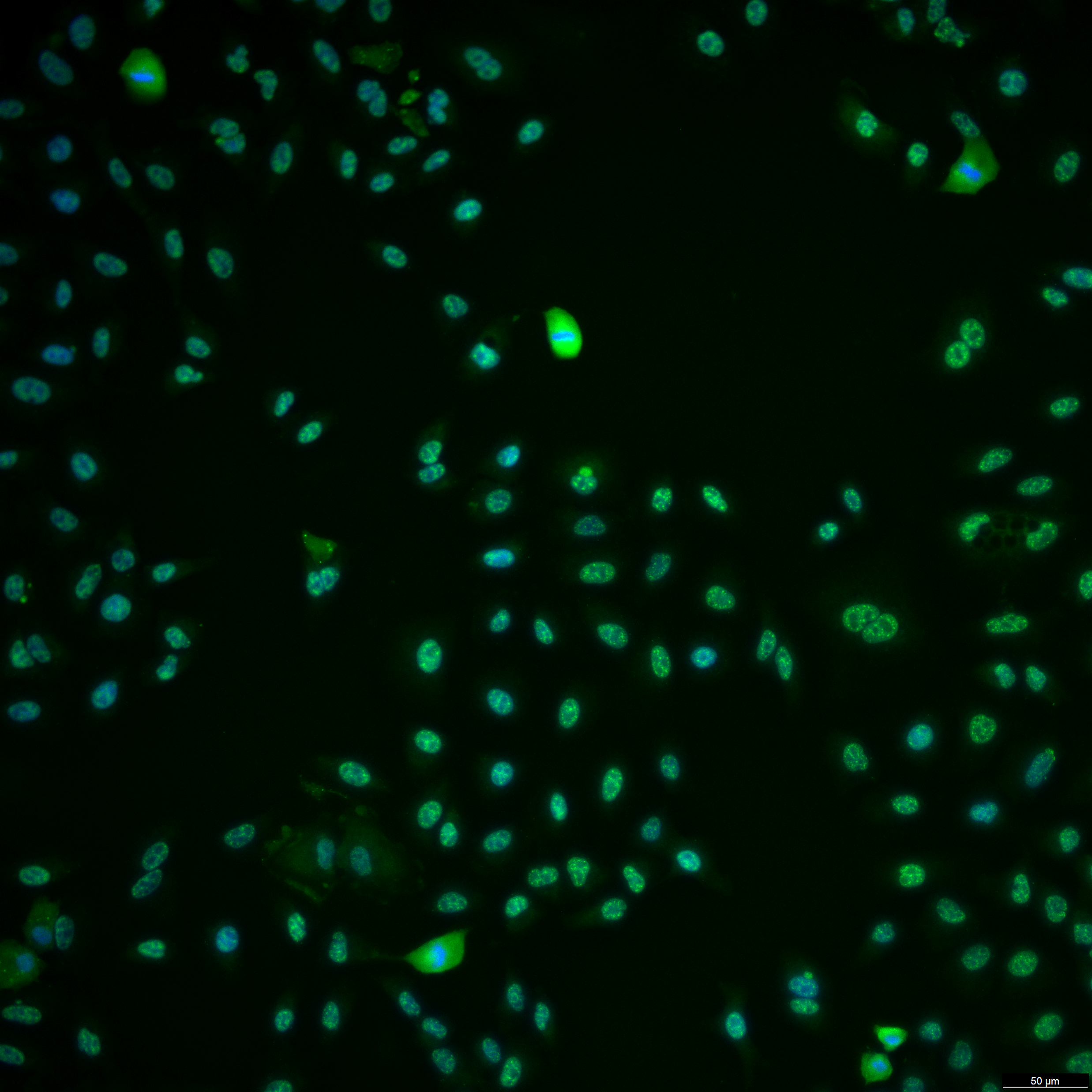

Supplement: Supplementary file 6 — Source data Fig. 2 [file 44318_2025_421_MOESM6_ESM.zip › Figure 2/Figure 2B/IFN γ+ RBN 10μM.tif]

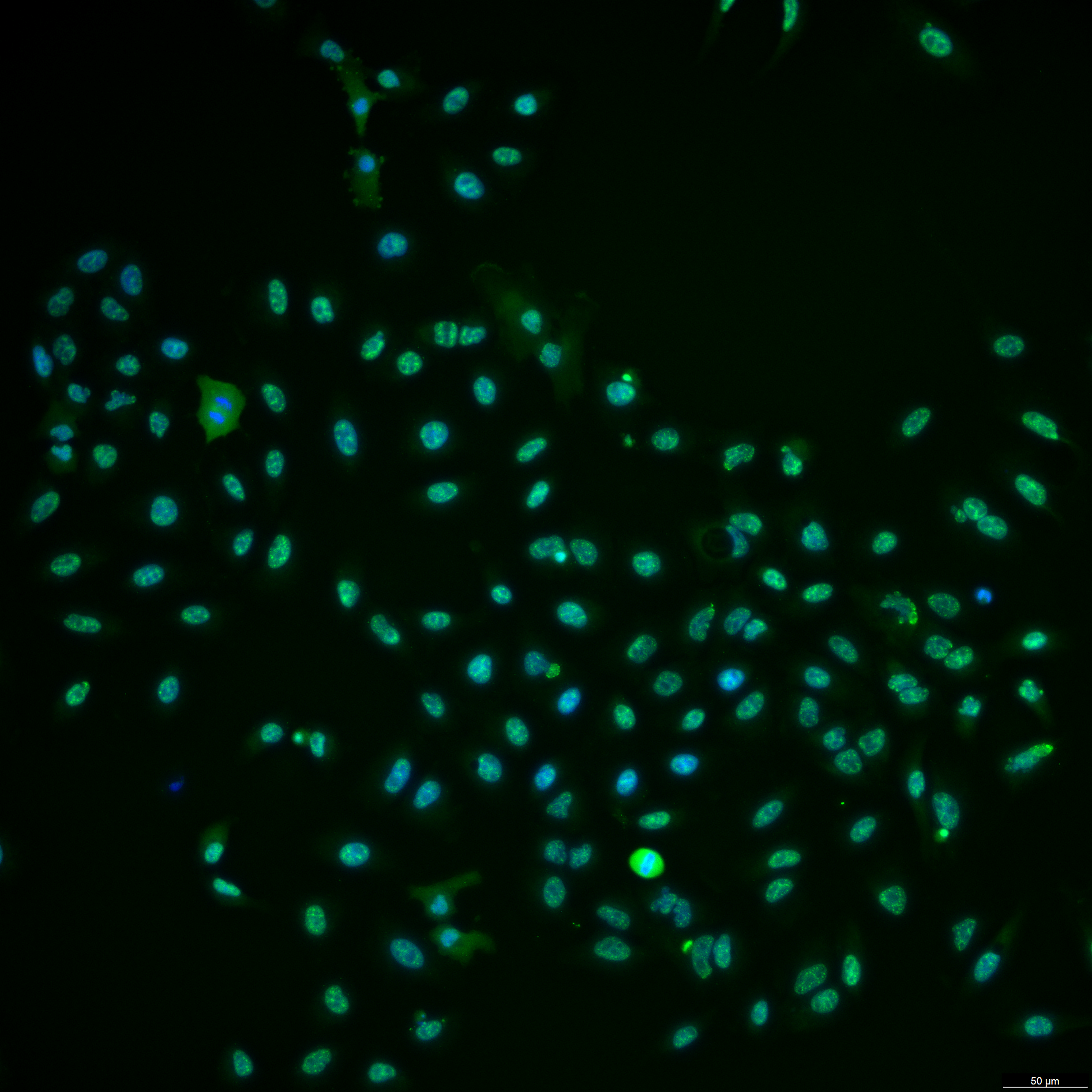

Supplement: Supplementary file 6 — Source data Fig. 2 [file 44318_2025_421_MOESM6_ESM.zip › Figure 2/Figure 2B/IFN γ+ RBN 1μM.tif]

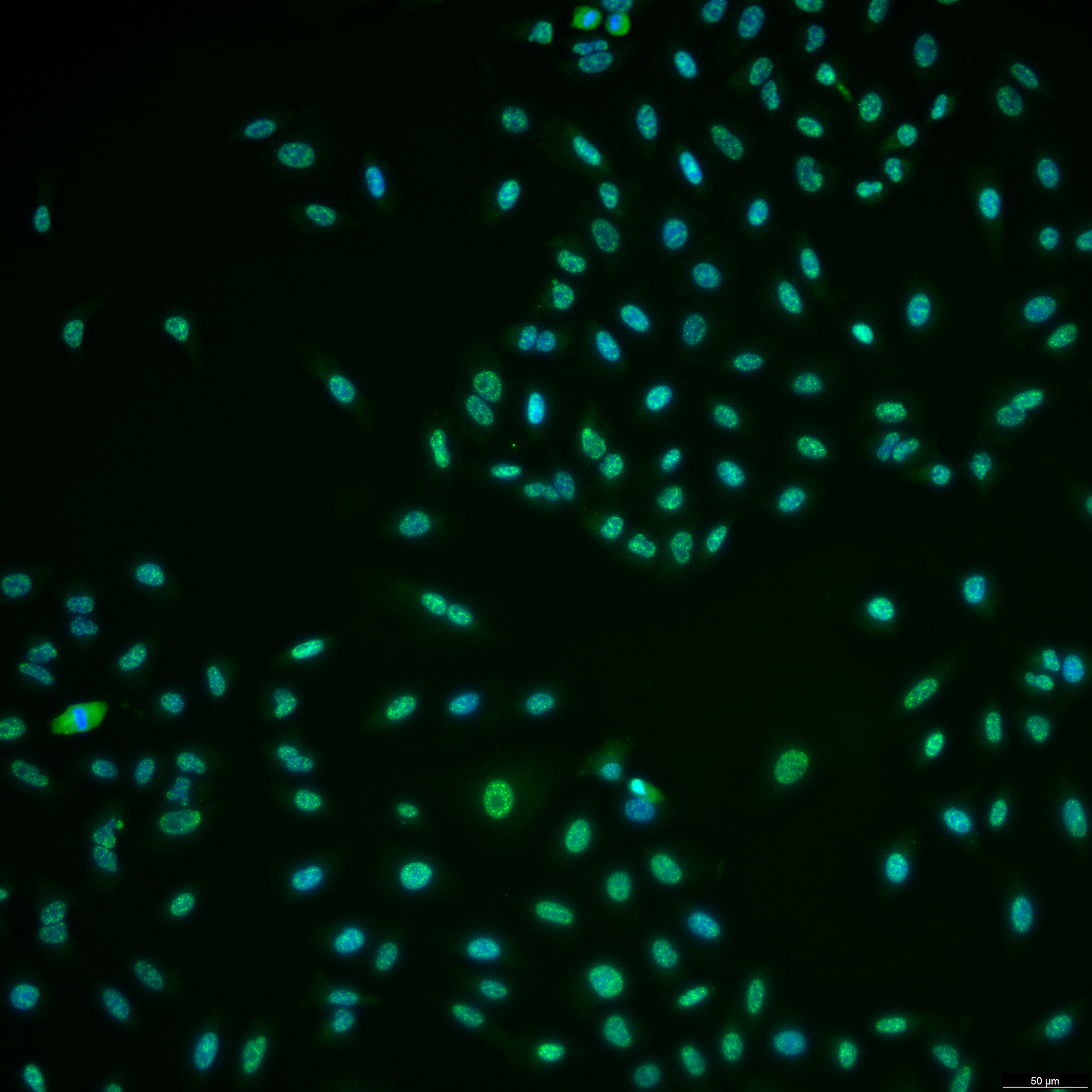

Supplement: Supplementary file 6 — Source data Fig. 2 [file 44318_2025_421_MOESM6_ESM.zip › Figure 2/Figure 2B/IFN γ+ RBN 5μM.tif]

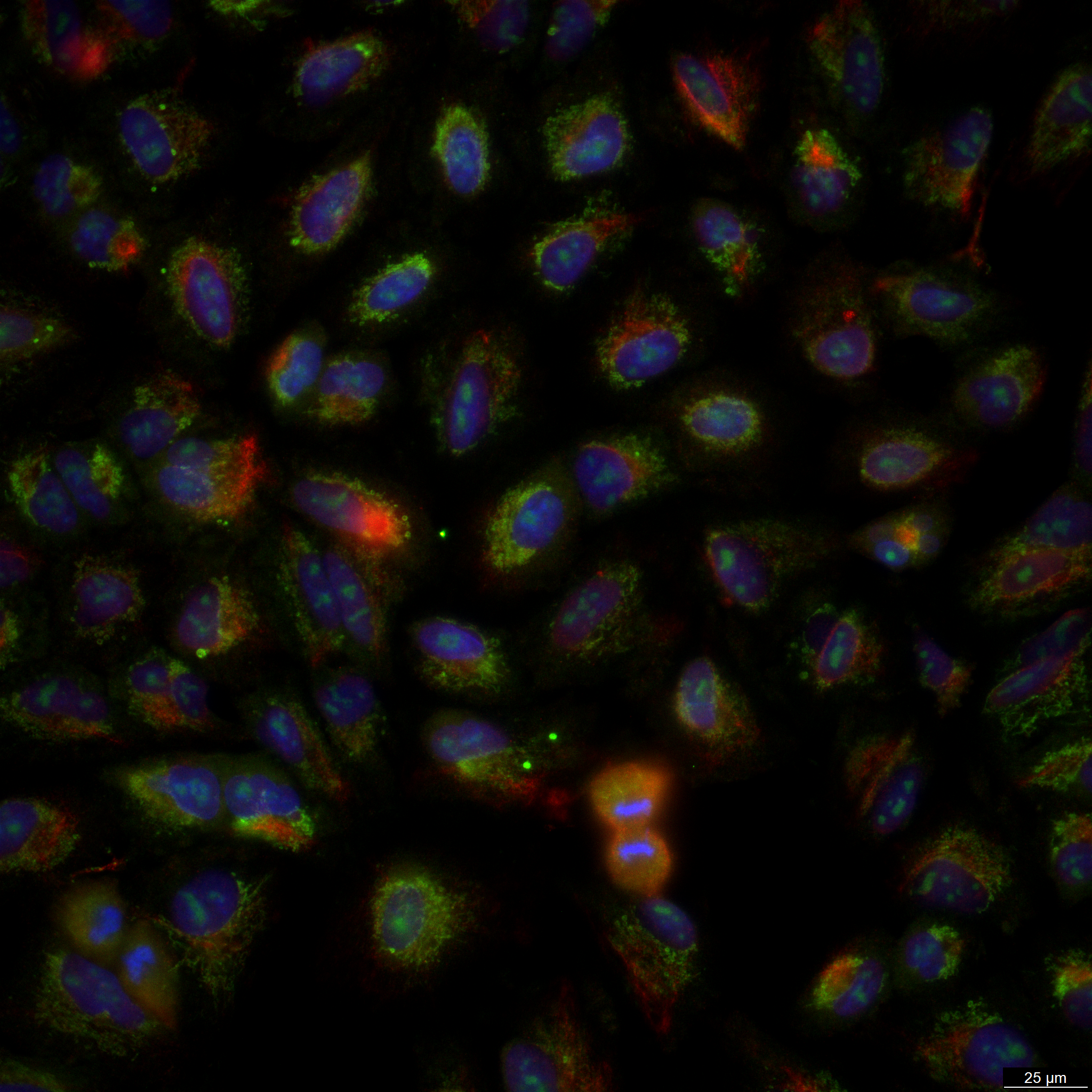

Supplement: Supplementary file 6 — Source data Fig. 2 [file 44318_2025_421_MOESM6_ESM.zip › Figure 2/Figure 2C/Control.tif]

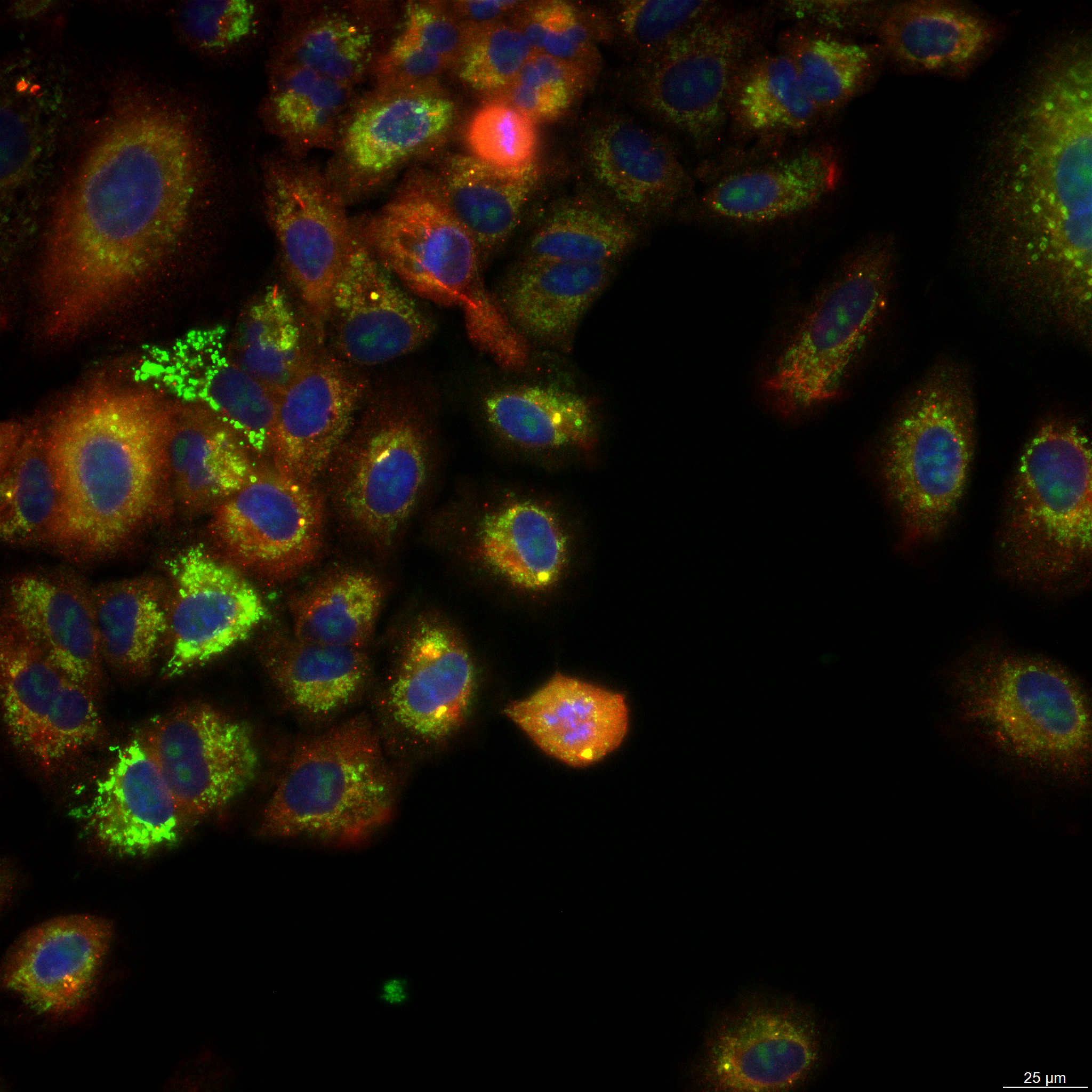

Supplement: Supplementary file 6 — Source data Fig. 2 [file 44318_2025_421_MOESM6_ESM.zip › Figure 2/Figure 2C/lFNγ.tif]

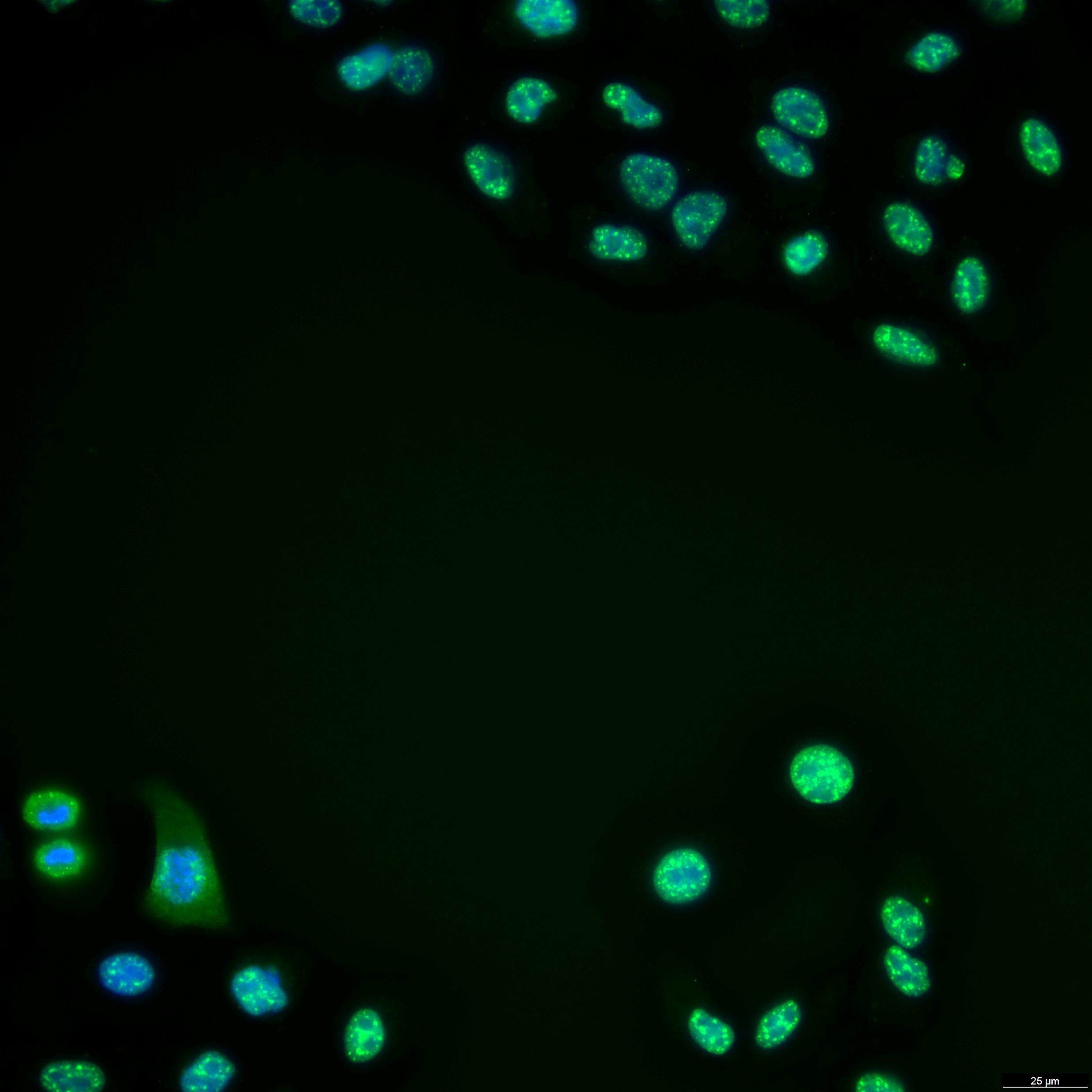

Supplement: Supplementary file 6 — Source data Fig. 2 [file 44318_2025_421_MOESM6_ESM.zip › Figure 2/Figure 2D/Control.tif]

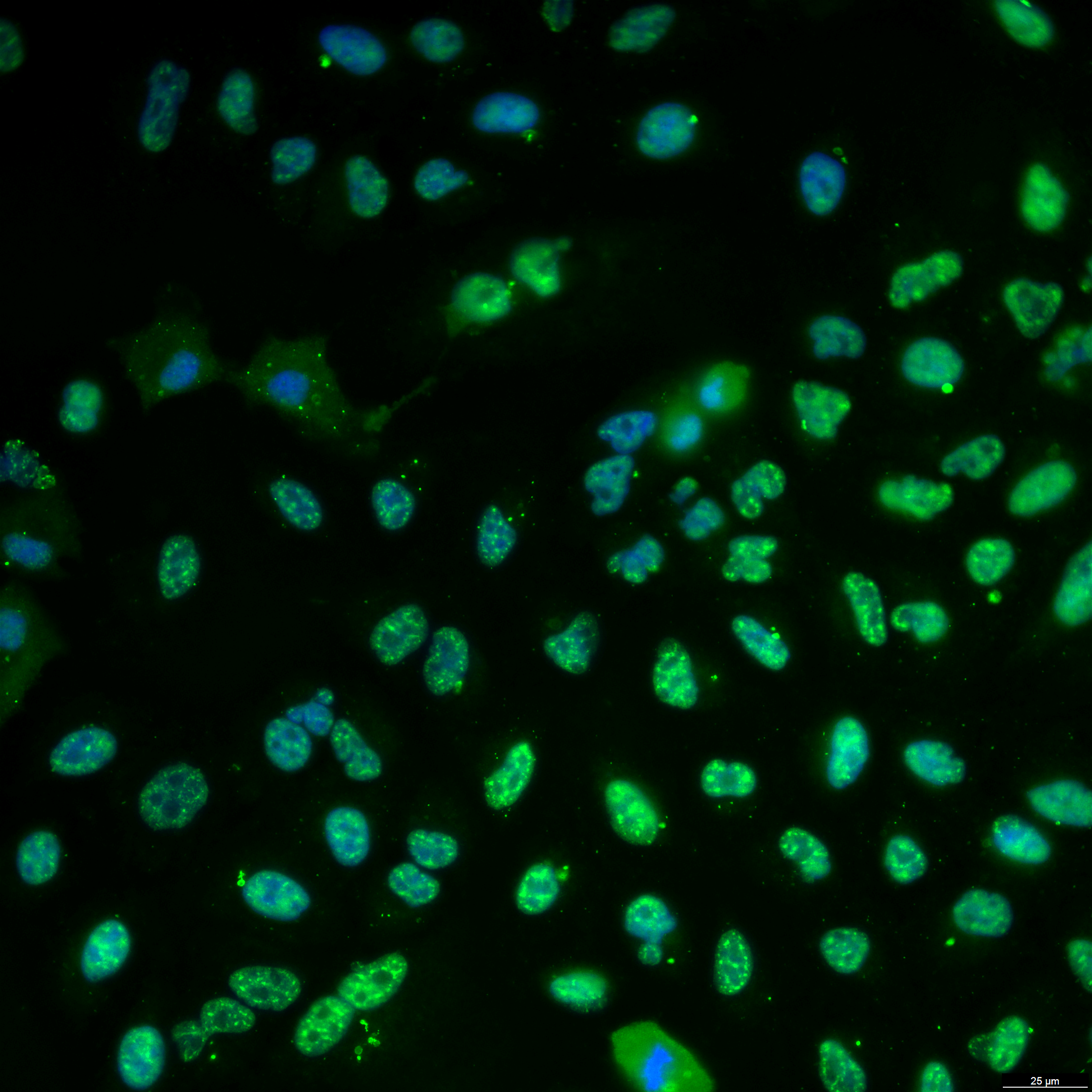

Supplement: Supplementary file 6 — Source data Fig. 2 [file 44318_2025_421_MOESM6_ESM.zip › Figure 2/Figure 2D/IFN γ+ RBN 0 h .tif]

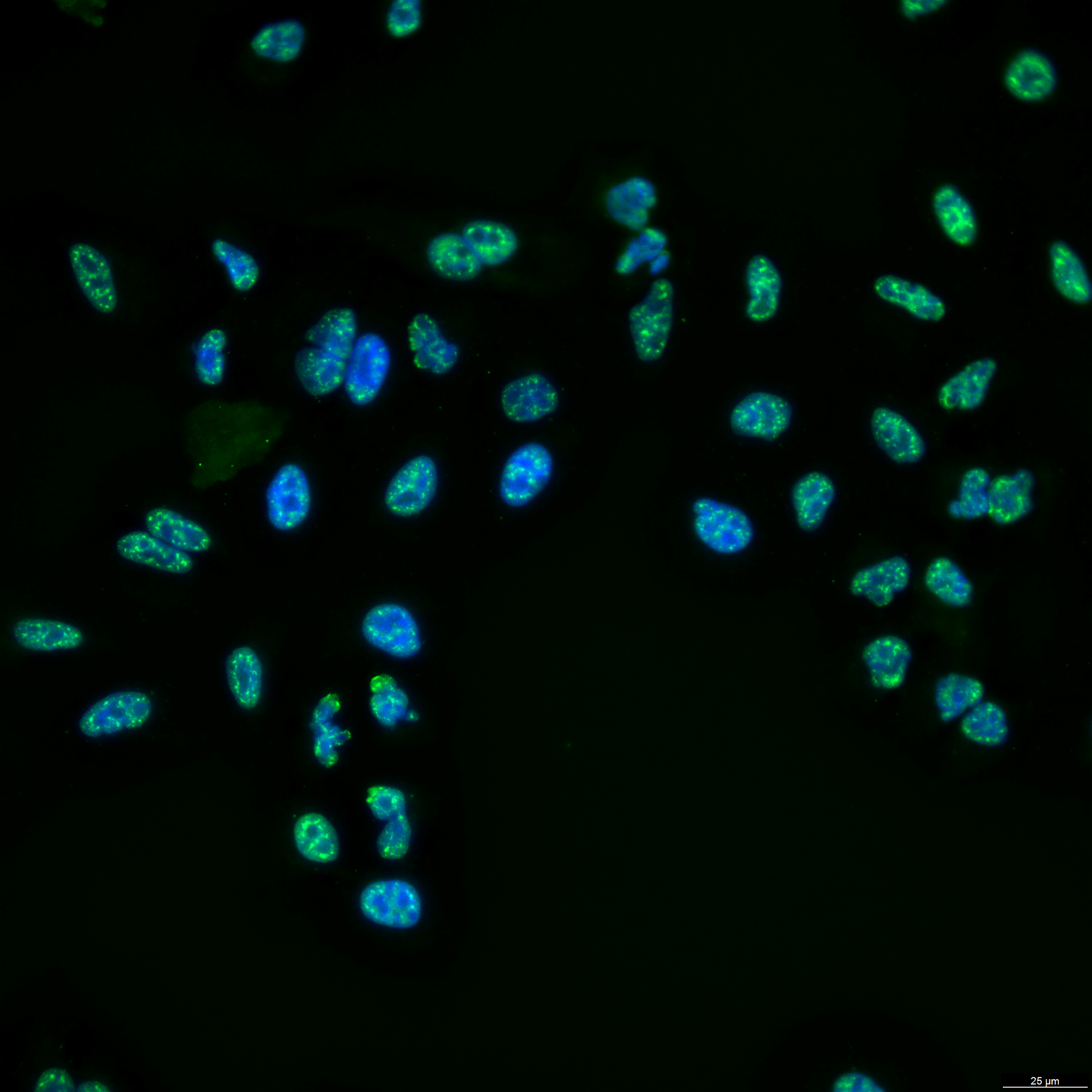

Supplement: Supplementary file 6 — Source data Fig. 2 [file 44318_2025_421_MOESM6_ESM.zip › Figure 2/Figure 2D/IFN γ+ RBN 1 h.tif]

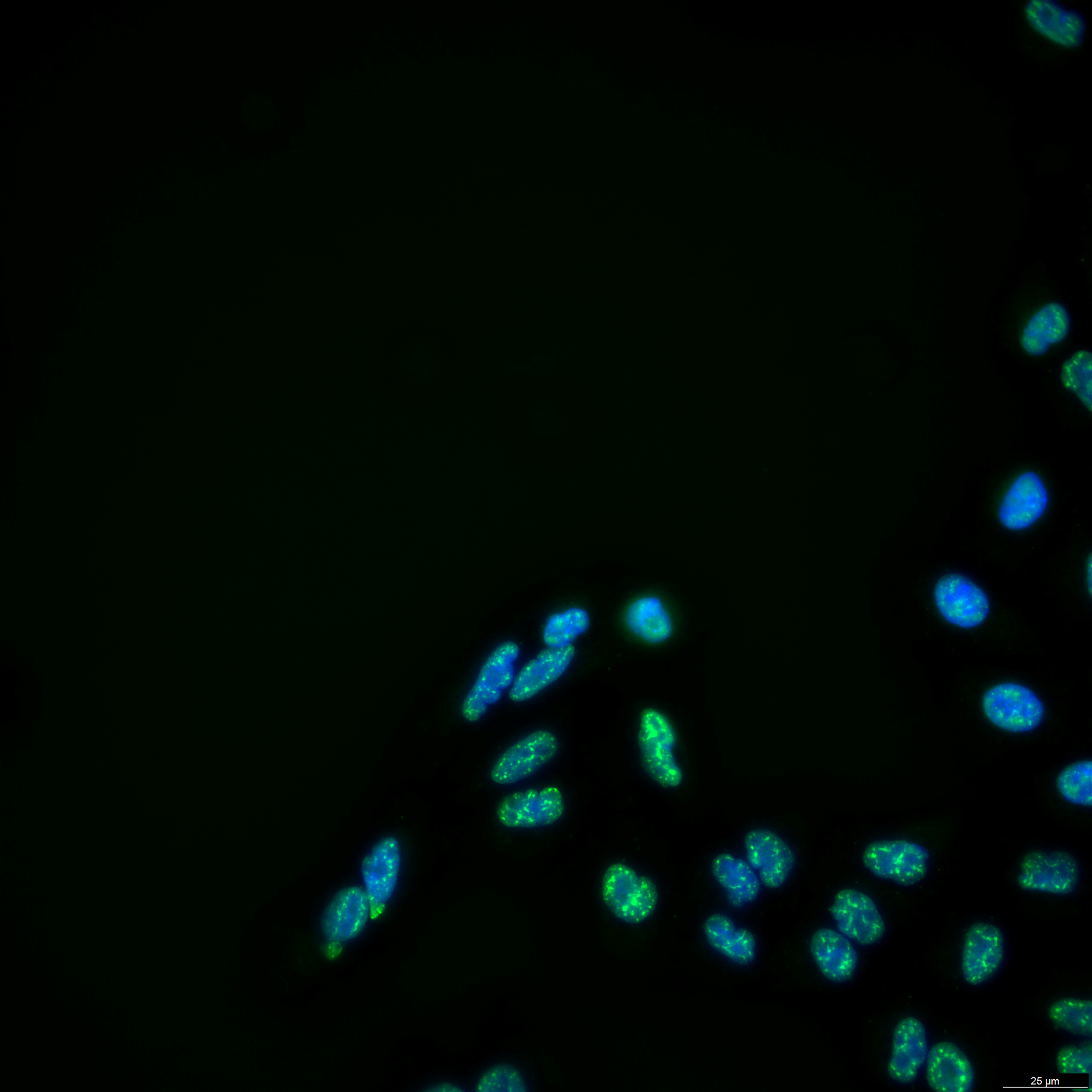

Supplement: Supplementary file 6 — Source data Fig. 2 [file 44318_2025_421_MOESM6_ESM.zip › Figure 2/Figure 2D/IFN γ+ RBN 10 h.tif]

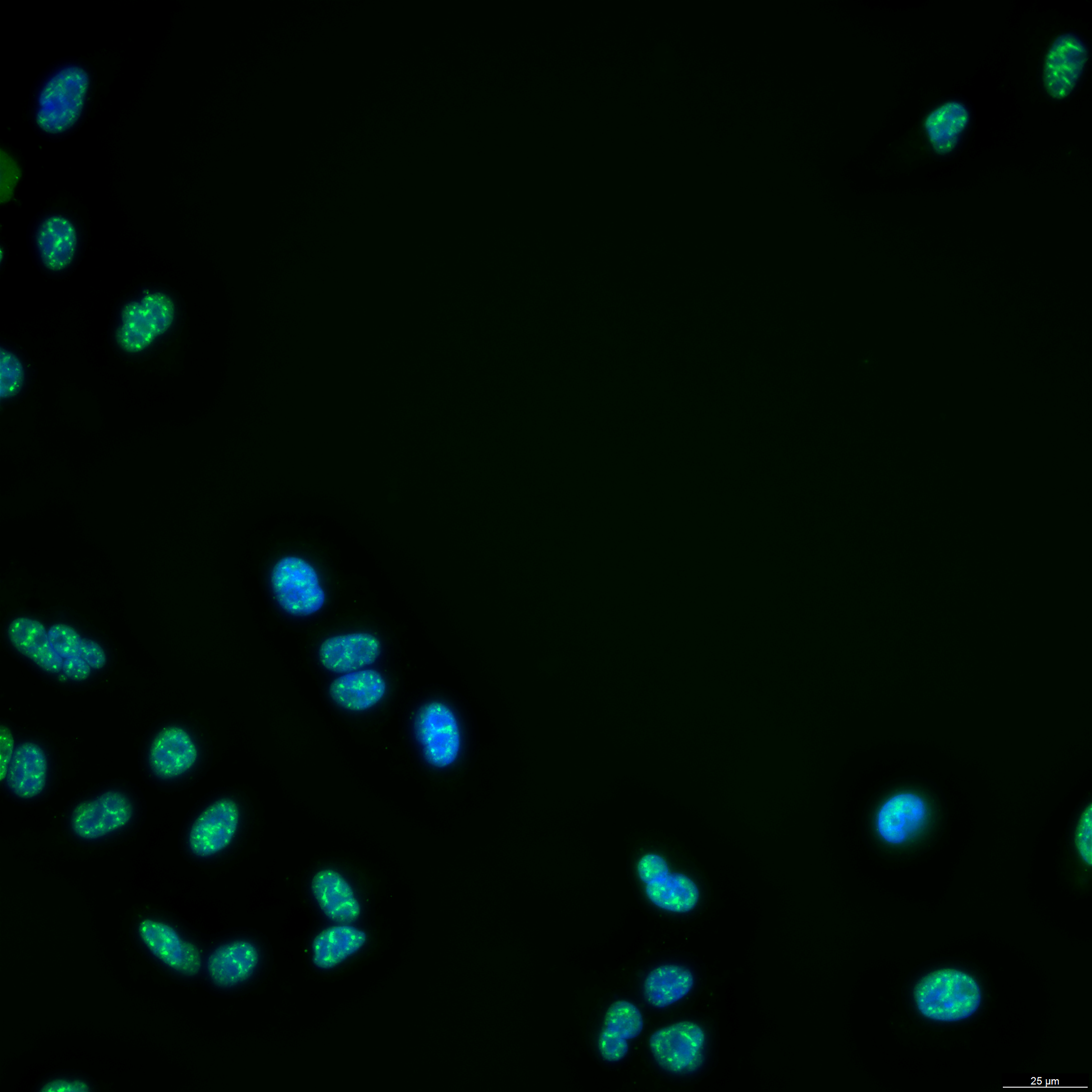

Supplement: Supplementary file 6 — Source data Fig. 2 [file 44318_2025_421_MOESM6_ESM.zip › Figure 2/Figure 2D/IFN γ+ RBN 3 h.tif]

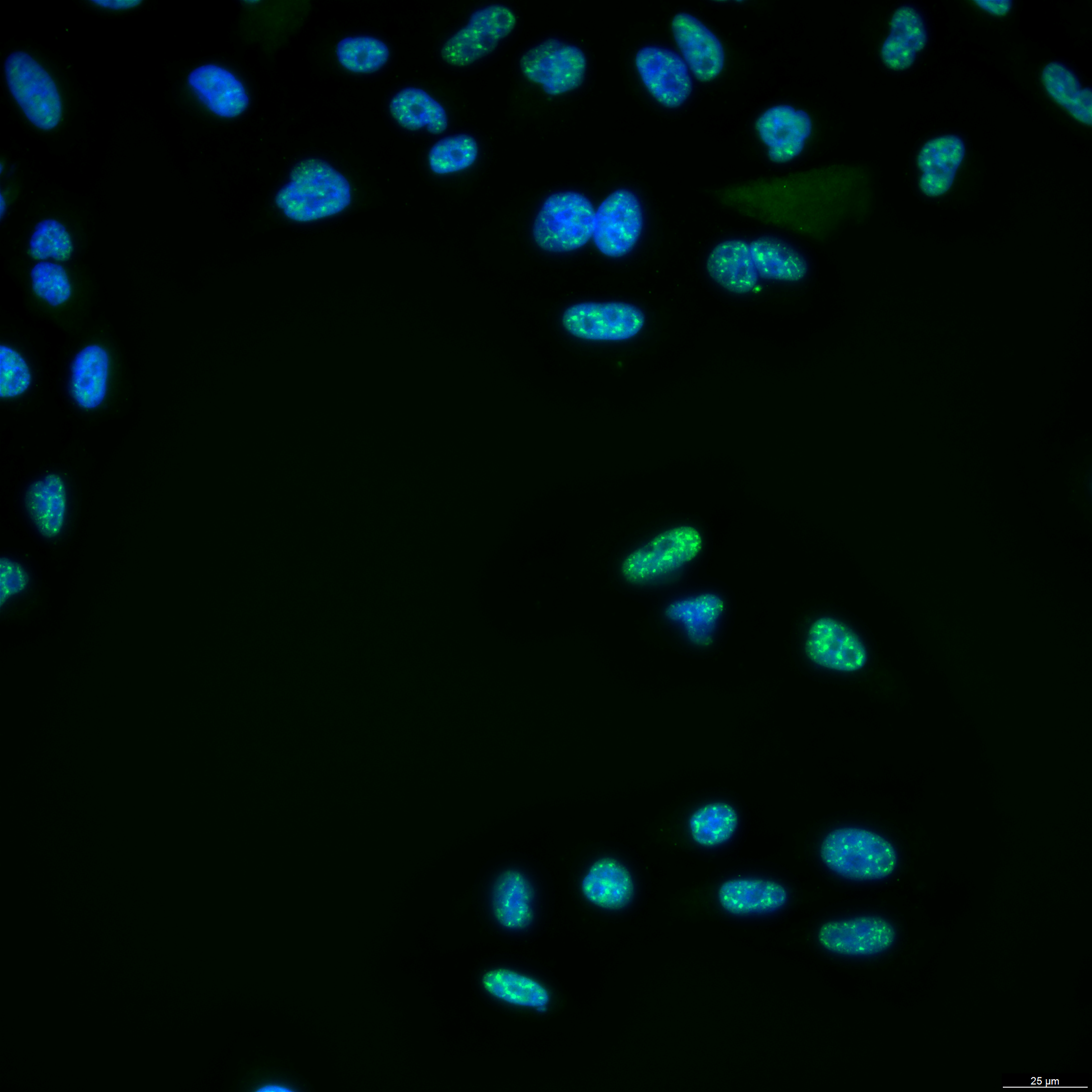

Supplement: Supplementary file 6 — Source data Fig. 2 [file 44318_2025_421_MOESM6_ESM.zip › Figure 2/Figure 2D/IFN γ+ RBN 5 h.tif]

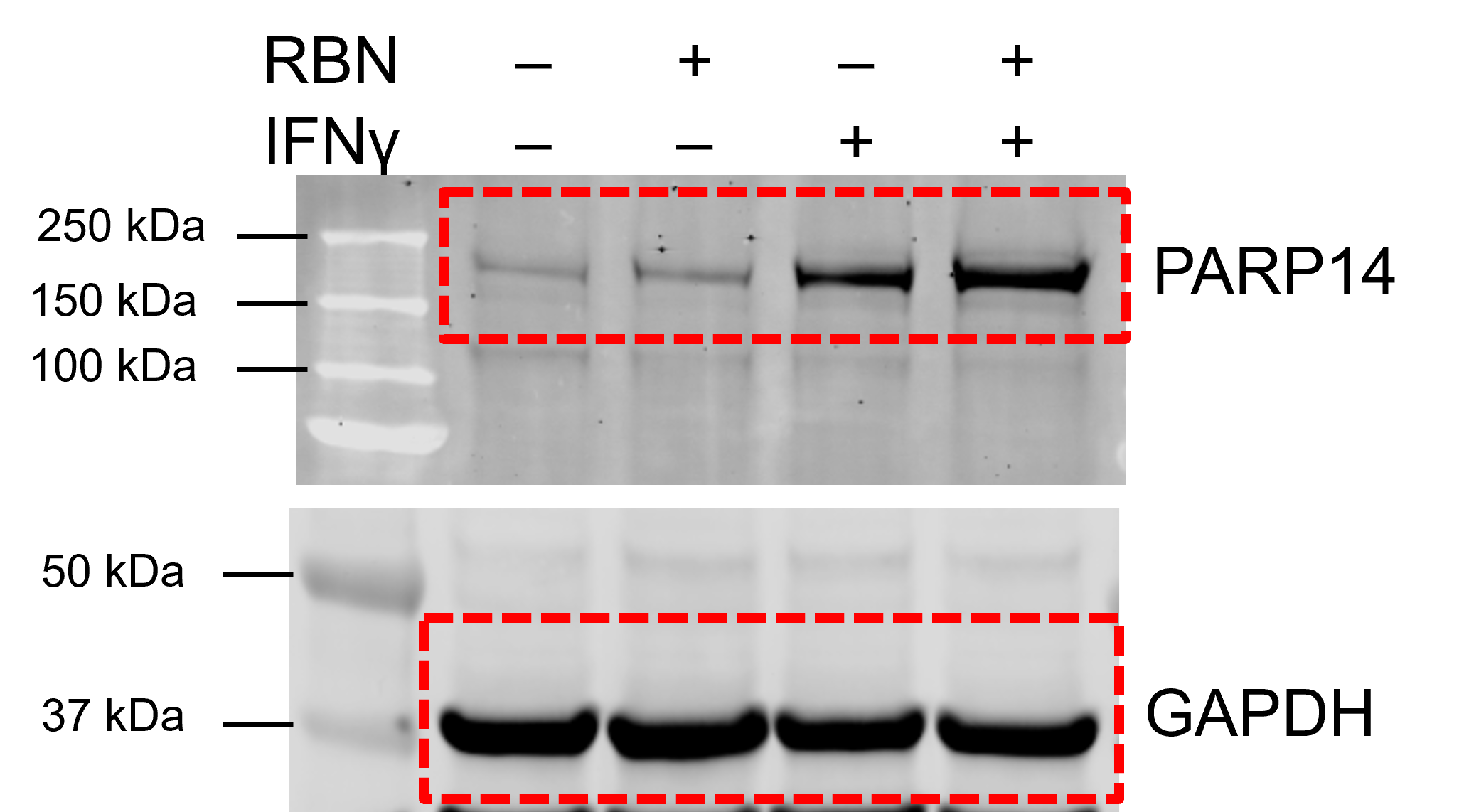

Supplement: Supplementary file 6 — Source data Fig. 2 [file 44318_2025_421_MOESM6_ESM.zip › Figure 2/Figure 2E.tif]

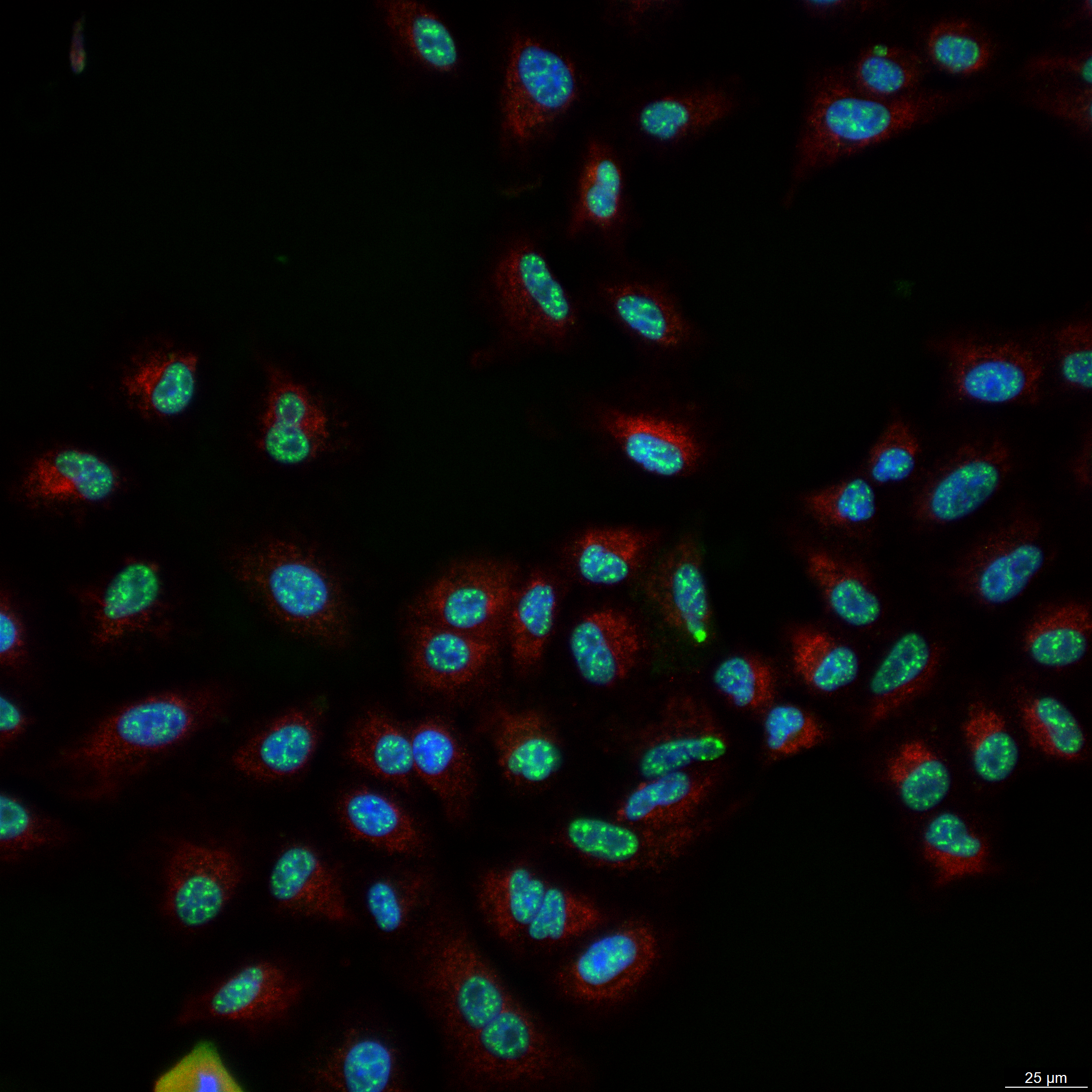

Supplement: Supplementary file 6 — Source data Fig. 2 [file 44318_2025_421_MOESM6_ESM.zip › Figure 2/Figure 2G/Control.tif]

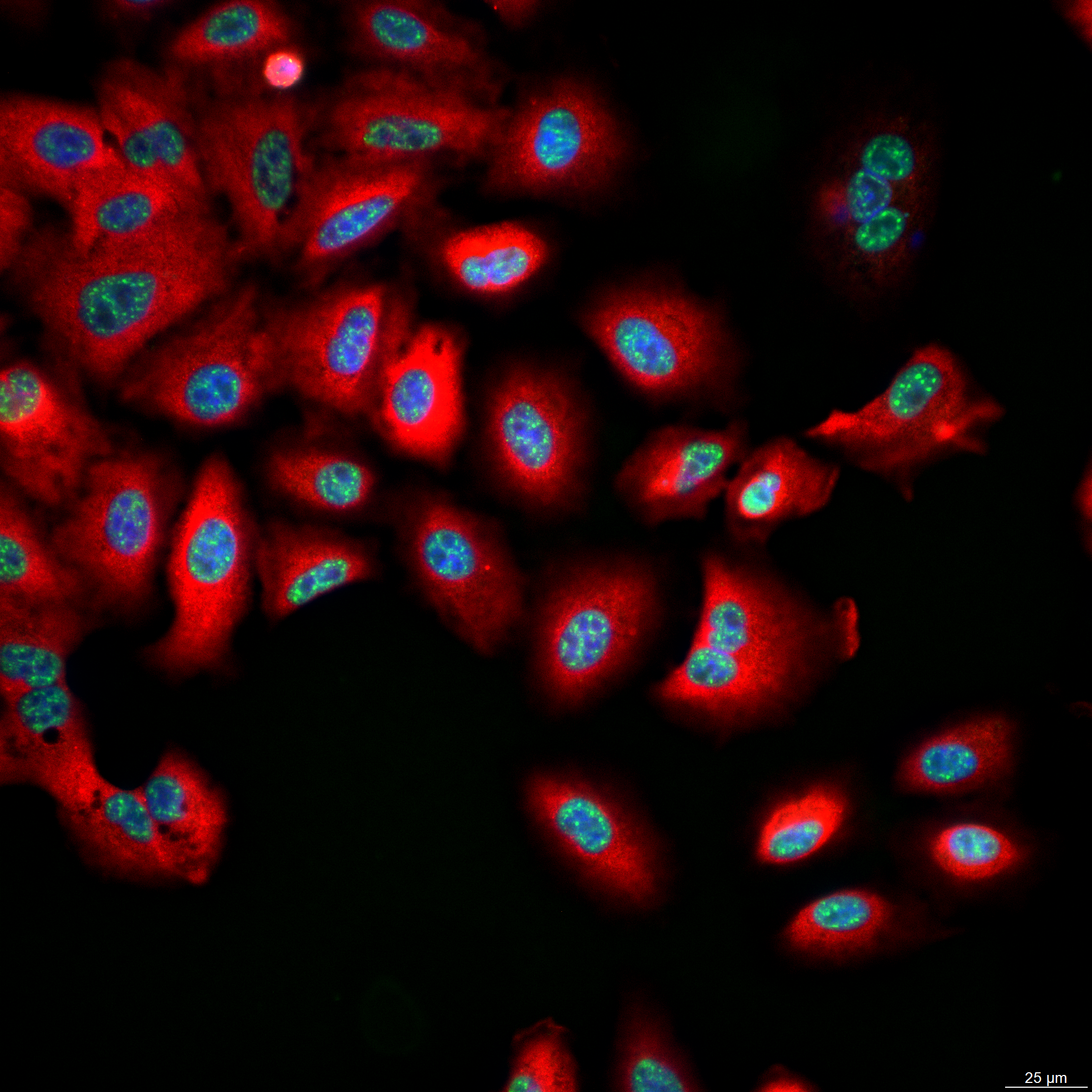

Supplement: Supplementary file 6 — Source data Fig. 2 [file 44318_2025_421_MOESM6_ESM.zip › Figure 2/Figure 2G/IFNγ+RBN.tif]

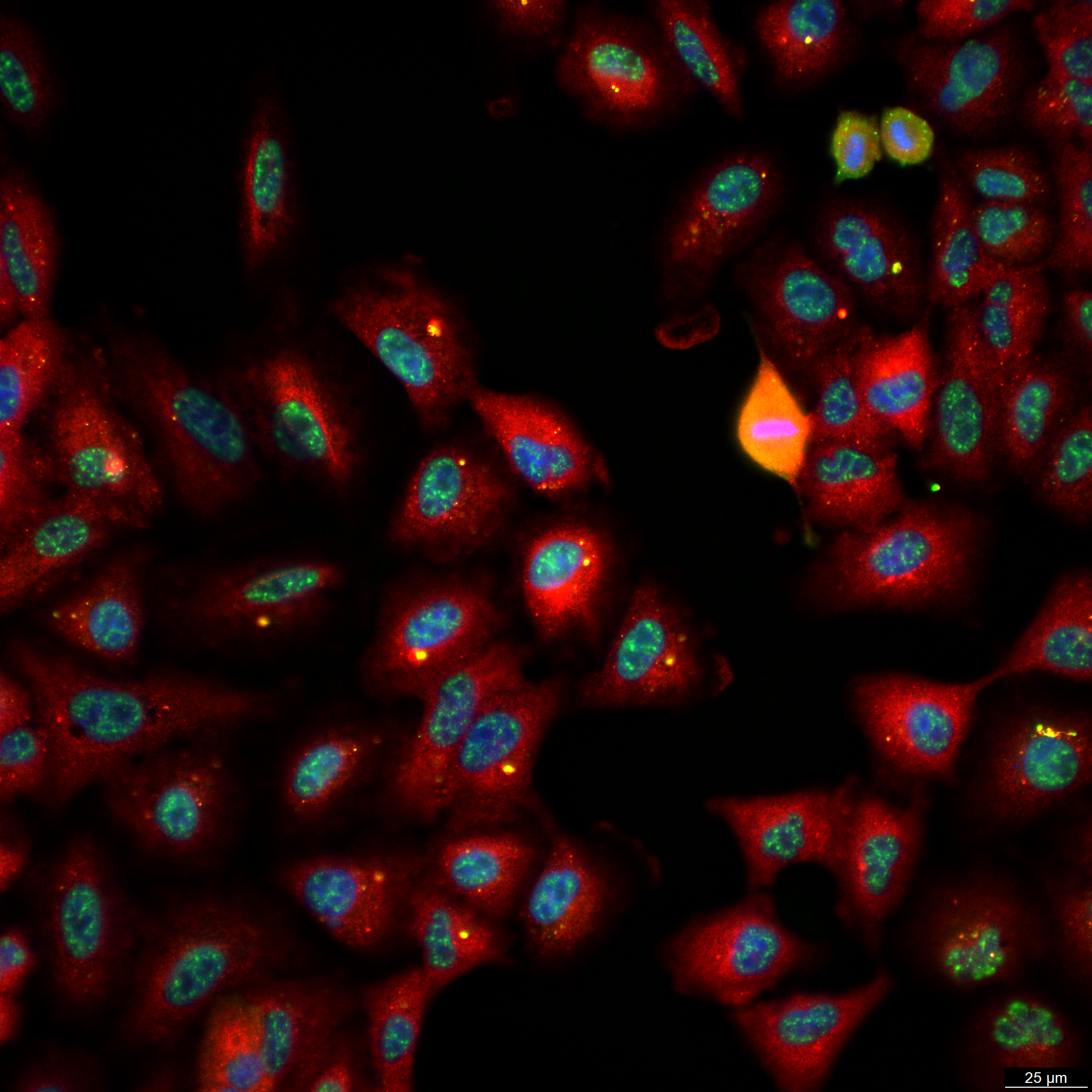

Supplement: Supplementary file 6 — Source data Fig. 2 [file 44318_2025_421_MOESM6_ESM.zip › Figure 2/Figure 2G/IFNγ.tif]

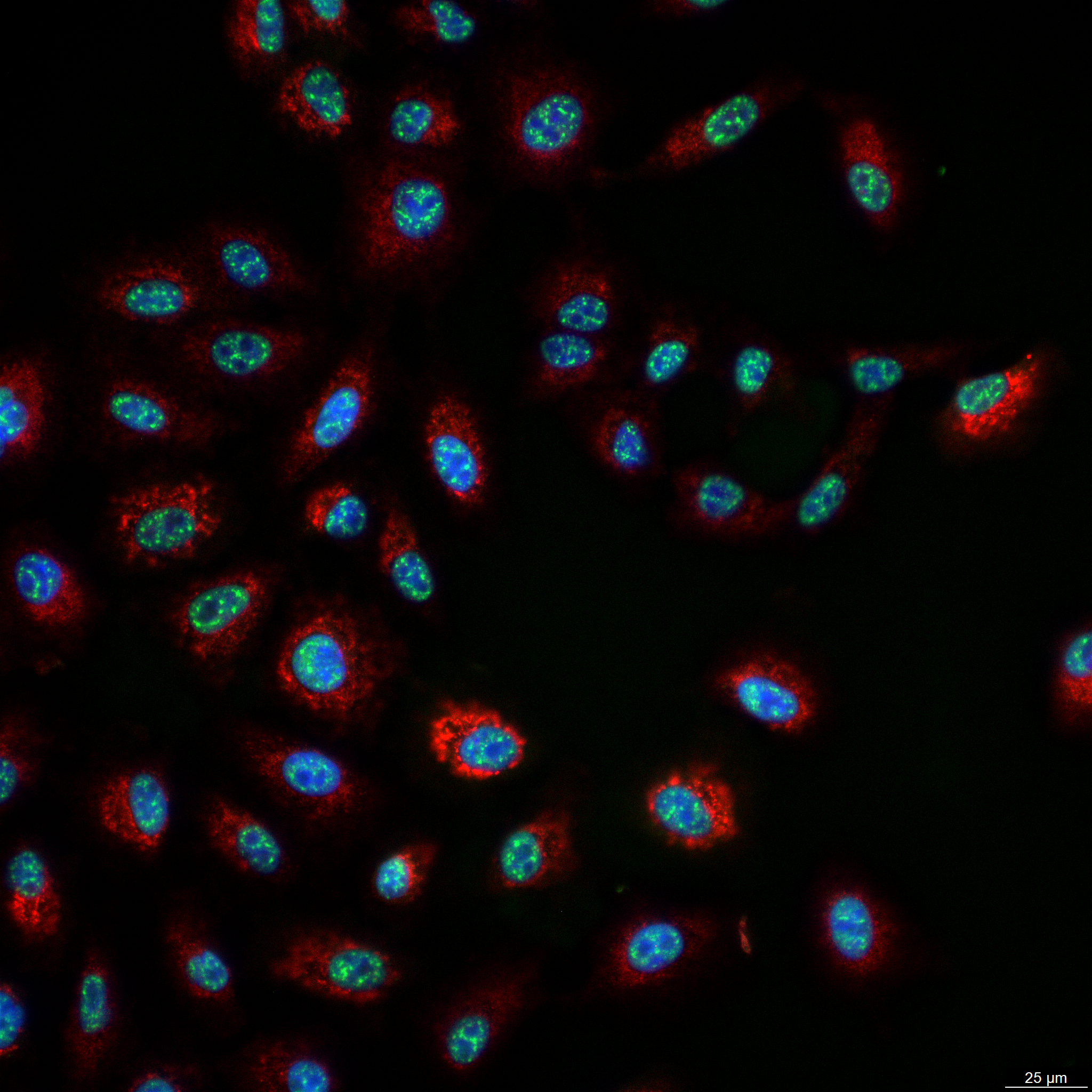

Supplement: Supplementary file 6 — Source data Fig. 2 [file 44318_2025_421_MOESM6_ESM.zip › Figure 2/Figure 2G/RBN.tif]

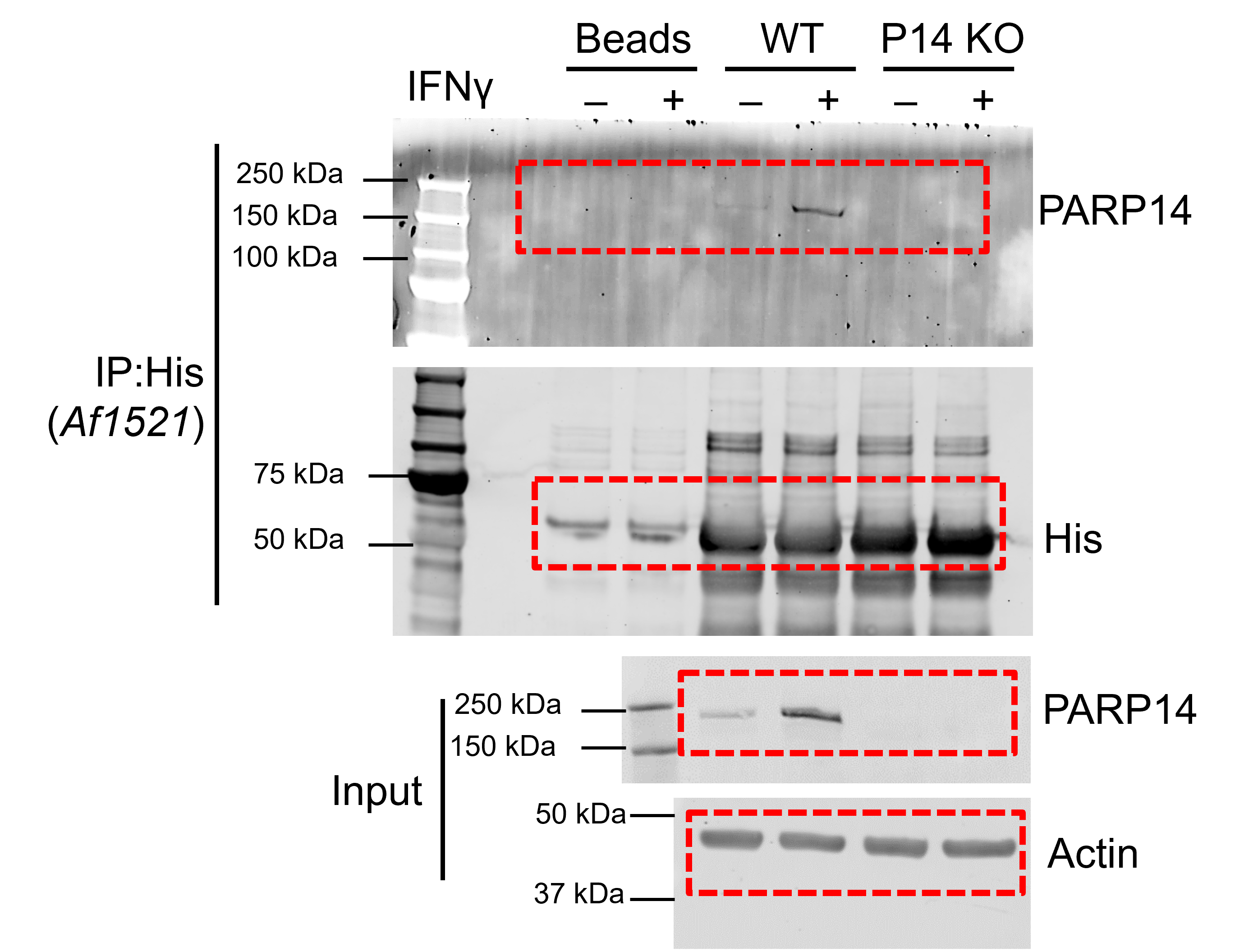

Supplement: Supplementary file 6 — Source data Fig. 2 [file 44318_2025_421_MOESM6_ESM.zip › Figure 2/Figure 2H.tif]

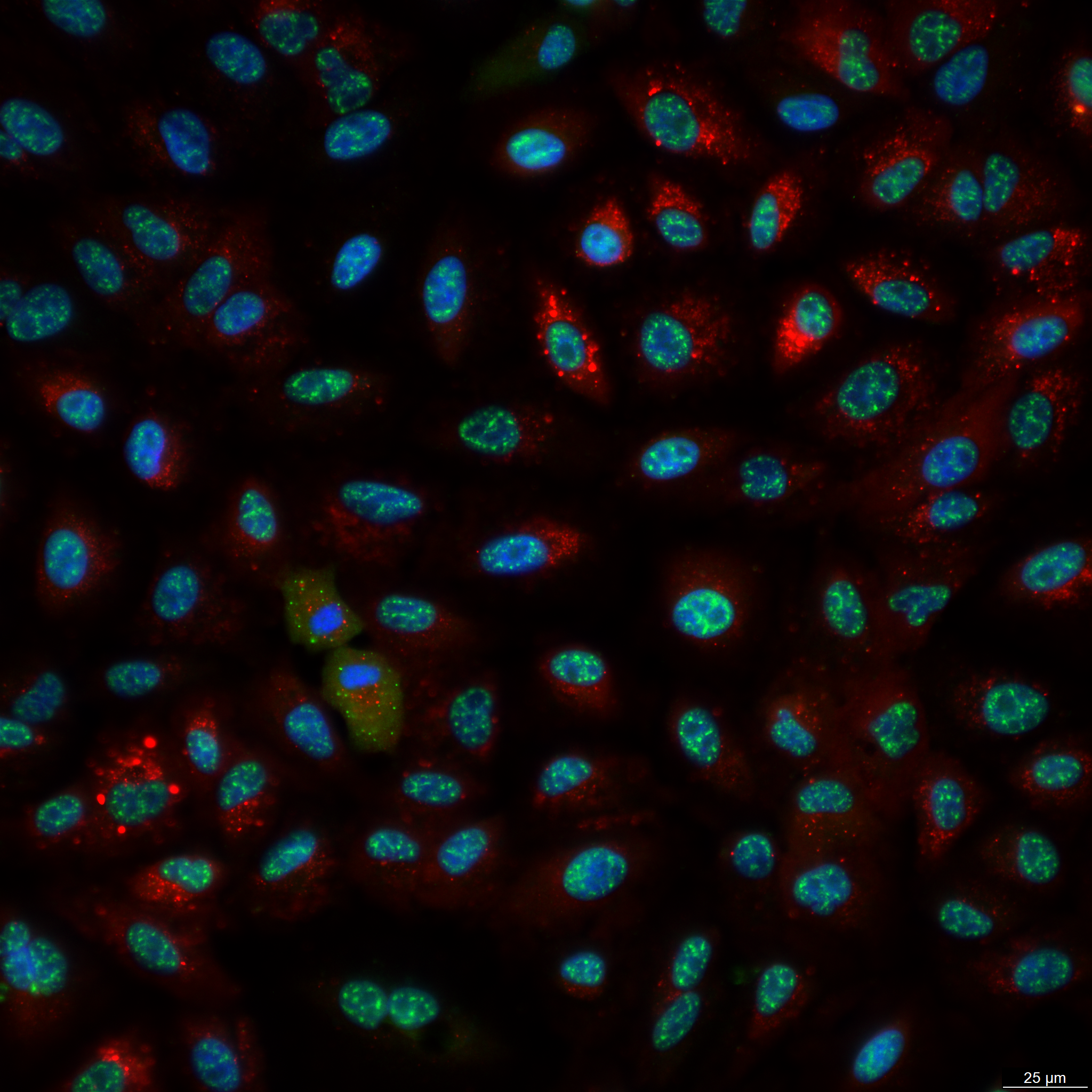

Supplement: Supplementary file 7 — Source data Fig. 3 [file 44318_2025_421_MOESM7_ESM.zip › Figure 3/Figure 3A/Control.tif]

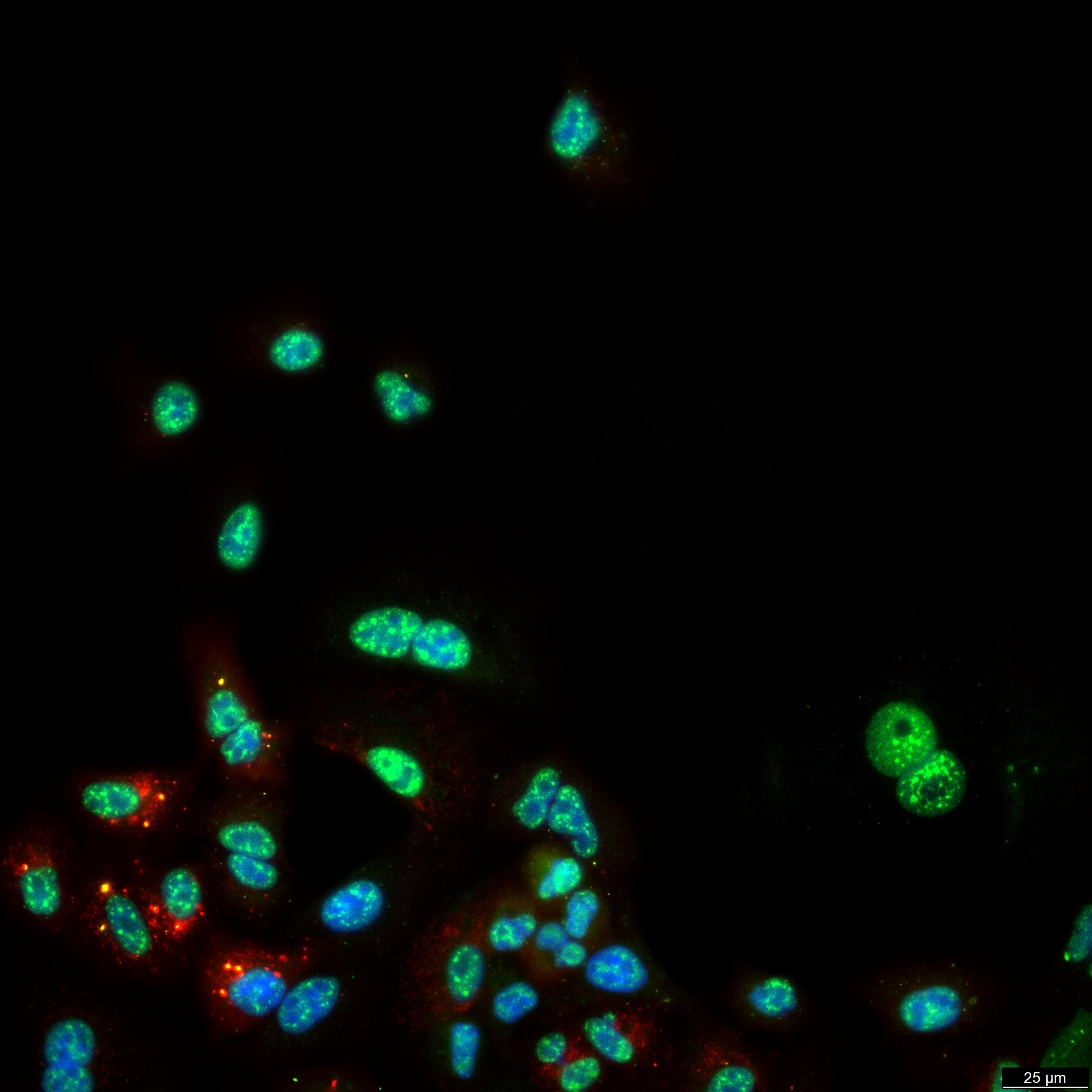

Supplement: Supplementary file 7 — Source data Fig. 3 [file 44318_2025_421_MOESM7_ESM.zip › Figure 3/Figure 3A/lFNγ.tif]

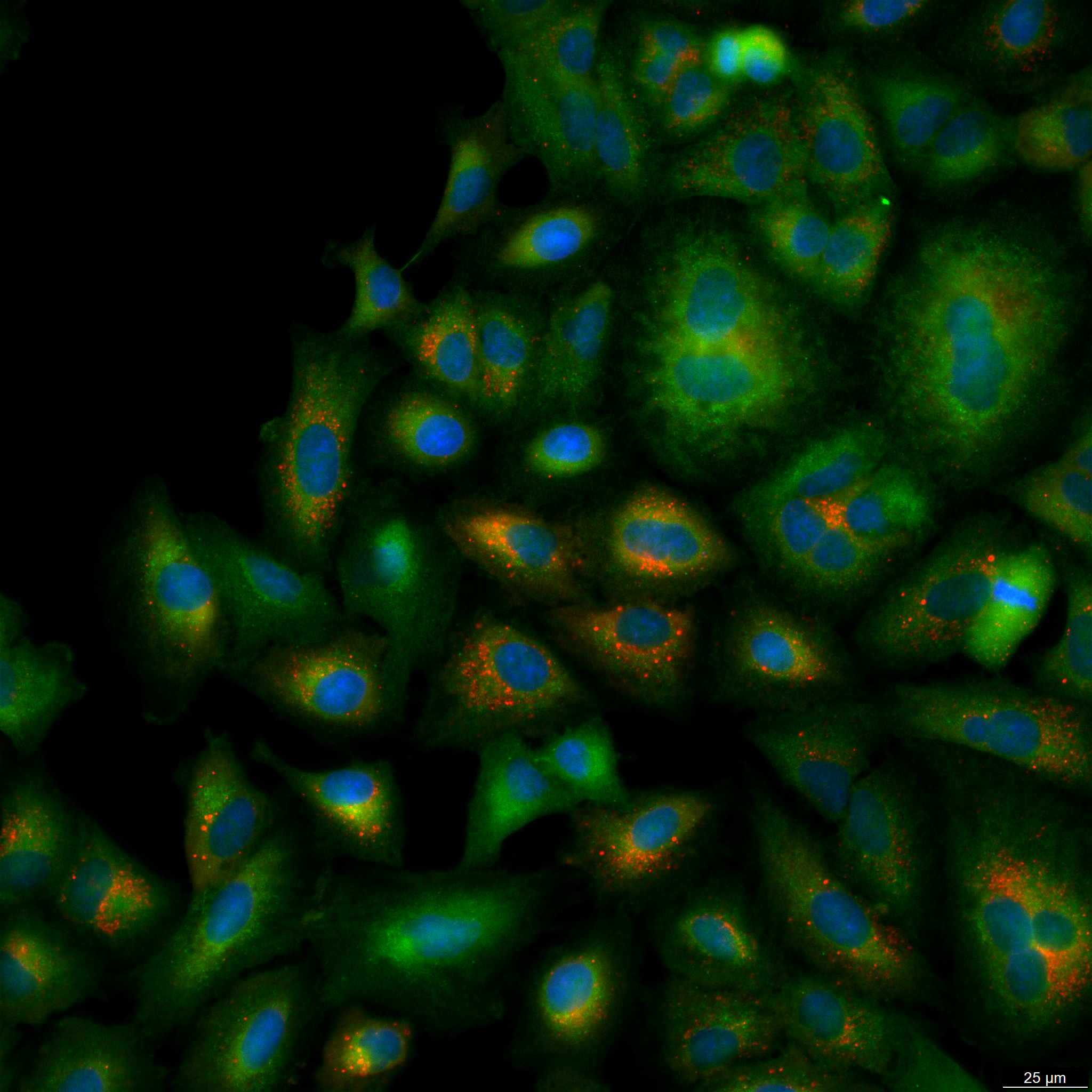

Supplement: Supplementary file 7 — Source data Fig. 3 [file 44318_2025_421_MOESM7_ESM.zip › Figure 3/Figure 3B/Control.tif]

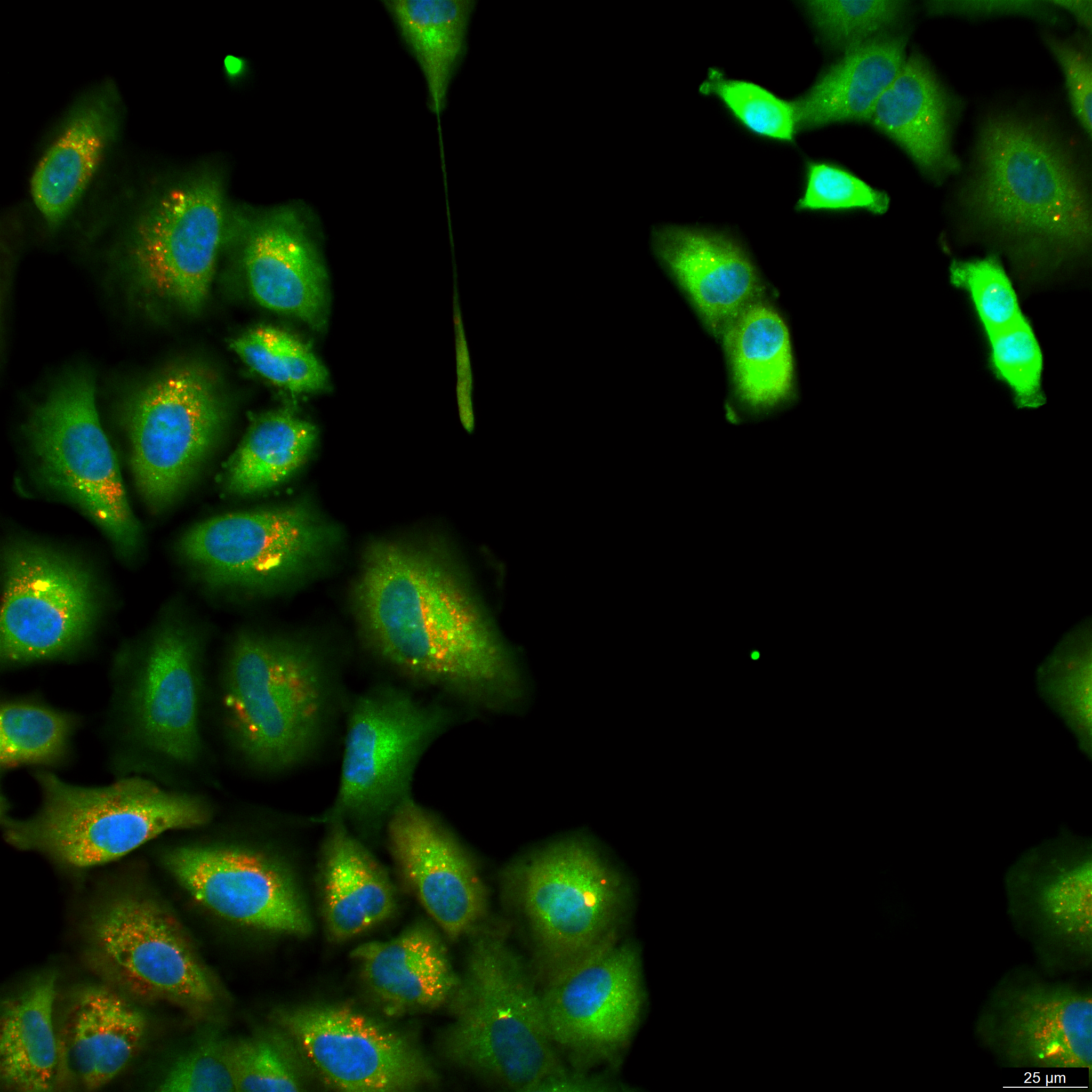

Supplement: Supplementary file 7 — Source data Fig. 3 [file 44318_2025_421_MOESM7_ESM.zip › Figure 3/Figure 3B/lFNγ.tif]

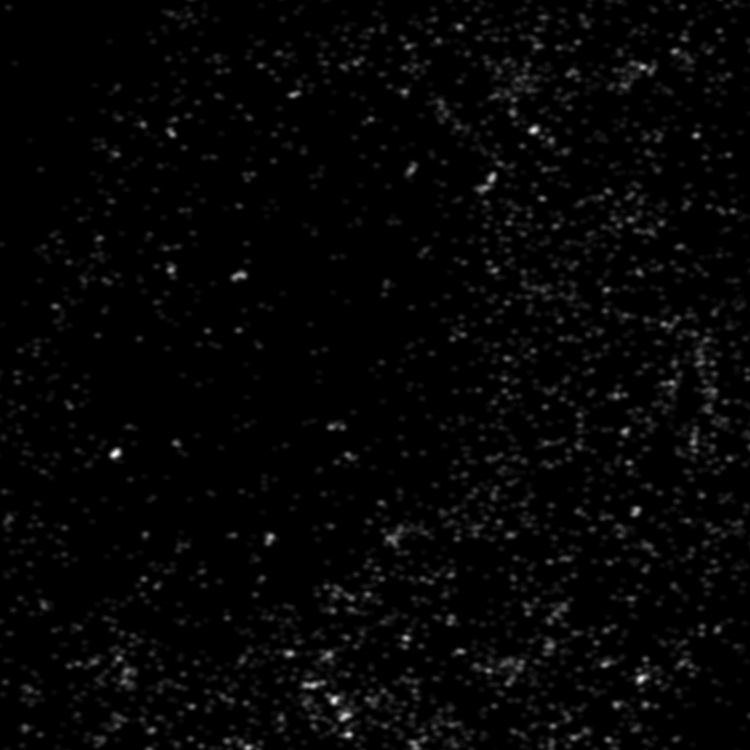

Supplement: Supplementary file 7 — Source data Fig. 3 [file 44318_2025_421_MOESM7_ESM.zip › Figure 3/Figure 3C/CONTROL.tif]

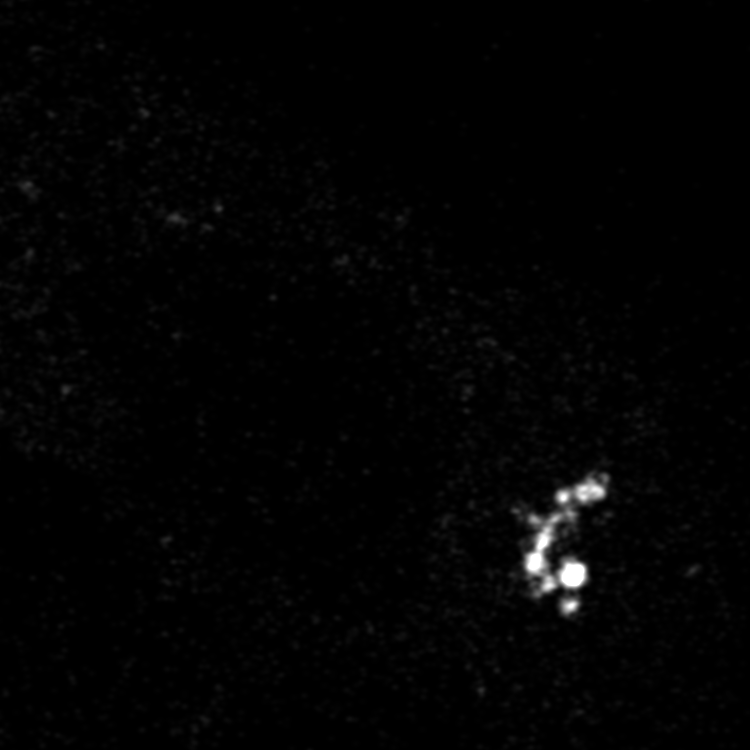

Supplement: Supplementary file 7 — Source data Fig. 3 [file 44318_2025_421_MOESM7_ESM.zip › Figure 3/Figure 3C/IFN.tif]

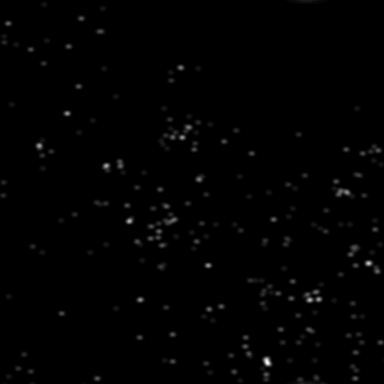

Supplement: Supplementary file 7 — Source data Fig. 3 [file 44318_2025_421_MOESM7_ESM.zip › Figure 3/Figure 3F/IFN.tif]

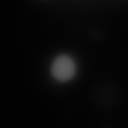

Supplement: Supplementary file 7 — Source data Fig. 3 [file 44318_2025_421_MOESM7_ESM.zip › Figure 3/Figure 3H/p62_IFN_1.tif]

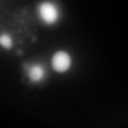

Supplement: Supplementary file 7 — Source data Fig. 3 [file 44318_2025_421_MOESM7_ESM.zip › Figure 3/Figure 3H/p62_IFN_2.tif]

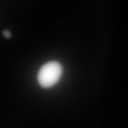

Supplement: Supplementary file 7 — Source data Fig. 3 [file 44318_2025_421_MOESM7_ESM.zip › Figure 3/Figure 3H/p62_IFN_3.tif]

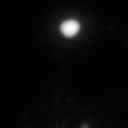

Supplement: Supplementary file 7 — Source data Fig. 3 [file 44318_2025_421_MOESM7_ESM.zip › Figure 3/Figure 3H/p62_IFN_RBN_1.tif]

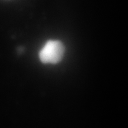

Supplement: Supplementary file 7 — Source data Fig. 3 [file 44318_2025_421_MOESM7_ESM.zip › Figure 3/Figure 3H/p62_IFN_RBN_2.tif]

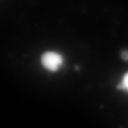

Supplement: Supplementary file 7 — Source data Fig. 3 [file 44318_2025_421_MOESM7_ESM.zip › Figure 3/Figure 3H/p62_IFN_RBN_3.tif]

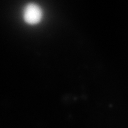

Supplement: Supplementary file 7 — Source data Fig. 3 [file 44318_2025_421_MOESM7_ESM.zip › Figure 3/Figure 3H/p62_IFN_RBN_4.tif]

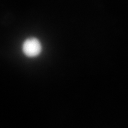

Supplement: Supplementary file 7 — Source data Fig. 3 [file 44318_2025_421_MOESM7_ESM.zip › Figure 3/Figure 3H/p62_IFN_RBN_5.tif]

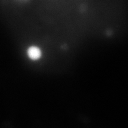

Supplement: Supplementary file 7 — Source data Fig. 3 [file 44318_2025_421_MOESM7_ESM.zip › Figure 3/Figure 3H/p62_IFN_RBN_6.tif]

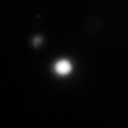

Supplement: Supplementary file 7 — Source data Fig. 3 [file 44318_2025_421_MOESM7_ESM.zip › Figure 3/Figure 3H/p62_no IFN_1.tif]

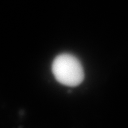

Supplement: Supplementary file 7 — Source data Fig. 3 [file 44318_2025_421_MOESM7_ESM.zip › Figure 3/Figure 3H/p62_no IFN_2.tif]

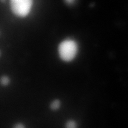

Supplement: Supplementary file 7 — Source data Fig. 3 [file 44318_2025_421_MOESM7_ESM.zip › Figure 3/Figure 3H/p62_no IFN_3.tif]

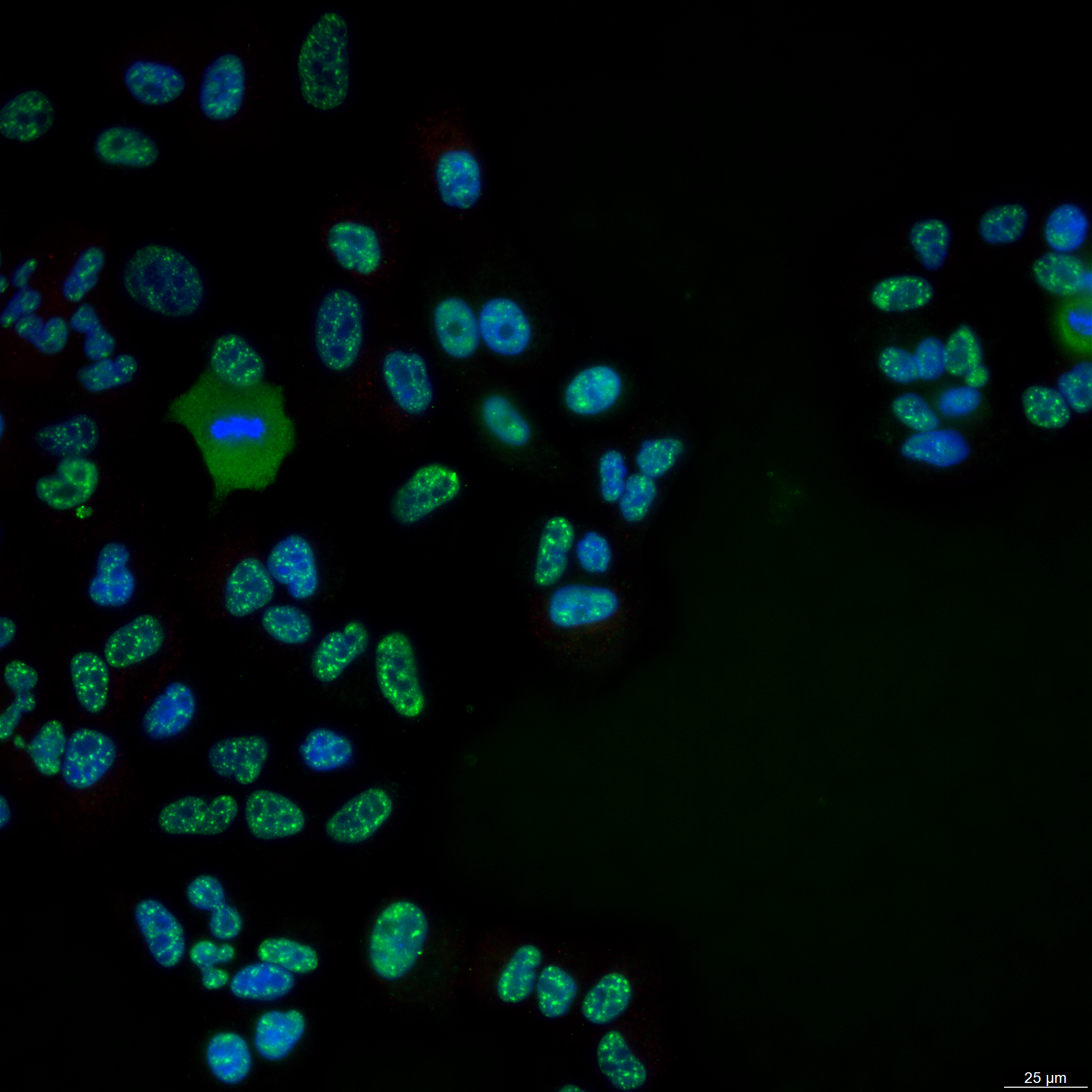

Supplement: Supplementary file 7 — Source data Fig. 3 [file 44318_2025_421_MOESM7_ESM.zip › Figure 3/Figure 3J/p62KD .tif]

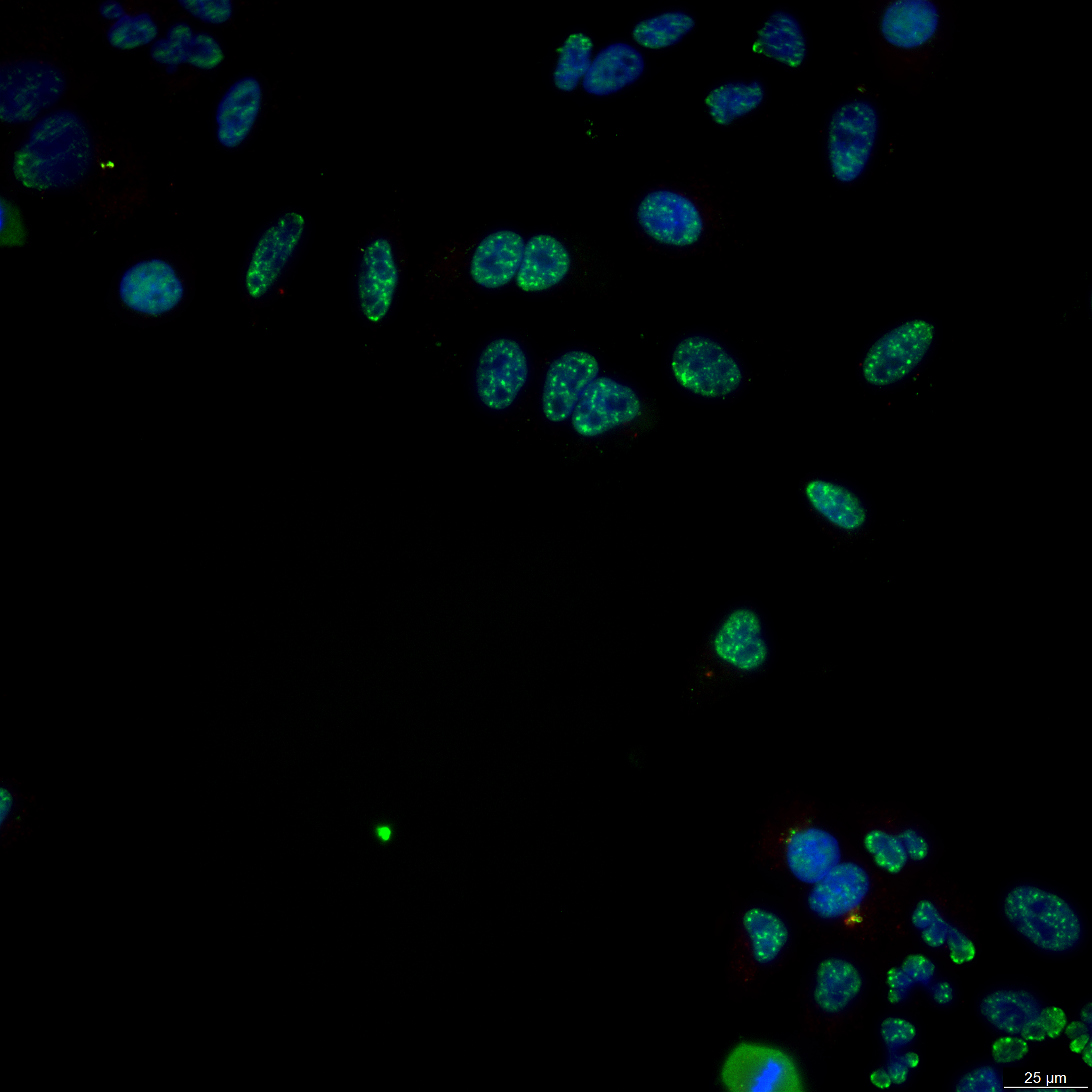

Supplement: Supplementary file 7 — Source data Fig. 3 [file 44318_2025_421_MOESM7_ESM.zip › Figure 3/Figure 3J/p62KD+lFNγ.tif]

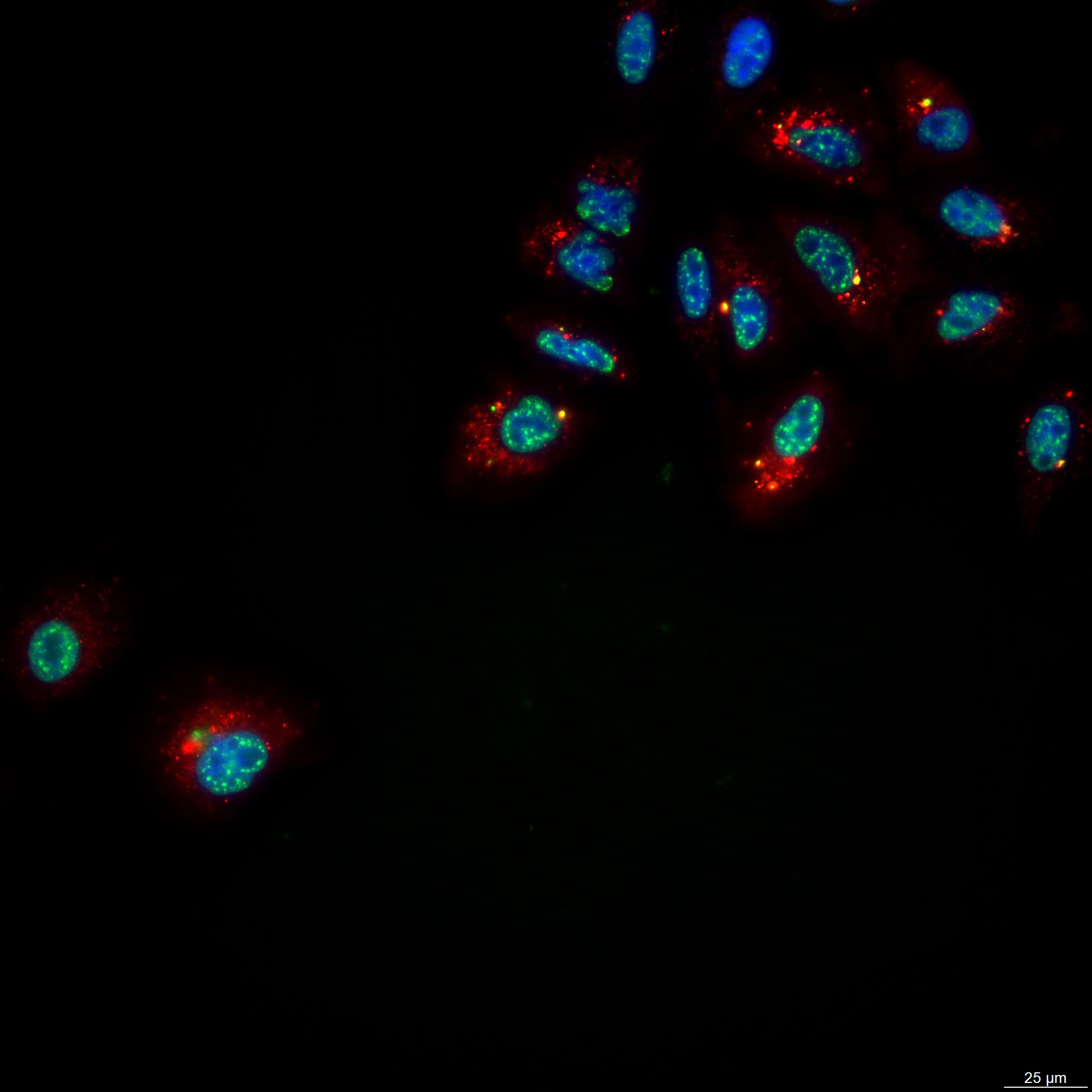

Supplement: Supplementary file 7 — Source data Fig. 3 [file 44318_2025_421_MOESM7_ESM.zip › Figure 3/Figure 3J/WT+lFNγ.tif]

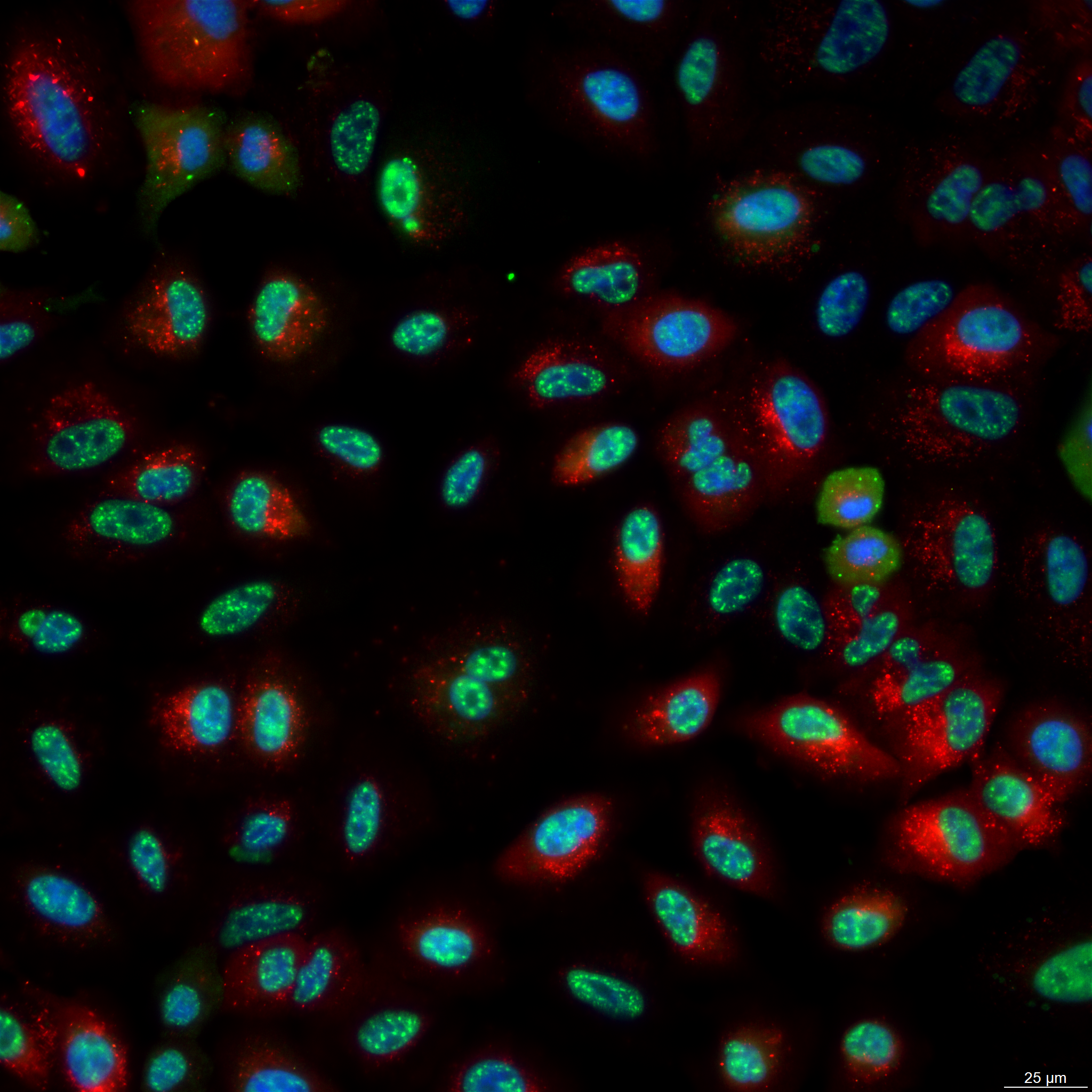

Supplement: Supplementary file 7 — Source data Fig. 3 [file 44318_2025_421_MOESM7_ESM.zip › Figure 3/Figure 3J/WT.tif]

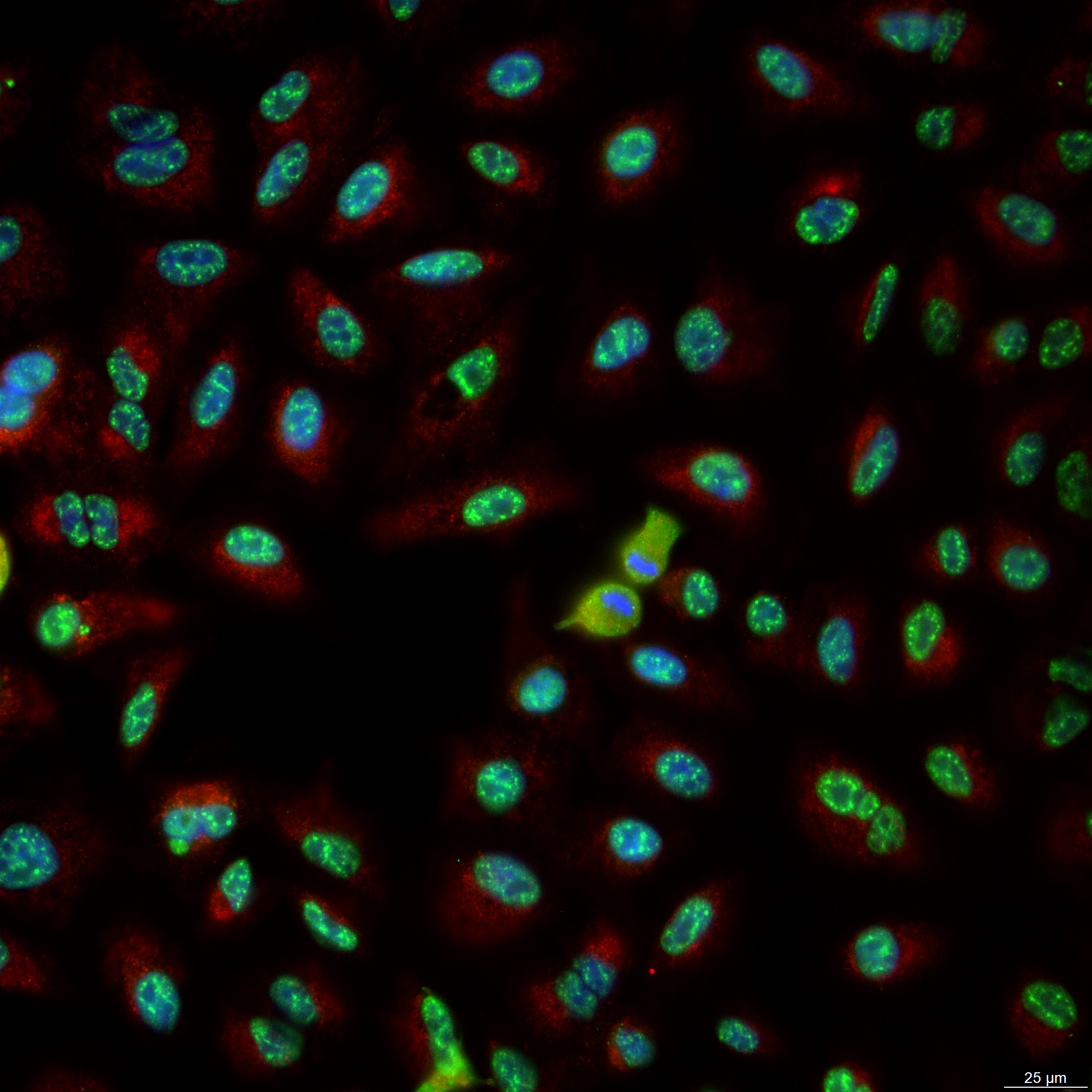

Supplement: Supplementary file 7 — Source data Fig. 3 [file 44318_2025_421_MOESM7_ESM.zip › Figure 3/Figure 3K/p62 KD.tif]

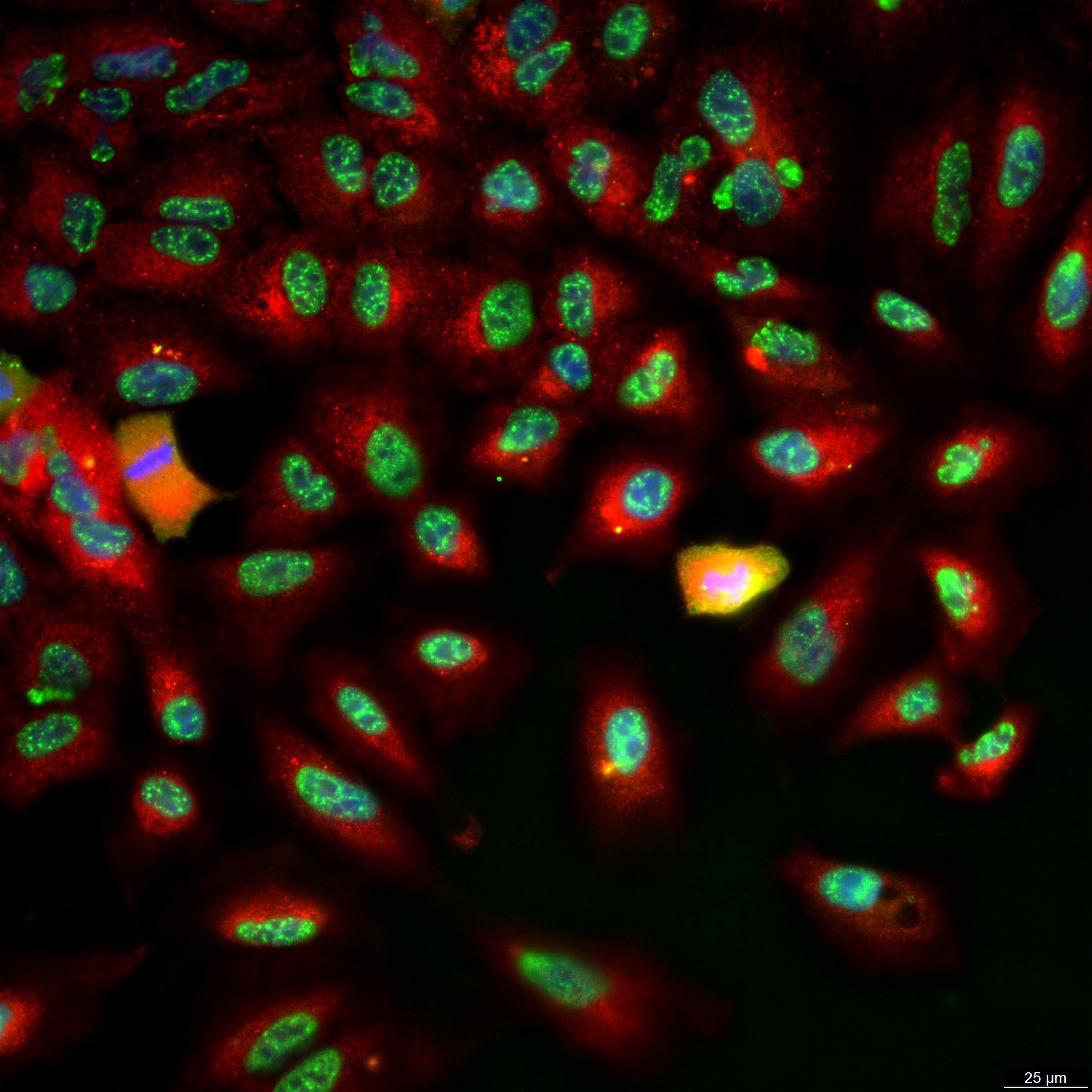

Supplement: Supplementary file 7 — Source data Fig. 3 [file 44318_2025_421_MOESM7_ESM.zip › Figure 3/Figure 3K/p62KD+lFNγ.tif]

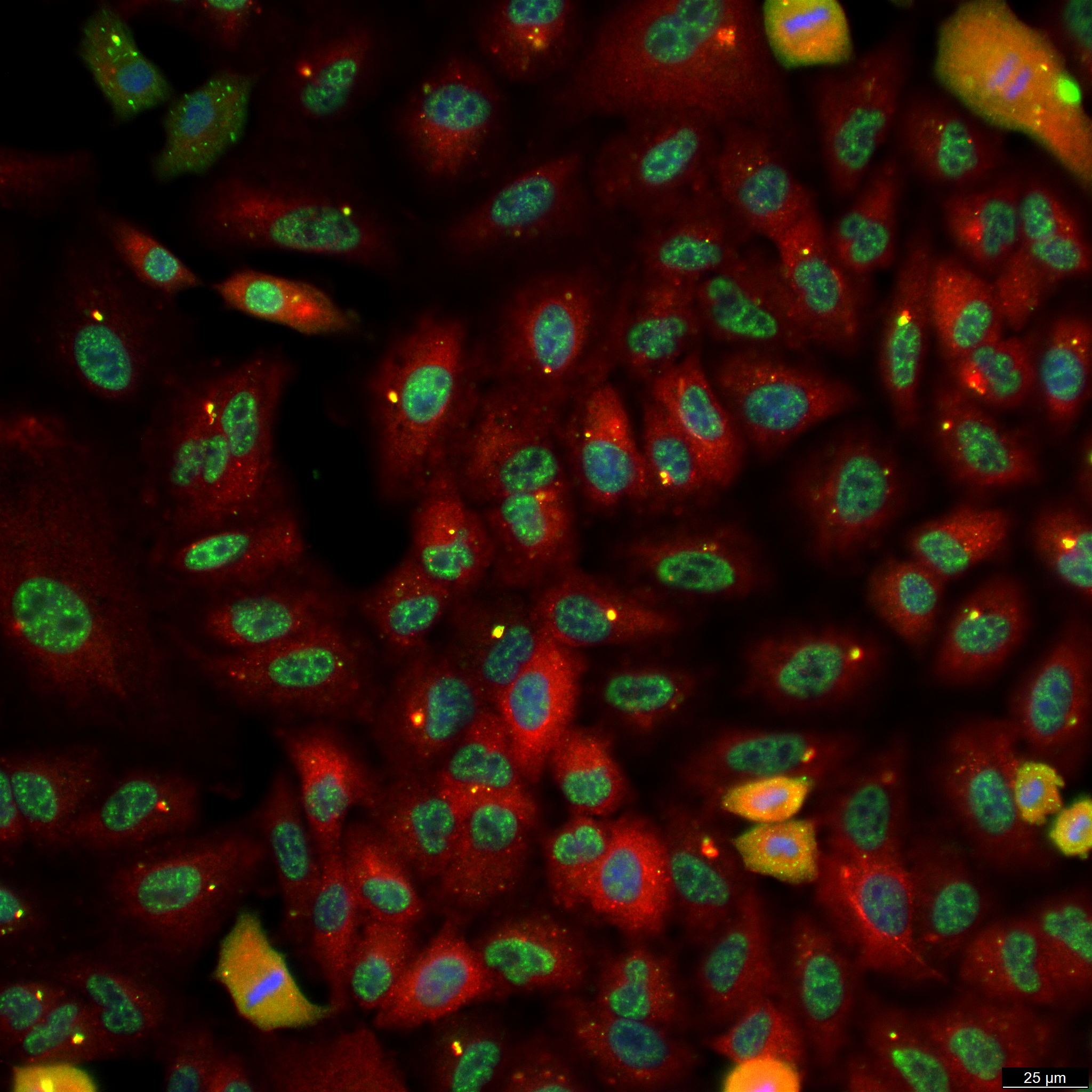

Supplement: Supplementary file 7 — Source data Fig. 3 [file 44318_2025_421_MOESM7_ESM.zip › Figure 3/Figure 3K/WT+lFNγ.tif]

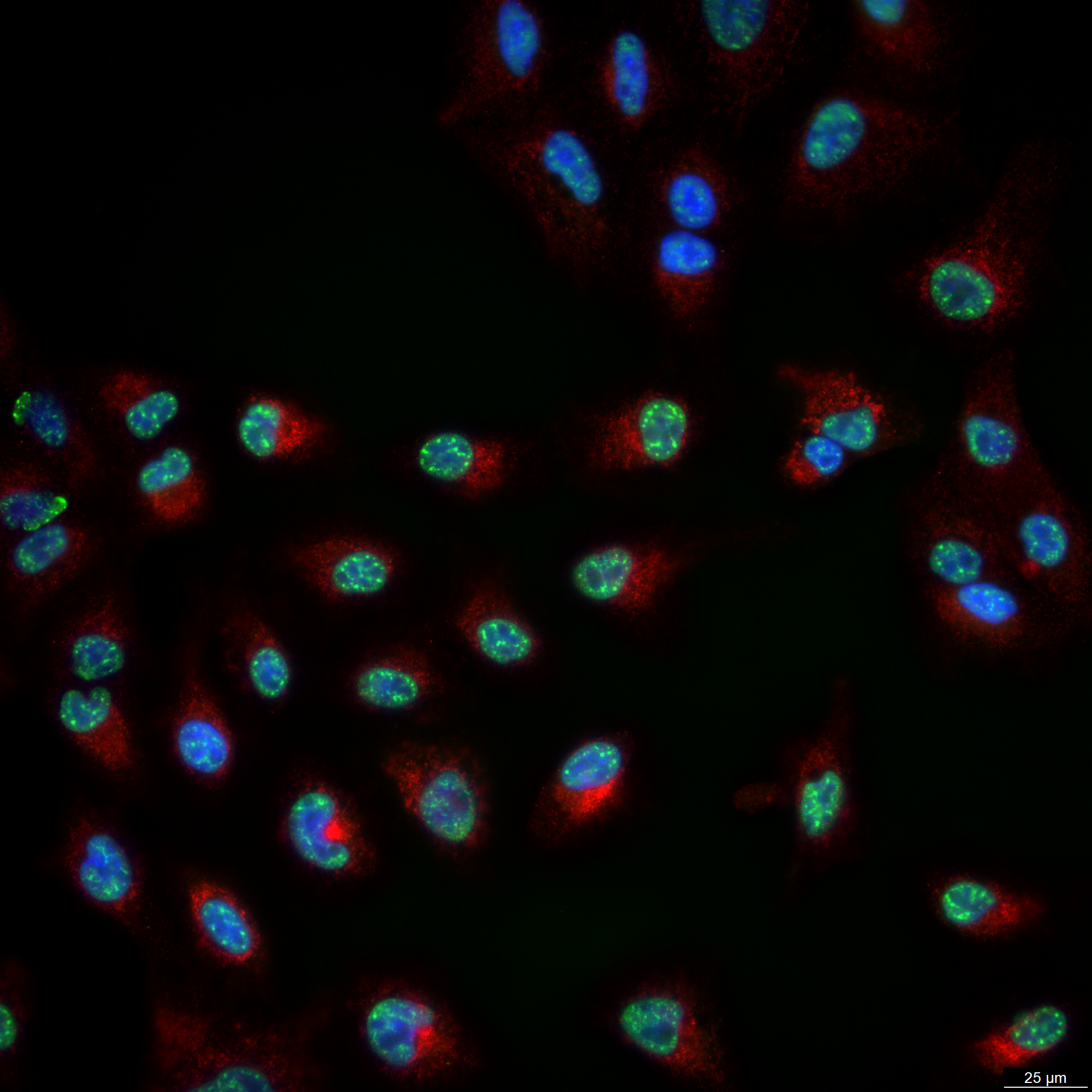

Supplement: Supplementary file 7 — Source data Fig. 3 [file 44318_2025_421_MOESM7_ESM.zip › Figure 3/Figure 3K/WT.tif]

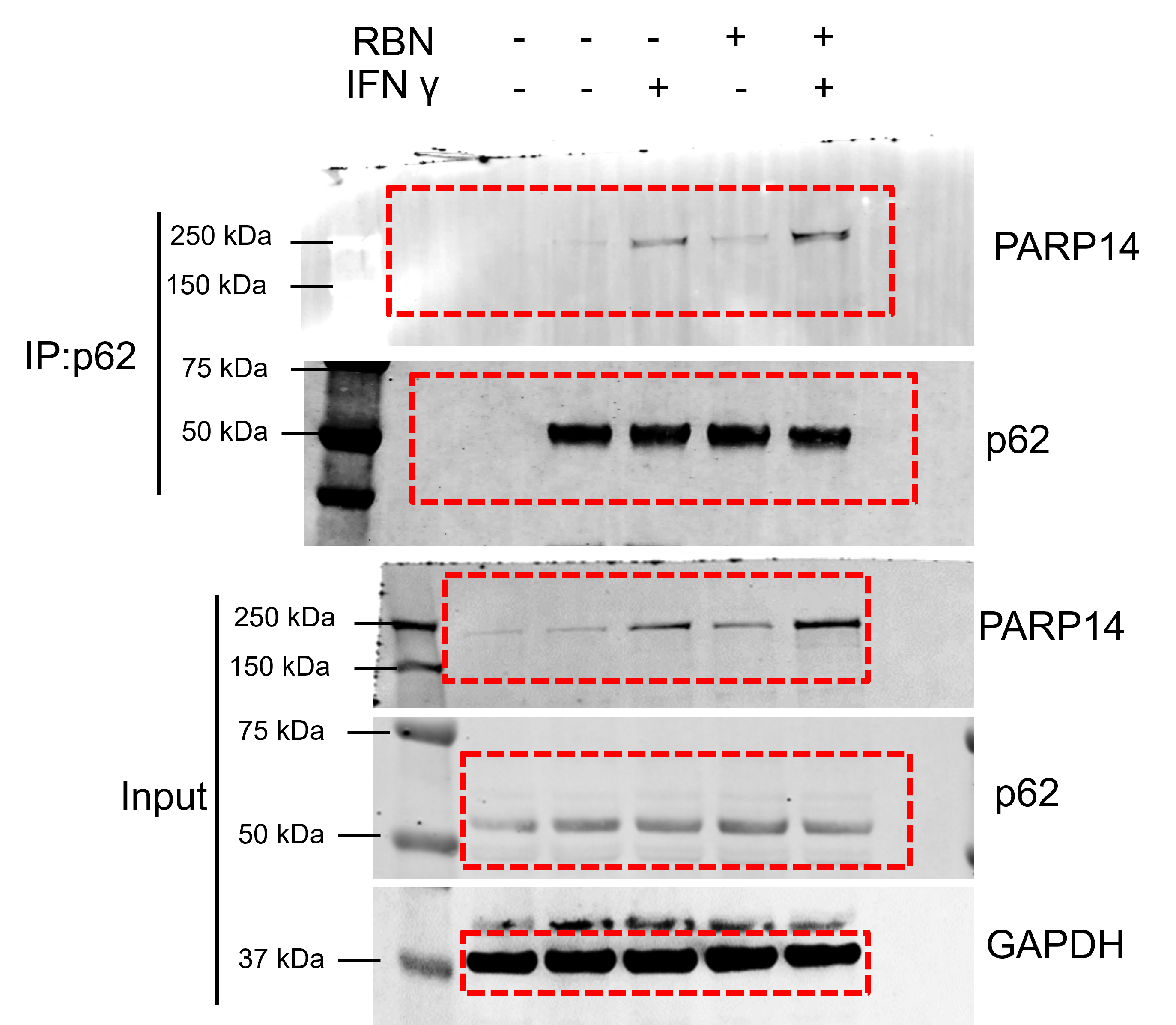

Supplement: Supplementary file 8 — Source data Fig. 4 [file 44318_2025_421_MOESM8_ESM.zip › Figure 4/Figure 4A.tif]

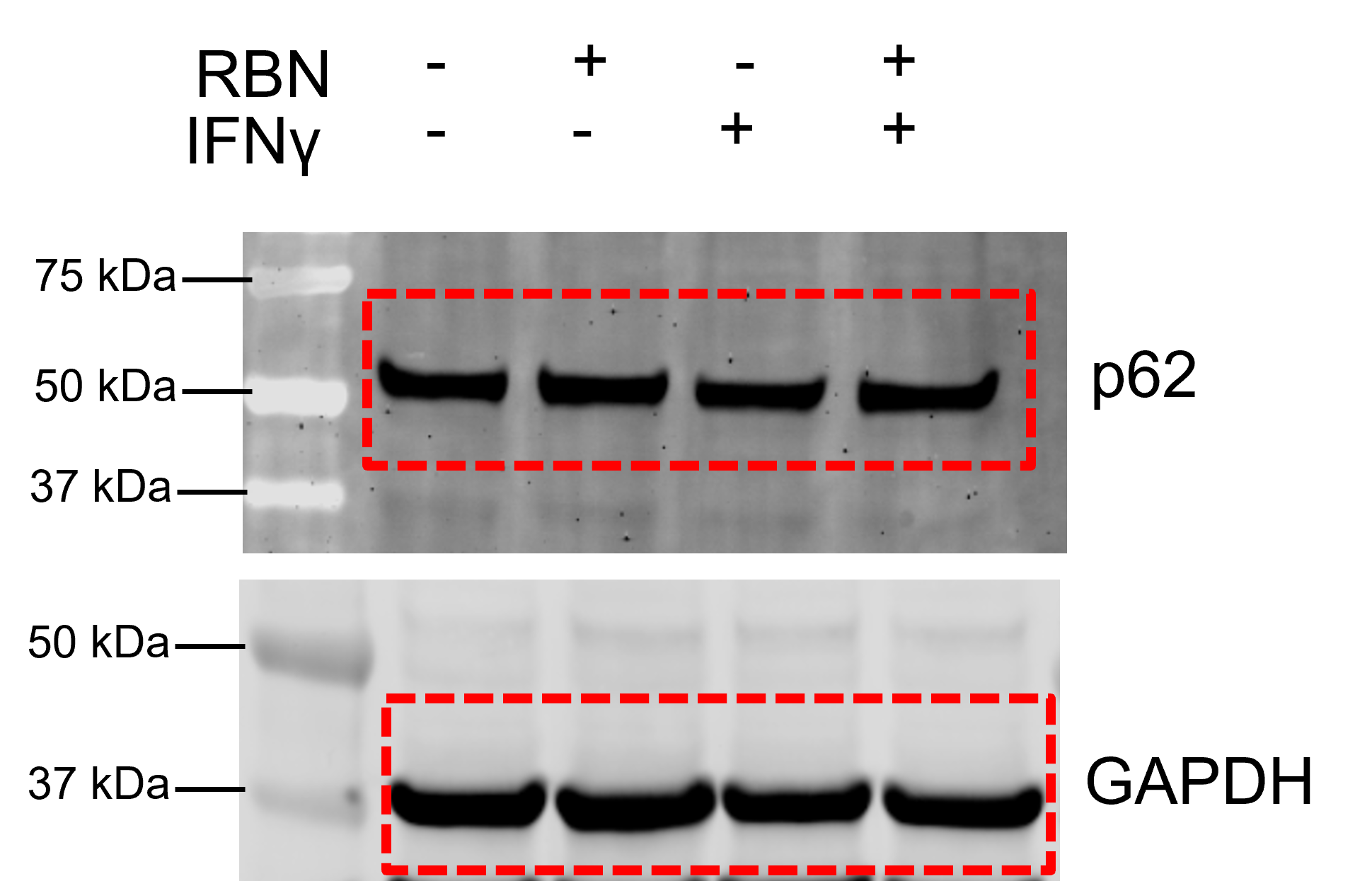

Supplement: Supplementary file 8 — Source data Fig. 4 [file 44318_2025_421_MOESM8_ESM.zip › Figure 4/Figure 4C Top Panel.tif]

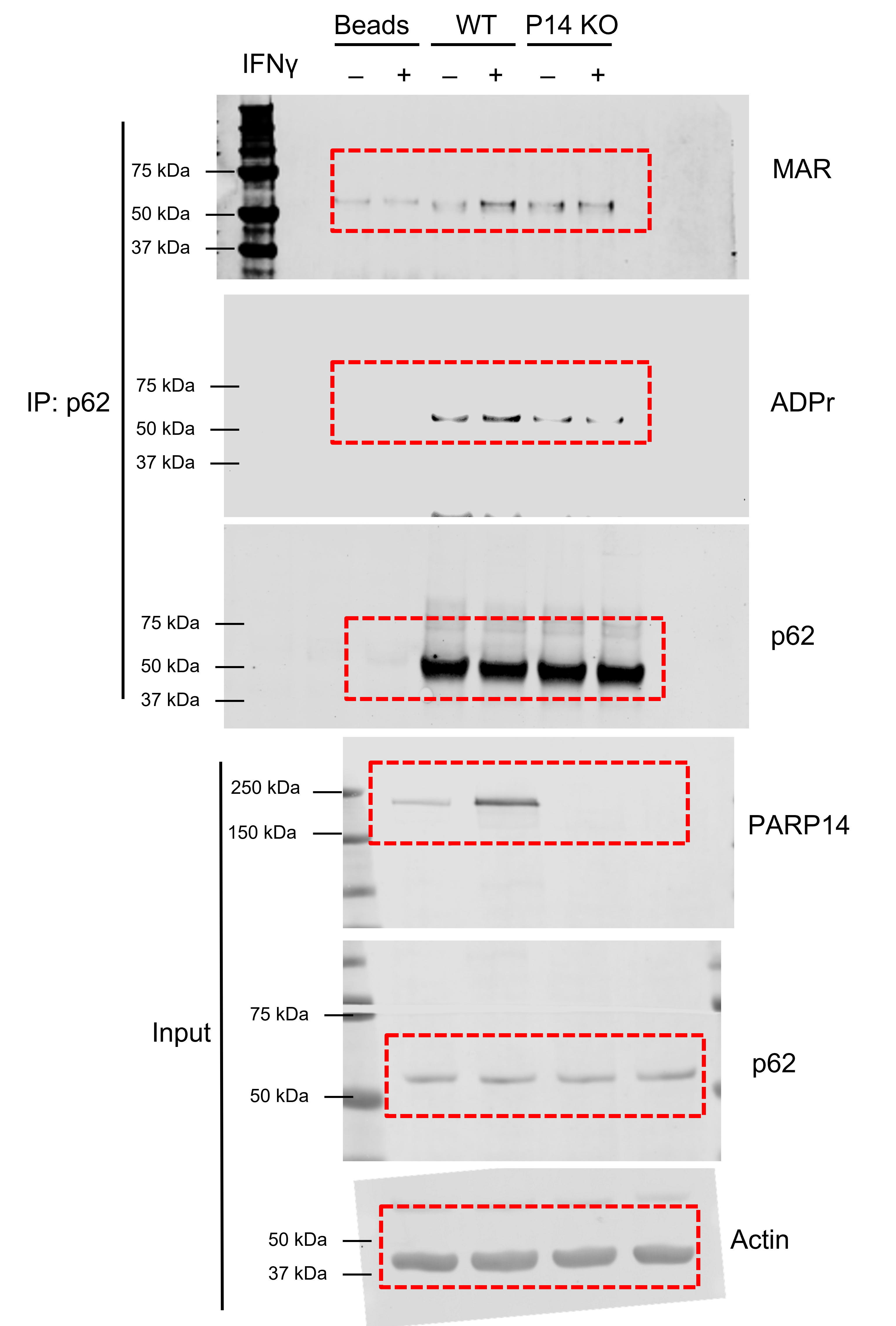

Supplement: Supplementary file 8 — Source data Fig. 4 [file 44318_2025_421_MOESM8_ESM.zip › Figure 4/Figure 4D.tif]

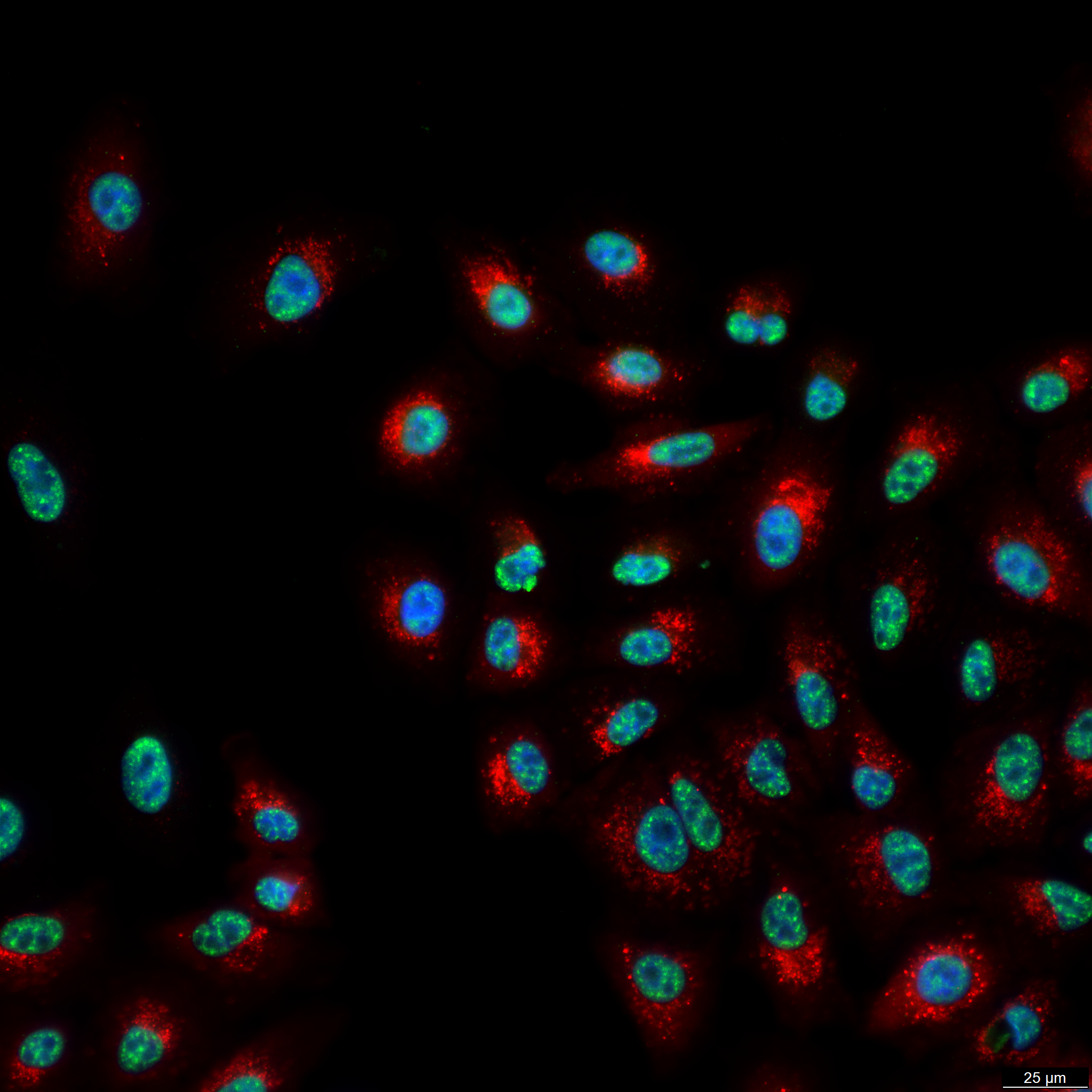

Supplement: Supplementary file 8 — Source data Fig. 4 [file 44318_2025_421_MOESM8_ESM.zip › Figure 4/Figure 4E/P14KO+lFNγ.tif]

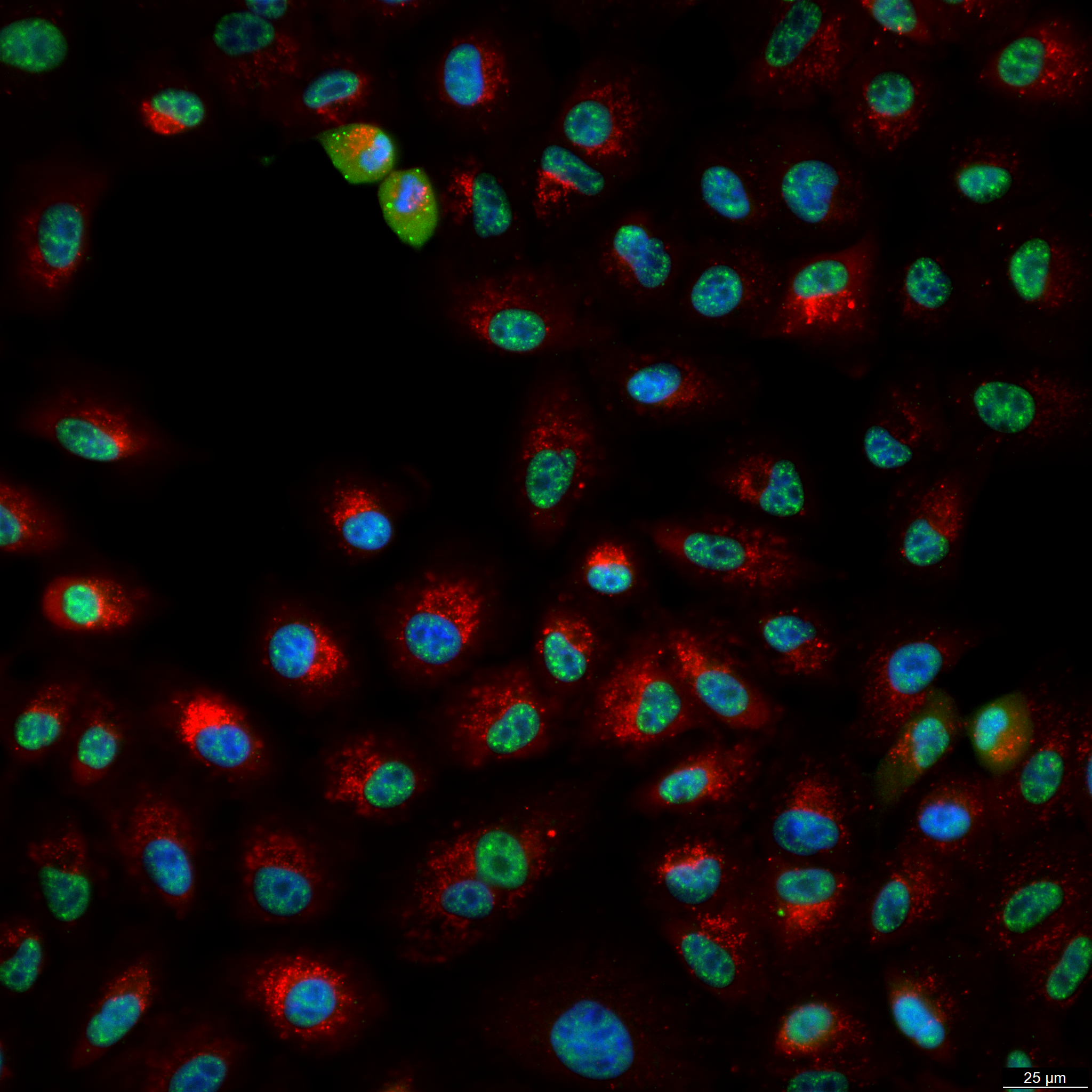

Supplement: Supplementary file 8 — Source data Fig. 4 [file 44318_2025_421_MOESM8_ESM.zip › Figure 4/Figure 4E/P14KO.tif]

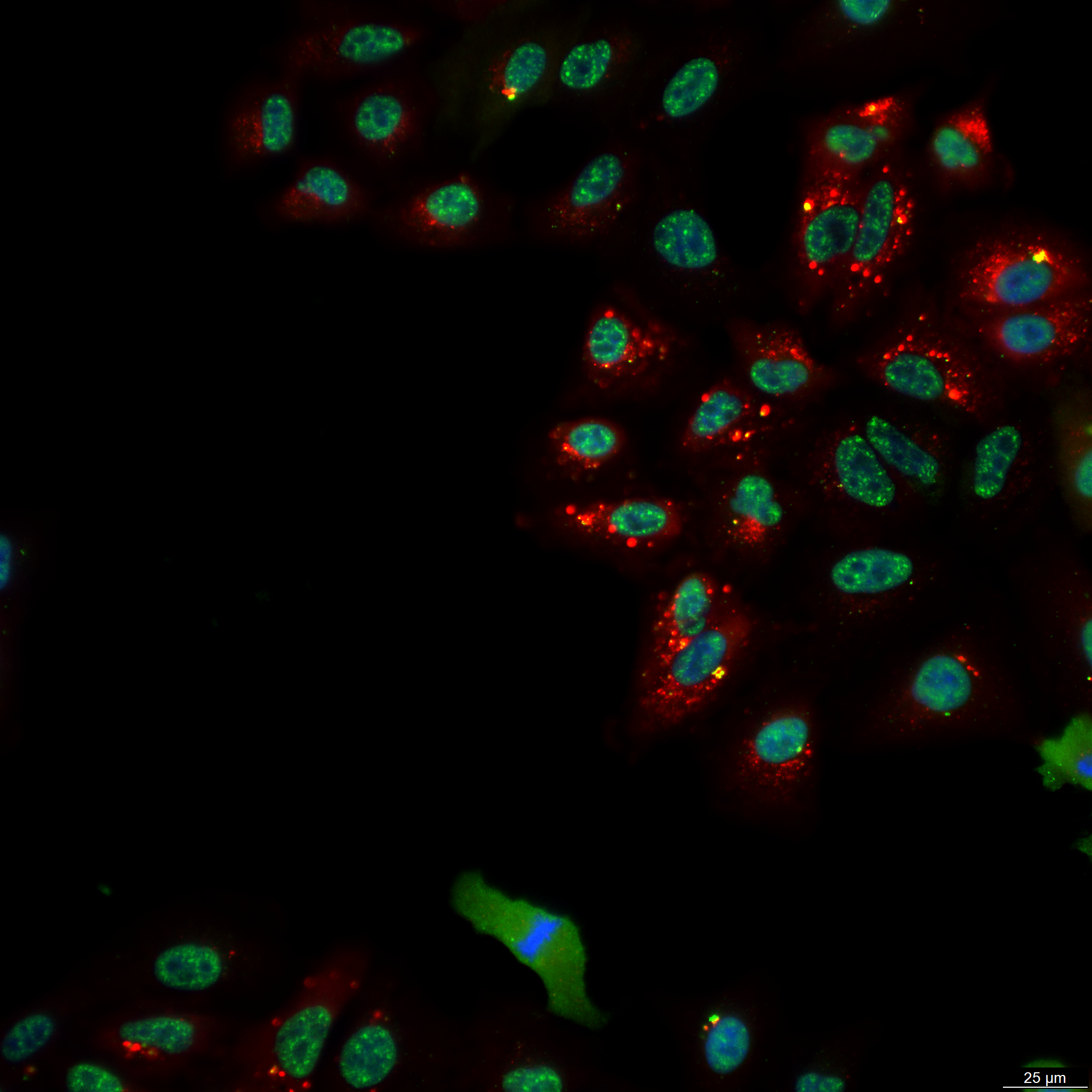

Supplement: Supplementary file 8 — Source data Fig. 4 [file 44318_2025_421_MOESM8_ESM.zip › Figure 4/Figure 4E/WT+lFNγ.tif]

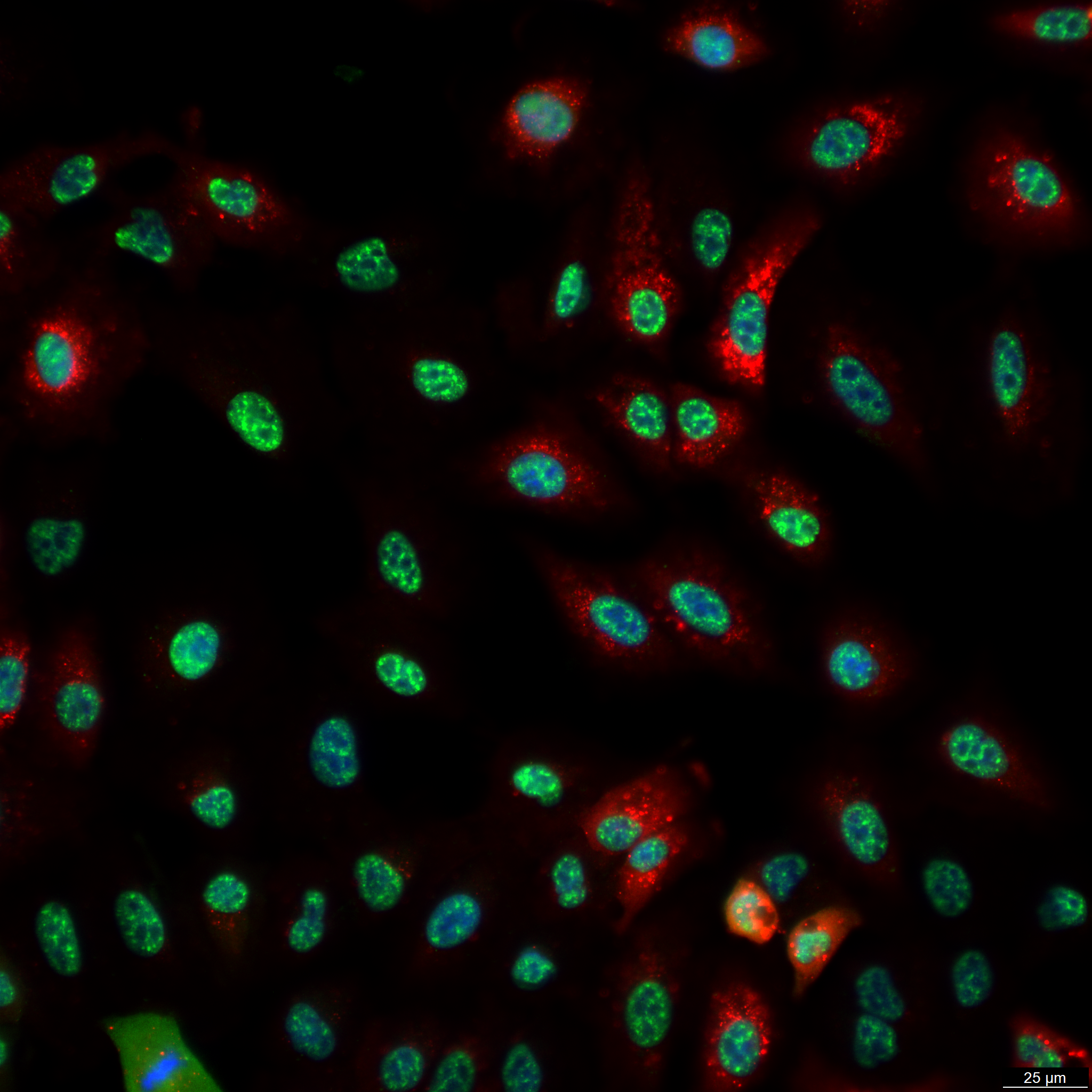

Supplement: Supplementary file 8 — Source data Fig. 4 [file 44318_2025_421_MOESM8_ESM.zip › Figure 4/Figure 4E/WT.tif]

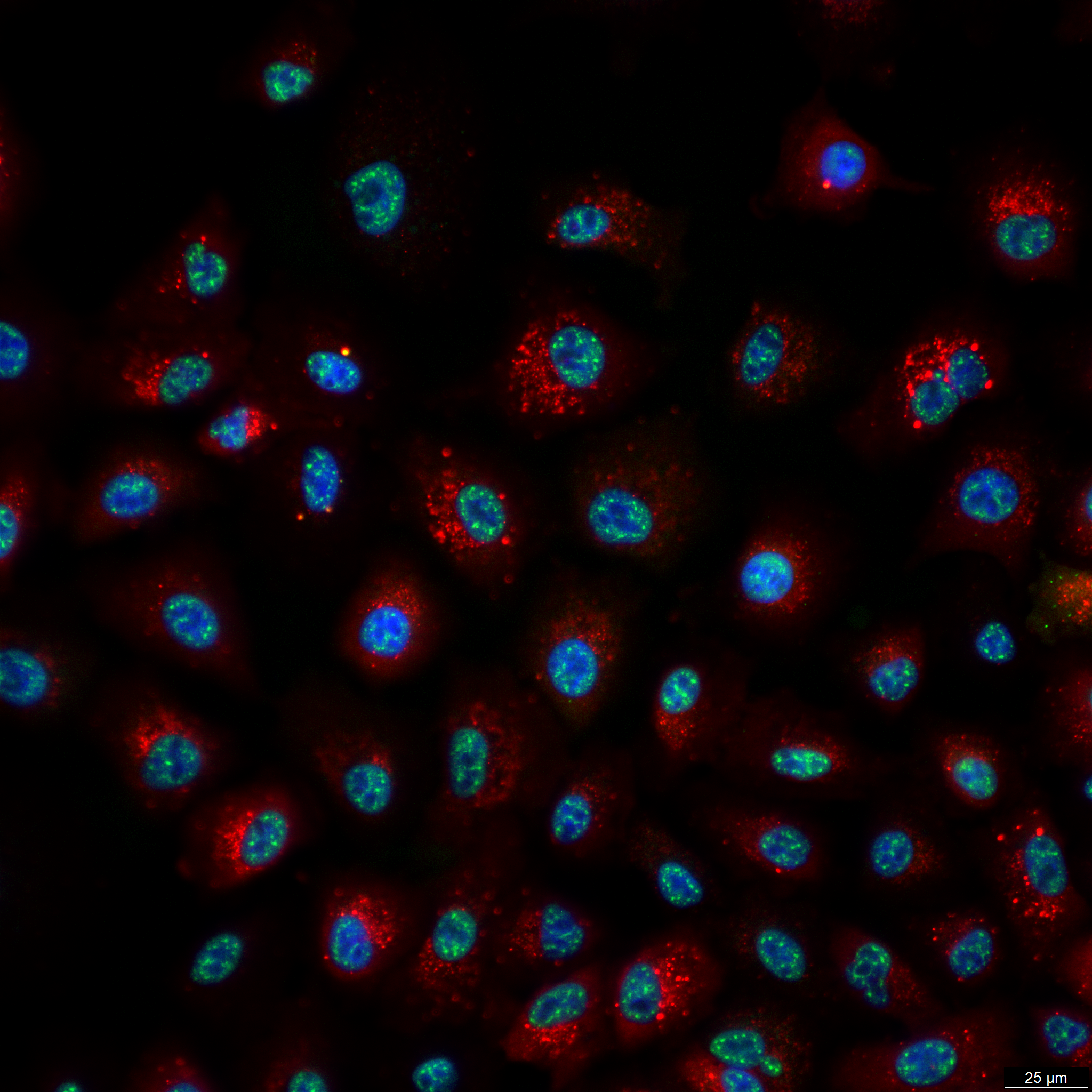

Supplement: Supplementary file 8 — Source data Fig. 4 [file 44318_2025_421_MOESM8_ESM.zip › Figure 4/Figure 4F/Control.tif]

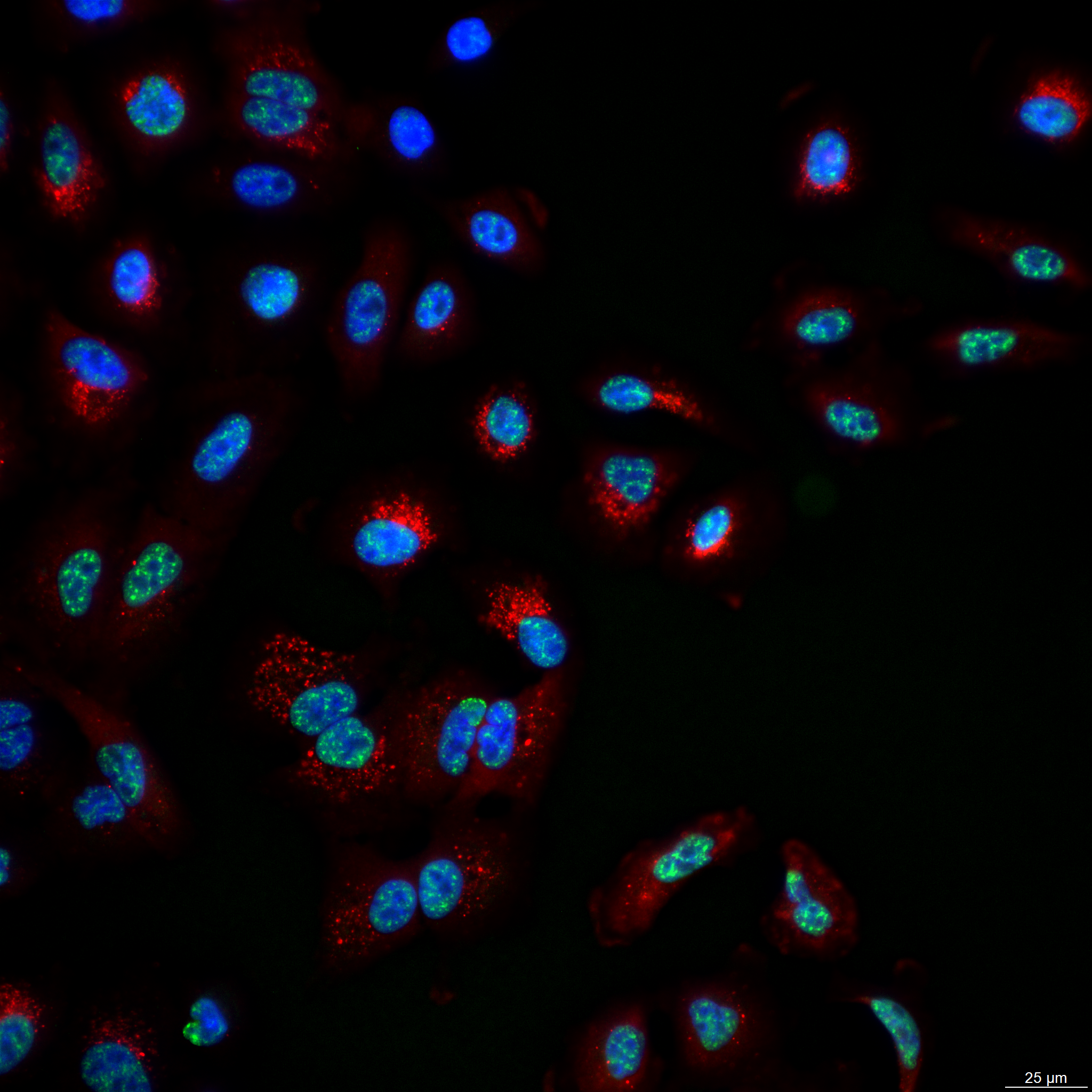

Supplement: Supplementary file 8 — Source data Fig. 4 [file 44318_2025_421_MOESM8_ESM.zip › Figure 4/Figure 4F/lFNγ+RBN.tif]

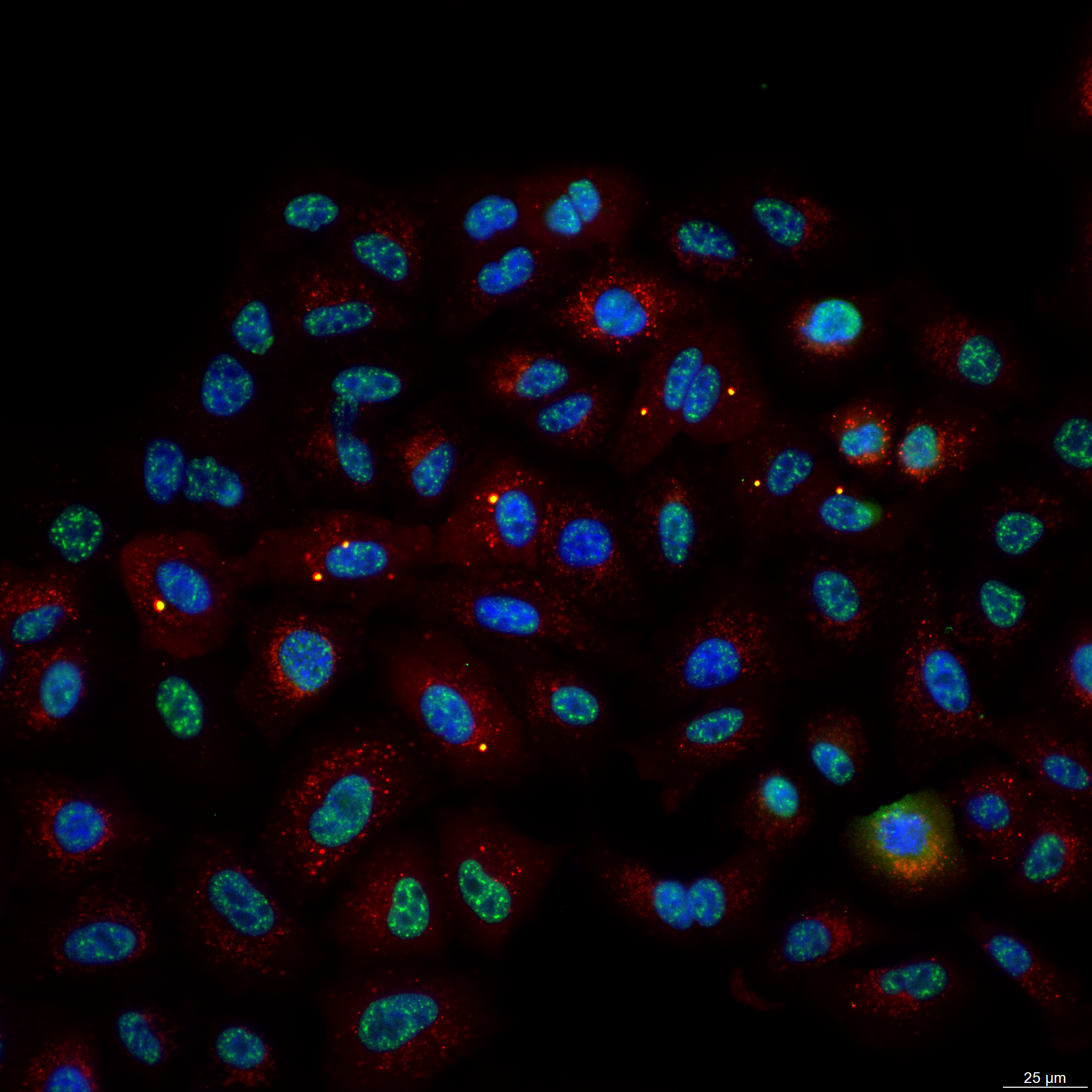

Supplement: Supplementary file 8 — Source data Fig. 4 [file 44318_2025_421_MOESM8_ESM.zip › Figure 4/Figure 4F/lFNγ.tif]

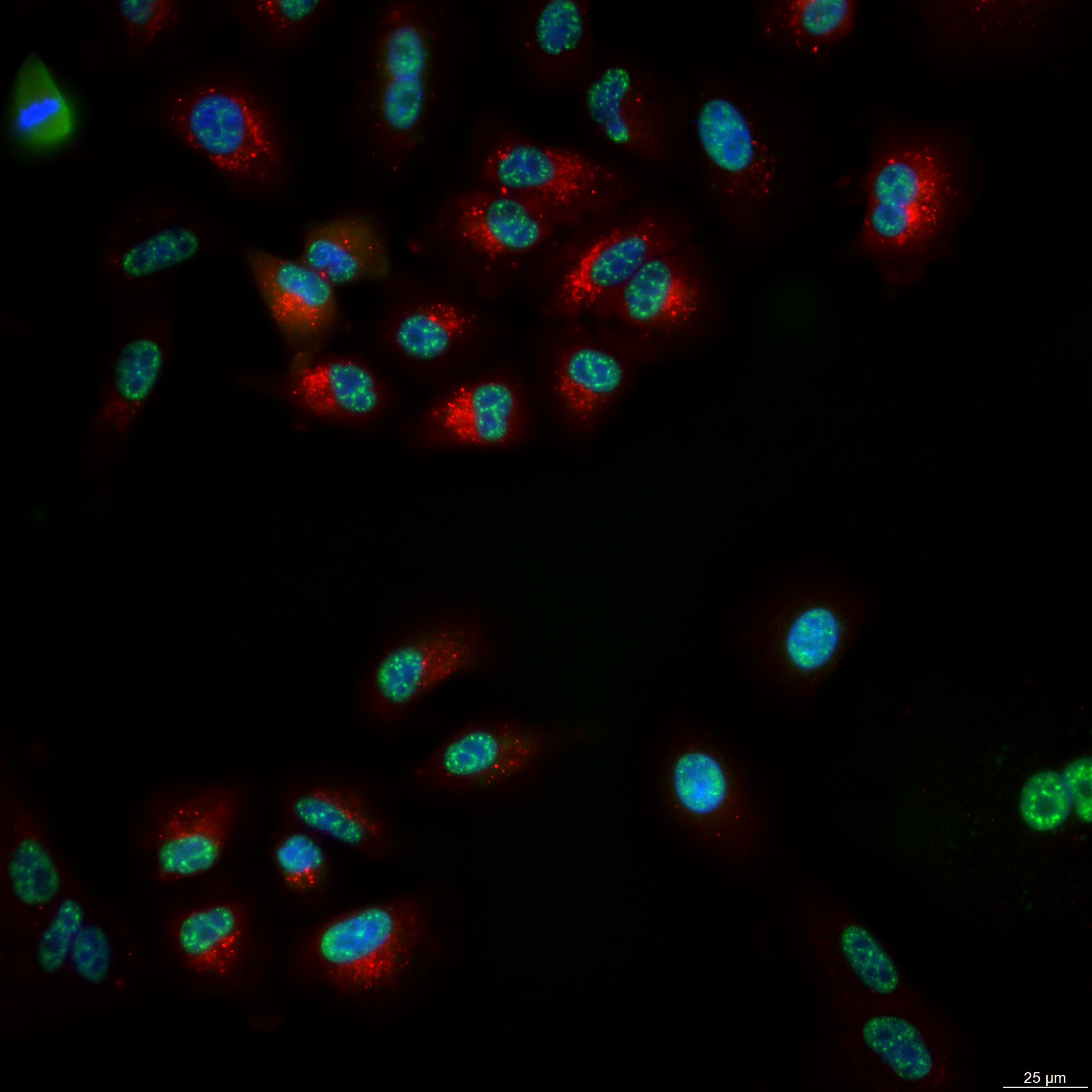

Supplement: Supplementary file 8 — Source data Fig. 4 [file 44318_2025_421_MOESM8_ESM.zip › Figure 4/Figure 4F/RBN.tif]

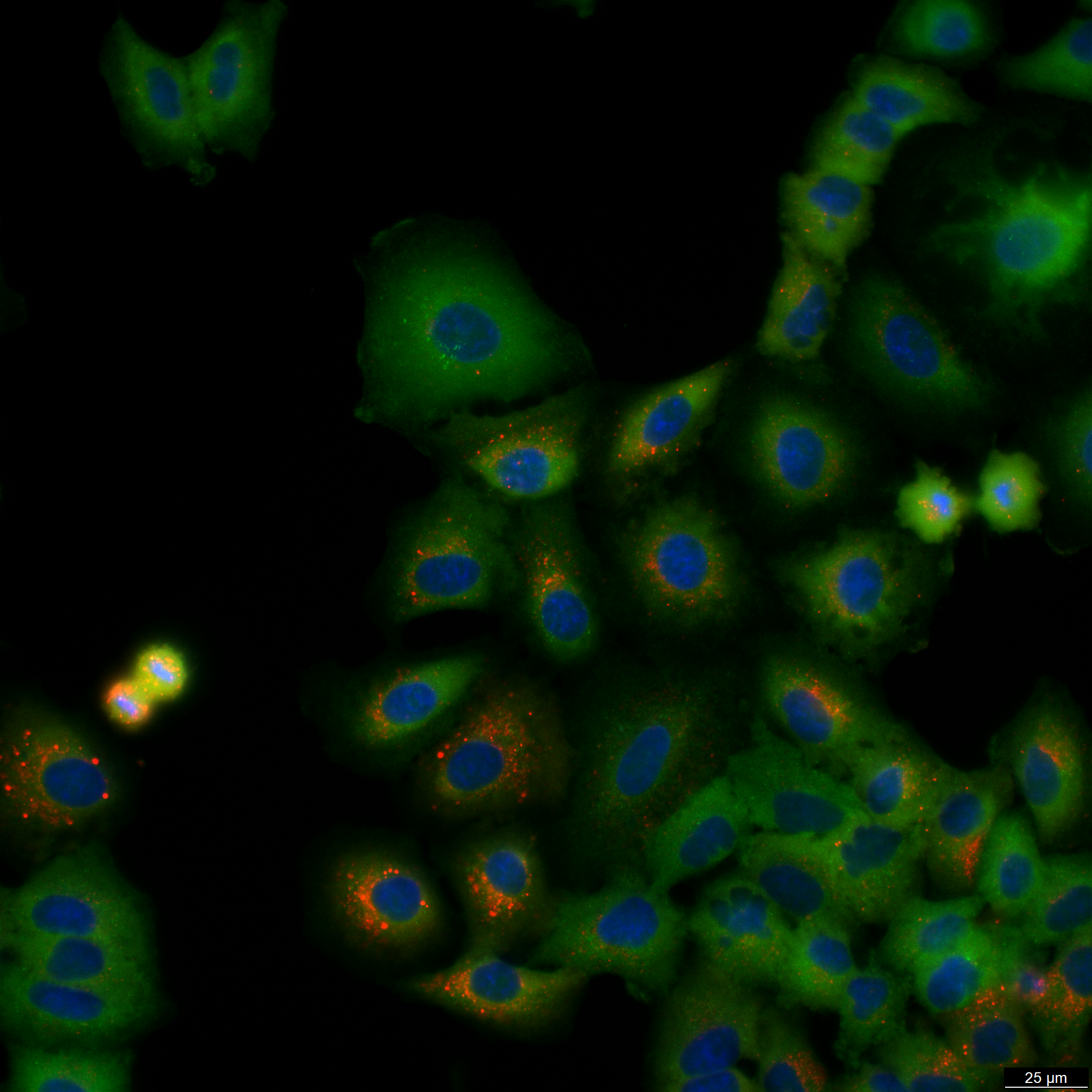

Supplement: Supplementary file 8 — Source data Fig. 4 [file 44318_2025_421_MOESM8_ESM.zip › Figure 4/Figure 4G/Control.tif]

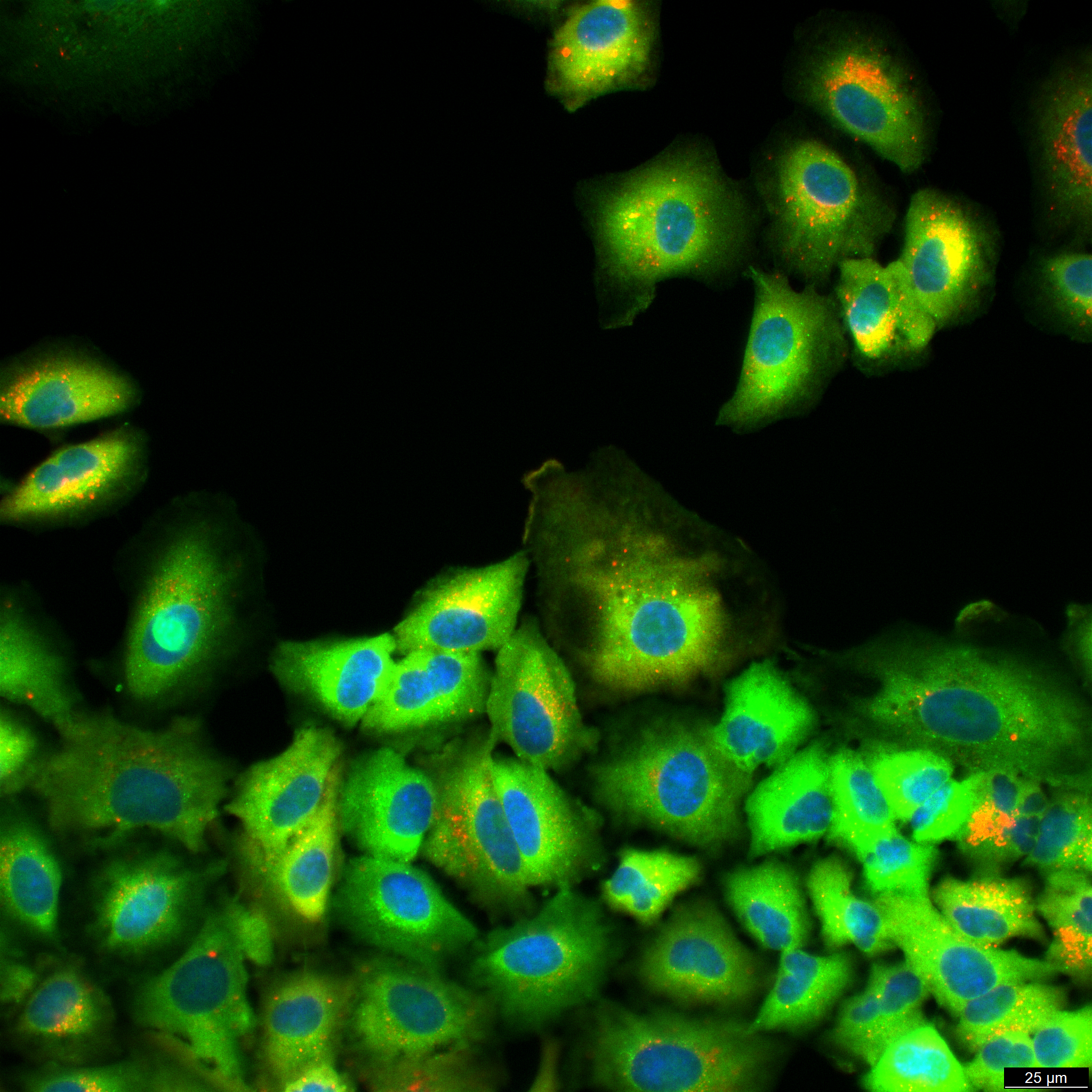

Supplement: Supplementary file 8 — Source data Fig. 4 [file 44318_2025_421_MOESM8_ESM.zip › Figure 4/Figure 4G/lFNγ+RBN.tif]

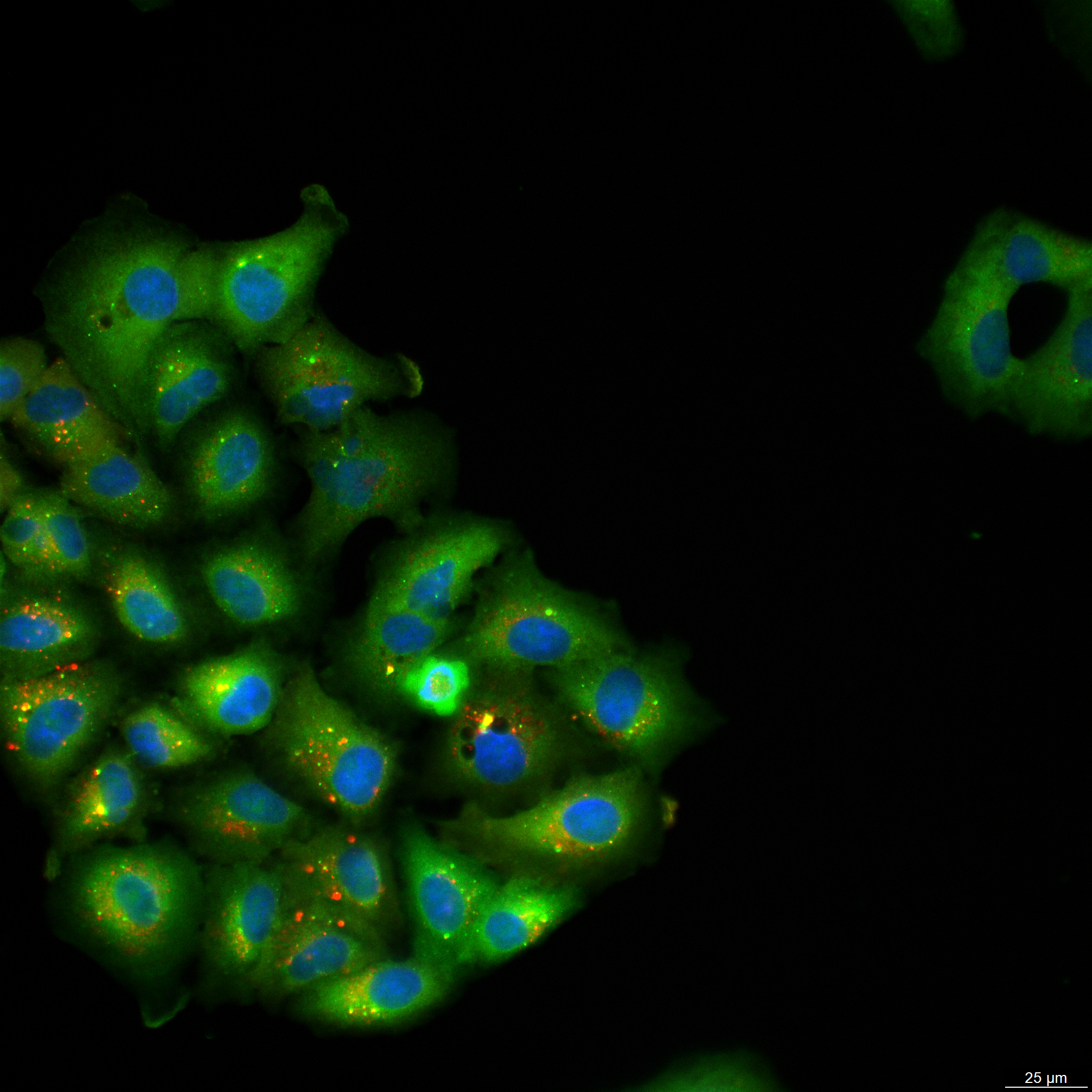

Supplement: Supplementary file 8 — Source data Fig. 4 [file 44318_2025_421_MOESM8_ESM.zip › Figure 4/Figure 4G/lFNγ.tif]

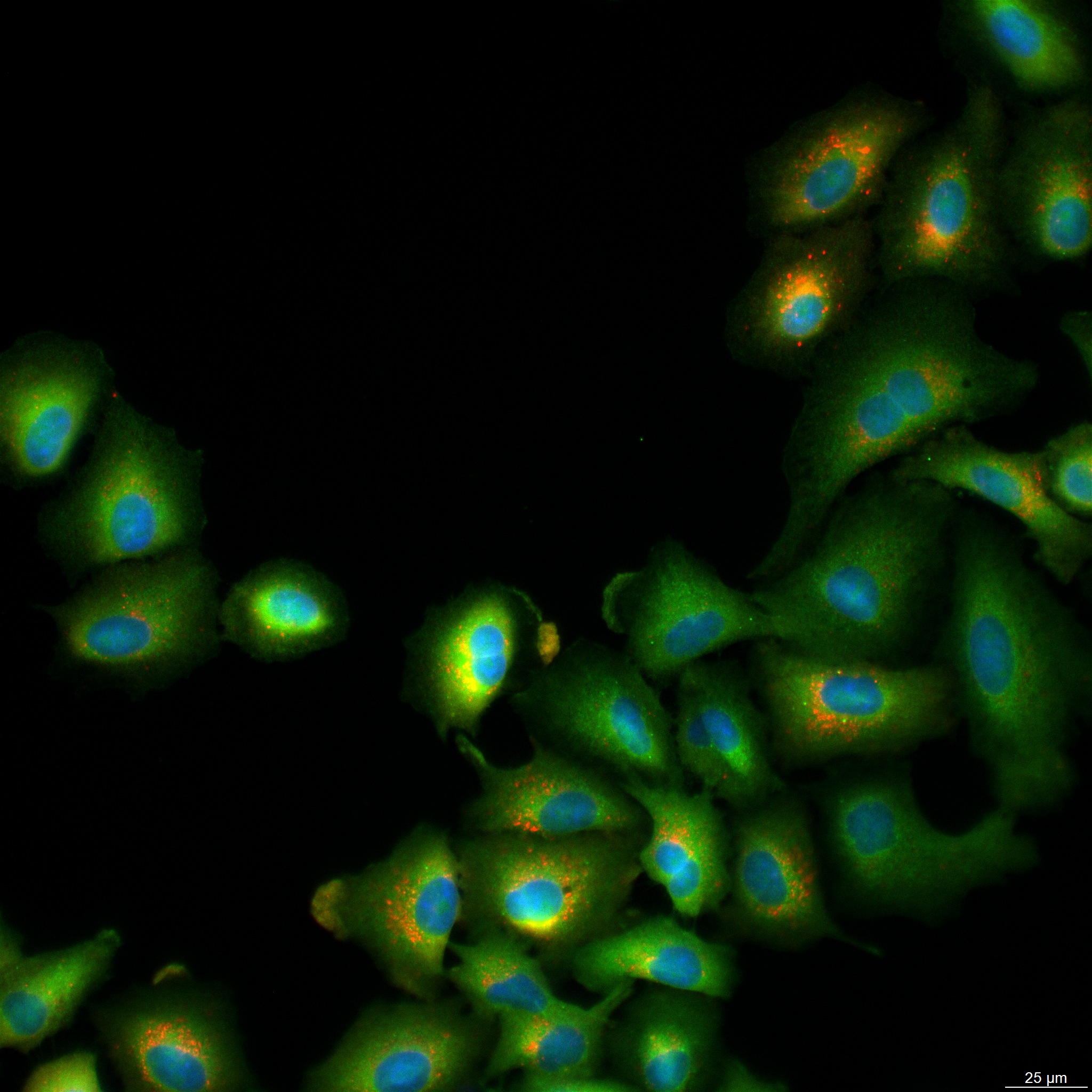

Supplement: Supplementary file 8 — Source data Fig. 4 [file 44318_2025_421_MOESM8_ESM.zip › Figure 4/Figure 4G/RBN.tif]

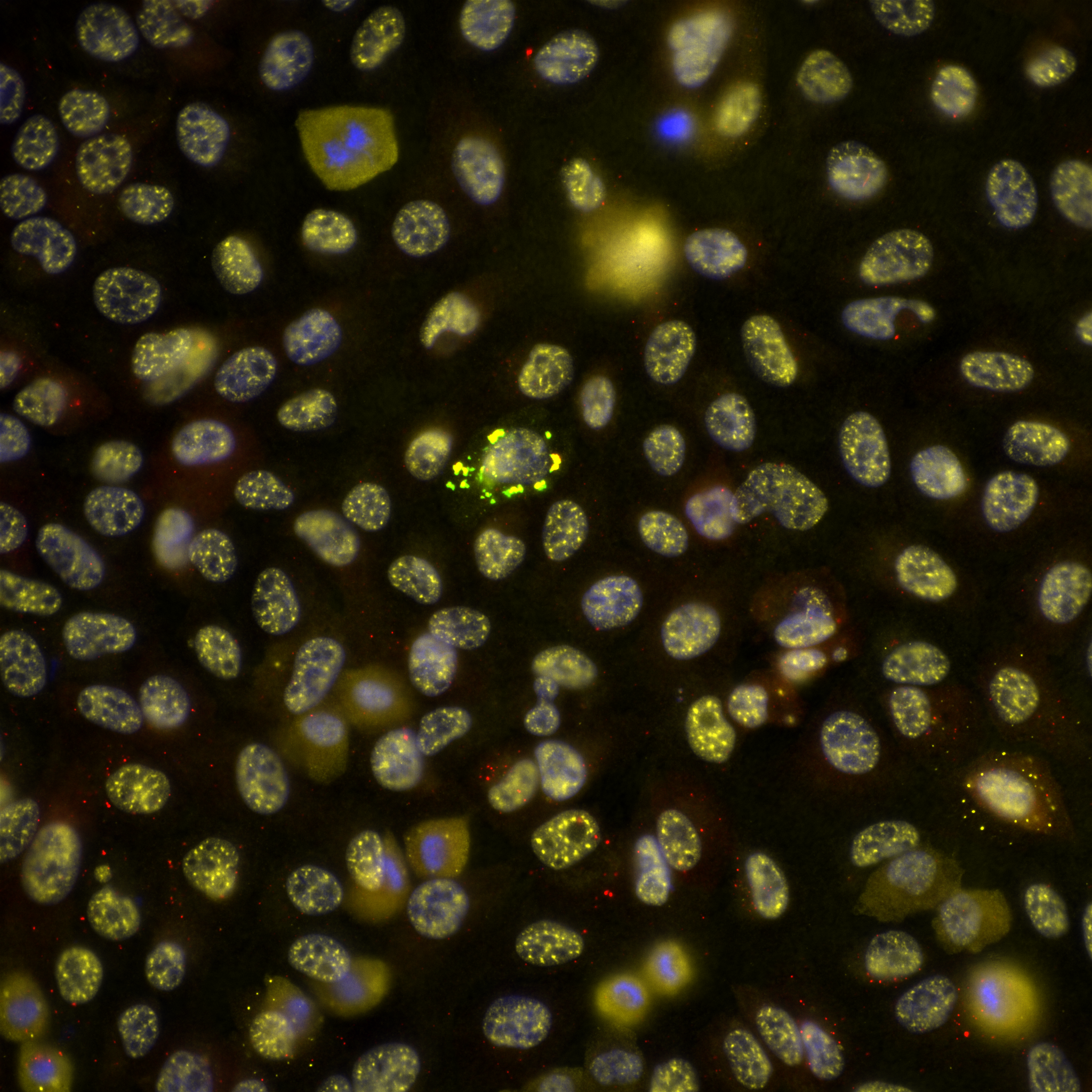

Supplement: Supplementary file 8 — Source data Fig. 4 [file 44318_2025_421_MOESM8_ESM.zip › Figure 4/Figure 4H/Cell_1.tif]

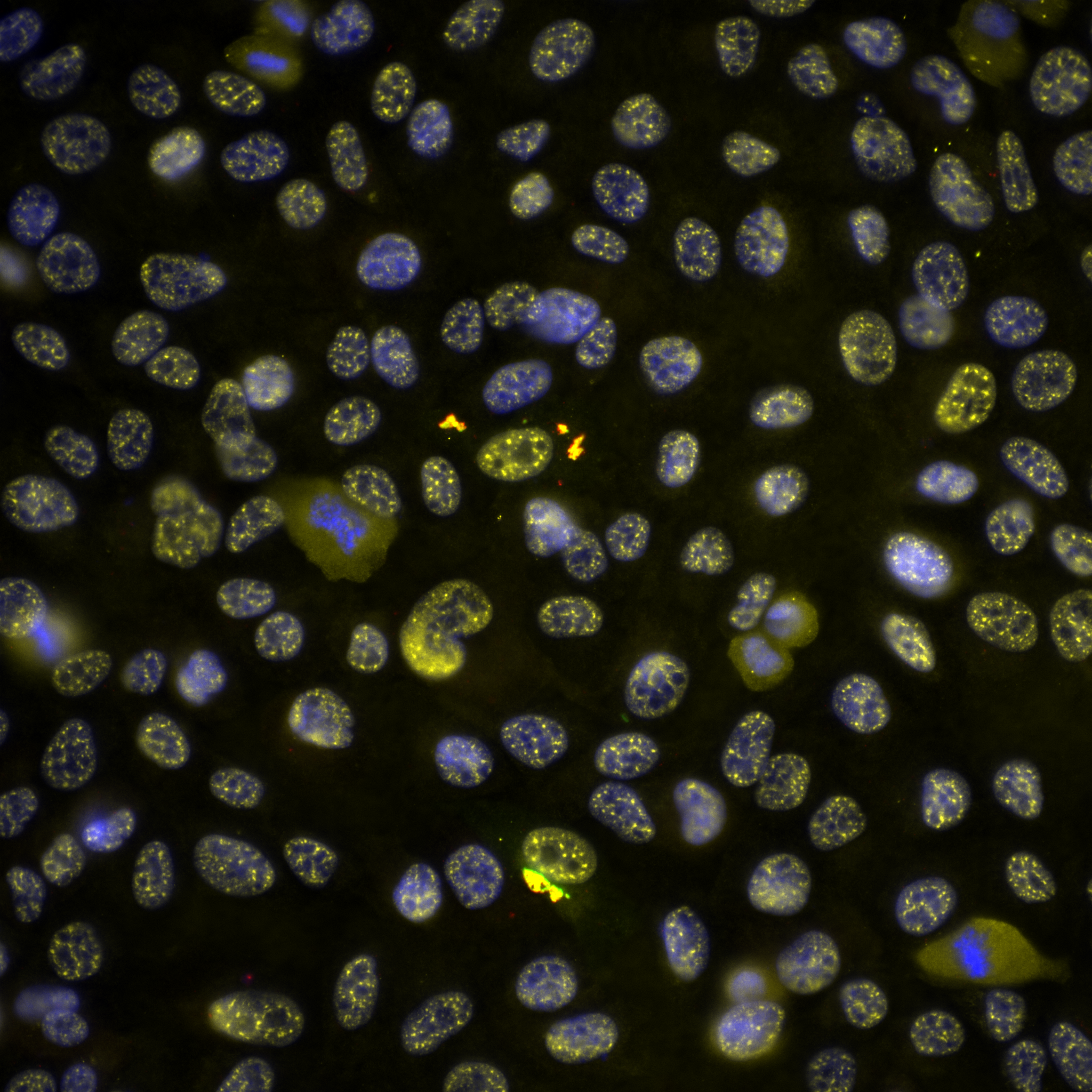

Supplement: Supplementary file 8 — Source data Fig. 4 [file 44318_2025_421_MOESM8_ESM.zip › Figure 4/Figure 4H/Cell_10.tif]

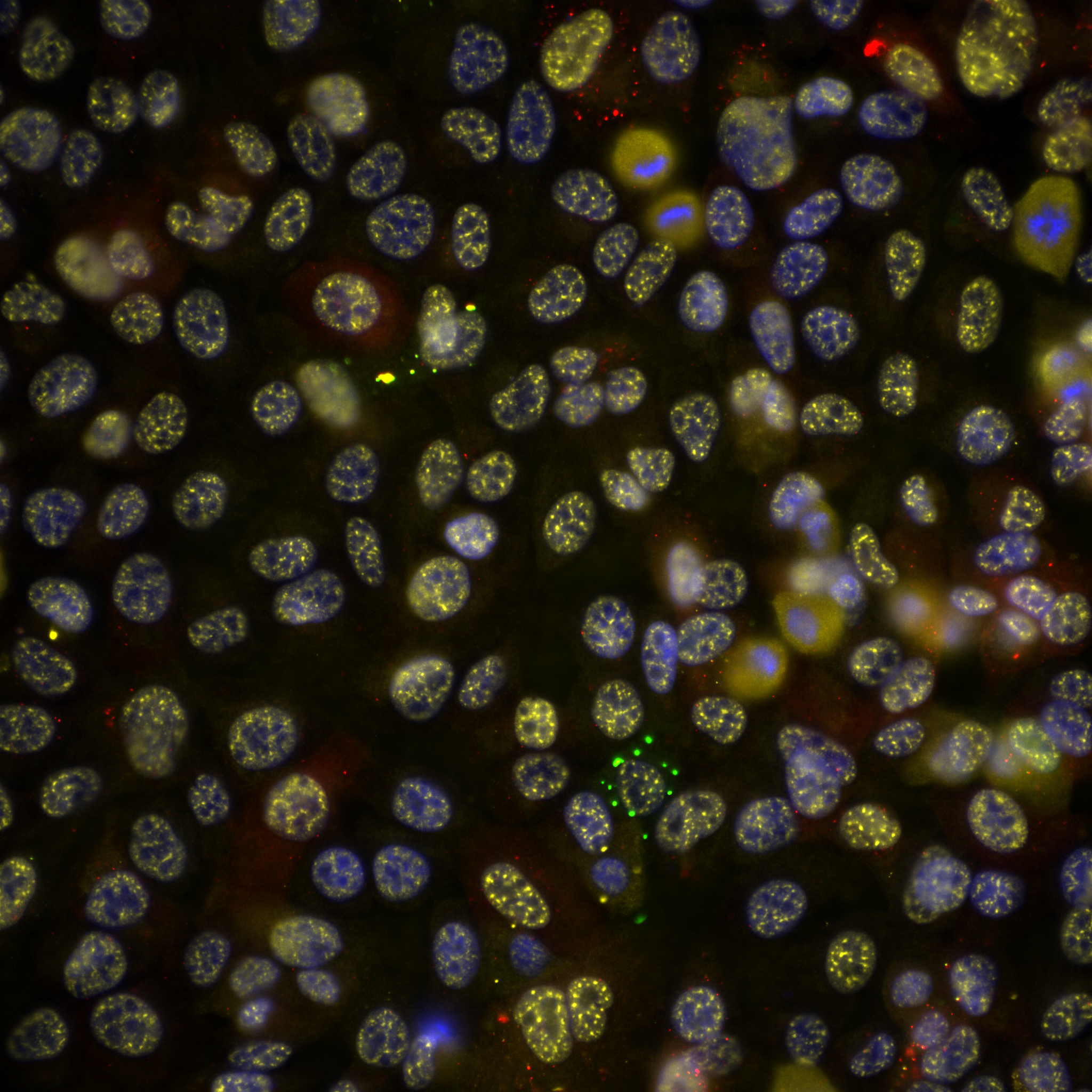

Supplement: Supplementary file 8 — Source data Fig. 4 [file 44318_2025_421_MOESM8_ESM.zip › Figure 4/Figure 4H/Cell_11.tif]

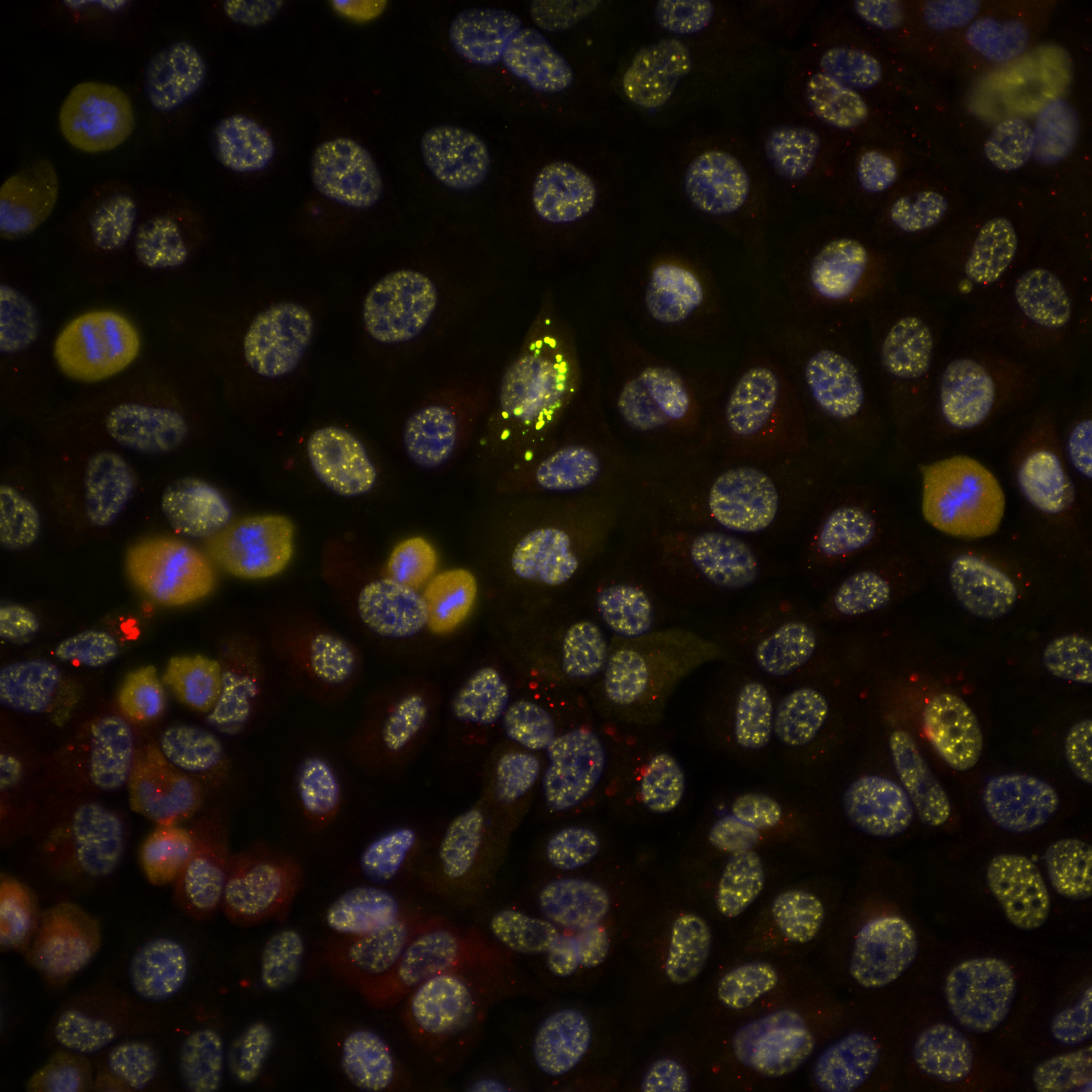

Supplement: Supplementary file 8 — Source data Fig. 4 [file 44318_2025_421_MOESM8_ESM.zip › Figure 4/Figure 4H/Cell_12.tif]

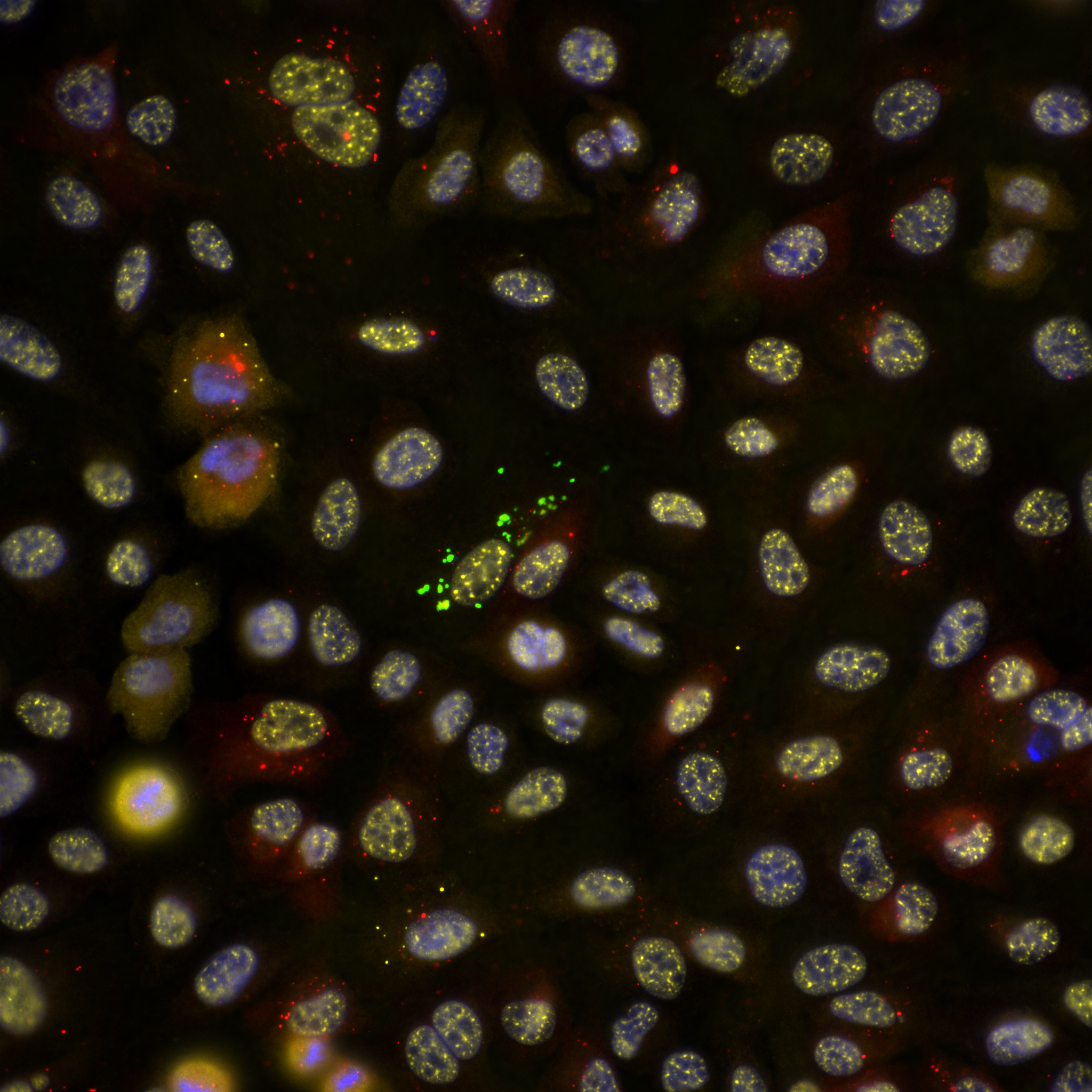

Supplement: Supplementary file 8 — Source data Fig. 4 [file 44318_2025_421_MOESM8_ESM.zip › Figure 4/Figure 4H/Cell_2.tif]

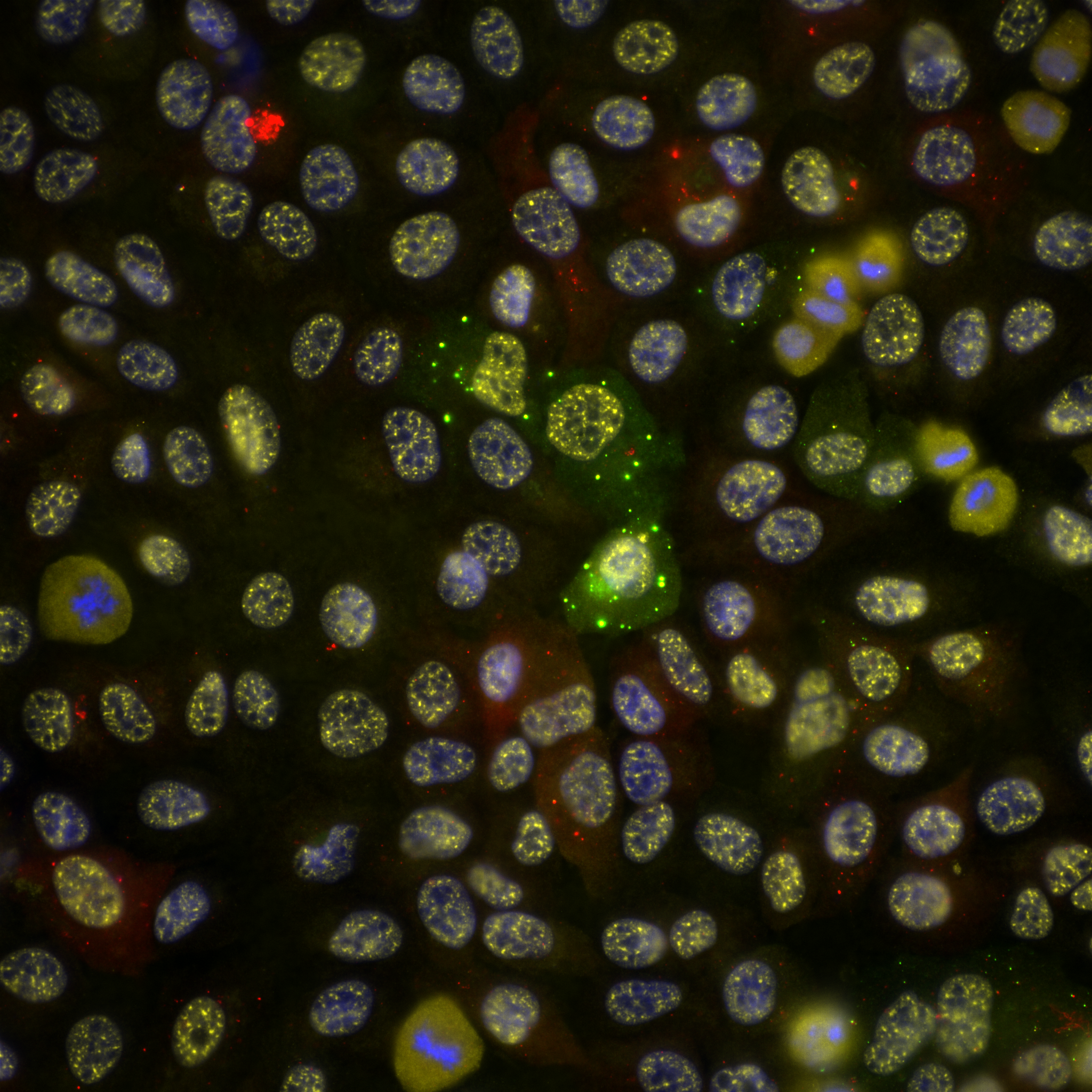

Supplement: Supplementary file 8 — Source data Fig. 4 [file 44318_2025_421_MOESM8_ESM.zip › Figure 4/Figure 4H/Cell_3_4.tif]

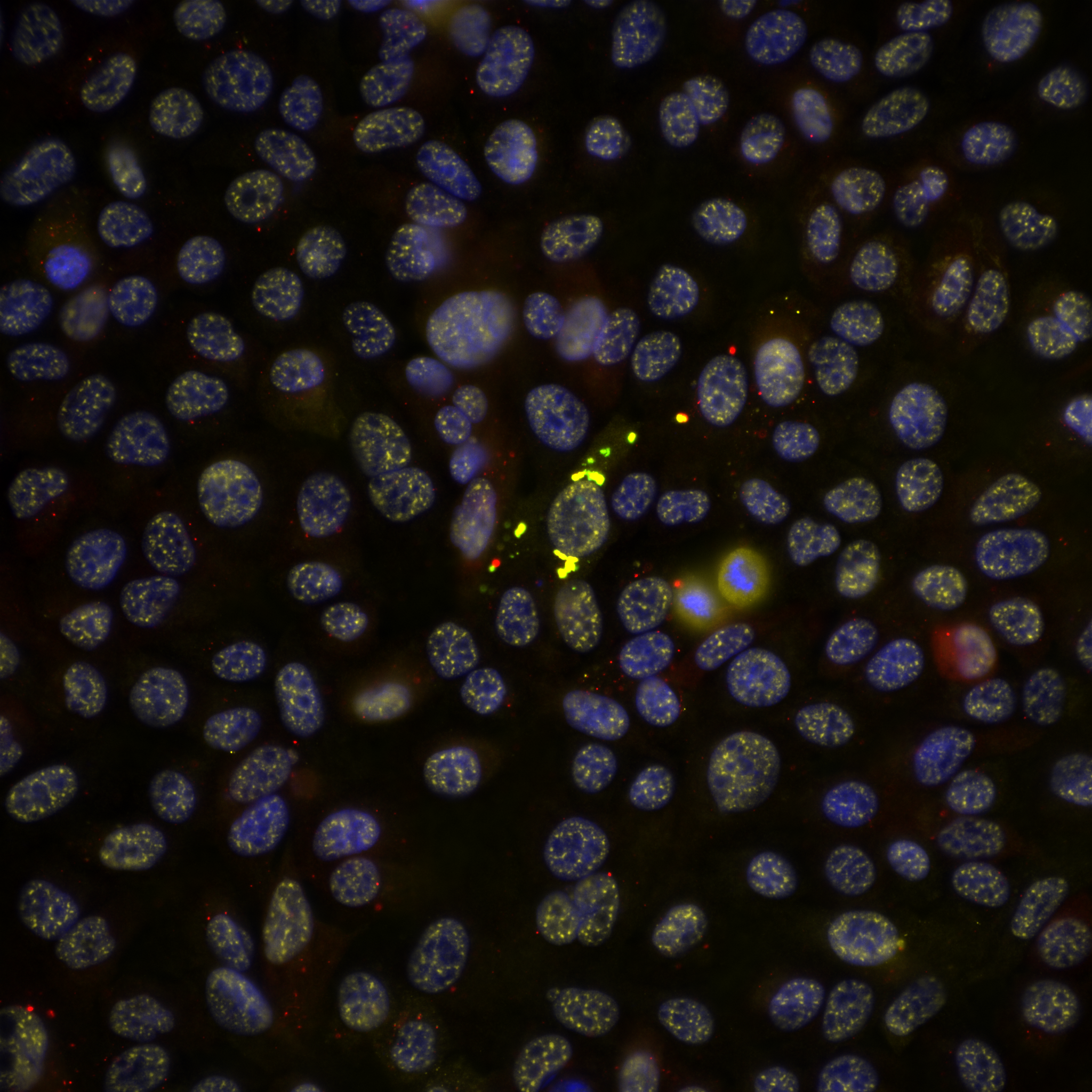

Supplement: Supplementary file 8 — Source data Fig. 4 [file 44318_2025_421_MOESM8_ESM.zip › Figure 4/Figure 4H/Cell_5.tif]

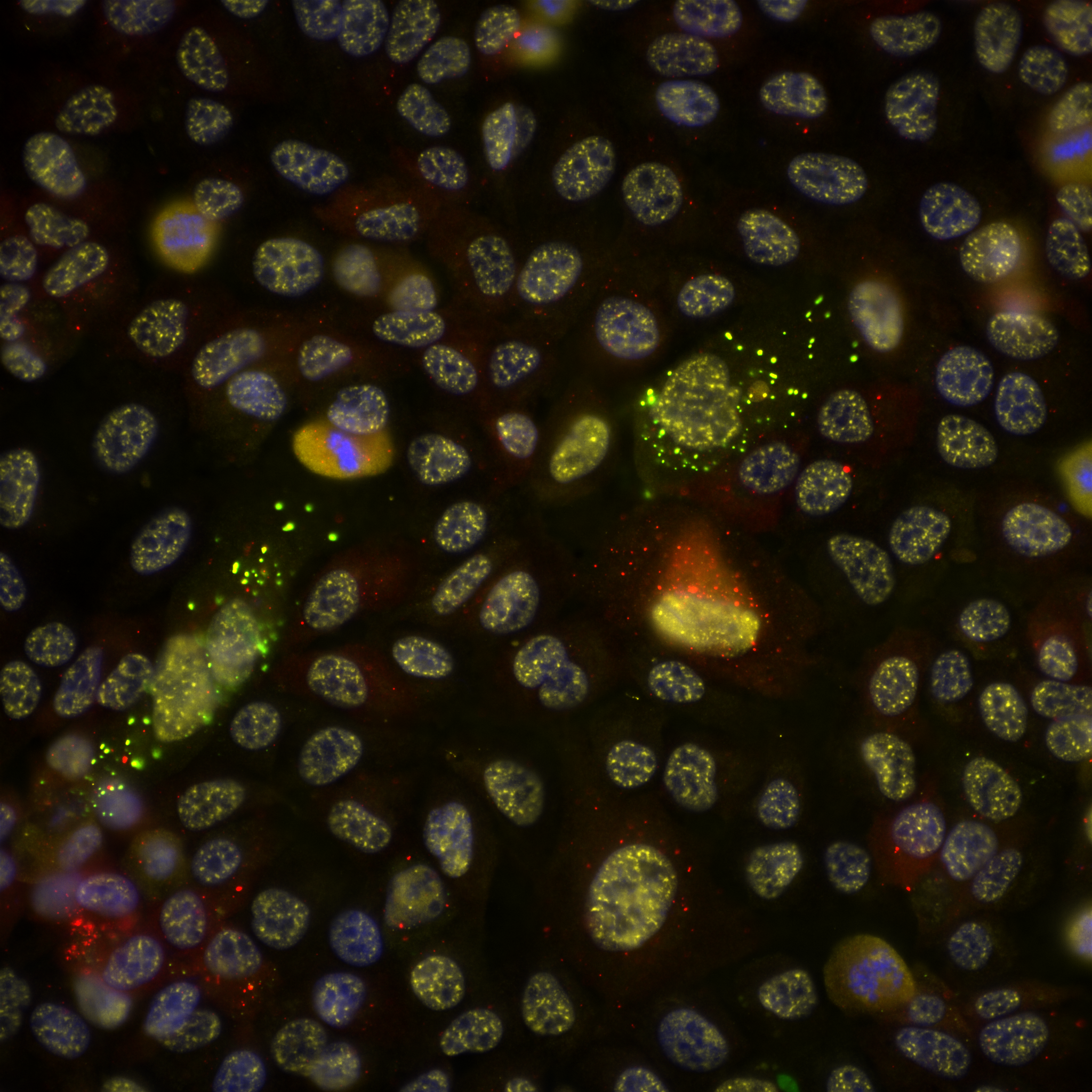

Supplement: Supplementary file 8 — Source data Fig. 4 [file 44318_2025_421_MOESM8_ESM.zip › Figure 4/Figure 4H/Cell_6.tif]

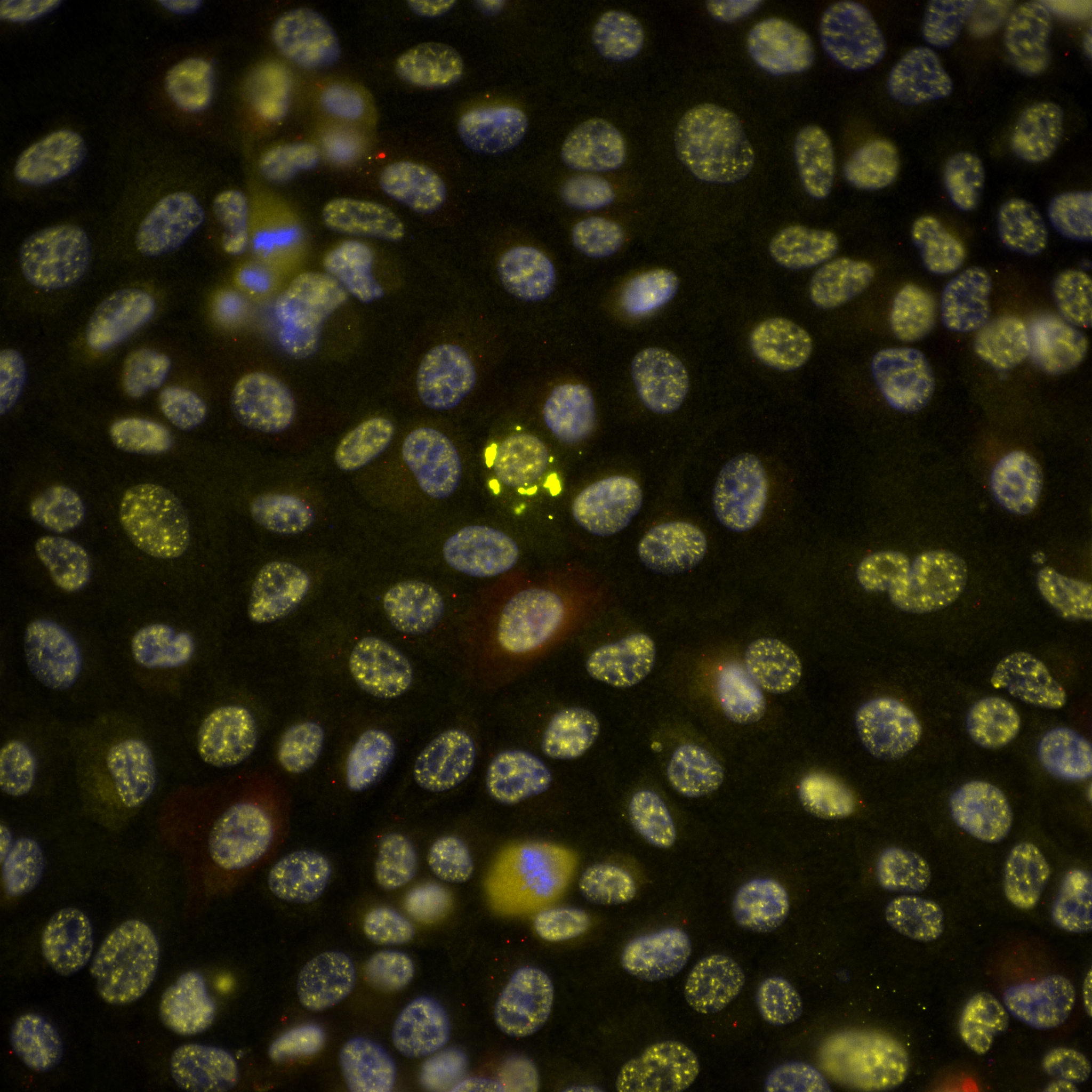

Supplement: Supplementary file 8 — Source data Fig. 4 [file 44318_2025_421_MOESM8_ESM.zip › Figure 4/Figure 4H/Cell_7.tif]

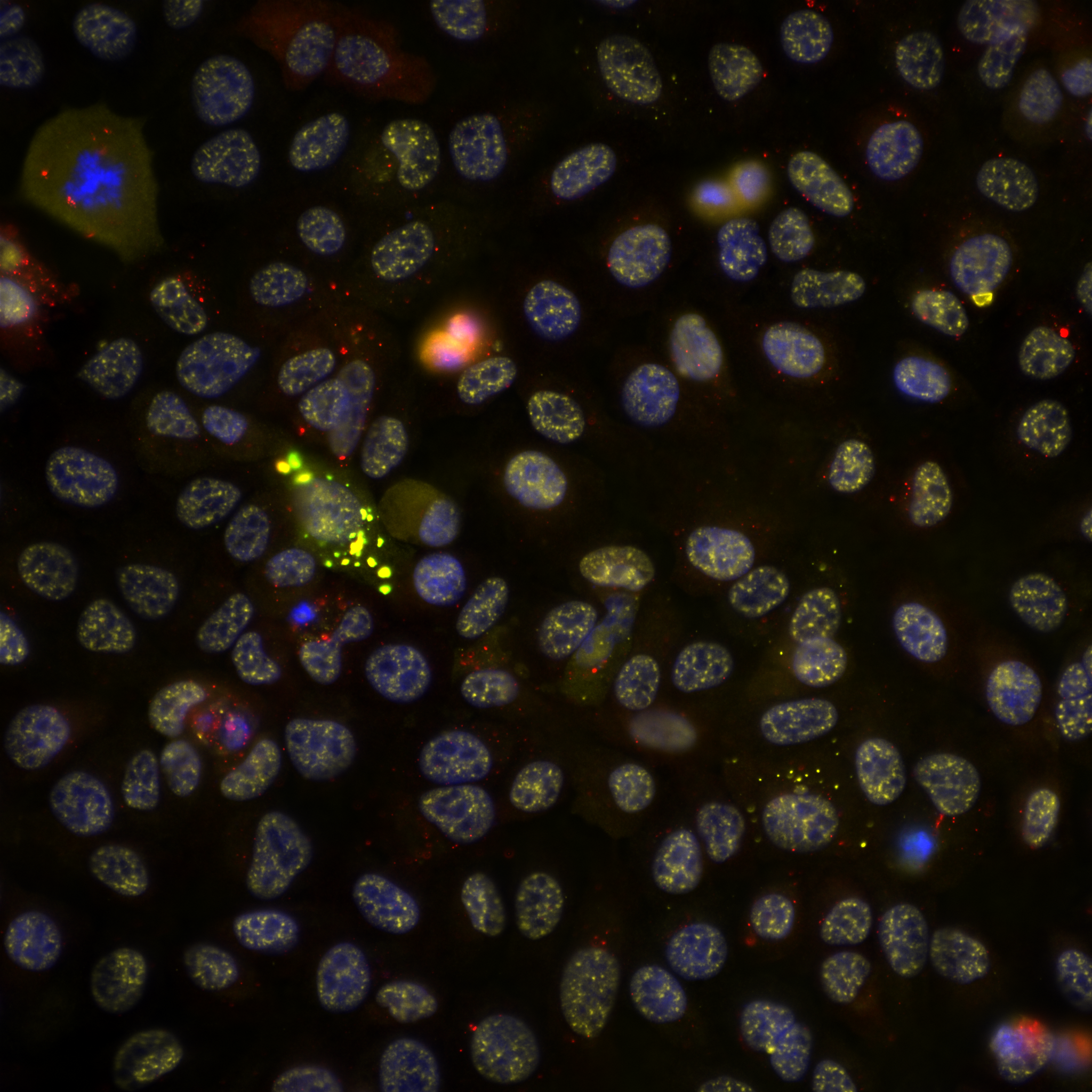

Supplement: Supplementary file 8 — Source data Fig. 4 [file 44318_2025_421_MOESM8_ESM.zip › Figure 4/Figure 4H/Cell_8.tif]
