# Supplementary figures and images for: Interferon-induced PARP14-mediated ADP-ribosylation in p62 bodies requires the ubiquitin-proteasome system (part 2 of 4)
Source: EMBO J. 2025 Apr 7;44(10):2741–73. doi: 10.1038/s44318-025-00421-4 (PMC12084362; doi:10.1038/s44318-025-00421-4)

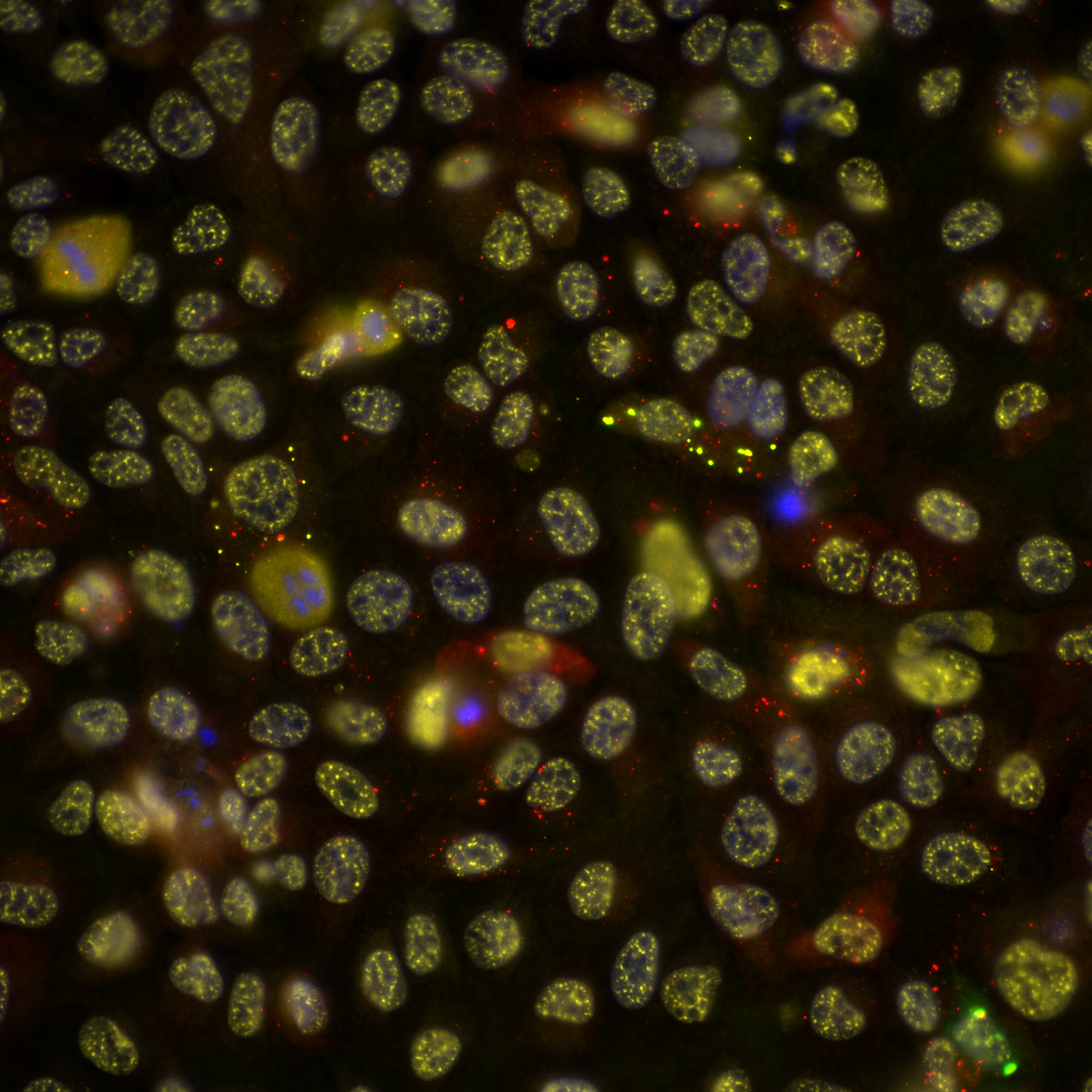

Supplement: Supplementary file 8 — Source data Fig. 4 [file 44318_2025_421_MOESM8_ESM.zip › Figure 4/Figure 4H/Cell_9.tif]

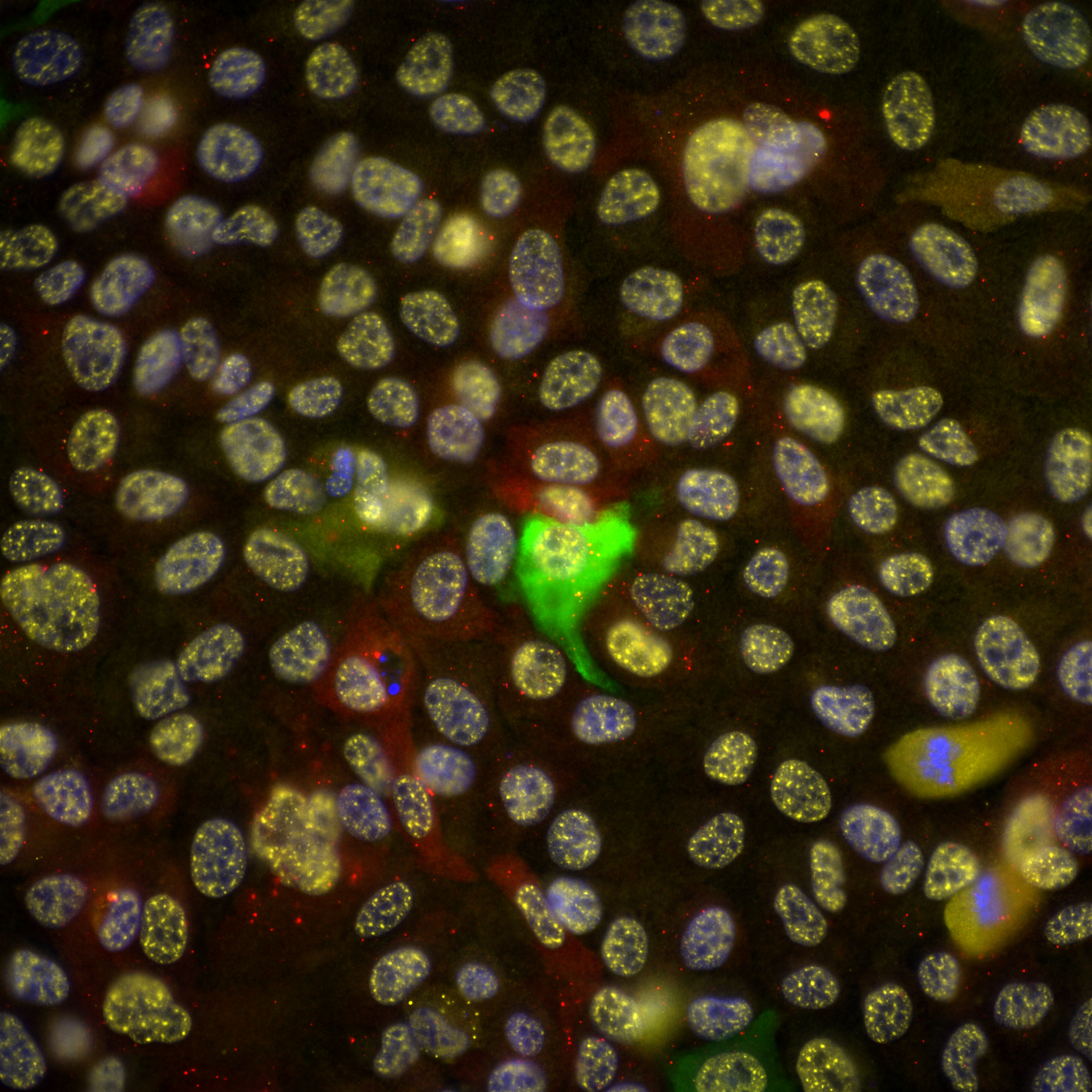

Supplement: Supplementary file 8 — Source data Fig. 4 [file 44318_2025_421_MOESM8_ESM.zip › Figure 4/Figure 4I/Cell_1.tif]

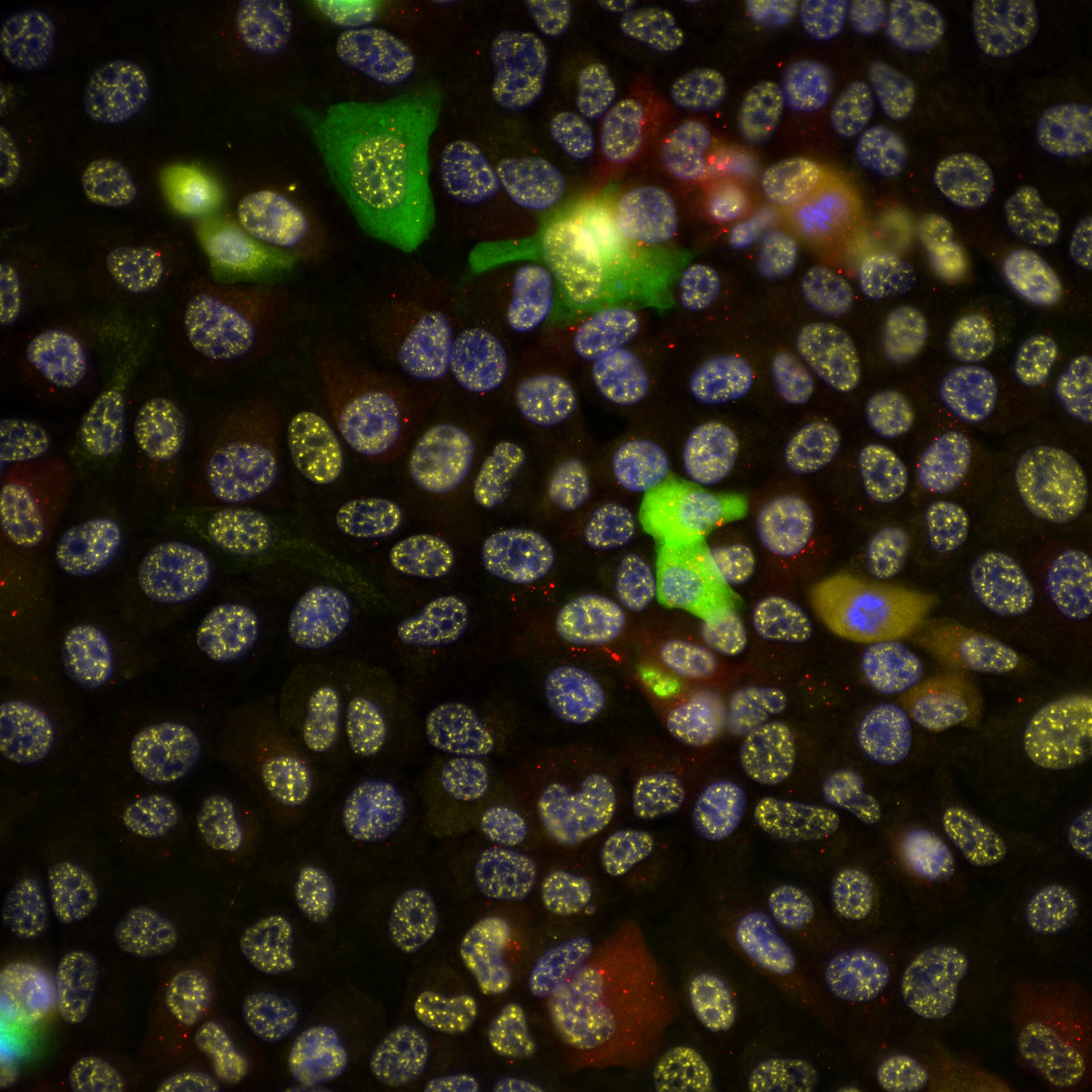

Supplement: Supplementary file 8 — Source data Fig. 4 [file 44318_2025_421_MOESM8_ESM.zip › Figure 4/Figure 4I/Cell_10.tif]

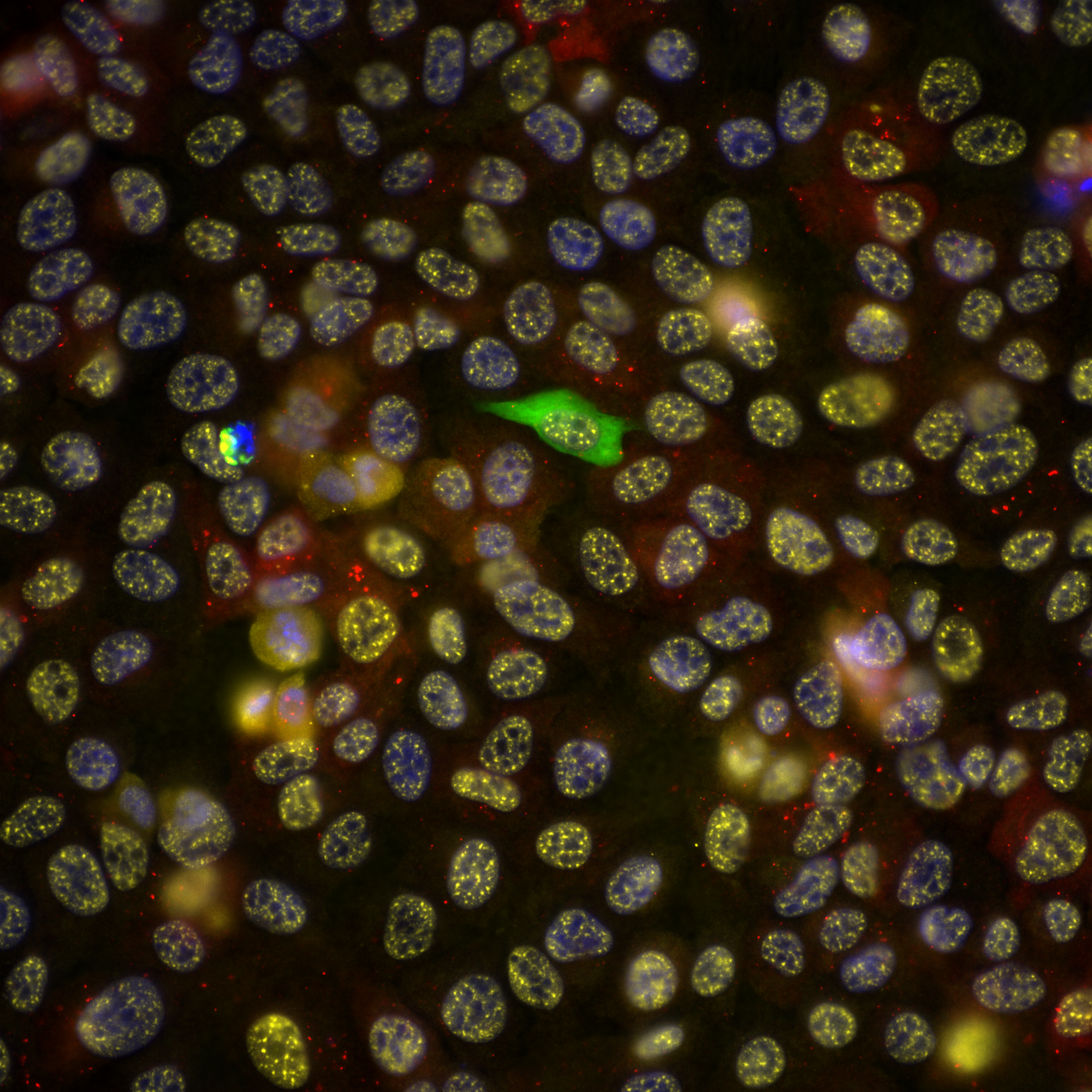

Supplement: Supplementary file 8 — Source data Fig. 4 [file 44318_2025_421_MOESM8_ESM.zip › Figure 4/Figure 4I/Cell_11.tif]

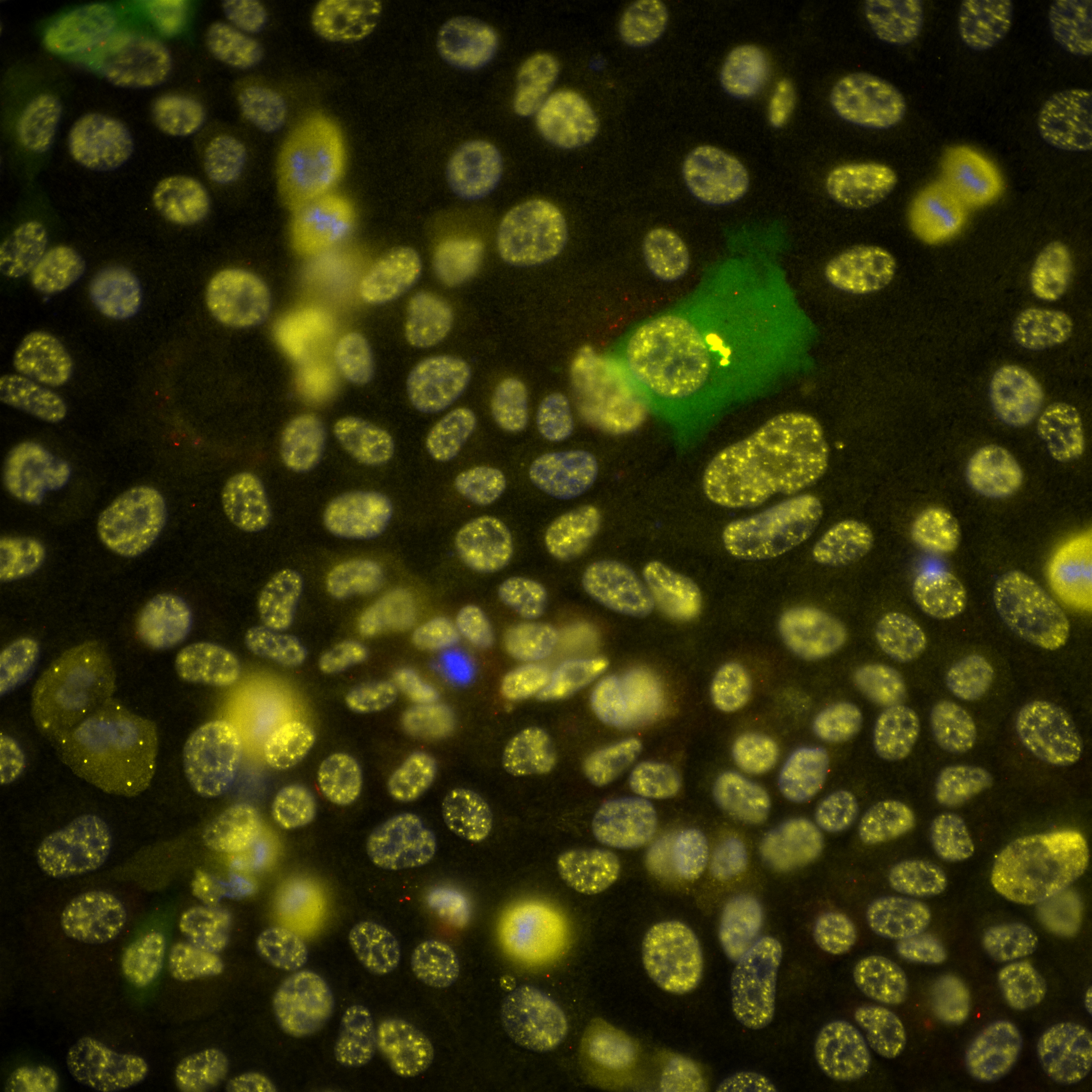

Supplement: Supplementary file 8 — Source data Fig. 4 [file 44318_2025_421_MOESM8_ESM.zip › Figure 4/Figure 4I/Cell_12.tif]

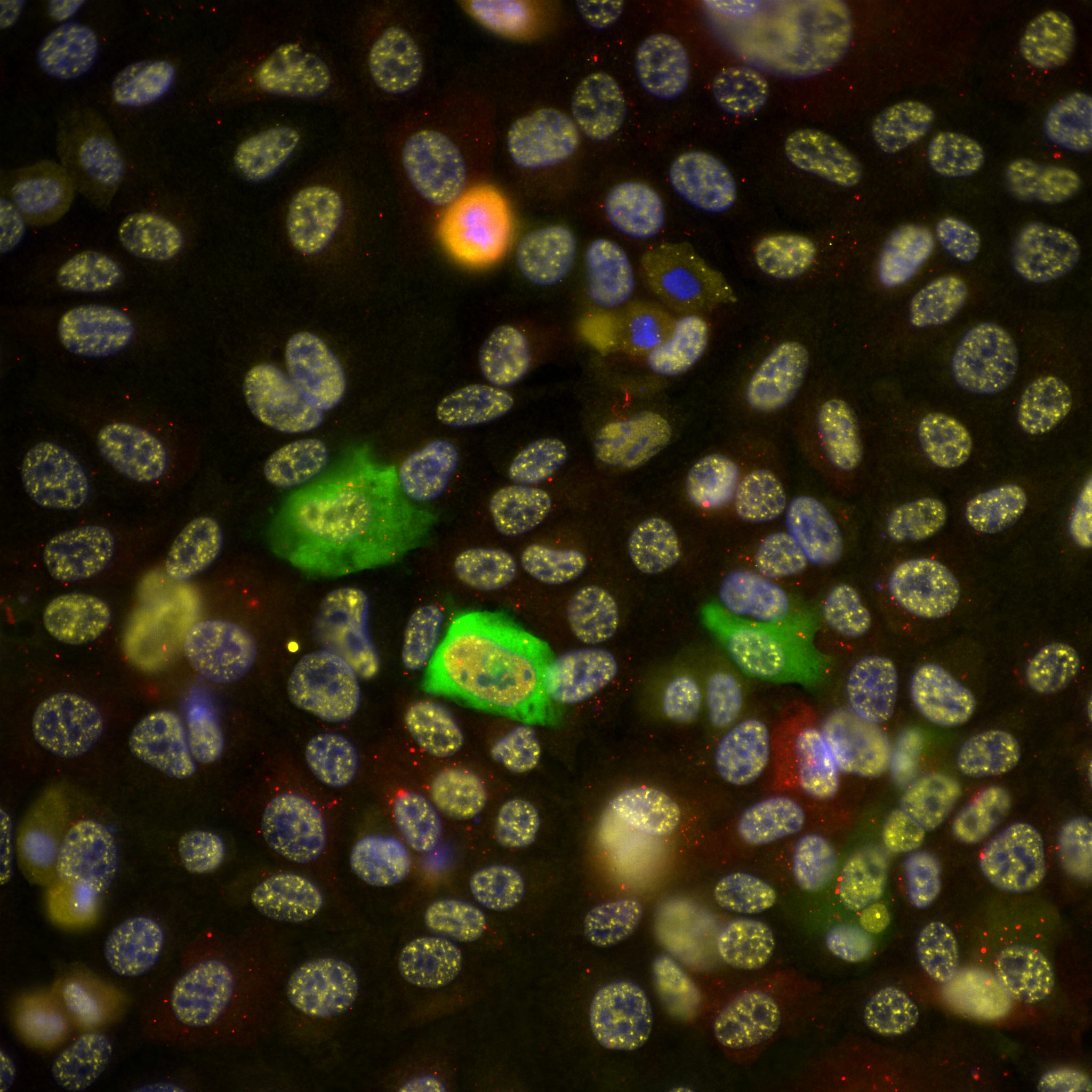

Supplement: Supplementary file 8 — Source data Fig. 4 [file 44318_2025_421_MOESM8_ESM.zip › Figure 4/Figure 4I/Cell_2_3.tif]

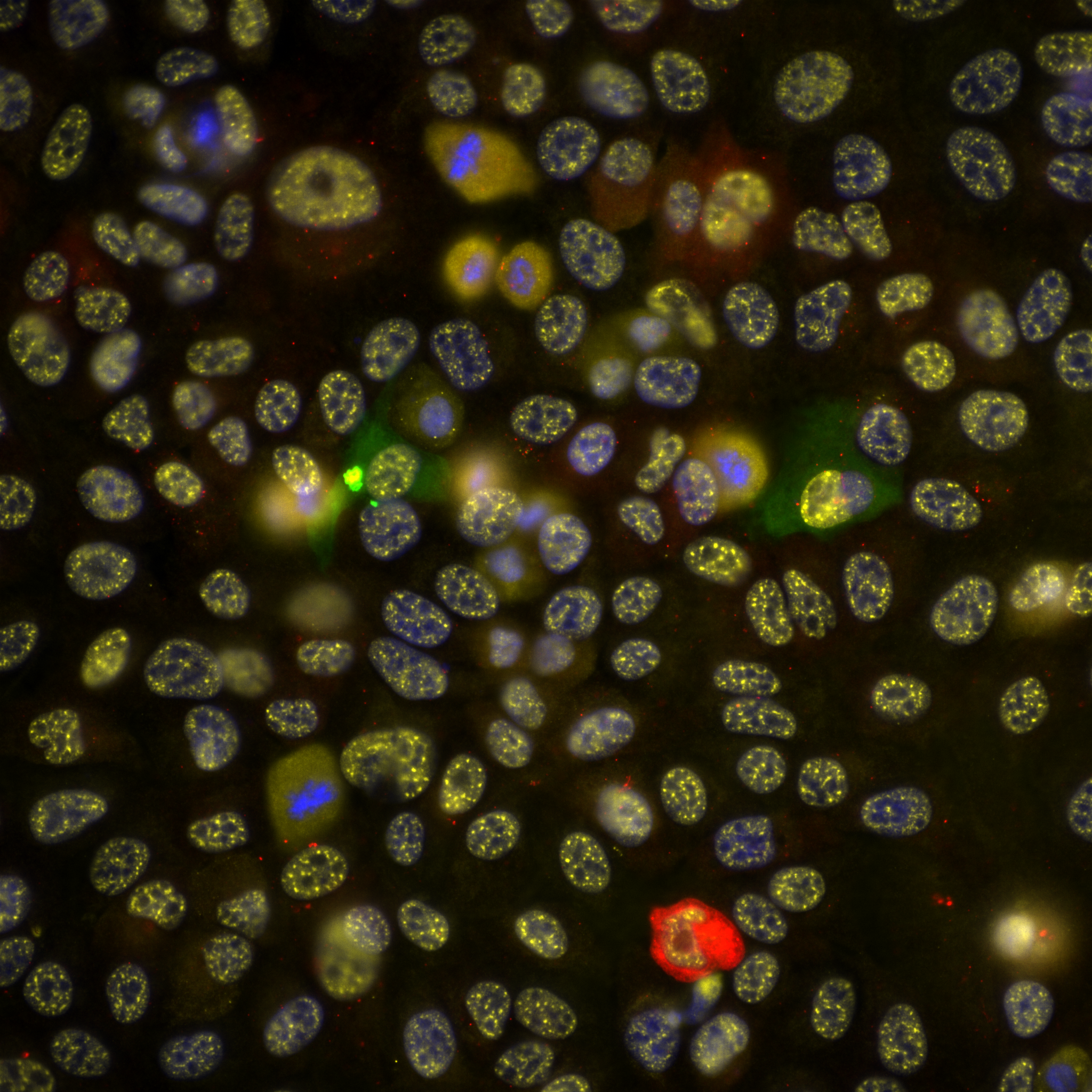

Supplement: Supplementary file 8 — Source data Fig. 4 [file 44318_2025_421_MOESM8_ESM.zip › Figure 4/Figure 4I/Cell_4.tif]

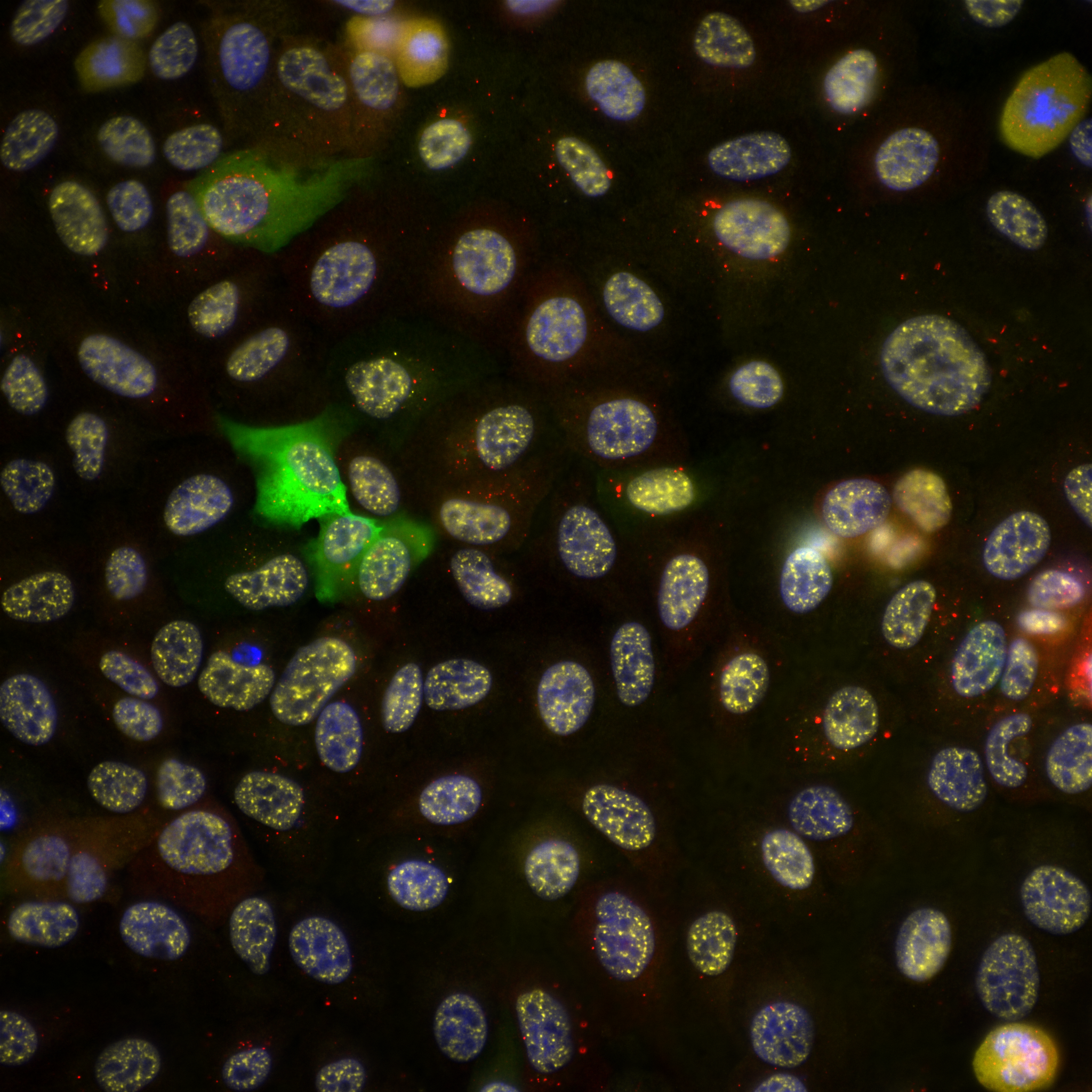

Supplement: Supplementary file 8 — Source data Fig. 4 [file 44318_2025_421_MOESM8_ESM.zip › Figure 4/Figure 4I/Cell_5_6.tif]

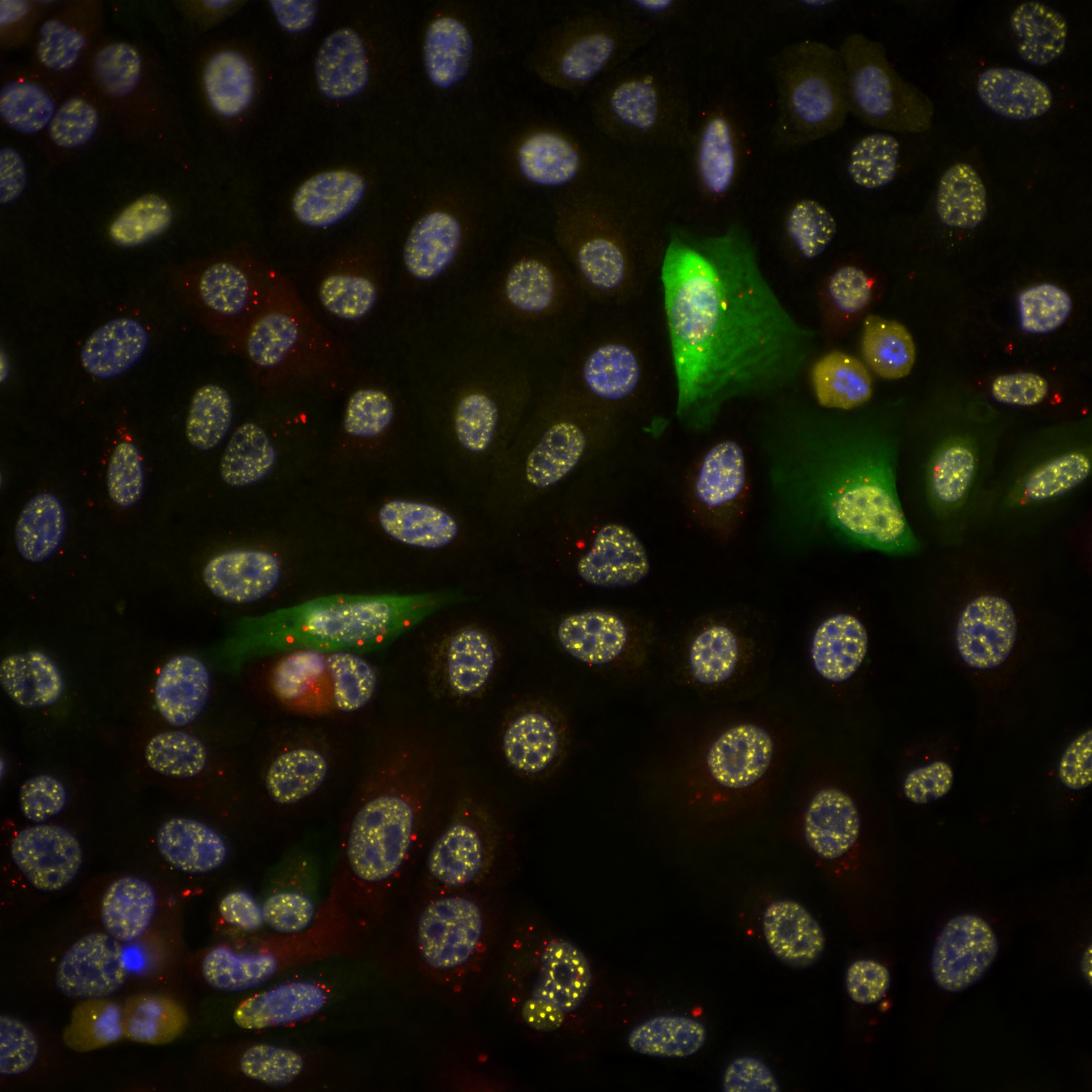

Supplement: Supplementary file 8 — Source data Fig. 4 [file 44318_2025_421_MOESM8_ESM.zip › Figure 4/Figure 4I/Cell_7_8.tif]

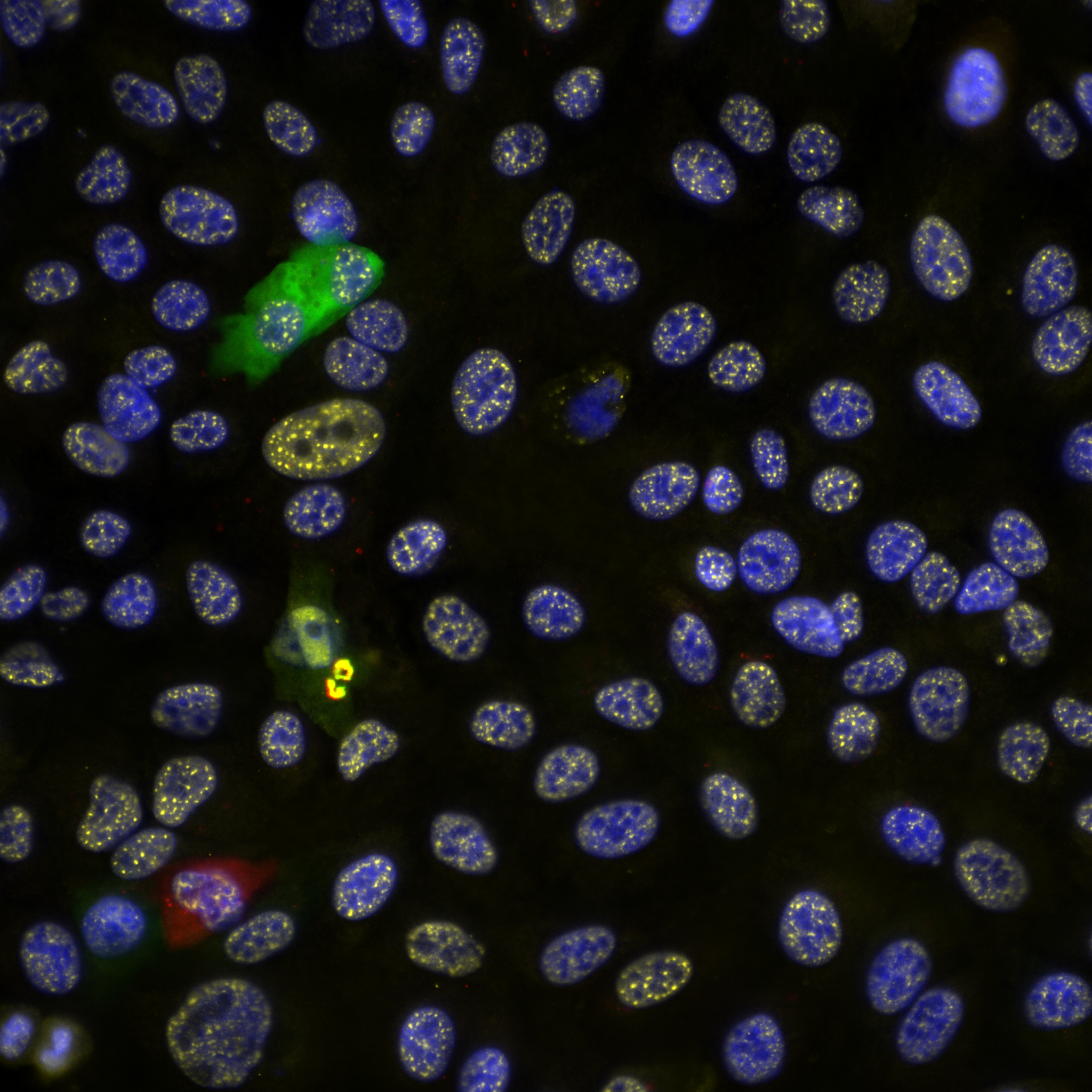

Supplement: Supplementary file 8 — Source data Fig. 4 [file 44318_2025_421_MOESM8_ESM.zip › Figure 4/Figure 4I/Cell_9.tif]

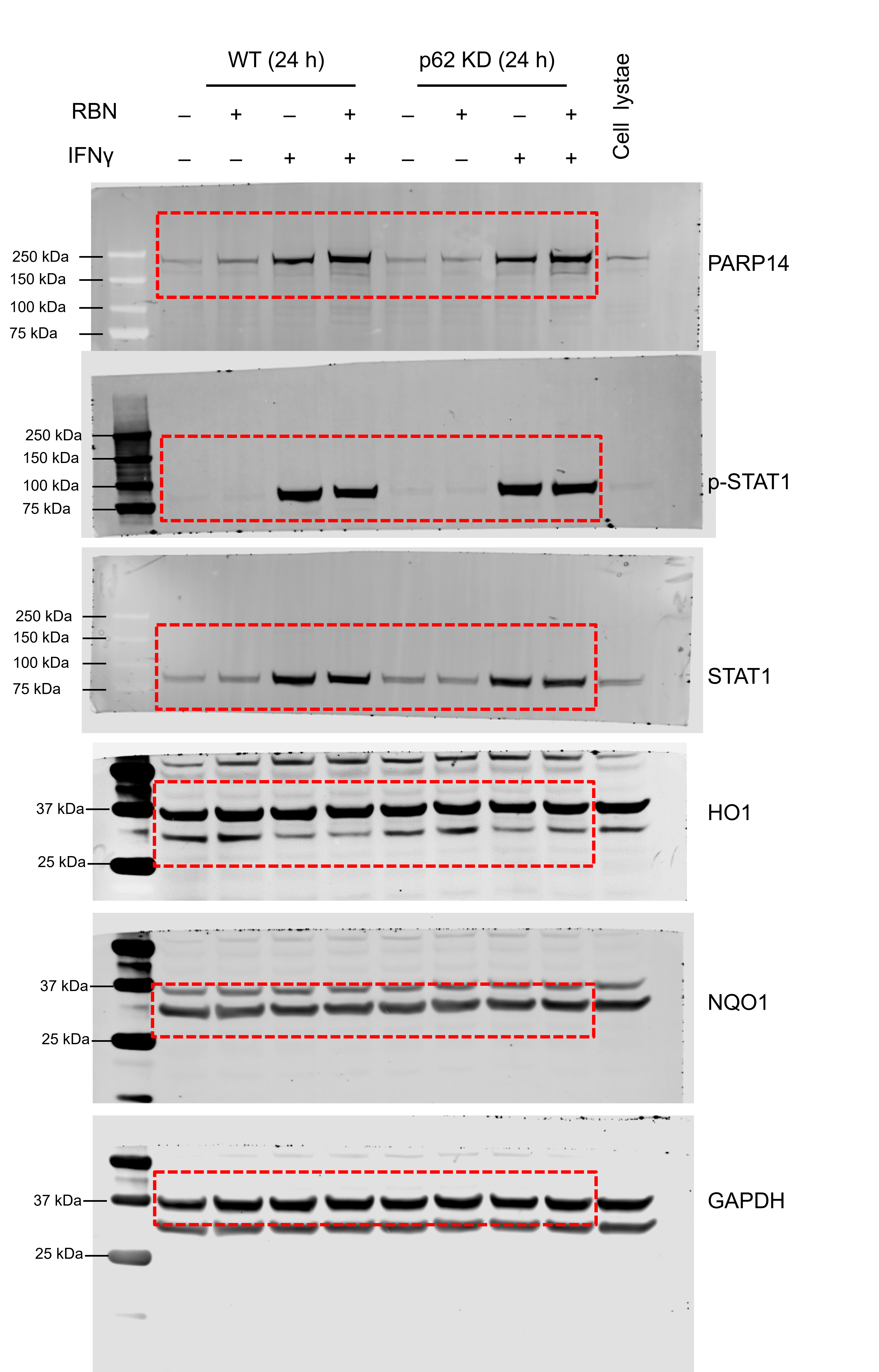

Supplement: Supplementary file 9 — Source data Fig. 5 [file 44318_2025_421_MOESM9_ESM.zip › Figure 5/Figure 5B.tif]

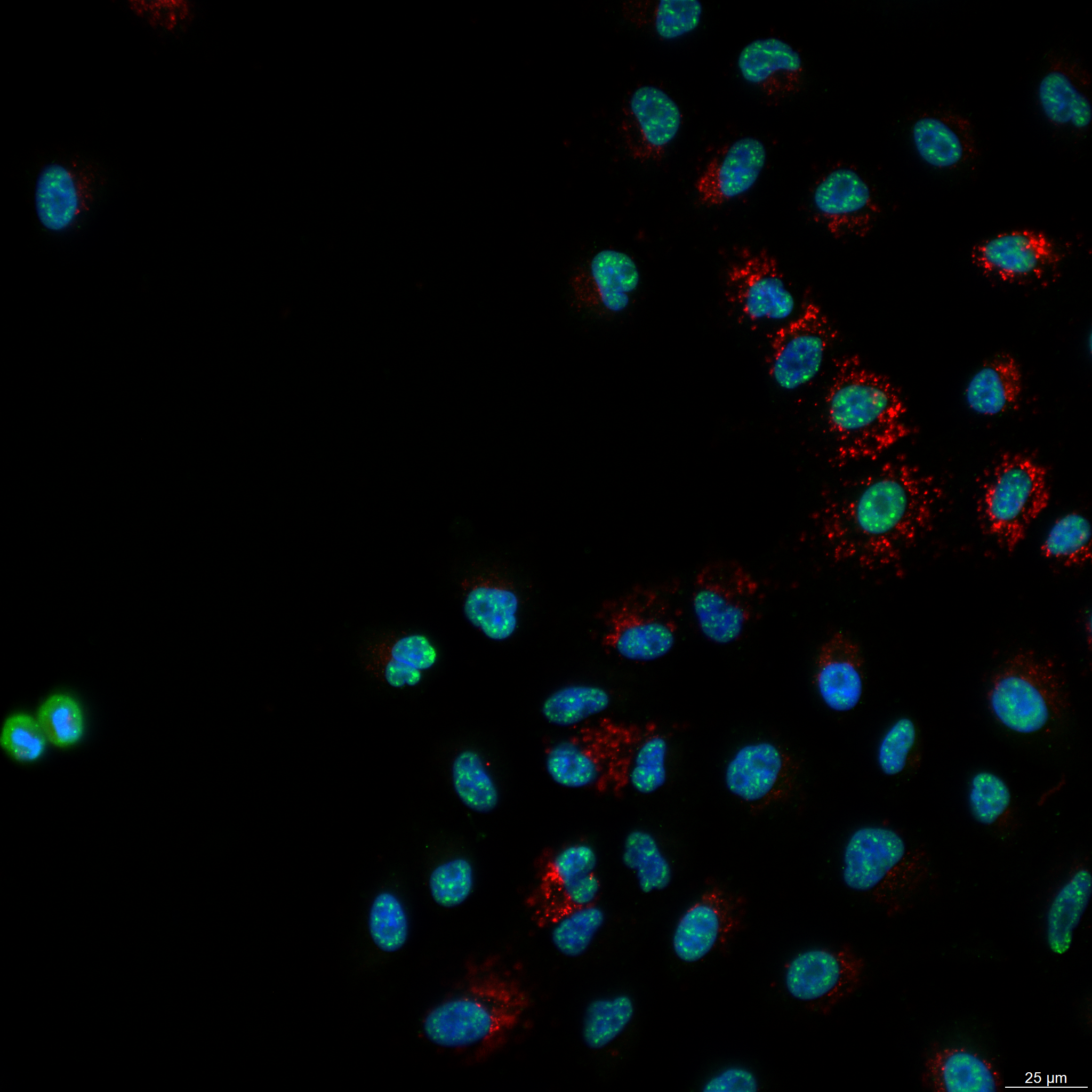

Supplement: Supplementary file 9 — Source data Fig. 5 [file 44318_2025_421_MOESM9_ESM.zip › Figure 5/Figure 5D/Control.tif]

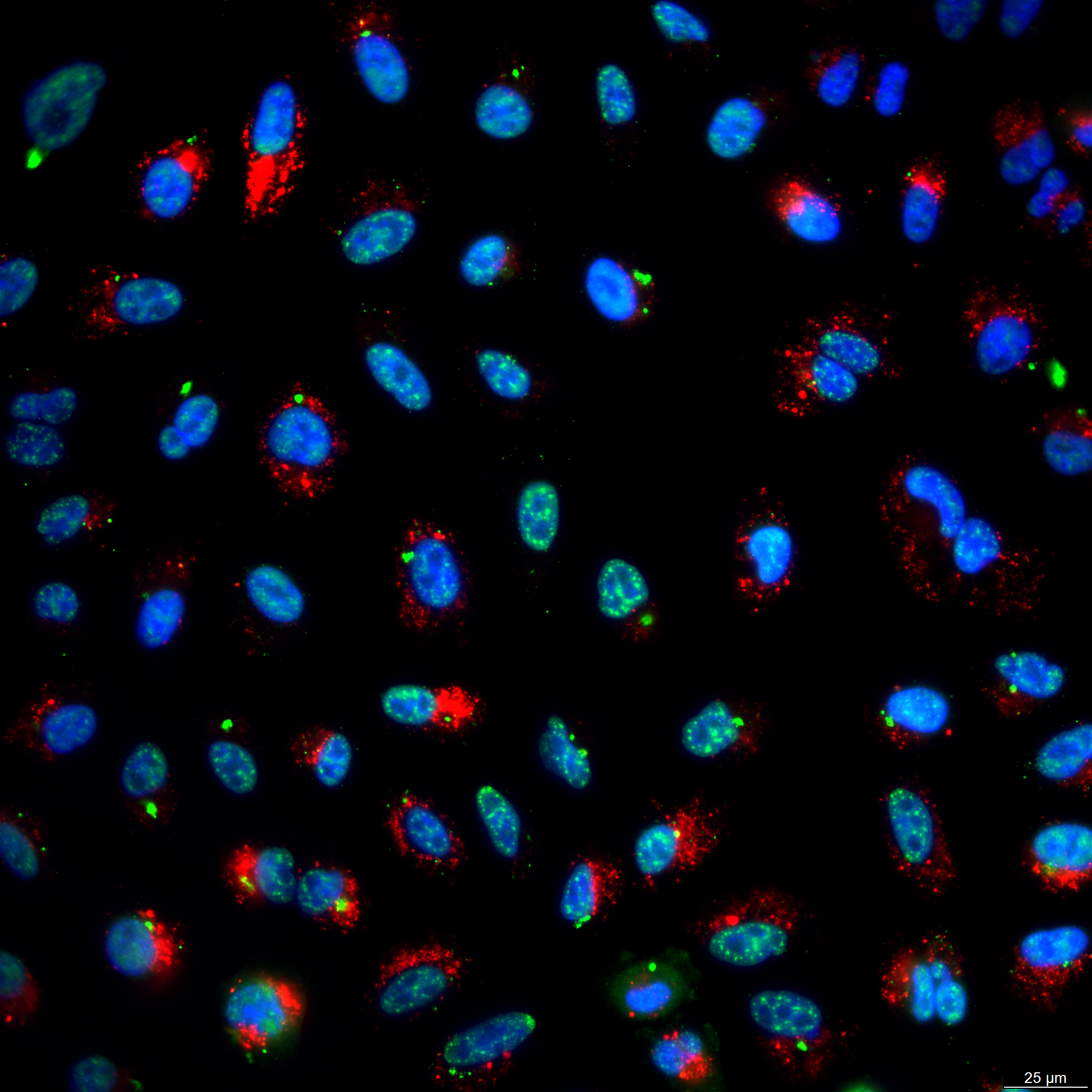

Supplement: Supplementary file 9 — Source data Fig. 5 [file 44318_2025_421_MOESM9_ESM.zip › Figure 5/Figure 5D/lFNγ.tif]

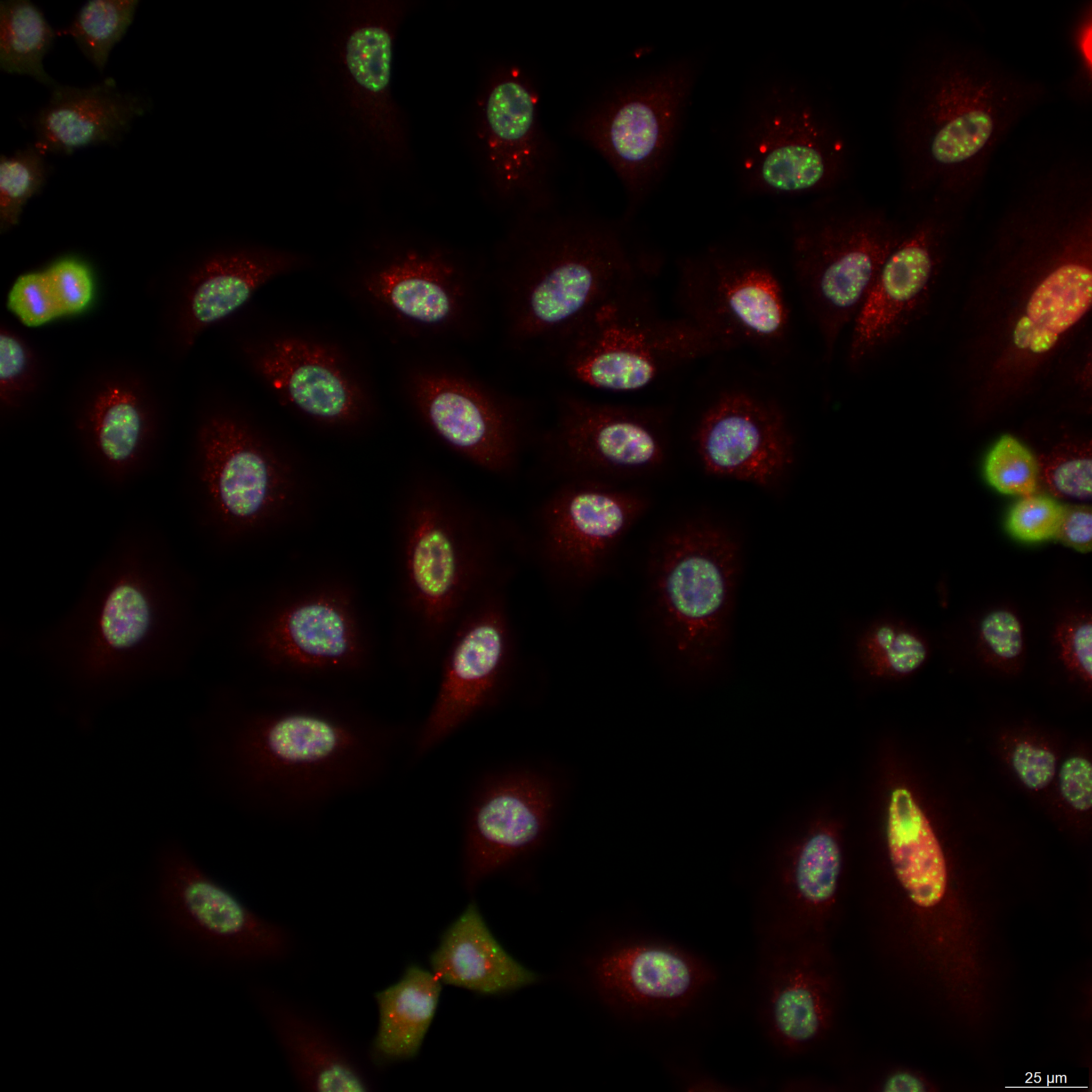

Supplement: Supplementary file 9 — Source data Fig. 5 [file 44318_2025_421_MOESM9_ESM.zip › Figure 5/Figure 5E/Control.tif]

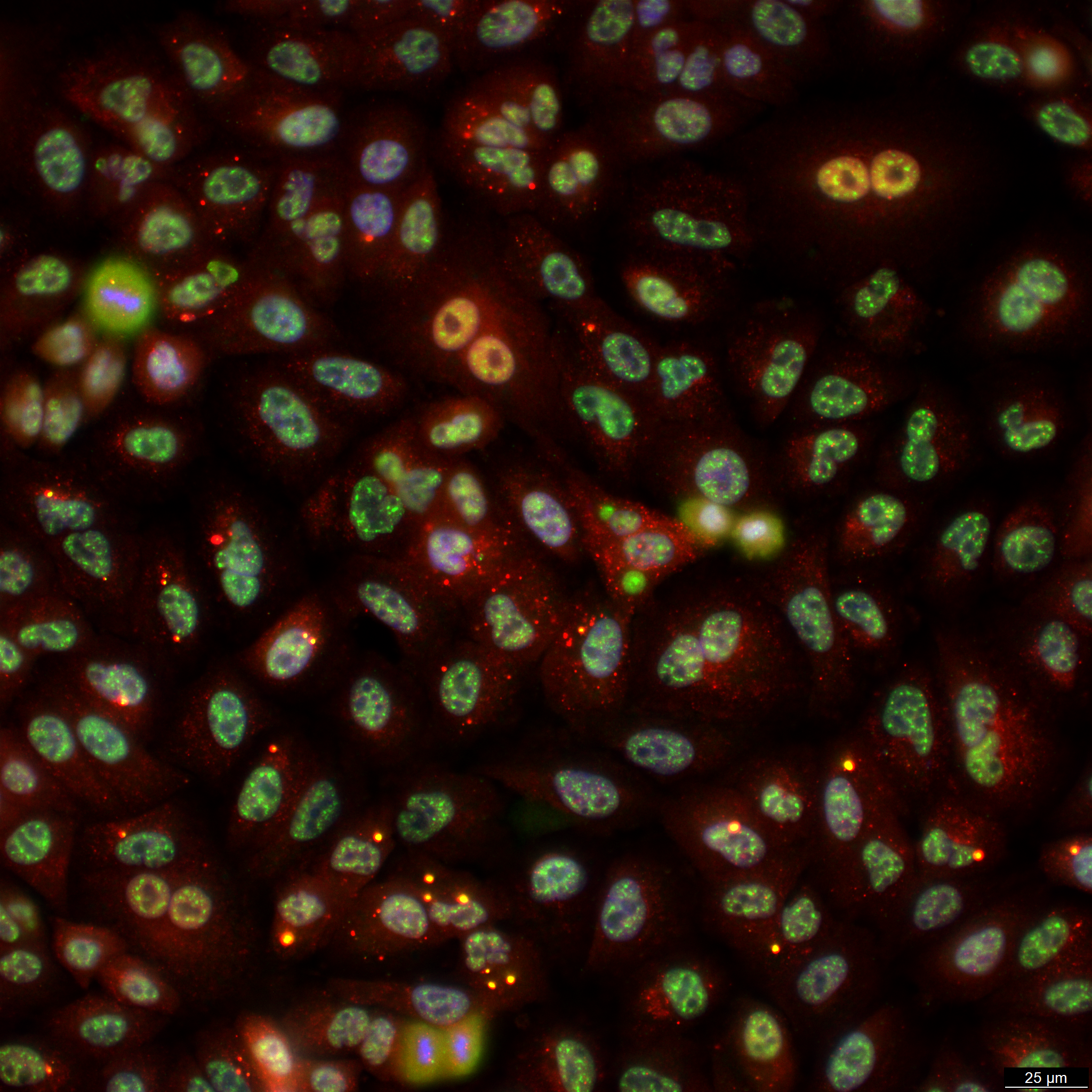

Supplement: Supplementary file 9 — Source data Fig. 5 [file 44318_2025_421_MOESM9_ESM.zip › Figure 5/Figure 5E/lFNγ.tif]

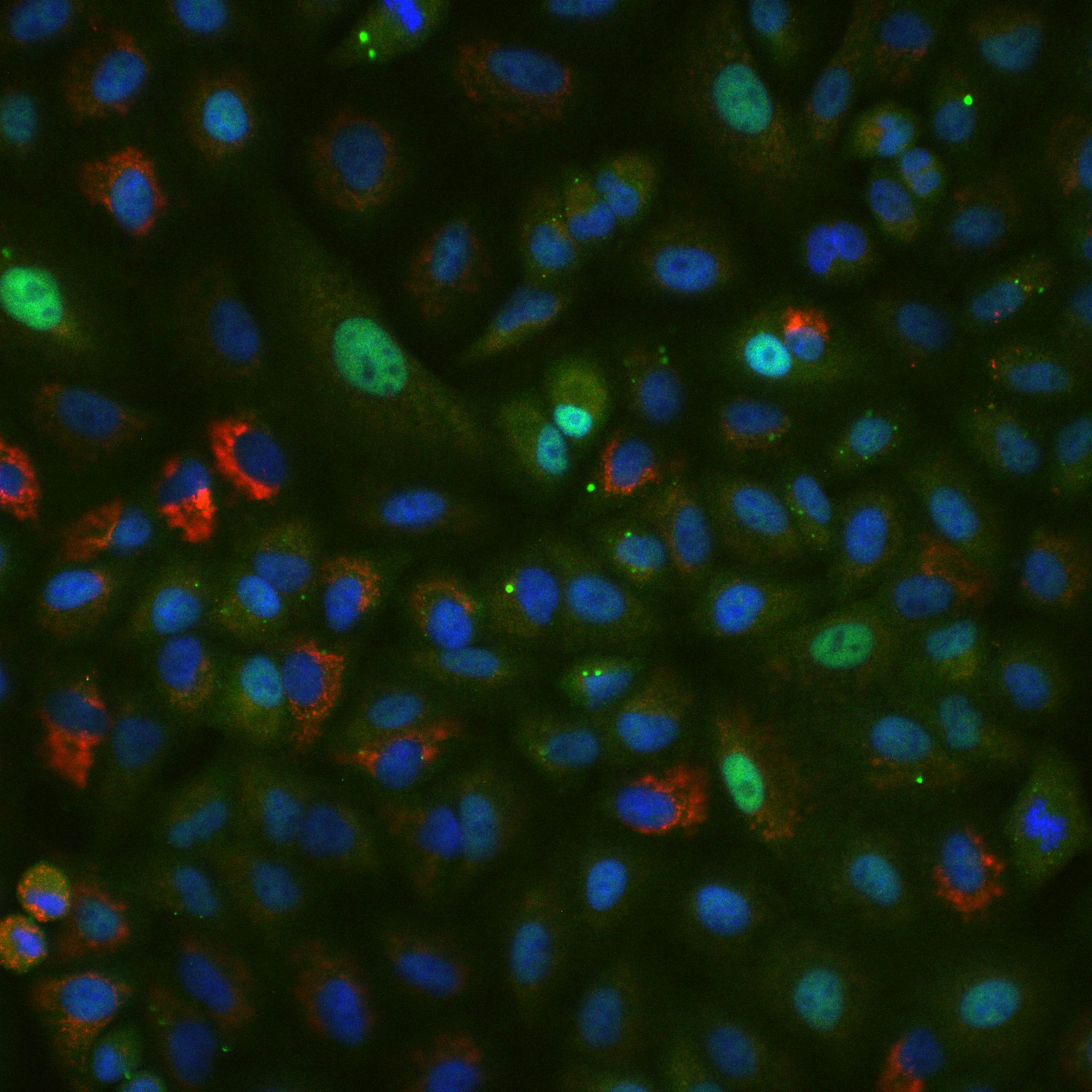

Supplement: Supplementary file 9 — Source data Fig. 5 [file 44318_2025_421_MOESM9_ESM.zip › Figure 5/Figure 5F/Control.tif]

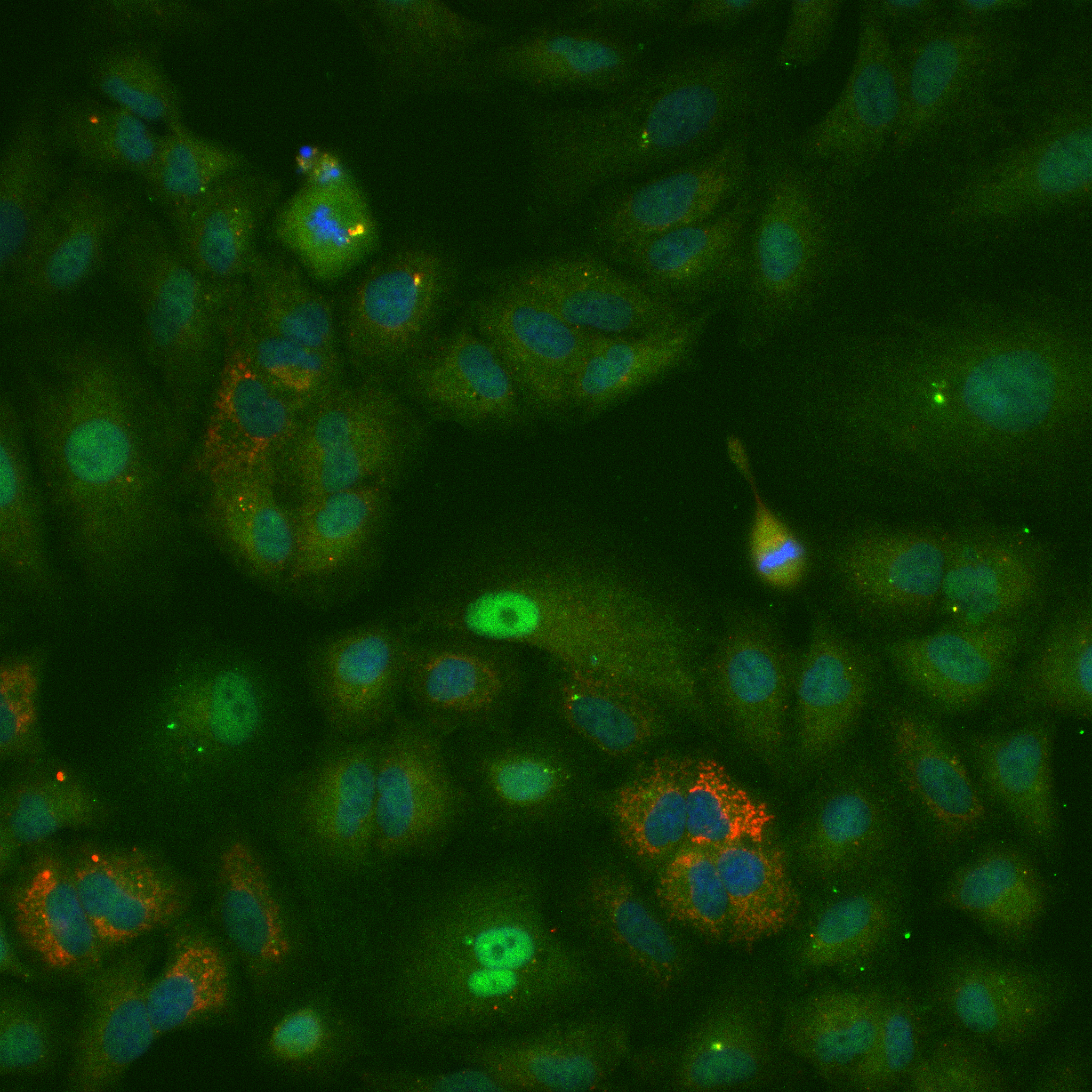

Supplement: Supplementary file 9 — Source data Fig. 5 [file 44318_2025_421_MOESM9_ESM.zip › Figure 5/Figure 5F/IFNγ.tif]

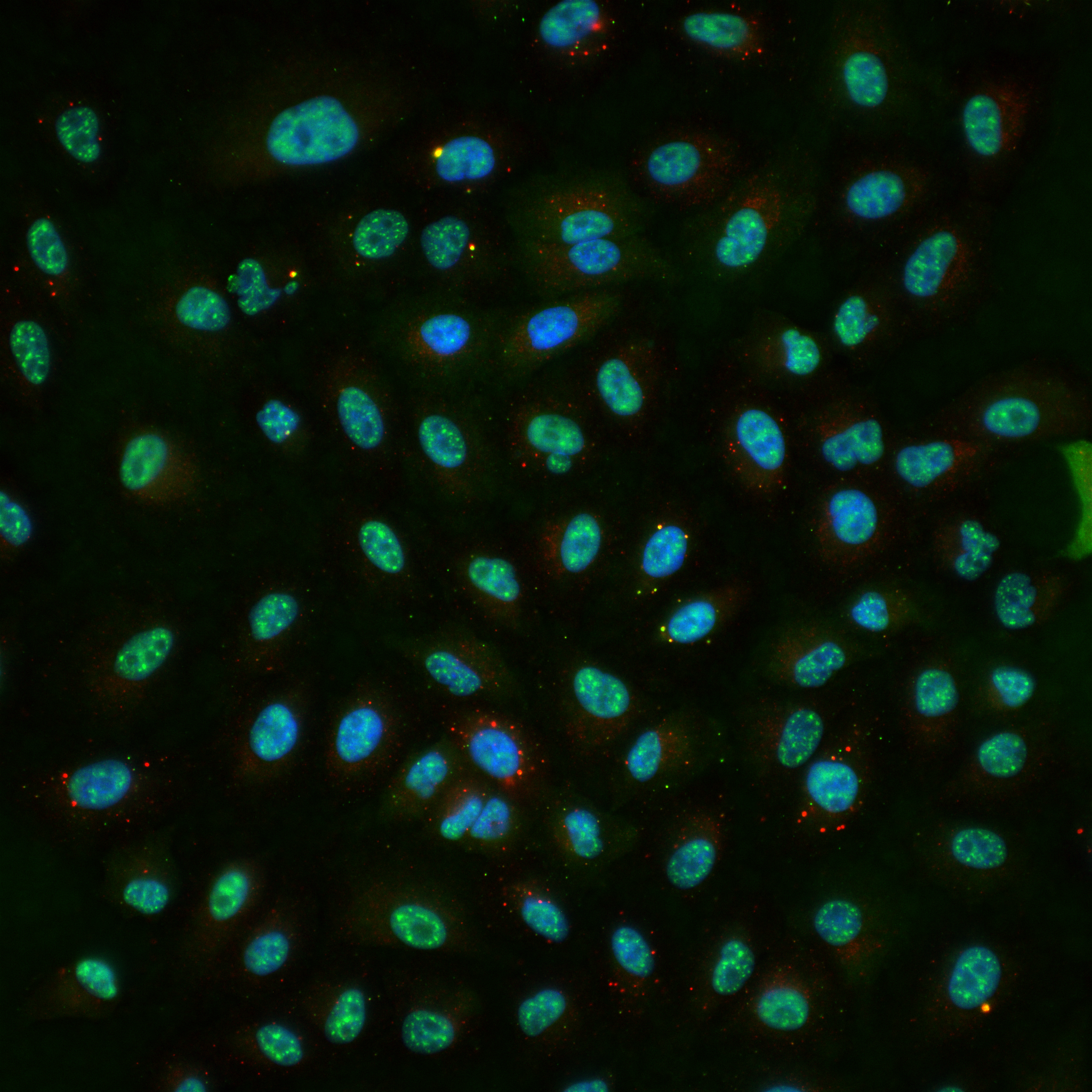

Supplement: Supplementary file 9 — Source data Fig. 5 [file 44318_2025_421_MOESM9_ESM.zip › Figure 5/Figure 5G/Control.tif]

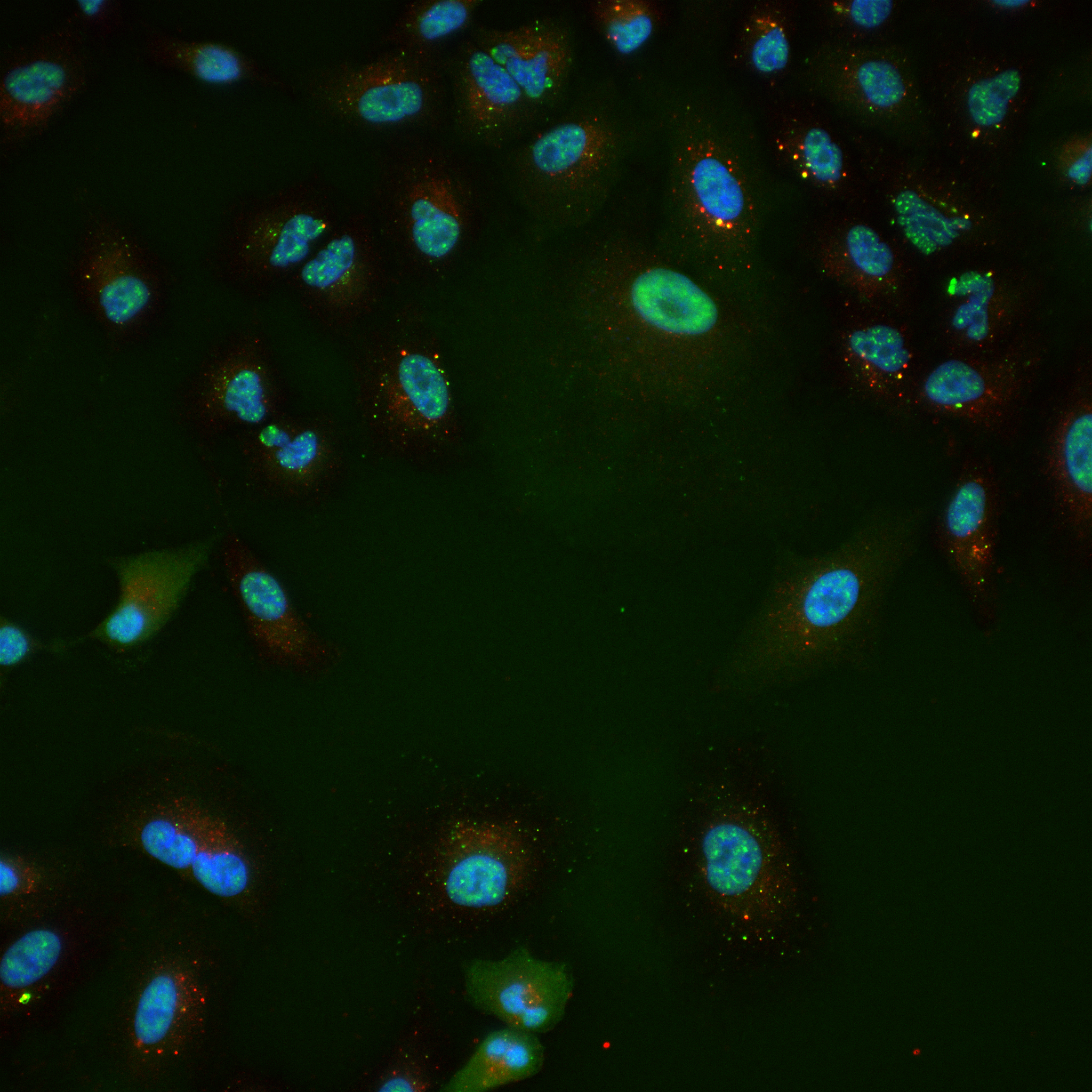

Supplement: Supplementary file 9 — Source data Fig. 5 [file 44318_2025_421_MOESM9_ESM.zip › Figure 5/Figure 5G/IFNγ.tif]

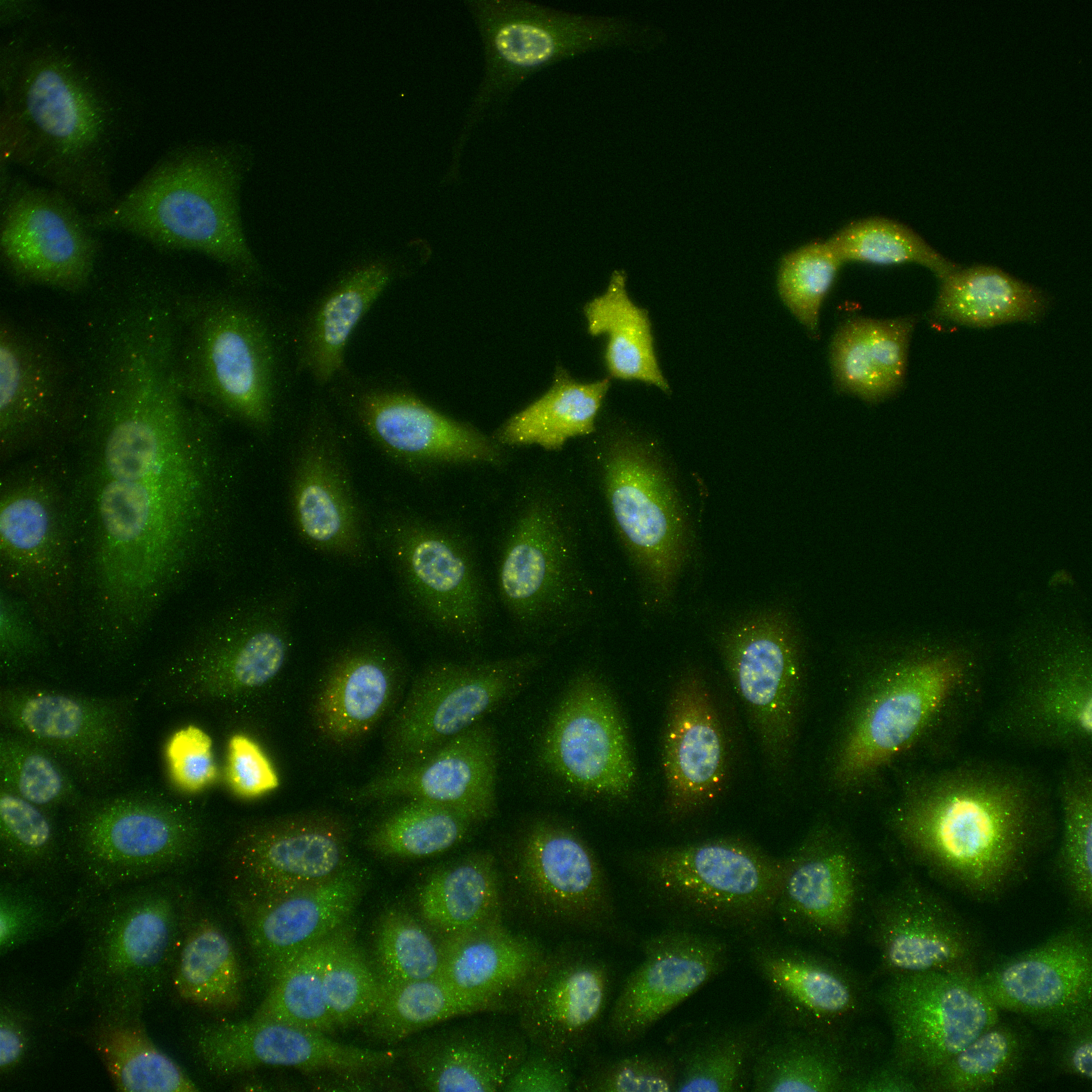

Supplement: Supplementary file 9 — Source data Fig. 5 [file 44318_2025_421_MOESM9_ESM.zip › Figure 5/Figure 5H/Control.tif]

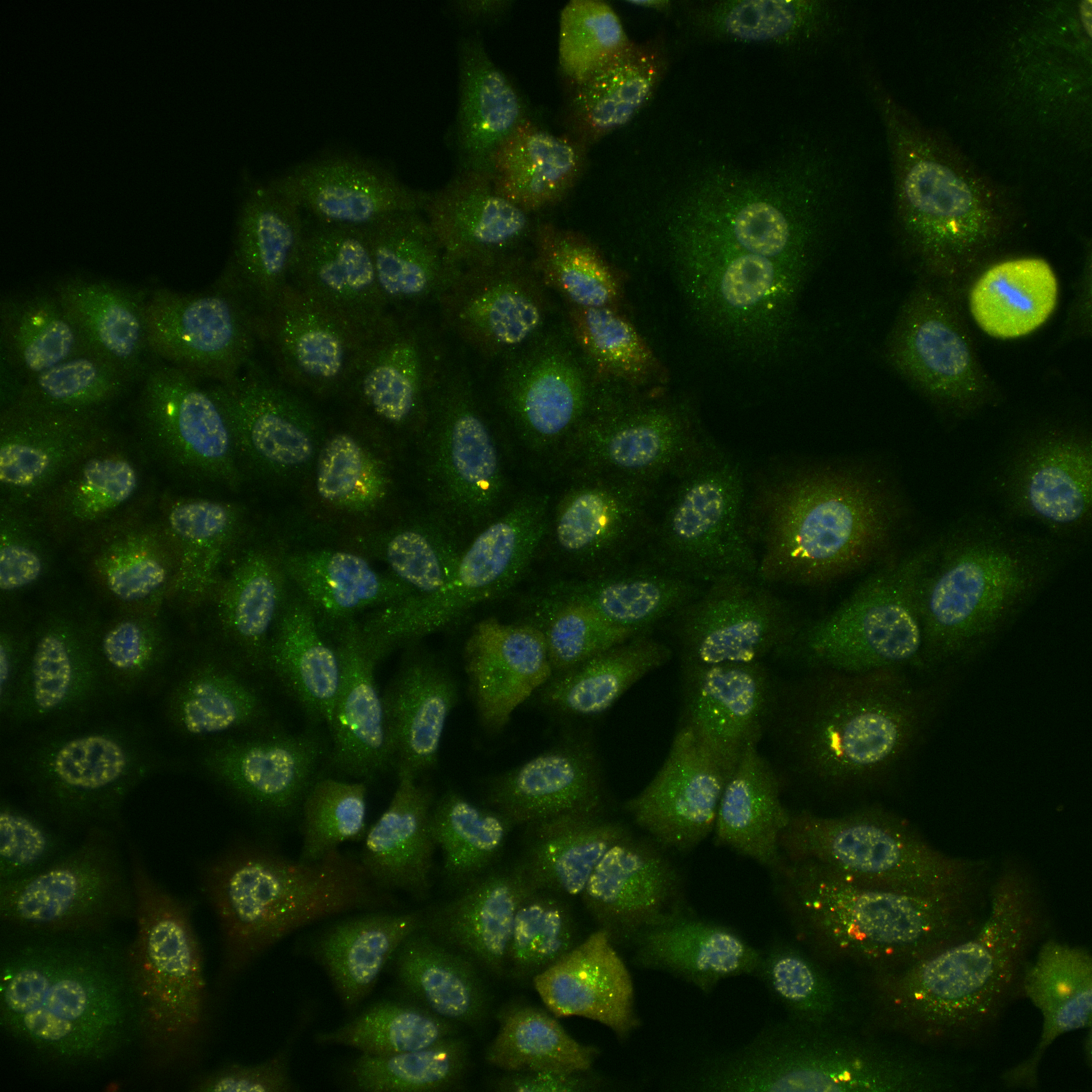

Supplement: Supplementary file 9 — Source data Fig. 5 [file 44318_2025_421_MOESM9_ESM.zip › Figure 5/Figure 5H/IFNγ.tif]

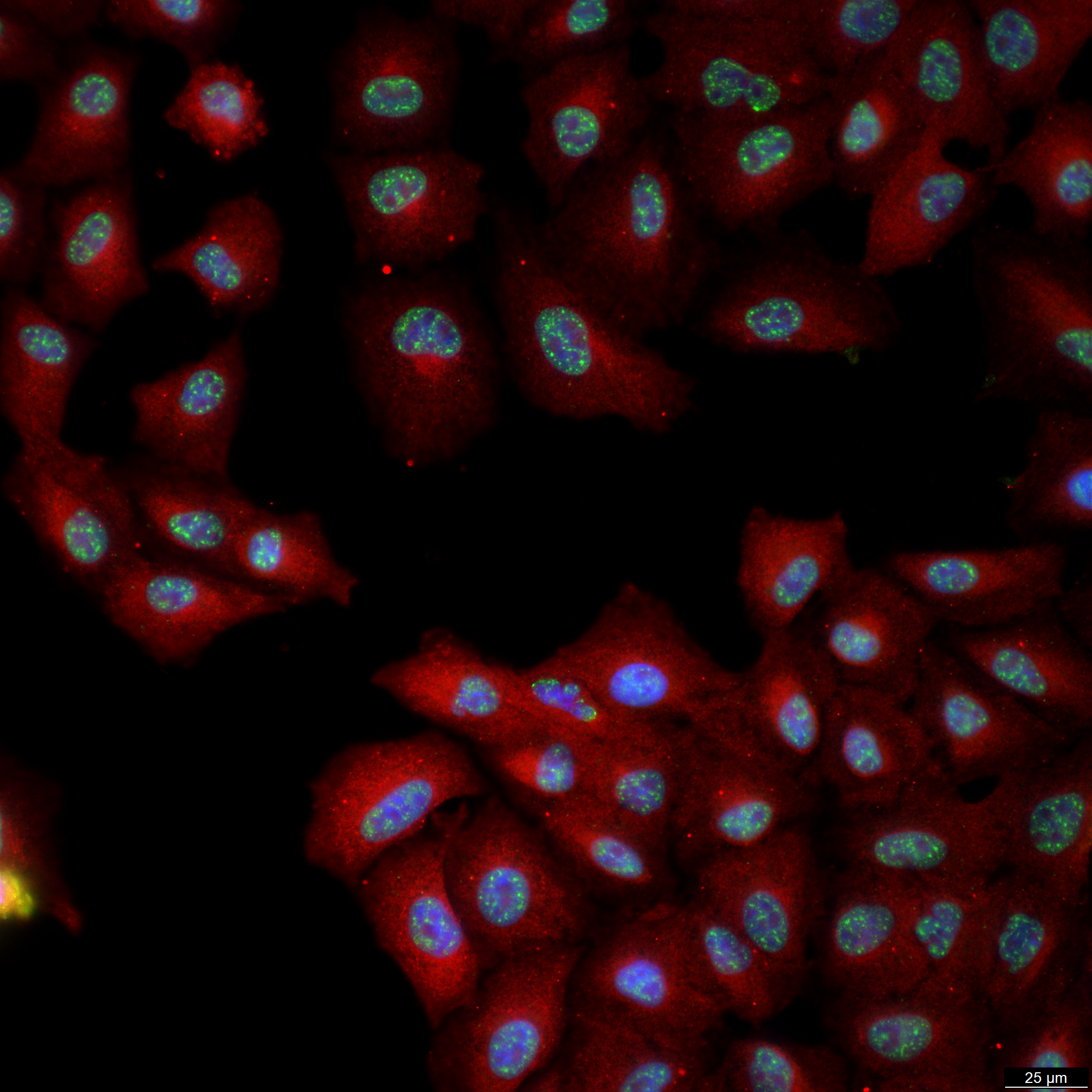

Supplement: Supplementary file 9 — Source data Fig. 5 [file 44318_2025_421_MOESM9_ESM.zip › Figure 5/Figure 5I/Control .tif]

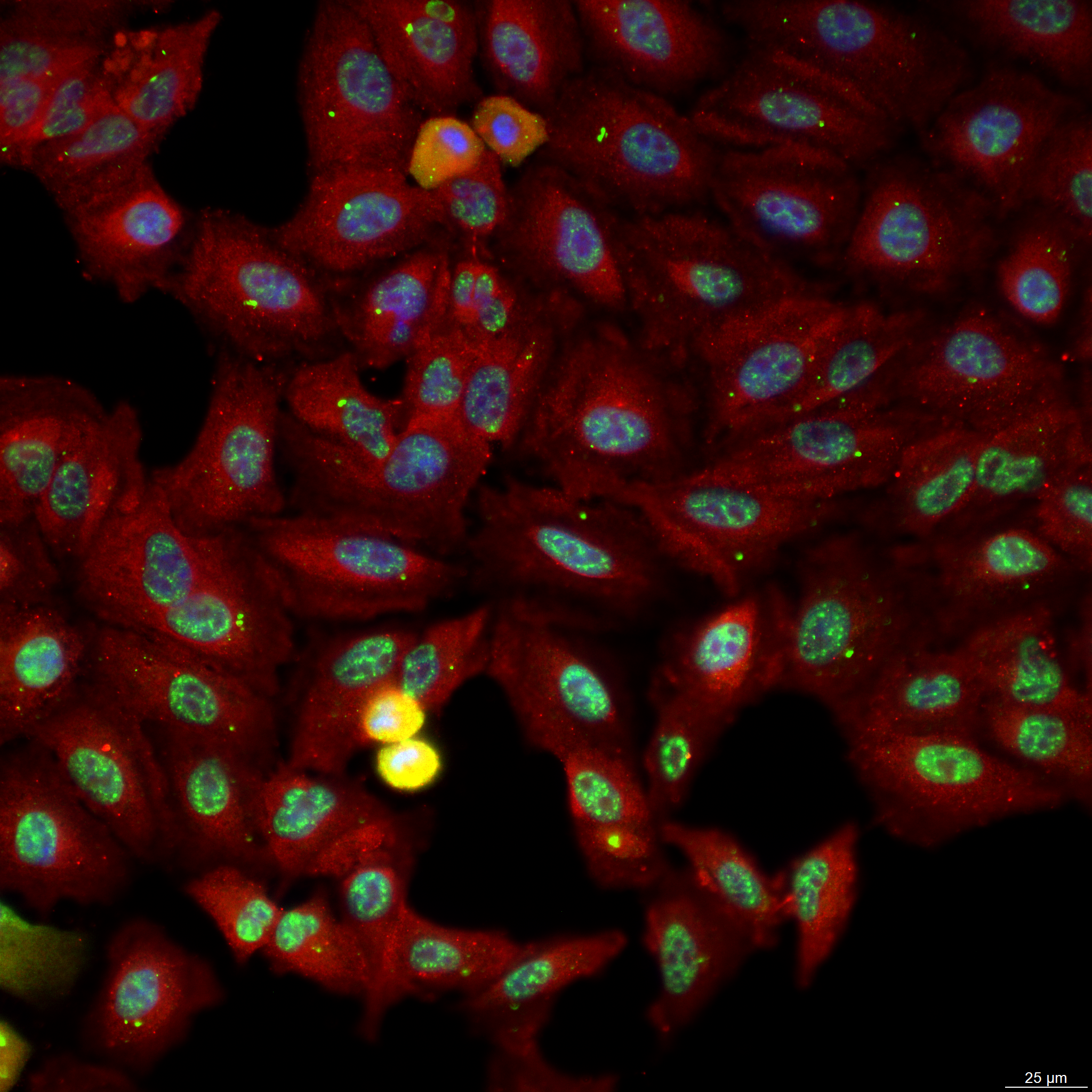

Supplement: Supplementary file 9 — Source data Fig. 5 [file 44318_2025_421_MOESM9_ESM.zip › Figure 5/Figure 5I/lFNγ.tif]

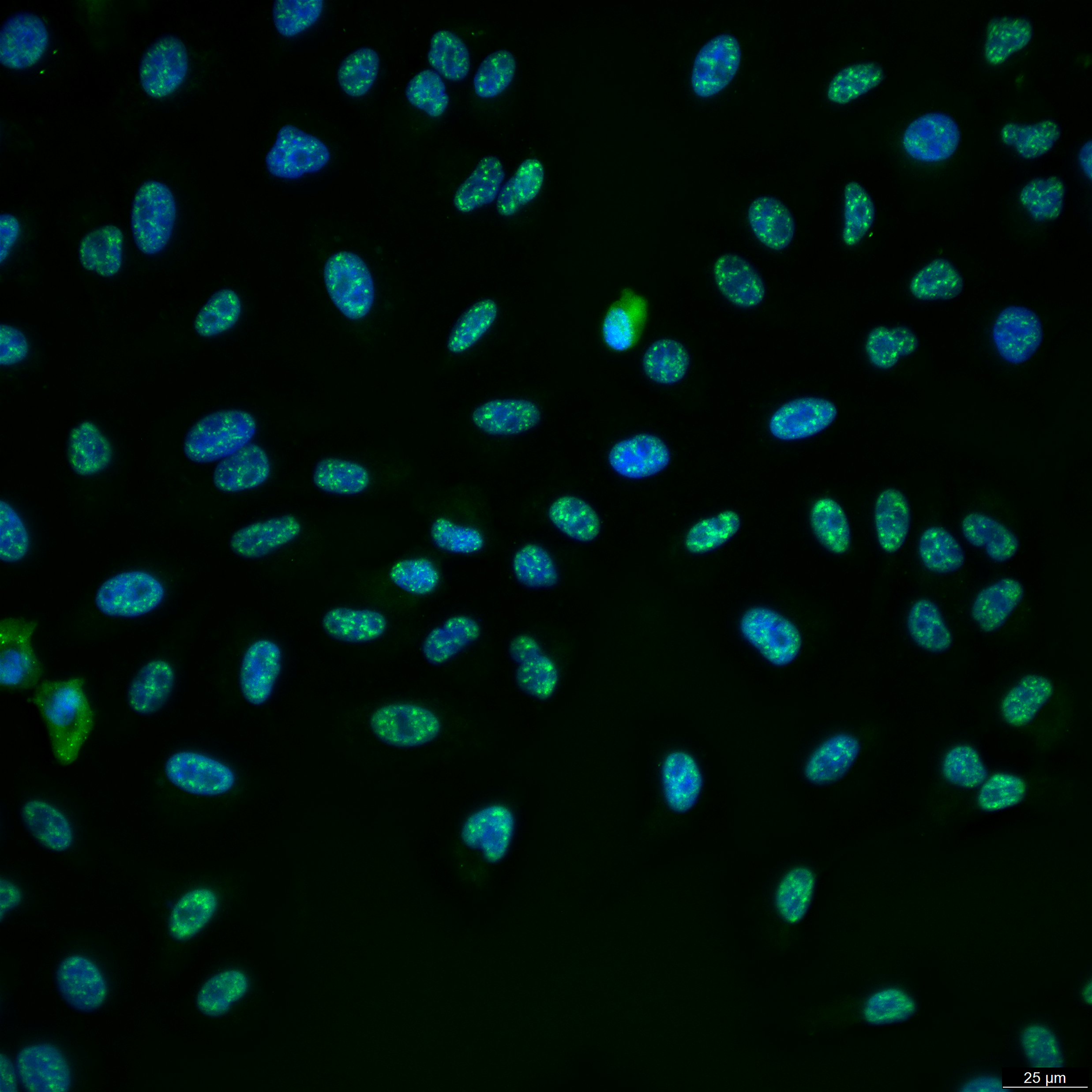

Supplement: Supplementary file 10 — Source data Fig. 6 [file 44318_2025_421_MOESM10_ESM.zip › Figure 6/Figure 6A/Control.tif]

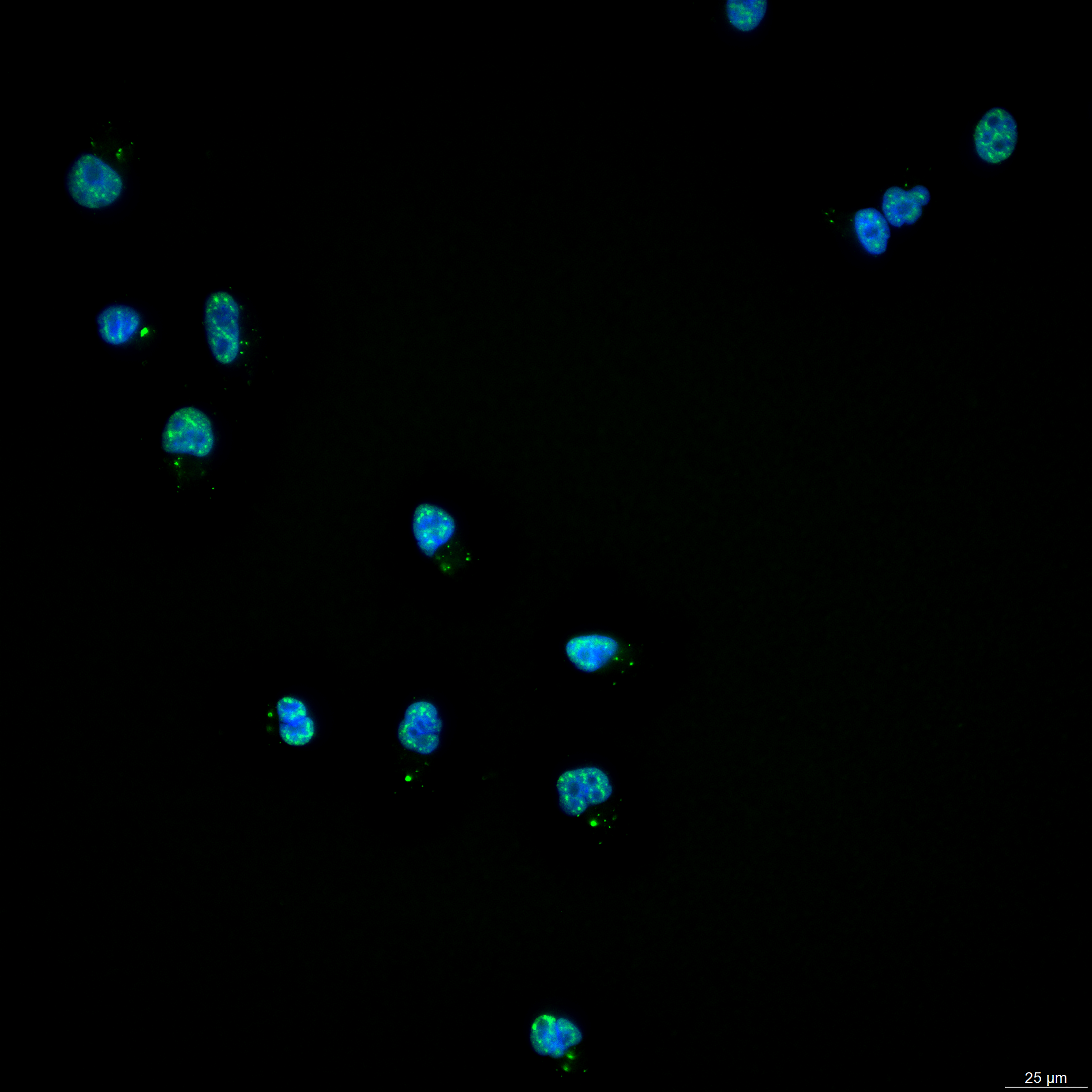

Supplement: Supplementary file 10 — Source data Fig. 6 [file 44318_2025_421_MOESM10_ESM.zip › Figure 6/Figure 6A/lFNγ+Baf A1.tif]

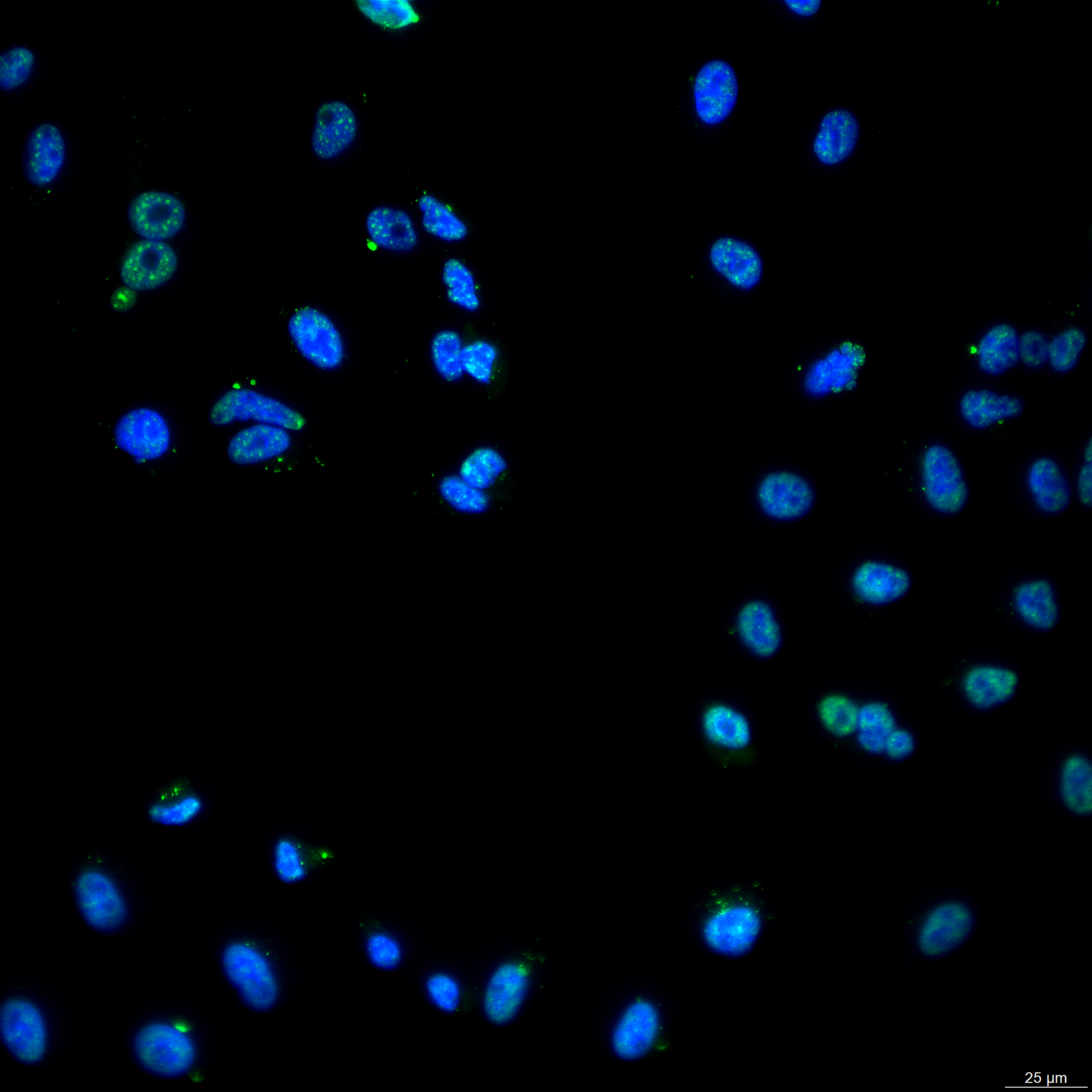

Supplement: Supplementary file 10 — Source data Fig. 6 [file 44318_2025_421_MOESM10_ESM.zip › Figure 6/Figure 6A/lFNγ+CQ.tif]

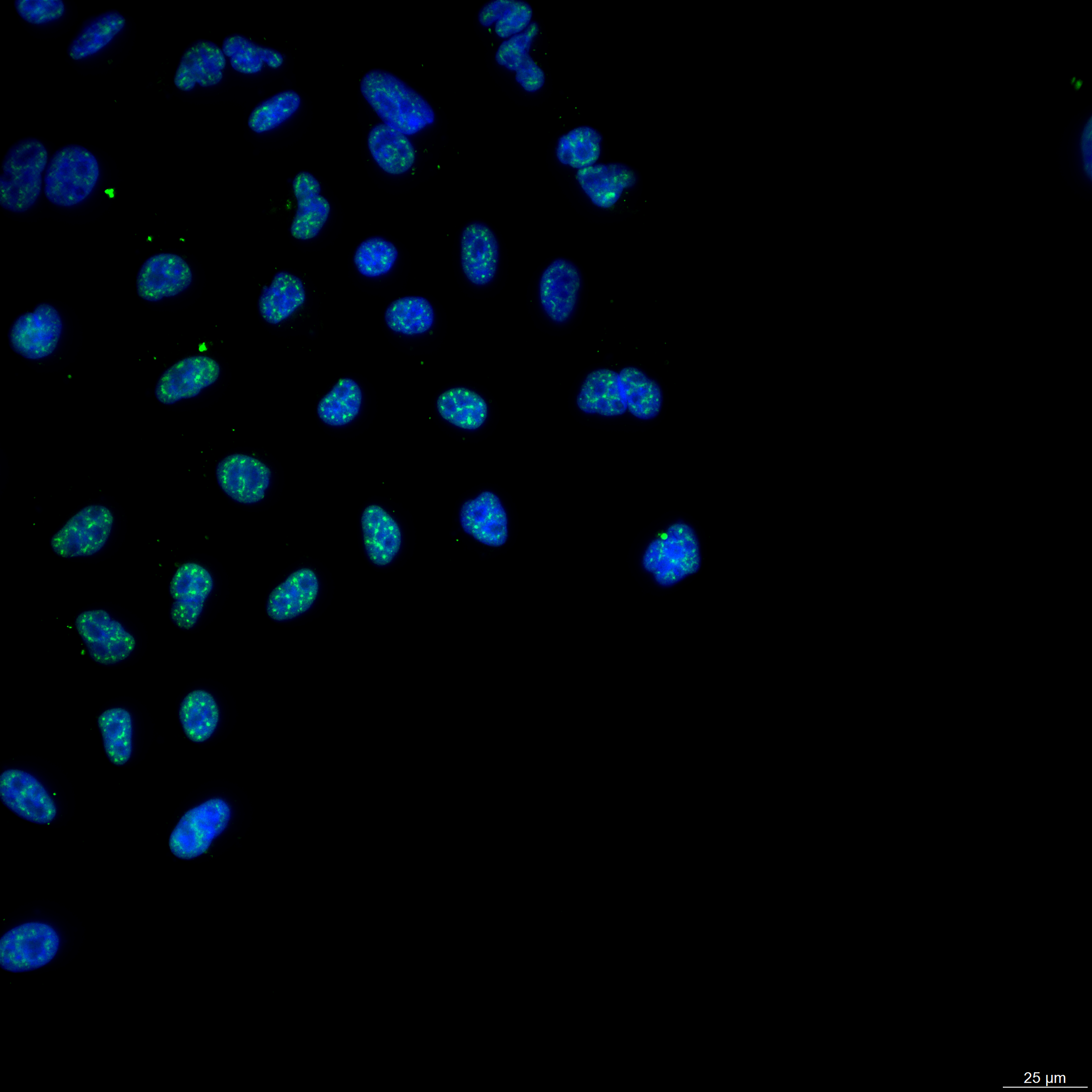

Supplement: Supplementary file 10 — Source data Fig. 6 [file 44318_2025_421_MOESM10_ESM.zip › Figure 6/Figure 6A/lFNγ.tif]

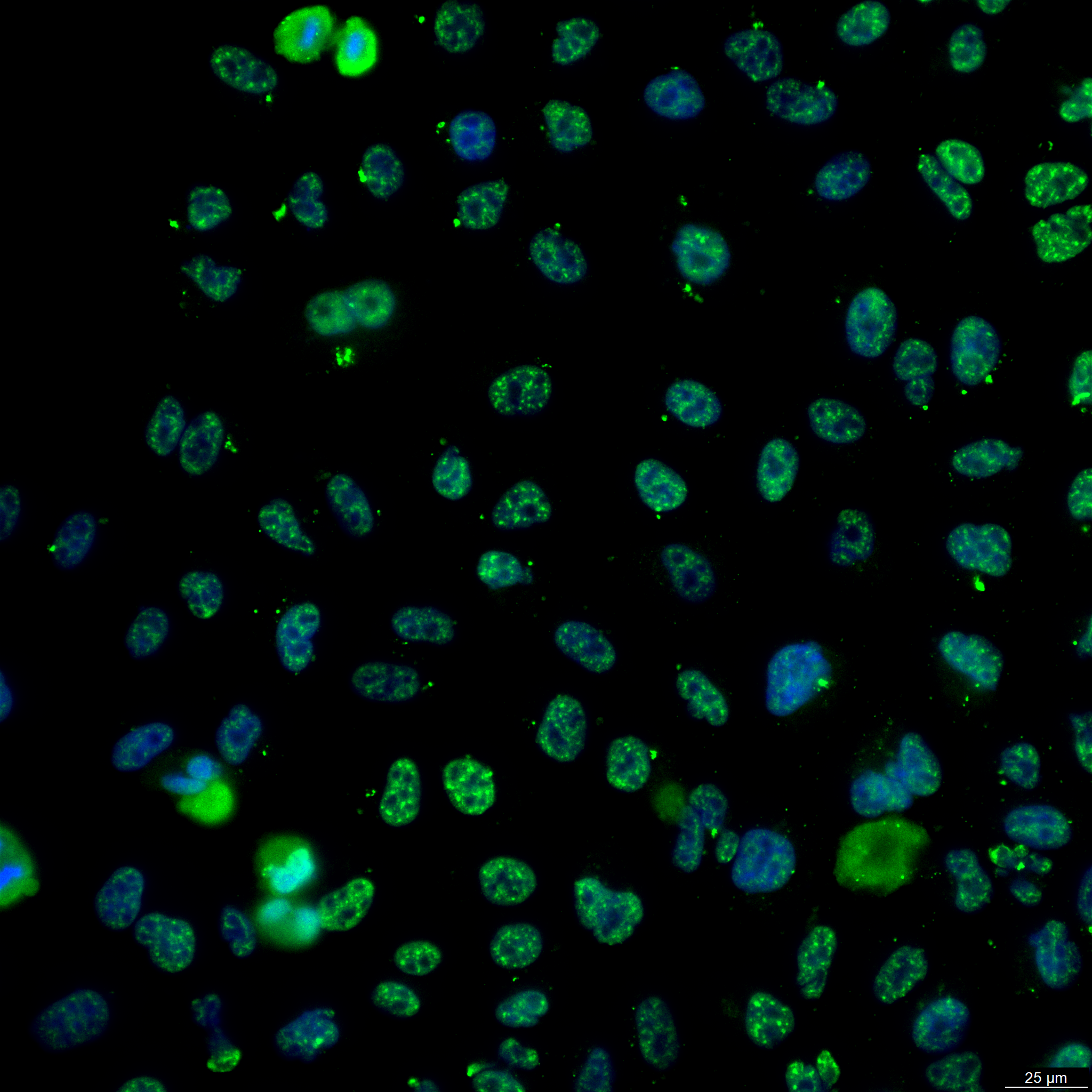

Supplement: Supplementary file 10 — Source data Fig. 6 [file 44318_2025_421_MOESM10_ESM.zip › Figure 6/Figure 6B/ATG5 KO +lFNγ.tif]

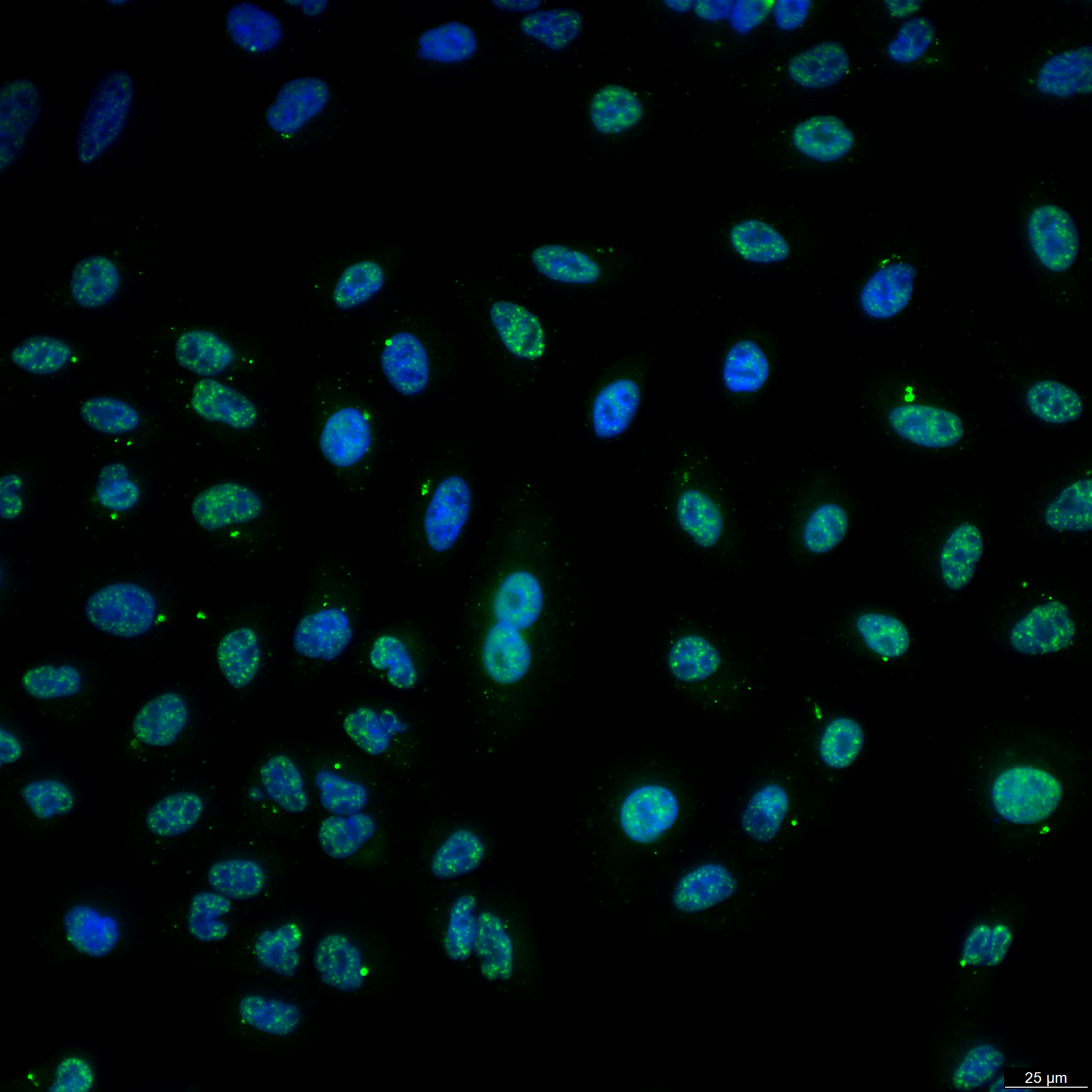

Supplement: Supplementary file 10 — Source data Fig. 6 [file 44318_2025_421_MOESM10_ESM.zip › Figure 6/Figure 6B/WT+lFNγ.tif]

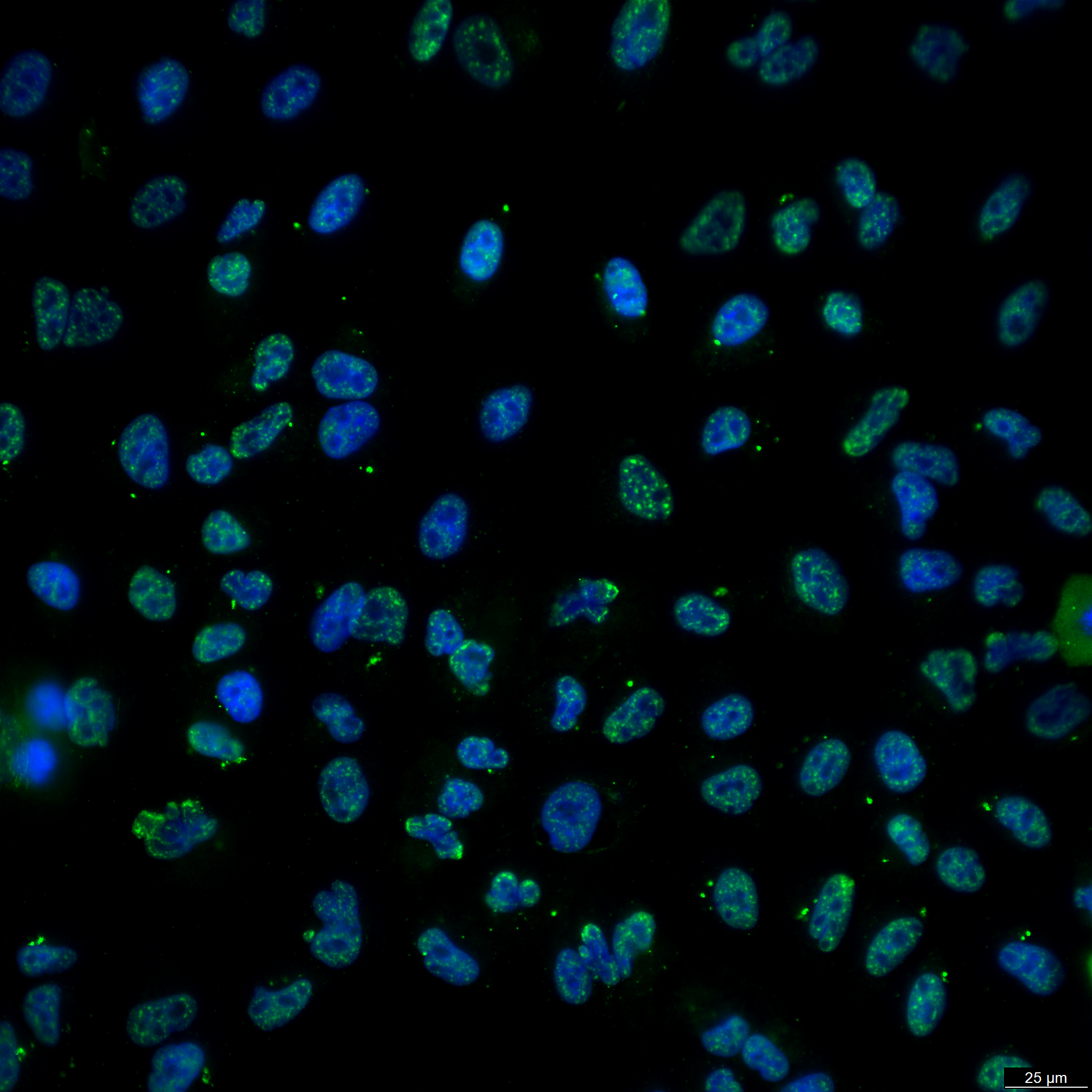

Supplement: Supplementary file 10 — Source data Fig. 6 [file 44318_2025_421_MOESM10_ESM.zip › Figure 6/Figure 6C/Beclin KO +lFNγ.tif]

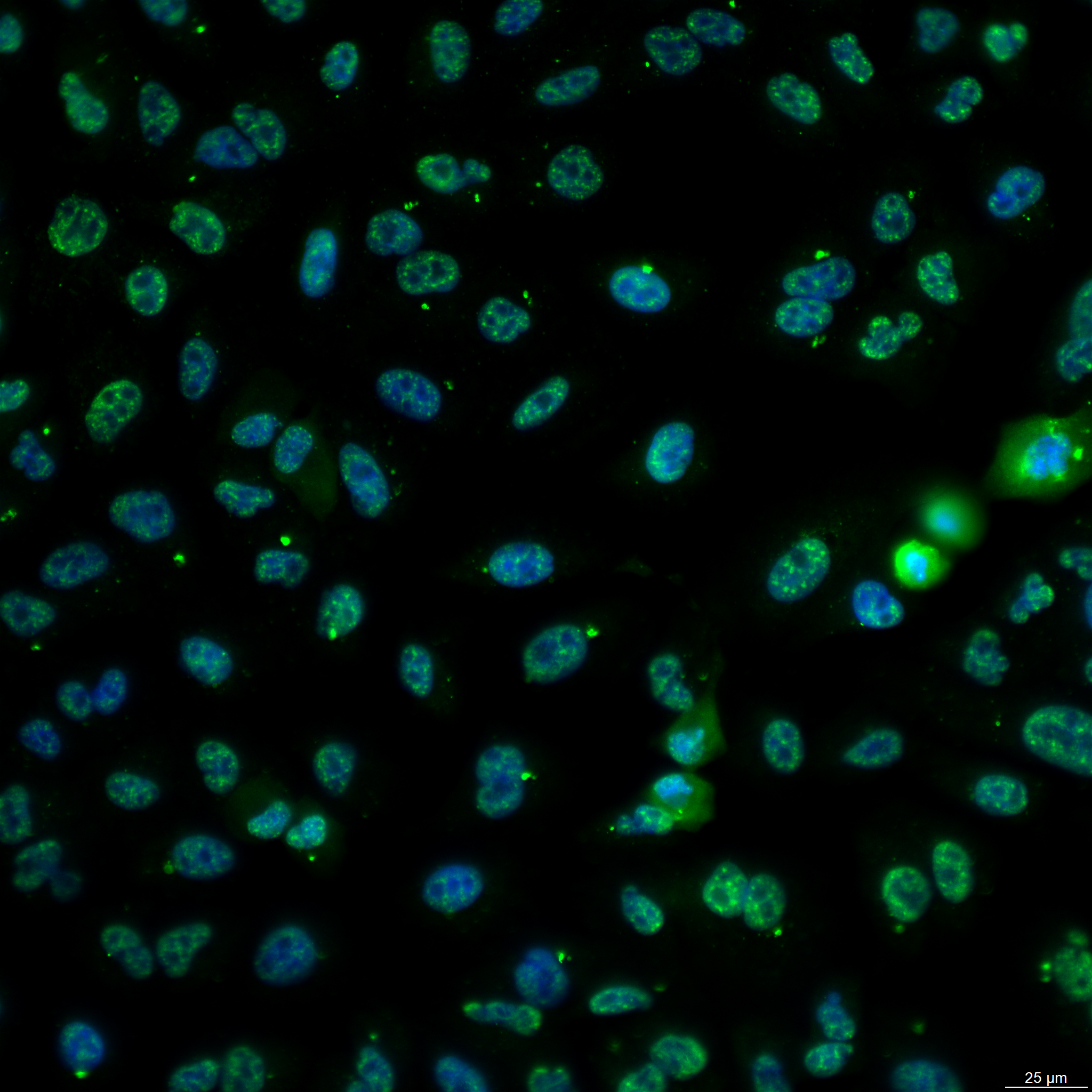

Supplement: Supplementary file 10 — Source data Fig. 6 [file 44318_2025_421_MOESM10_ESM.zip › Figure 6/Figure 6C/WT+lFNγ.tif]

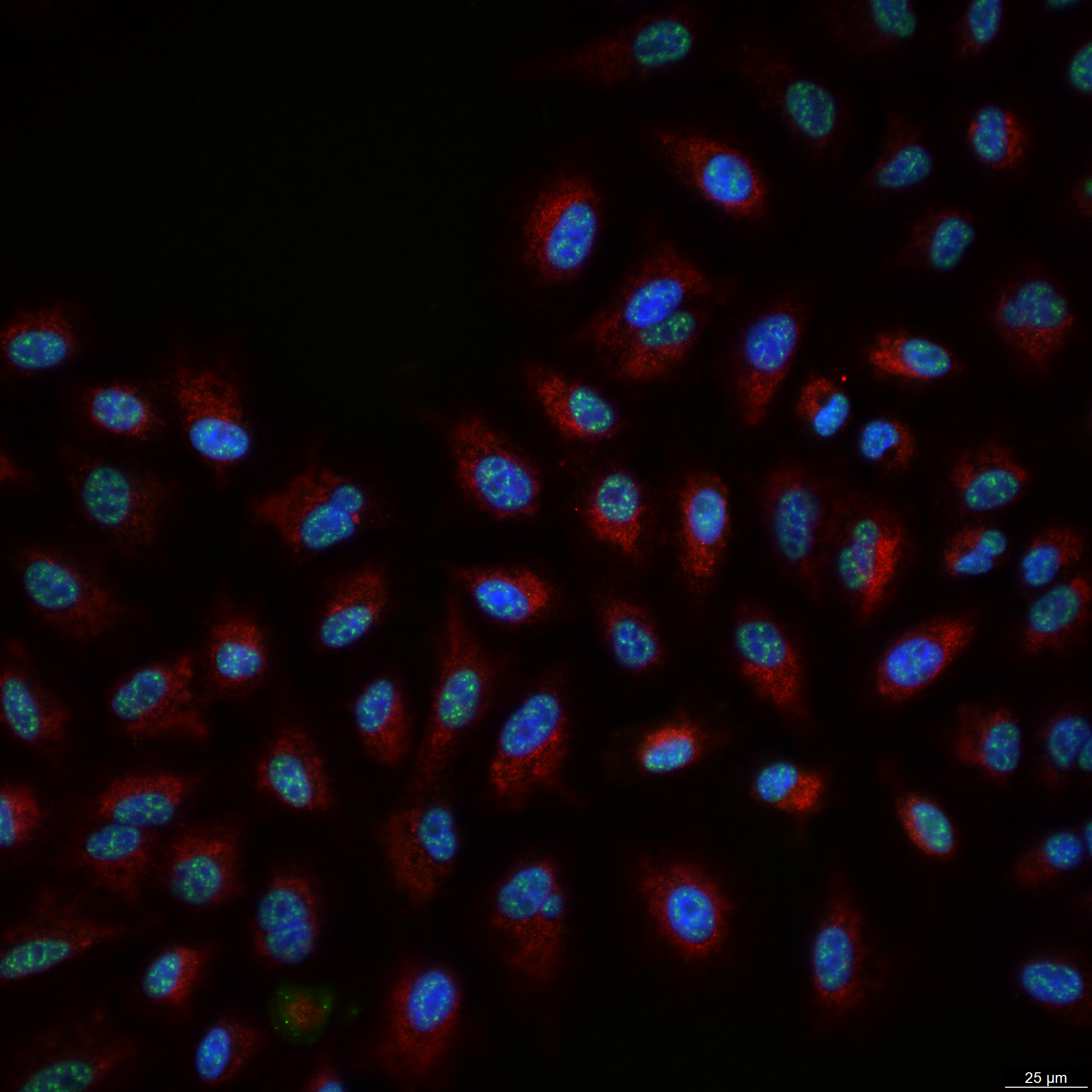

Supplement: Supplementary file 10 — Source data Fig. 6 [file 44318_2025_421_MOESM10_ESM.zip › Figure 6/Figure 6D/Control.tif]

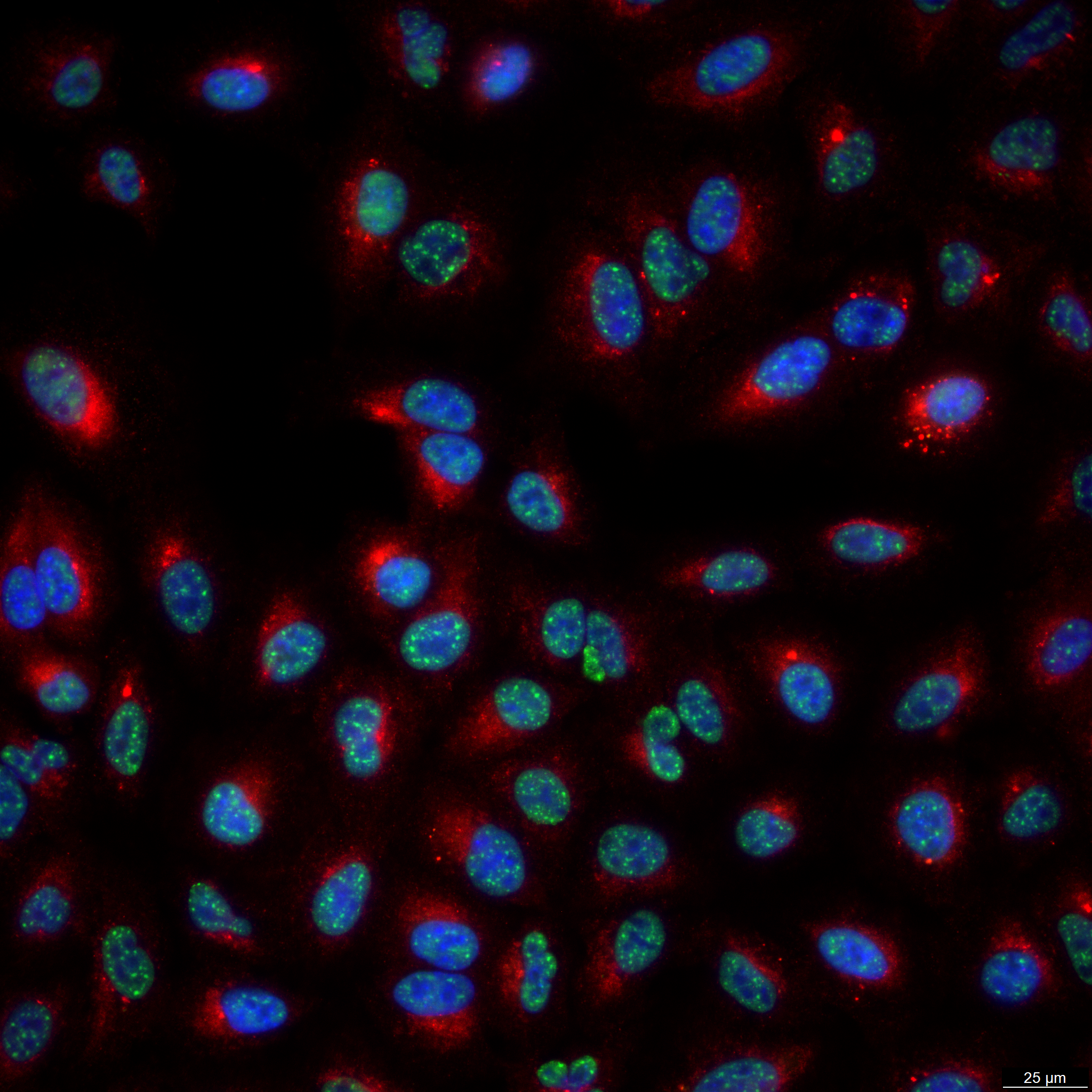

Supplement: Supplementary file 10 — Source data Fig. 6 [file 44318_2025_421_MOESM10_ESM.zip › Figure 6/Figure 6D/lFNγ+Epox.tif]

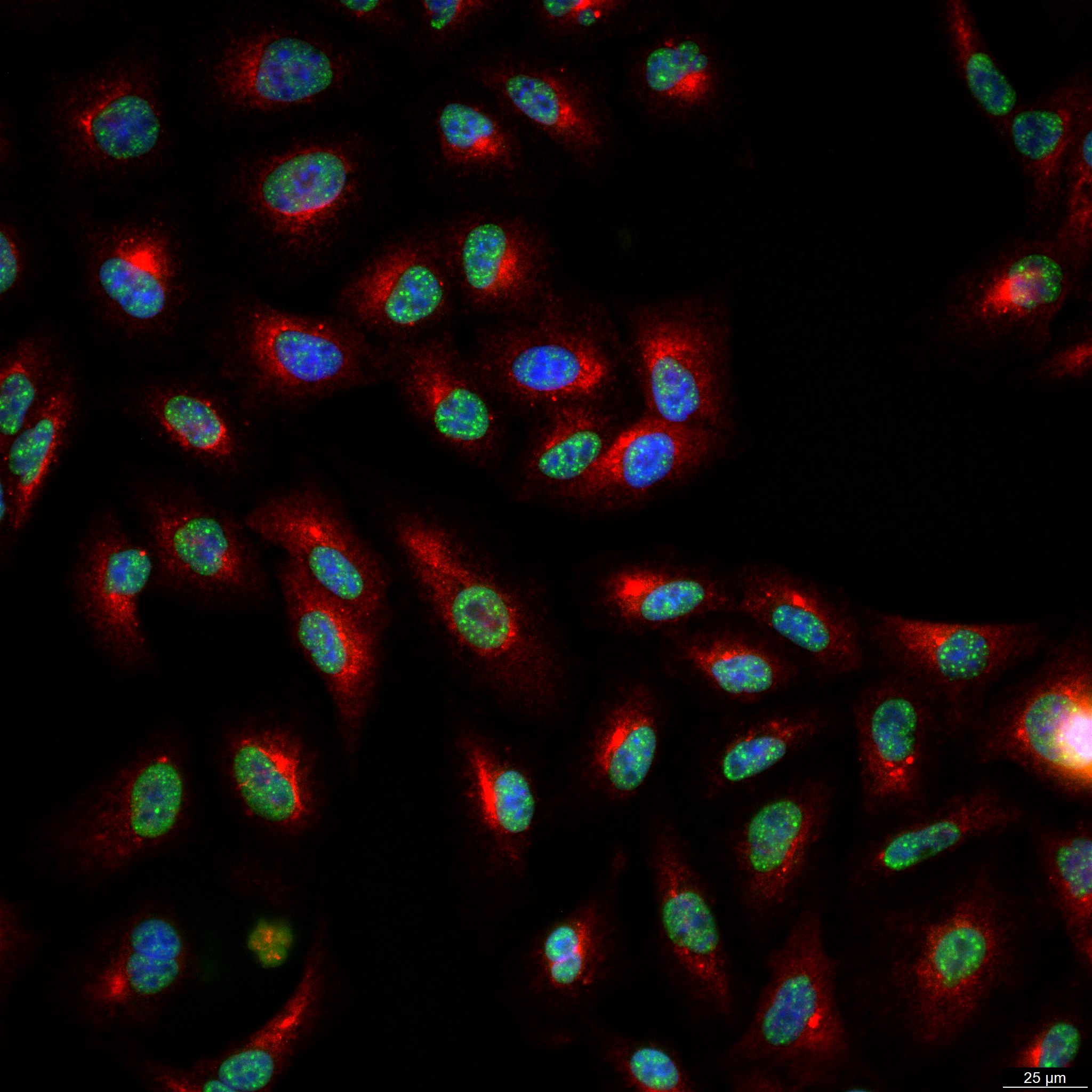

Supplement: Supplementary file 10 — Source data Fig. 6 [file 44318_2025_421_MOESM10_ESM.zip › Figure 6/Figure 6D/lFNγ+MG132.tif]

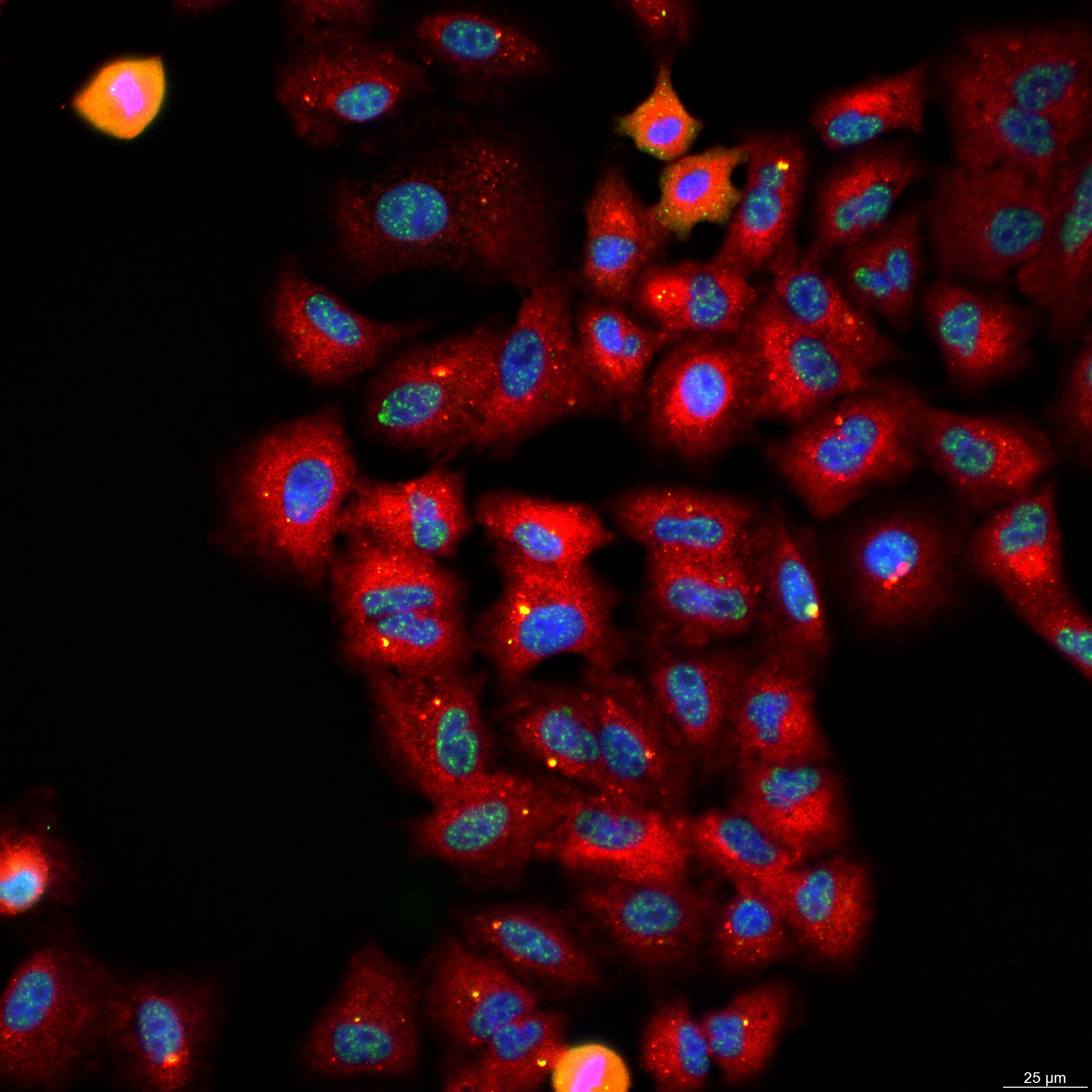

Supplement: Supplementary file 10 — Source data Fig. 6 [file 44318_2025_421_MOESM10_ESM.zip › Figure 6/Figure 6D/lFNγ.tif]

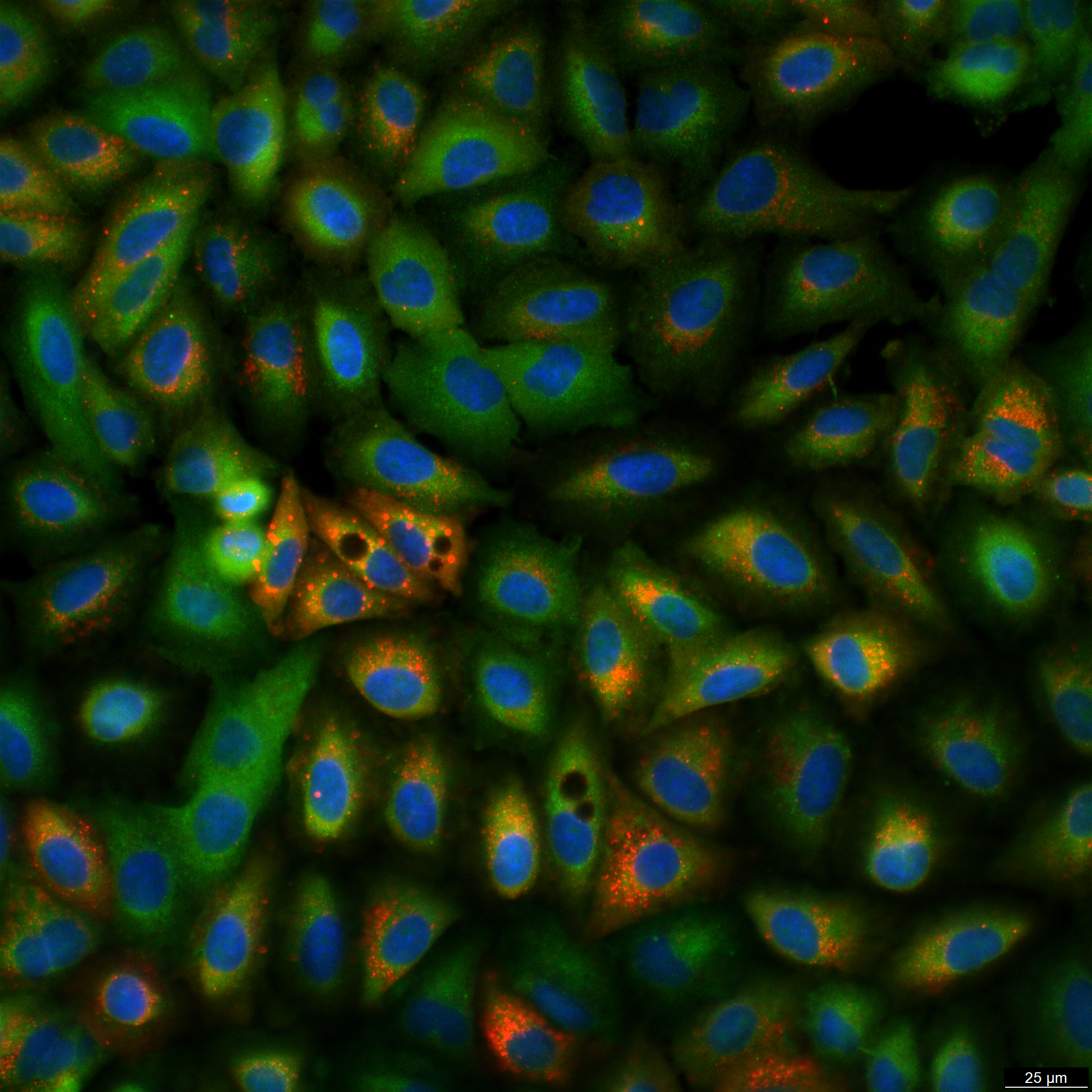

Supplement: Supplementary file 10 — Source data Fig. 6 [file 44318_2025_421_MOESM10_ESM.zip › Figure 6/Figure 6E/Control.tif]

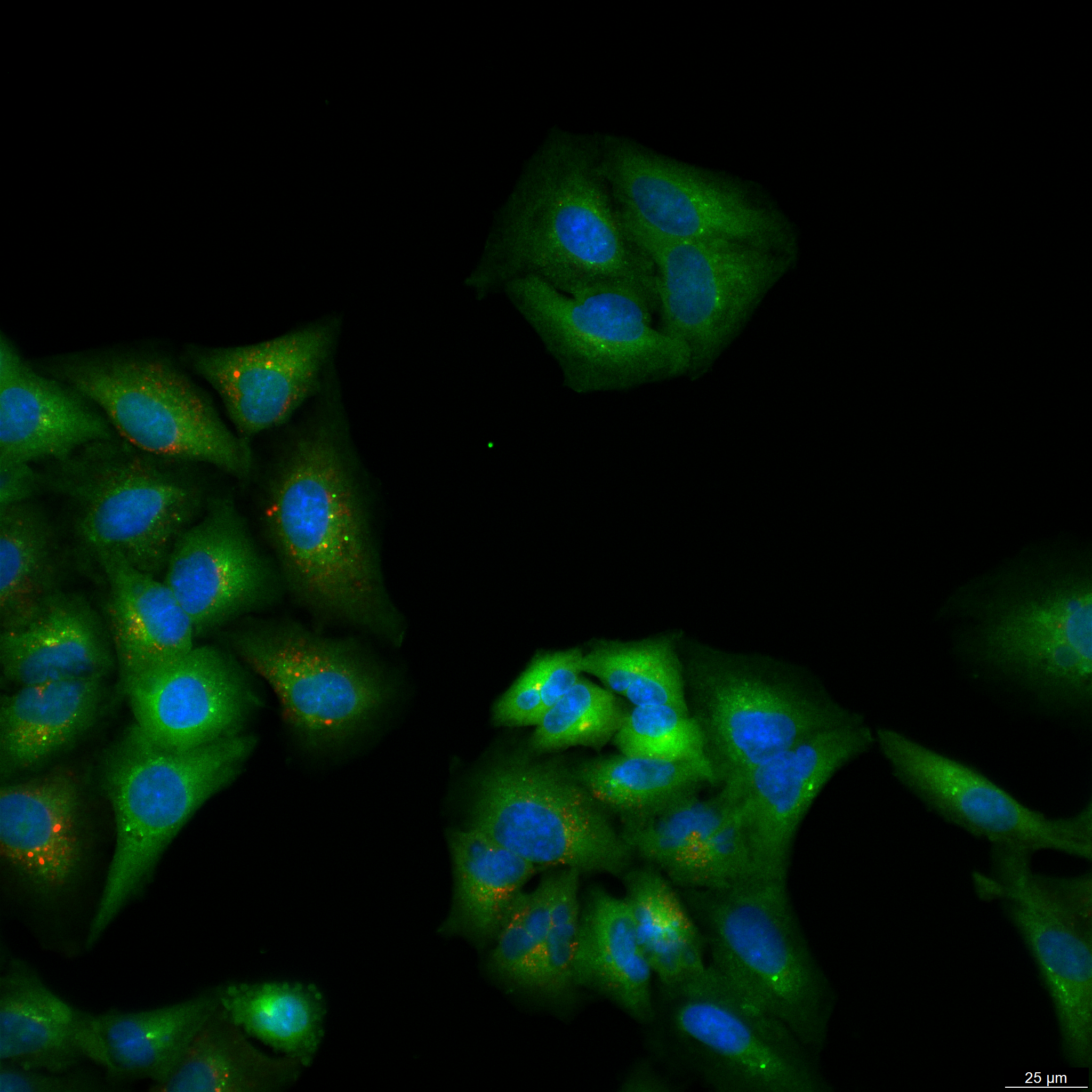

Supplement: Supplementary file 10 — Source data Fig. 6 [file 44318_2025_421_MOESM10_ESM.zip › Figure 6/Figure 6E/lFNγ+MG132.tif]

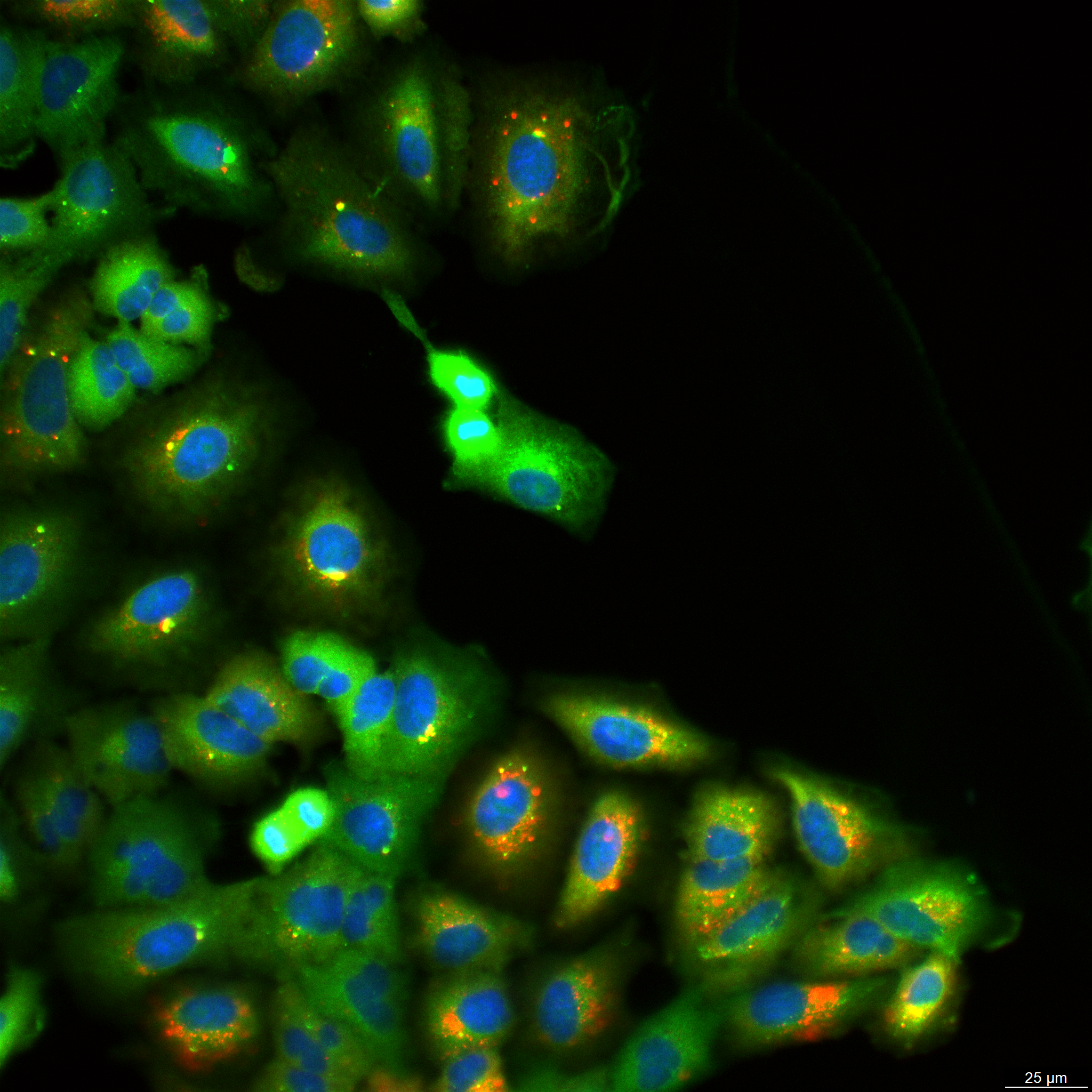

Supplement: Supplementary file 10 — Source data Fig. 6 [file 44318_2025_421_MOESM10_ESM.zip › Figure 6/Figure 6E/lFNγ.tif]

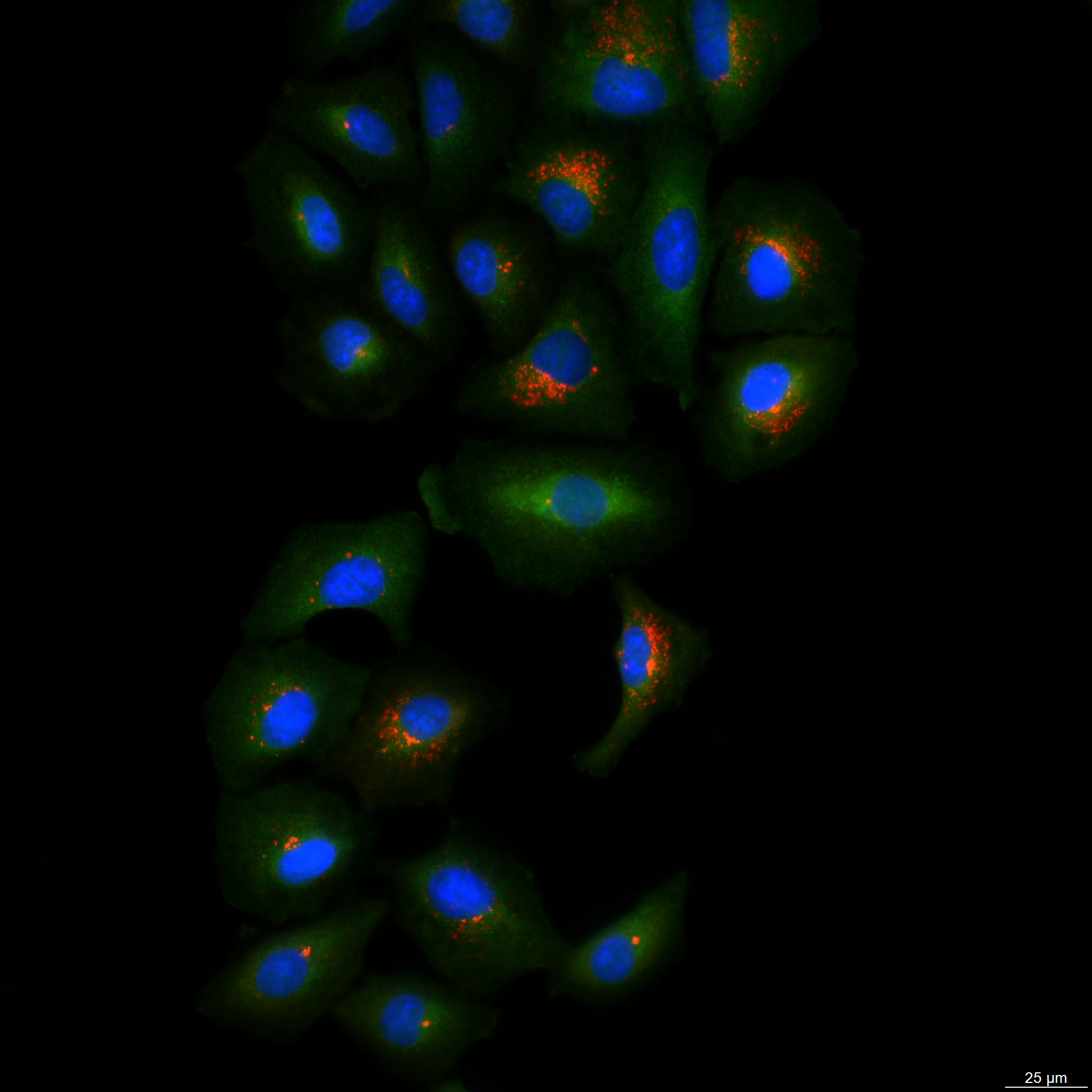

Supplement: Supplementary file 10 — Source data Fig. 6 [file 44318_2025_421_MOESM10_ESM.zip › Figure 6/Figure 6E/MG132.tif]

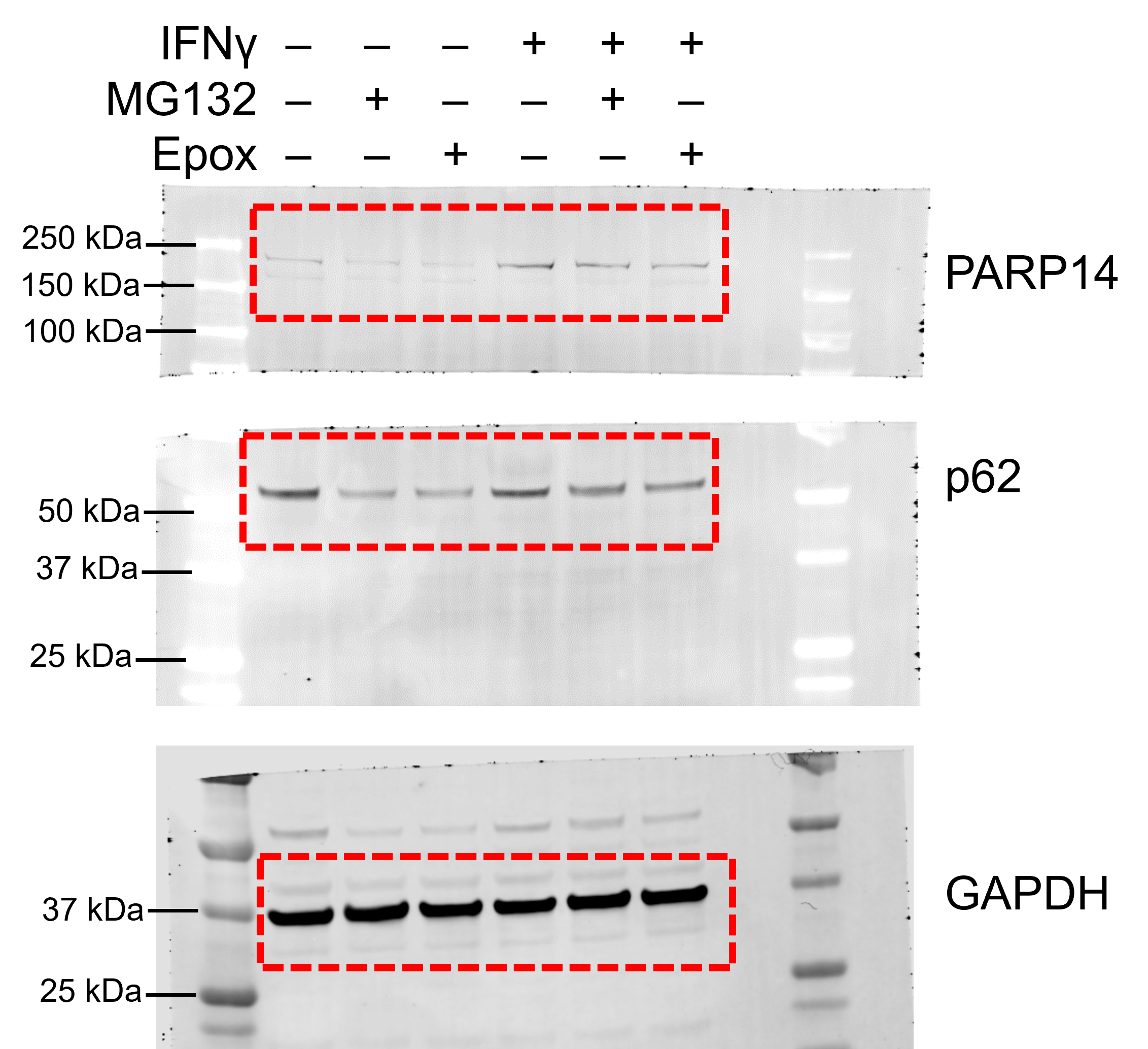

Supplement: Supplementary file 10 — Source data Fig. 6 [file 44318_2025_421_MOESM10_ESM.zip › Figure 6/Figure 6F.tif]

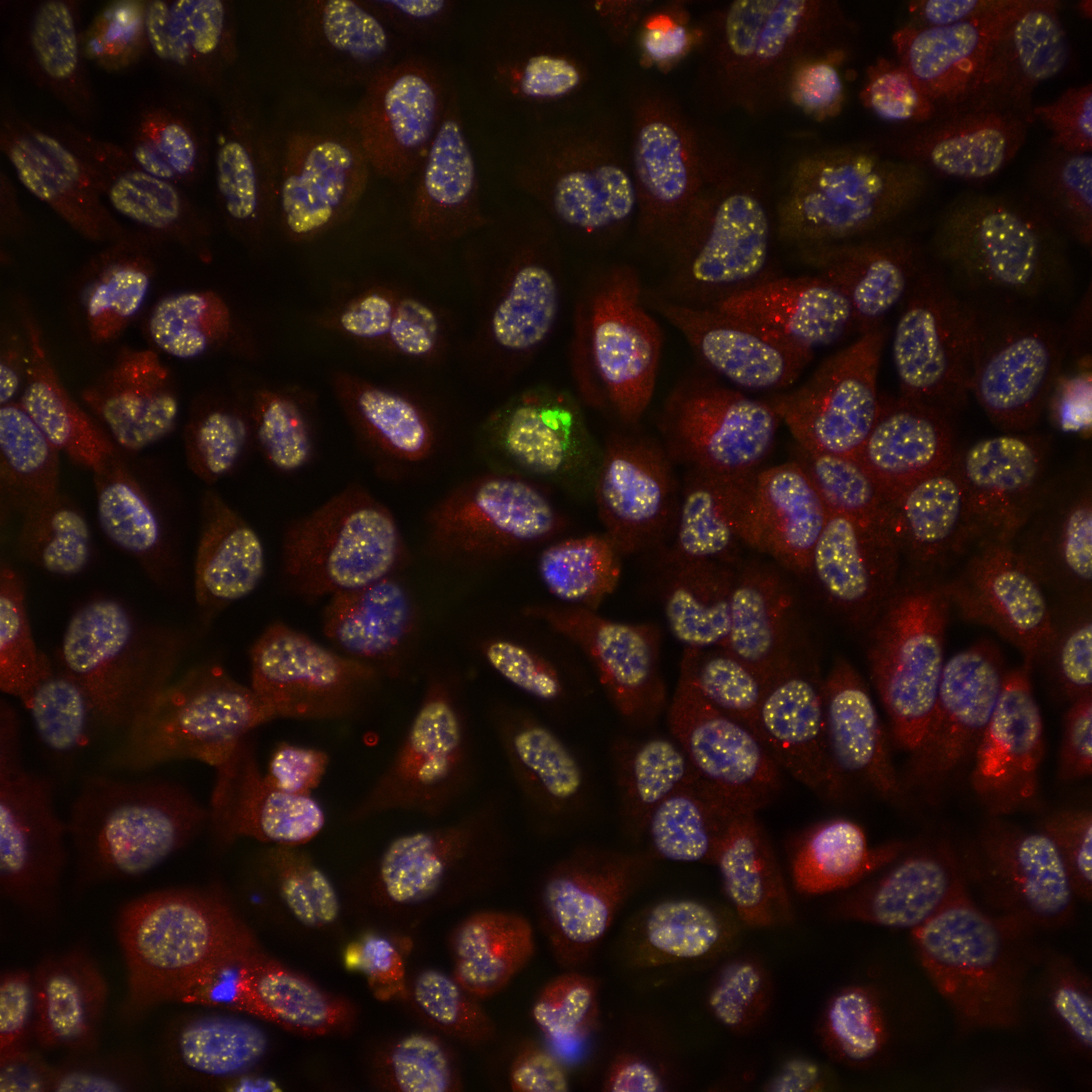

Supplement: Supplementary file 10 — Source data Fig. 6 [file 44318_2025_421_MOESM10_ESM.zip › Figure 6/Figure 6G/Cell_10.tif]

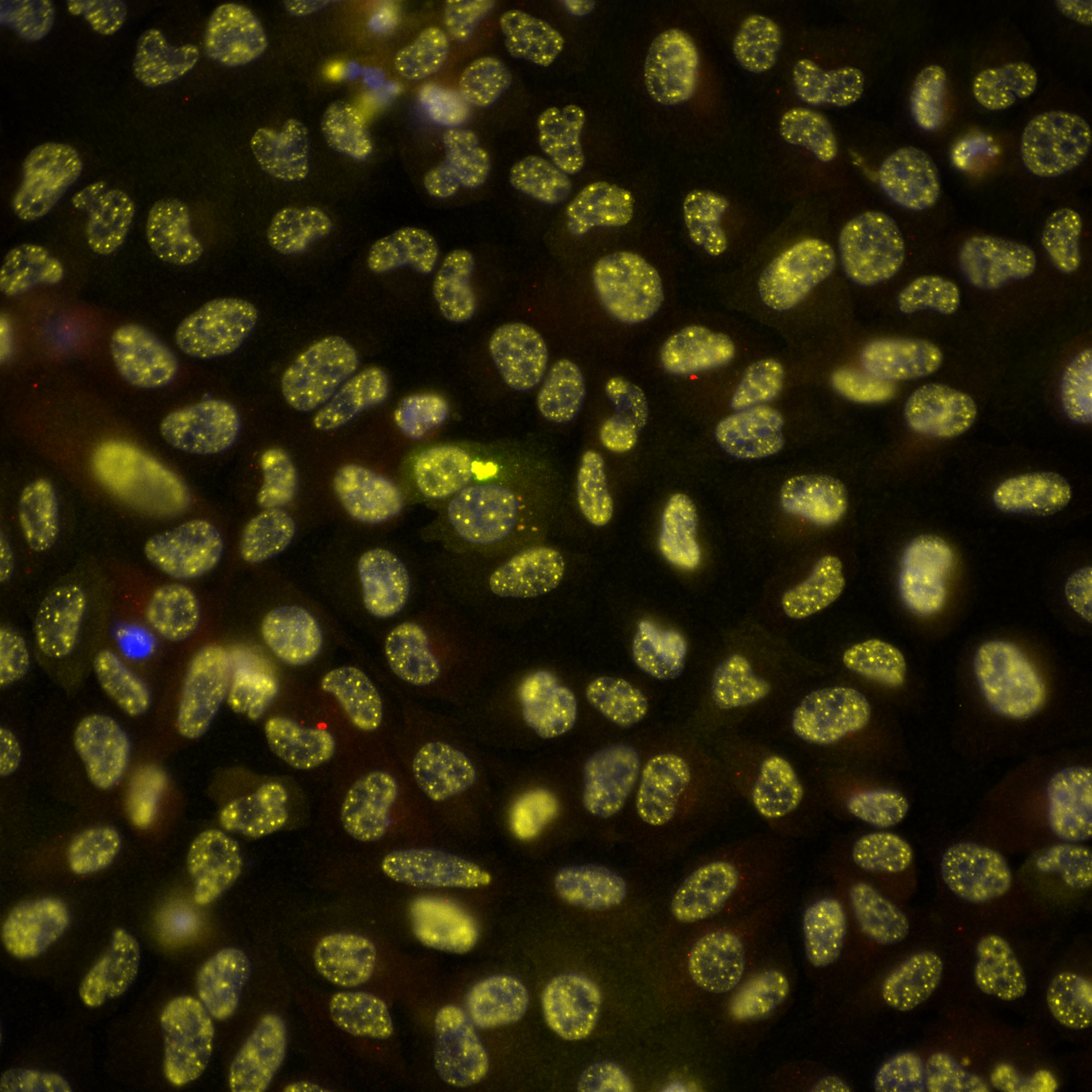

Supplement: Supplementary file 10 — Source data Fig. 6 [file 44318_2025_421_MOESM10_ESM.zip › Figure 6/Figure 6G/Cell_11.tif]

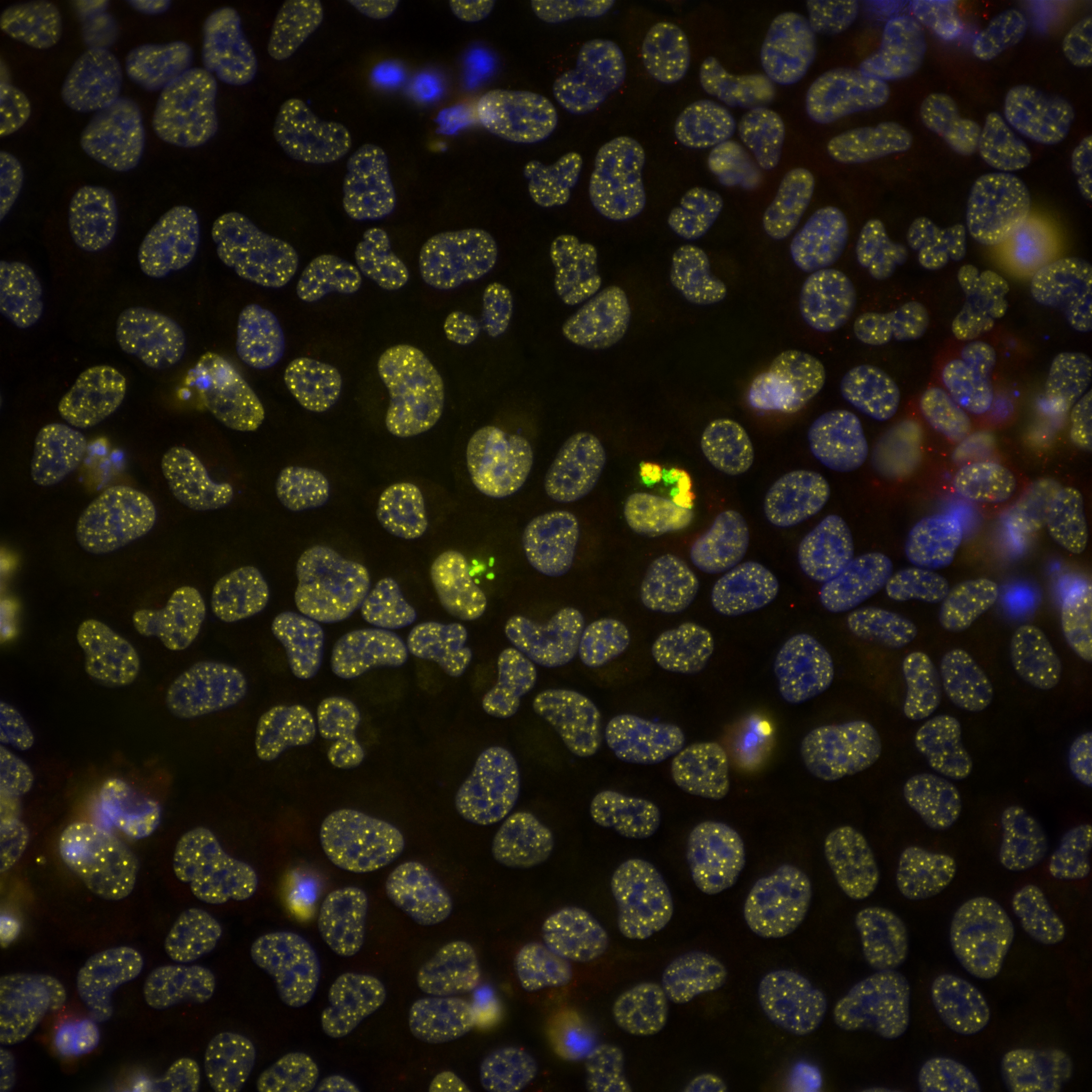

Supplement: Supplementary file 10 — Source data Fig. 6 [file 44318_2025_421_MOESM10_ESM.zip › Figure 6/Figure 6G/Cell_12.tif]

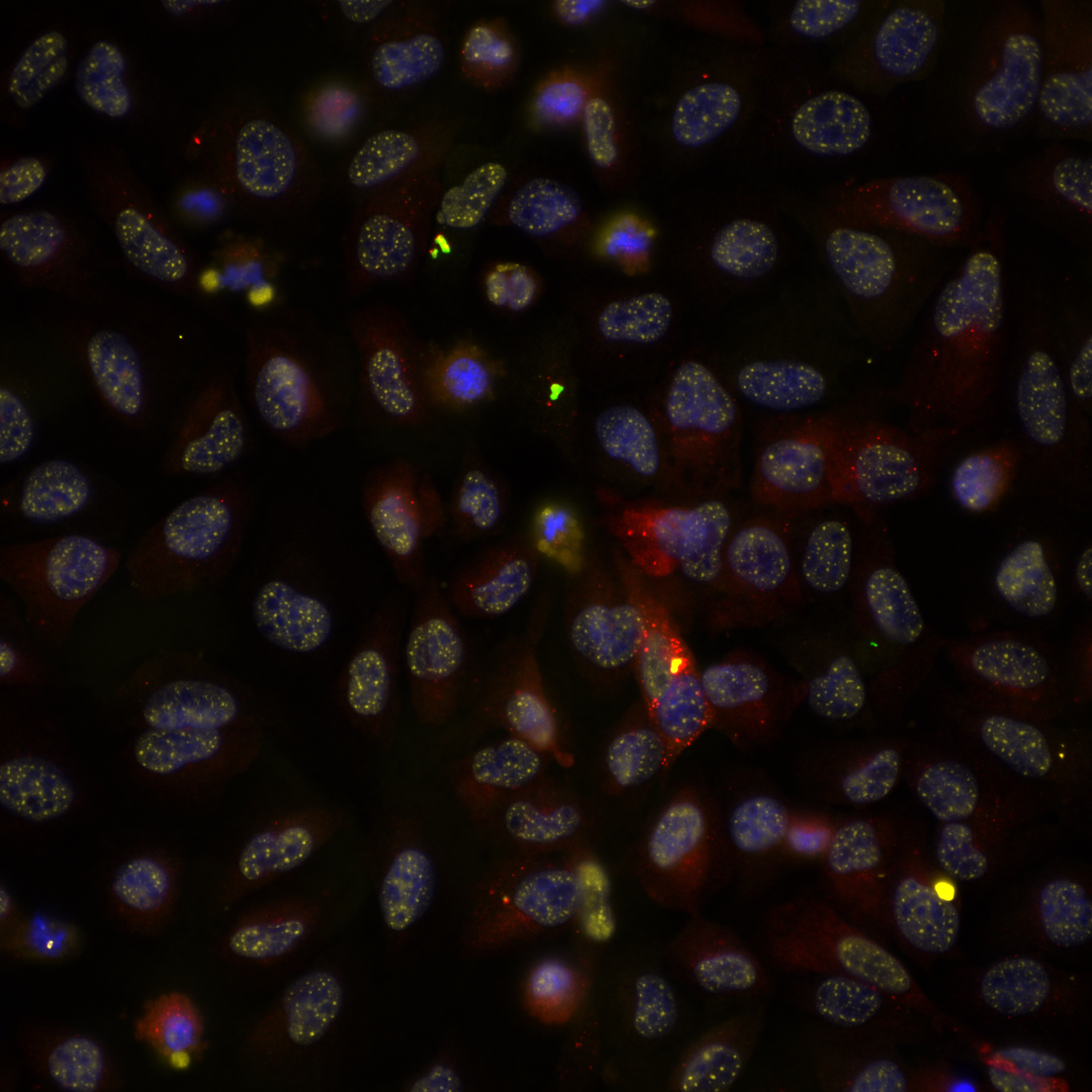

Supplement: Supplementary file 10 — Source data Fig. 6 [file 44318_2025_421_MOESM10_ESM.zip › Figure 6/Figure 6G/Cell_1_2_3.tif]

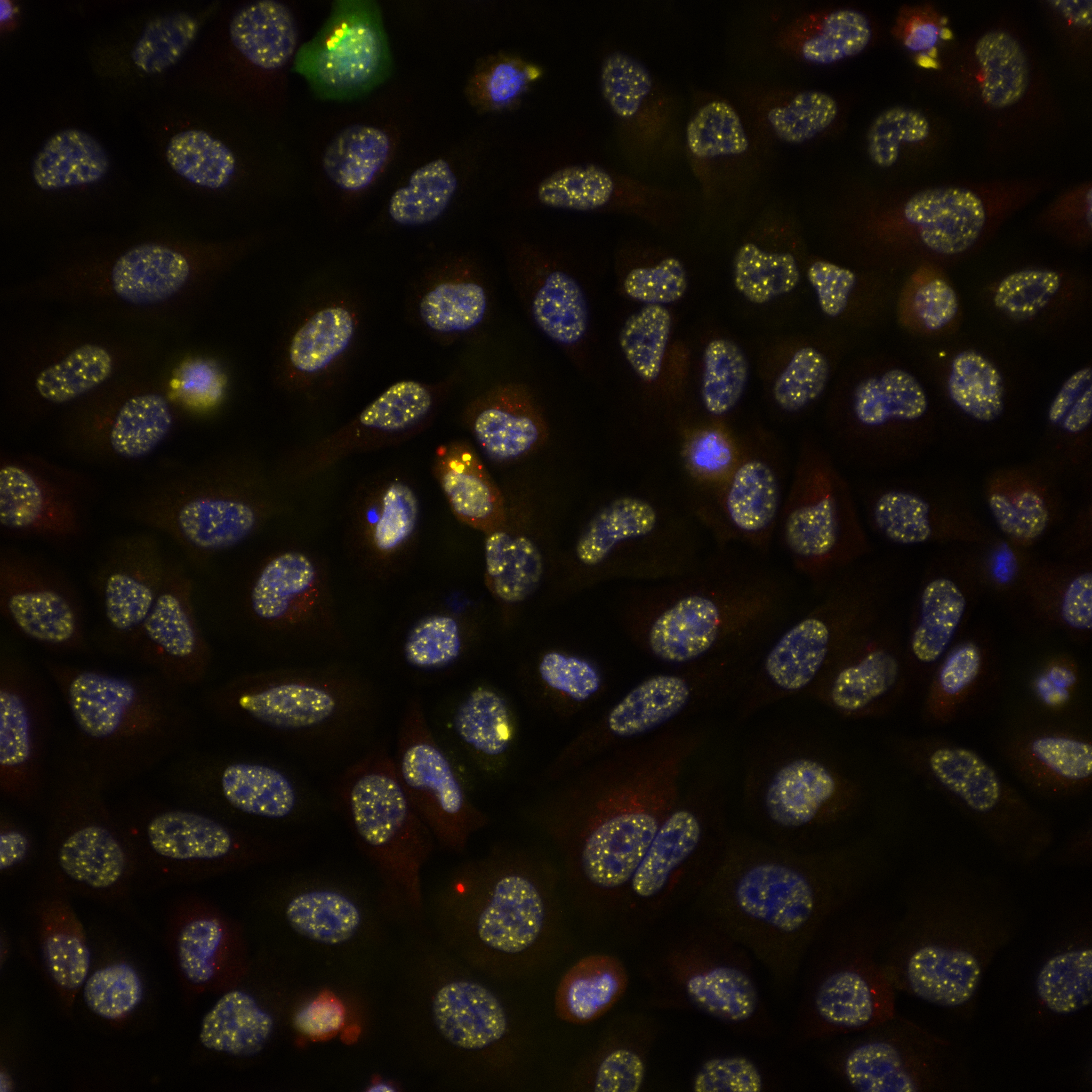

Supplement: Supplementary file 10 — Source data Fig. 6 [file 44318_2025_421_MOESM10_ESM.zip › Figure 6/Figure 6G/Cell_4_8.tif]

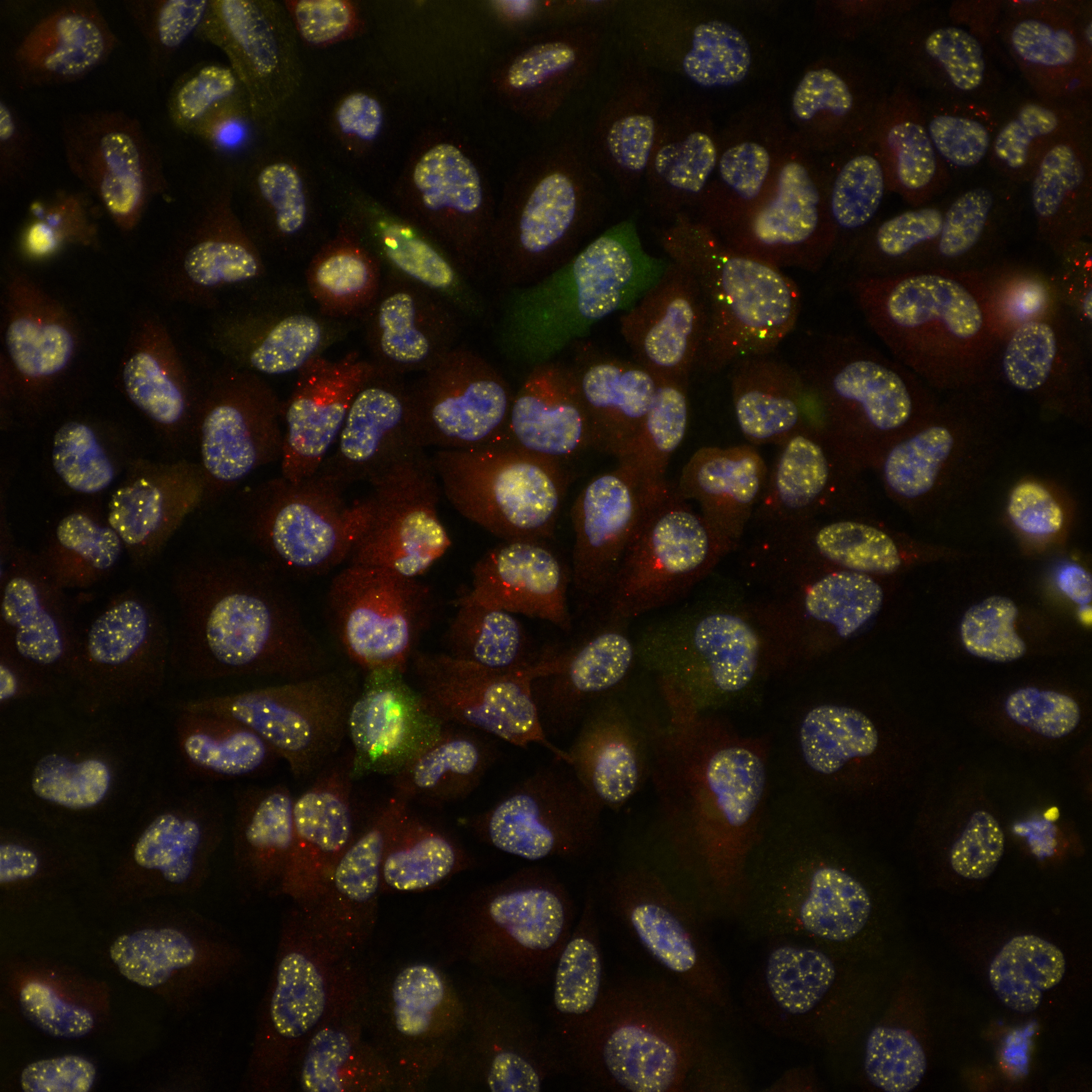

Supplement: Supplementary file 10 — Source data Fig. 6 [file 44318_2025_421_MOESM10_ESM.zip › Figure 6/Figure 6G/Cell_5_6.tif]

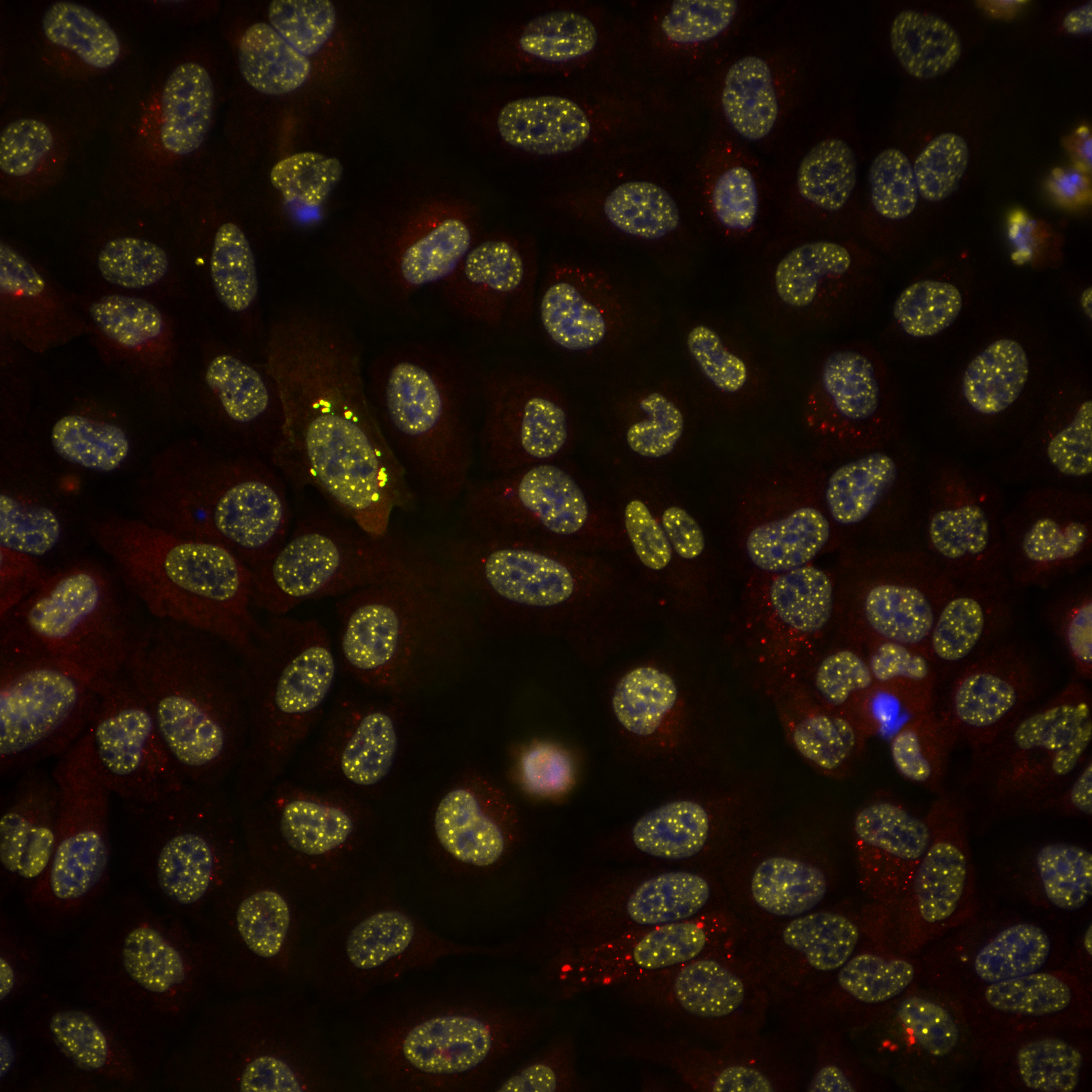

Supplement: Supplementary file 10 — Source data Fig. 6 [file 44318_2025_421_MOESM10_ESM.zip › Figure 6/Figure 6G/Cell_7.tif]

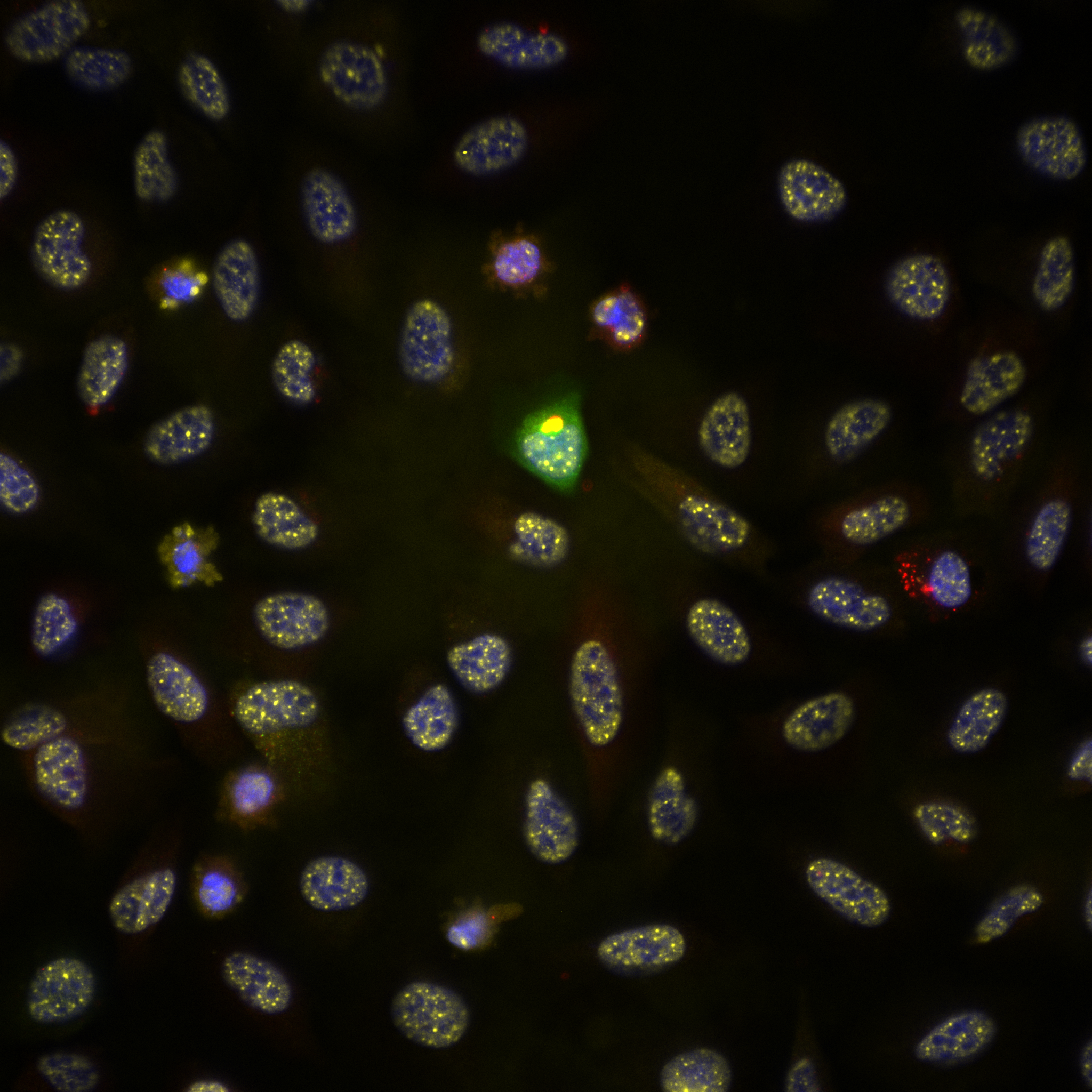

Supplement: Supplementary file 10 — Source data Fig. 6 [file 44318_2025_421_MOESM10_ESM.zip › Figure 6/Figure 6G/Cell_9.tif]

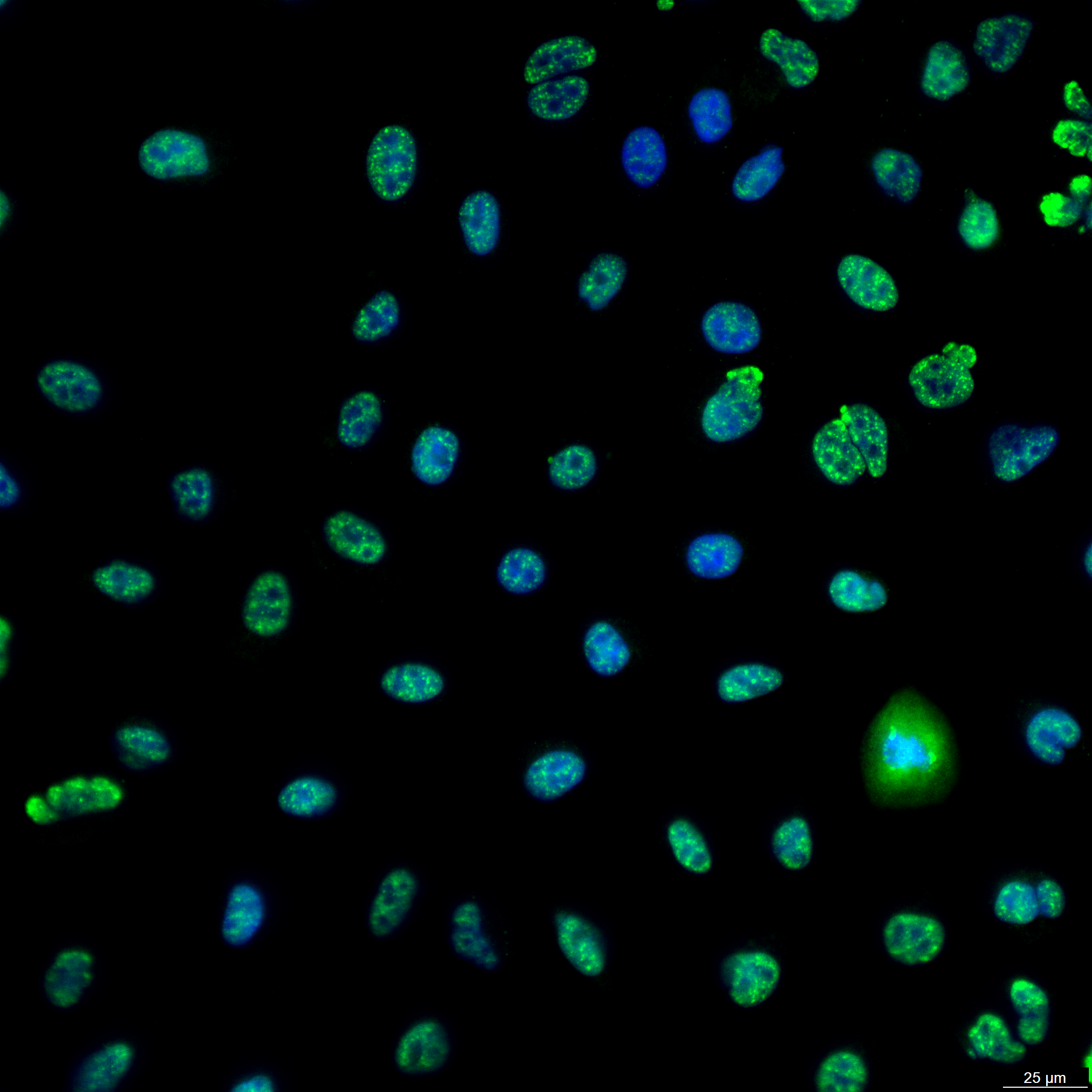

Supplement: Supplementary file 11 — Source data Fig. 7 [file 44318_2025_421_MOESM11_ESM.zip › Figure 7/Figure 7A/UBA1 KD.tif]

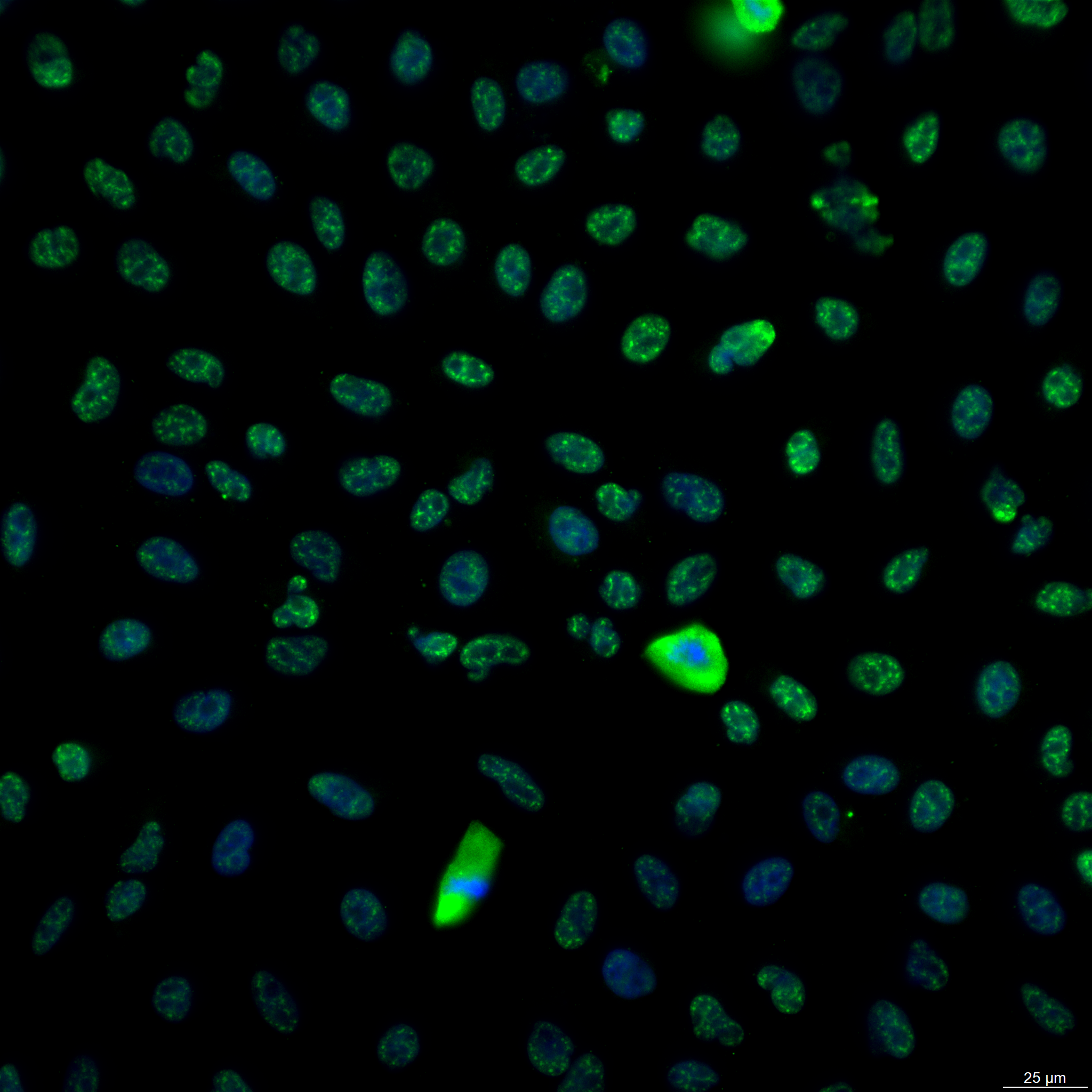

Supplement: Supplementary file 11 — Source data Fig. 7 [file 44318_2025_421_MOESM11_ESM.zip › Figure 7/Figure 7A/UBA1+lFNγ.tif]

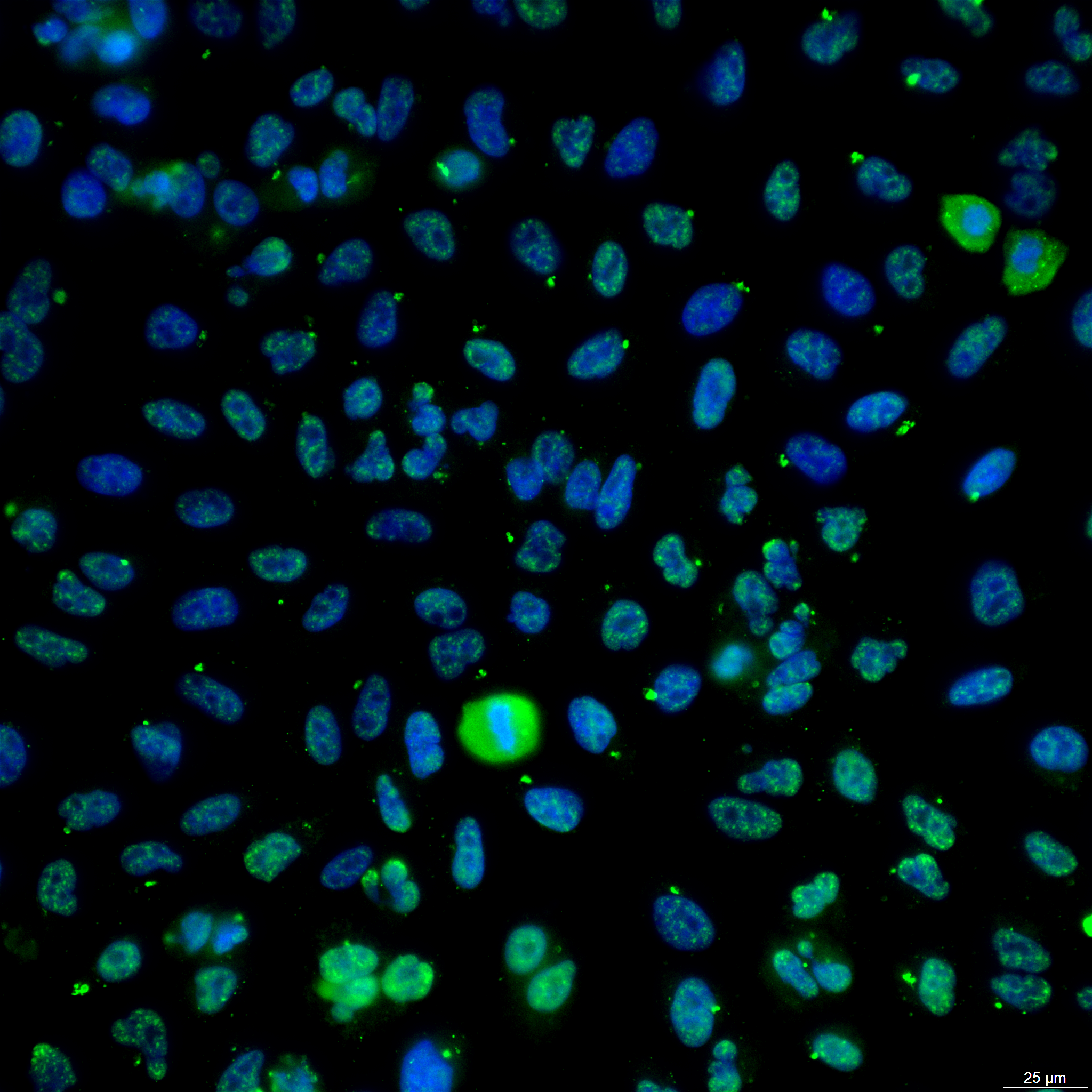

Supplement: Supplementary file 11 — Source data Fig. 7 [file 44318_2025_421_MOESM11_ESM.zip › Figure 7/Figure 7A/WT+lFNγ.tif]

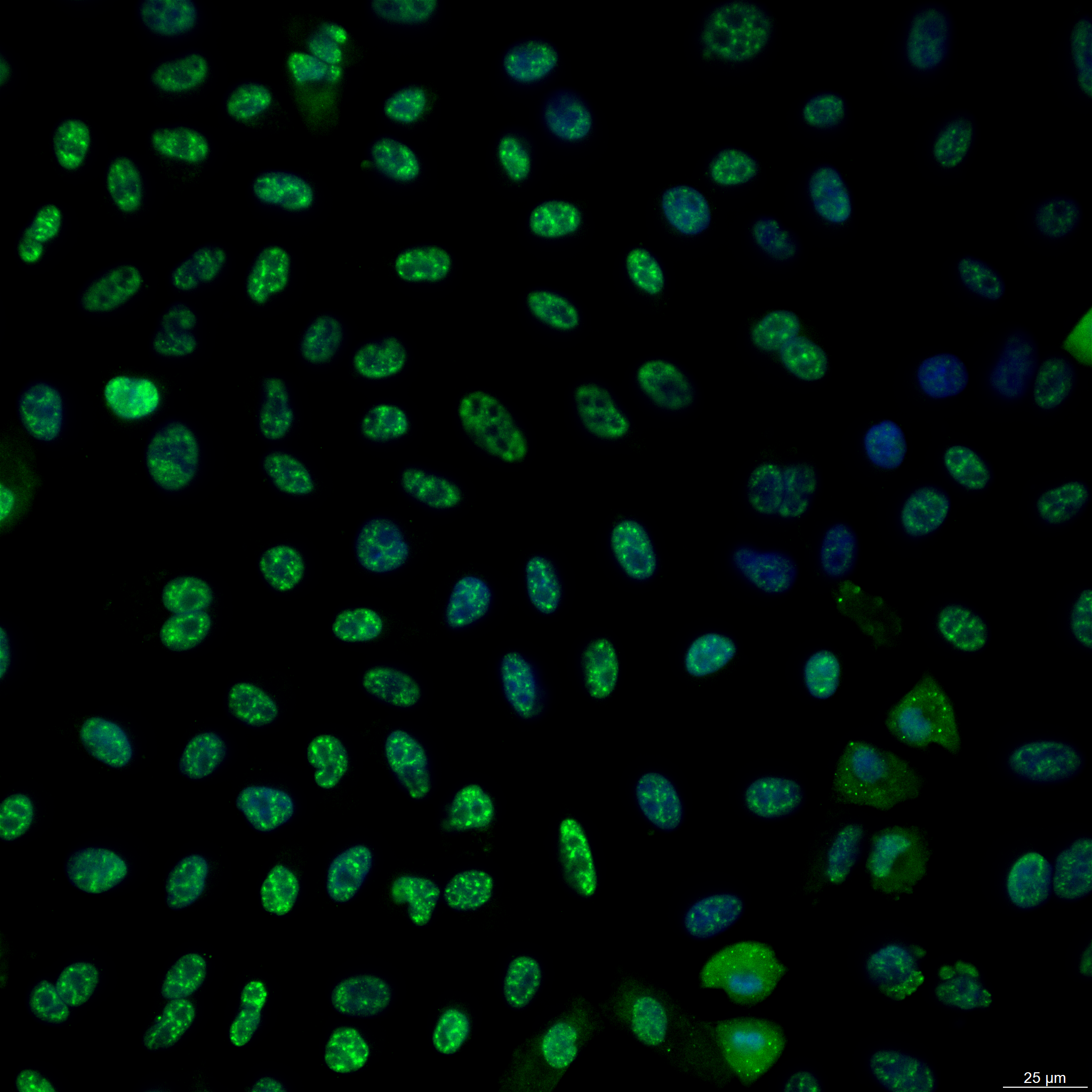

Supplement: Supplementary file 11 — Source data Fig. 7 [file 44318_2025_421_MOESM11_ESM.zip › Figure 7/Figure 7A/WT.tif]

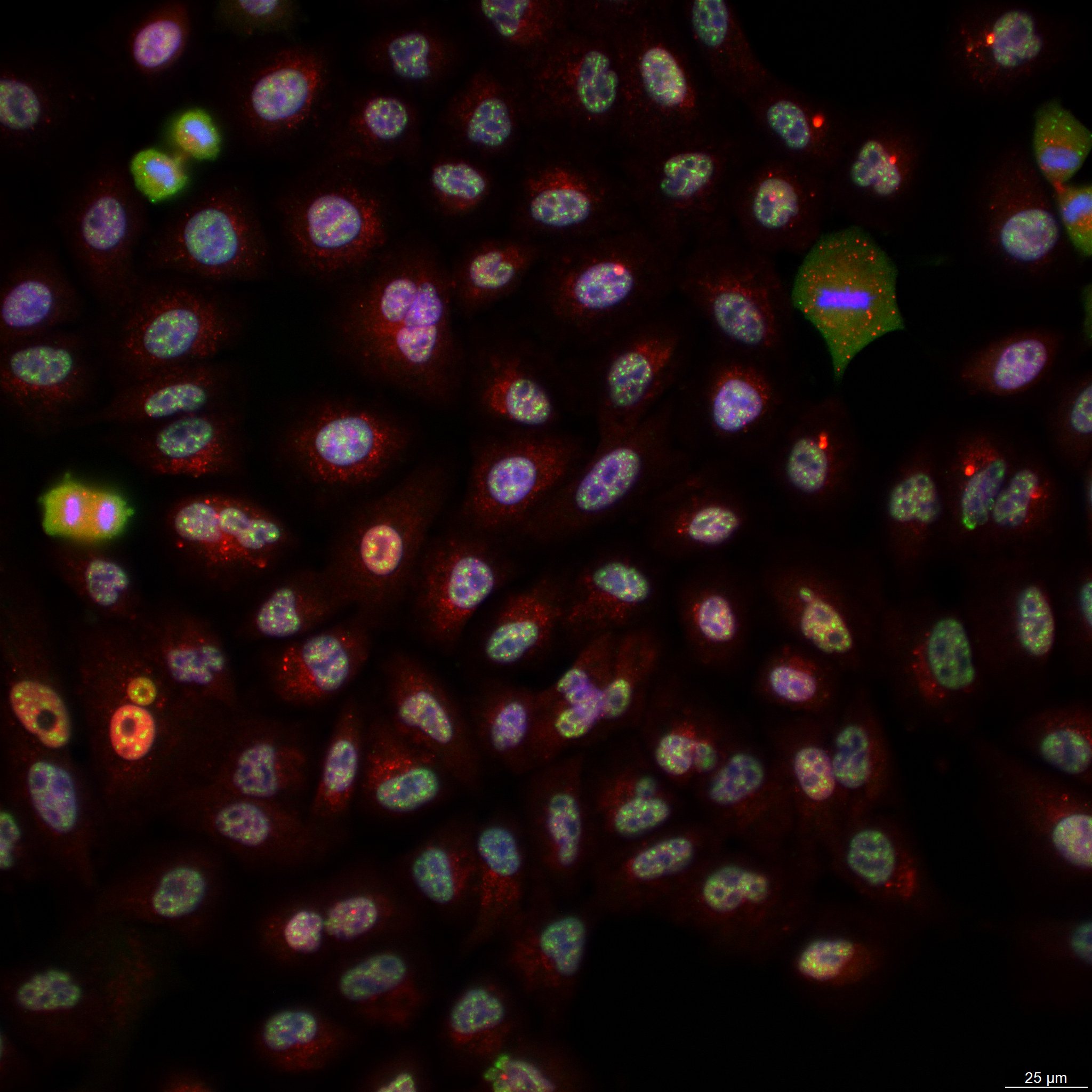

Supplement: Supplementary file 11 — Source data Fig. 7 [file 44318_2025_421_MOESM11_ESM.zip › Figure 7/Figure 7B/Control 1_overlay.tif]

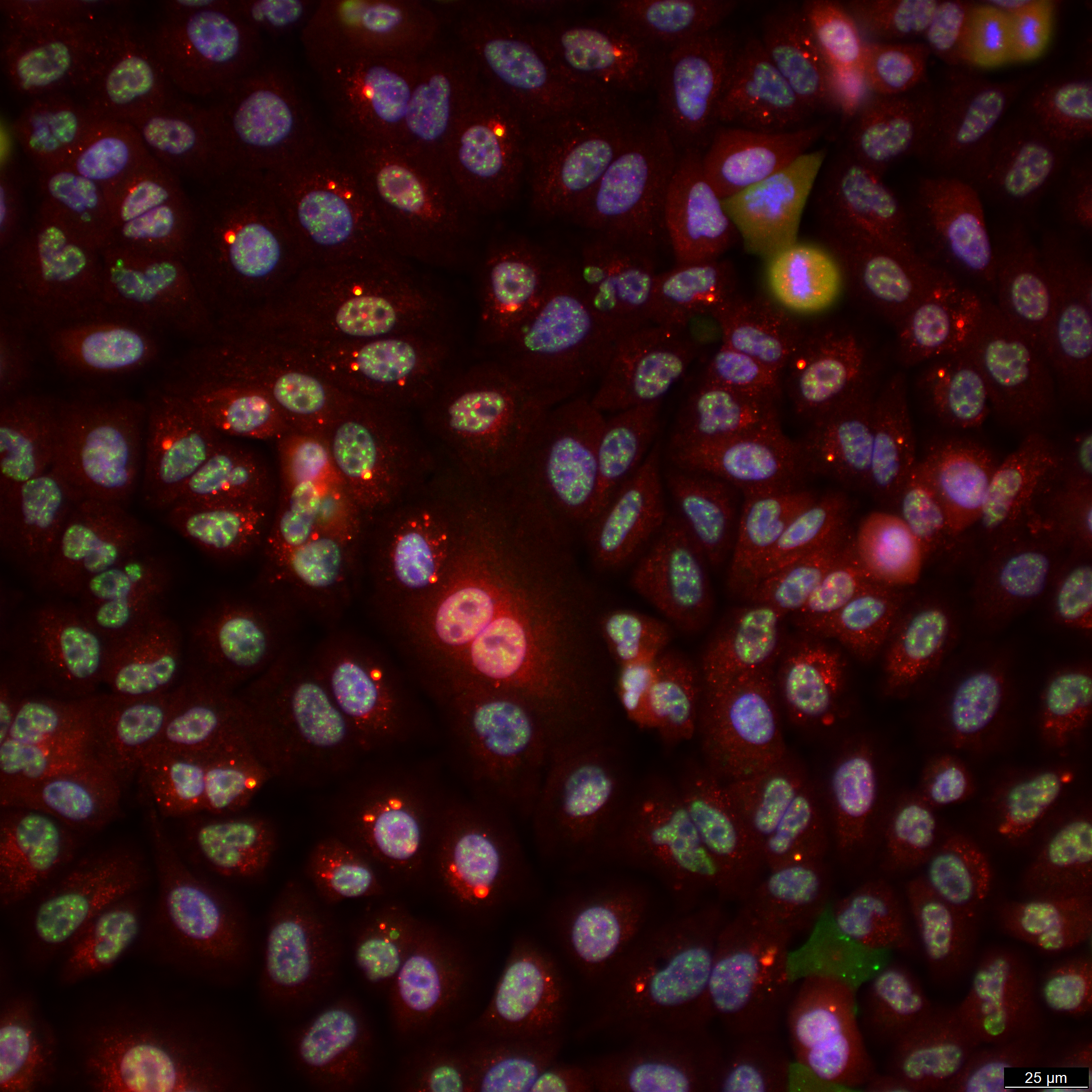

Supplement: Supplementary file 11 — Source data Fig. 7 [file 44318_2025_421_MOESM11_ESM.zip › Figure 7/Figure 7B/IFN 1_overlay.tif]

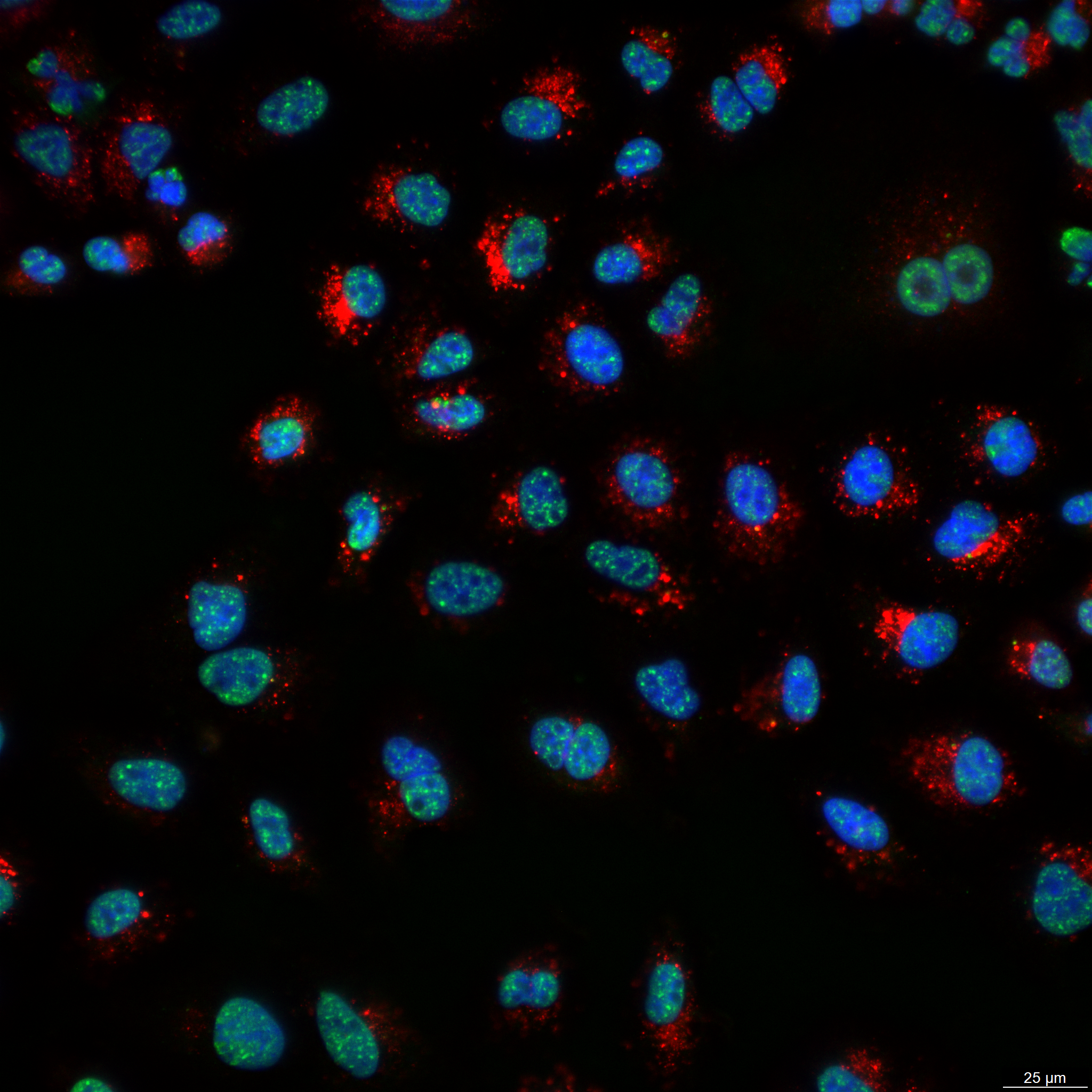

Supplement: Supplementary file 11 — Source data Fig. 7 [file 44318_2025_421_MOESM11_ESM.zip › Figure 7/Figure 7B/IFN+TAK243 1_overlay.tif]

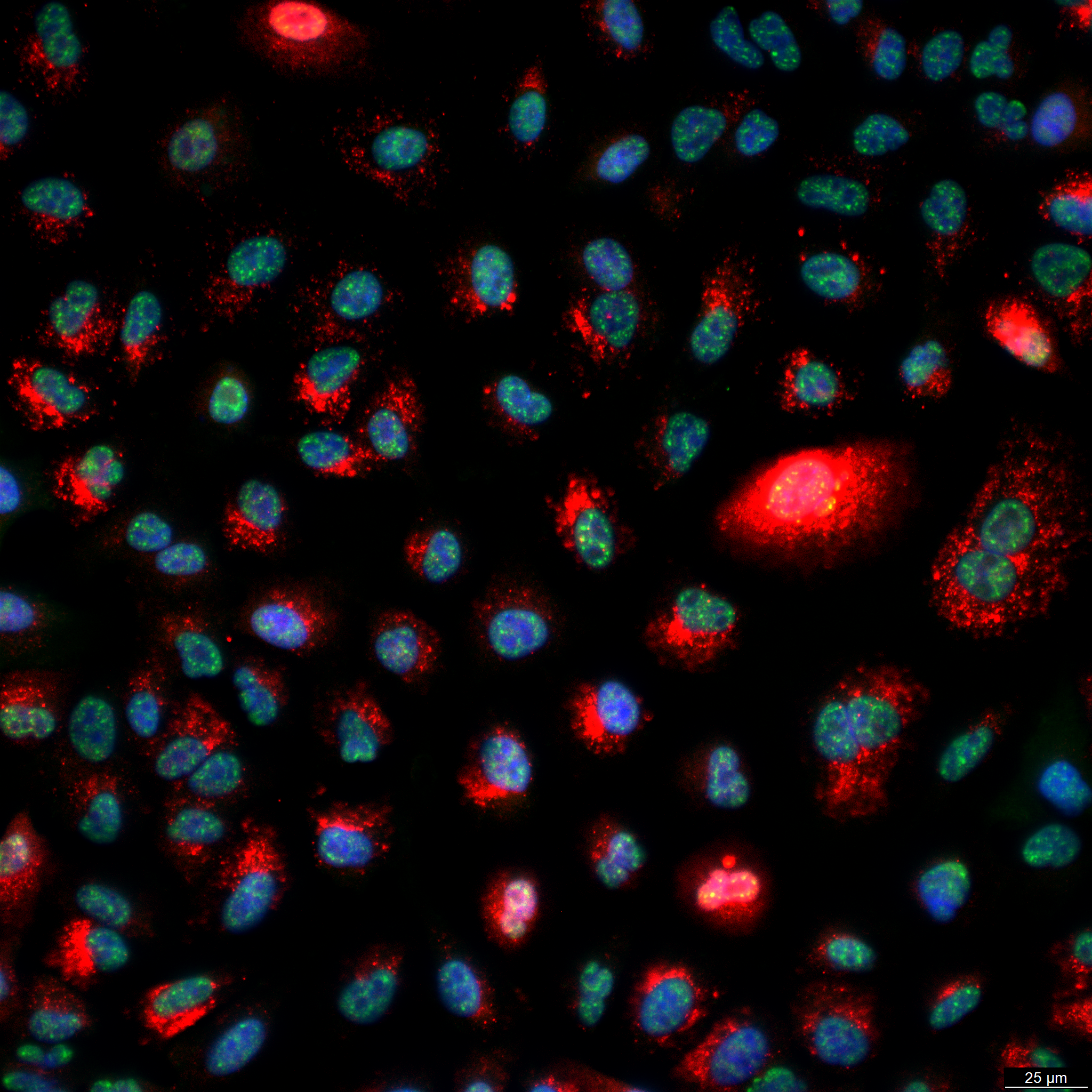

Supplement: Supplementary file 11 — Source data Fig. 7 [file 44318_2025_421_MOESM11_ESM.zip › Figure 7/Figure 7B/TAK243 1_overlay.tif]

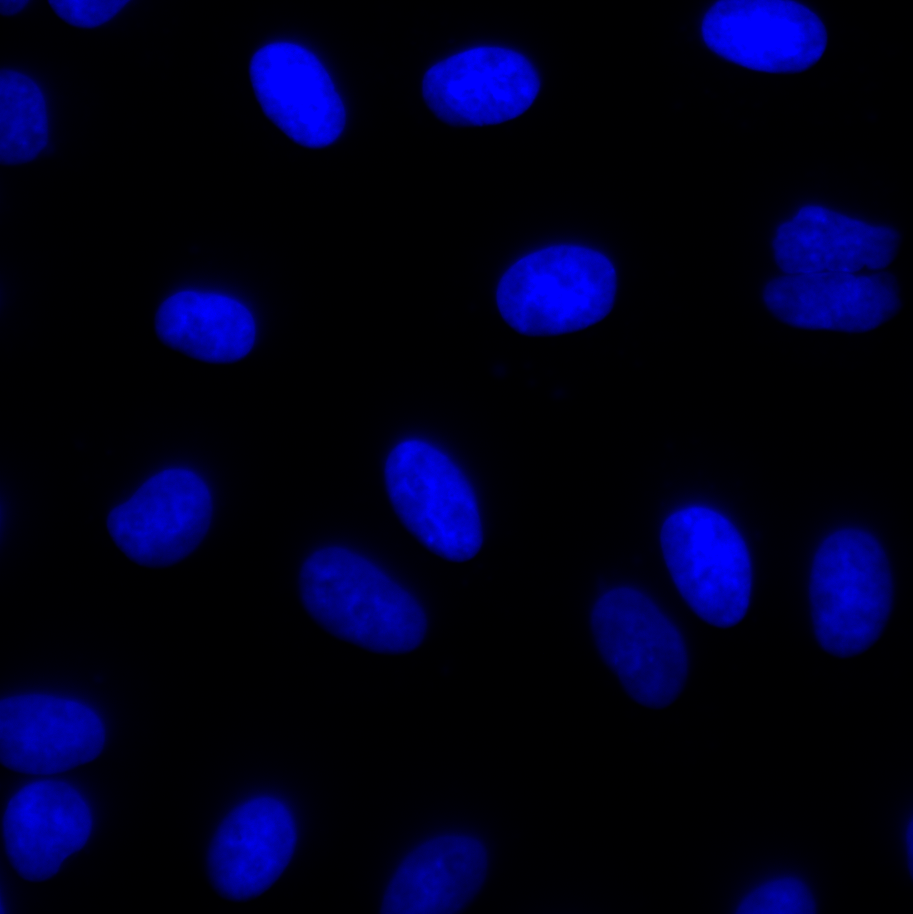

Supplement: Supplementary file 11 — Source data Fig. 7 [file 44318_2025_421_MOESM11_ESM.zip › Figure 7/Figure 7C/Control 1 DTX3L_Crop001_ch00_SV.tif]

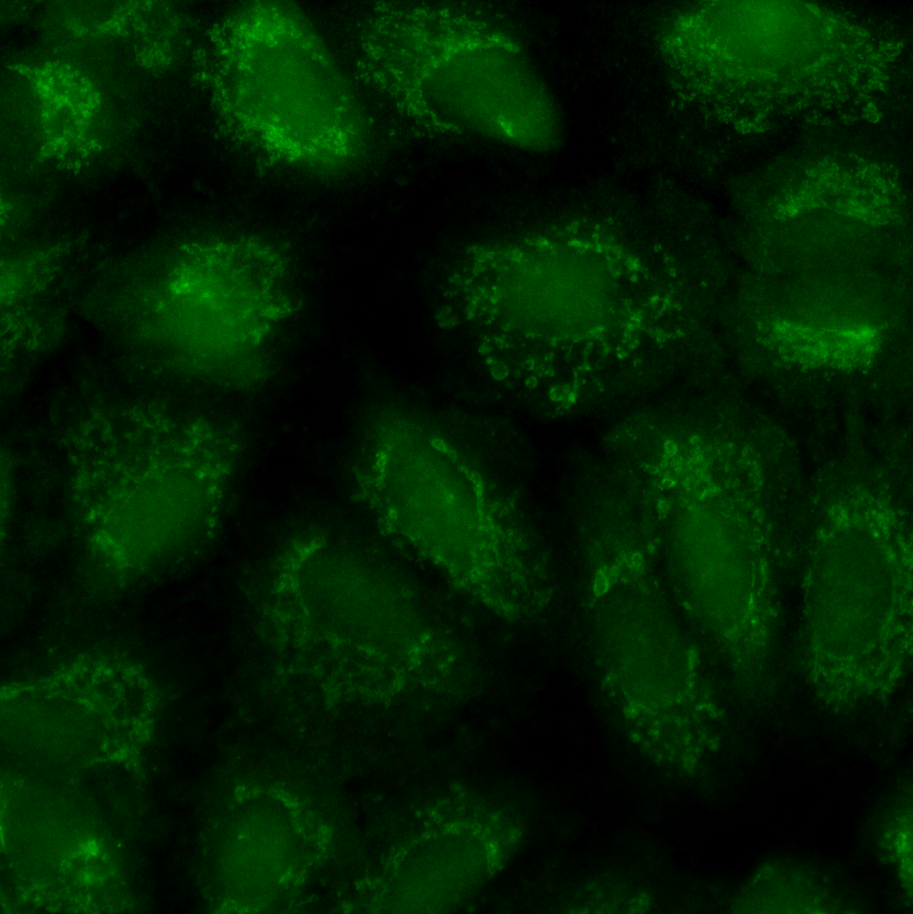

Supplement: Supplementary file 11 — Source data Fig. 7 [file 44318_2025_421_MOESM11_ESM.zip › Figure 7/Figure 7C/Control 1 DTX3L_Crop001_ch01_SV.tif]

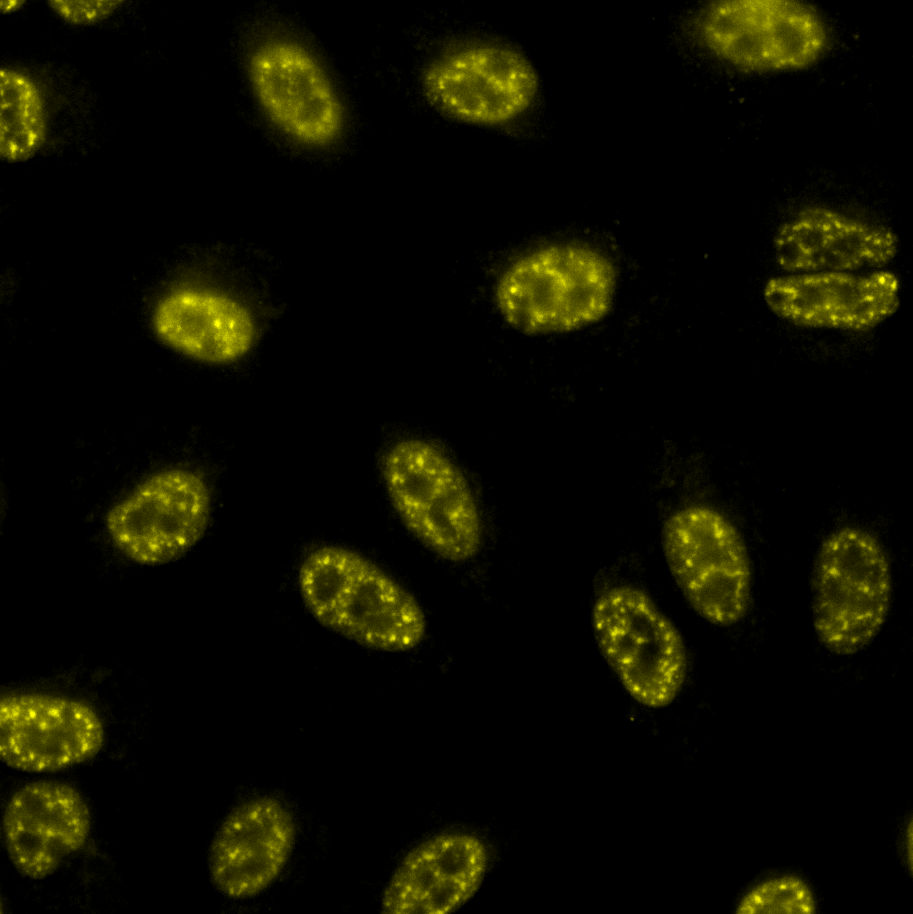

Supplement: Supplementary file 11 — Source data Fig. 7 [file 44318_2025_421_MOESM11_ESM.zip › Figure 7/Figure 7C/Control 1 DTX3L_Crop001_ch02_SV.tif]

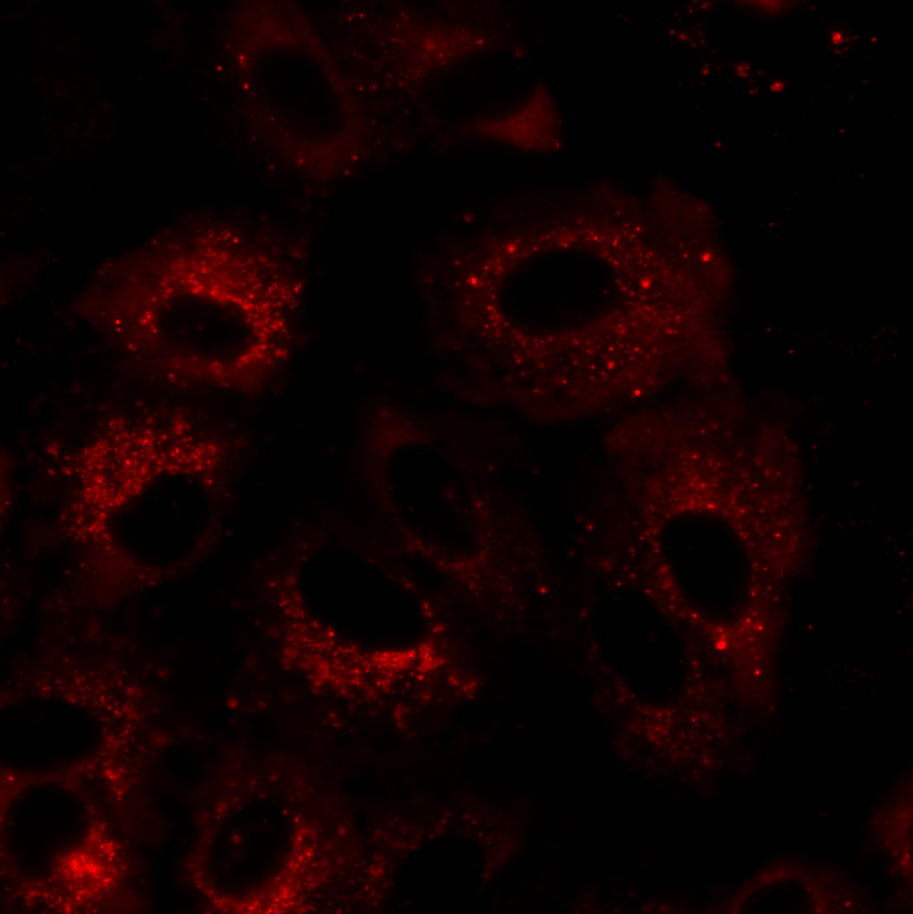

Supplement: Supplementary file 11 — Source data Fig. 7 [file 44318_2025_421_MOESM11_ESM.zip › Figure 7/Figure 7C/Control 1 DTX3L_Crop001_ch03_SV.tif]

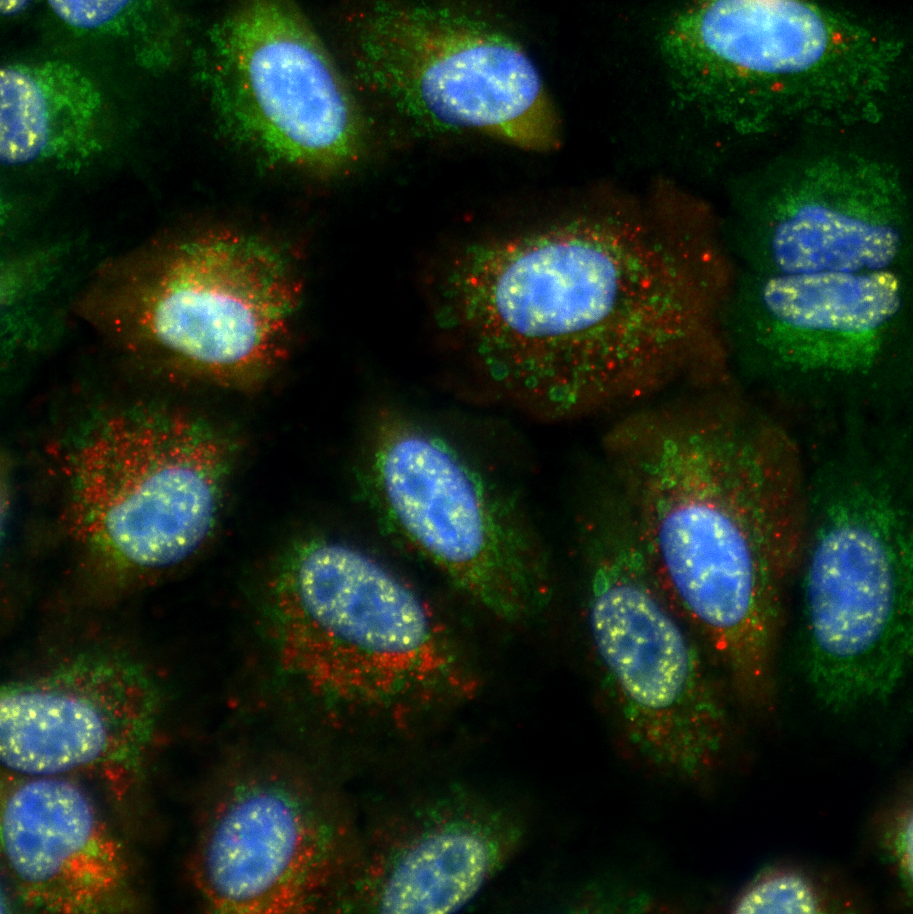

Supplement: Supplementary file 11 — Source data Fig. 7 [file 44318_2025_421_MOESM11_ESM.zip › Figure 7/Figure 7C/Control 1 DTX3L_Crop001_overlay.tif]

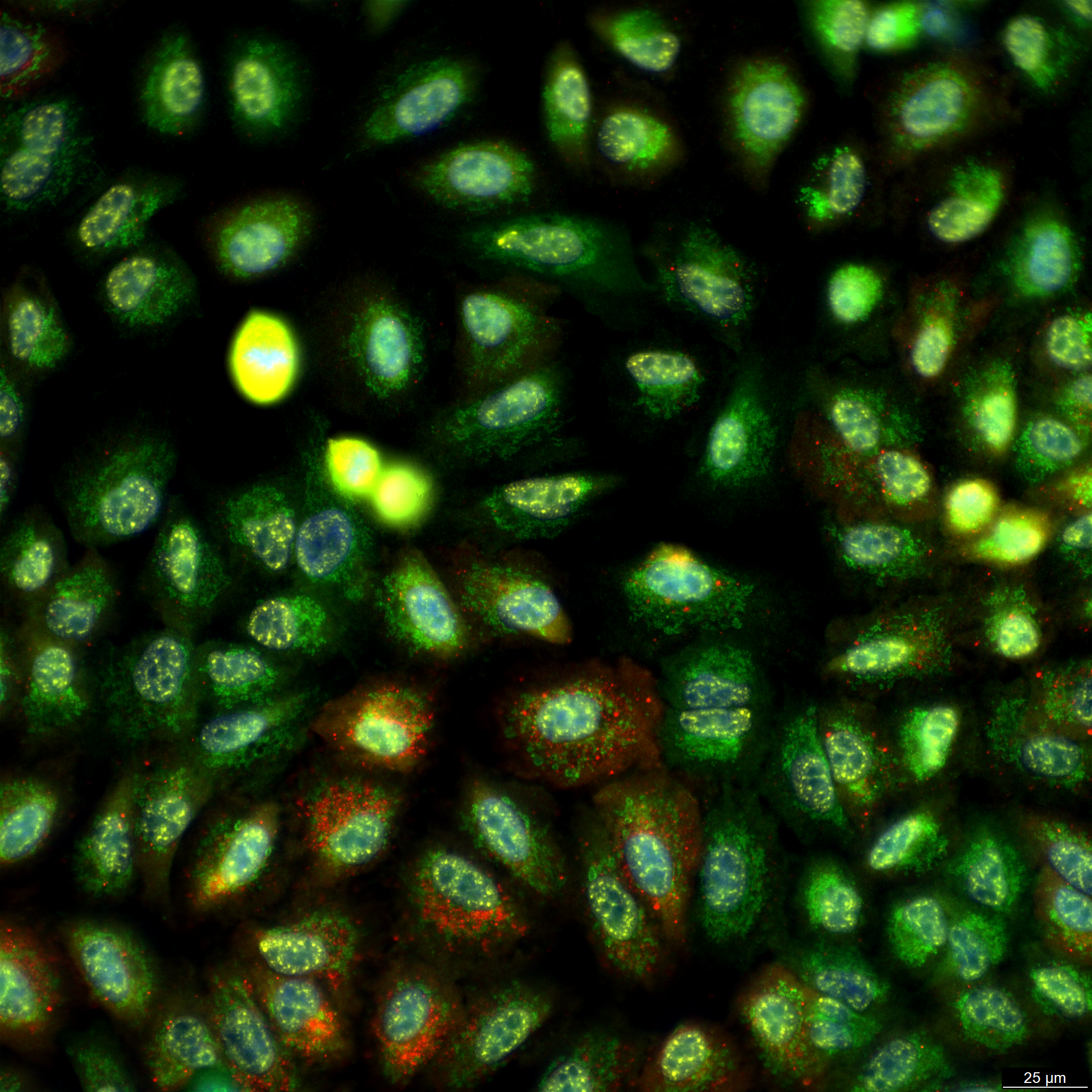

Supplement: Supplementary file 11 — Source data Fig. 7 [file 44318_2025_421_MOESM11_ESM.zip › Figure 7/Figure 7C/Control Merge.tif]

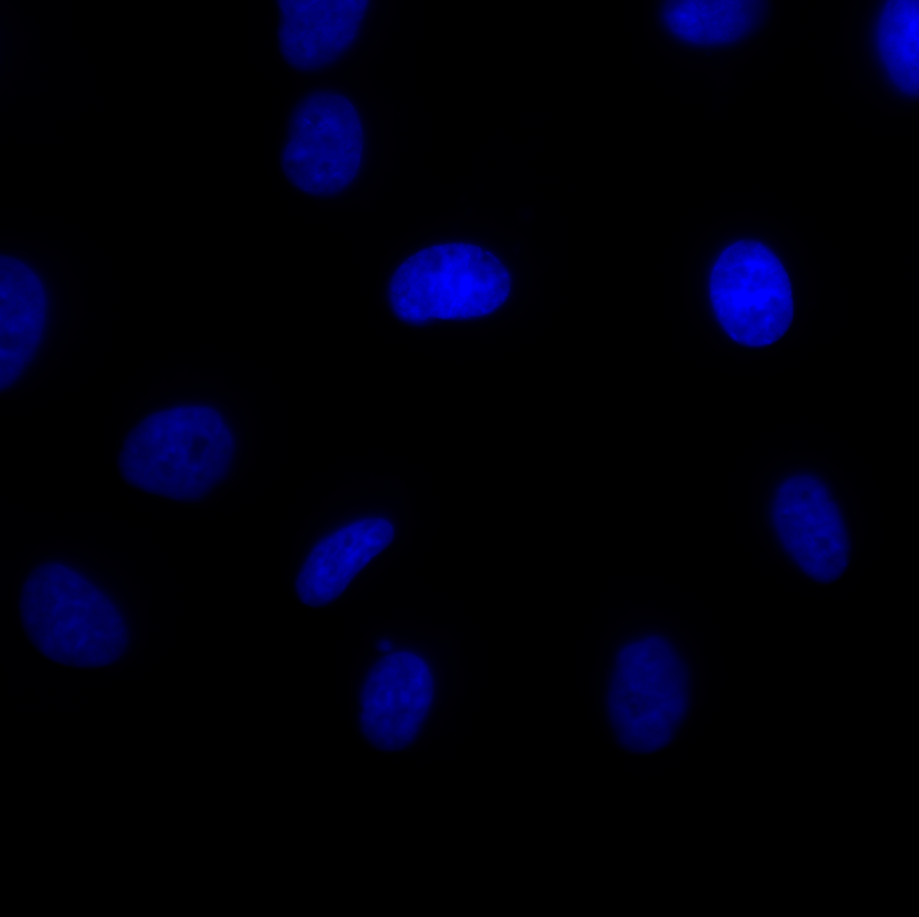

Supplement: Supplementary file 11 — Source data Fig. 7 [file 44318_2025_421_MOESM11_ESM.zip › Figure 7/Figure 7C/IFN DTX3L 2_Crop001_ch00_SV.tif]

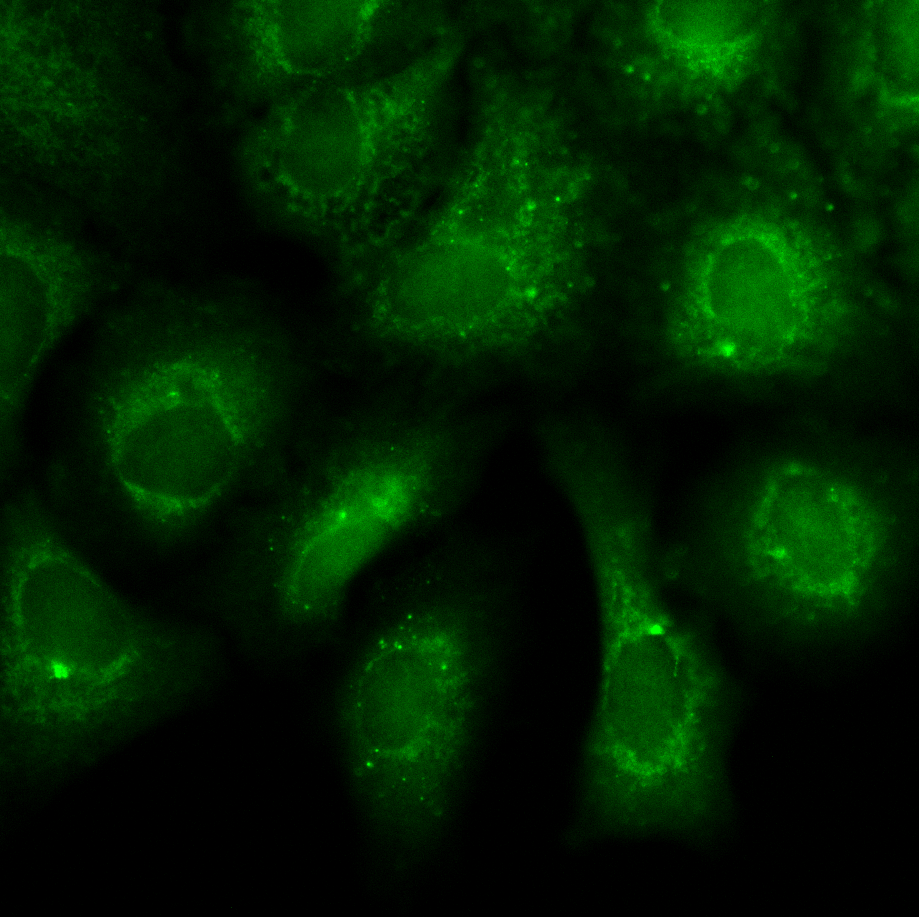

Supplement: Supplementary file 11 — Source data Fig. 7 [file 44318_2025_421_MOESM11_ESM.zip › Figure 7/Figure 7C/IFN DTX3L 2_Crop001_ch01_SV.tif]

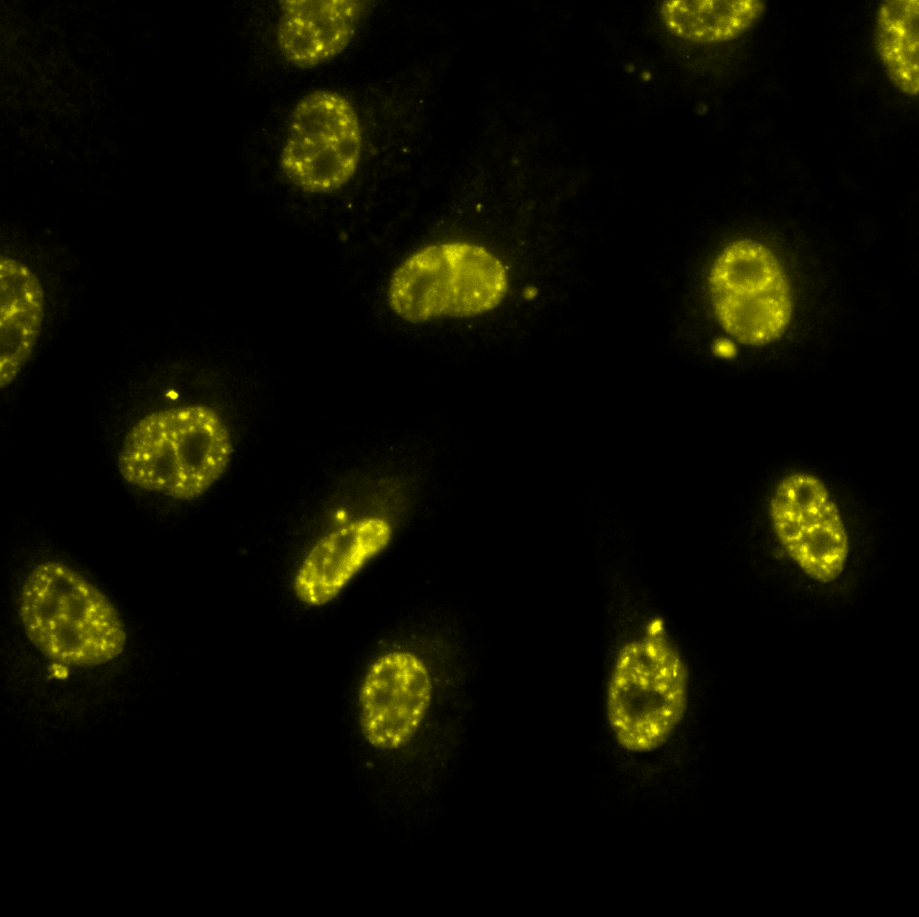

Supplement: Supplementary file 11 — Source data Fig. 7 [file 44318_2025_421_MOESM11_ESM.zip › Figure 7/Figure 7C/IFN DTX3L 2_Crop001_ch02_SV.tif]

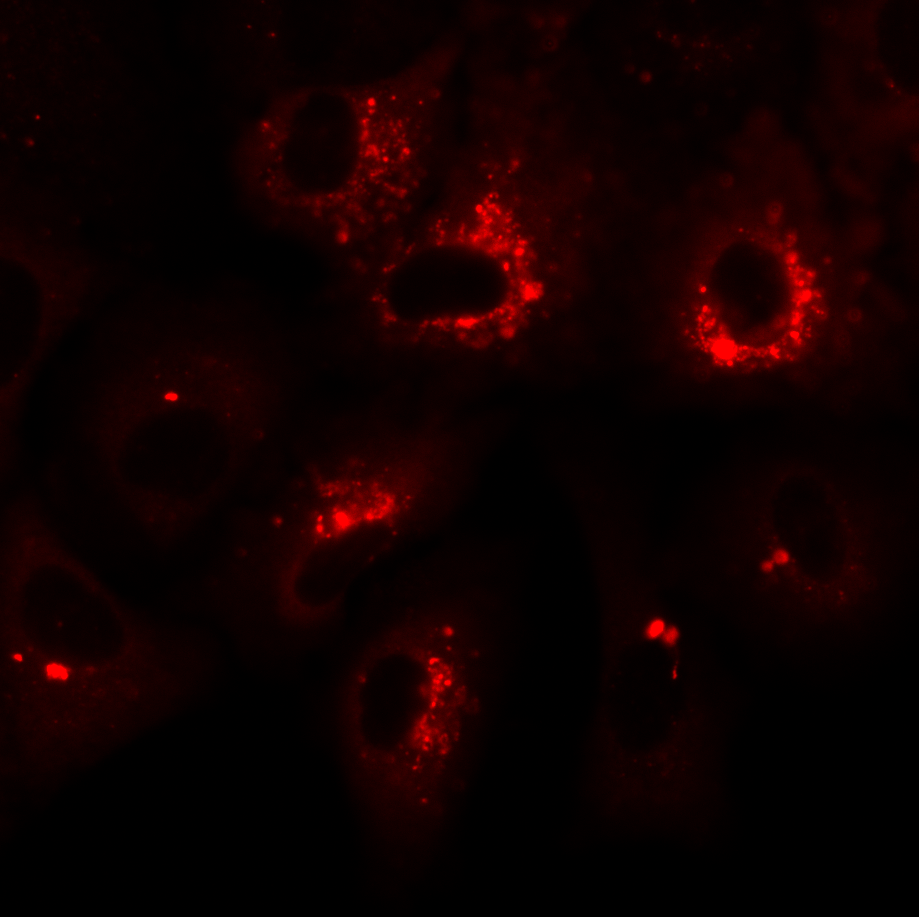

Supplement: Supplementary file 11 — Source data Fig. 7 [file 44318_2025_421_MOESM11_ESM.zip › Figure 7/Figure 7C/IFN DTX3L 2_Crop001_ch03_SV.tif]

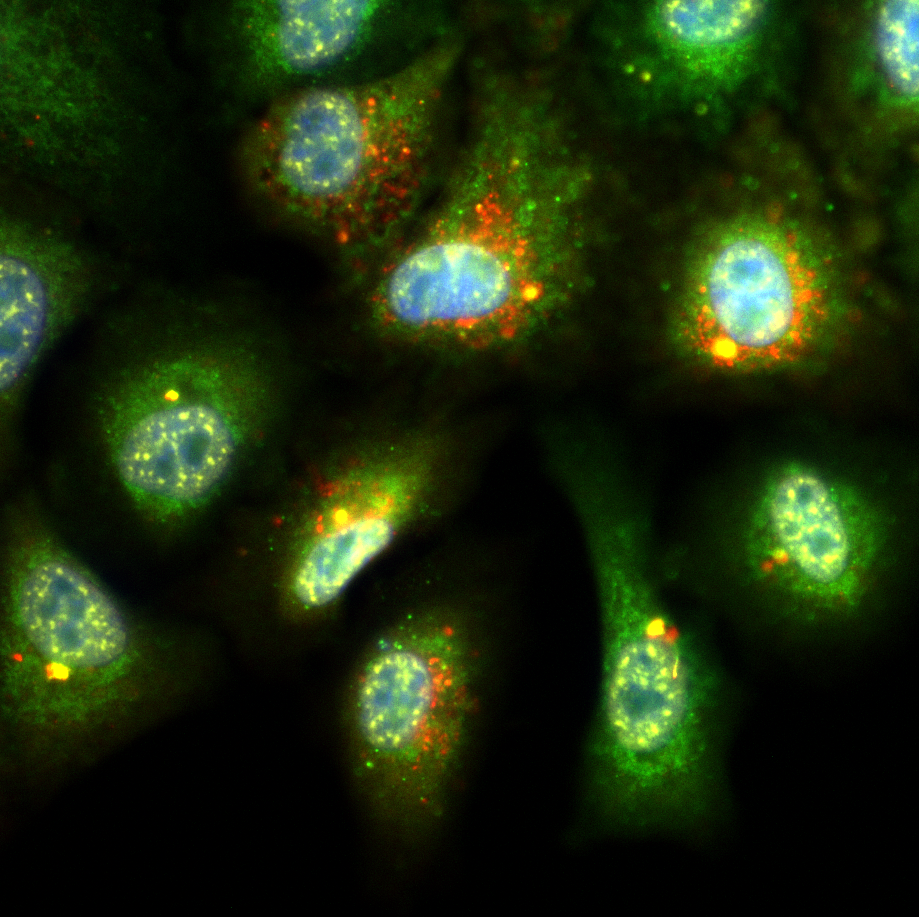

Supplement: Supplementary file 11 — Source data Fig. 7 [file 44318_2025_421_MOESM11_ESM.zip › Figure 7/Figure 7C/IFN DTX3L 2_Crop001_overlay.tif]

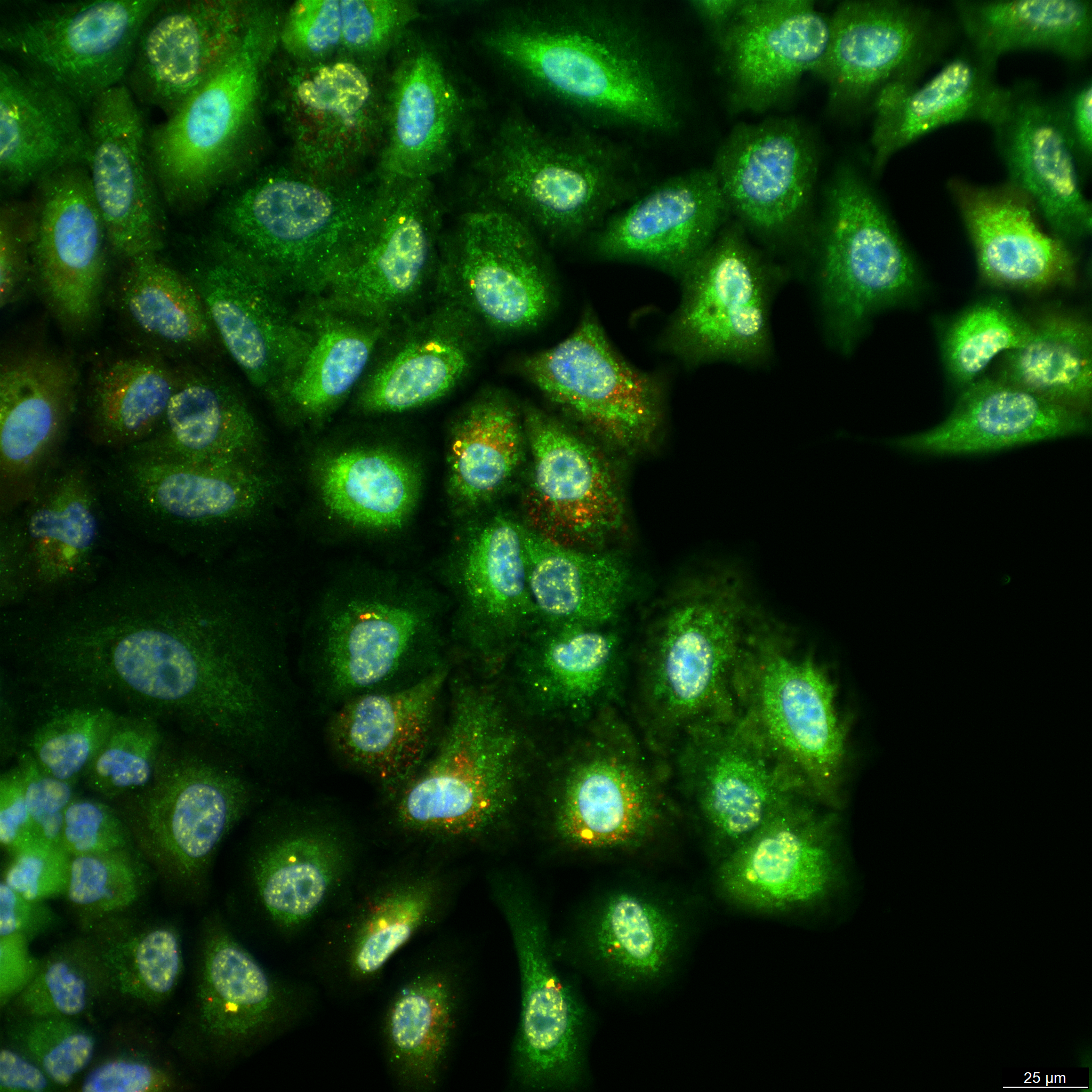

Supplement: Supplementary file 11 — Source data Fig. 7 [file 44318_2025_421_MOESM11_ESM.zip › Figure 7/Figure 7C/IFN Merge.tif]

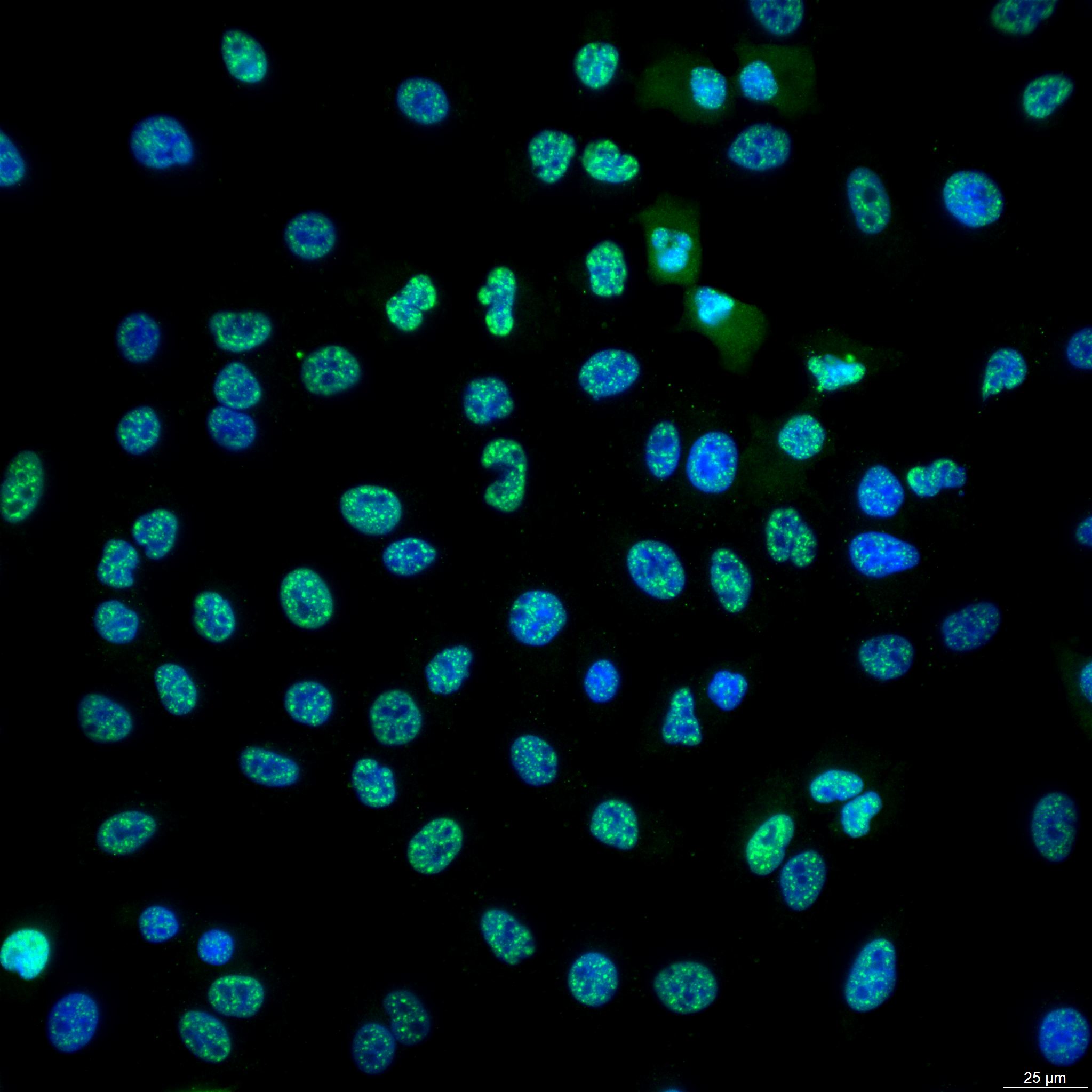

Supplement: Supplementary file 11 — Source data Fig. 7 [file 44318_2025_421_MOESM11_ESM.zip › Figure 7/Figure 7D/DTX3L KD.tif]

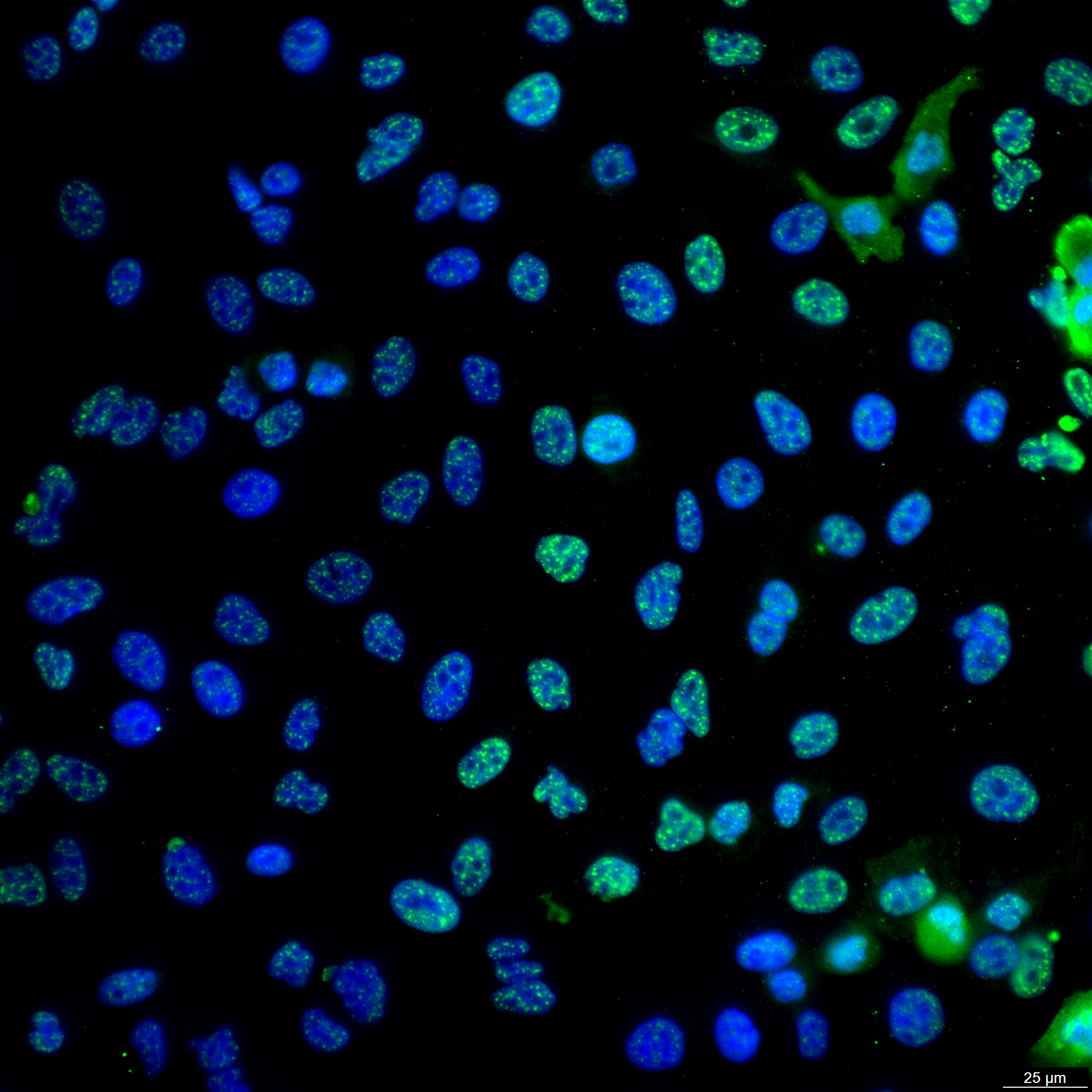

Supplement: Supplementary file 11 — Source data Fig. 7 [file 44318_2025_421_MOESM11_ESM.zip › Figure 7/Figure 7D/DTX3L+lFNγ.tif]

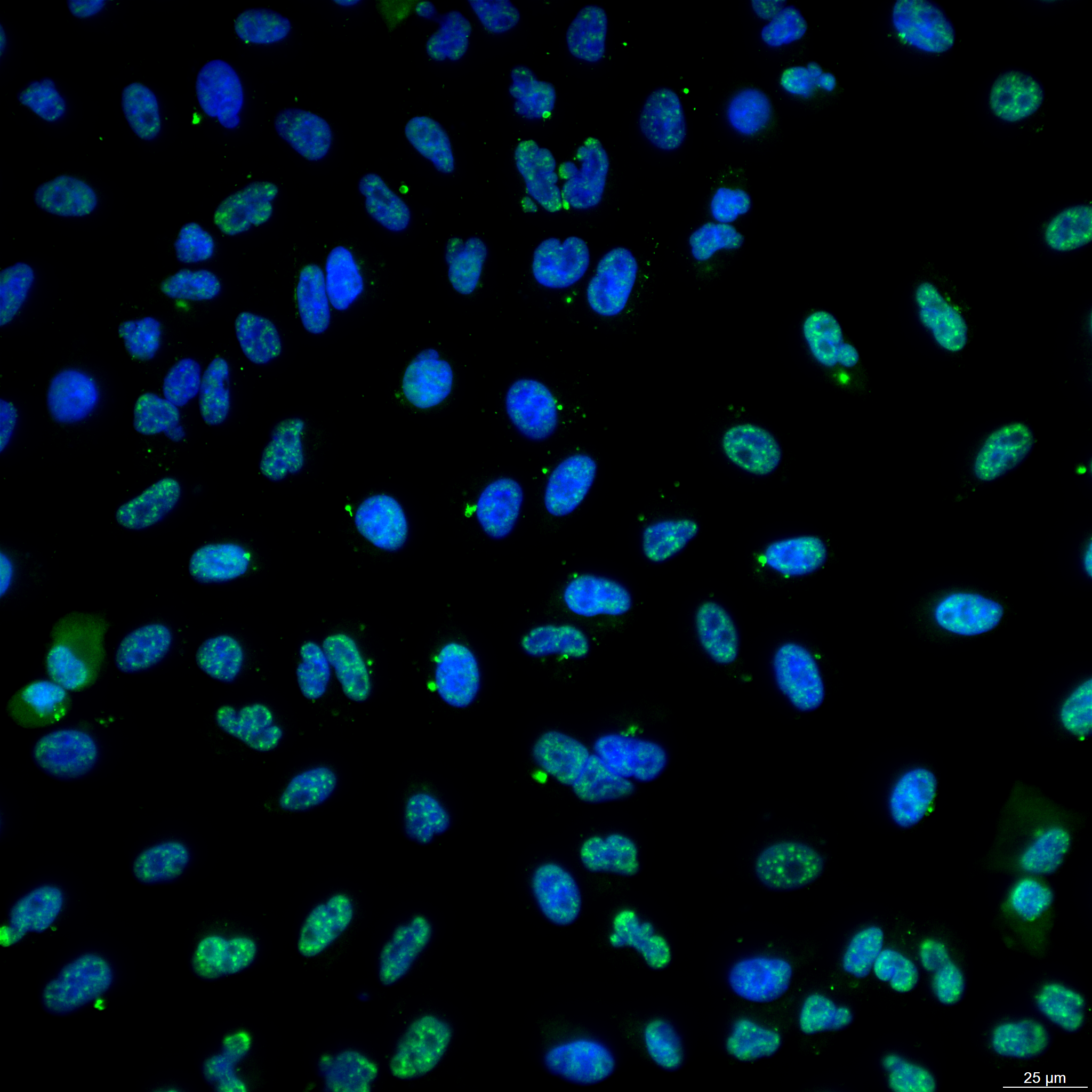

Supplement: Supplementary file 11 — Source data Fig. 7 [file 44318_2025_421_MOESM11_ESM.zip › Figure 7/Figure 7D/WT+lFNγ.tif]

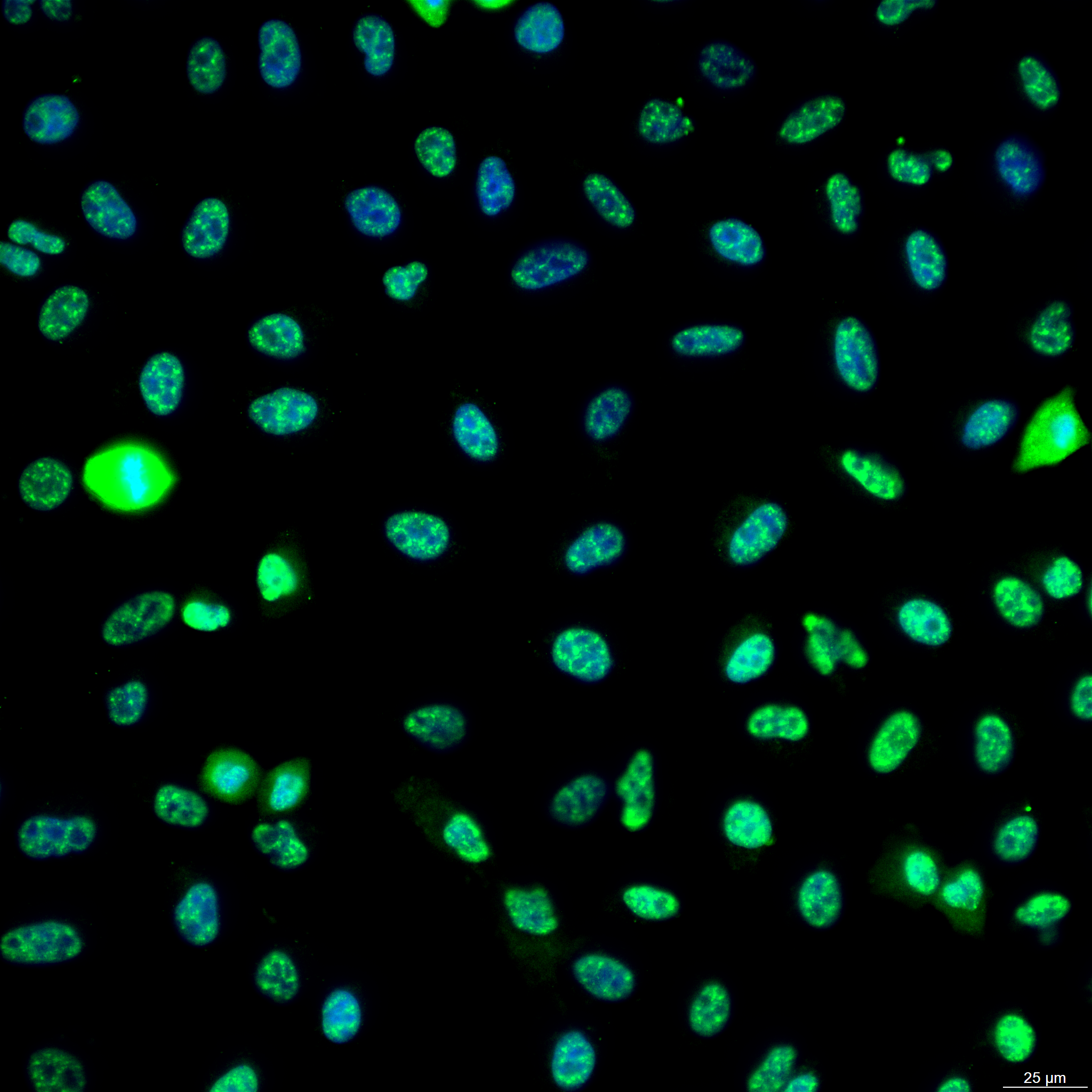

Supplement: Supplementary file 11 — Source data Fig. 7 [file 44318_2025_421_MOESM11_ESM.zip › Figure 7/Figure 7D/WT.tif]

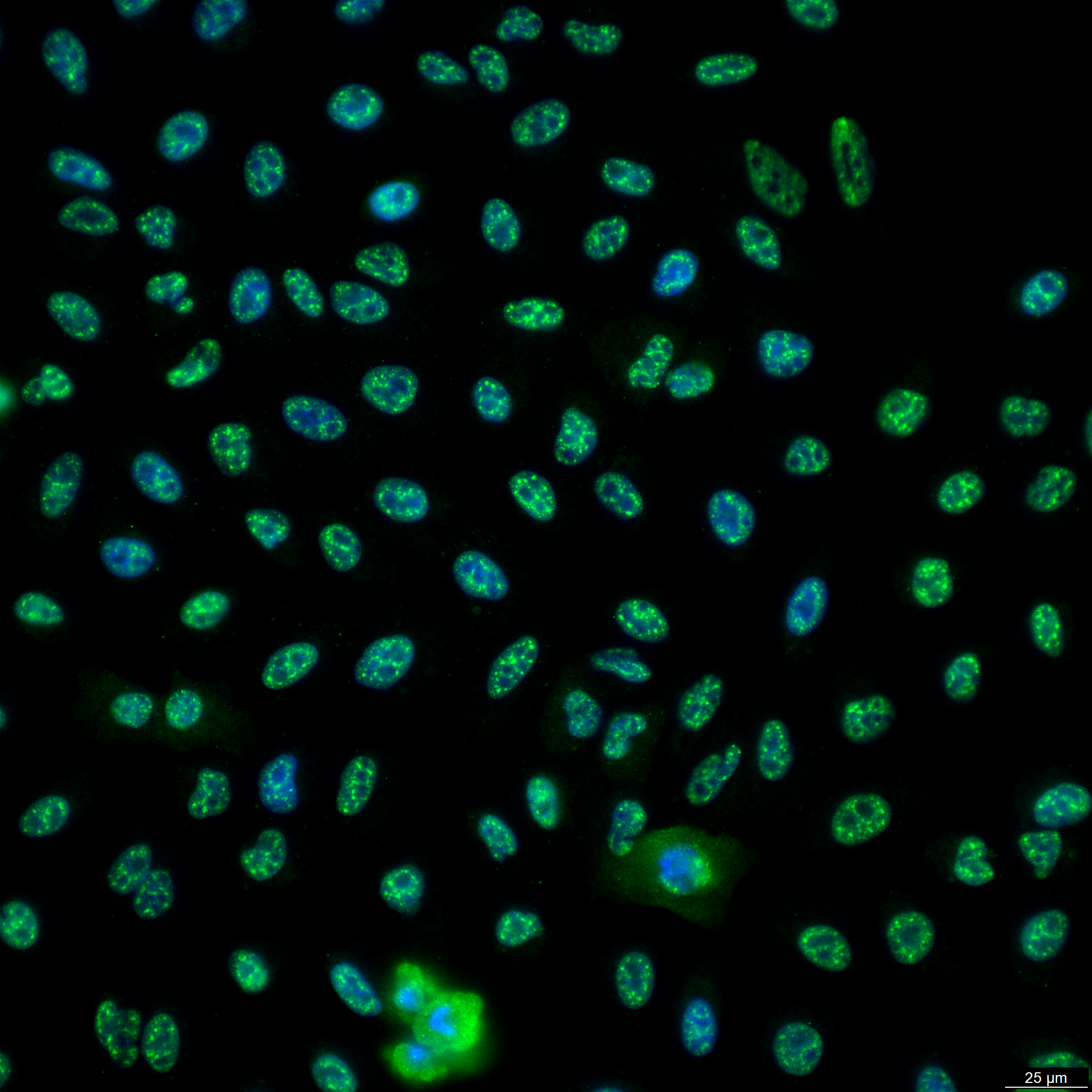

Supplement: Supplementary file 11 — Source data Fig. 7 [file 44318_2025_421_MOESM11_ESM.zip › Figure 7/Figure 7E/Control.tif]

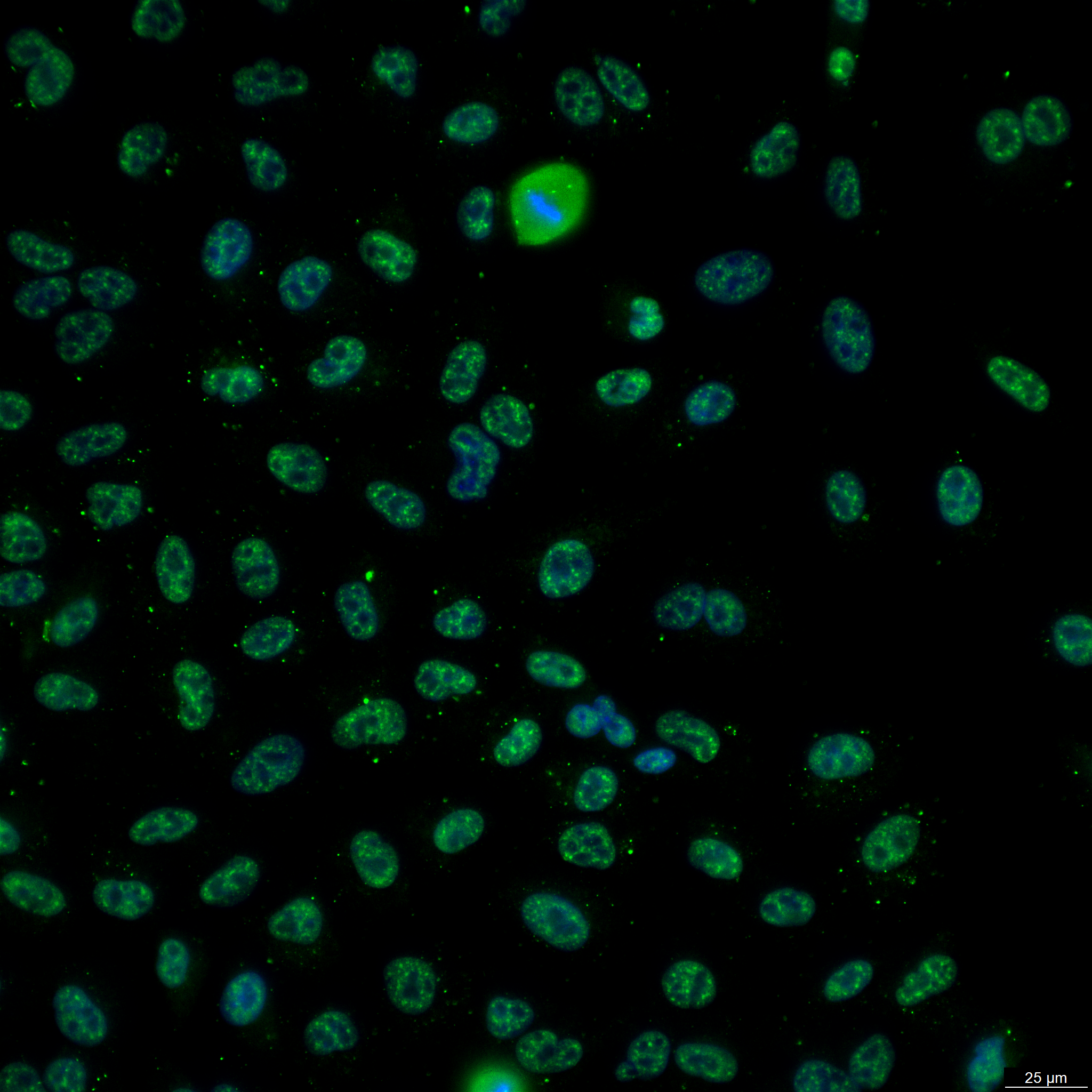

Supplement: Supplementary file 11 — Source data Fig. 7 [file 44318_2025_421_MOESM11_ESM.zip › Figure 7/Figure 7E/lFNγ+ PR619 25 μM.tif]

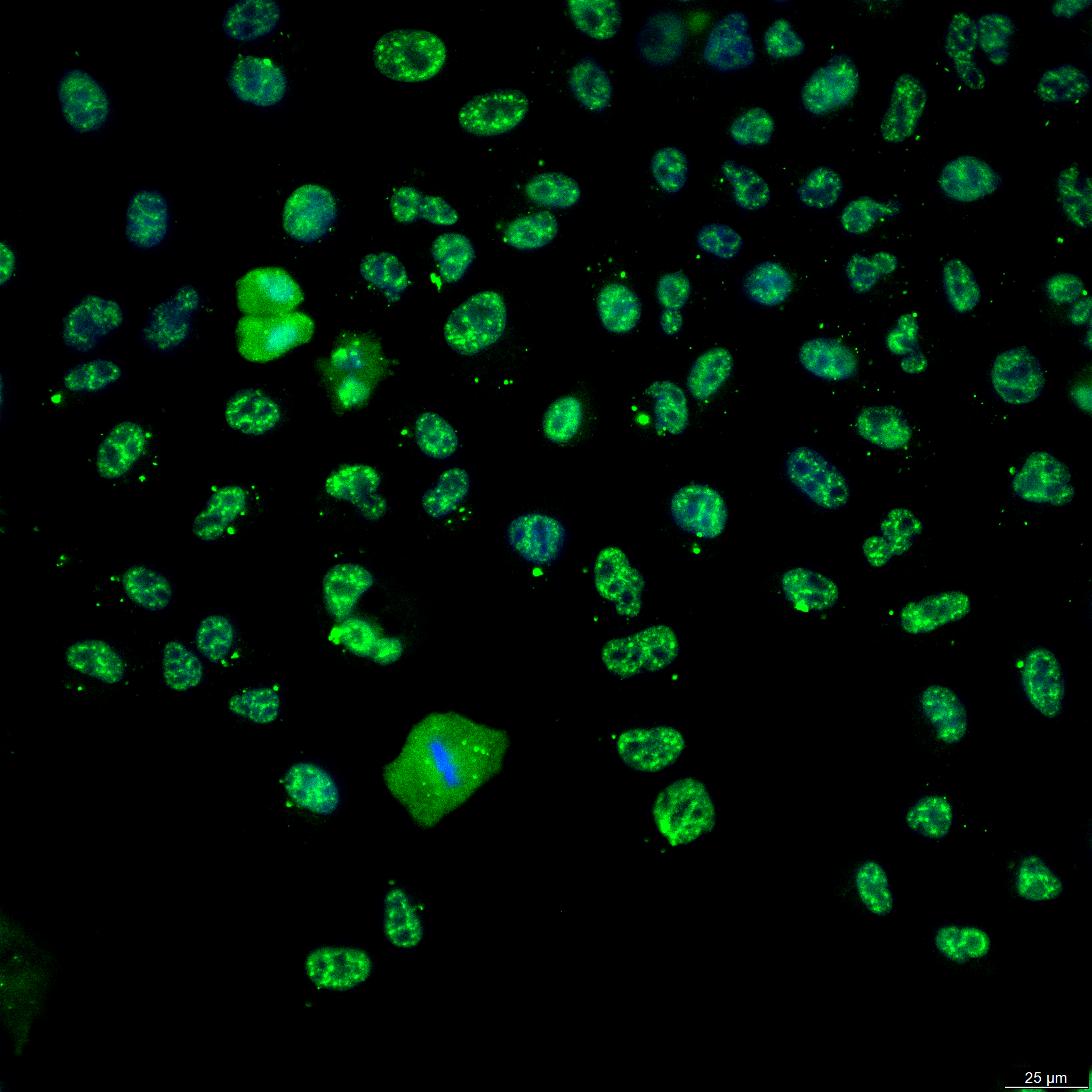

Supplement: Supplementary file 11 — Source data Fig. 7 [file 44318_2025_421_MOESM11_ESM.zip › Figure 7/Figure 7E/lFNγ+ PR619 50 μM.tif]

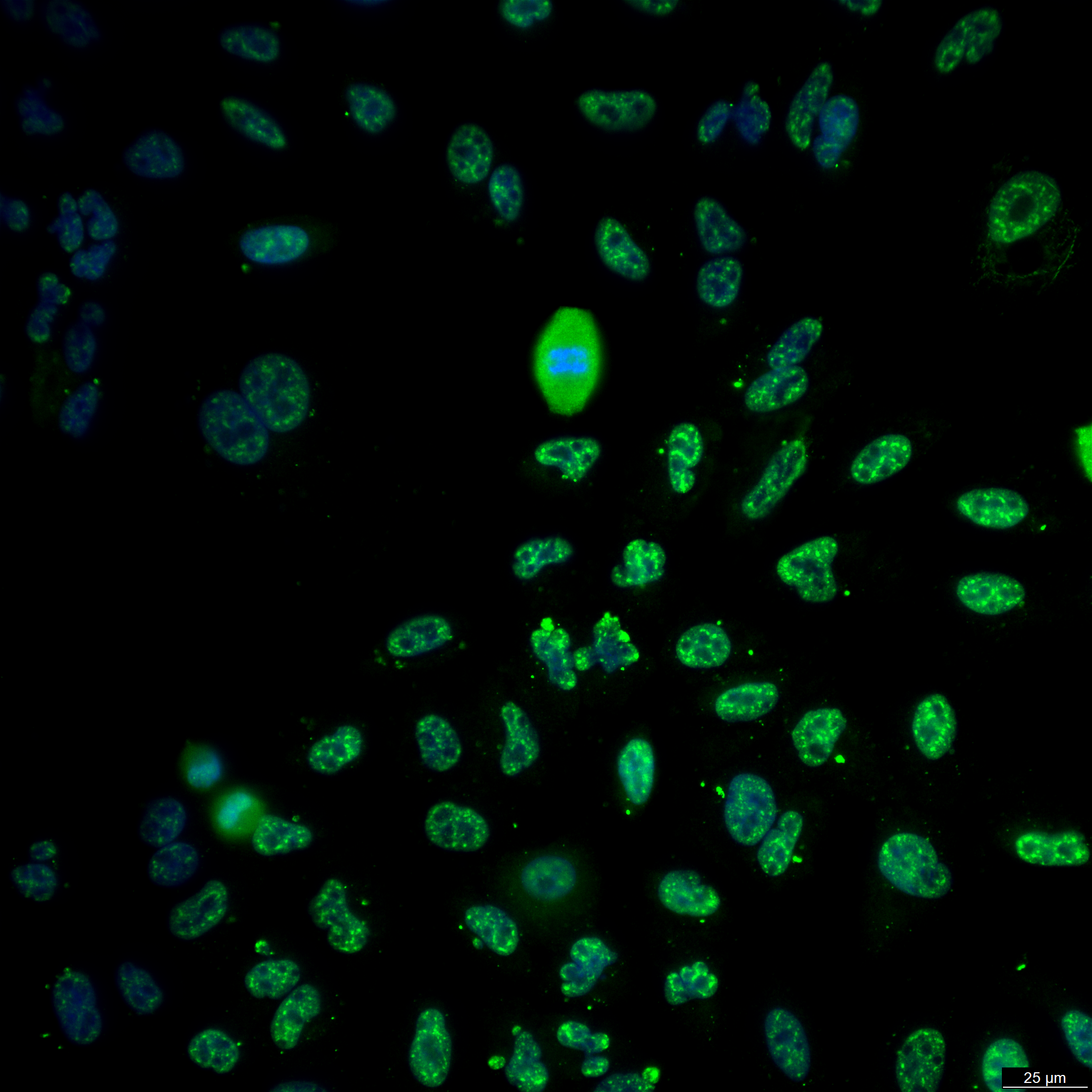

Supplement: Supplementary file 11 — Source data Fig. 7 [file 44318_2025_421_MOESM11_ESM.zip › Figure 7/Figure 7E/lFNγ+PR619 10 μM.tif]

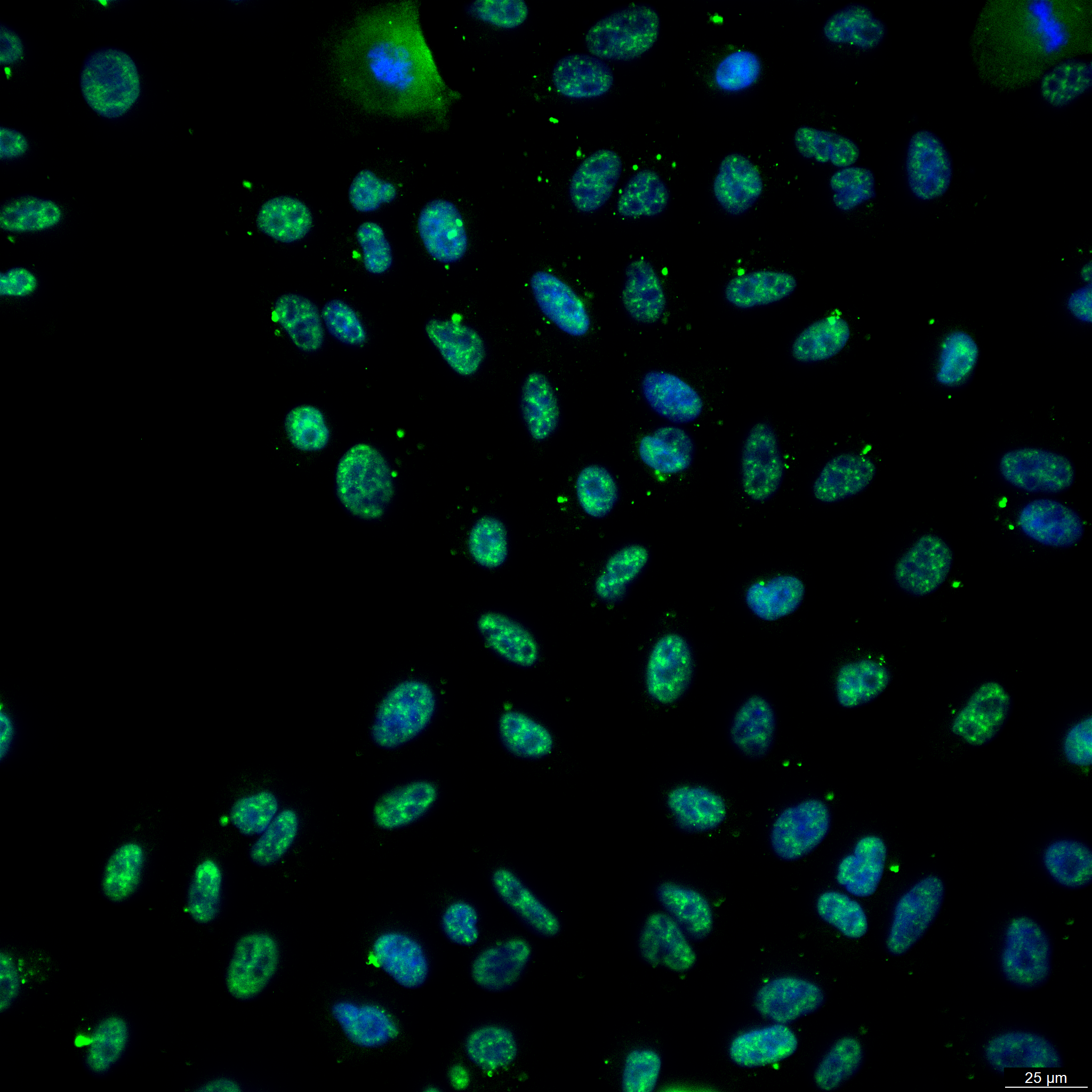

Supplement: Supplementary file 11 — Source data Fig. 7 [file 44318_2025_421_MOESM11_ESM.zip › Figure 7/Figure 7E/lFNγ+PR619 100 μM.tif]

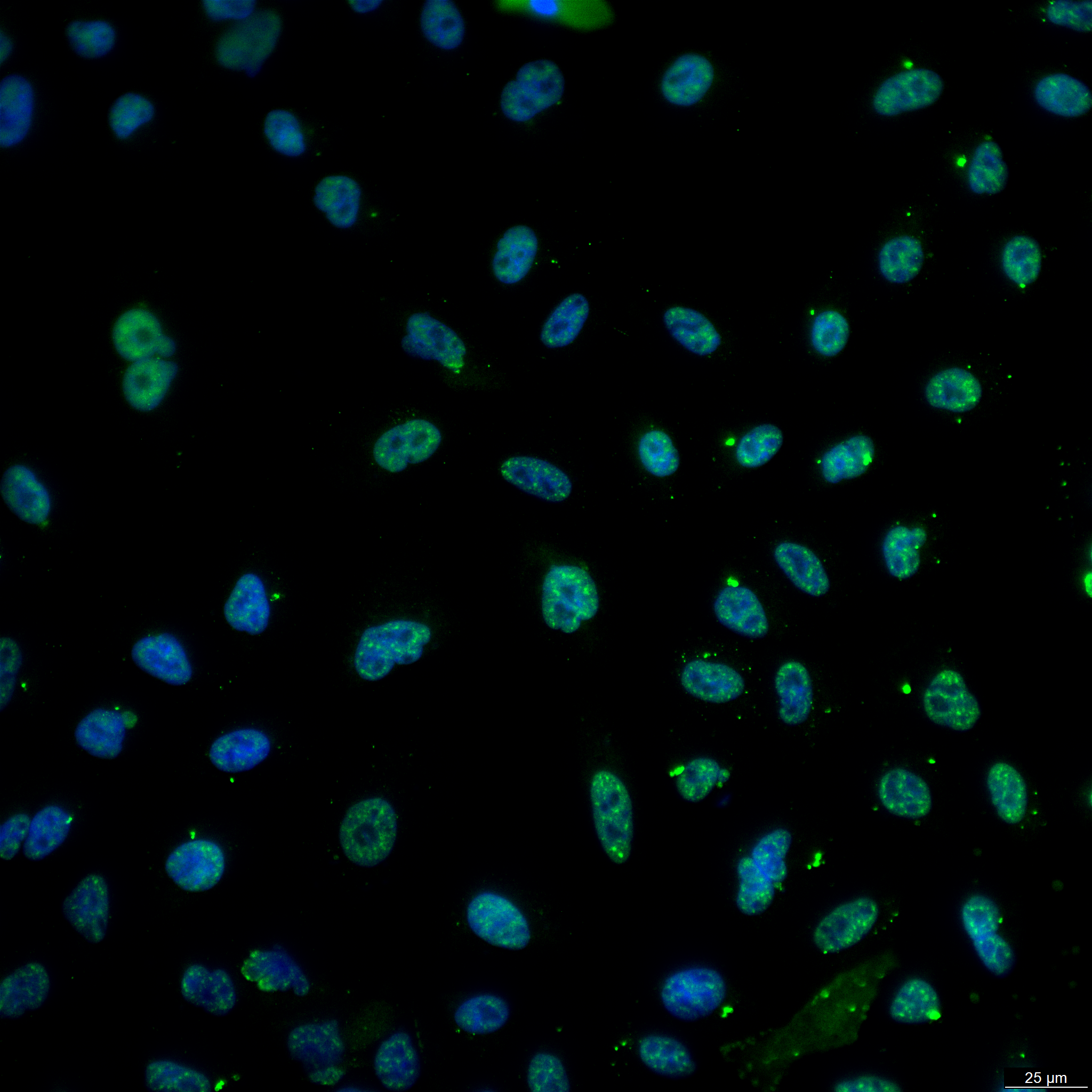

Supplement: Supplementary file 11 — Source data Fig. 7 [file 44318_2025_421_MOESM11_ESM.zip › Figure 7/Figure 7E/lFNγ.tif]

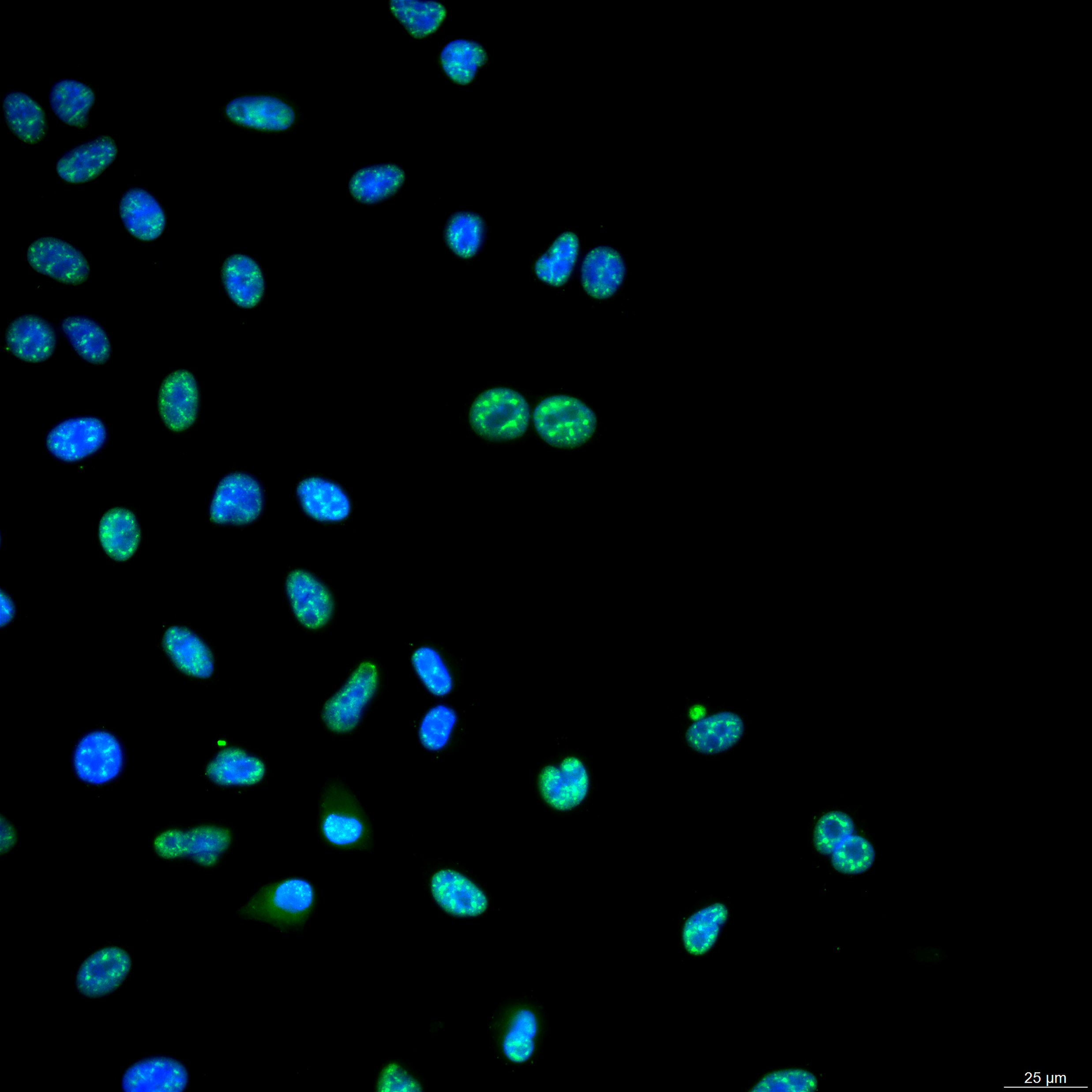

Supplement: Supplementary file 12 — Figure EV1 Source Data [file 44318_2025_421_MOESM12_ESM.zip › EV1/EV1A/Control.tif]

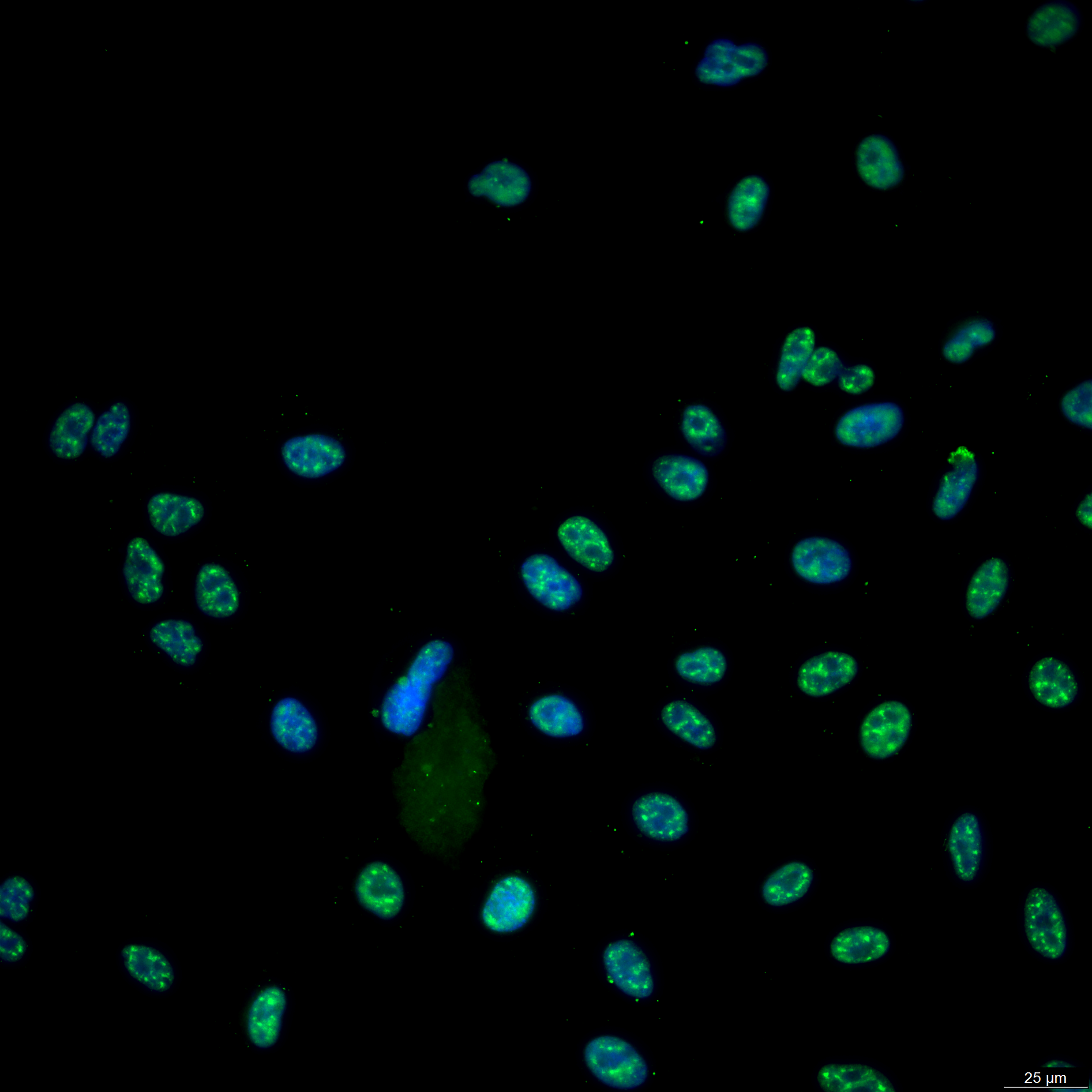

Supplement: Supplementary file 12 — Figure EV1 Source Data [file 44318_2025_421_MOESM12_ESM.zip › EV1/EV1A/IFNα 1000 IU.tif]

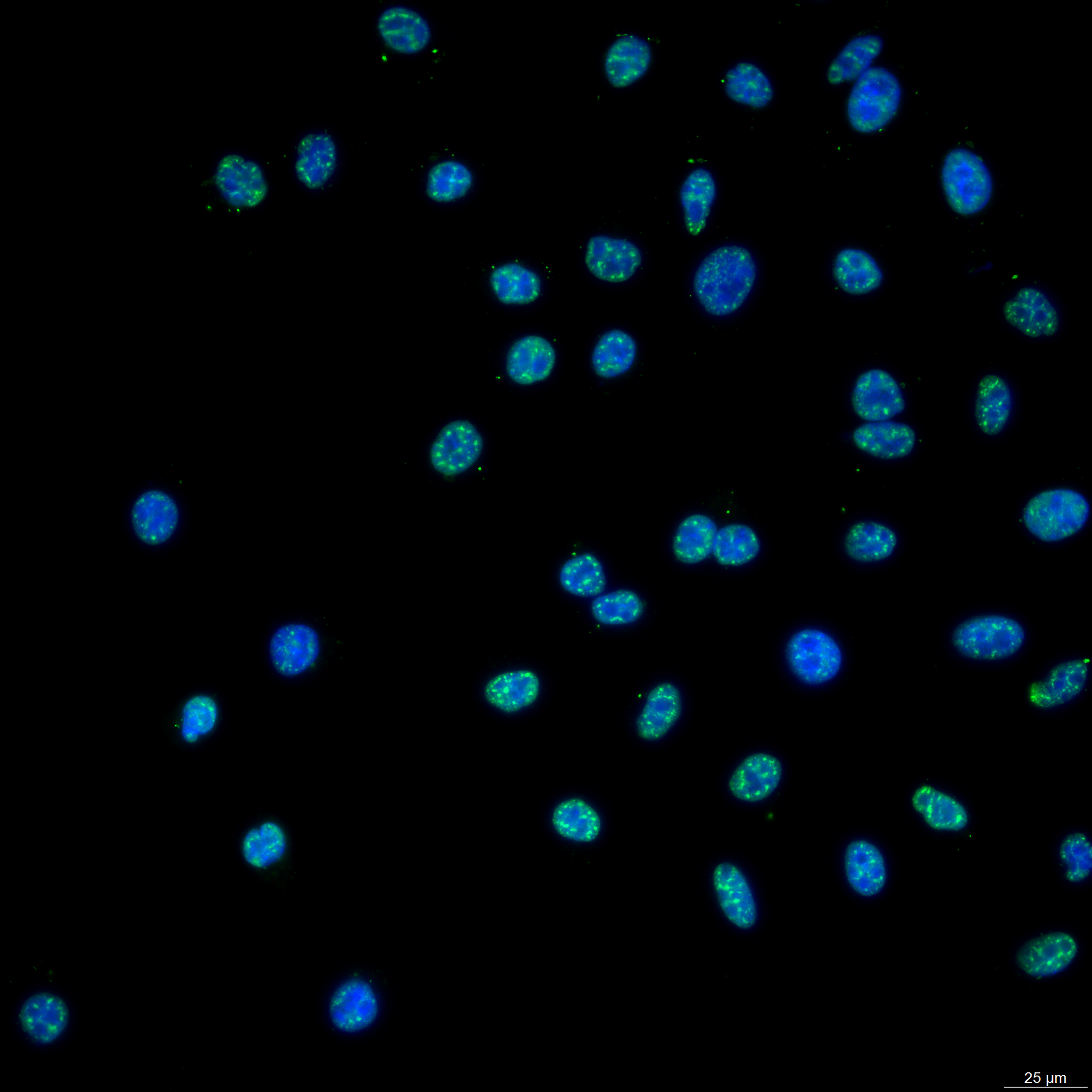

Supplement: Supplementary file 12 — Figure EV1 Source Data [file 44318_2025_421_MOESM12_ESM.zip › EV1/EV1A/IFNα 500 IU.tif]

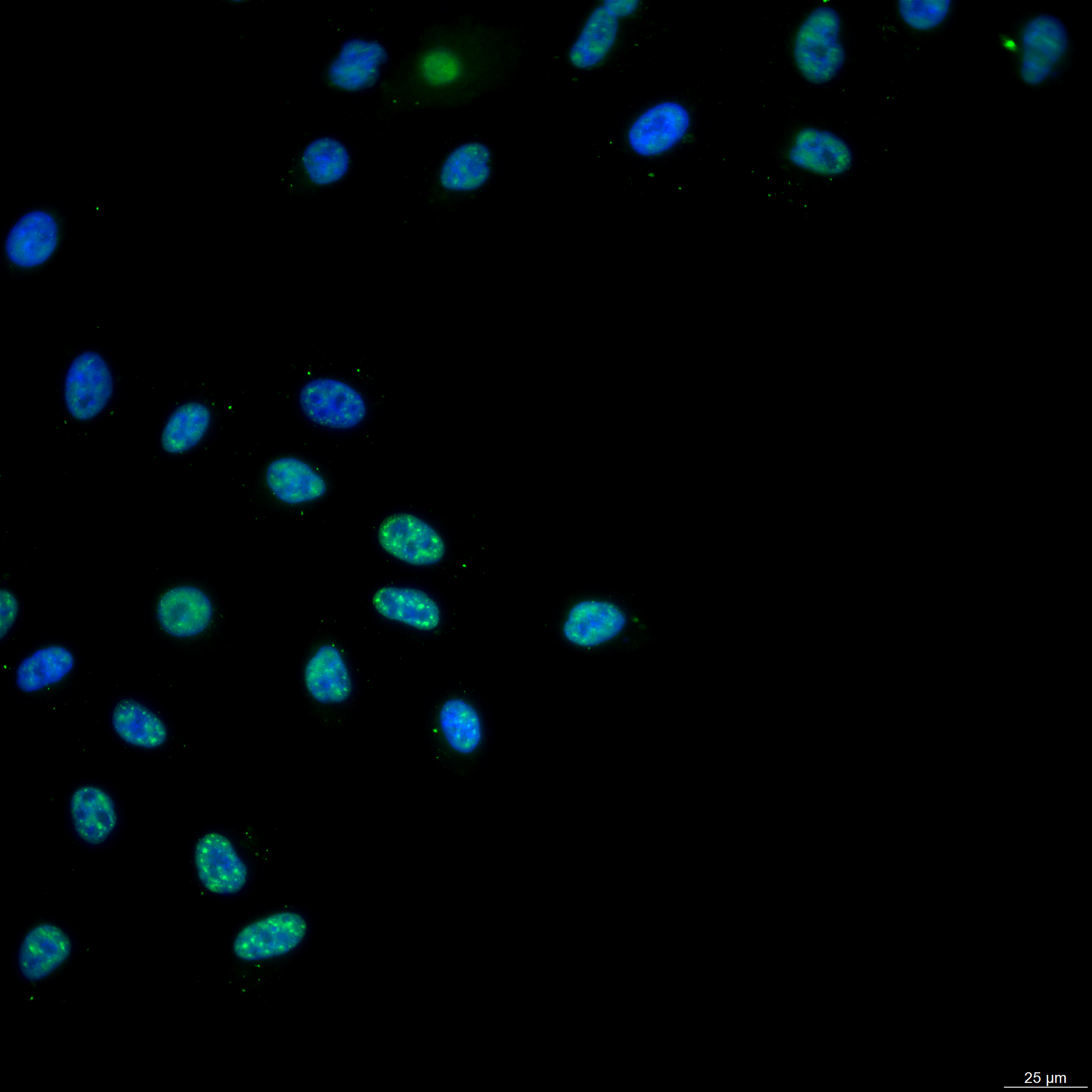

Supplement: Supplementary file 12 — Figure EV1 Source Data [file 44318_2025_421_MOESM12_ESM.zip › EV1/EV1A/IFNβ 1000 IU.tif]

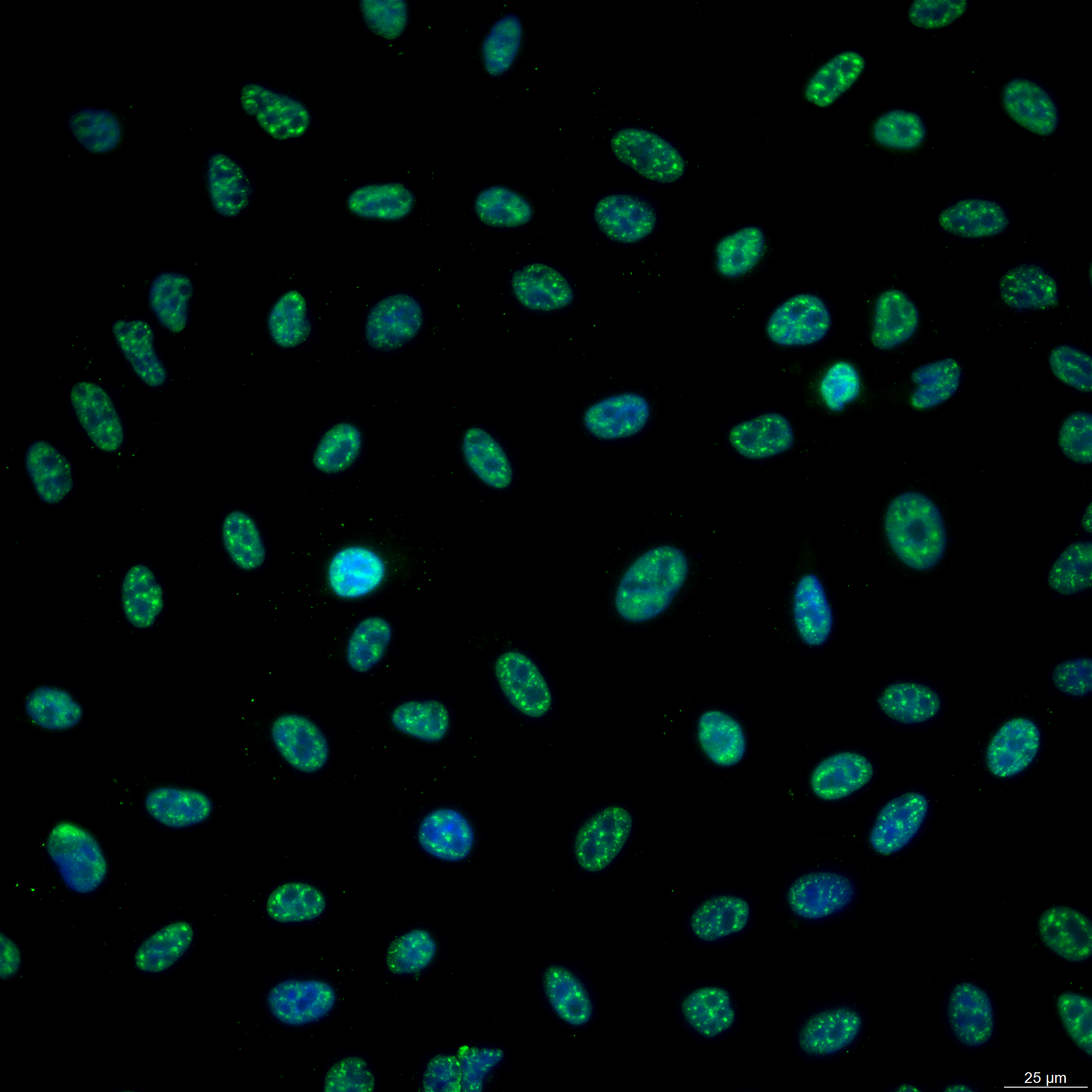

Supplement: Supplementary file 12 — Figure EV1 Source Data [file 44318_2025_421_MOESM12_ESM.zip › EV1/EV1A/IFNβ 500 IU.tif]

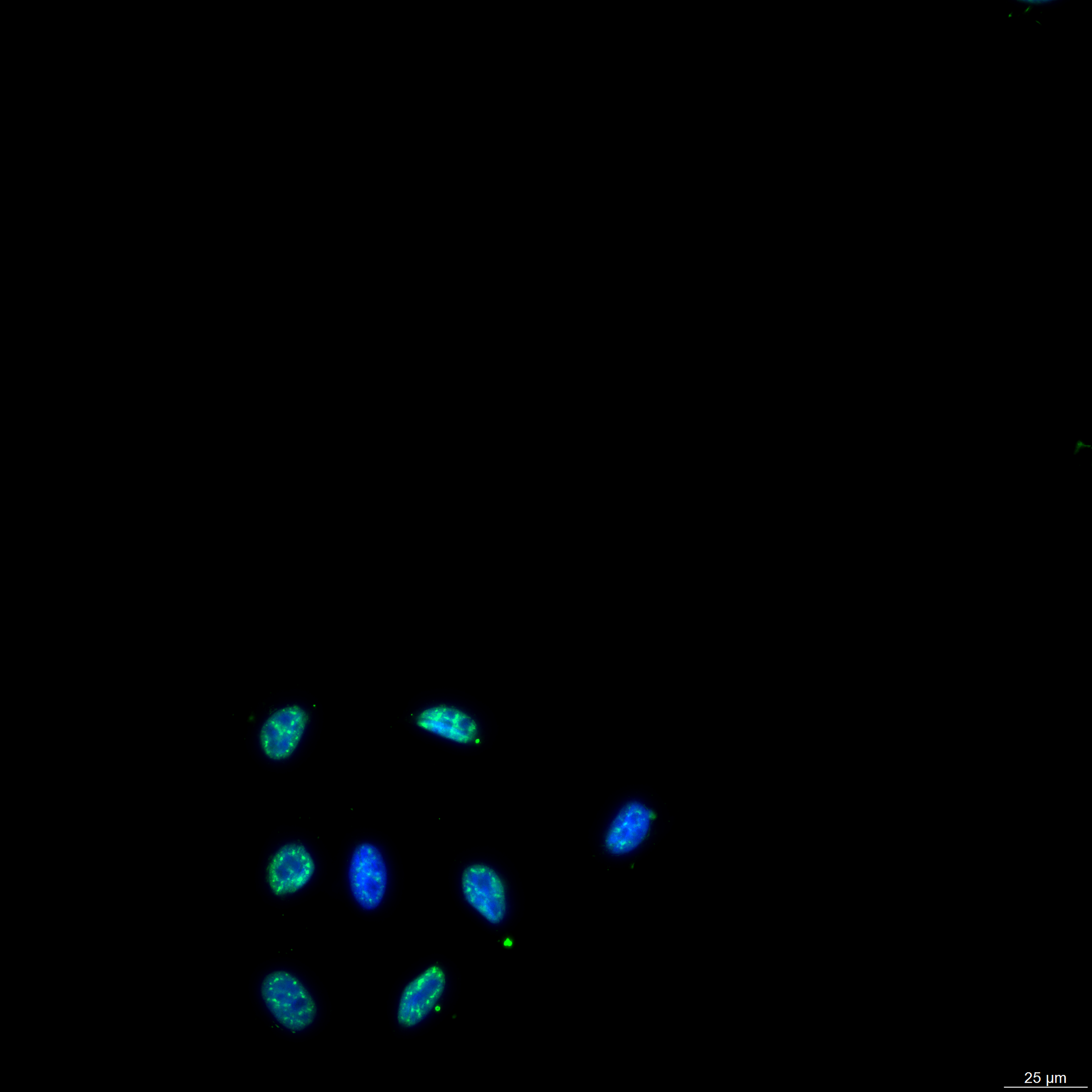

Supplement: Supplementary file 12 — Figure EV1 Source Data [file 44318_2025_421_MOESM12_ESM.zip › EV1/EV1A/IFNγ 1000 IU.tif]

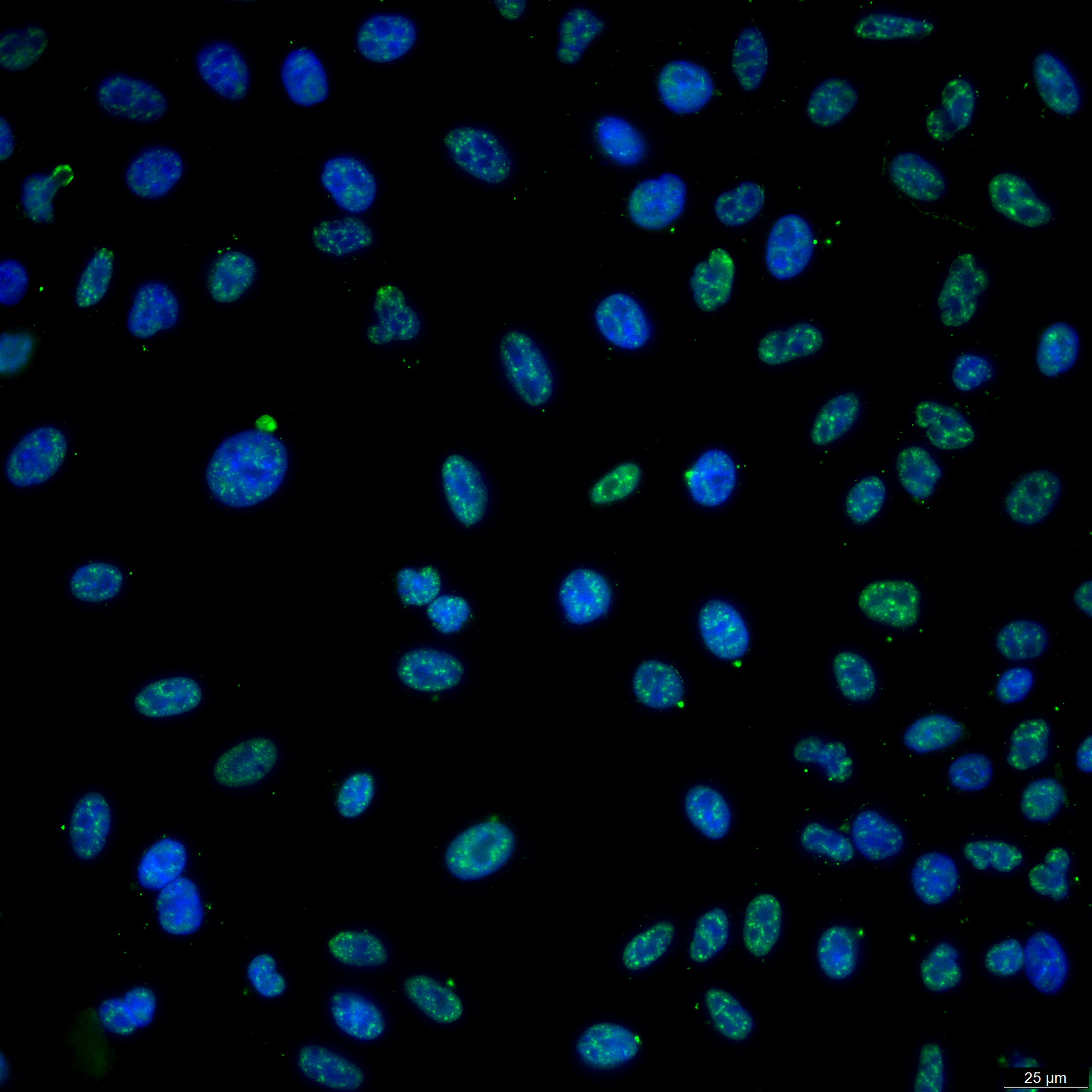

Supplement: Supplementary file 12 — Figure EV1 Source Data [file 44318_2025_421_MOESM12_ESM.zip › EV1/EV1A/IFNγ 500 IU.tif]

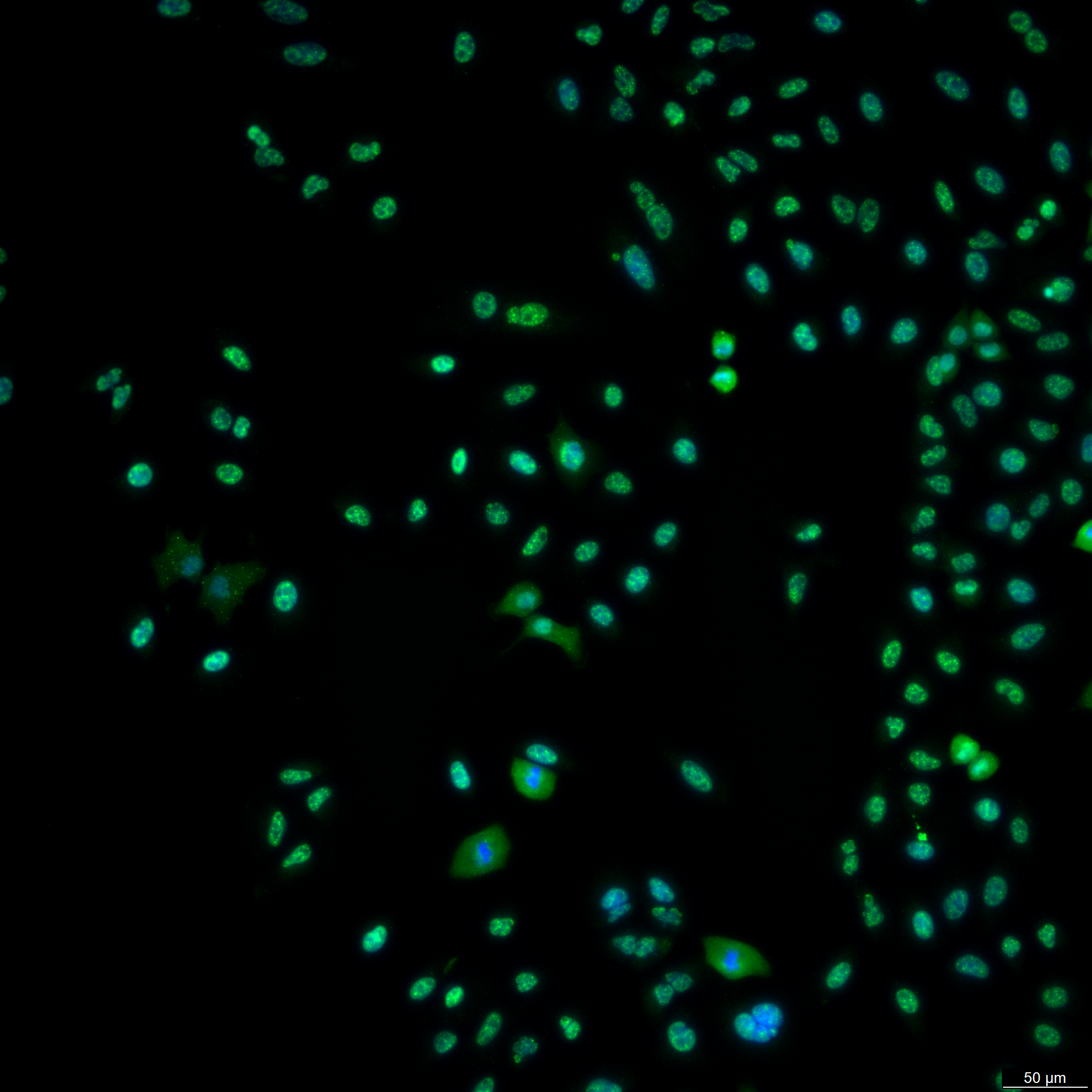

Supplement: Supplementary file 12 — Figure EV1 Source Data [file 44318_2025_421_MOESM12_ESM.zip › EV1/EV1B/IFN 0 HR .tif]

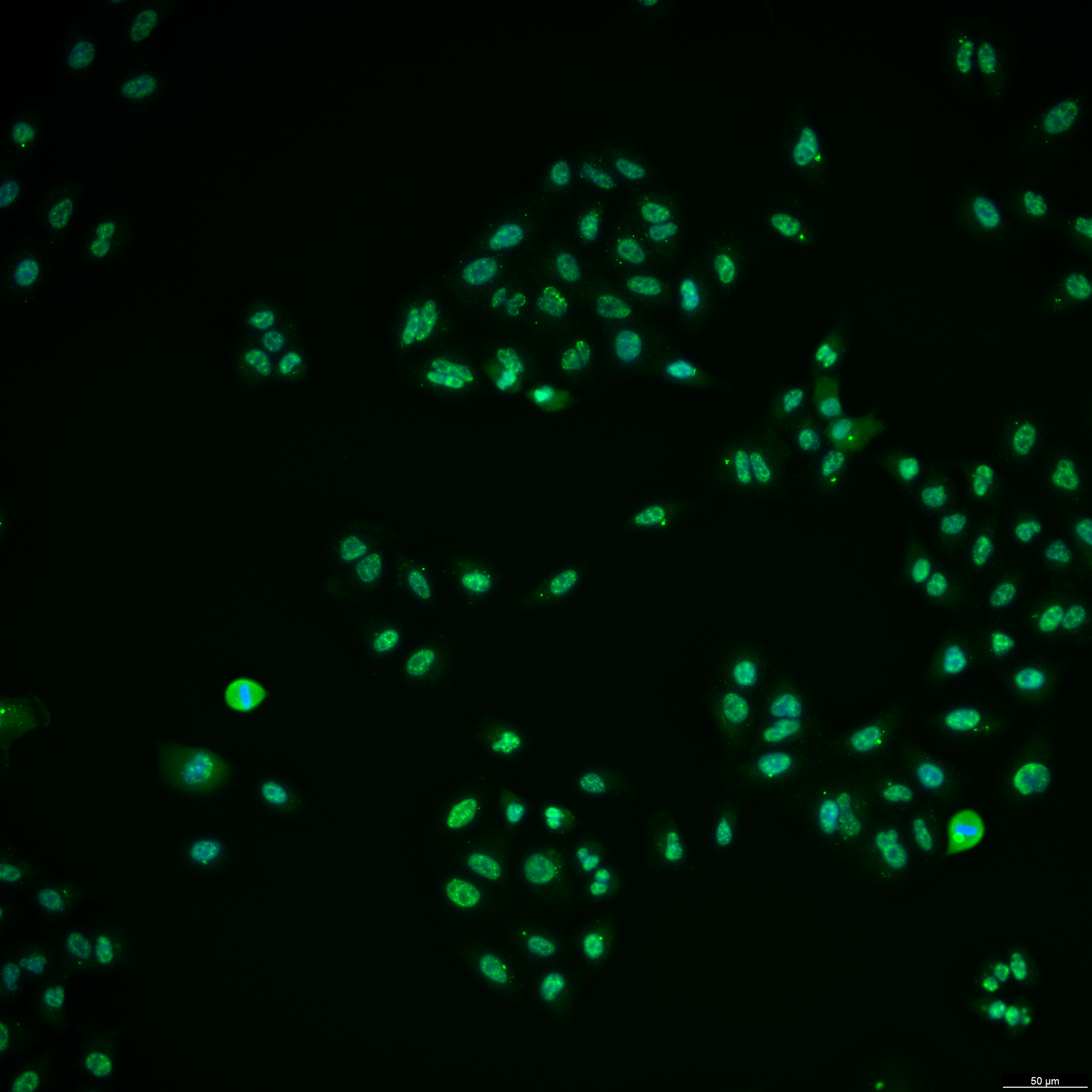

Supplement: Supplementary file 12 — Figure EV1 Source Data [file 44318_2025_421_MOESM12_ESM.zip › EV1/EV1B/IFN 10 HR .tif]

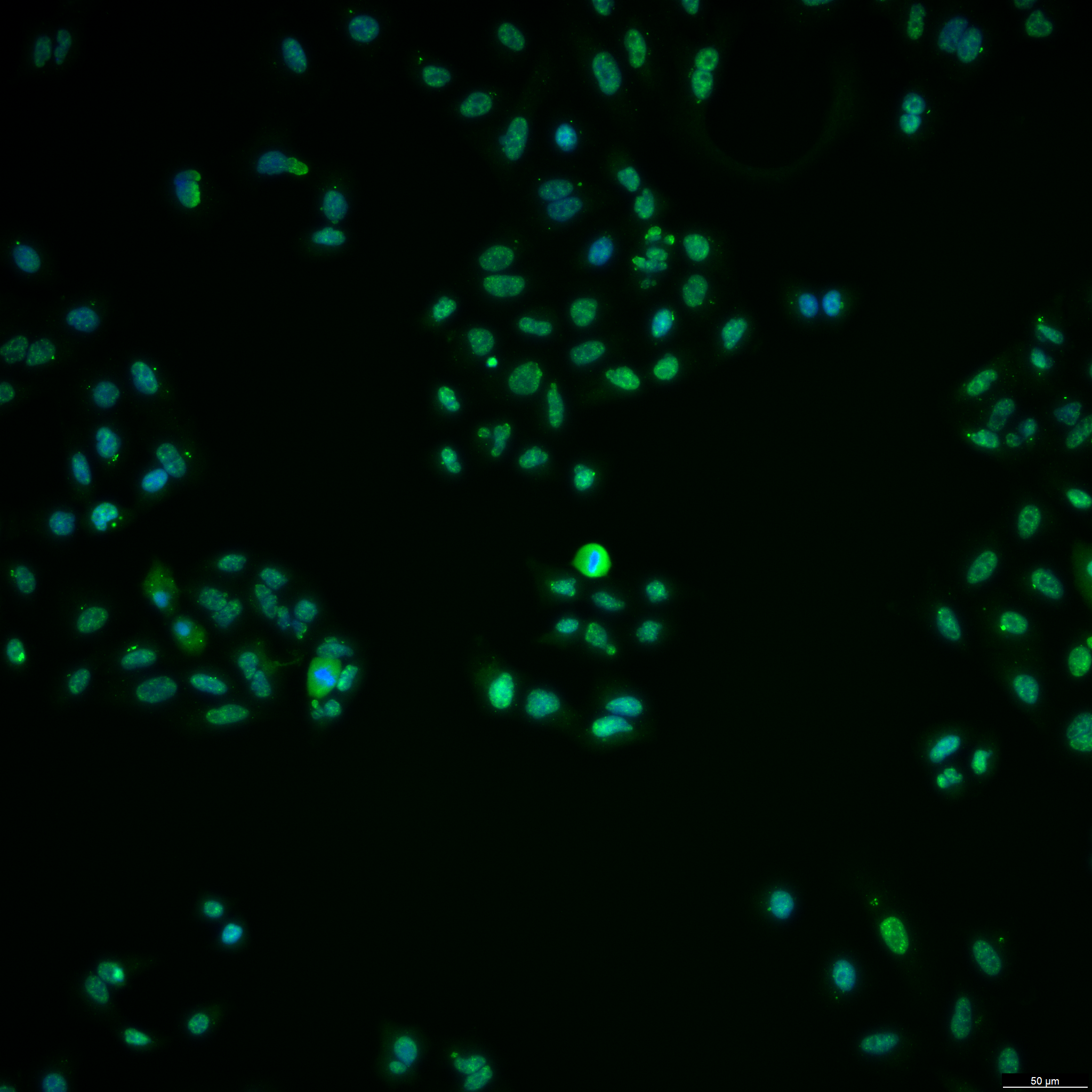

Supplement: Supplementary file 12 — Figure EV1 Source Data [file 44318_2025_421_MOESM12_ESM.zip › EV1/EV1B/IFN 16 HR .tif]

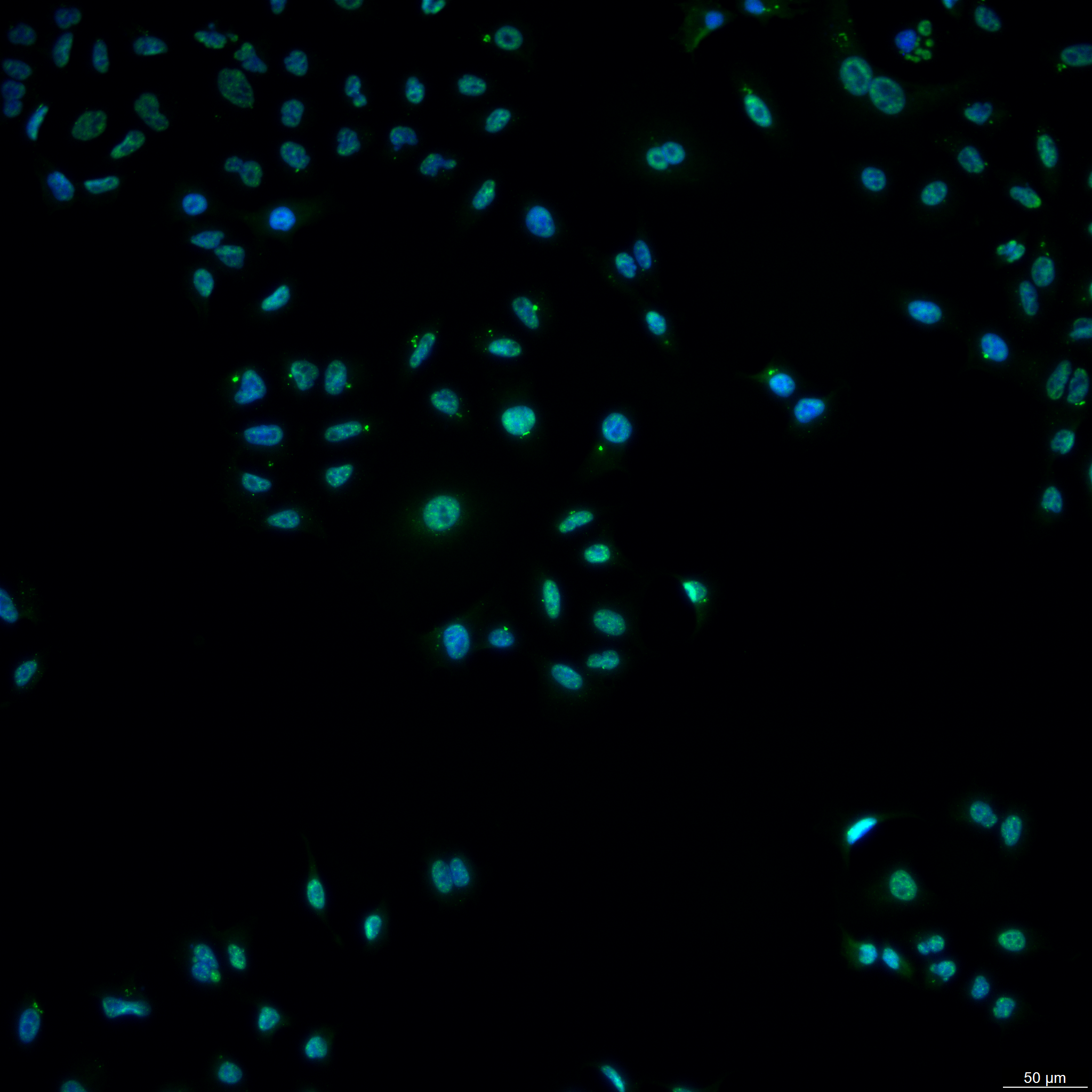

Supplement: Supplementary file 12 — Figure EV1 Source Data [file 44318_2025_421_MOESM12_ESM.zip › EV1/EV1B/IFN 1HR .tif]

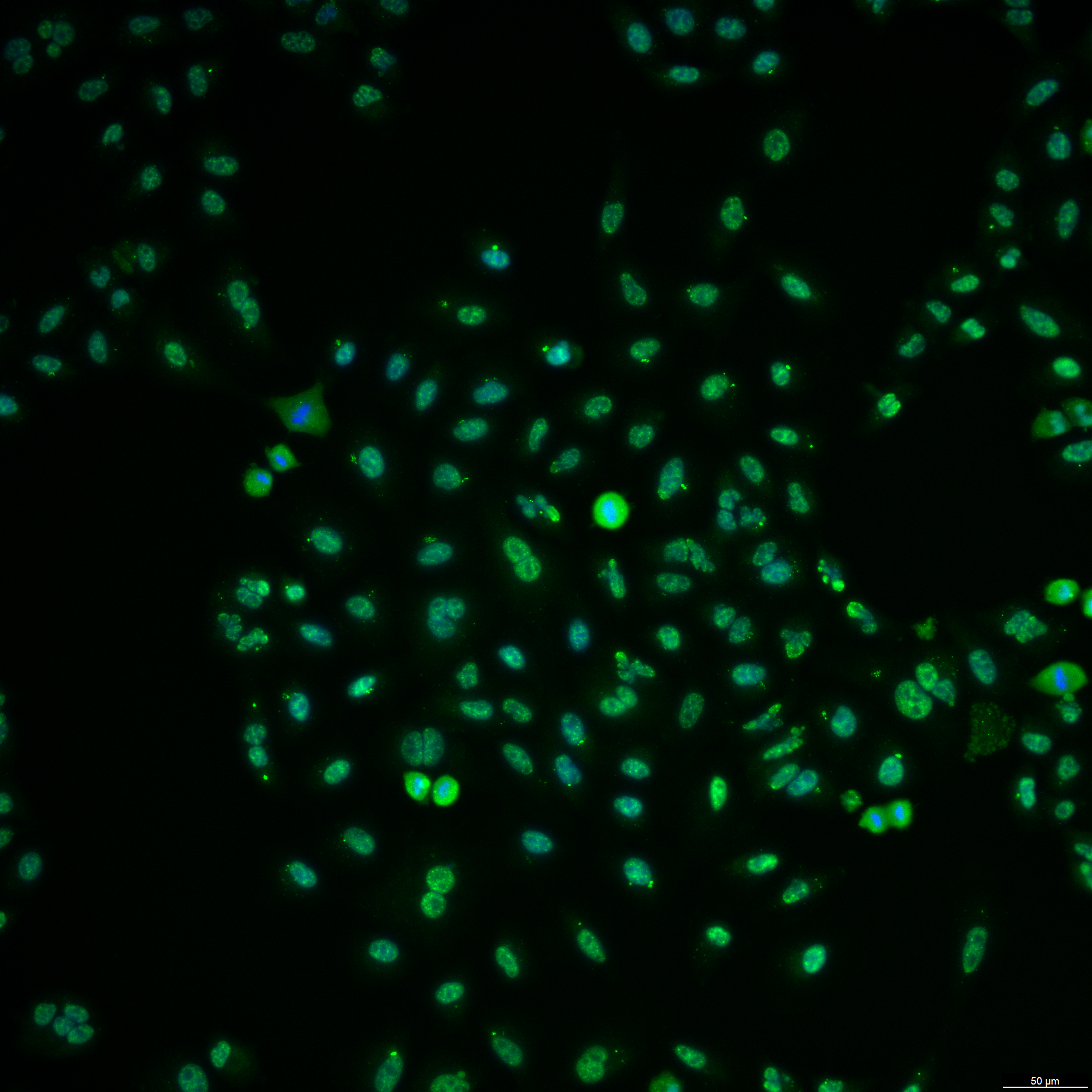

Supplement: Supplementary file 12 — Figure EV1 Source Data [file 44318_2025_421_MOESM12_ESM.zip › EV1/EV1B/IFN 24 HR .tif]

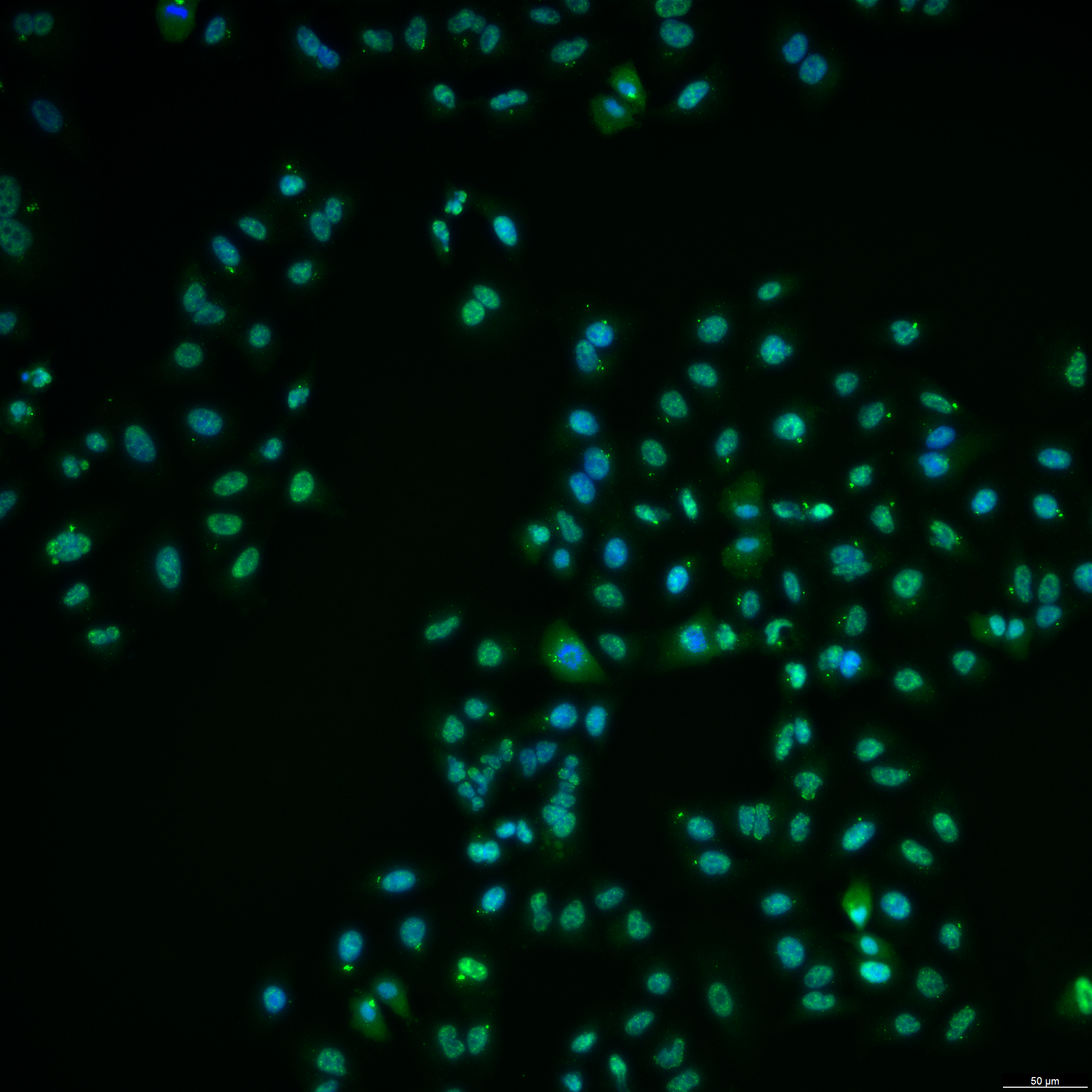

Supplement: Supplementary file 12 — Figure EV1 Source Data [file 44318_2025_421_MOESM12_ESM.zip › EV1/EV1B/IFN 3HR .tif]

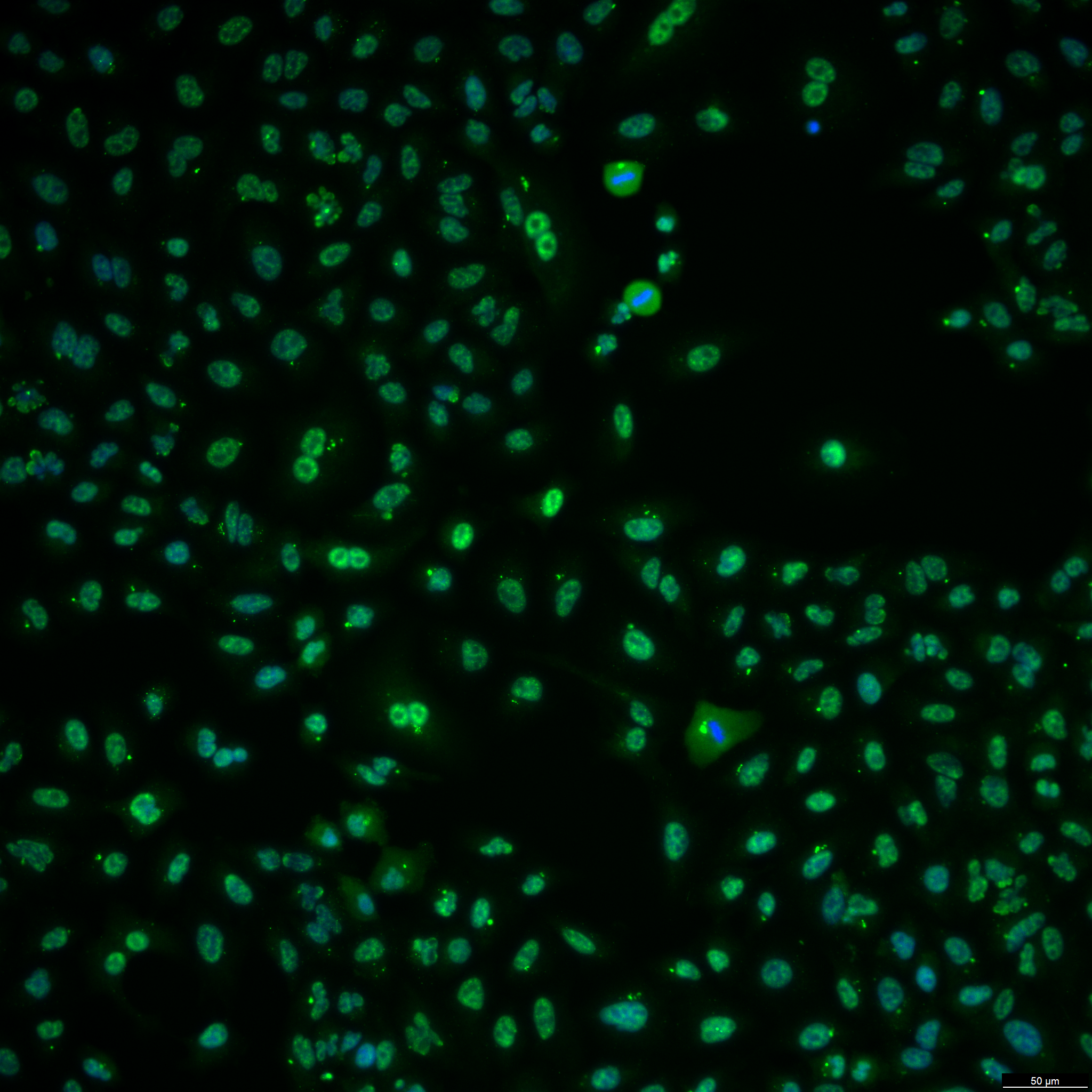

Supplement: Supplementary file 12 — Figure EV1 Source Data [file 44318_2025_421_MOESM12_ESM.zip › EV1/EV1B/IFN 6HR .tif]

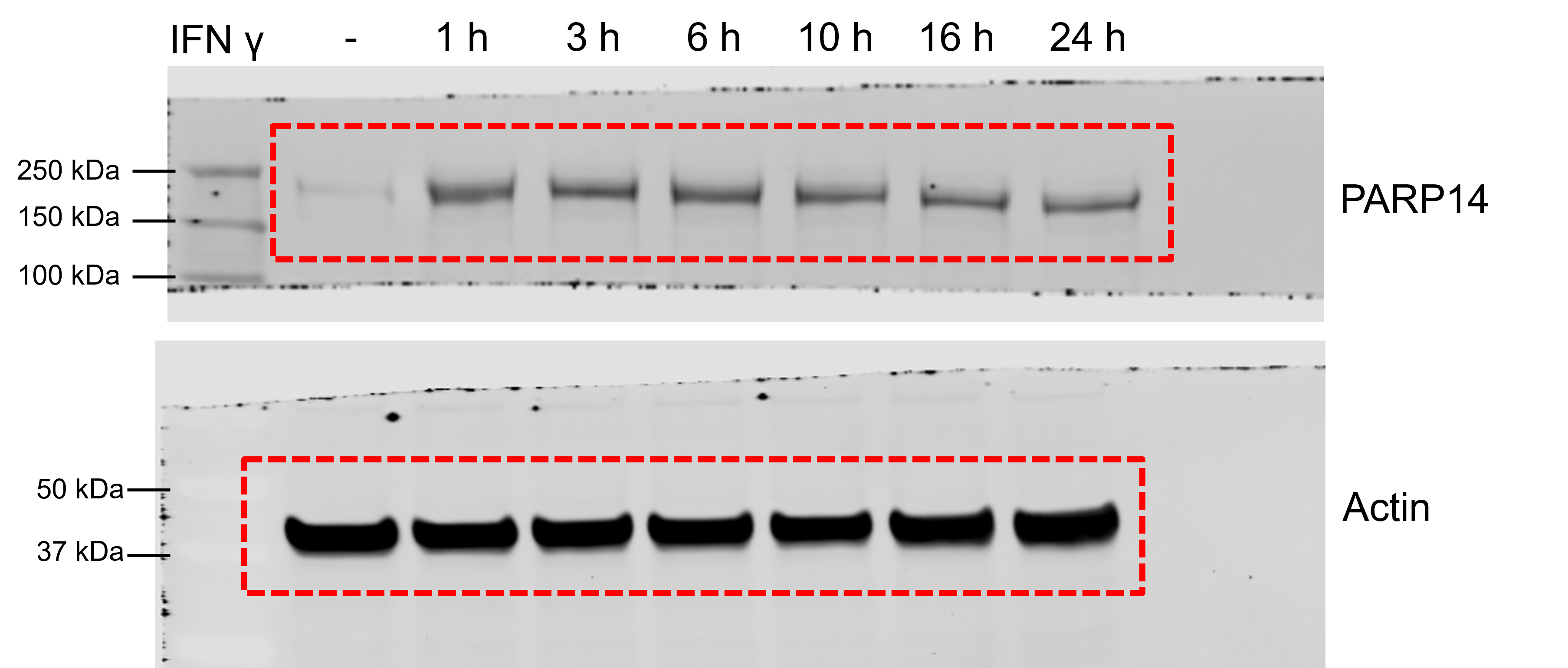

Supplement: Supplementary file 12 — Figure EV1 Source Data [file 44318_2025_421_MOESM12_ESM.zip › EV1/EV1E lower panel.tif]

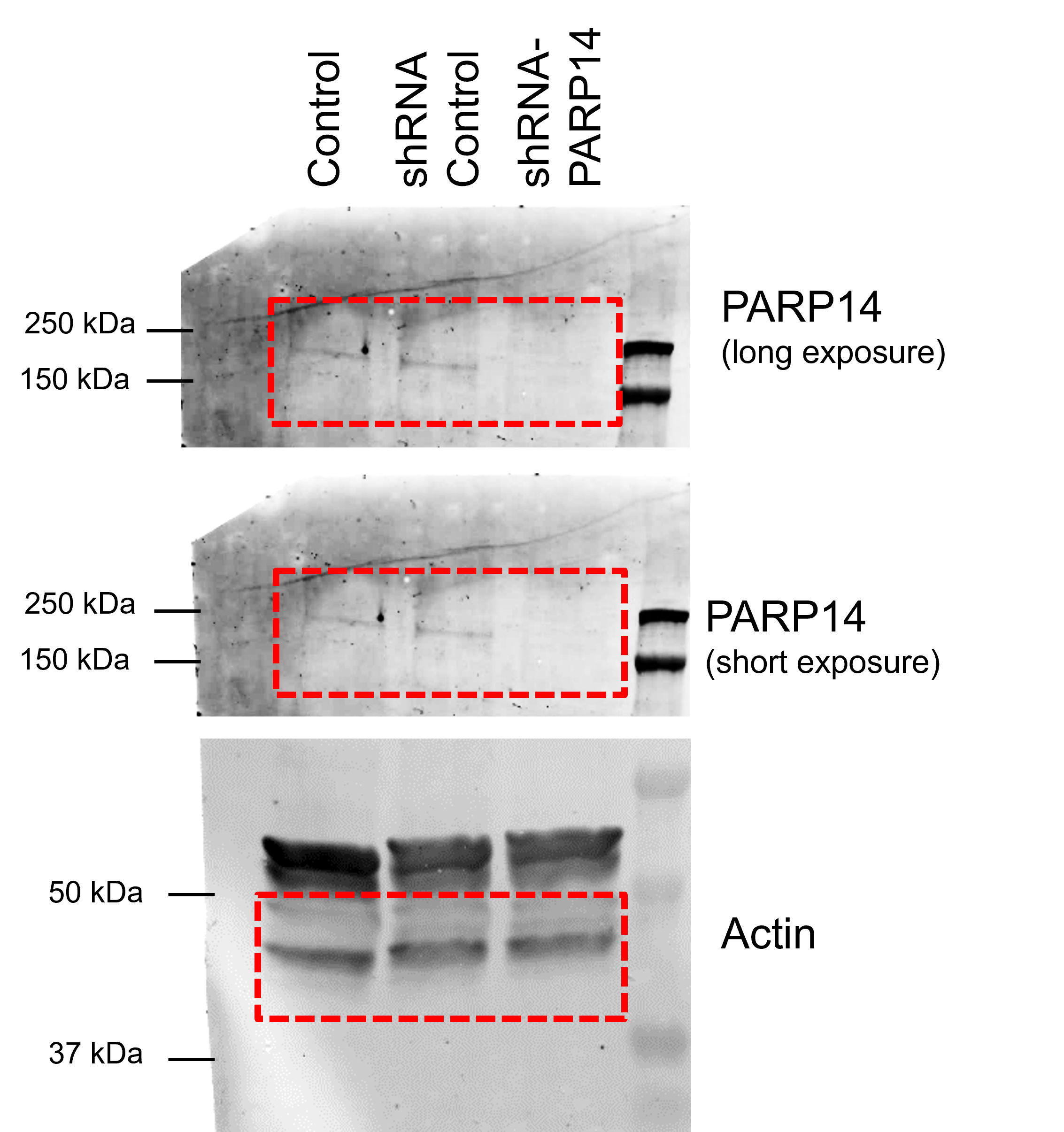

Supplement: Supplementary file 12 — Figure EV1 Source Data [file 44318_2025_421_MOESM12_ESM.zip › EV1/EV1G.tif]

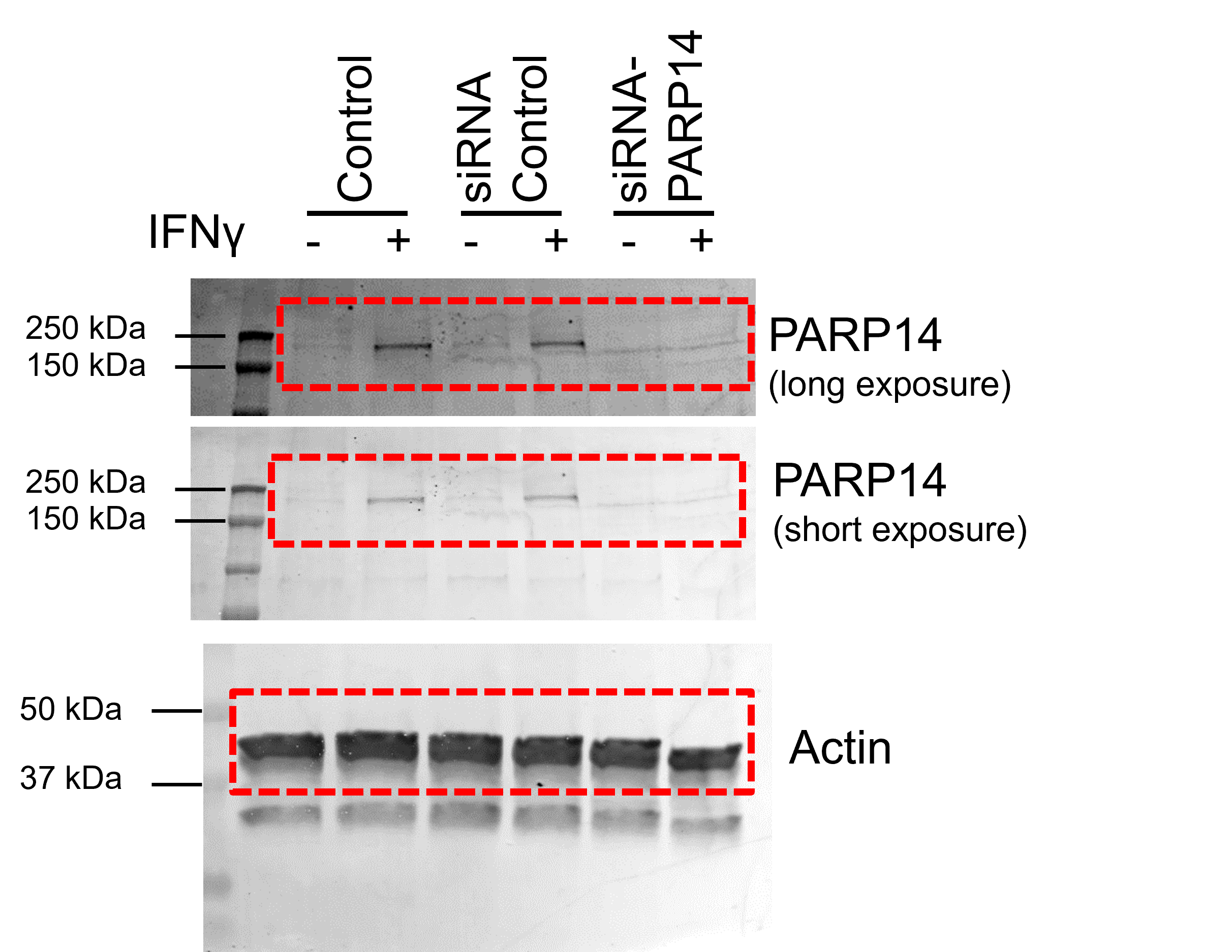

Supplement: Supplementary file 12 — Figure EV1 Source Data [file 44318_2025_421_MOESM12_ESM.zip › EV1/EV1H.tif]

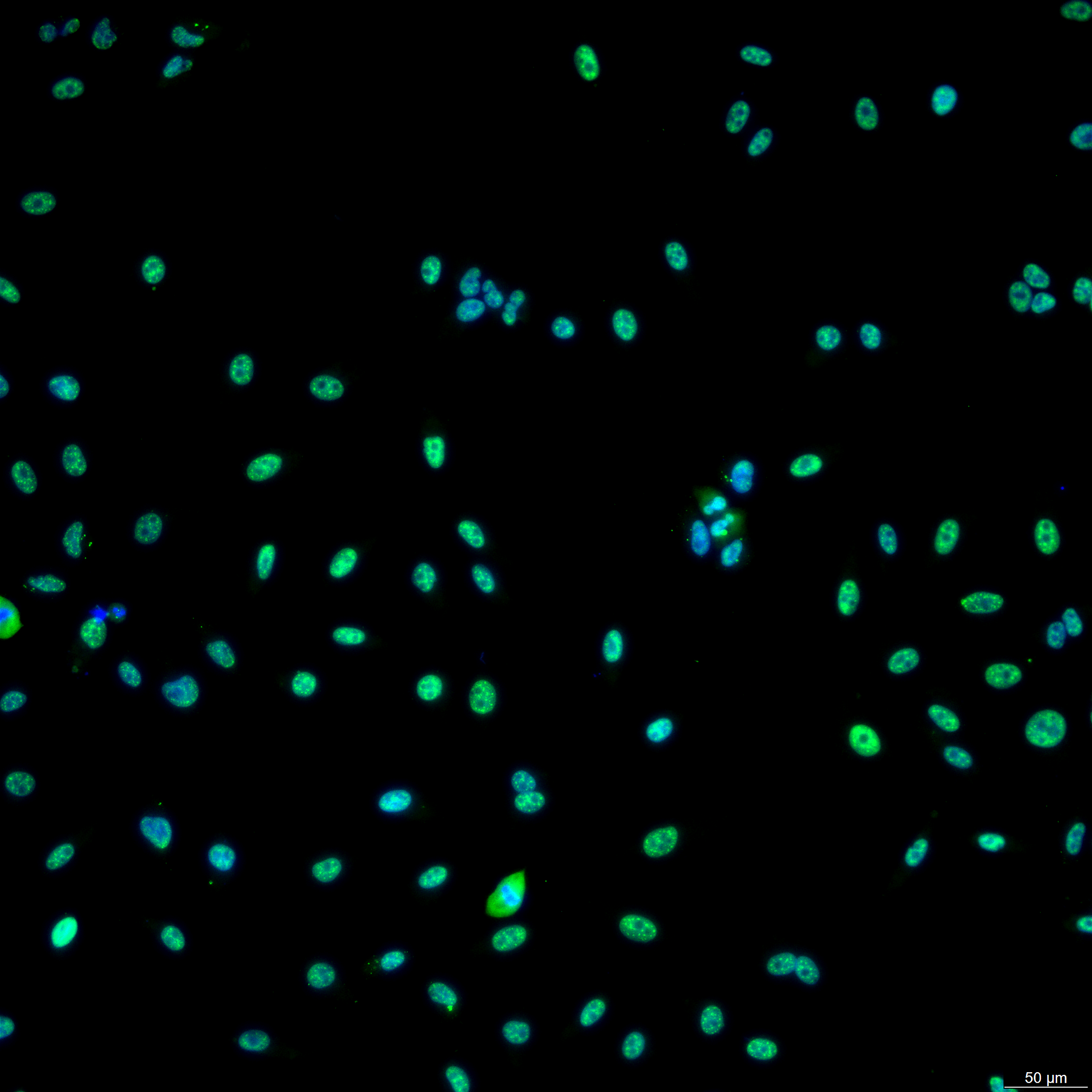

Supplement: Supplementary file 12 — Figure EV1 Source Data [file 44318_2025_421_MOESM12_ESM.zip › EV1/EV1I/Control.tif]

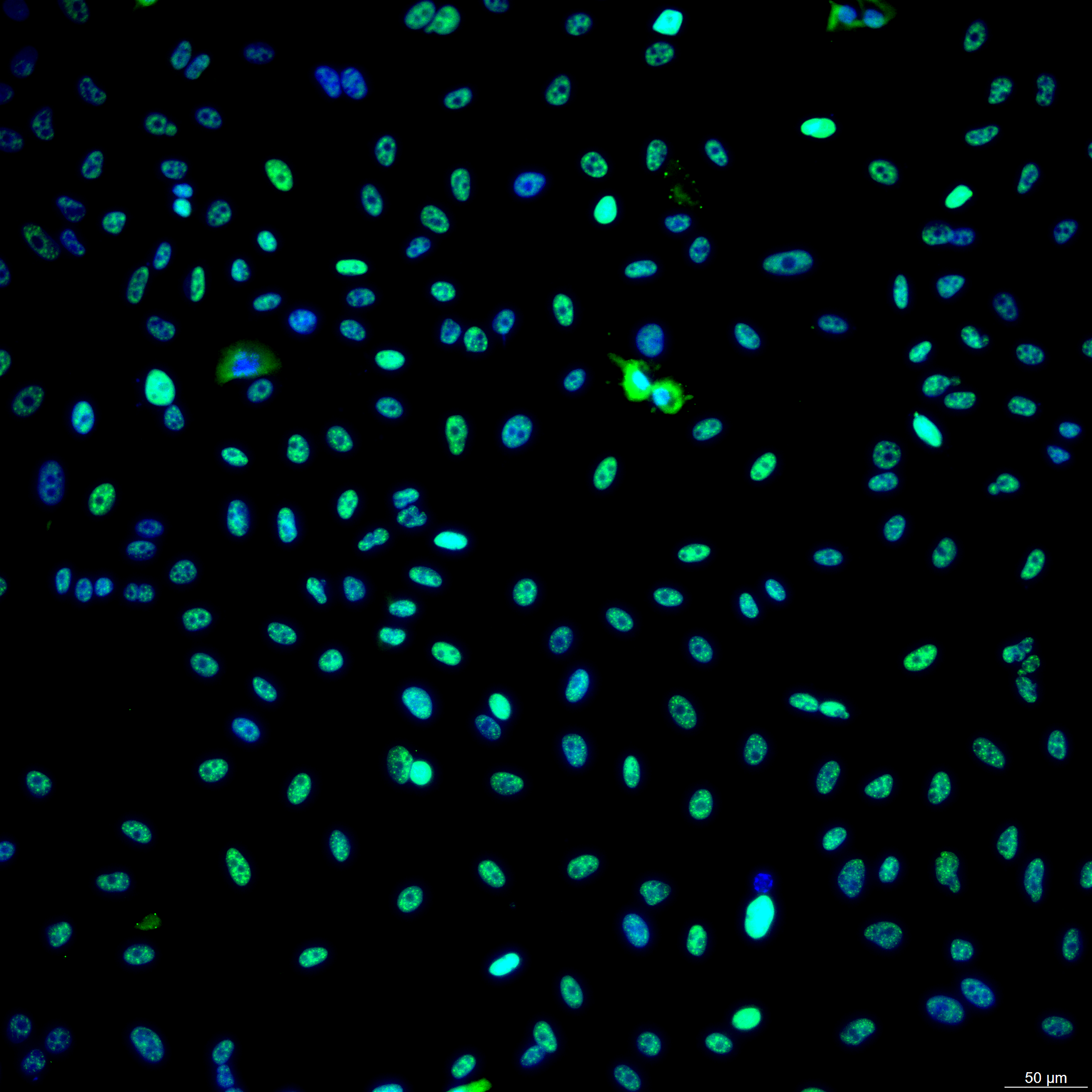

Supplement: Supplementary file 12 — Figure EV1 Source Data [file 44318_2025_421_MOESM12_ESM.zip › EV1/EV1I/si-PARP14 KD IFN γ.png]

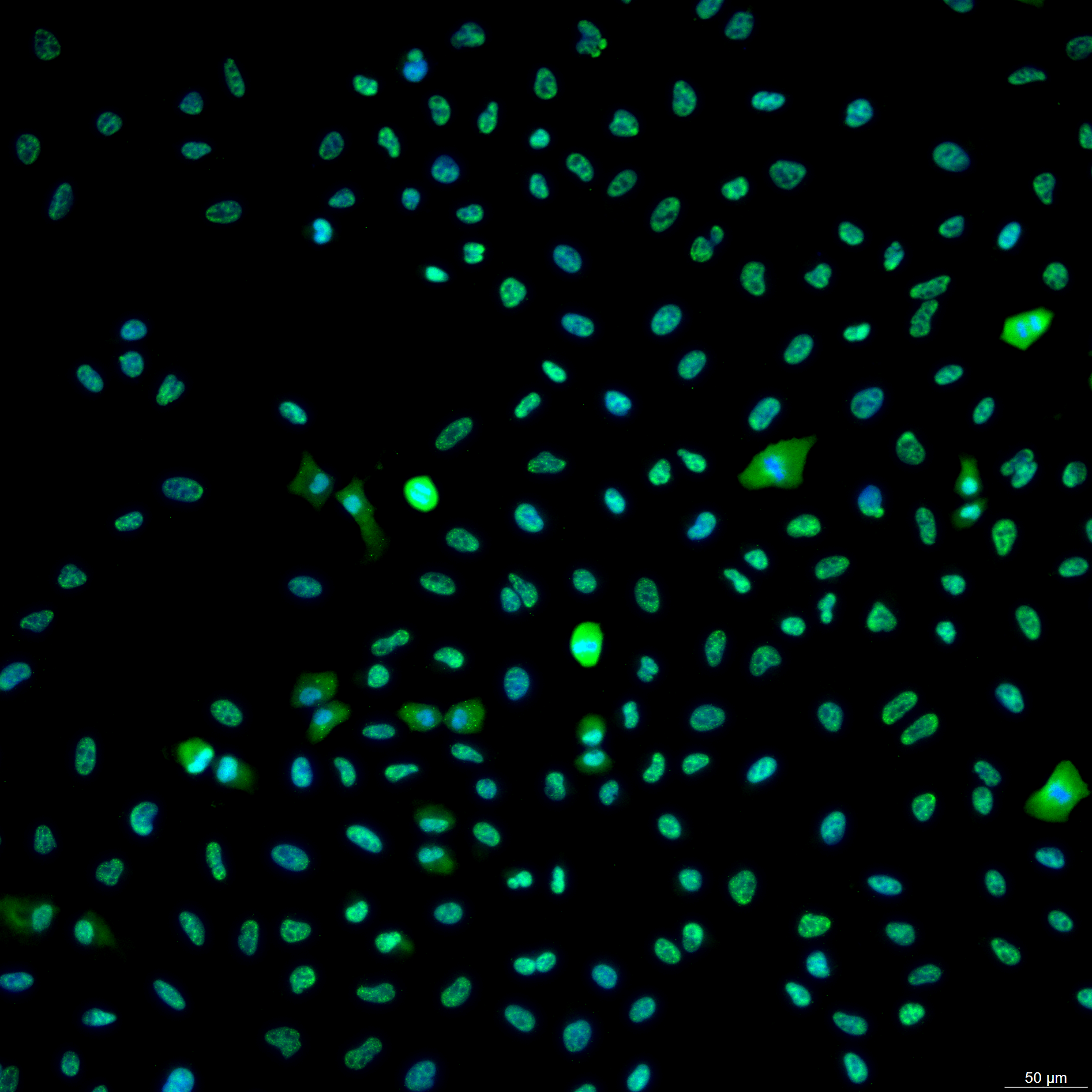

Supplement: Supplementary file 12 — Figure EV1 Source Data [file 44318_2025_421_MOESM12_ESM.zip › EV1/EV1I/si-PARP14 KD.png]

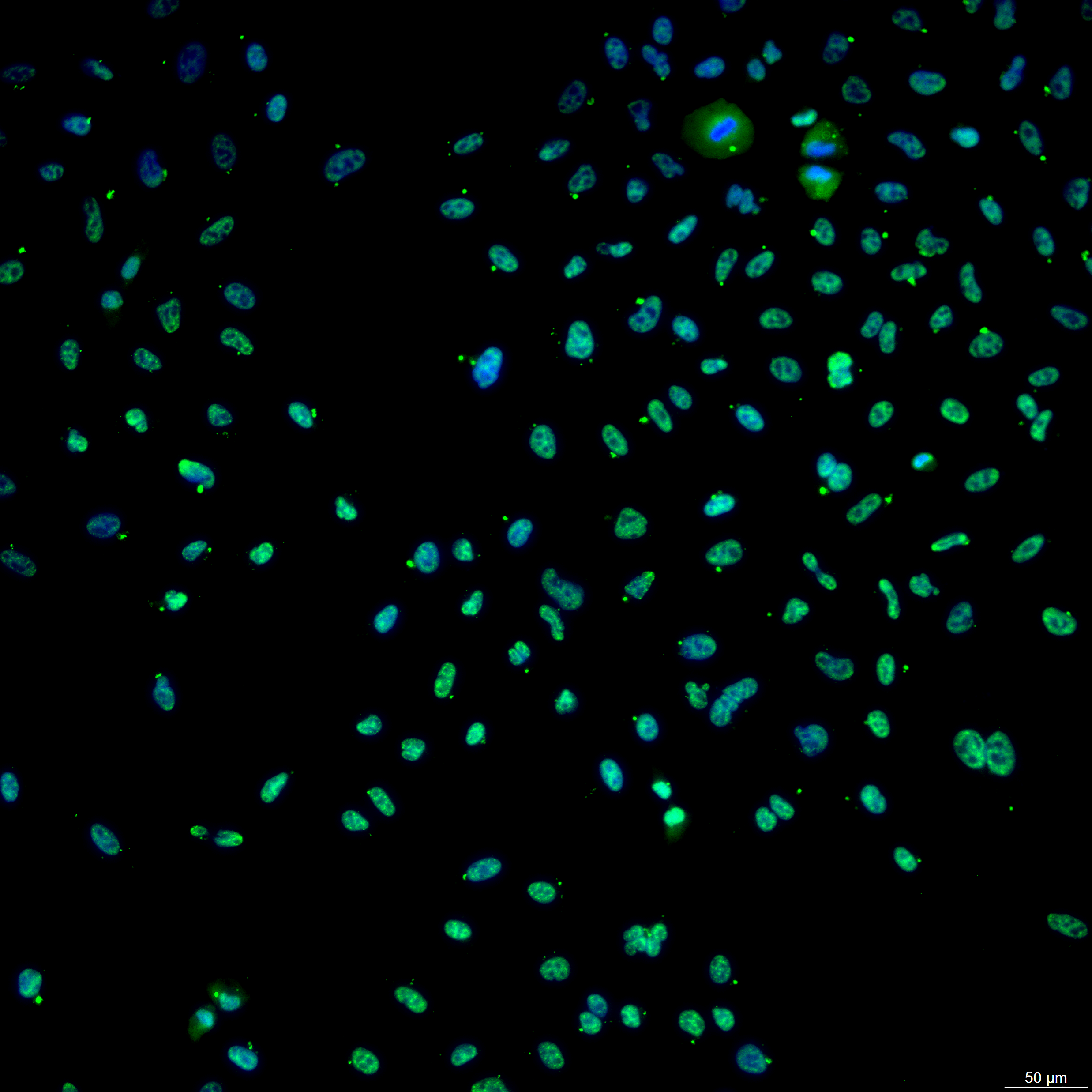

Supplement: Supplementary file 12 — Figure EV1 Source Data [file 44318_2025_421_MOESM12_ESM.zip › EV1/EV1I/WT IFN γ.png]

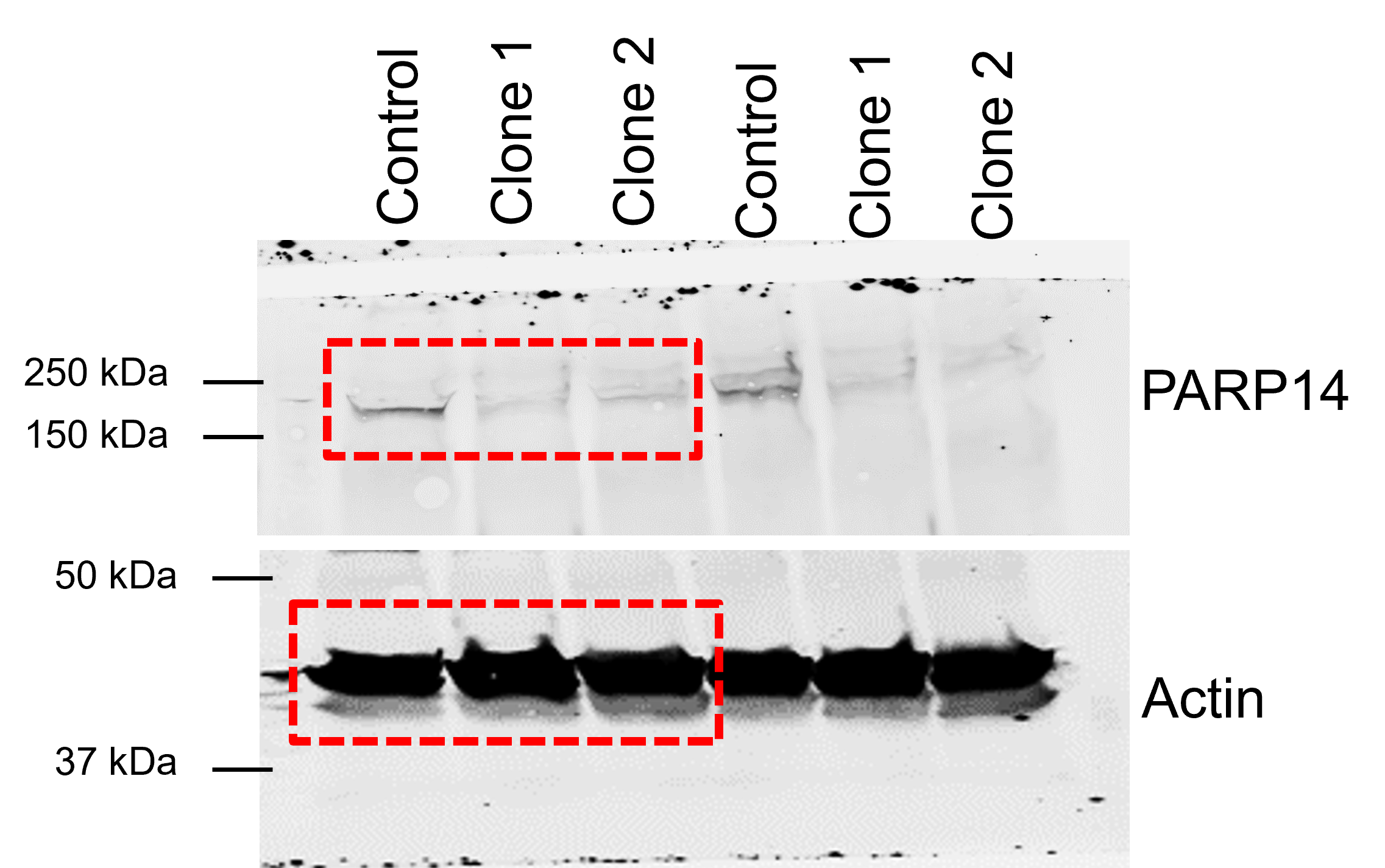

Supplement: Supplementary file 12 — Figure EV1 Source Data [file 44318_2025_421_MOESM12_ESM.zip › EV1/EV1J.tif]
