# Supplementary figures and images for: Interferon-induced PARP14-mediated ADP-ribosylation in p62 bodies requires the ubiquitin-proteasome system (part 3 of 4)
Source: EMBO J. 2025 Apr 7;44(10):2741–73. doi: 10.1038/s44318-025-00421-4 (PMC12084362; doi:10.1038/s44318-025-00421-4)

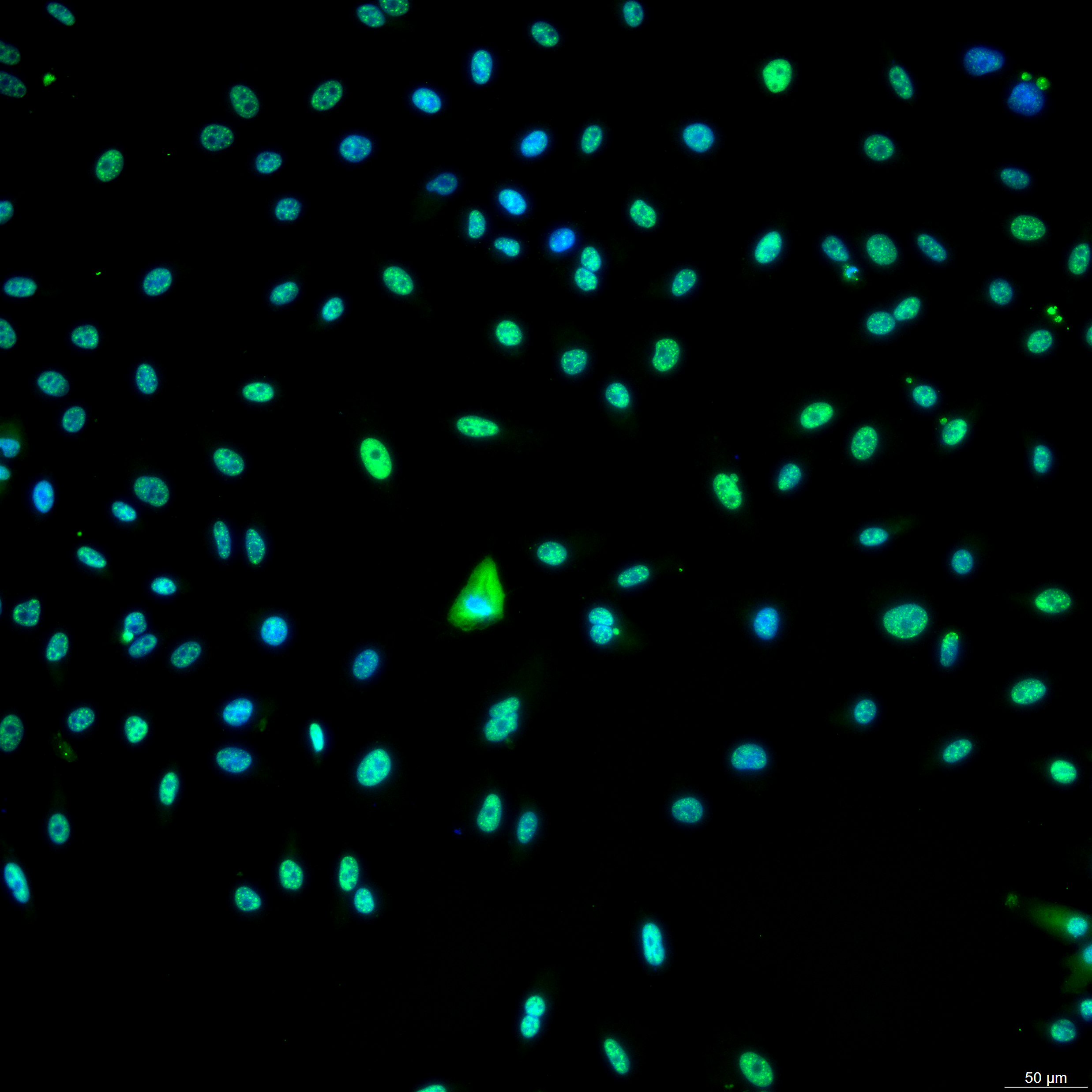

Supplement: Supplementary file 12 — Figure EV1 Source Data [file 44318_2025_421_MOESM12_ESM.zip › EV1/EV1K/Control.png]

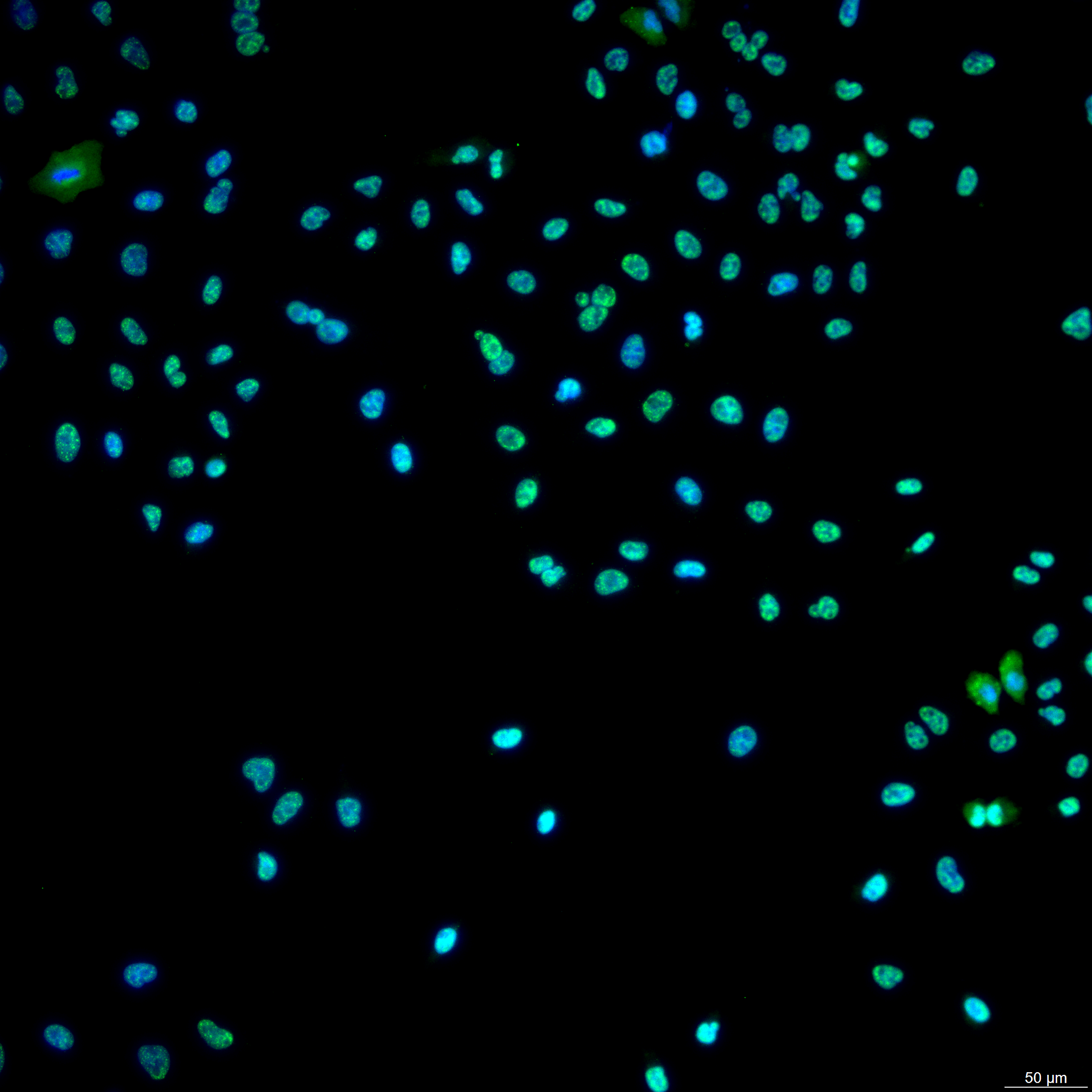

Supplement: Supplementary file 12 — Figure EV1 Source Data [file 44318_2025_421_MOESM12_ESM.zip › EV1/EV1K/sh-PARP14 KD IFN γ.png]

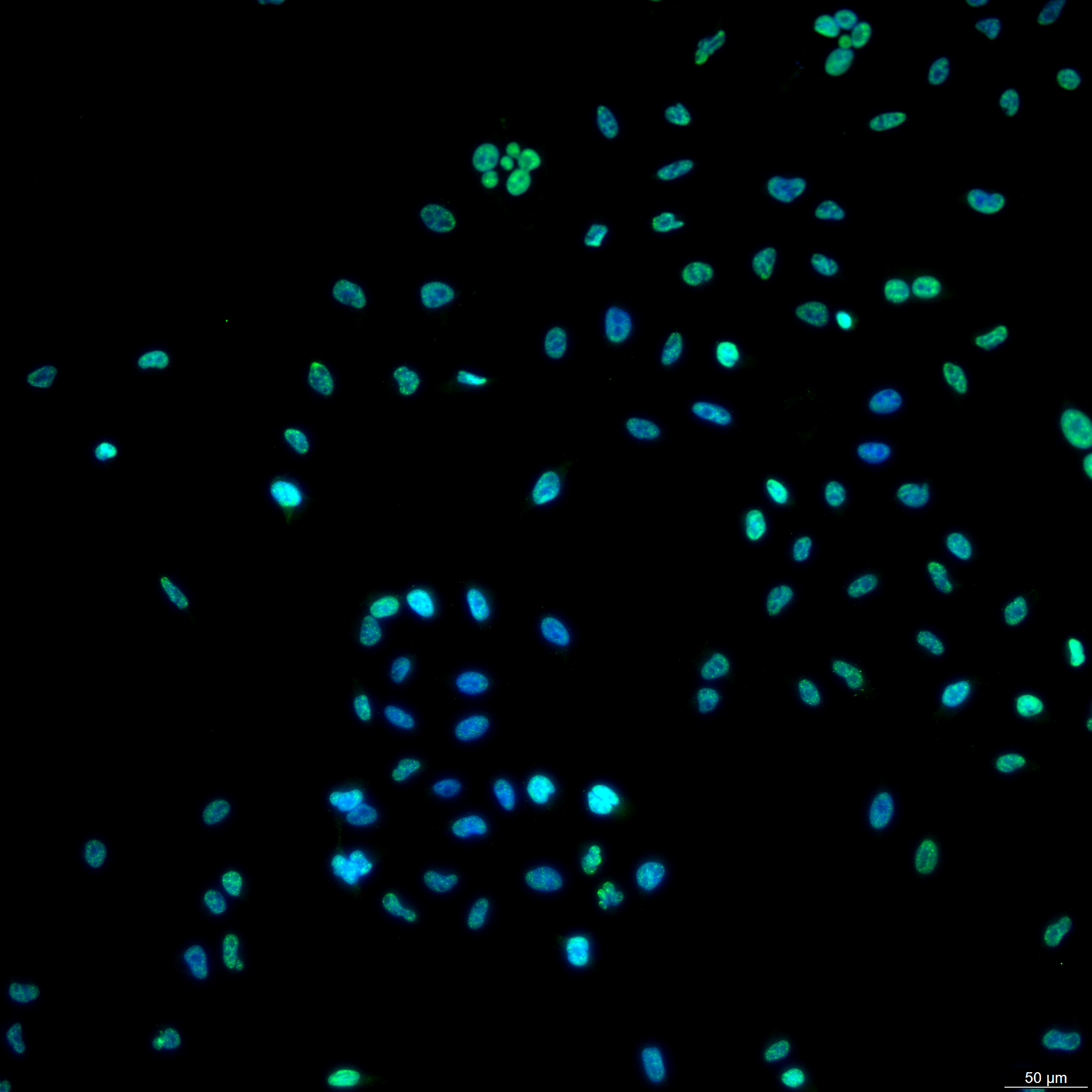

Supplement: Supplementary file 12 — Figure EV1 Source Data [file 44318_2025_421_MOESM12_ESM.zip › EV1/EV1K/sh-PARP14 KD.png]

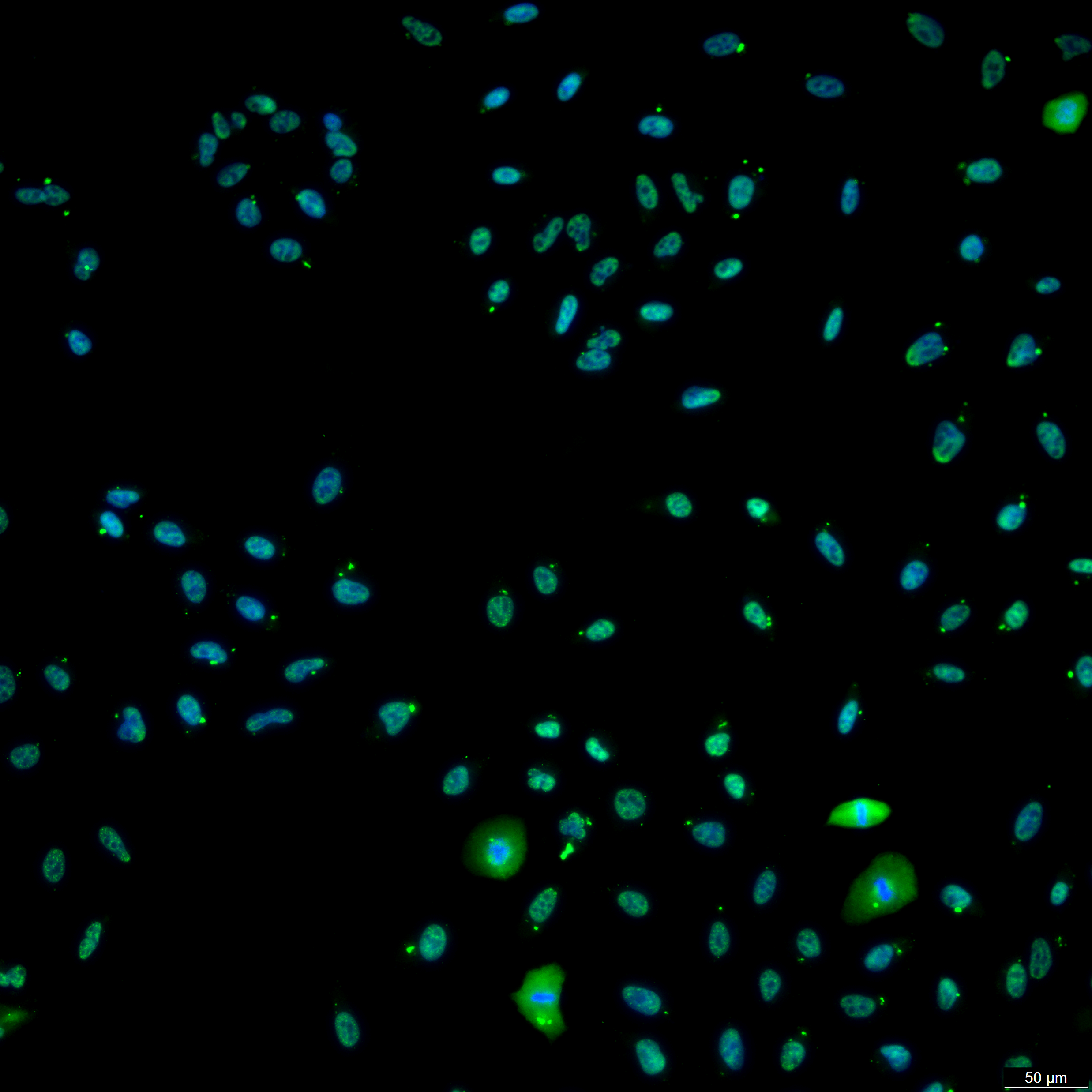

Supplement: Supplementary file 12 — Figure EV1 Source Data [file 44318_2025_421_MOESM12_ESM.zip › EV1/EV1K/WT IFN γ.png]

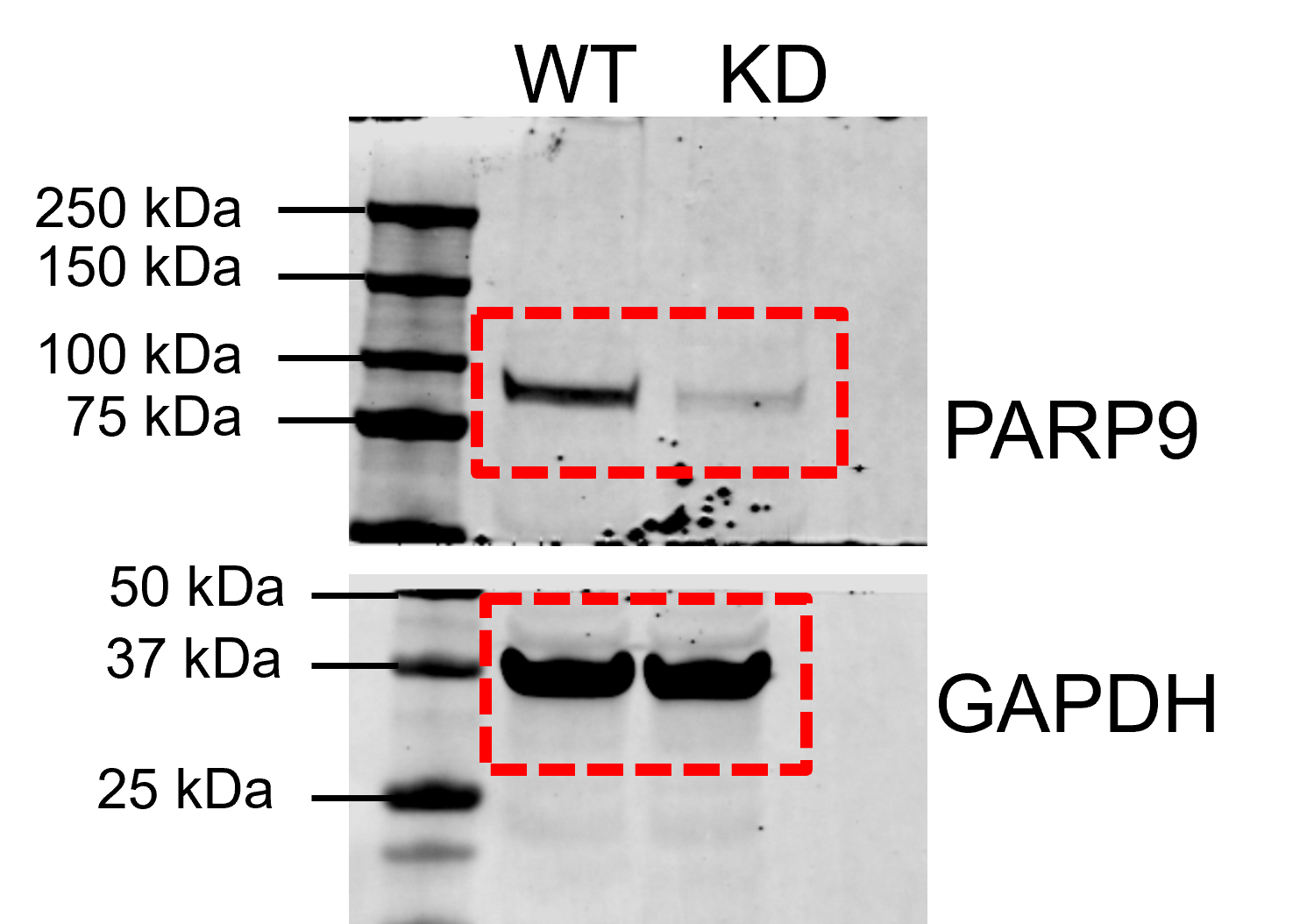

Supplement: Supplementary file 12 — Figure EV1 Source Data [file 44318_2025_421_MOESM12_ESM.zip › EV1/EV1L.tif]

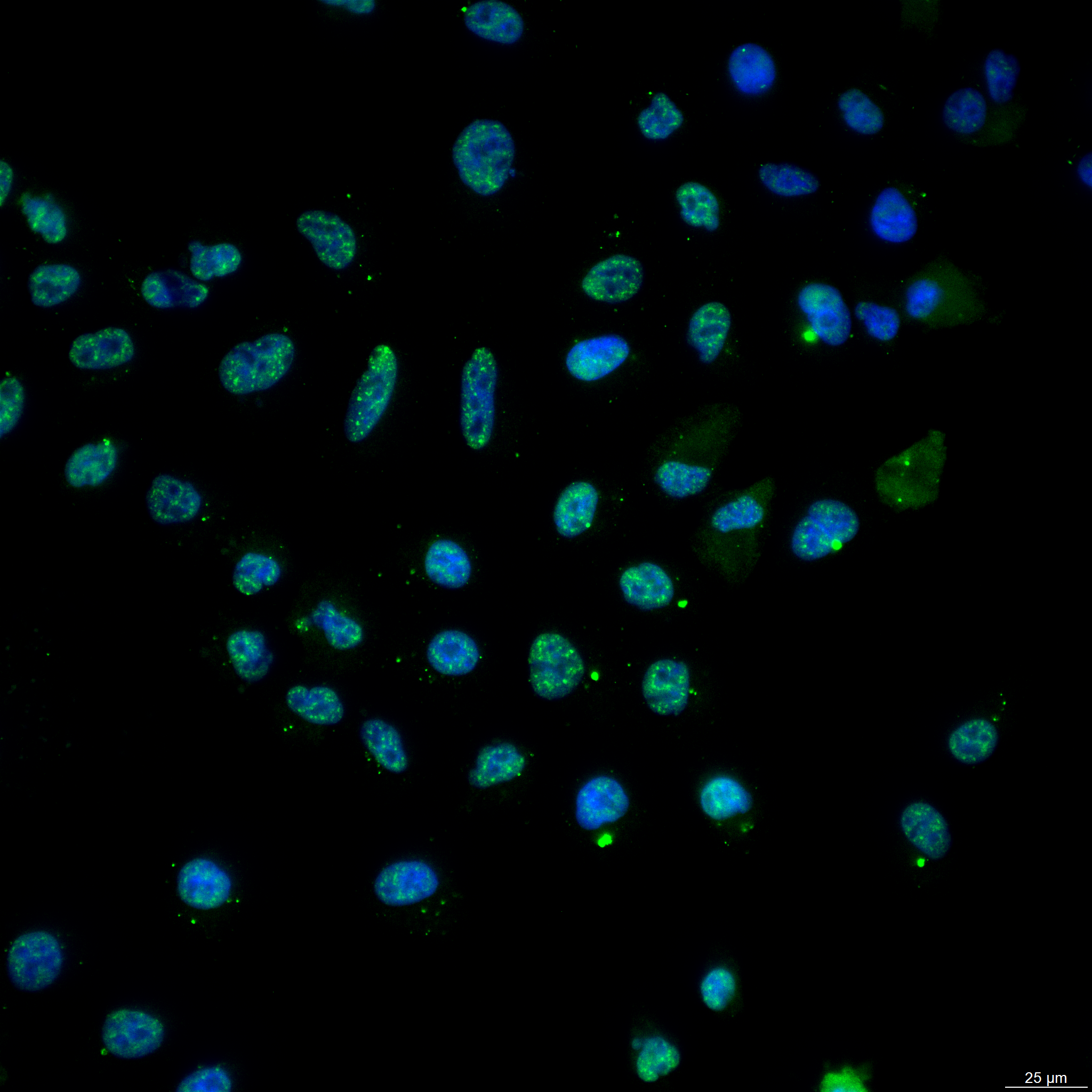

Supplement: Supplementary file 12 — Figure EV1 Source Data [file 44318_2025_421_MOESM12_ESM.zip › EV1/EV1M/PARP9 KD+lFNγ.tif]

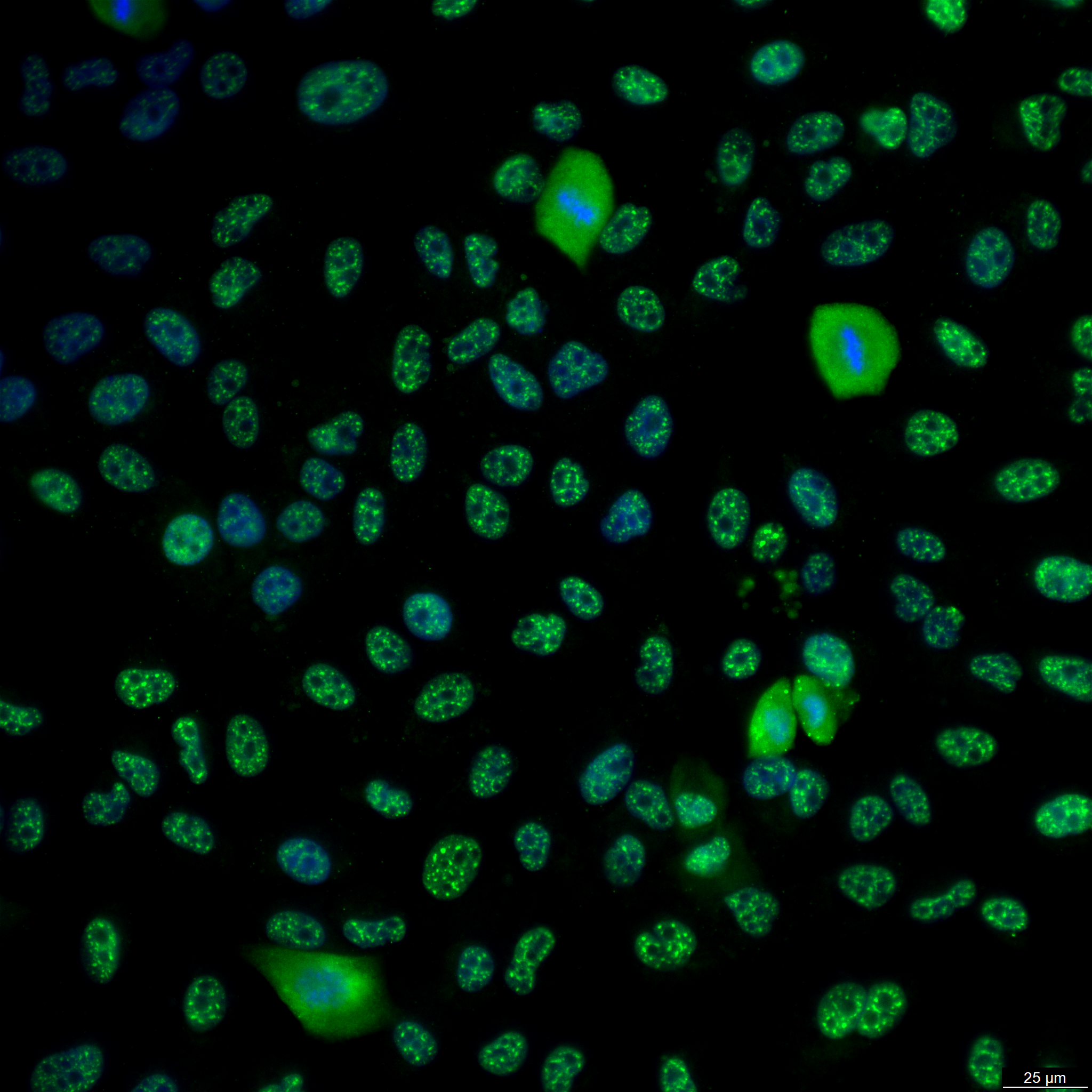

Supplement: Supplementary file 12 — Figure EV1 Source Data [file 44318_2025_421_MOESM12_ESM.zip › EV1/EV1M/PARP9 KD.tif]

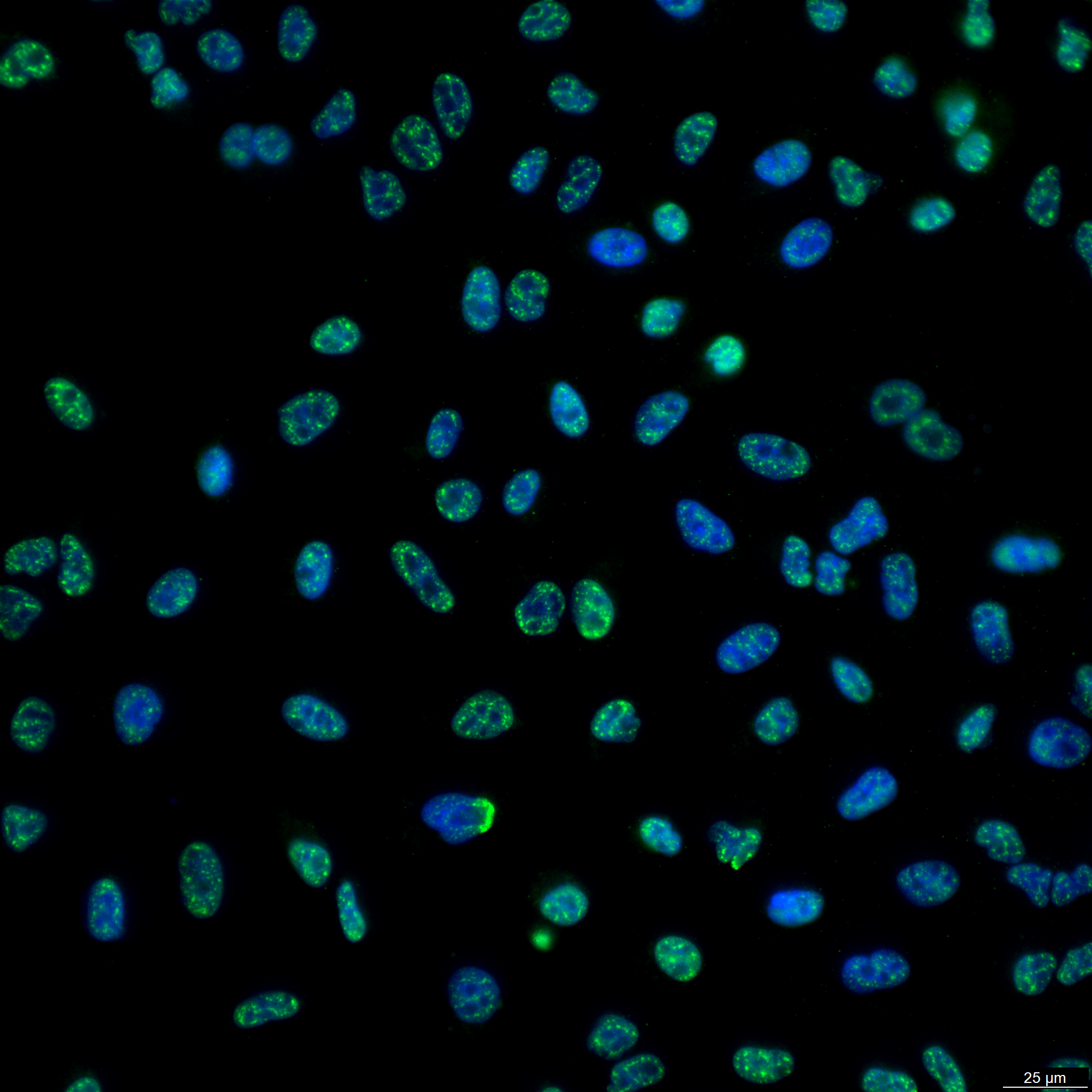

Supplement: Supplementary file 12 — Figure EV1 Source Data [file 44318_2025_421_MOESM12_ESM.zip › EV1/EV1M/WT.tif]

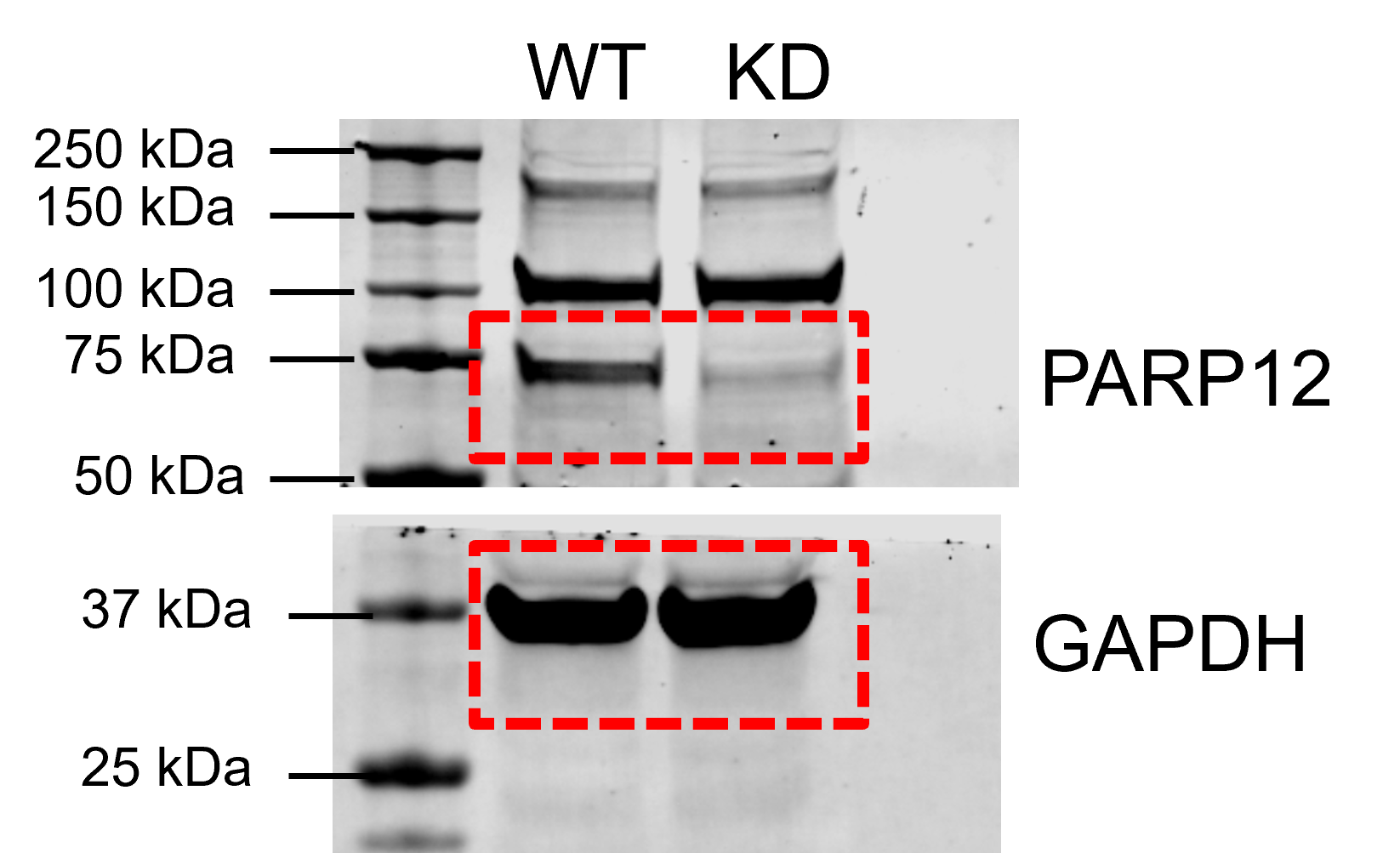

Supplement: Supplementary file 12 — Figure EV1 Source Data [file 44318_2025_421_MOESM12_ESM.zip › EV1/EV1N.tif]

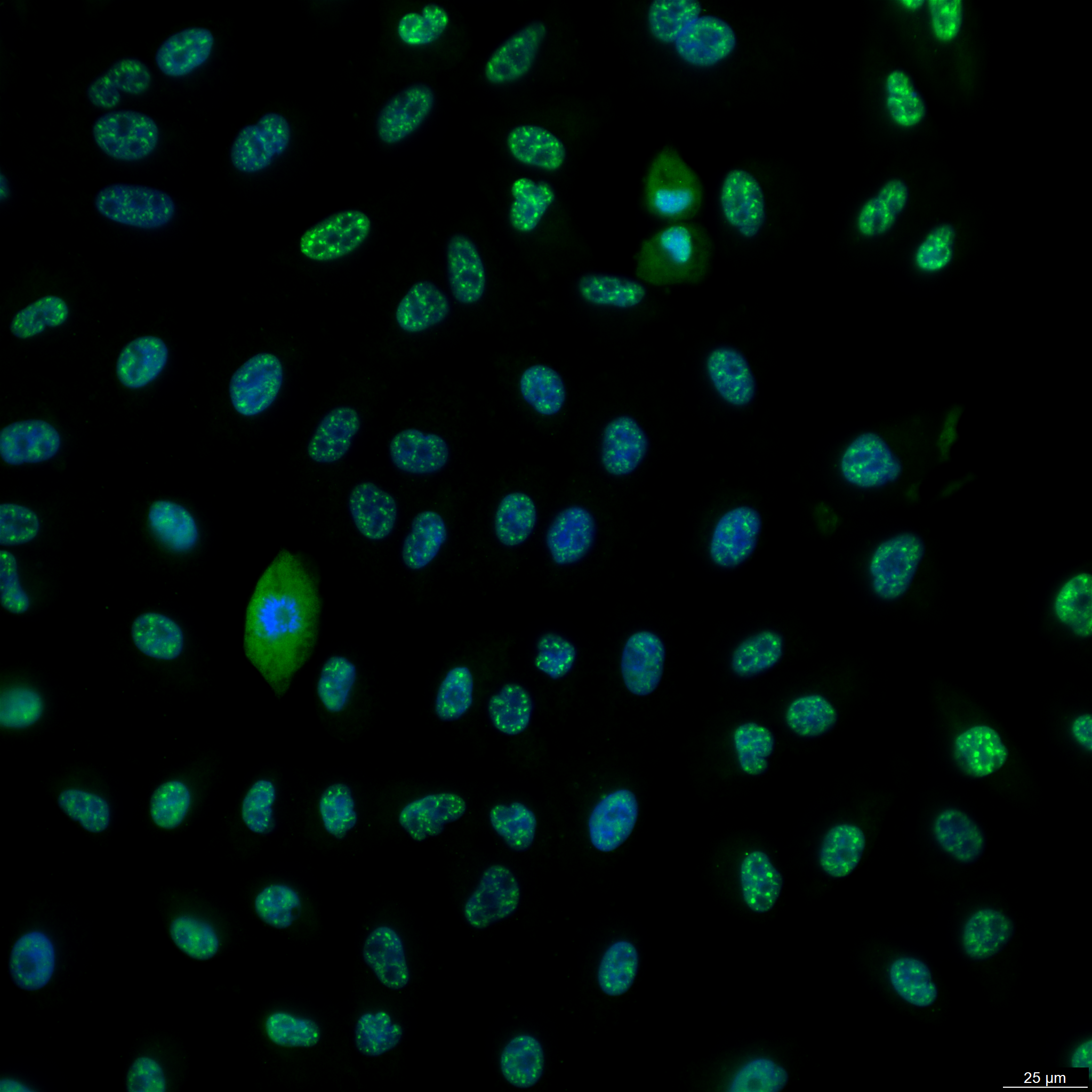

Supplement: Supplementary file 12 — Figure EV1 Source Data [file 44318_2025_421_MOESM12_ESM.zip › EV1/EV1O/PARP12 KD.tif]

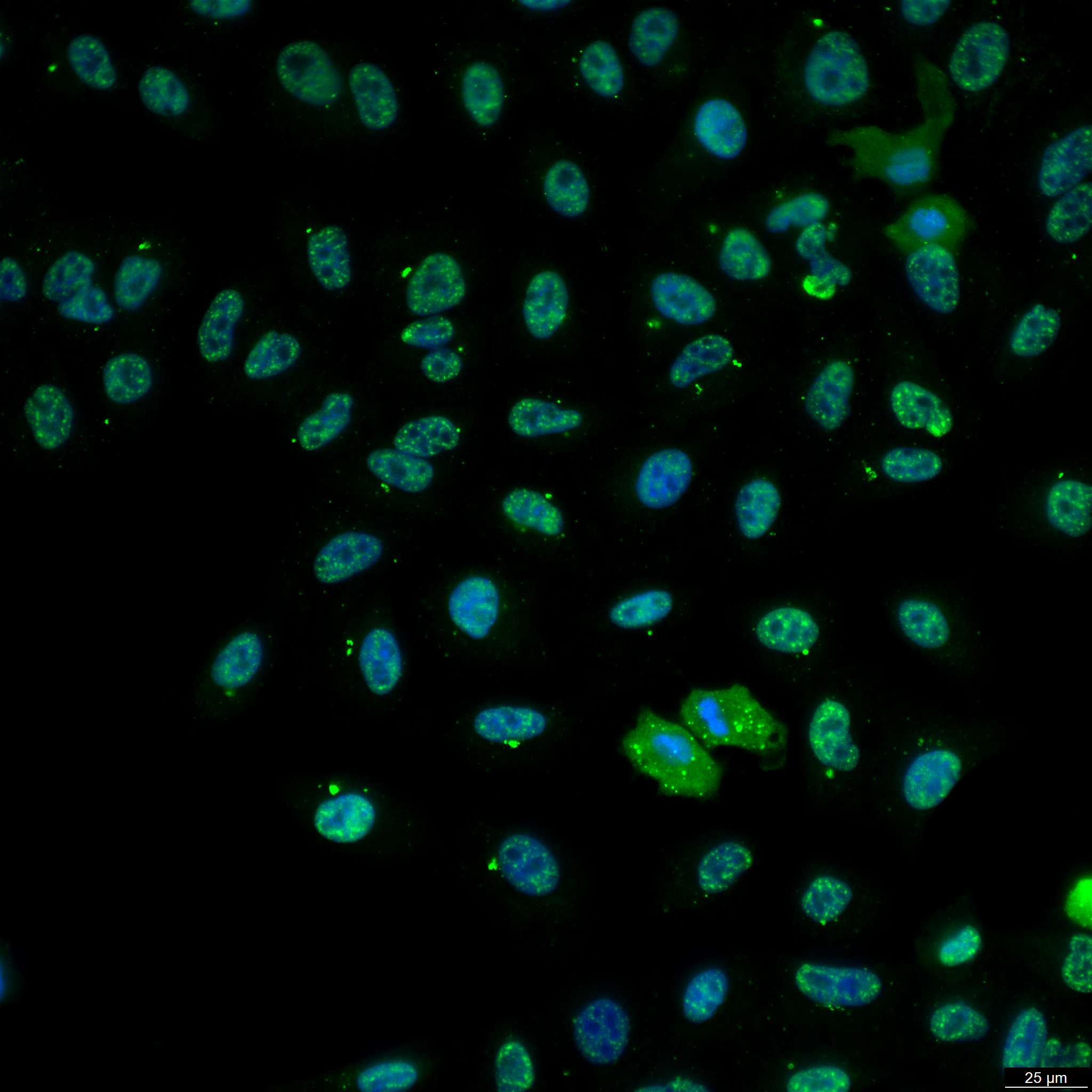

Supplement: Supplementary file 12 — Figure EV1 Source Data [file 44318_2025_421_MOESM12_ESM.zip › EV1/EV1O/PARP12 KD+lFNγ.tif]

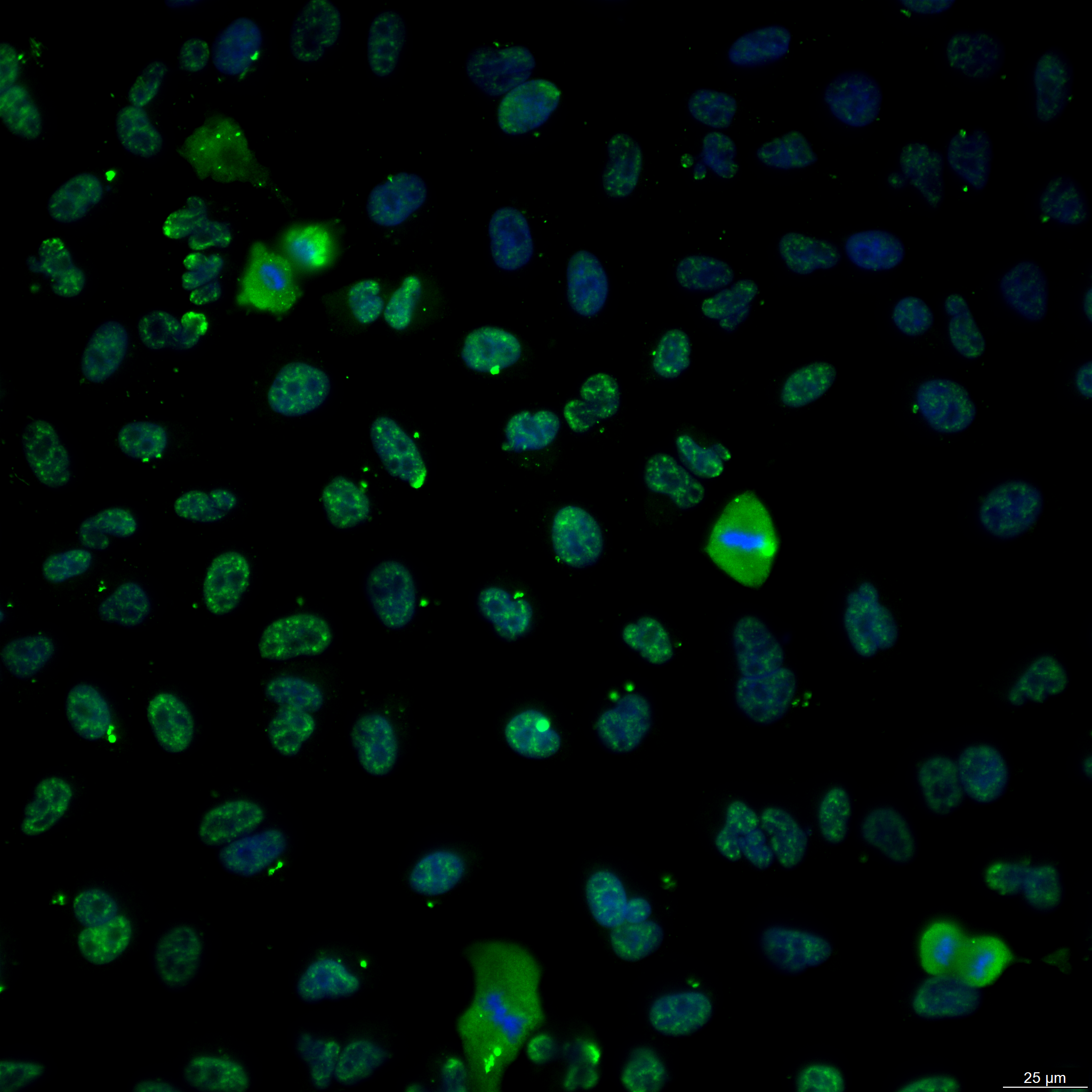

Supplement: Supplementary file 12 — Figure EV1 Source Data [file 44318_2025_421_MOESM12_ESM.zip › EV1/EV1O/WT+lFNγ.tif]

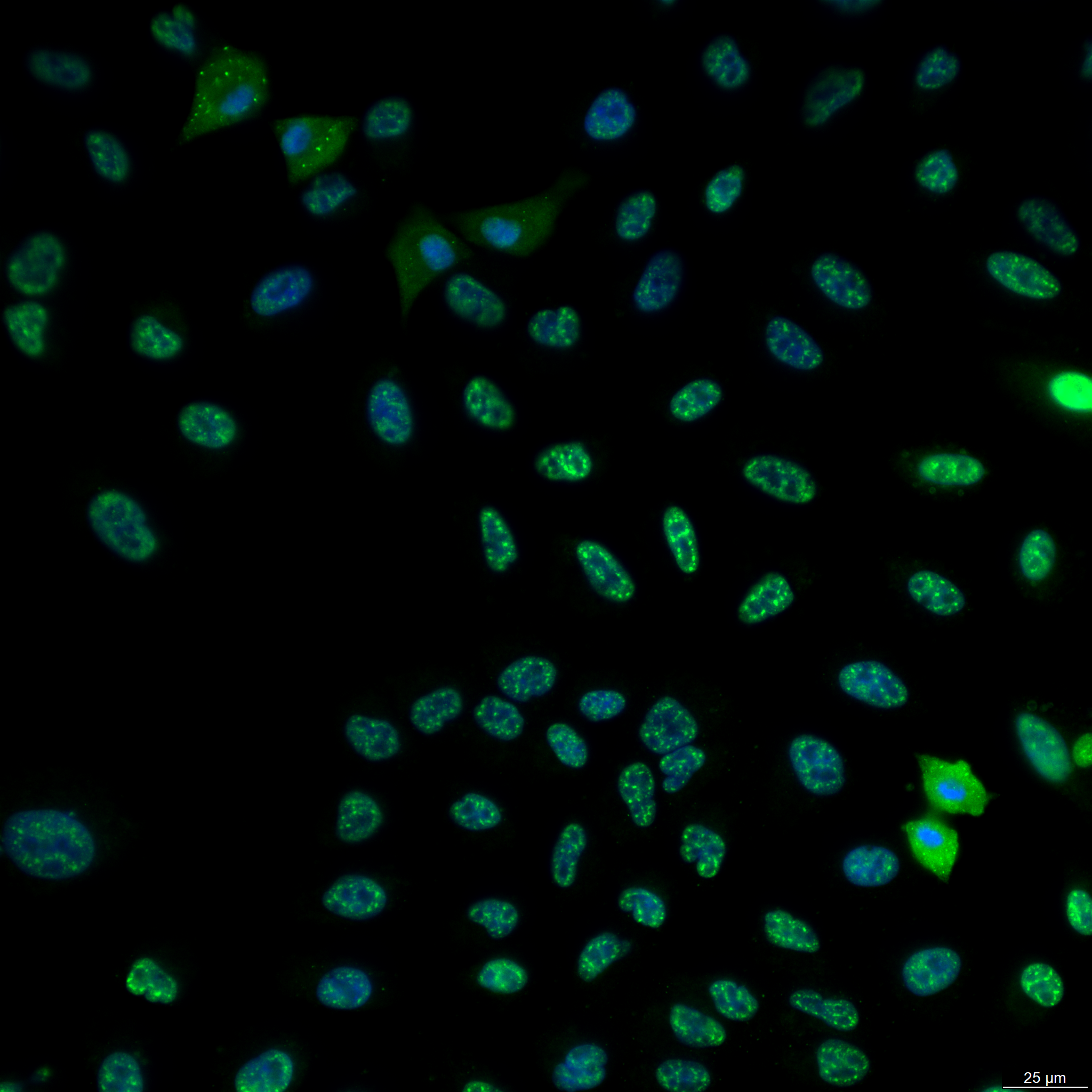

Supplement: Supplementary file 12 — Figure EV1 Source Data [file 44318_2025_421_MOESM12_ESM.zip › EV1/EV1O/WT.tif]

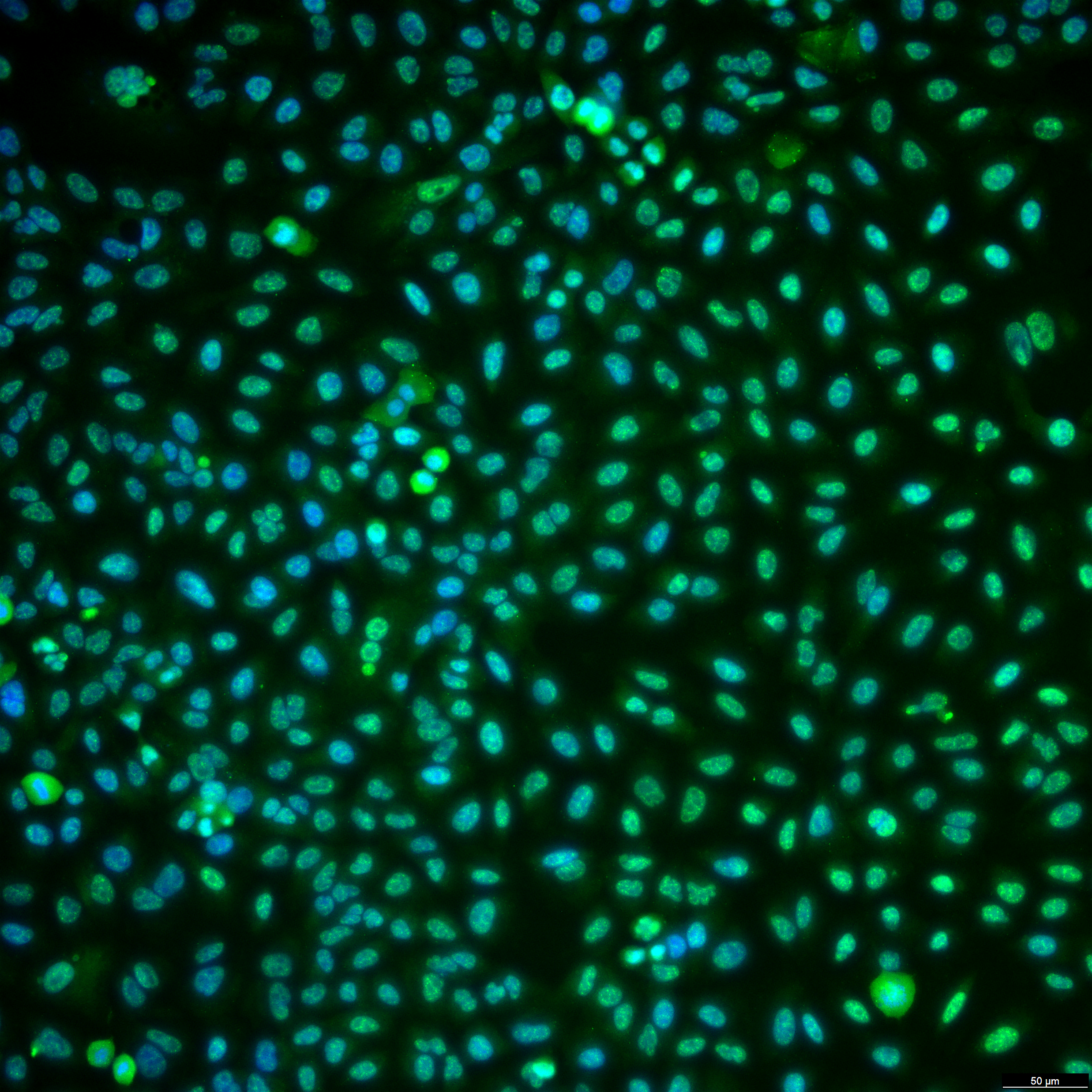

Supplement: Supplementary file 13 — Figure EV2 Source Data [file 44318_2025_421_MOESM13_ESM.zip › EV2/EV2G/Control.tif]

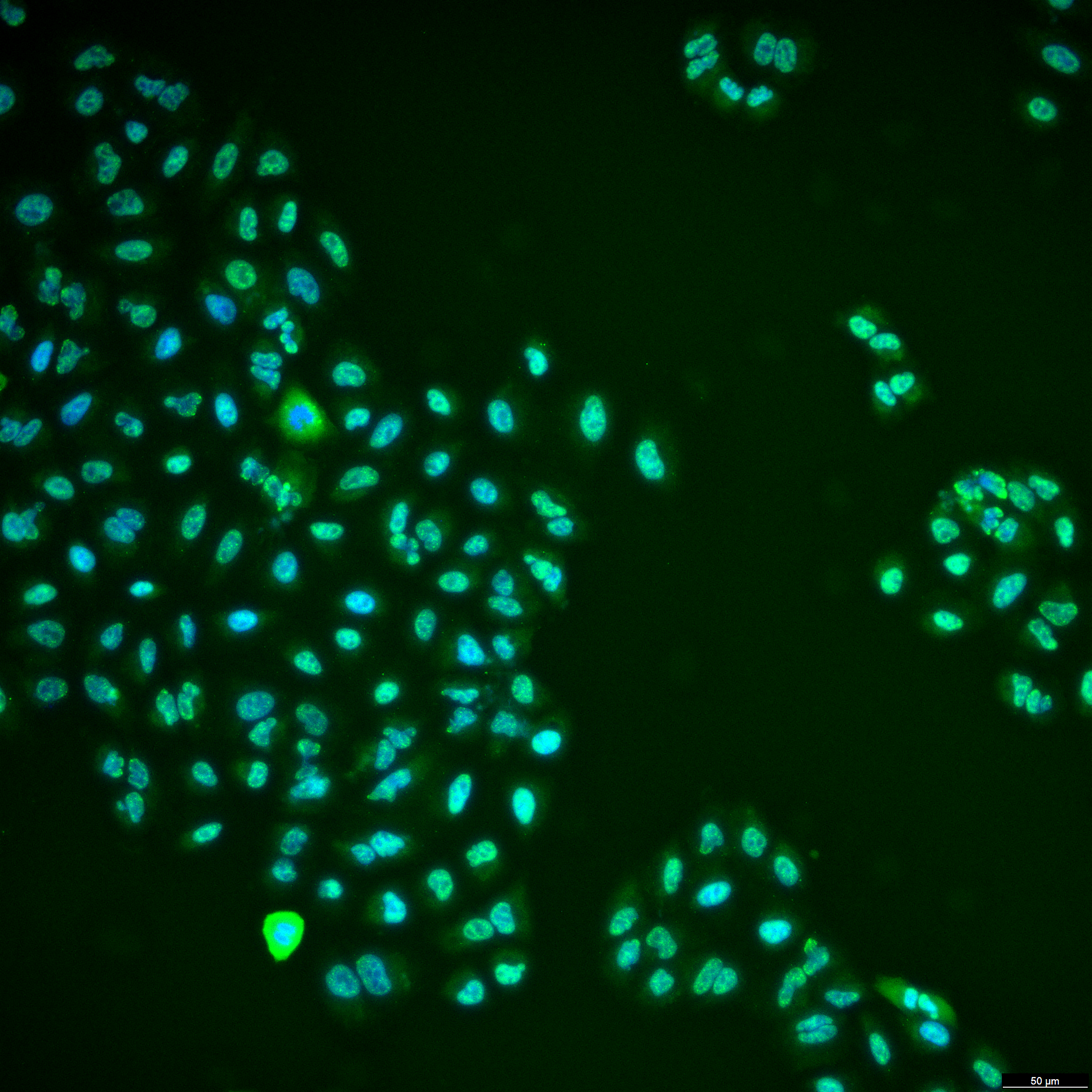

Supplement: Supplementary file 13 — Figure EV2 Source Data [file 44318_2025_421_MOESM13_ESM.zip › EV2/EV2G/IFN γ+ ITK7 10 μM .tif]

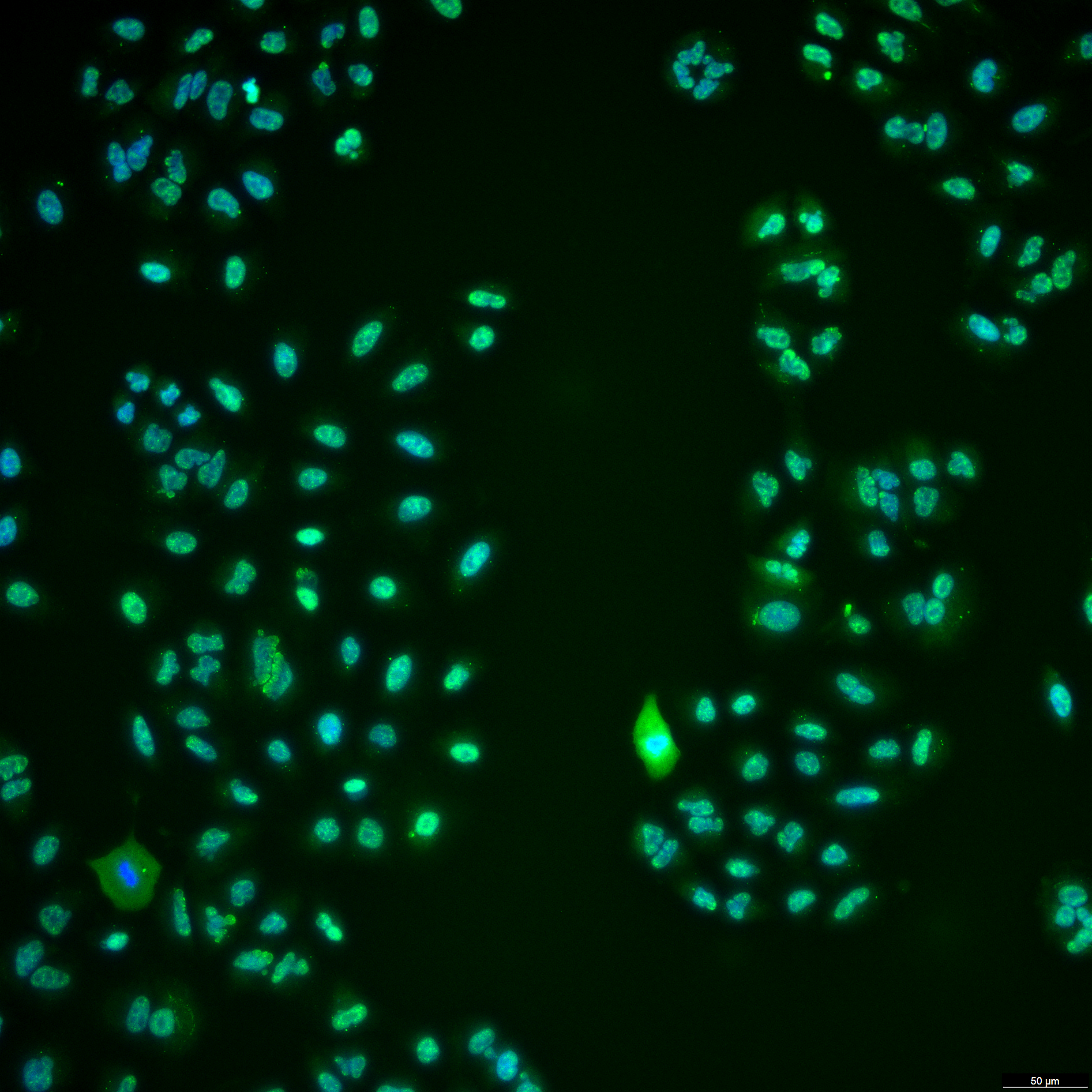

Supplement: Supplementary file 13 — Figure EV2 Source Data [file 44318_2025_421_MOESM13_ESM.zip › EV2/EV2G/IFN γ+ ITK7 100nM .tif]

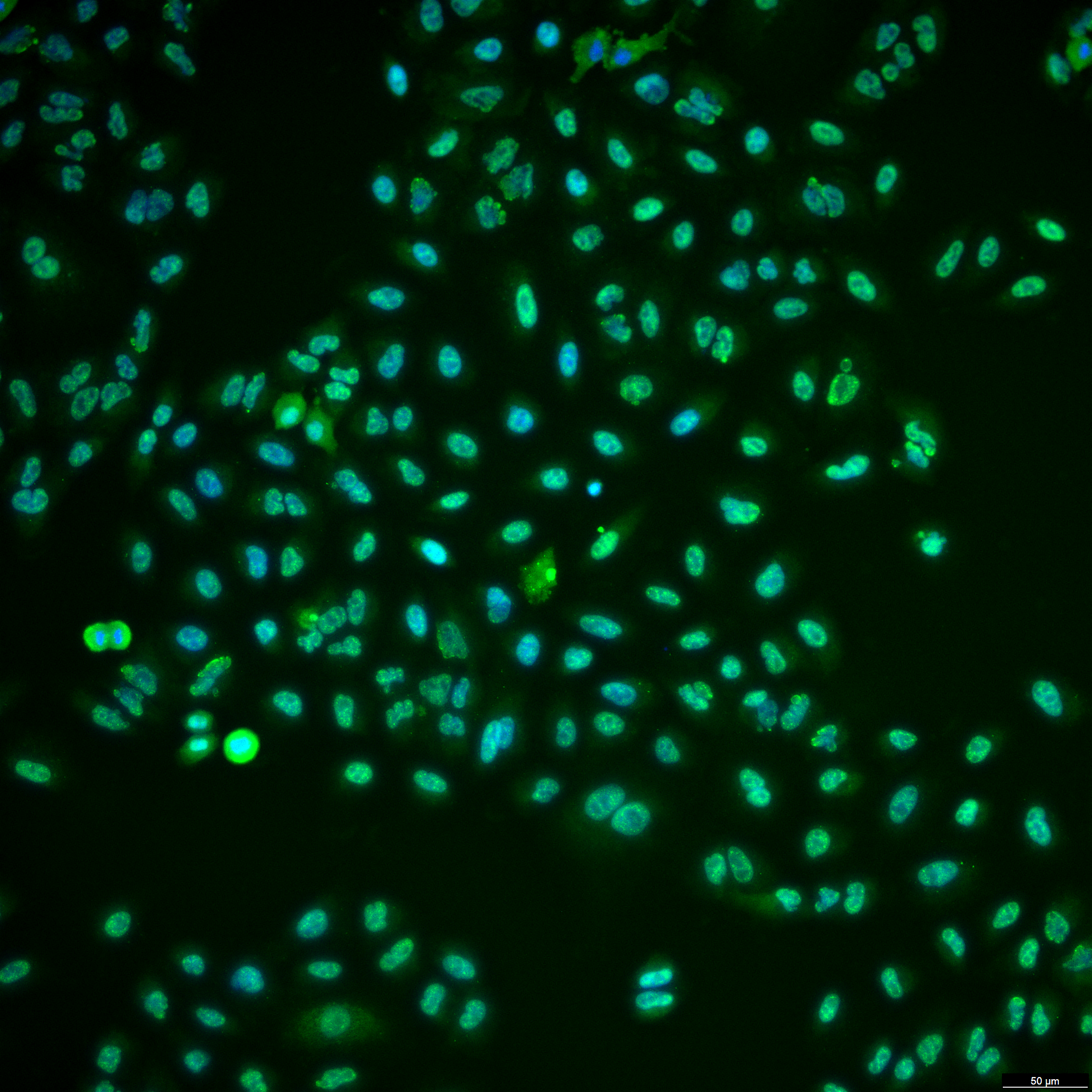

Supplement: Supplementary file 13 — Figure EV2 Source Data [file 44318_2025_421_MOESM13_ESM.zip › EV2/EV2G/IFN γ+ ITK7 1μM .tif]

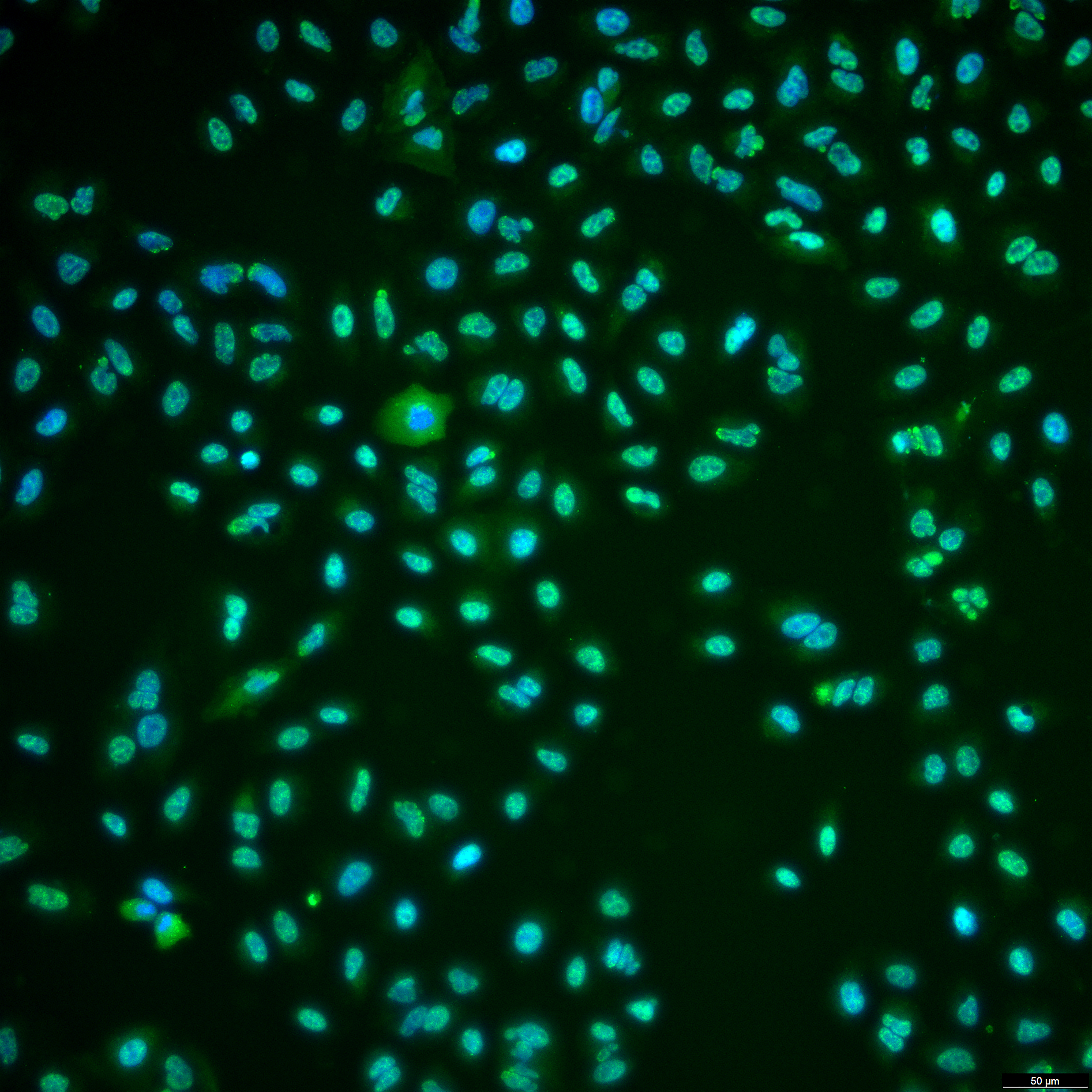

Supplement: Supplementary file 13 — Figure EV2 Source Data [file 44318_2025_421_MOESM13_ESM.zip › EV2/EV2G/IFN γ+ ITK7 5μM .tif]

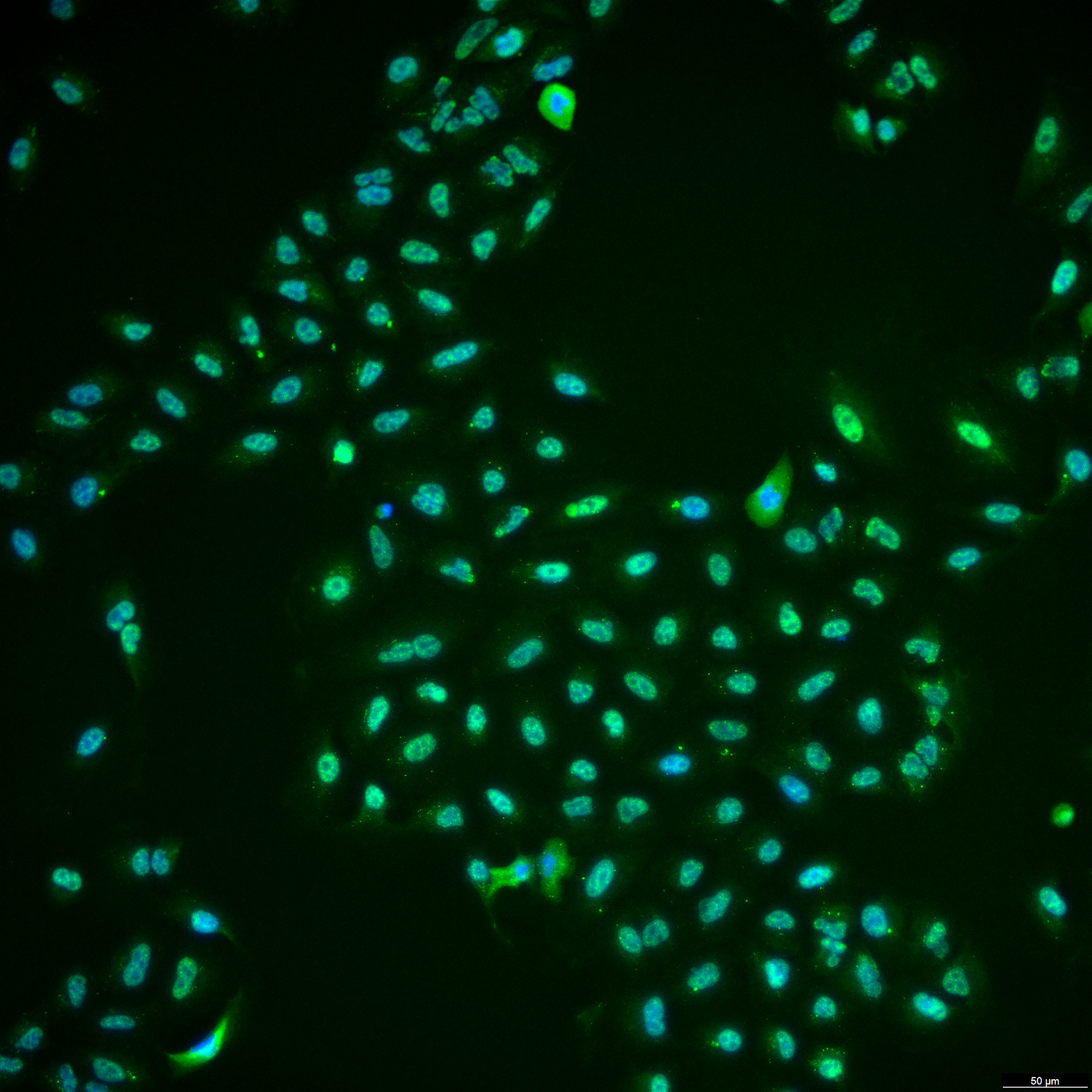

Supplement: Supplementary file 13 — Figure EV2 Source Data [file 44318_2025_421_MOESM13_ESM.zip › EV2/EV2G/IFN γ.tif]

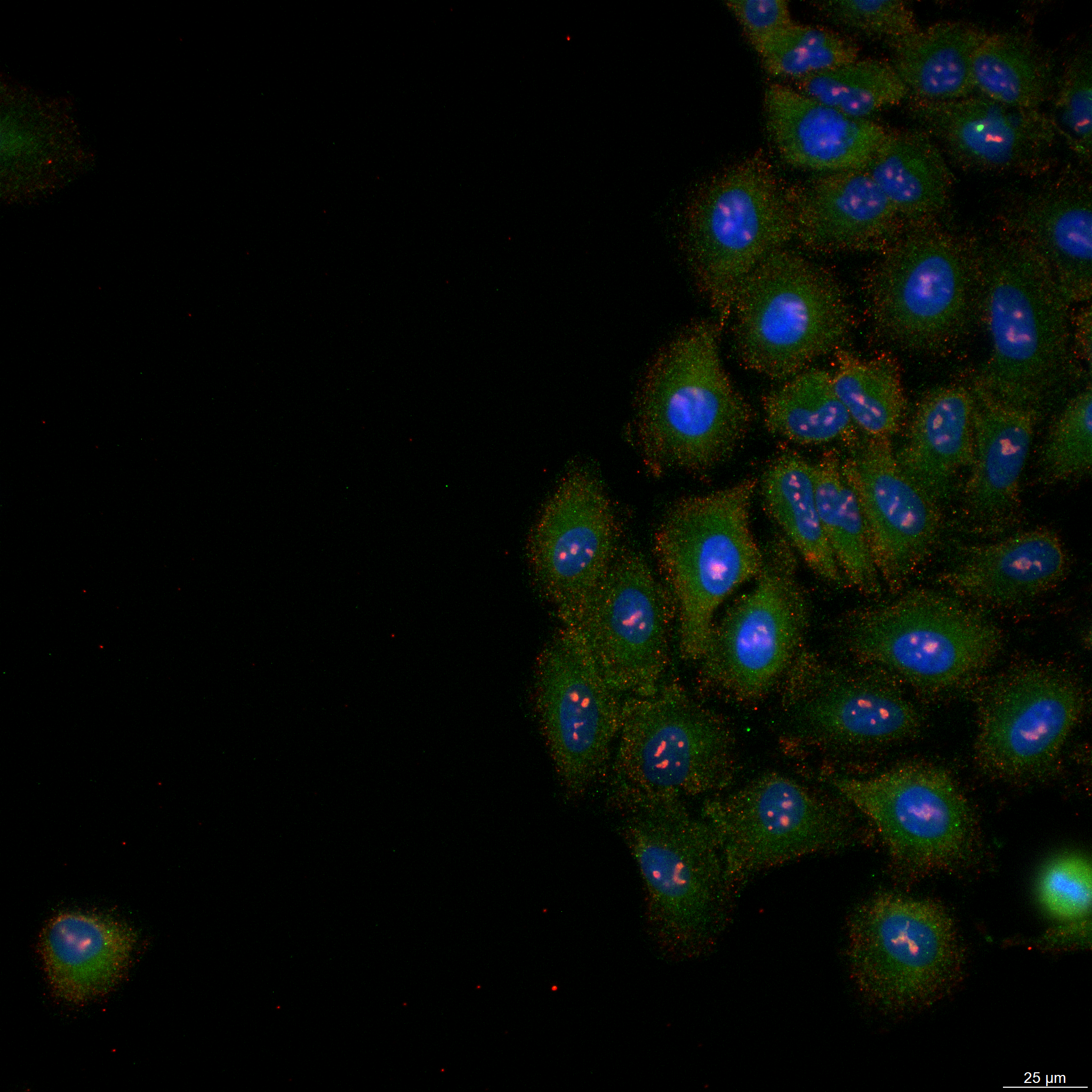

Supplement: Supplementary file 13 — Figure EV2 Source Data [file 44318_2025_421_MOESM13_ESM.zip › EV2/EV2H/Control .tif]

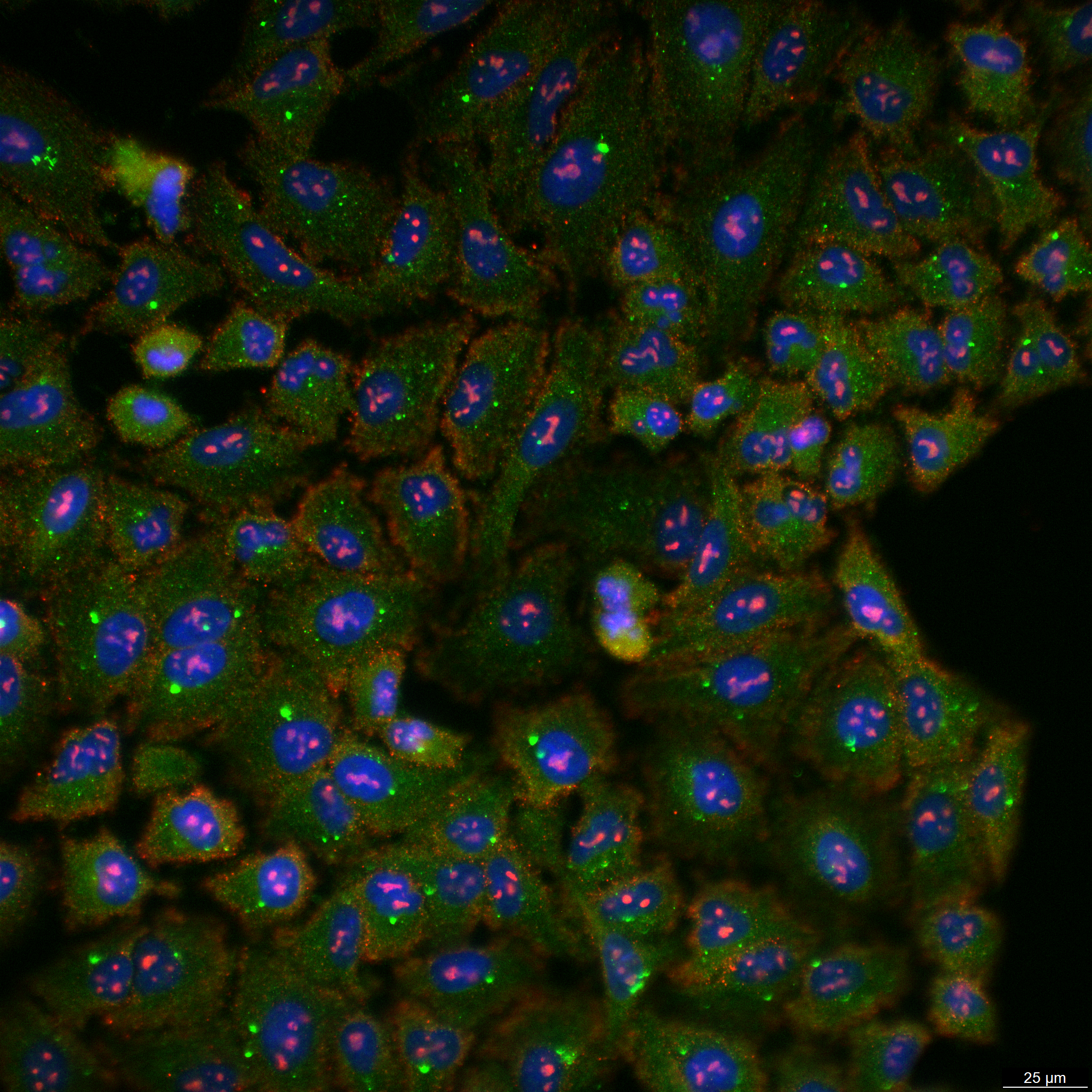

Supplement: Supplementary file 13 — Figure EV2 Source Data [file 44318_2025_421_MOESM13_ESM.zip › EV2/EV2H/IFN γ.tif]

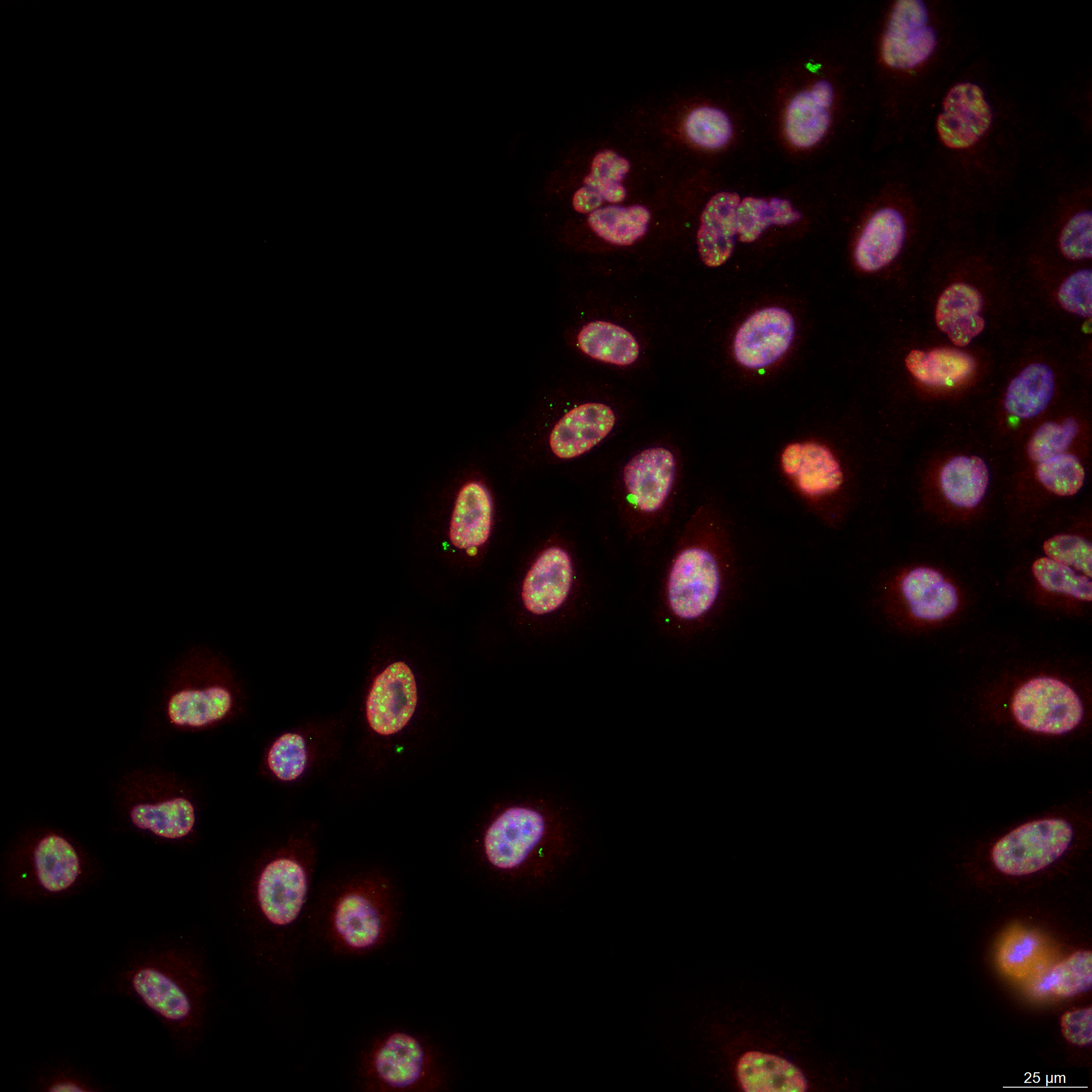

Supplement: Supplementary file 14 — Figure EV3 Source Data [file 44318_2025_421_MOESM14_ESM.zip › EV3/EV3A/20S β2.tif]

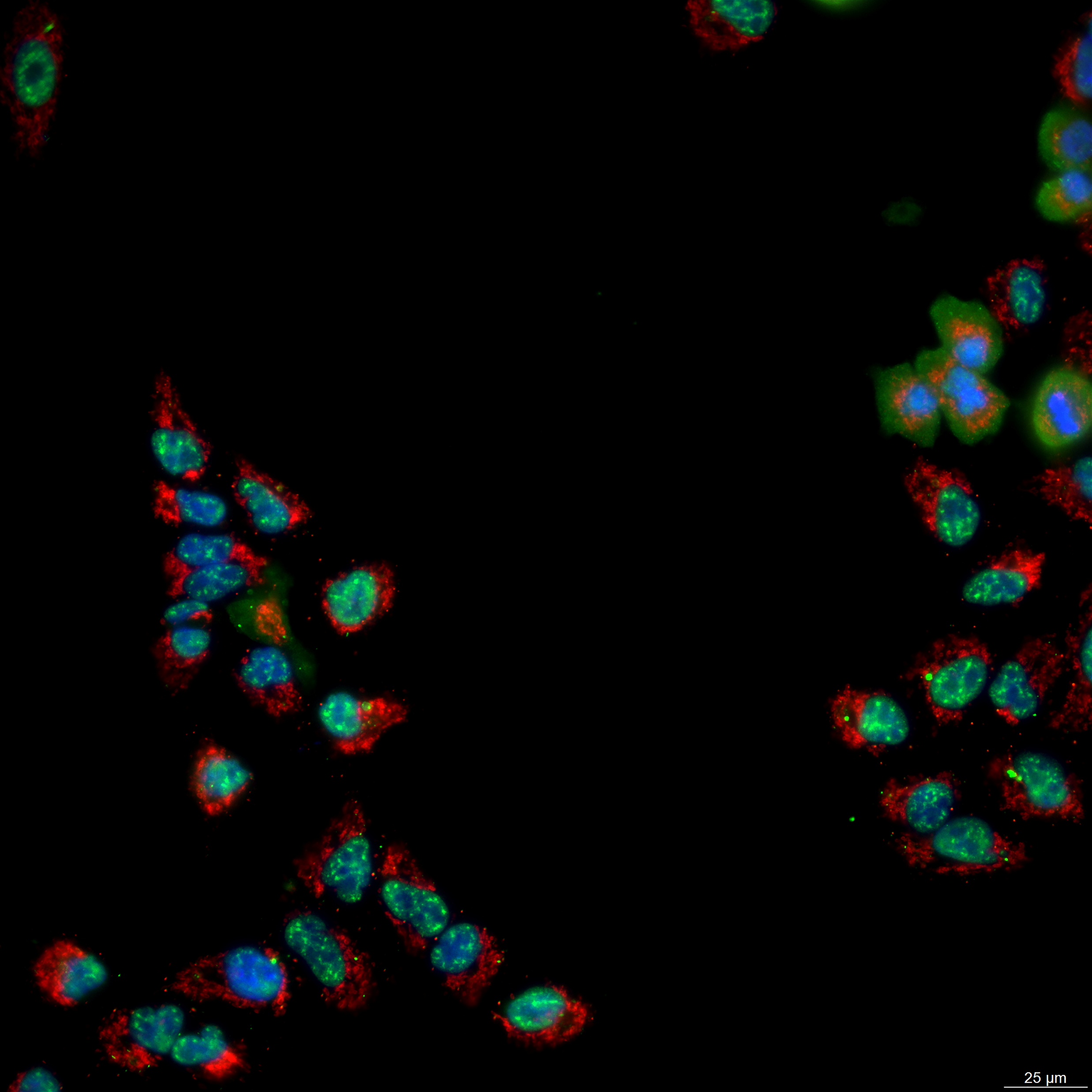

Supplement: Supplementary file 14 — Figure EV3 Source Data [file 44318_2025_421_MOESM14_ESM.zip › EV3/EV3A/Calnexin.tif]

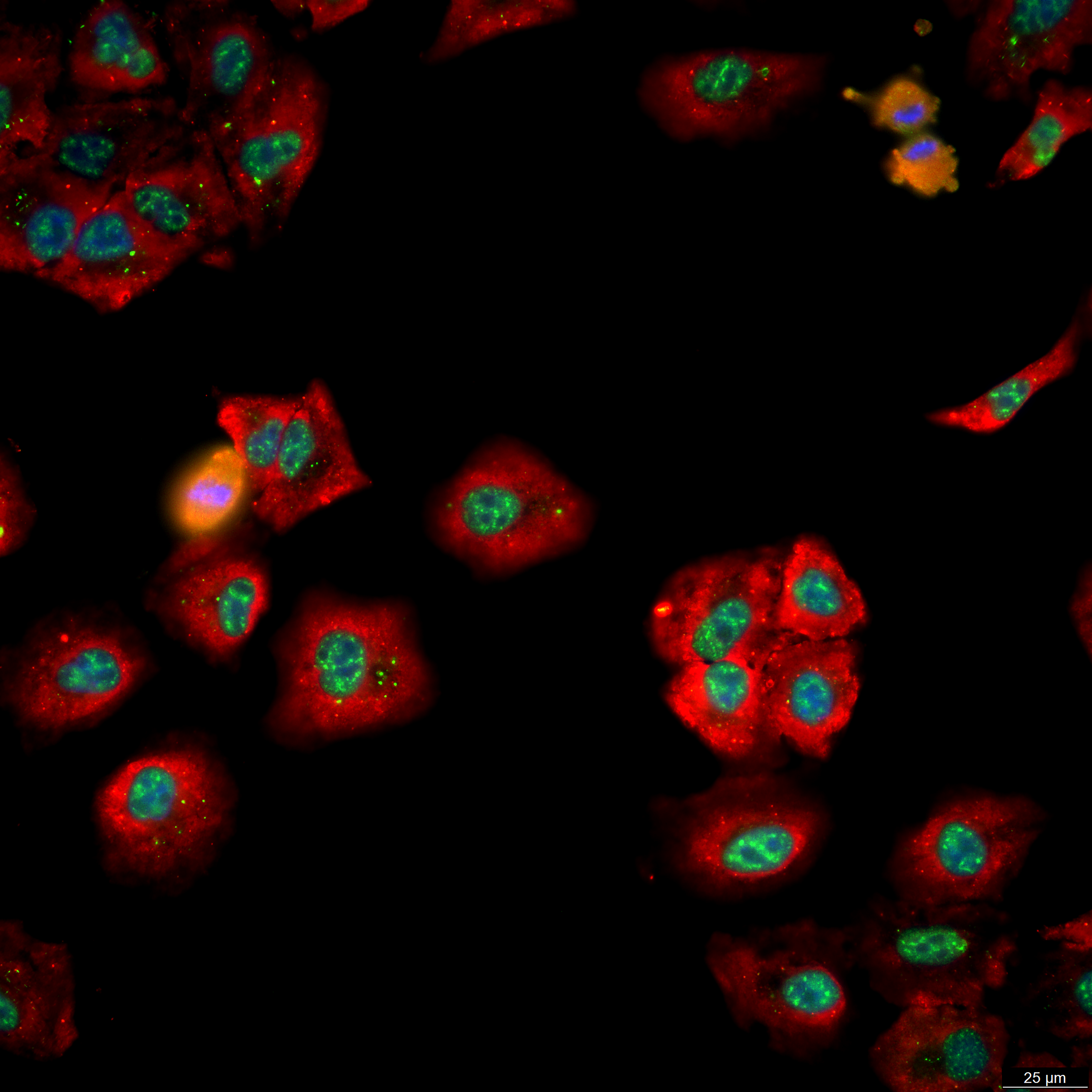

Supplement: Supplementary file 14 — Figure EV3 Source Data [file 44318_2025_421_MOESM14_ESM.zip › EV3/EV3A/DDX6.tif]

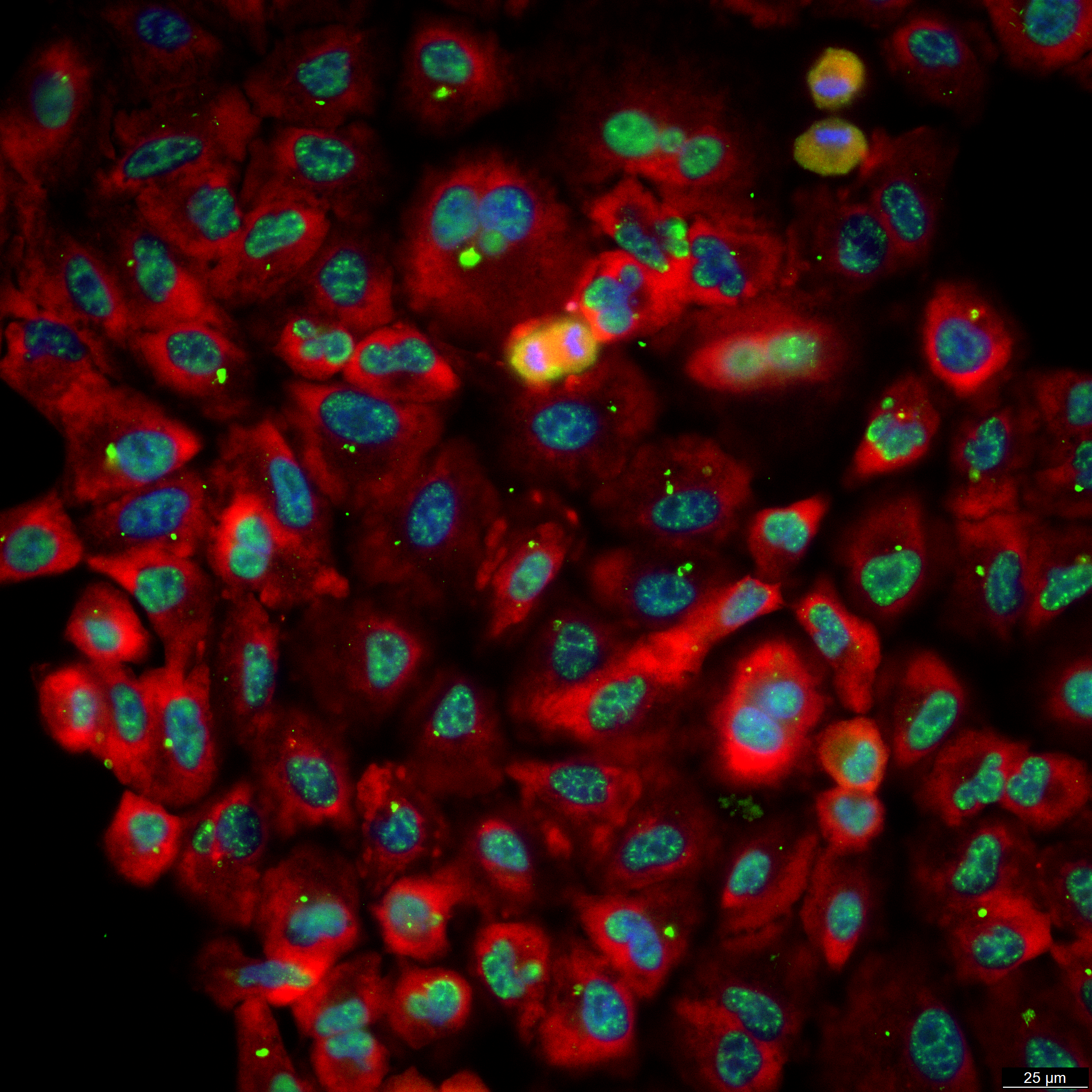

Supplement: Supplementary file 14 — Figure EV3 Source Data [file 44318_2025_421_MOESM14_ESM.zip › EV3/EV3A/G3BP1.tif]

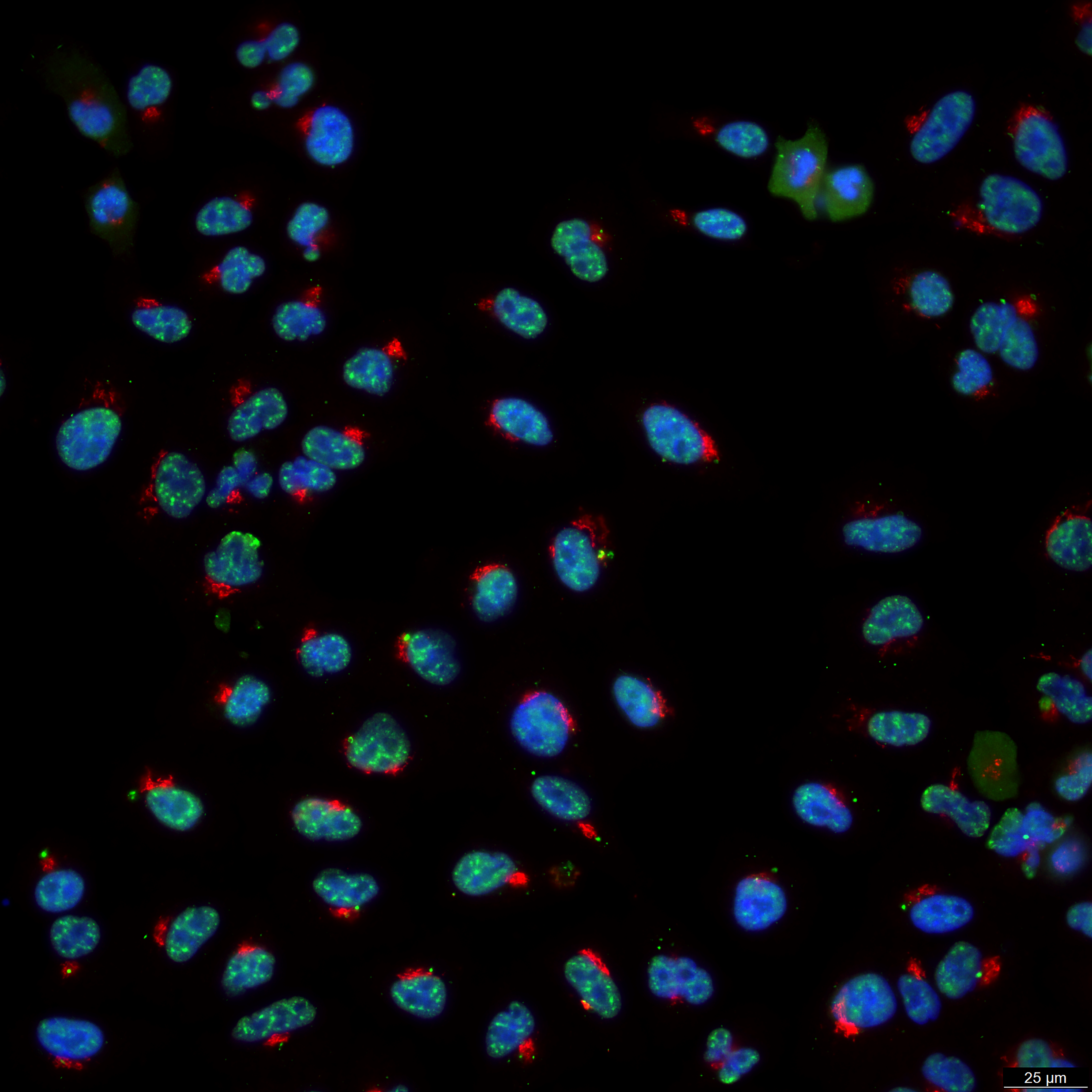

Supplement: Supplementary file 14 — Figure EV3 Source Data [file 44318_2025_421_MOESM14_ESM.zip › EV3/EV3A/GM130.tif]

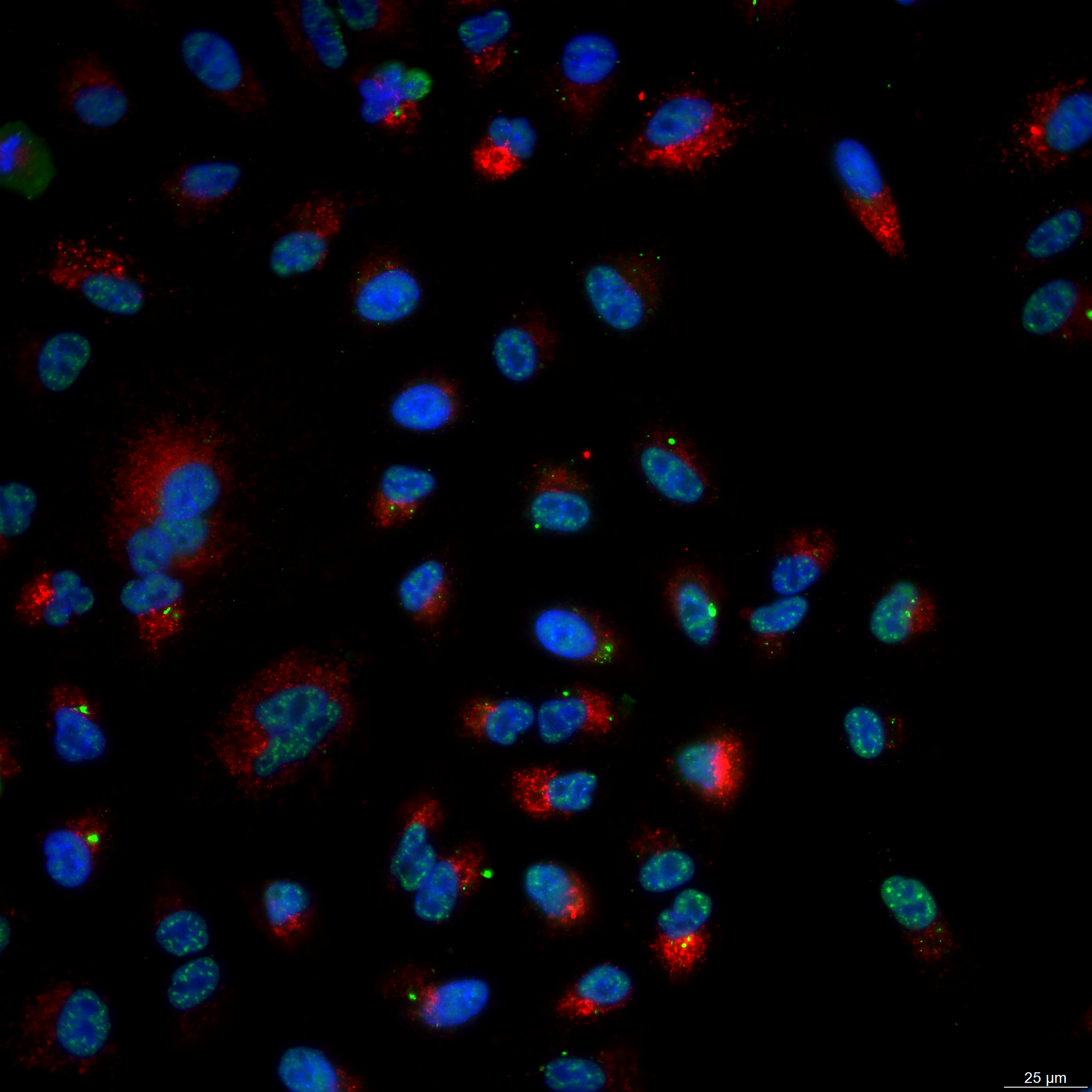

Supplement: Supplementary file 14 — Figure EV3 Source Data [file 44318_2025_421_MOESM14_ESM.zip › EV3/EV3A/LAMP1.tif]

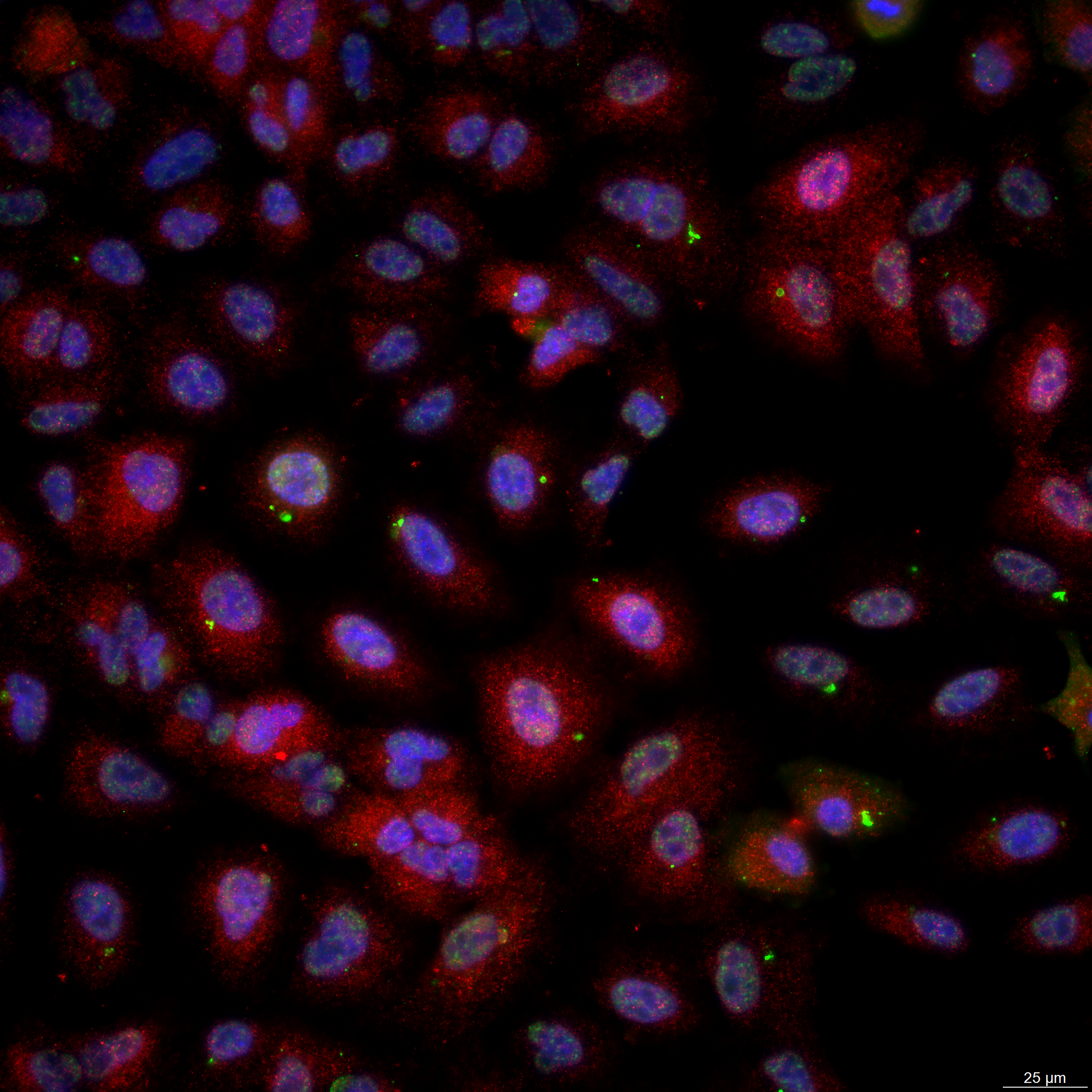

Supplement: Supplementary file 14 — Figure EV3 Source Data [file 44318_2025_421_MOESM14_ESM.zip › EV3/EV3A/LMP7.tif]

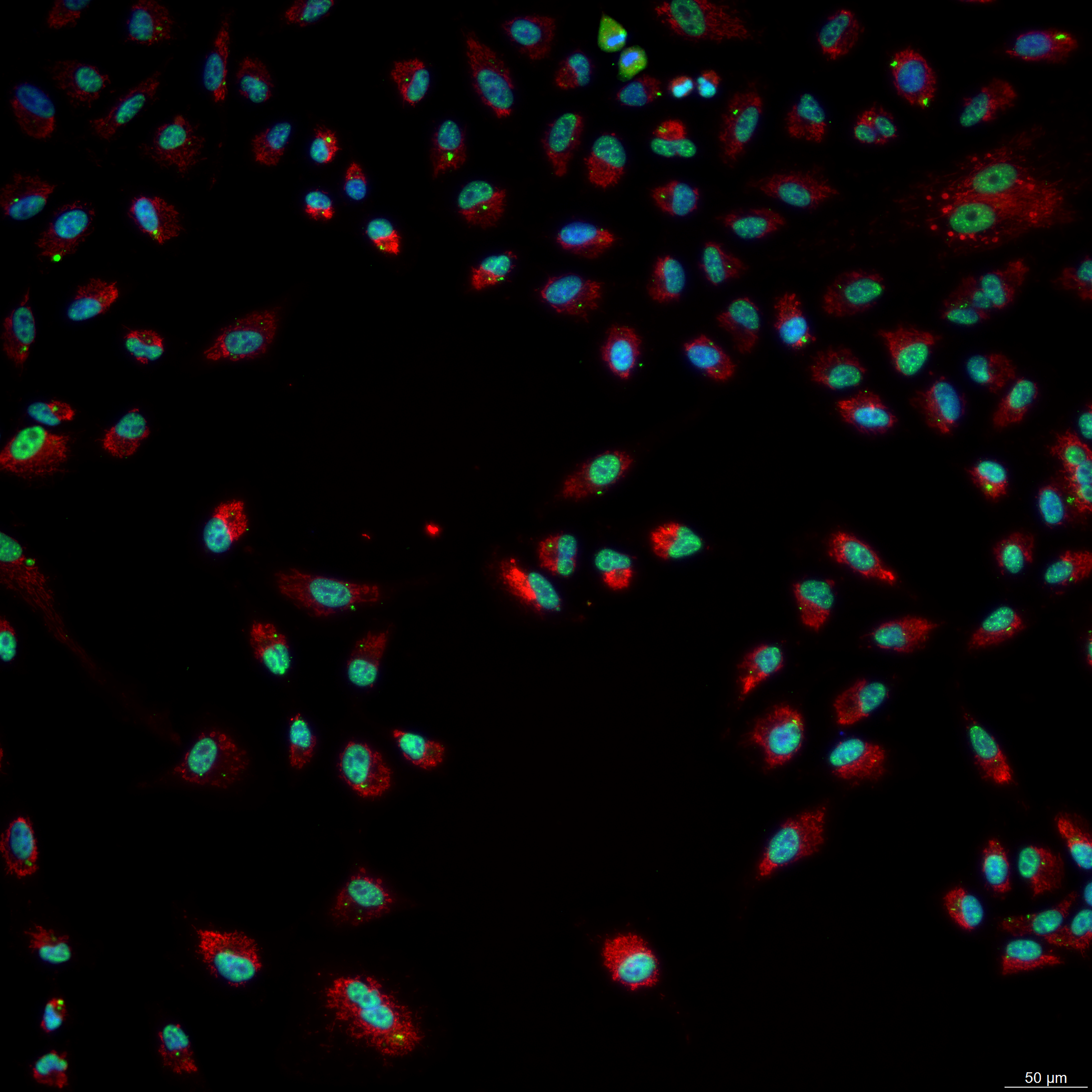

Supplement: Supplementary file 14 — Figure EV3 Source Data [file 44318_2025_421_MOESM14_ESM.zip › EV3/EV3A/Mitotracker.tif]

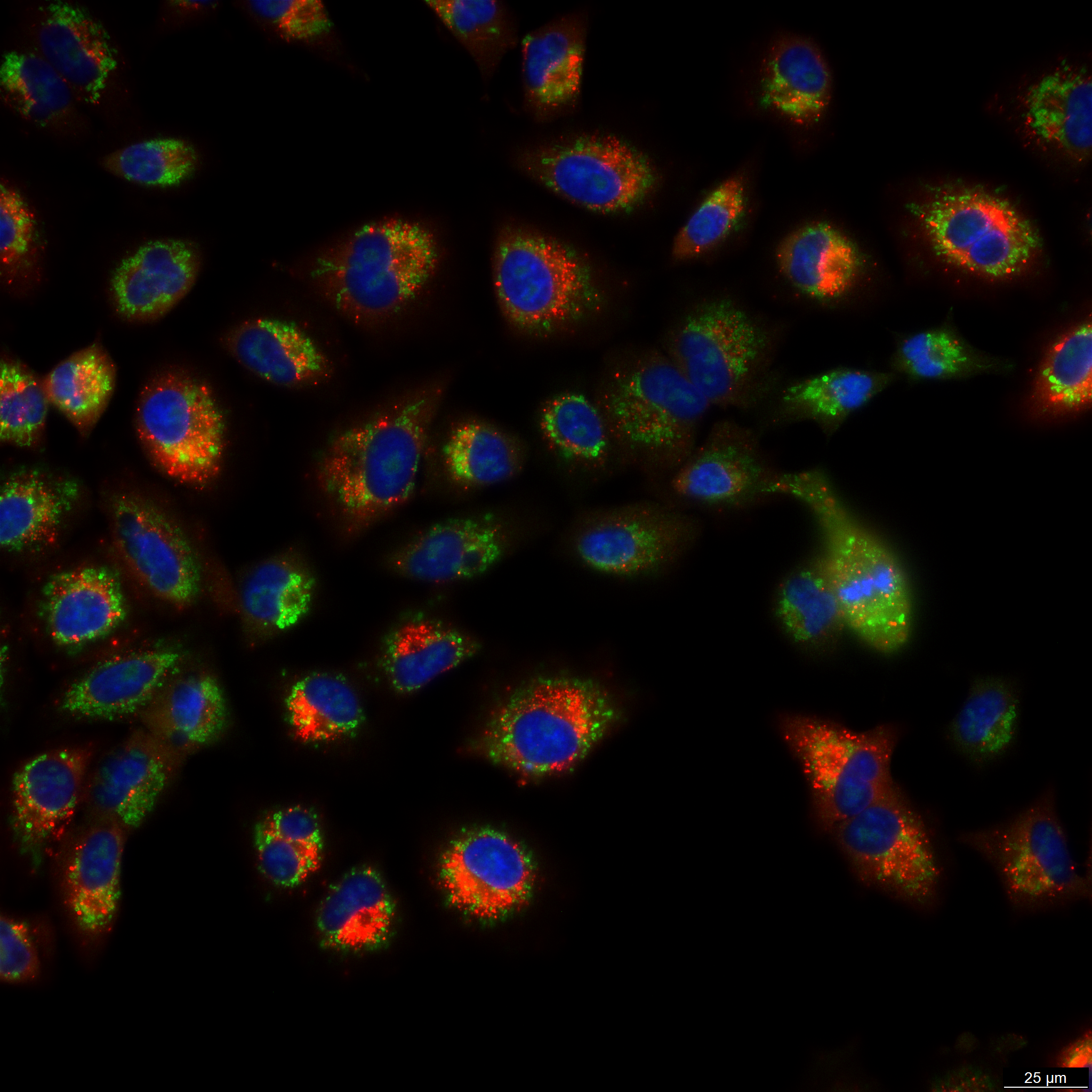

Supplement: Supplementary file 14 — Figure EV3 Source Data [file 44318_2025_421_MOESM14_ESM.zip › EV3/EV3C/Control.tif]

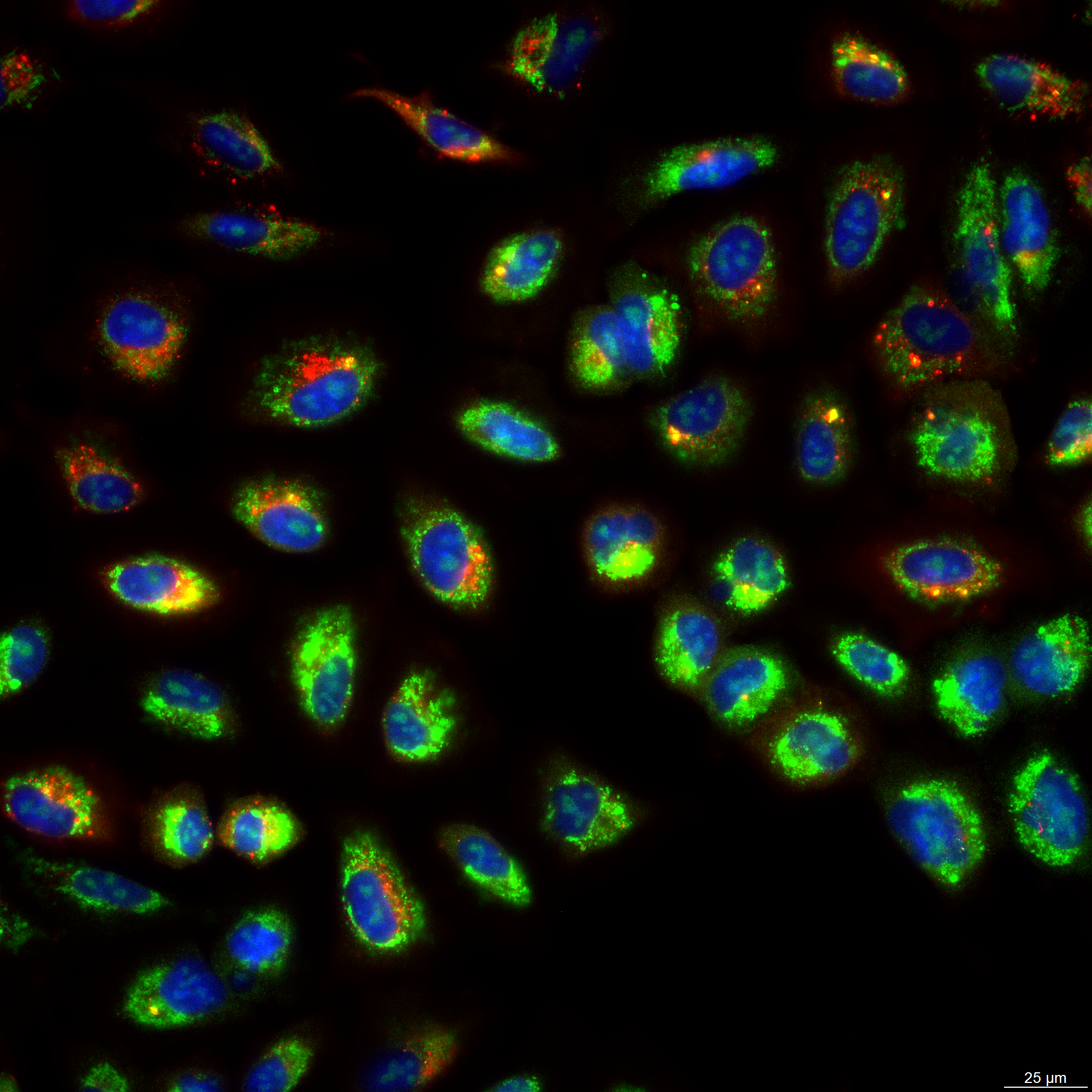

Supplement: Supplementary file 14 — Figure EV3 Source Data [file 44318_2025_421_MOESM14_ESM.zip › EV3/EV3C/lFNγ.tif]

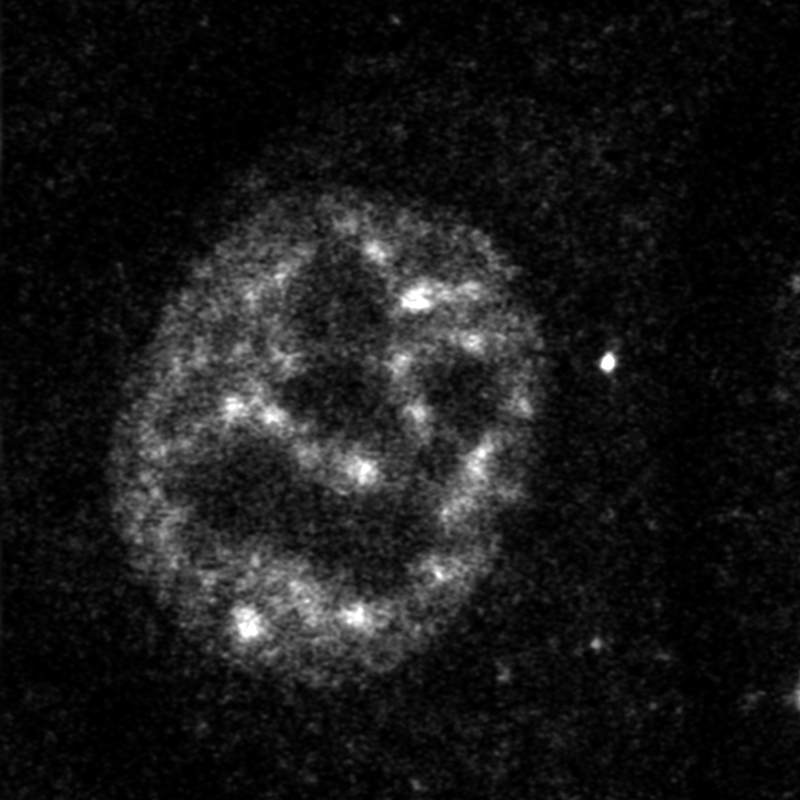

Supplement: Supplementary file 14 — Figure EV3 Source Data [file 44318_2025_421_MOESM14_ESM.zip › EV3/EV3F/Control.tif]

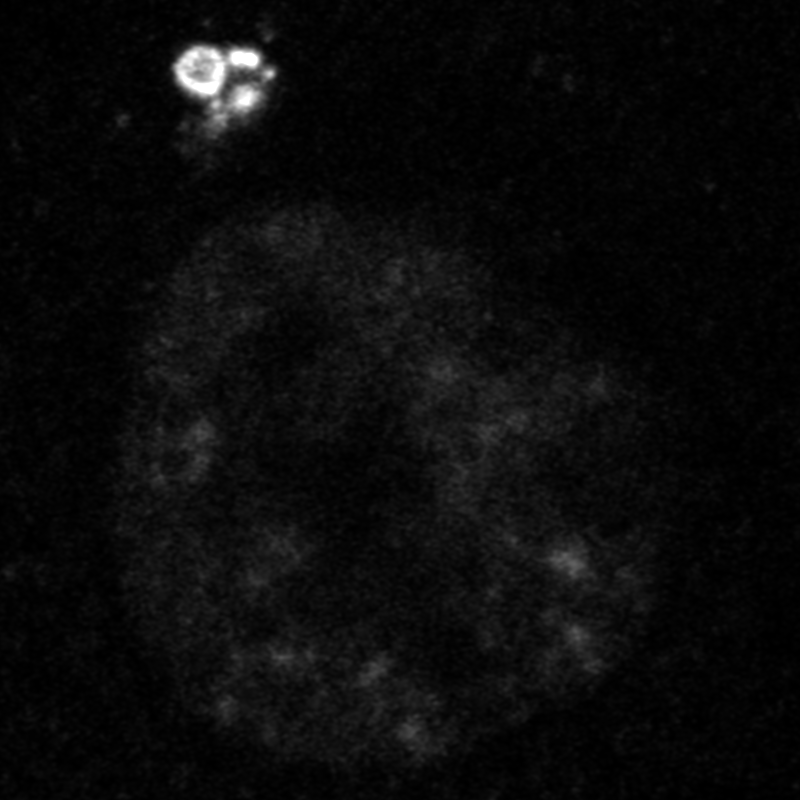

Supplement: Supplementary file 14 — Figure EV3 Source Data [file 44318_2025_421_MOESM14_ESM.zip › EV3/EV3F/IFN.tif]

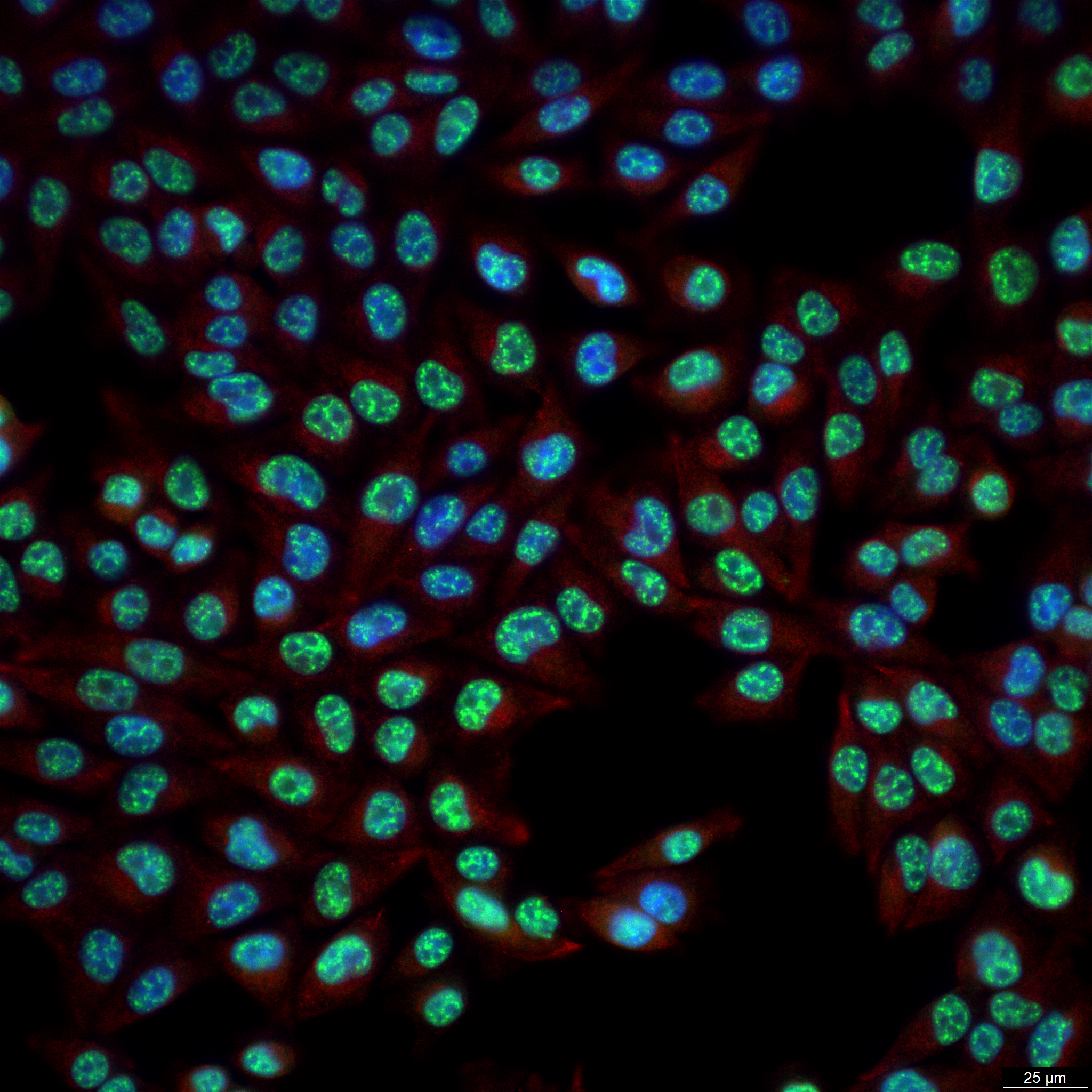

Supplement: Supplementary file 14 — Figure EV3 Source Data [file 44318_2025_421_MOESM14_ESM.zip › EV3/EV3H Left panel/CONTROL 1.tif]

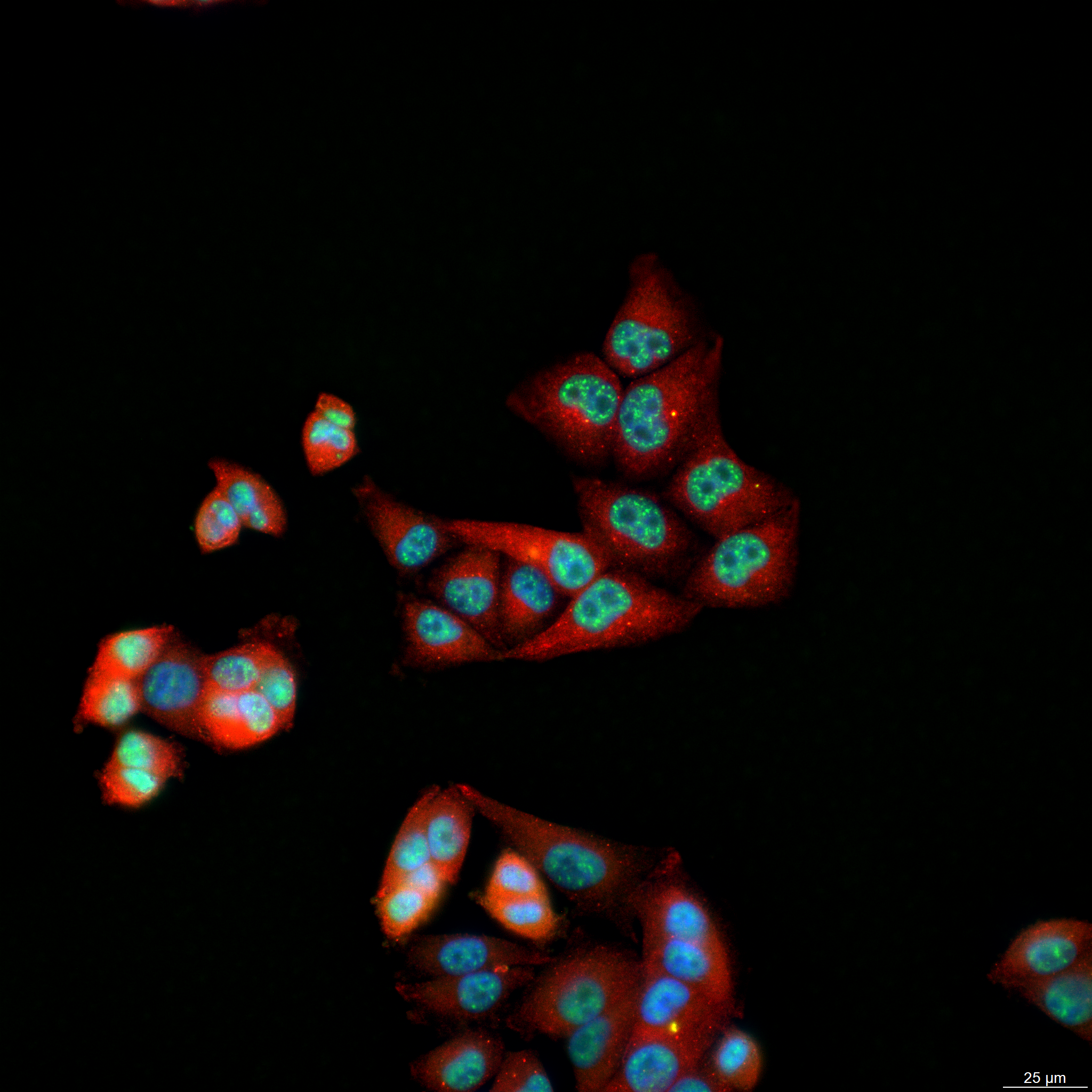

Supplement: Supplementary file 14 — Figure EV3 Source Data [file 44318_2025_421_MOESM14_ESM.zip › EV3/EV3H Left panel/IFNγ.tif]

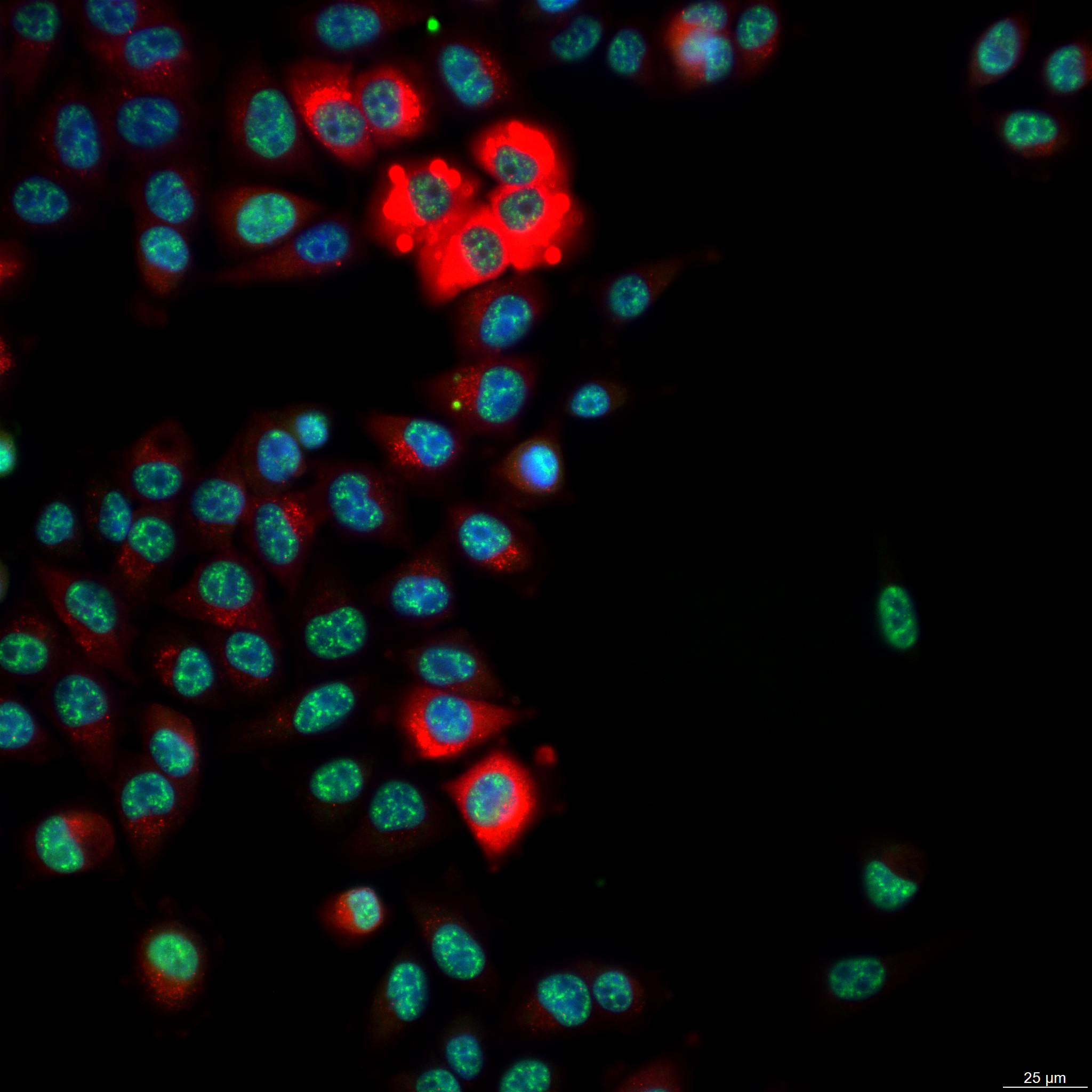

Supplement: Supplementary file 14 — Figure EV3 Source Data [file 44318_2025_421_MOESM14_ESM.zip › EV3/EV3H Right panel/Control .tif]

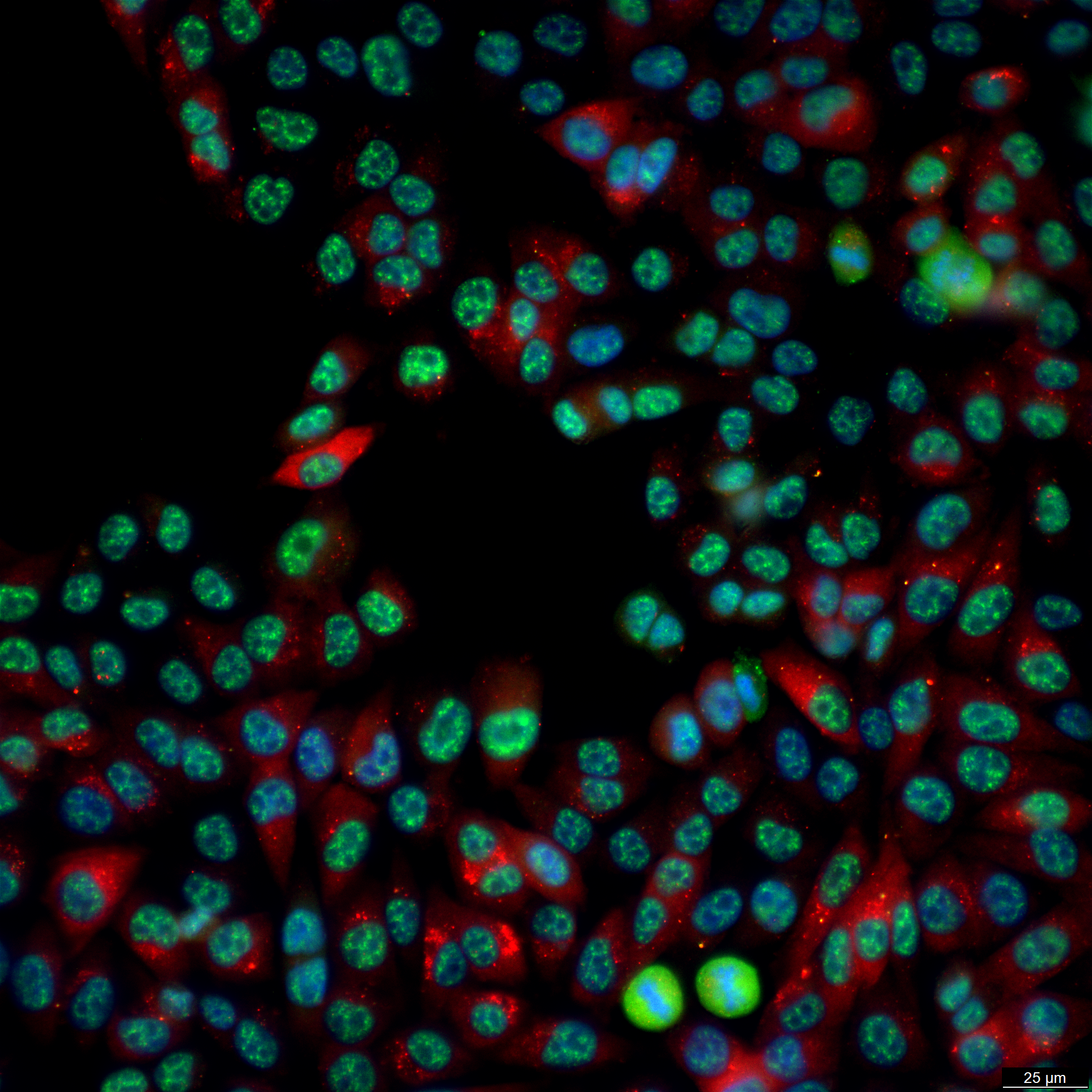

Supplement: Supplementary file 14 — Figure EV3 Source Data [file 44318_2025_421_MOESM14_ESM.zip › EV3/EV3H Right panel/IFNγ.tif]

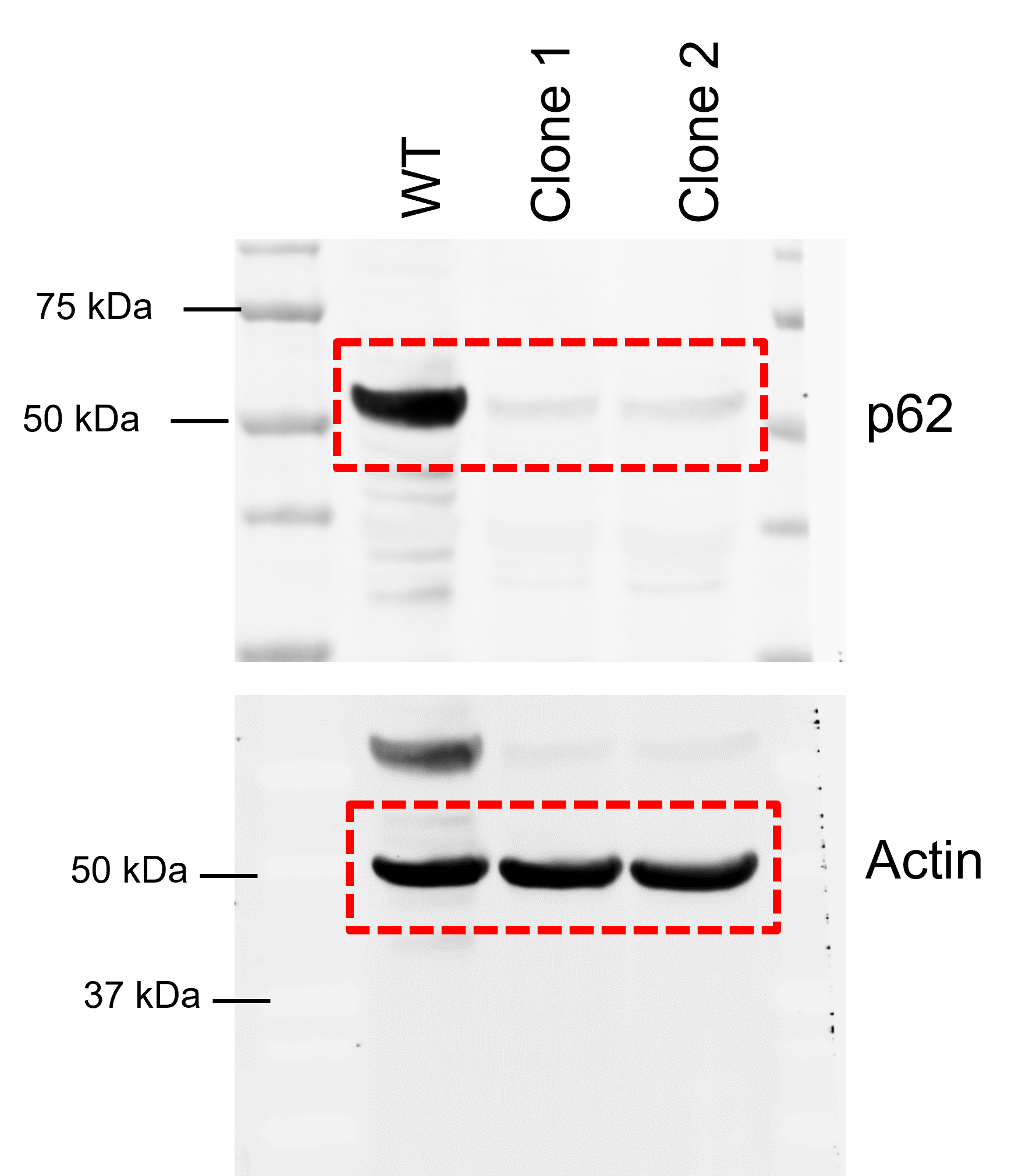

Supplement: Supplementary file 14 — Figure EV3 Source Data [file 44318_2025_421_MOESM14_ESM.zip › EV3/EV3K.tif]

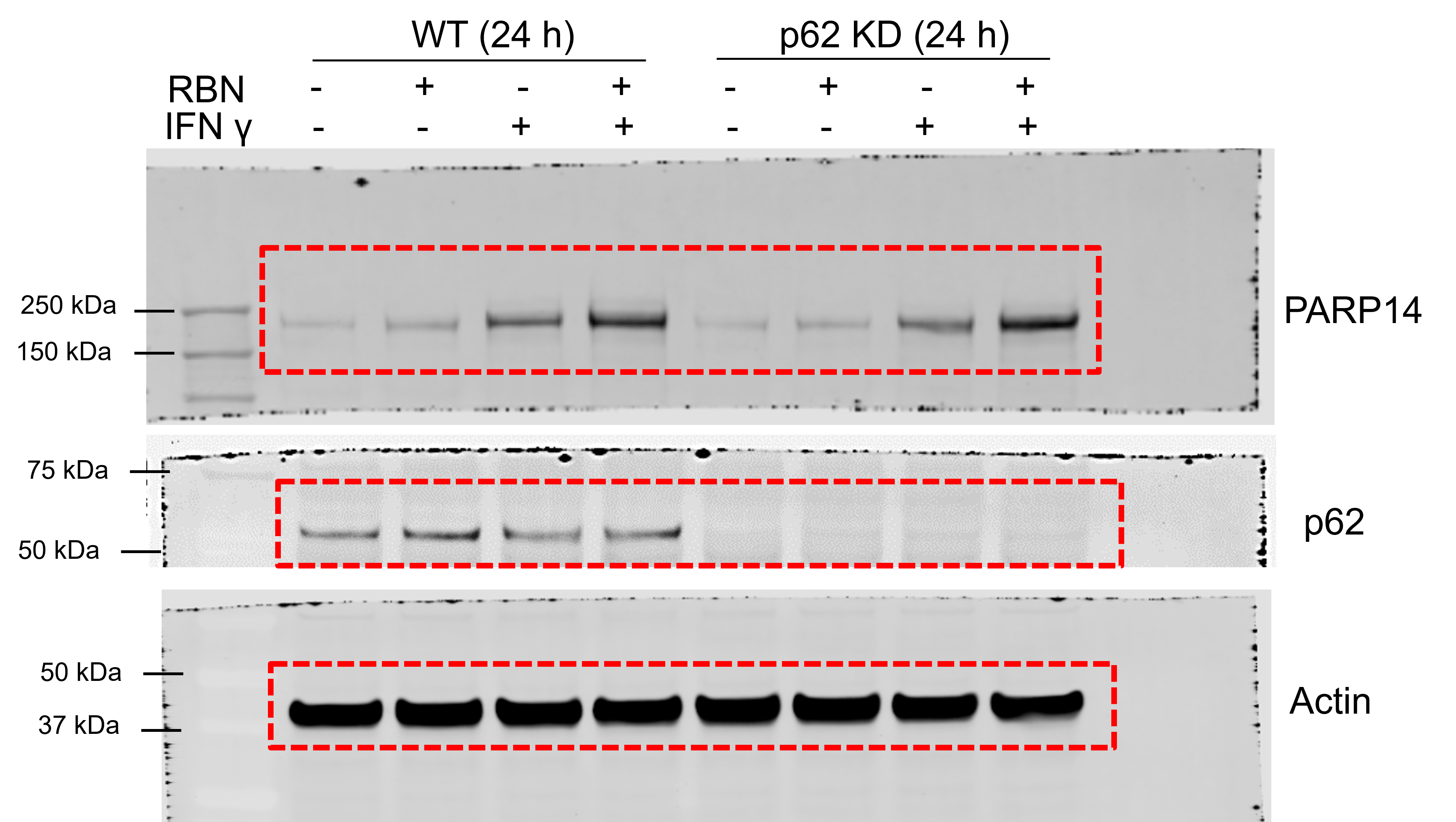

Supplement: Supplementary file 14 — Figure EV3 Source Data [file 44318_2025_421_MOESM14_ESM.zip › EV3/EV3L.tif]

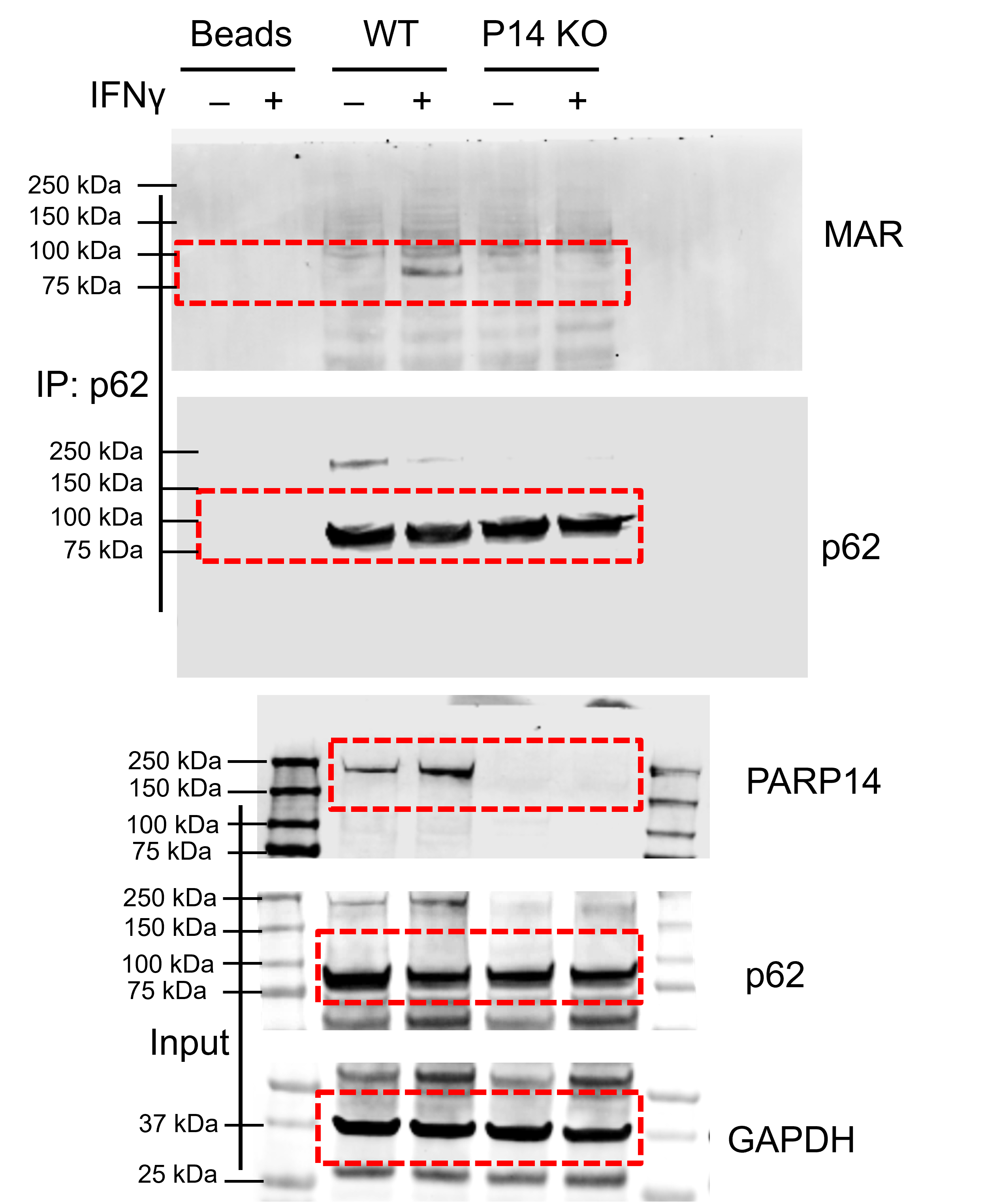

Supplement: Supplementary file 15 — Figure EV4 Source Data [file 44318_2025_421_MOESM15_ESM.zip › EV4/EV4B.tif]

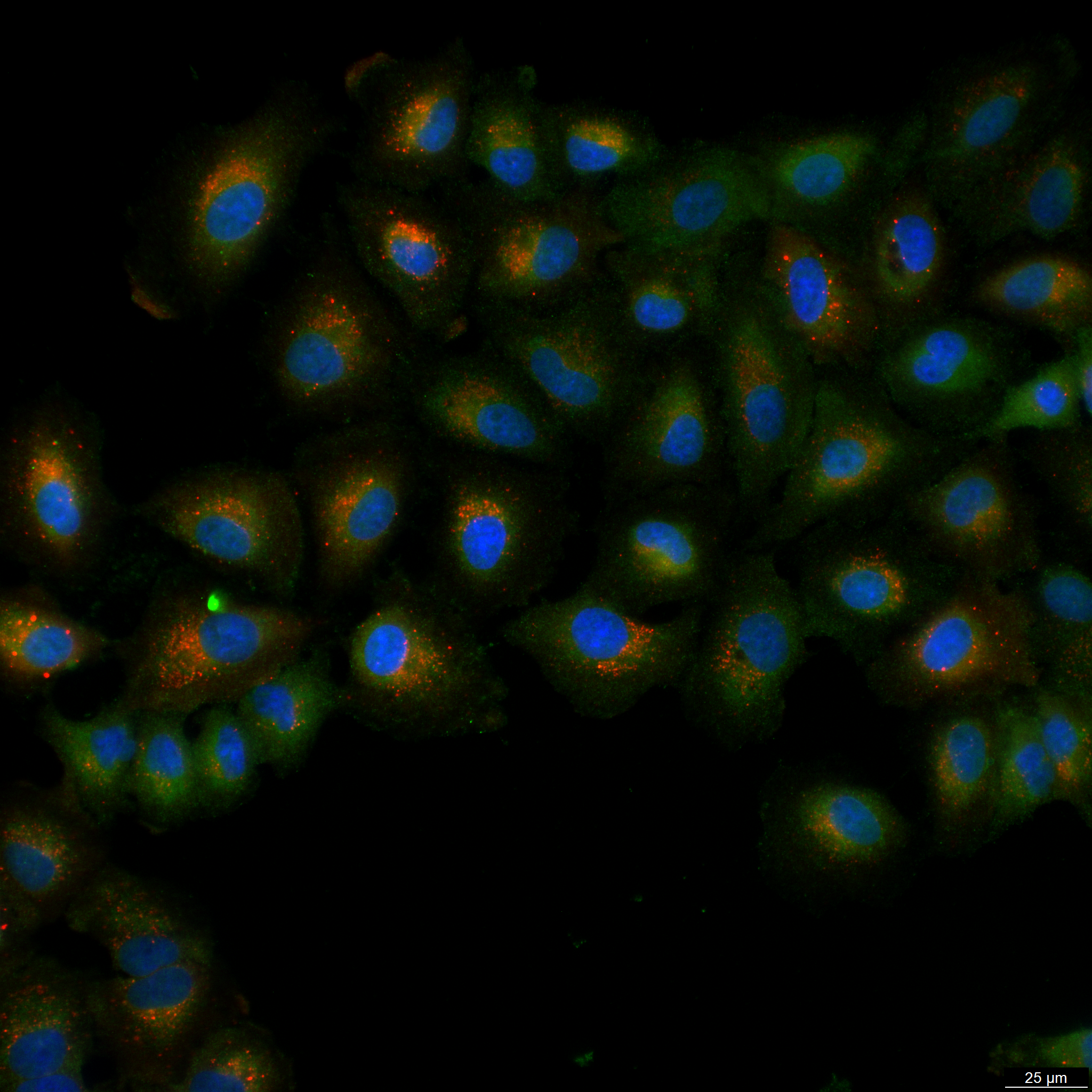

Supplement: Supplementary file 15 — Figure EV4 Source Data [file 44318_2025_421_MOESM15_ESM.zip › EV4/EV4C/Control.tif]

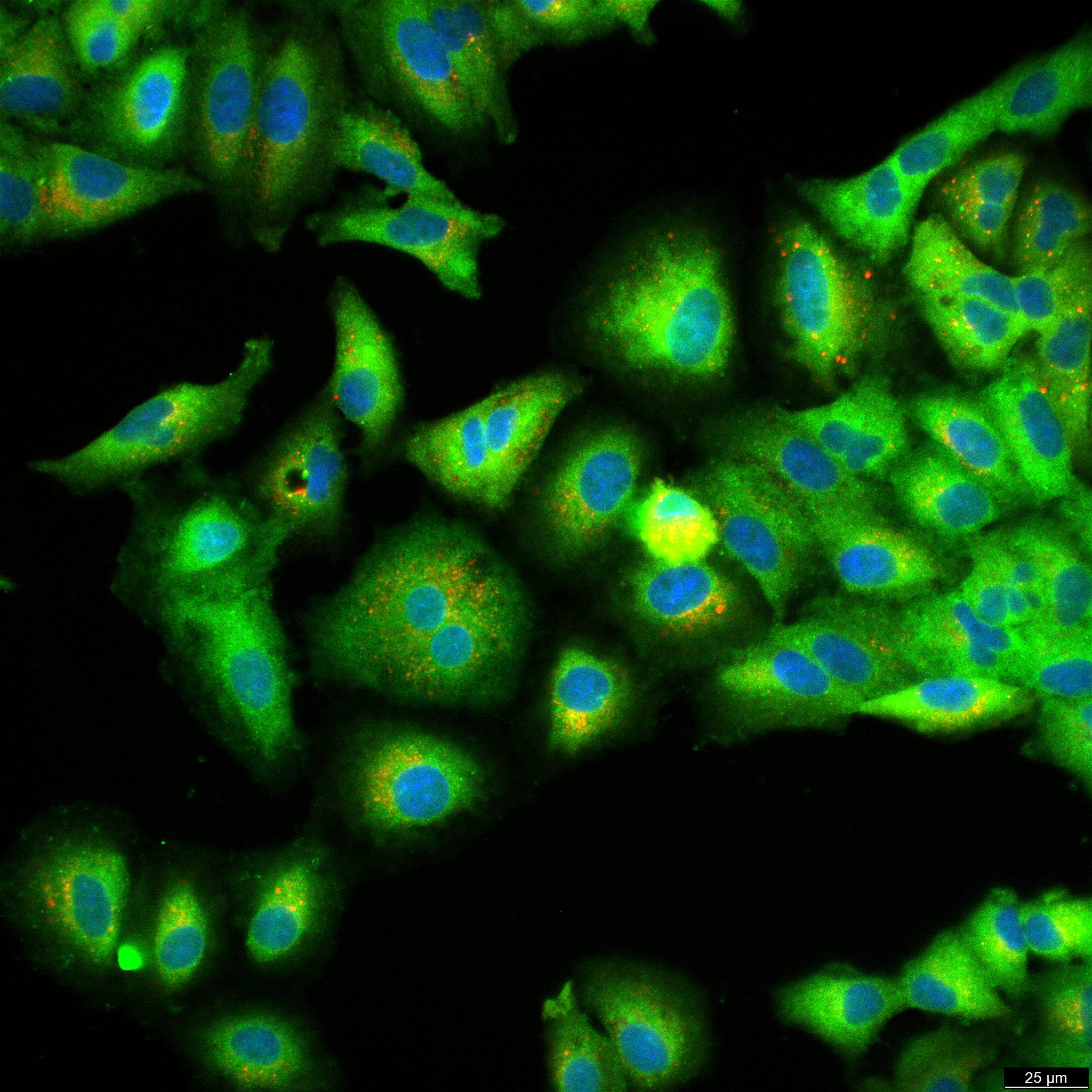

Supplement: Supplementary file 15 — Figure EV4 Source Data [file 44318_2025_421_MOESM15_ESM.zip › EV4/EV4C/lFNγ+RBN.tif]

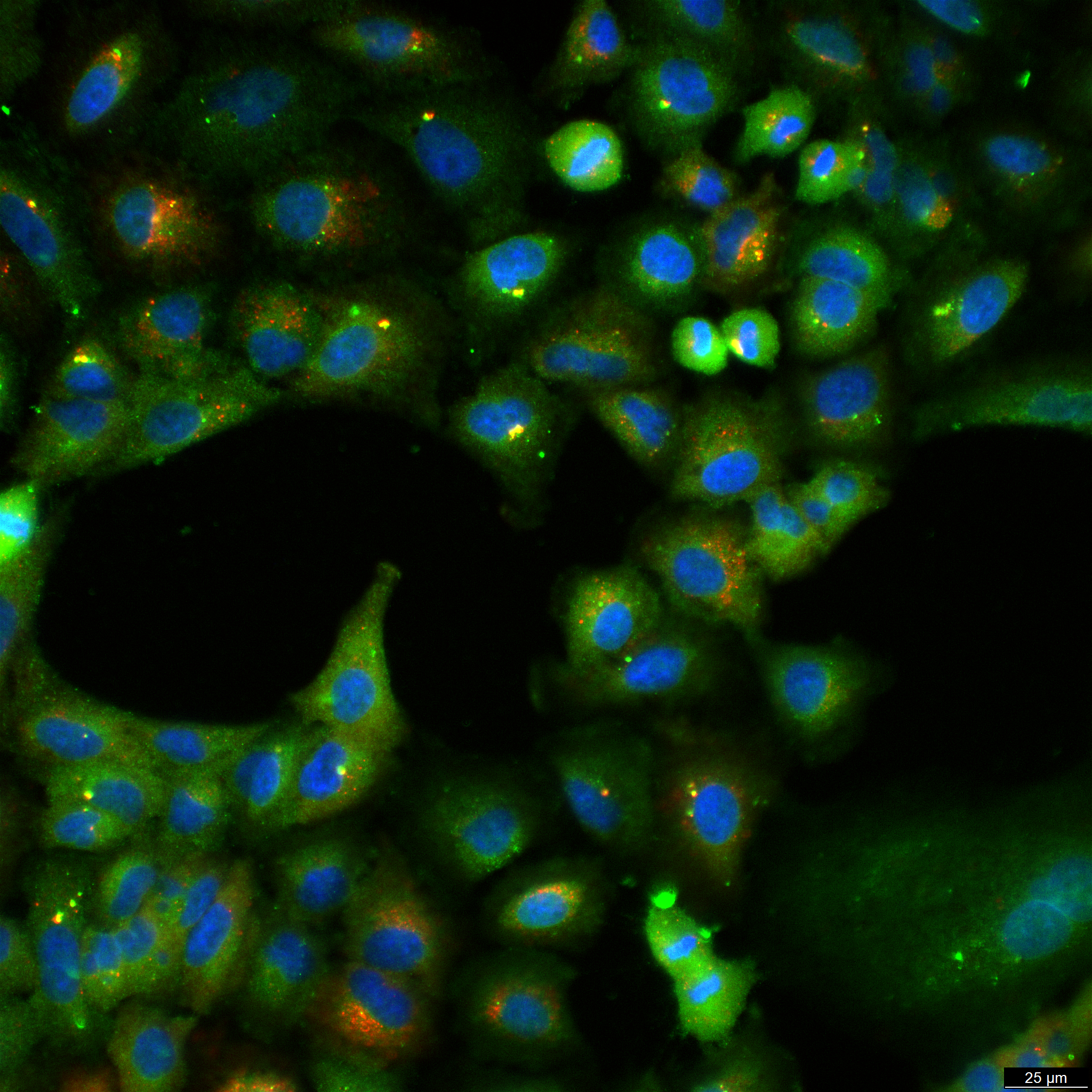

Supplement: Supplementary file 15 — Figure EV4 Source Data [file 44318_2025_421_MOESM15_ESM.zip › EV4/EV4C/lFNγ.tif]

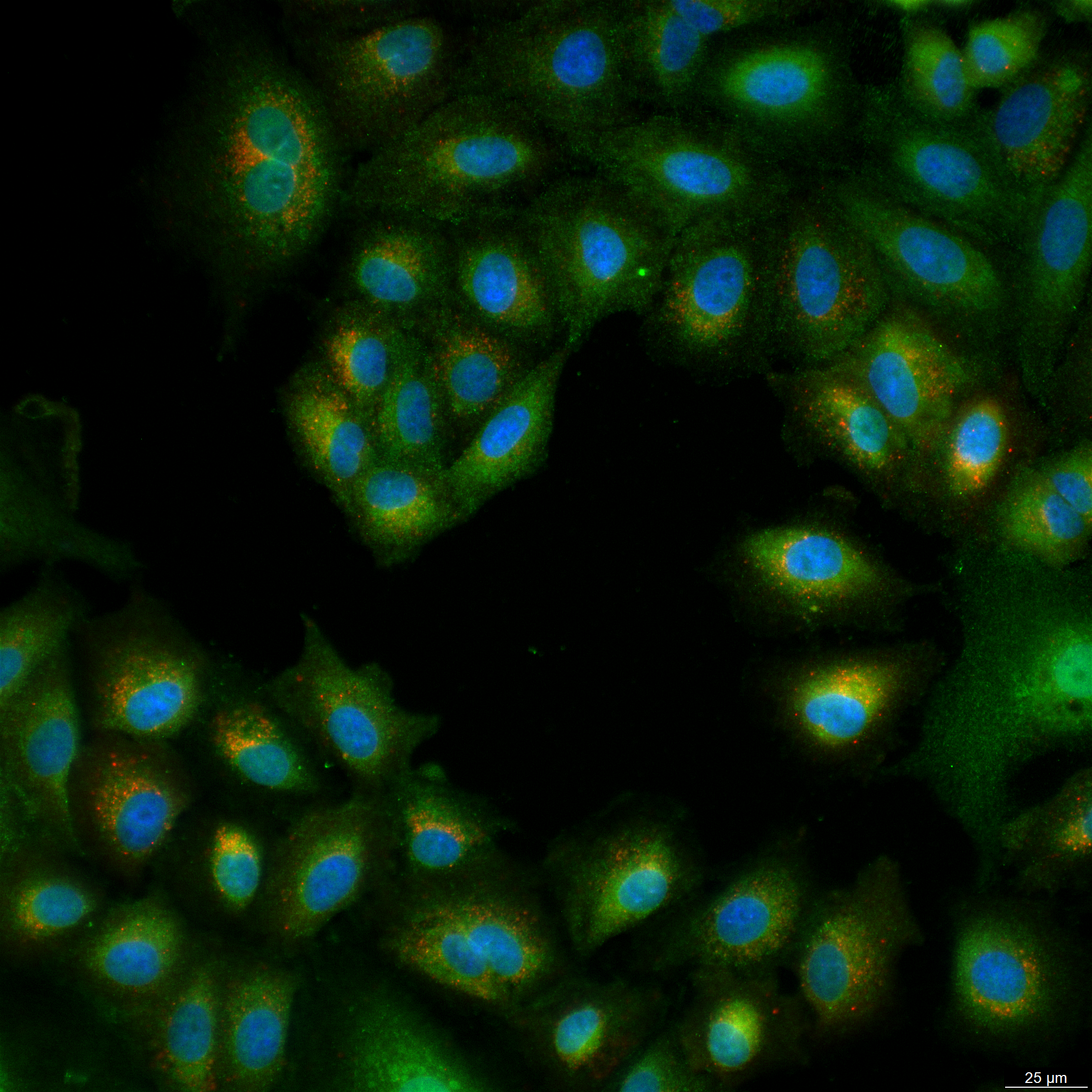

Supplement: Supplementary file 15 — Figure EV4 Source Data [file 44318_2025_421_MOESM15_ESM.zip › EV4/EV4C/RBN.tif]

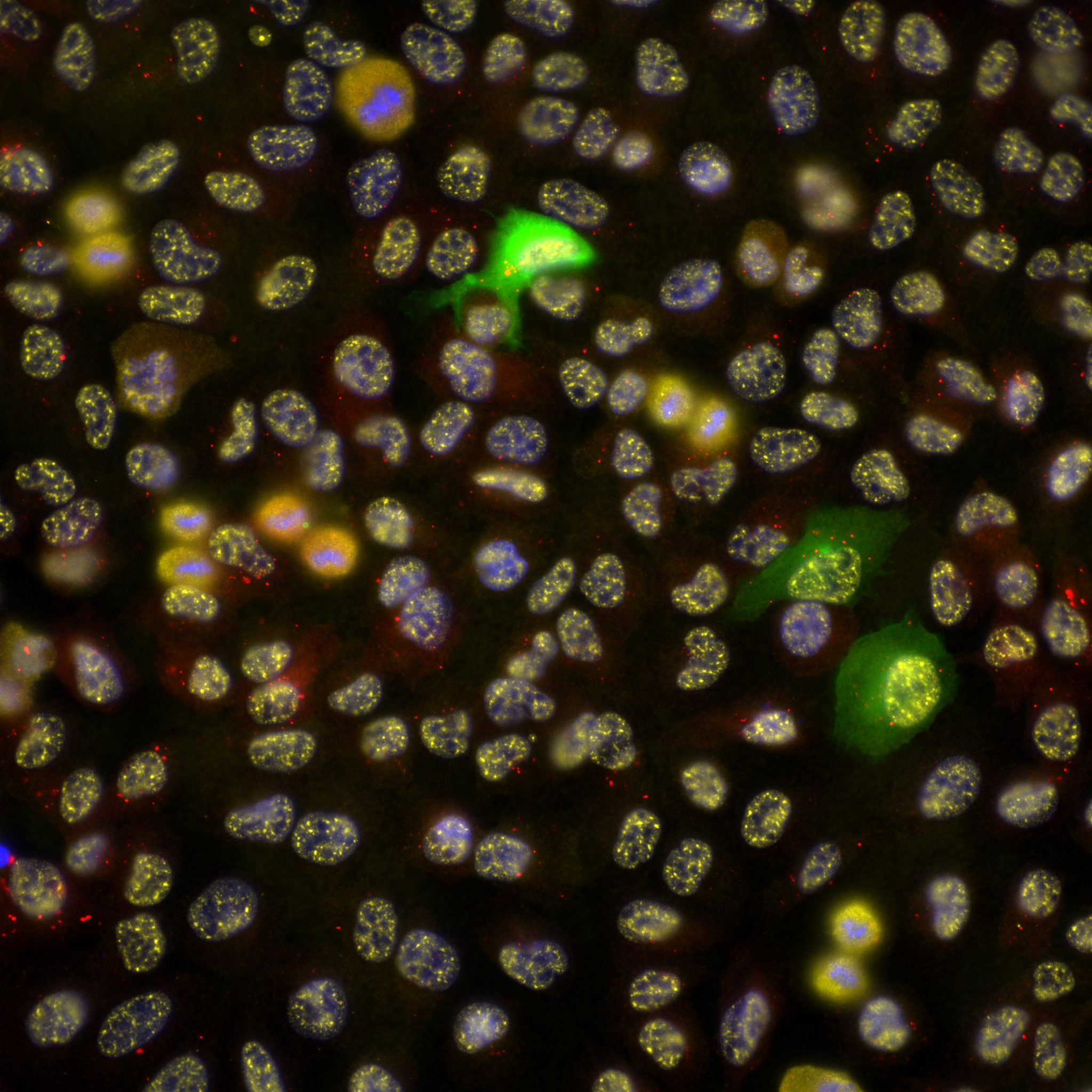

Supplement: Supplementary file 15 — Figure EV4 Source Data [file 44318_2025_421_MOESM15_ESM.zip › EV4/EV4D/Cell_1.tif]

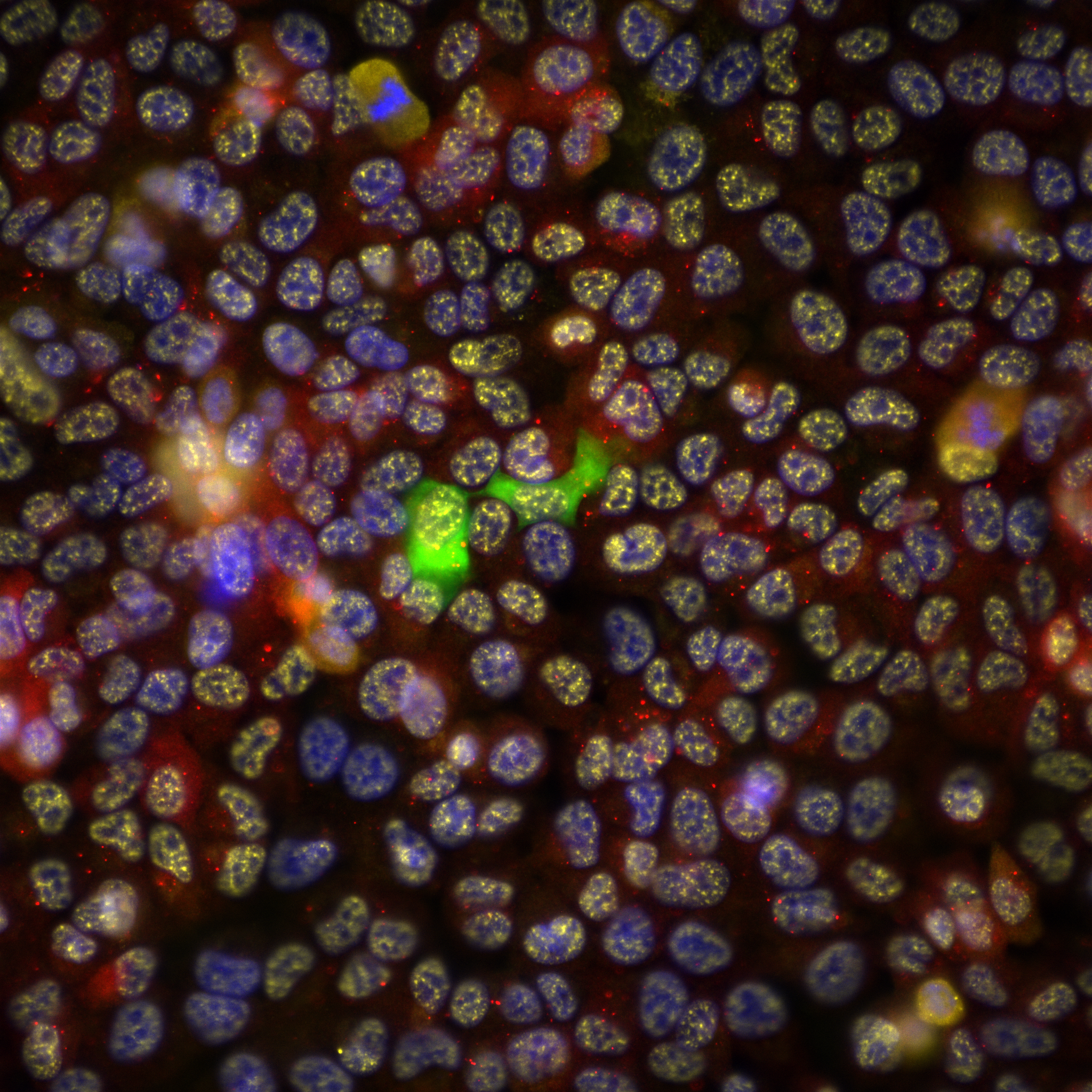

Supplement: Supplementary file 15 — Figure EV4 Source Data [file 44318_2025_421_MOESM15_ESM.zip › EV4/EV4D/Cell_10_11.tif]

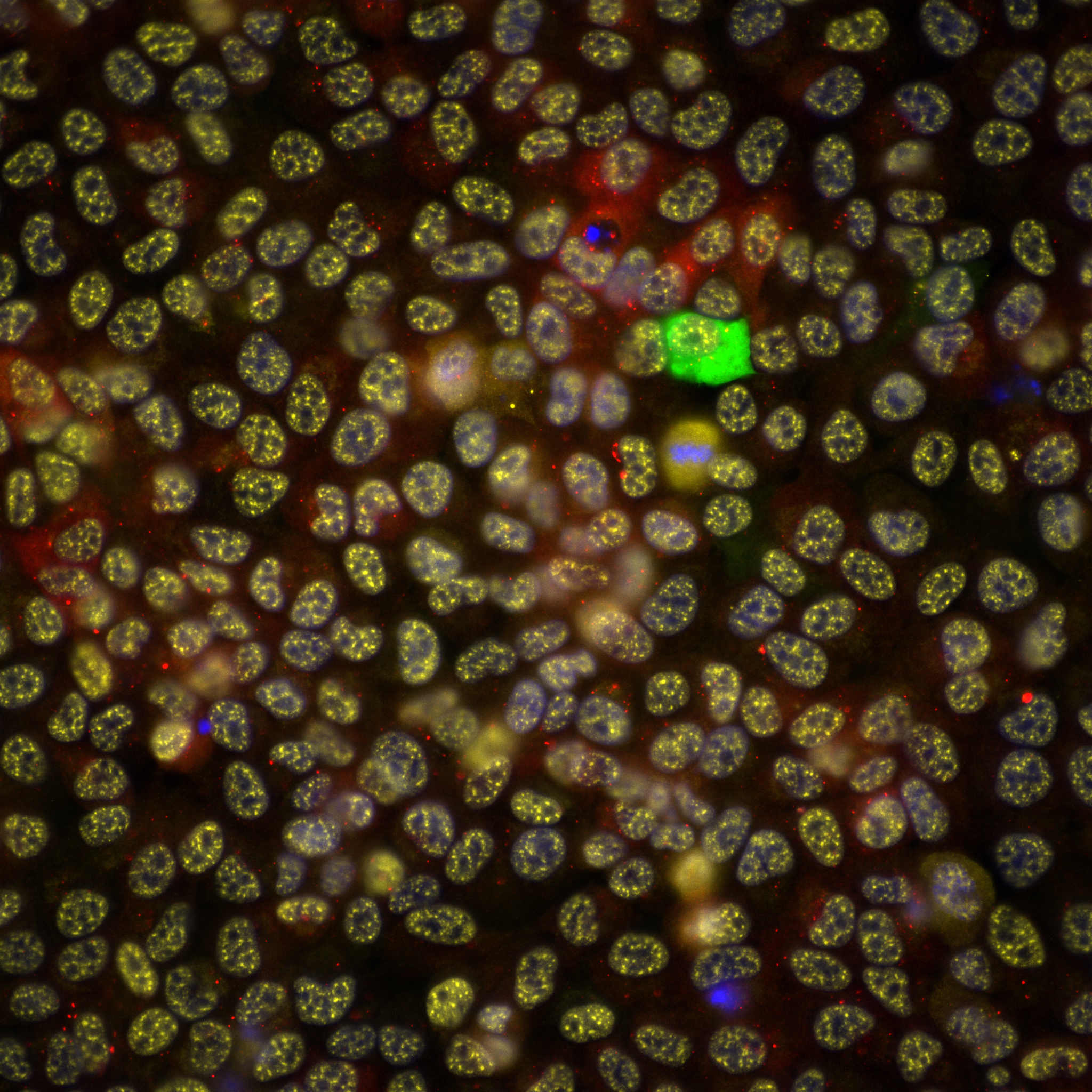

Supplement: Supplementary file 15 — Figure EV4 Source Data [file 44318_2025_421_MOESM15_ESM.zip › EV4/EV4D/Cell_12.tif]

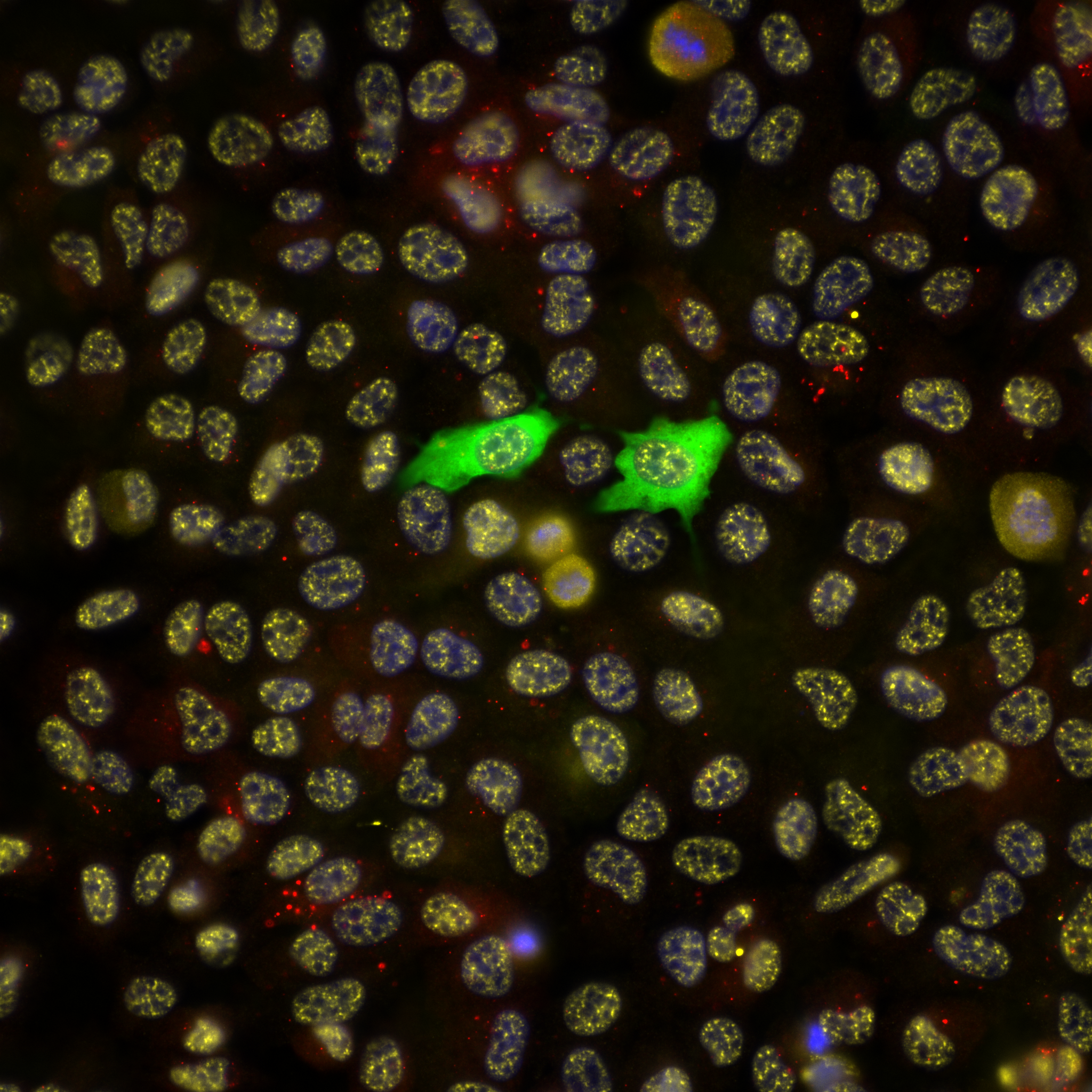

Supplement: Supplementary file 15 — Figure EV4 Source Data [file 44318_2025_421_MOESM15_ESM.zip › EV4/EV4D/Cell_2_3.tif]

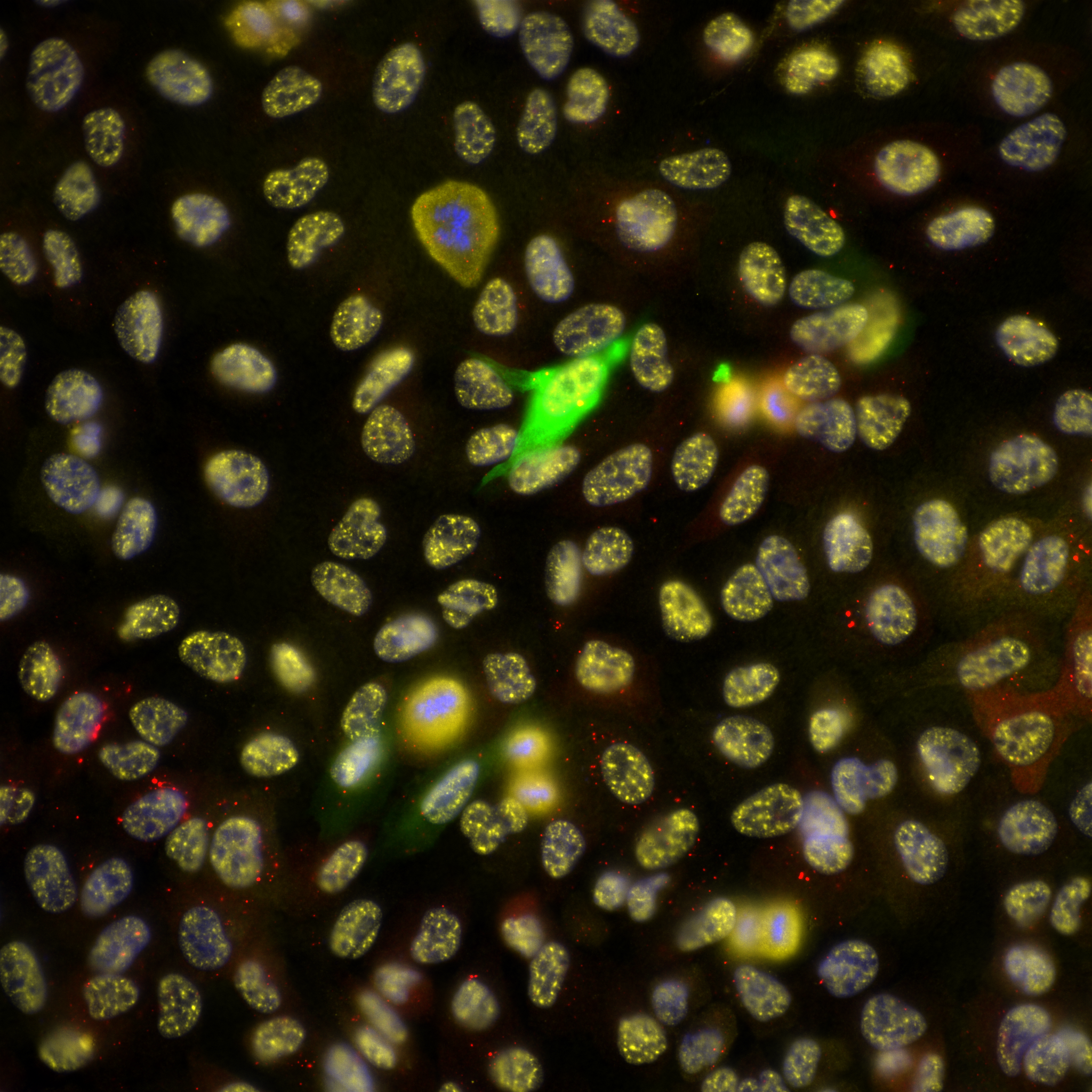

Supplement: Supplementary file 15 — Figure EV4 Source Data [file 44318_2025_421_MOESM15_ESM.zip › EV4/EV4D/Cell_4.tif]

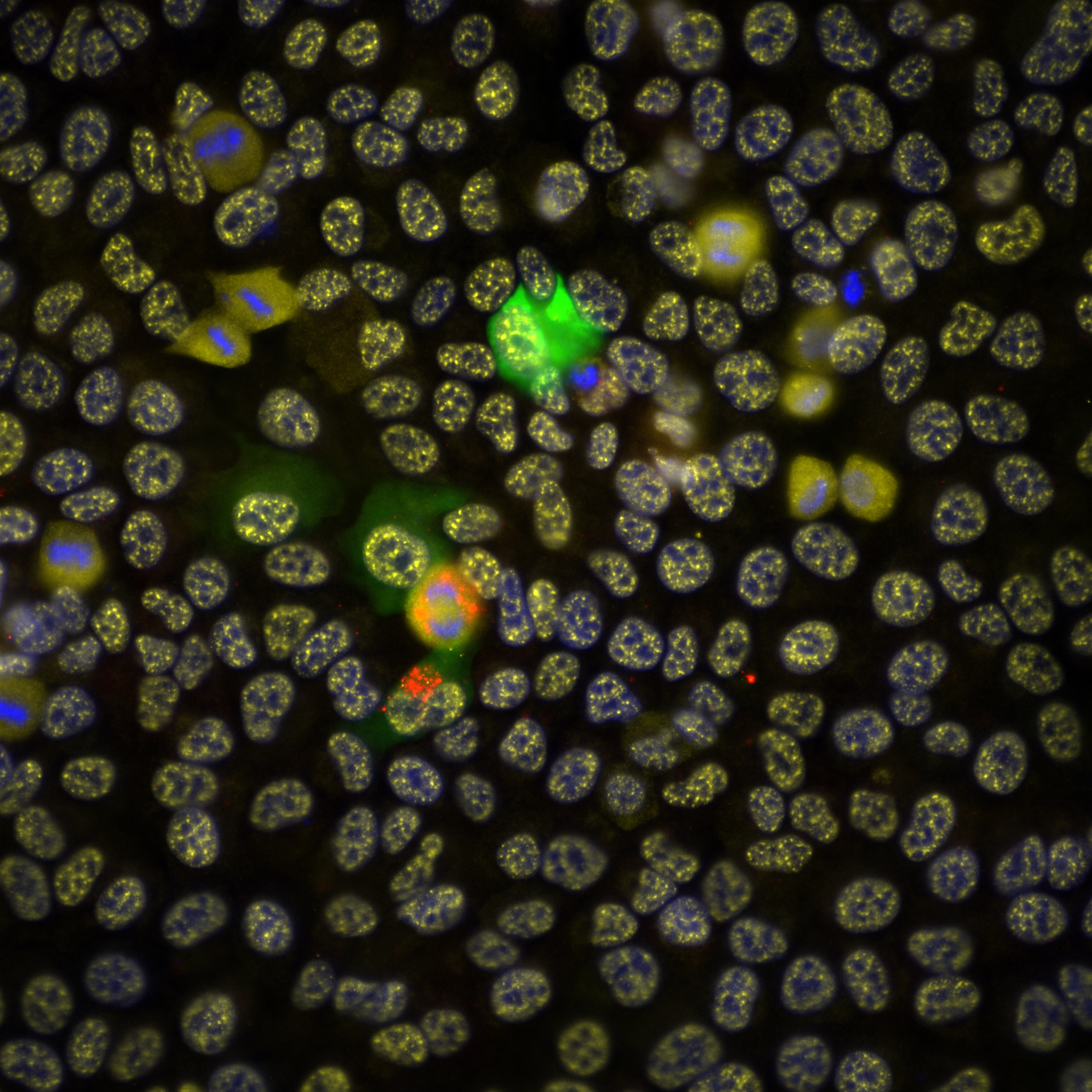

Supplement: Supplementary file 15 — Figure EV4 Source Data [file 44318_2025_421_MOESM15_ESM.zip › EV4/EV4D/Cell_5_6_7_8.tif]

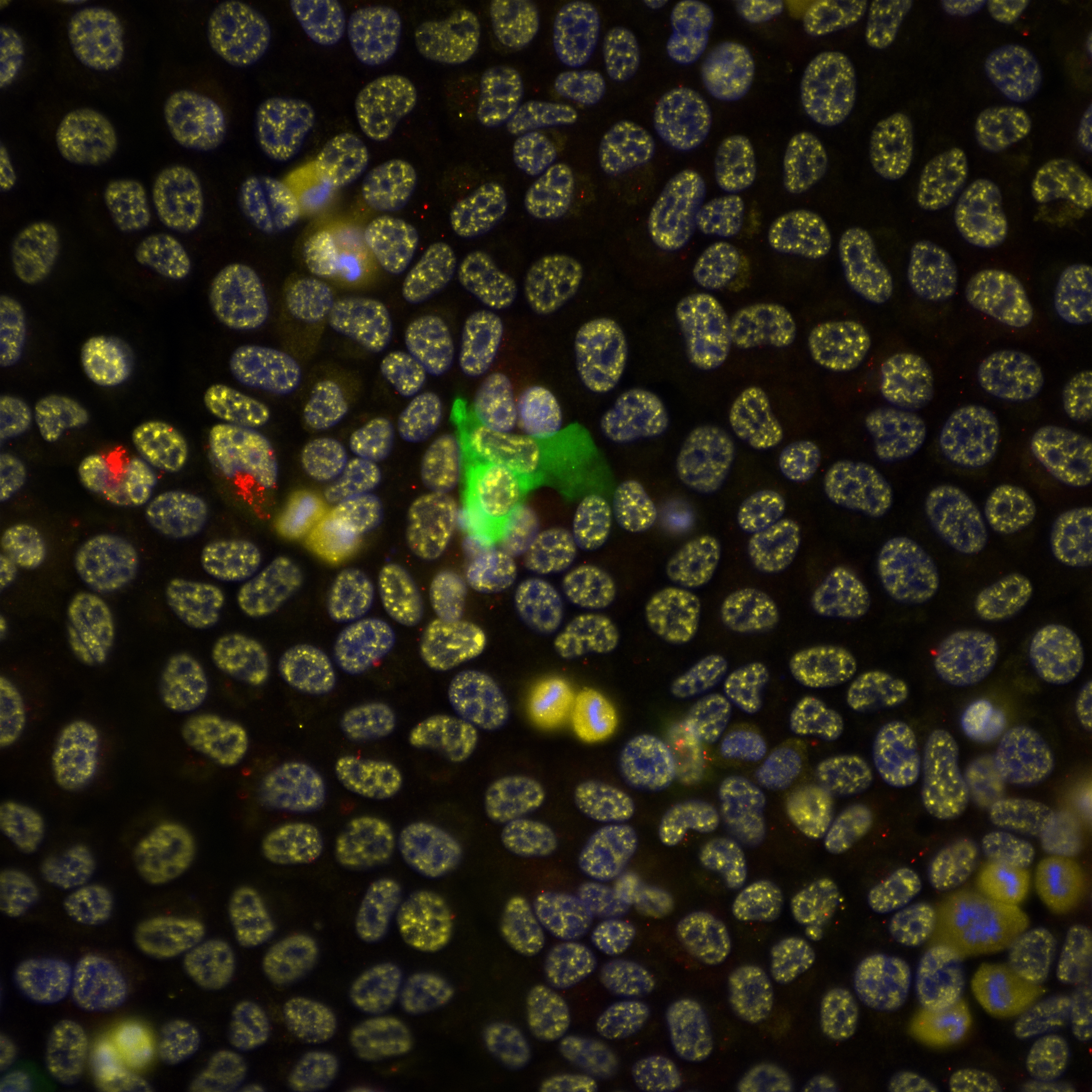

Supplement: Supplementary file 15 — Figure EV4 Source Data [file 44318_2025_421_MOESM15_ESM.zip › EV4/EV4D/Cell_9.tif]

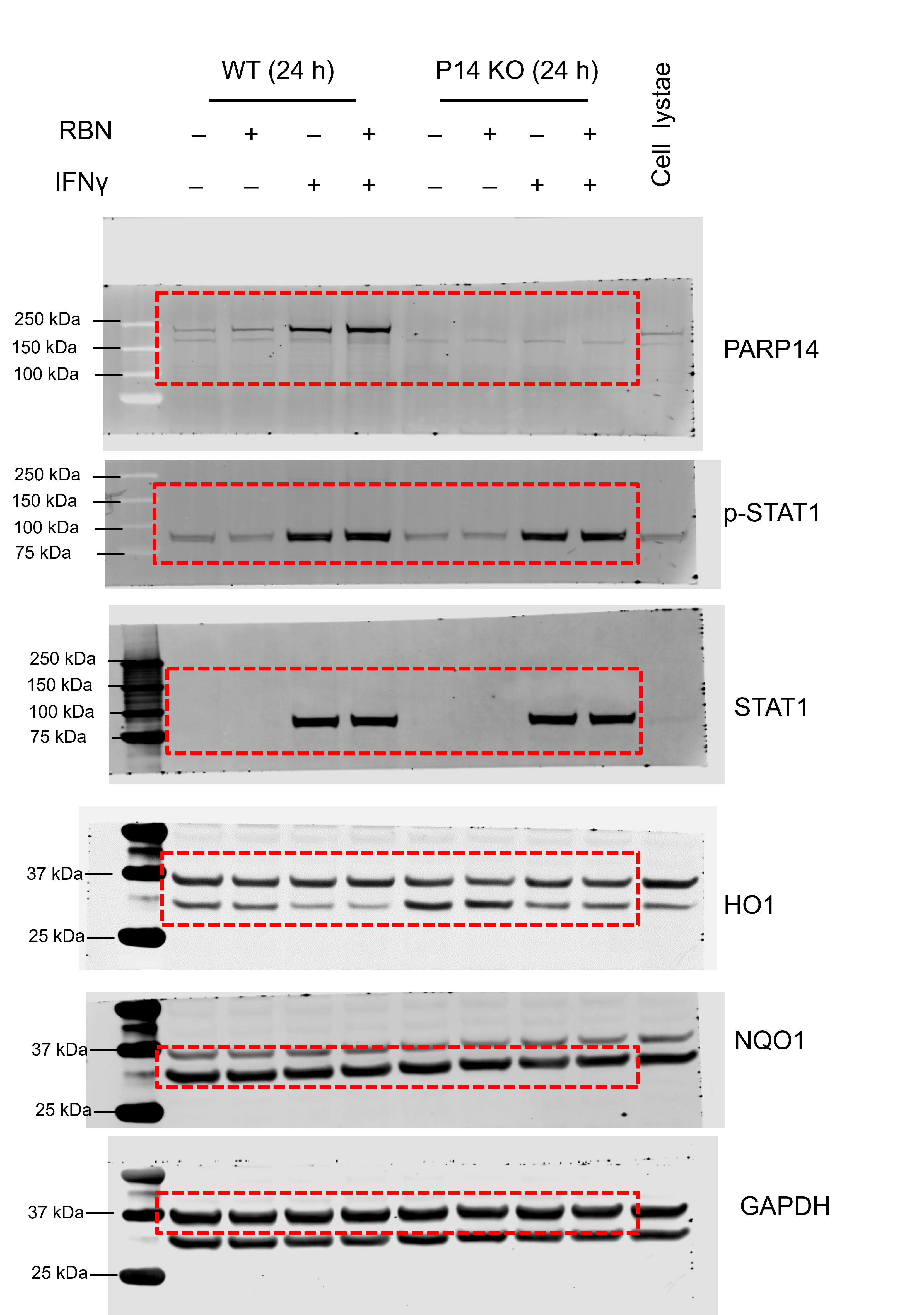

Supplement: Supplementary file 16 — Figure EV5 Source Data [file 44318_2025_421_MOESM16_ESM.zip › EV5/EV5A.tif]

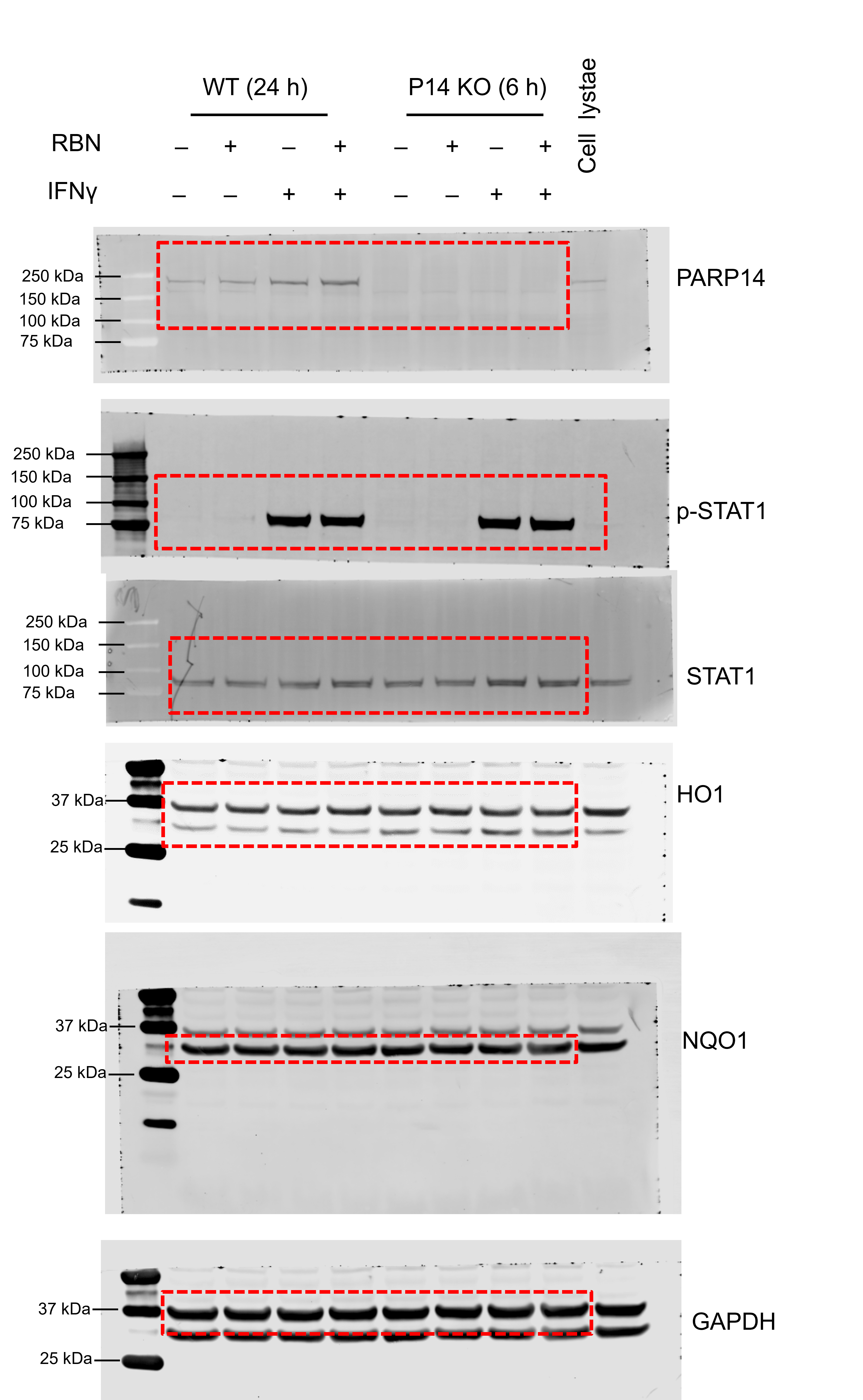

Supplement: Supplementary file 16 — Figure EV5 Source Data [file 44318_2025_421_MOESM16_ESM.zip › EV5/EV5B.tif]

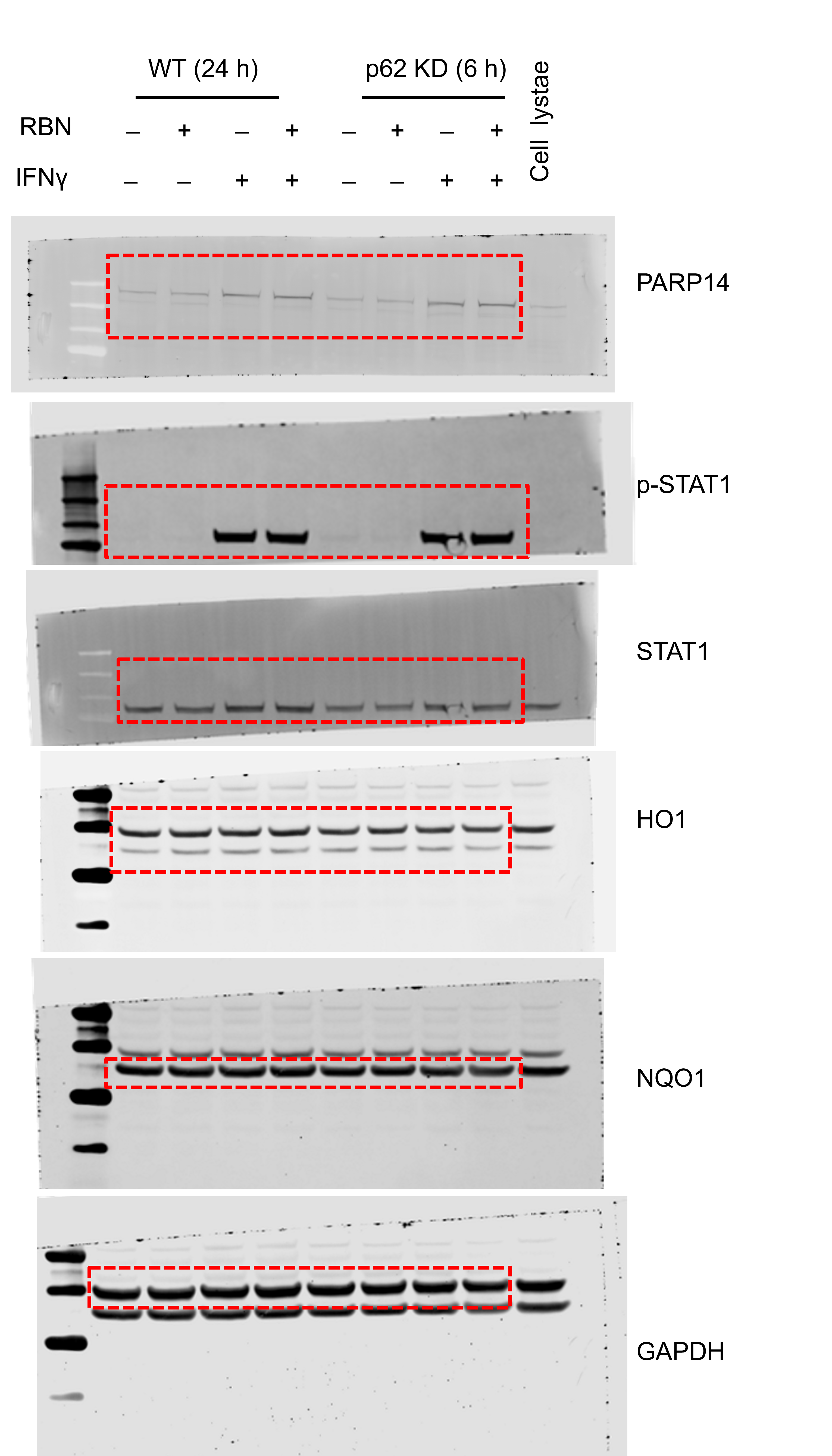

Supplement: Supplementary file 16 — Figure EV5 Source Data [file 44318_2025_421_MOESM16_ESM.zip › EV5/EV5C.tif]

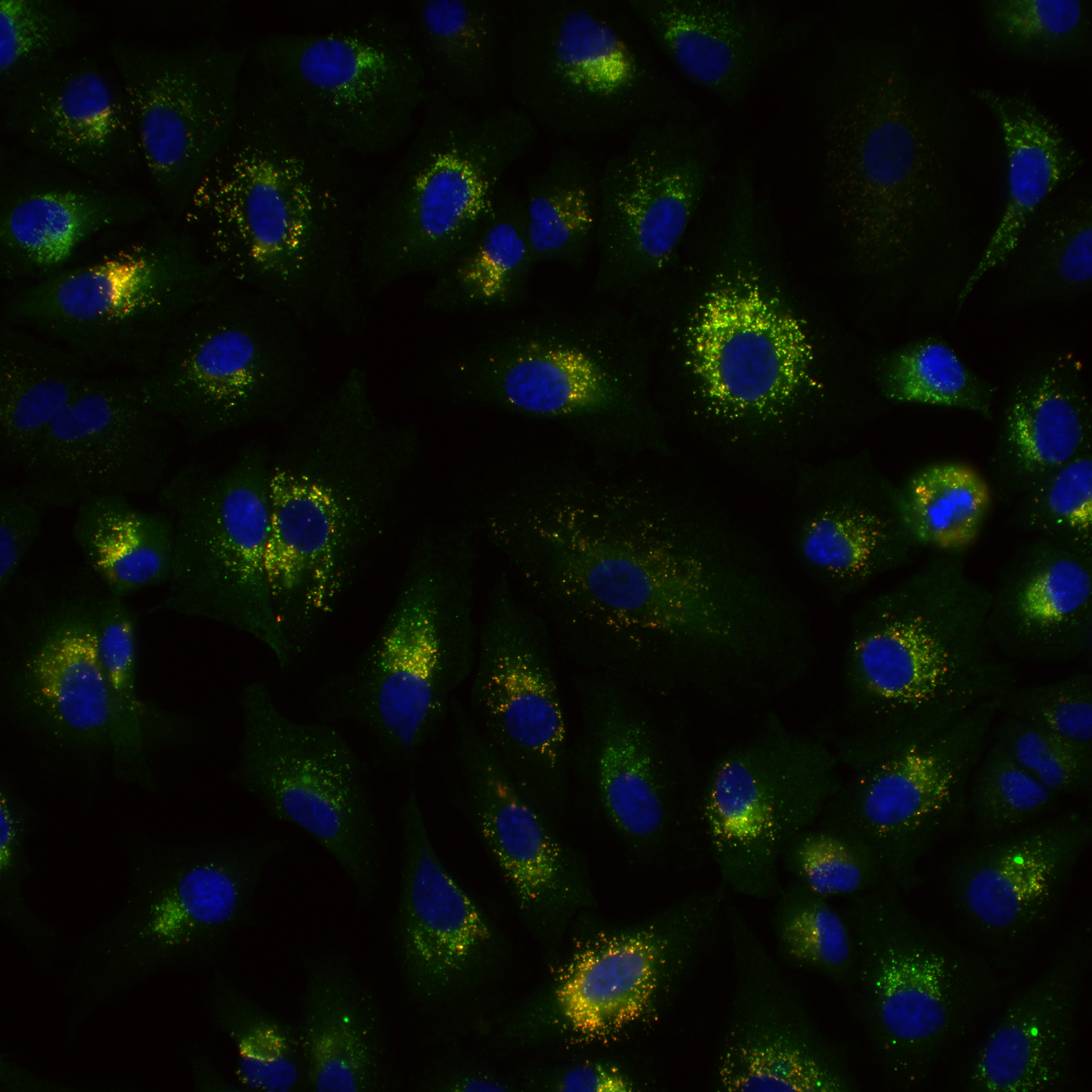

Supplement: Supplementary file 16 — Figure EV5 Source Data [file 44318_2025_421_MOESM16_ESM.zip › EV5/EV5E/Control_R2_LC3B_p62_A4 R3_overlay.tif]

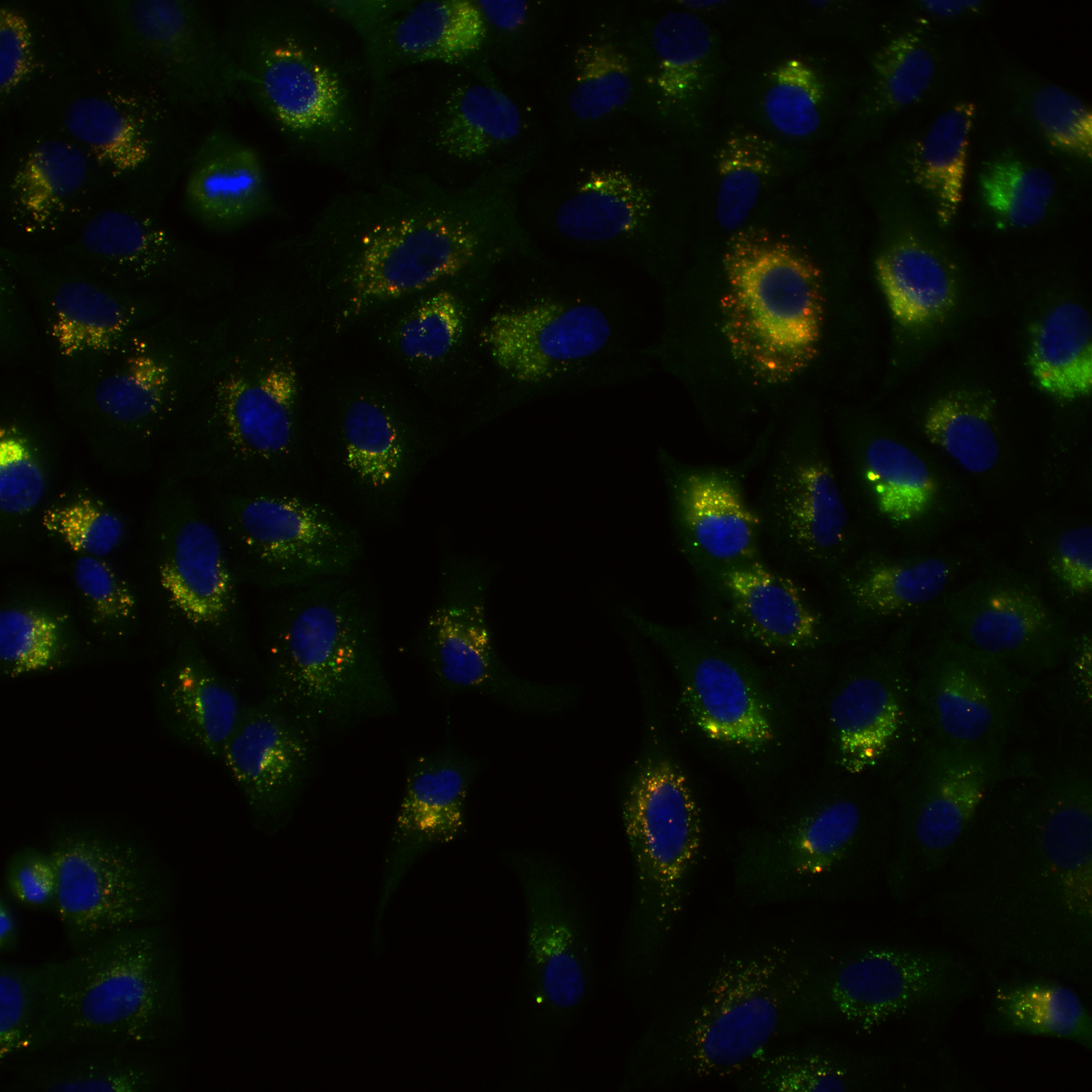

Supplement: Supplementary file 16 — Figure EV5 Source Data [file 44318_2025_421_MOESM16_ESM.zip › EV5/EV5E/IFNg_R1_LC3B_p62_C4 R3_overlay.tif]

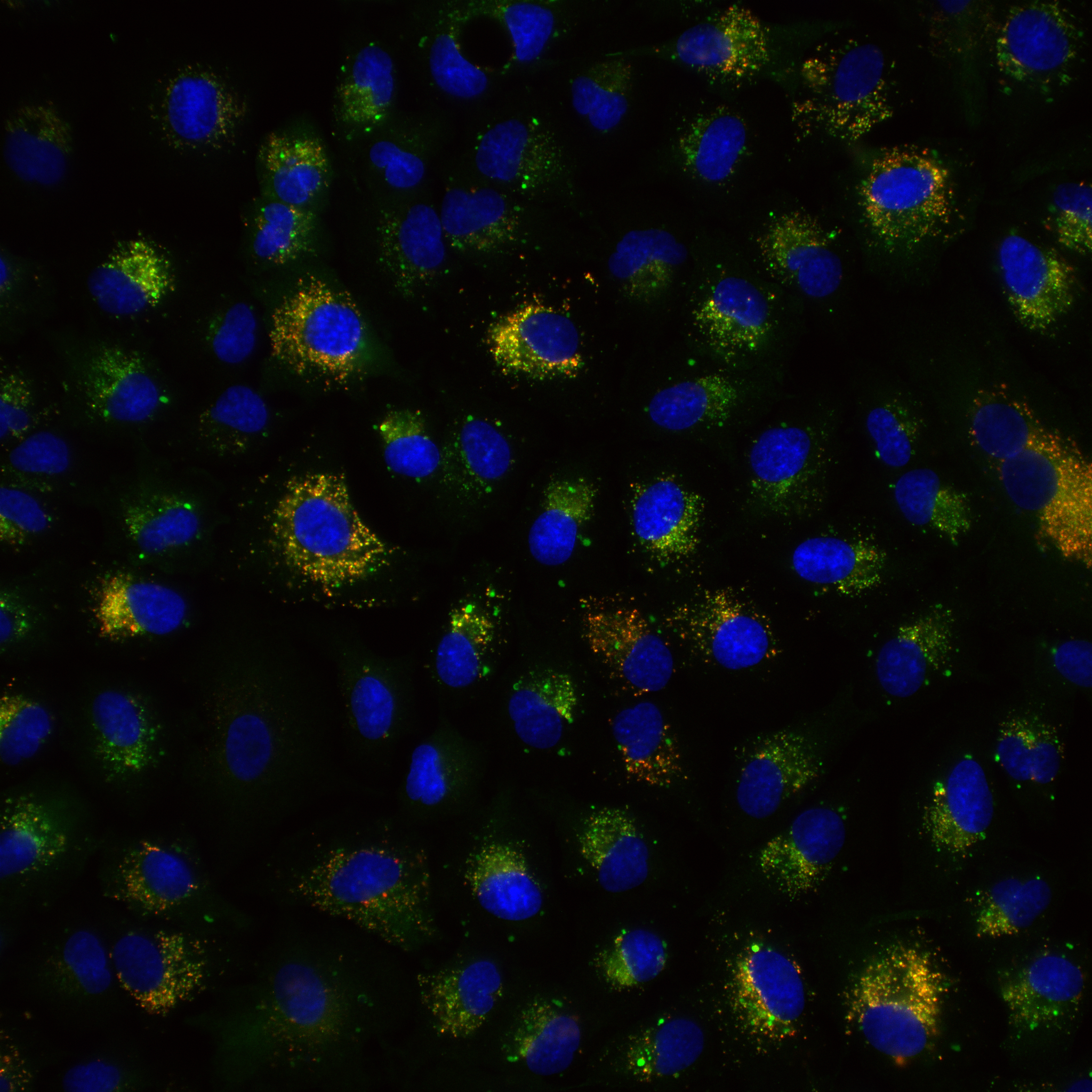

Supplement: Supplementary file 16 — Figure EV5 Source Data [file 44318_2025_421_MOESM16_ESM.zip › EV5/EV5E/Puro_R1_LC3B_p62_B4 R1_overlay.tif]

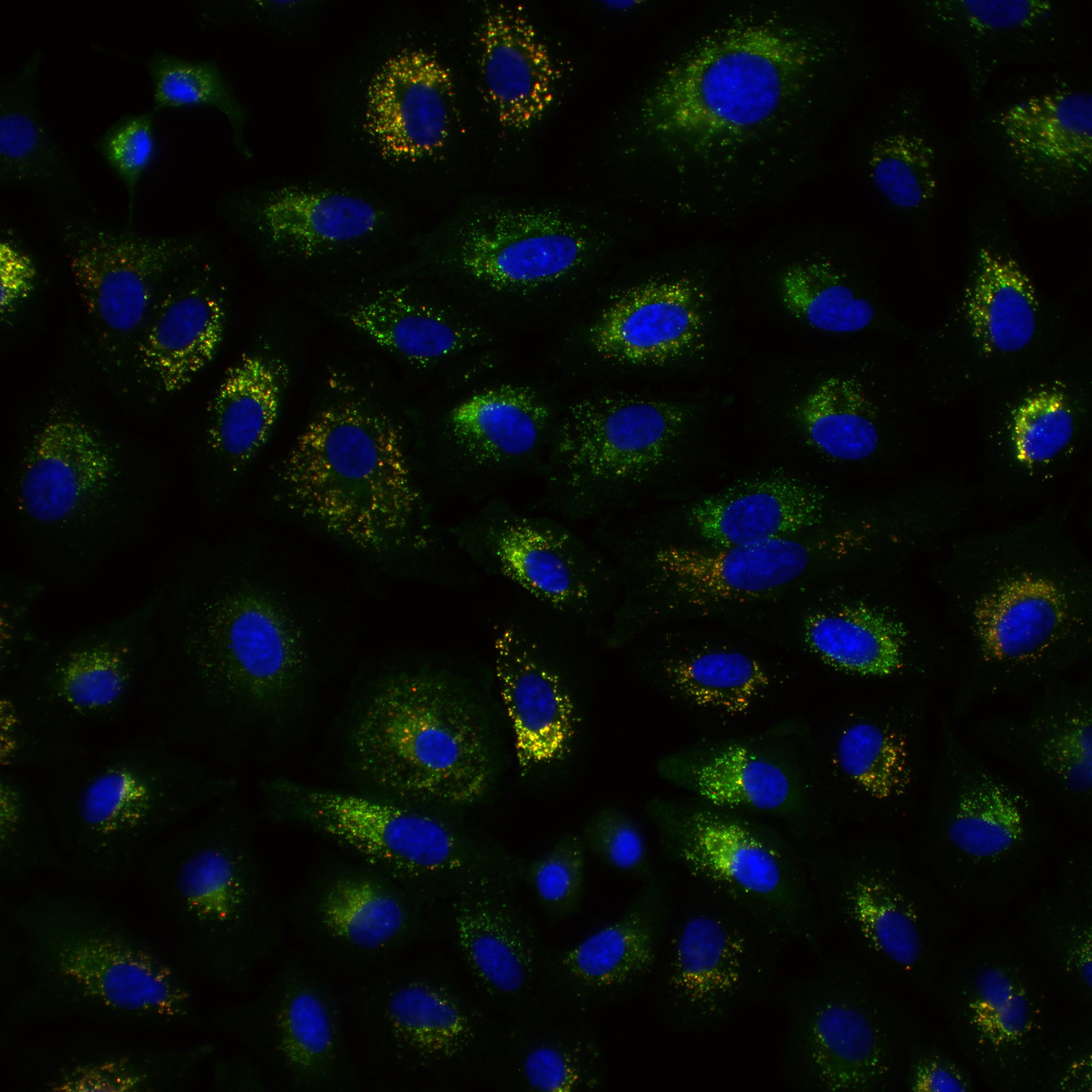

Supplement: Supplementary file 16 — Figure EV5 Source Data [file 44318_2025_421_MOESM16_ESM.zip › EV5/EV5E/Torin_R3_LC3B_p62_D6 R3_overlay.tif]

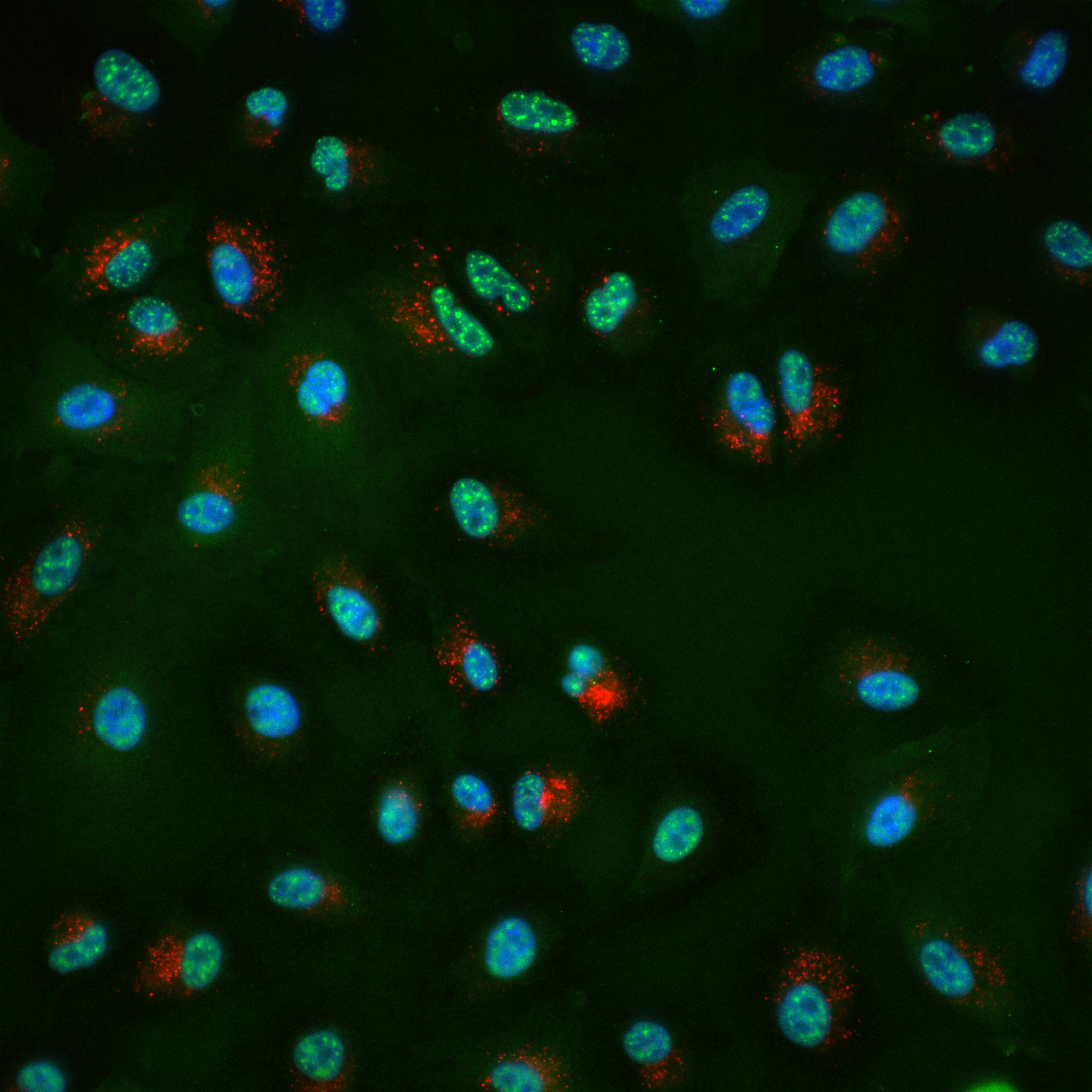

Supplement: Supplementary file 16 — Figure EV5 Source Data [file 44318_2025_421_MOESM16_ESM.zip › EV5/EV5F/Control_R1_LC3B_ADPr_A3 R2_overlay.tif]

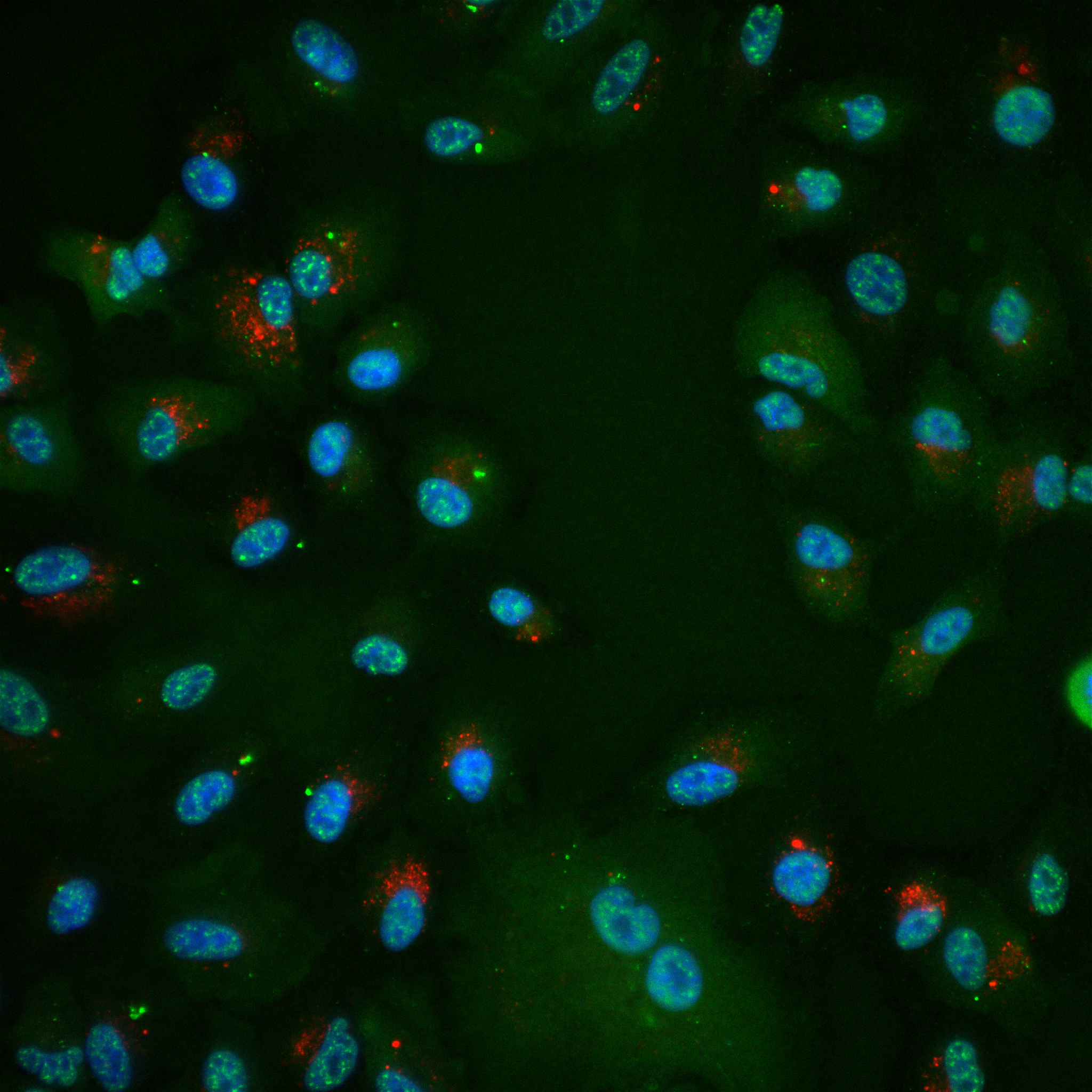

Supplement: Supplementary file 16 — Figure EV5 Source Data [file 44318_2025_421_MOESM16_ESM.zip › EV5/EV5F/IFNg_R1_LC3B_ADPr_C3 R1_overlay.tif]

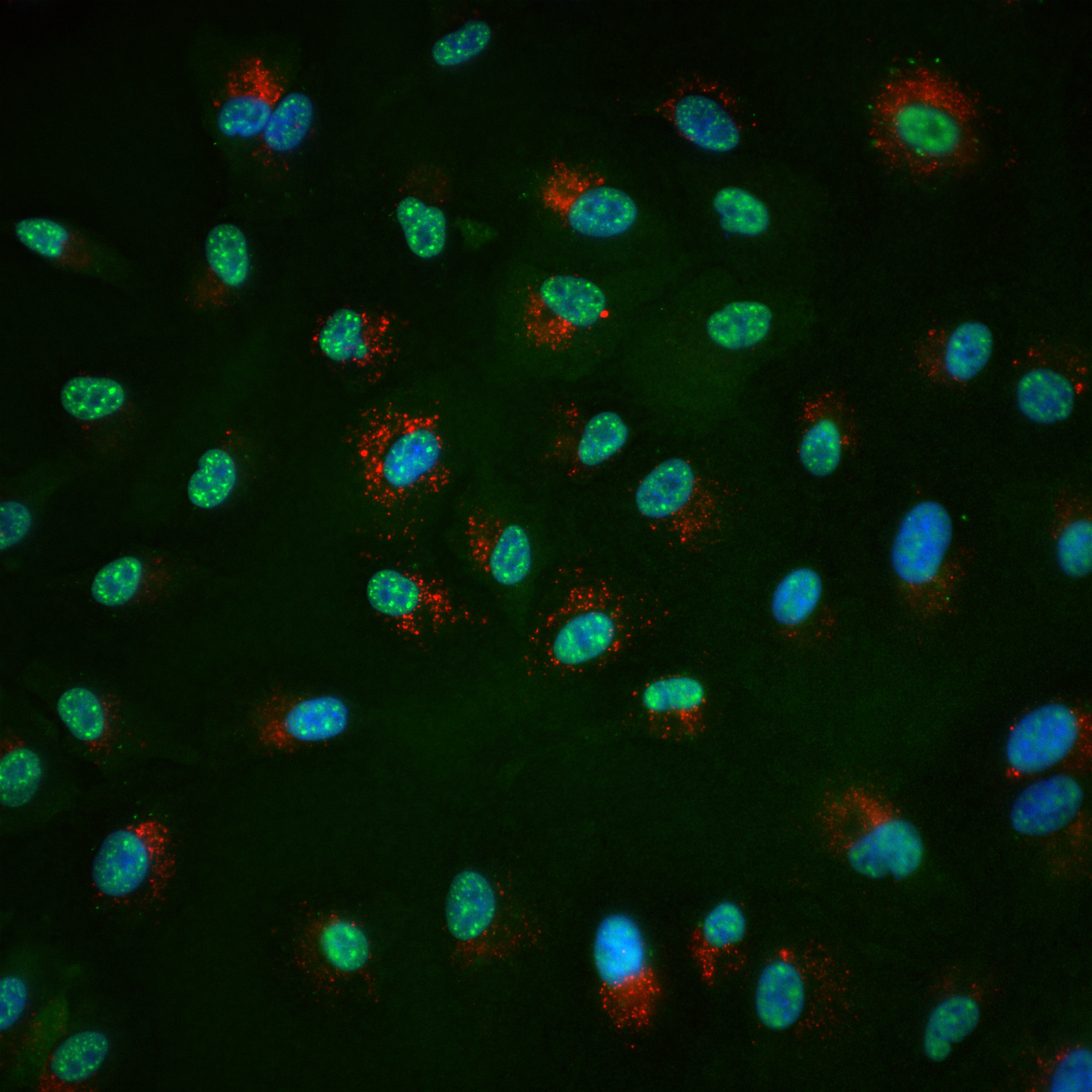

Supplement: Supplementary file 16 — Figure EV5 Source Data [file 44318_2025_421_MOESM16_ESM.zip › EV5/EV5F/Puro_R2_LC3B_ADPr_B3 R3_overlay.tif]

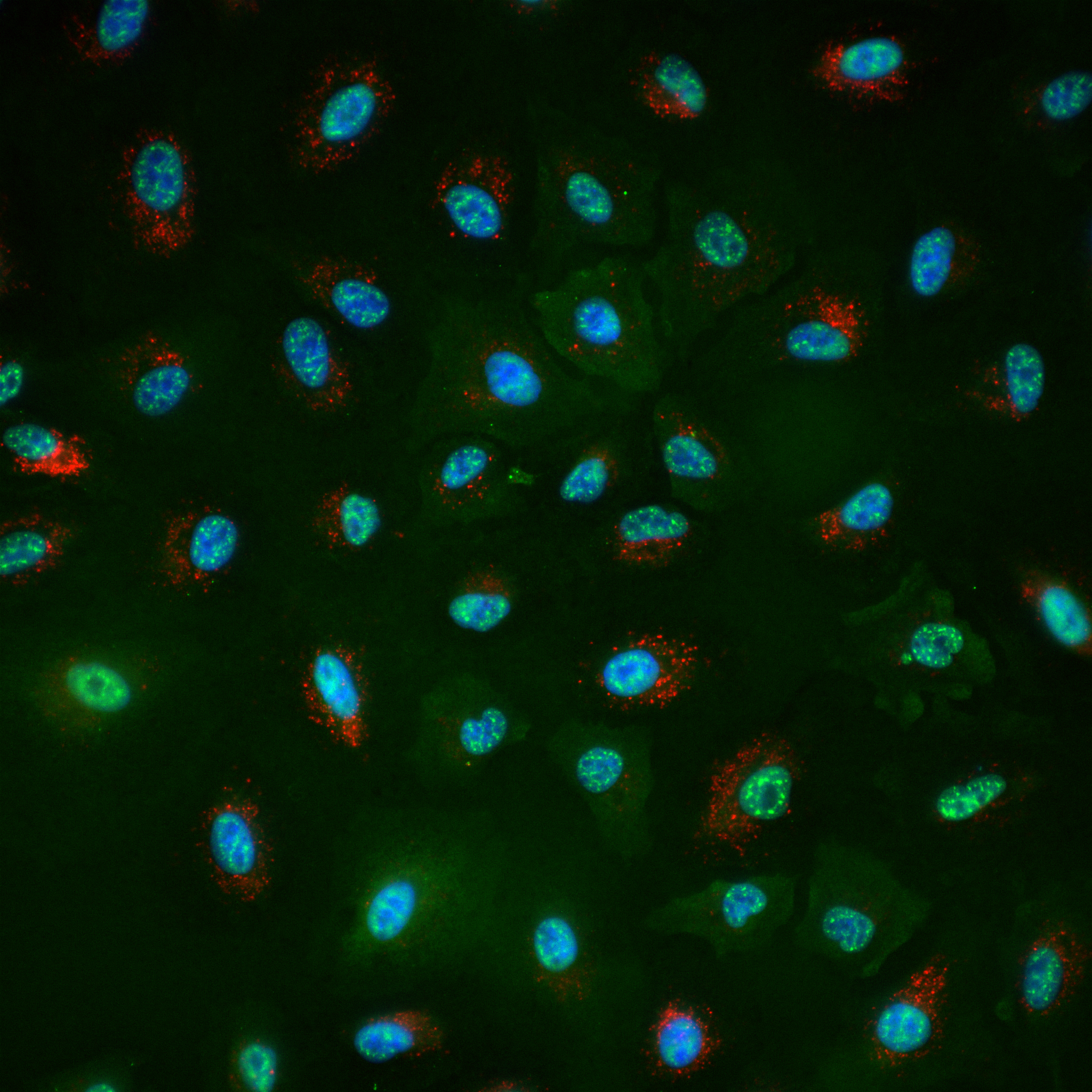

Supplement: Supplementary file 16 — Figure EV5 Source Data [file 44318_2025_421_MOESM16_ESM.zip › EV5/EV5F/Torin_R1_LC3B_ADPr_D5 R2_overlay.tif]

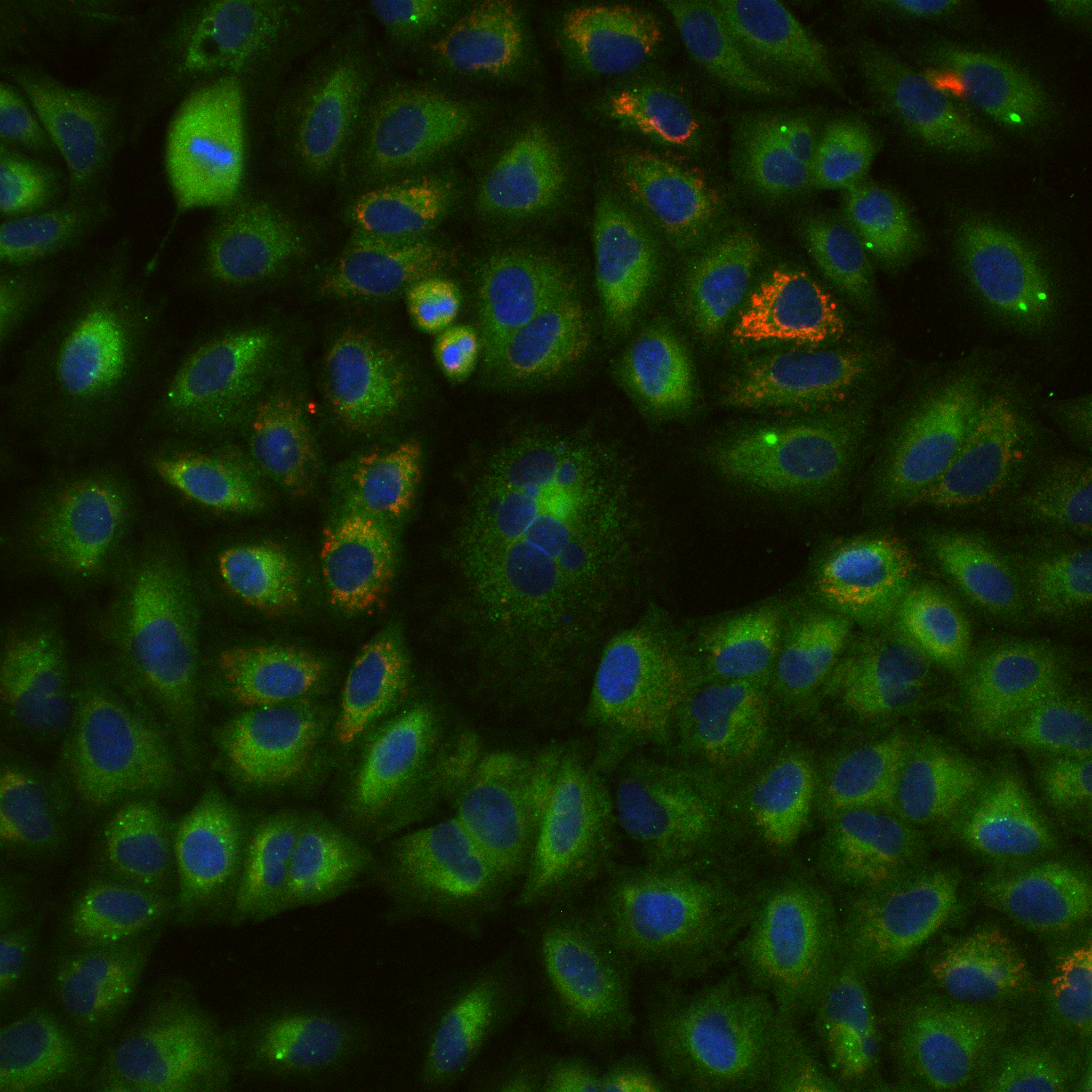

Supplement: Supplementary file 16 — Figure EV5 Source Data [file 44318_2025_421_MOESM16_ESM.zip › EV5/EV5H/Control.tif]

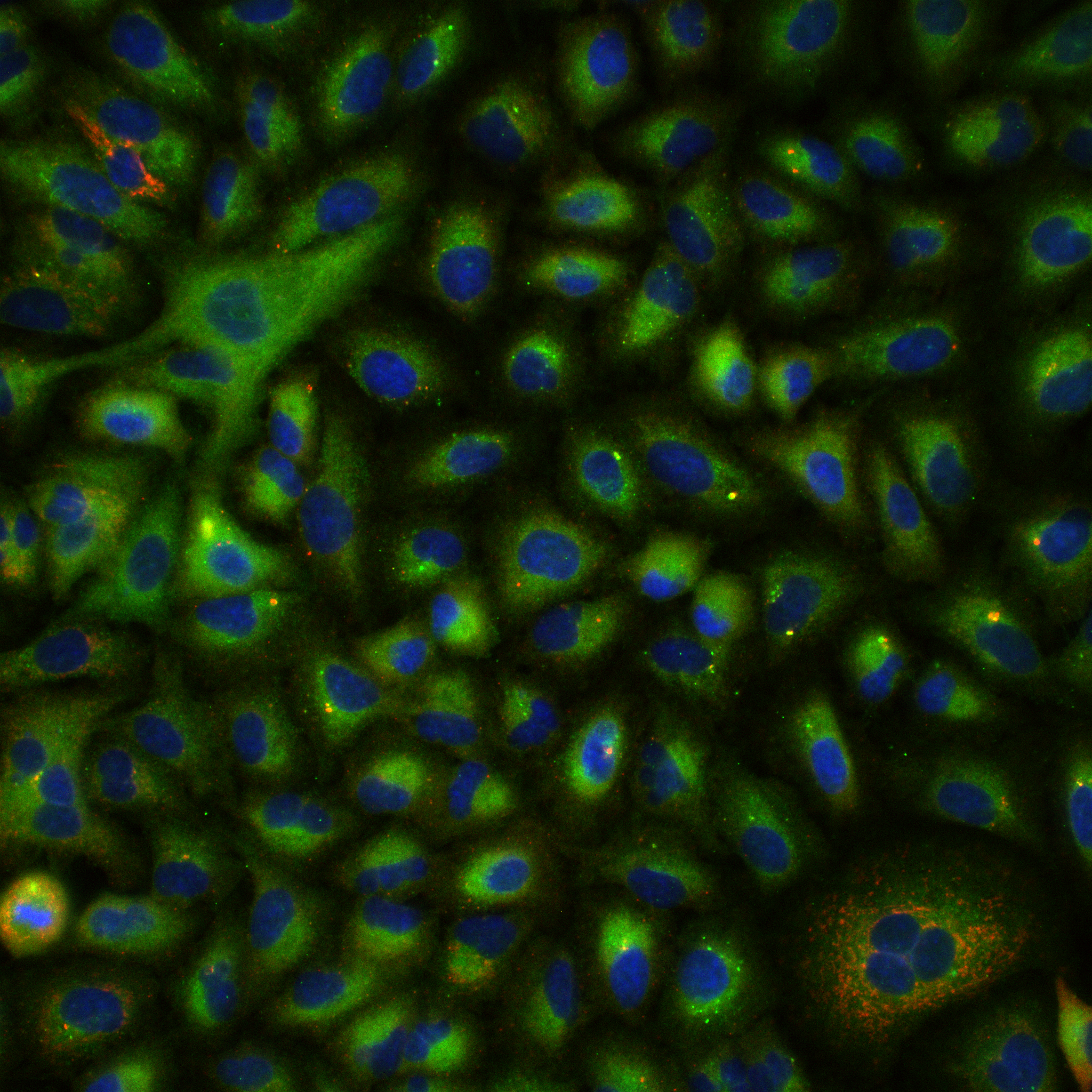

Supplement: Supplementary file 16 — Figure EV5 Source Data [file 44318_2025_421_MOESM16_ESM.zip › EV5/EV5H/IFN.tif]

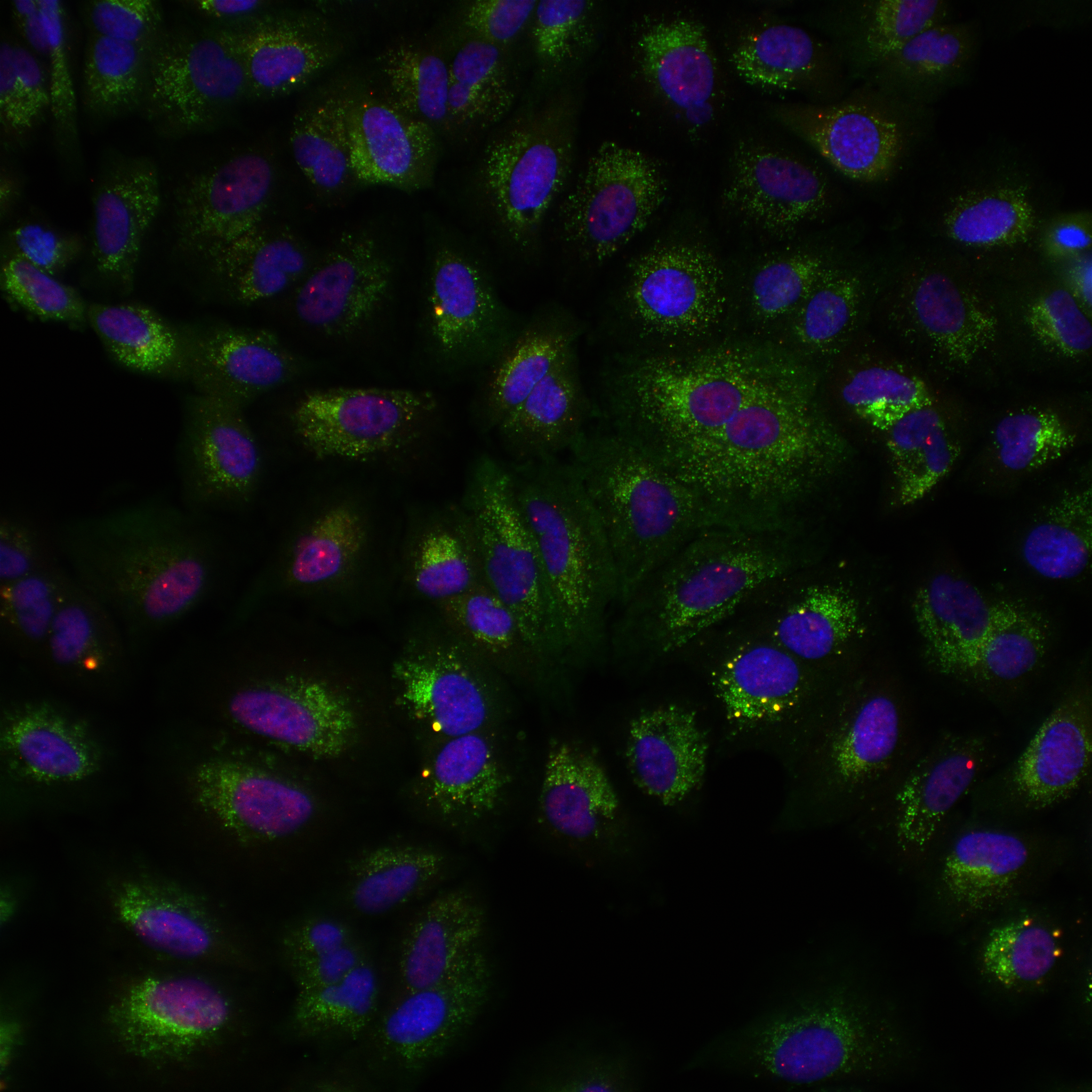

Supplement: Supplementary file 16 — Figure EV5 Source Data [file 44318_2025_421_MOESM16_ESM.zip › EV5/EV5I/Control_R2_UbFK2_p62_A5 R3_overlay.tif]

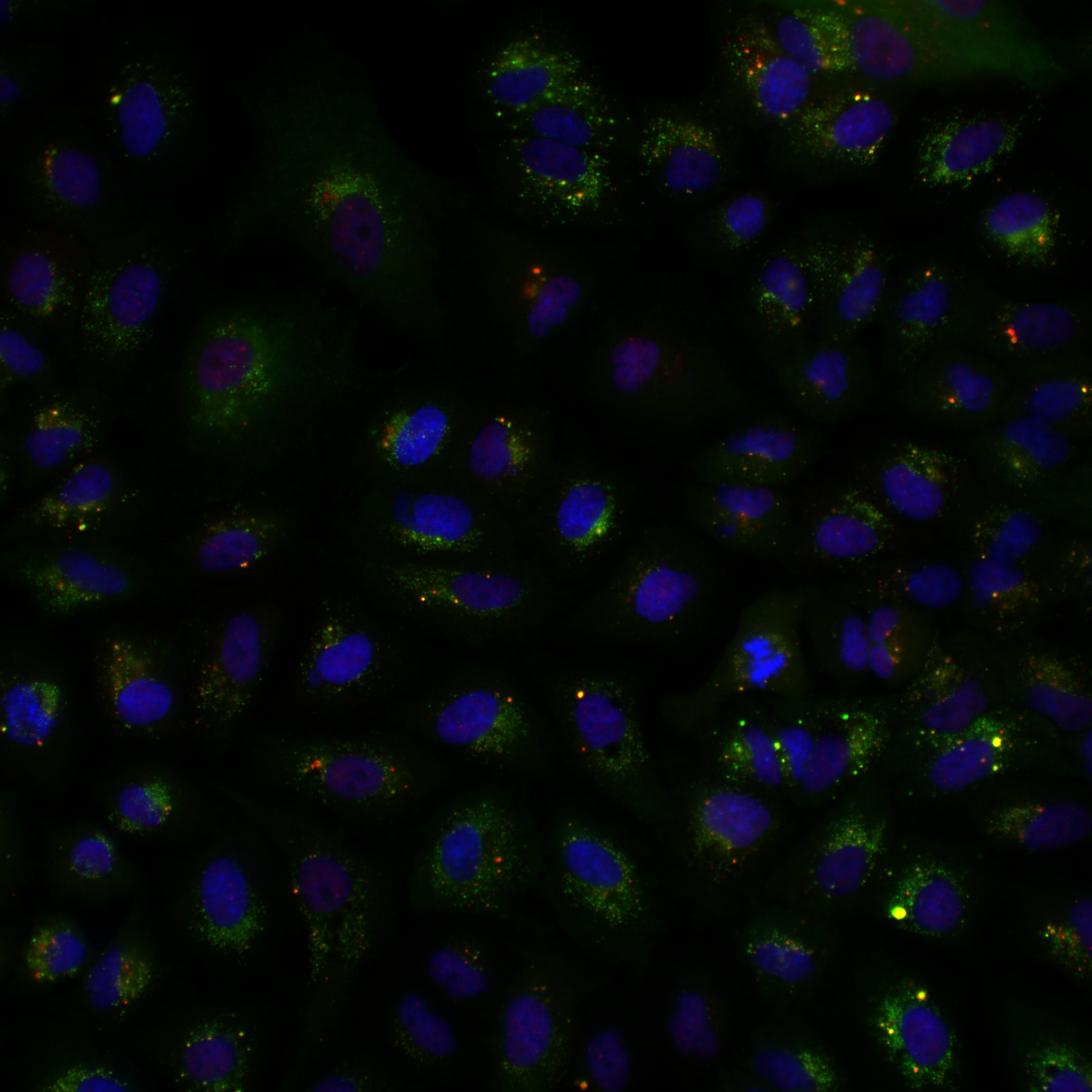

Supplement: Supplementary file 16 — Figure EV5 Source Data [file 44318_2025_421_MOESM16_ESM.zip › EV5/EV5I/IFNg_R1_UbFK2_p62_C5 R2_overlay.tif]

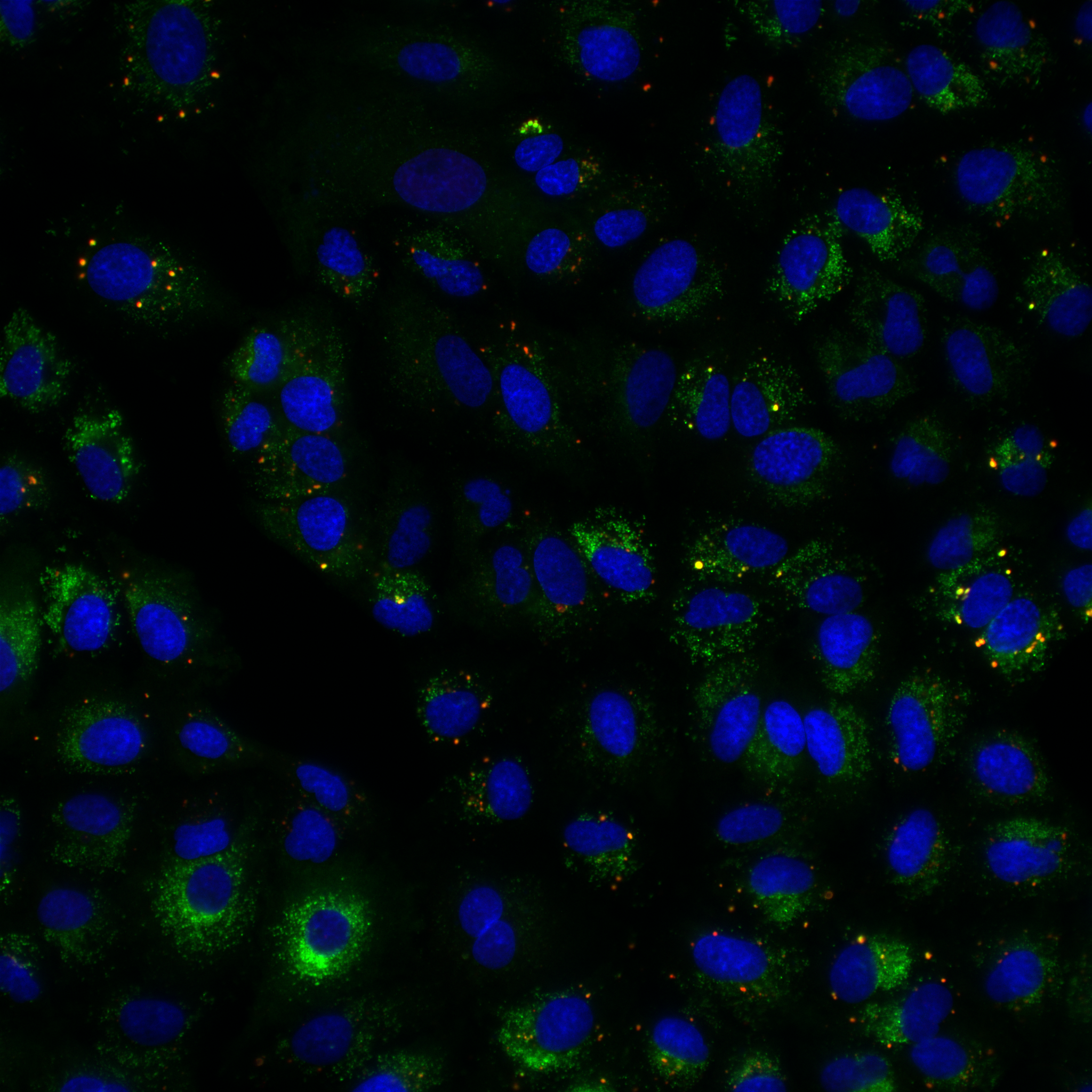

Supplement: Supplementary file 16 — Figure EV5 Source Data [file 44318_2025_421_MOESM16_ESM.zip › EV5/EV5I/Puro_R1_UbFK2_p62_B5 R3_overlay.tif]

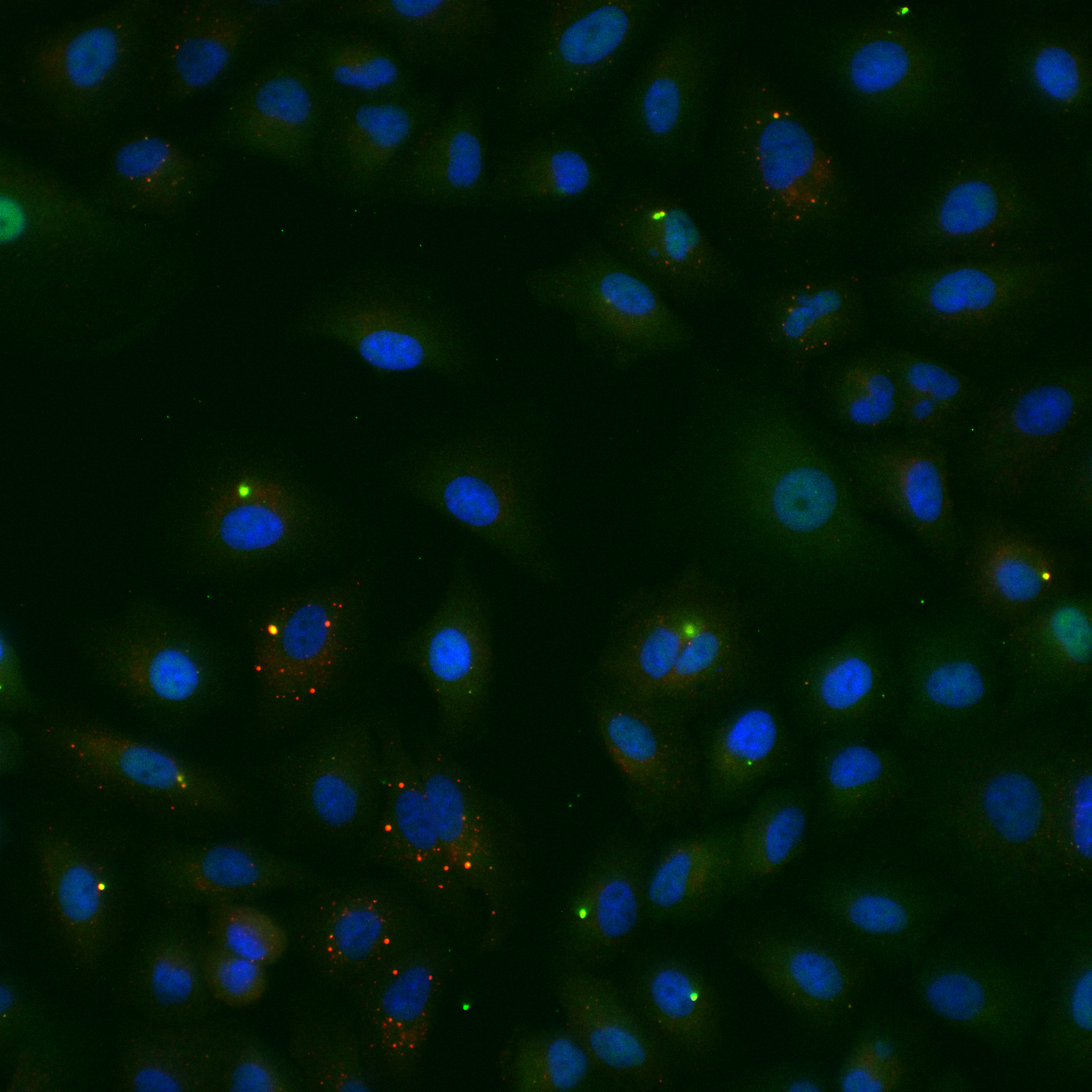

Supplement: Supplementary file 16 — Figure EV5 Source Data [file 44318_2025_421_MOESM16_ESM.zip › EV5/EV5J/Control_R1_UbK48_p62_A2 R3_overlay.tif]

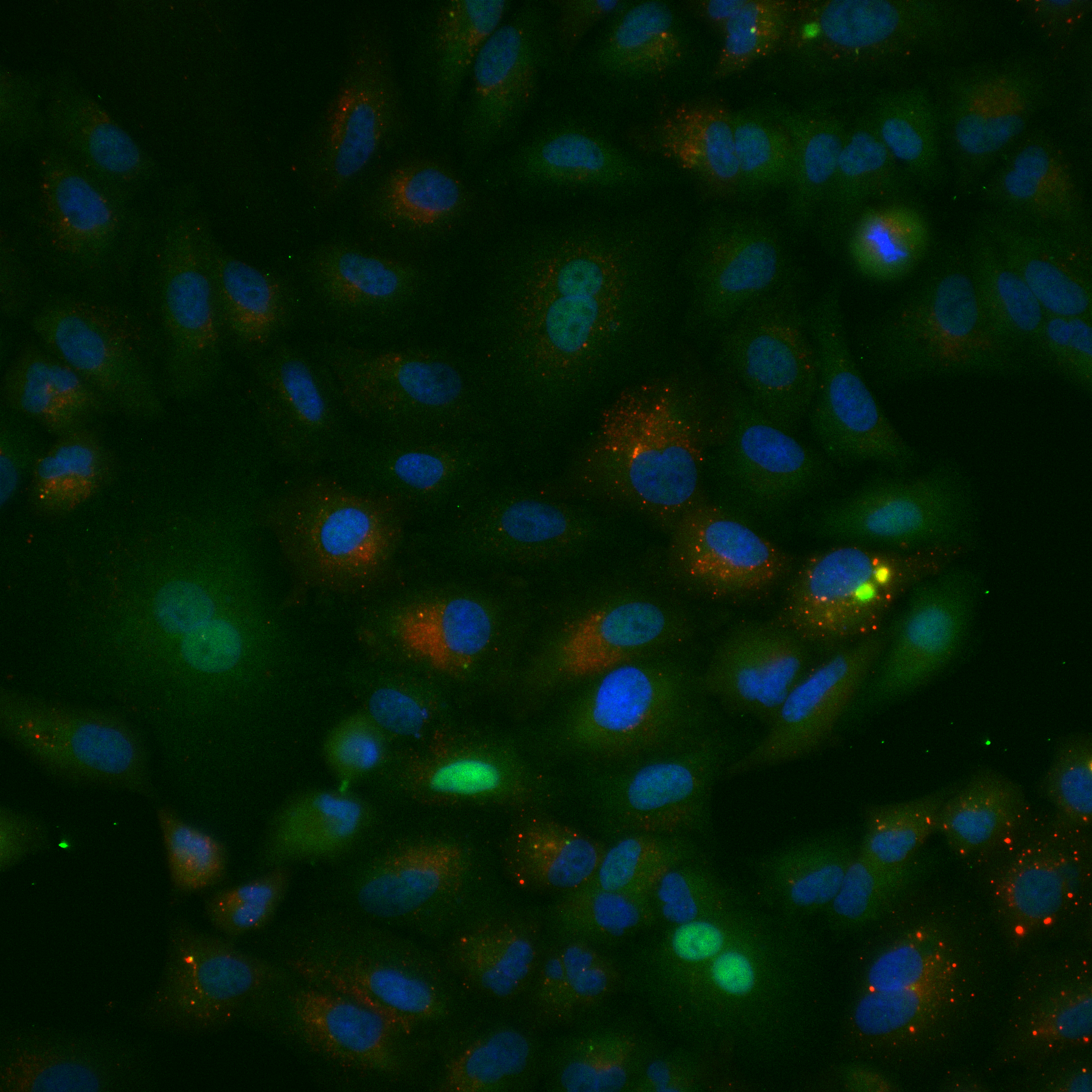

Supplement: Supplementary file 16 — Figure EV5 Source Data [file 44318_2025_421_MOESM16_ESM.zip › EV5/EV5J/IFNg_R1_UbK48_p62_C2 R3_overlay.tif]

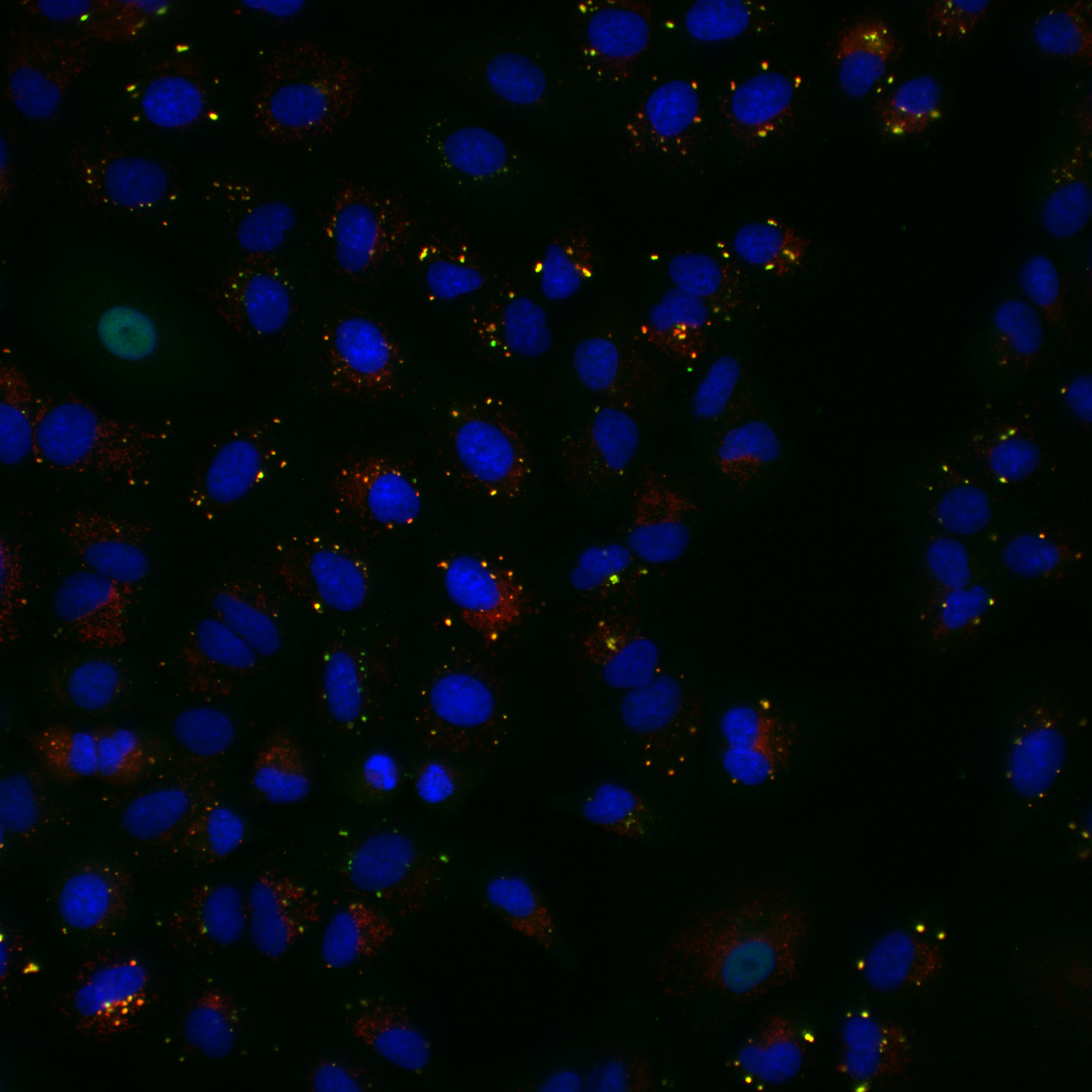

Supplement: Supplementary file 16 — Figure EV5 Source Data [file 44318_2025_421_MOESM16_ESM.zip › EV5/EV5J/Puro_R1_UbK48_p62_B2 R4_overlay.tif]

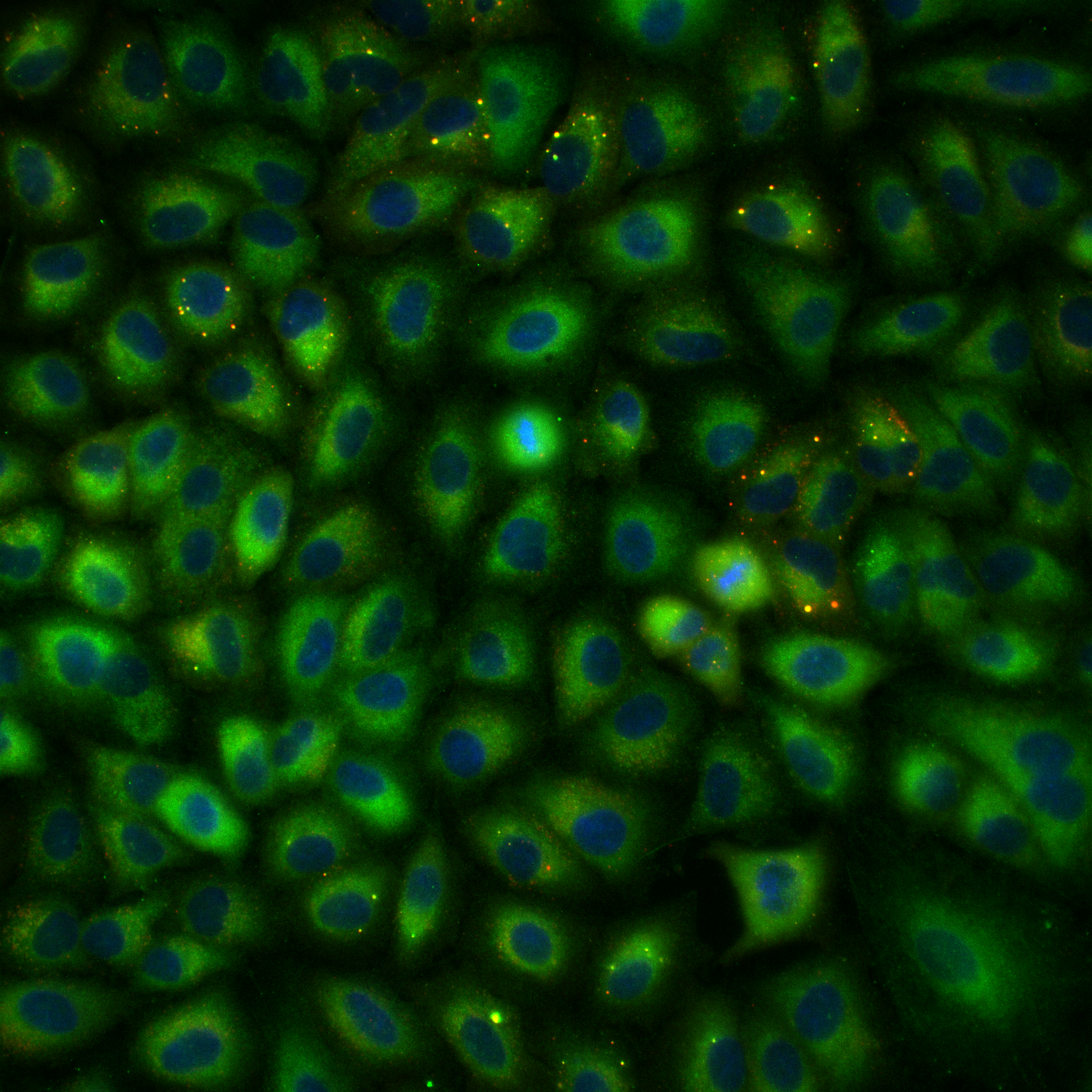

Supplement: Supplementary file 16 — Figure EV5 Source Data [file 44318_2025_421_MOESM16_ESM.zip › EV5/EV5K/Control_R1_UbK63_p62_A4 R5_overlay.tif]

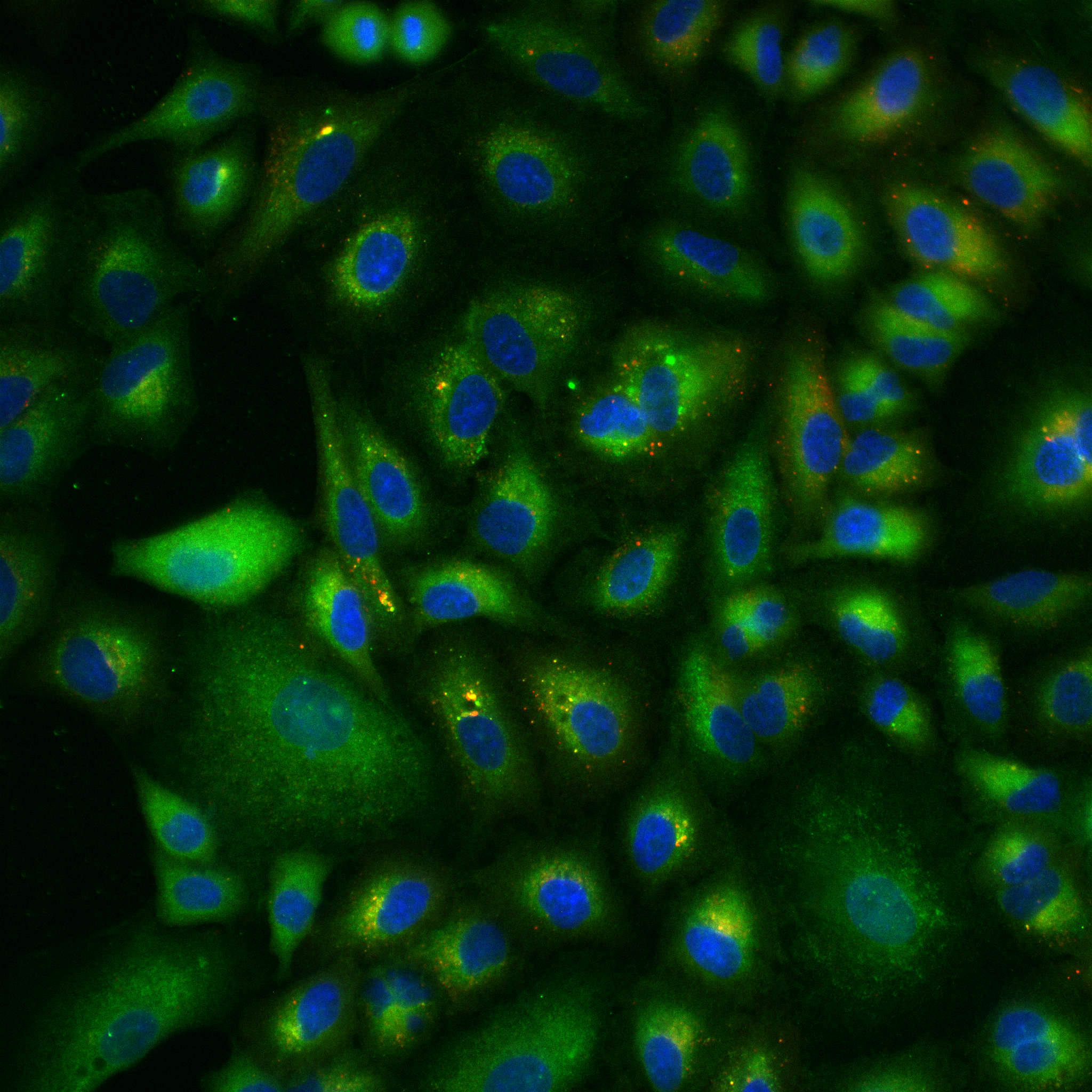

Supplement: Supplementary file 16 — Figure EV5 Source Data [file 44318_2025_421_MOESM16_ESM.zip › EV5/EV5K/IFNg_R3_UbK63_p62_C4 R8_overlay.tif]

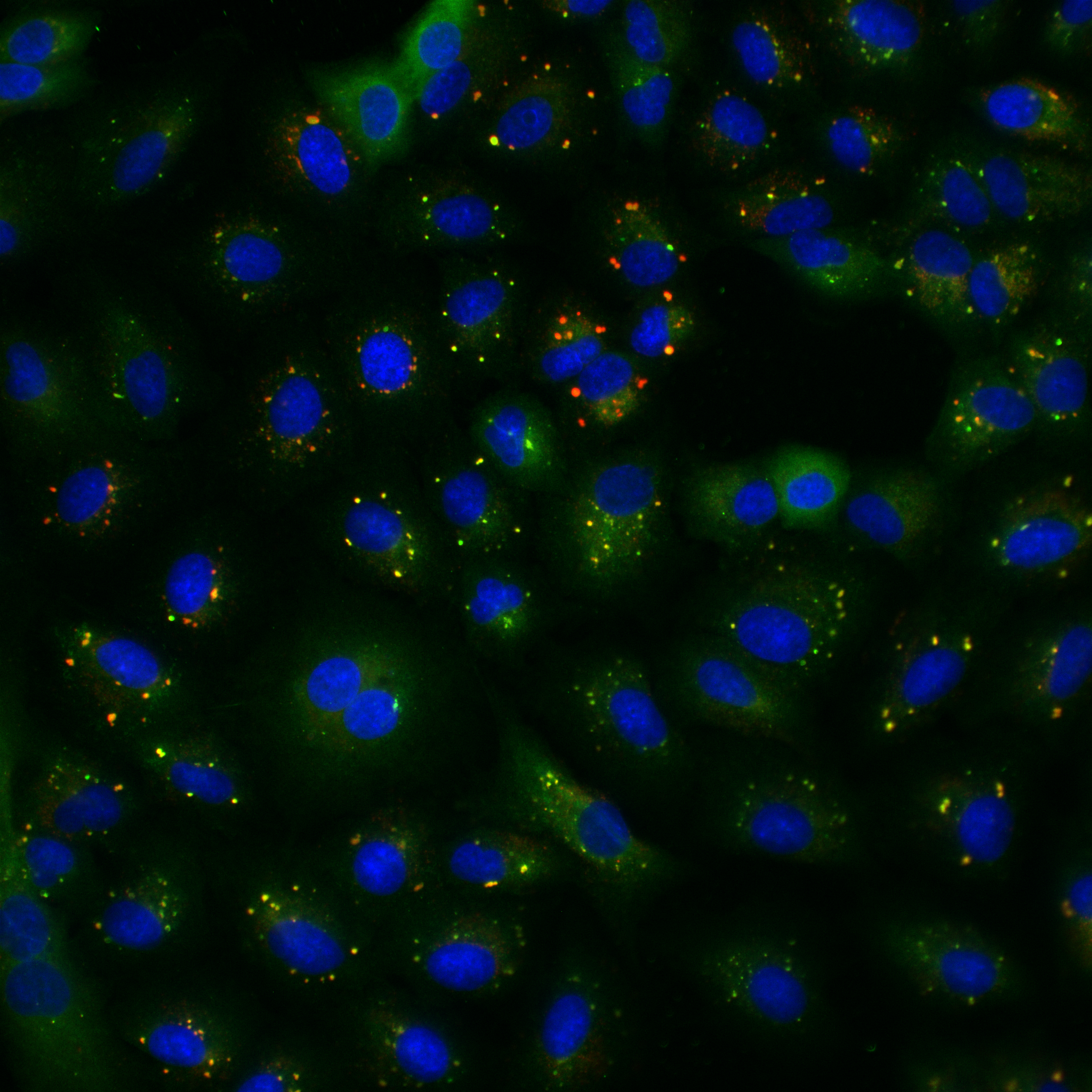

Supplement: Supplementary file 16 — Figure EV5 Source Data [file 44318_2025_421_MOESM16_ESM.zip › EV5/EV5K/Puro_R1_UbK63_p62_B4 R4_overlay.tif]

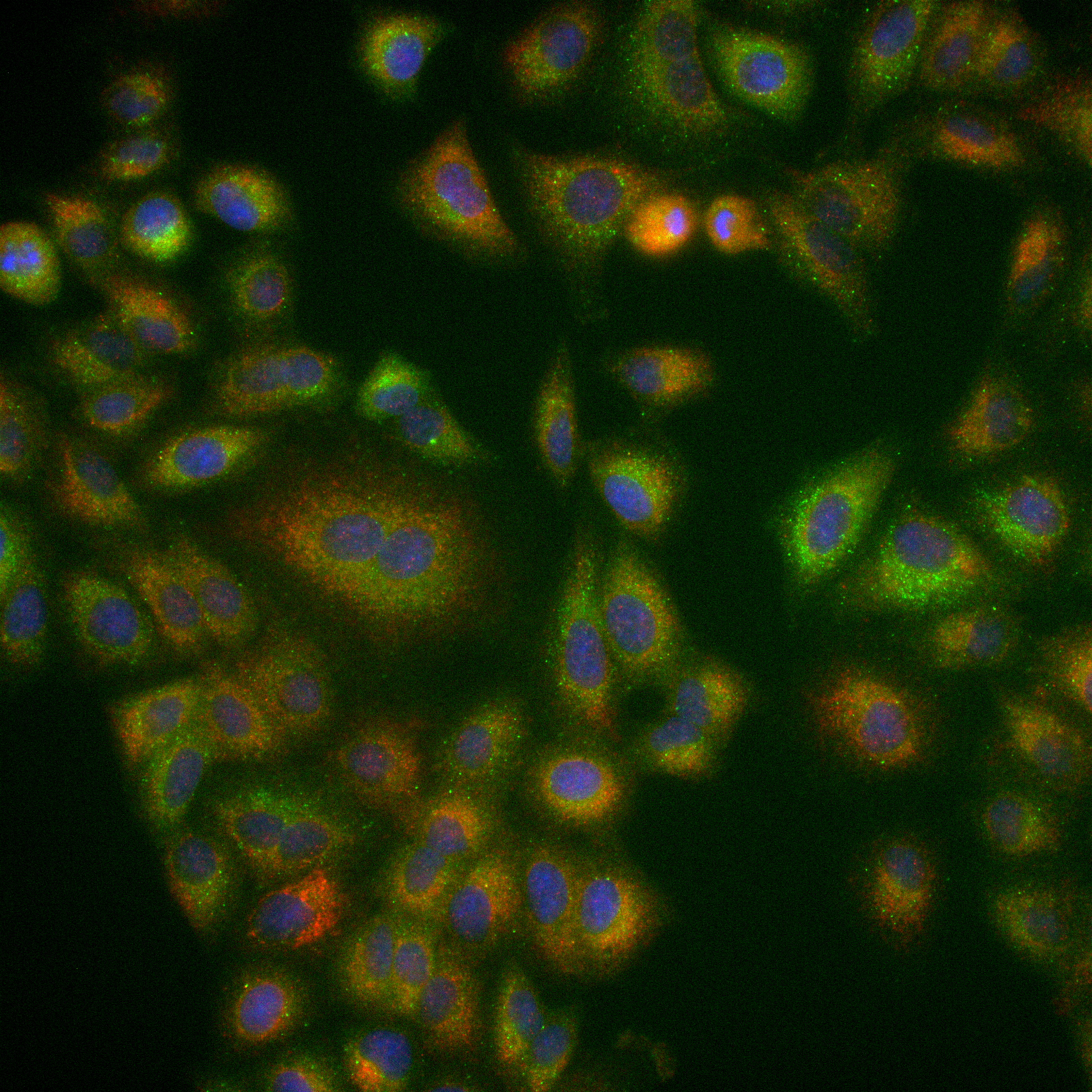

Supplement: Supplementary file 16 — Figure EV5 Source Data [file 44318_2025_421_MOESM16_ESM.zip › EV5/EV5M/Control.tif]

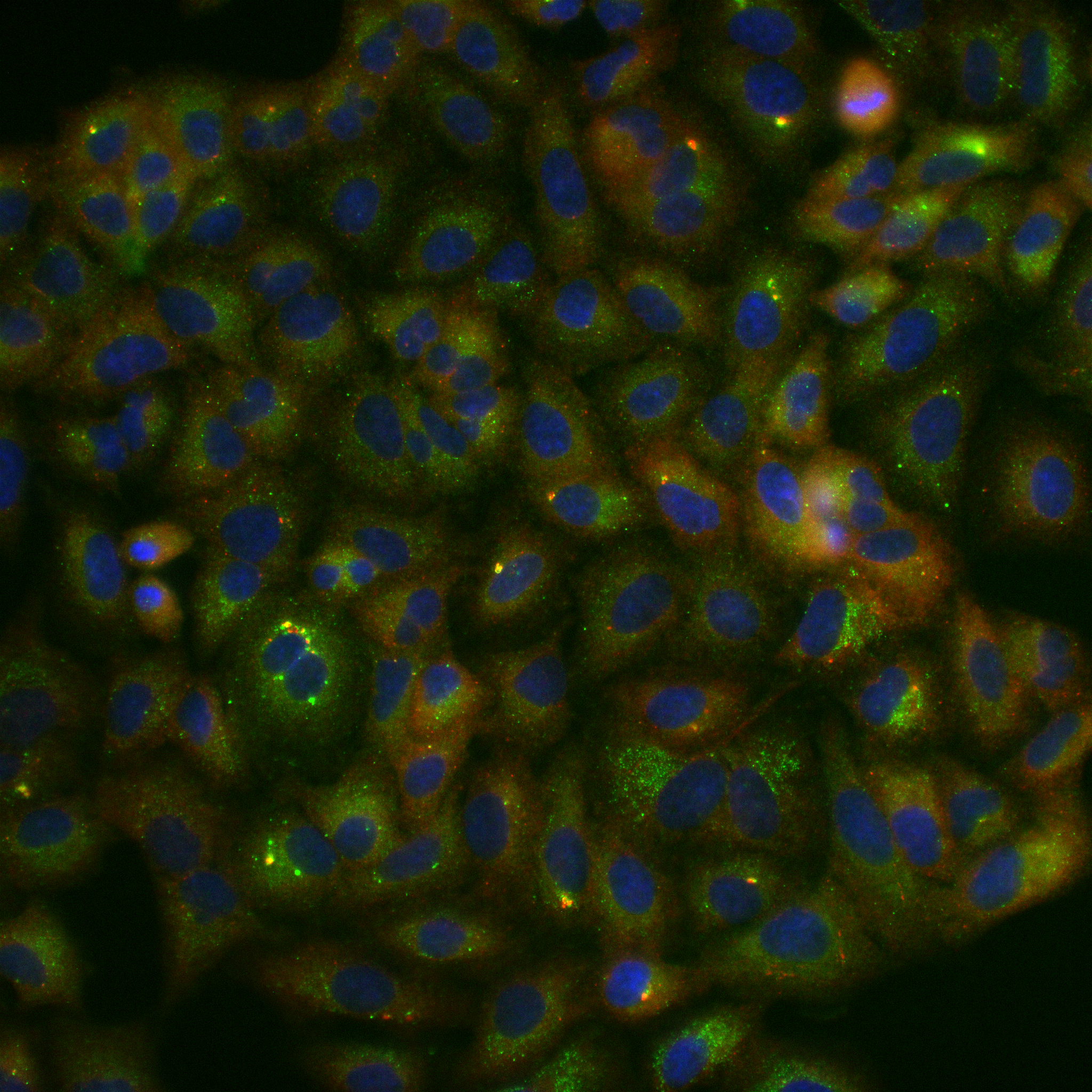

Supplement: Supplementary file 16 — Figure EV5 Source Data [file 44318_2025_421_MOESM16_ESM.zip › EV5/EV5M/IFNγ.tif]

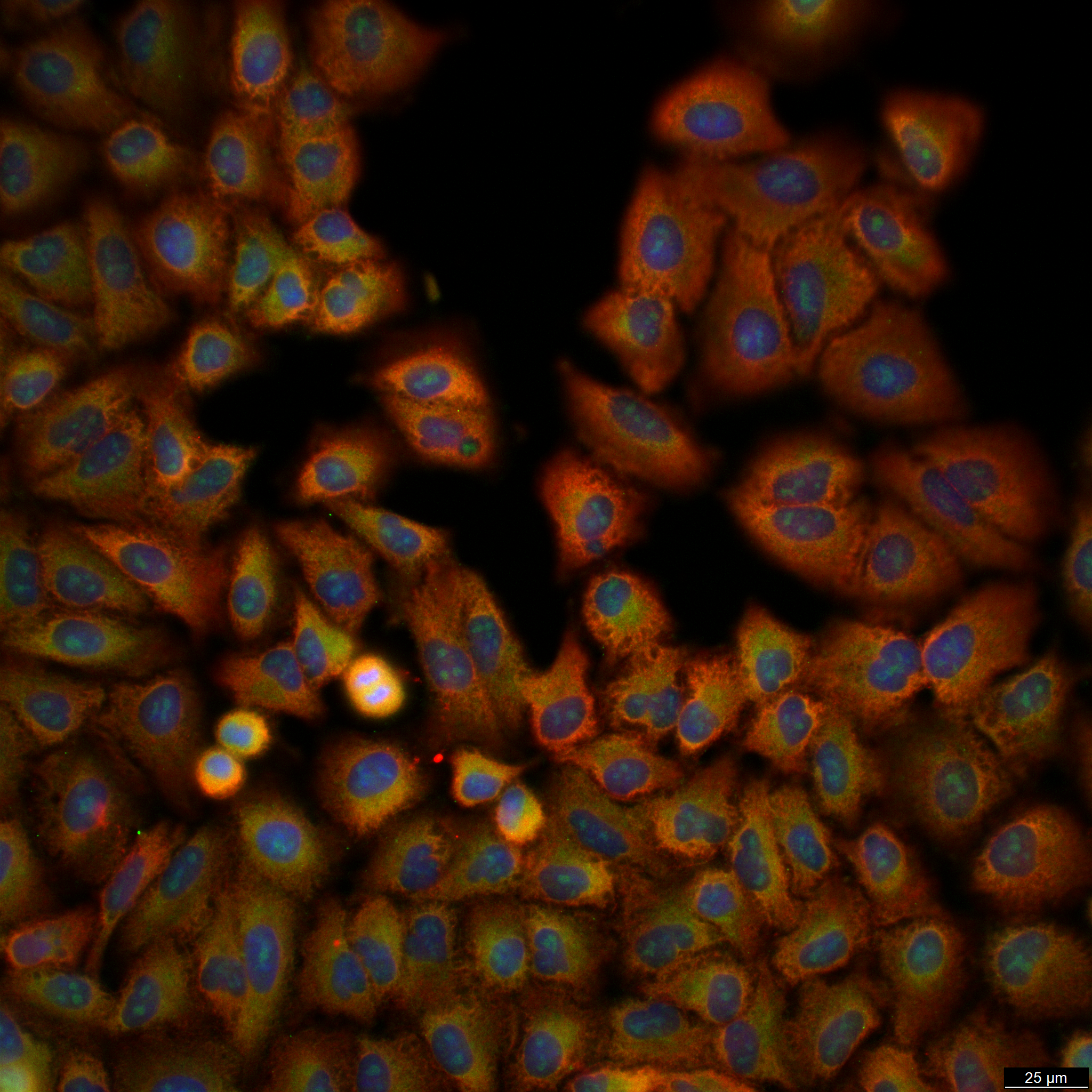

Supplement: Supplementary file 16 — Figure EV5 Source Data [file 44318_2025_421_MOESM16_ESM.zip › EV5/EV5N/Control.tif]

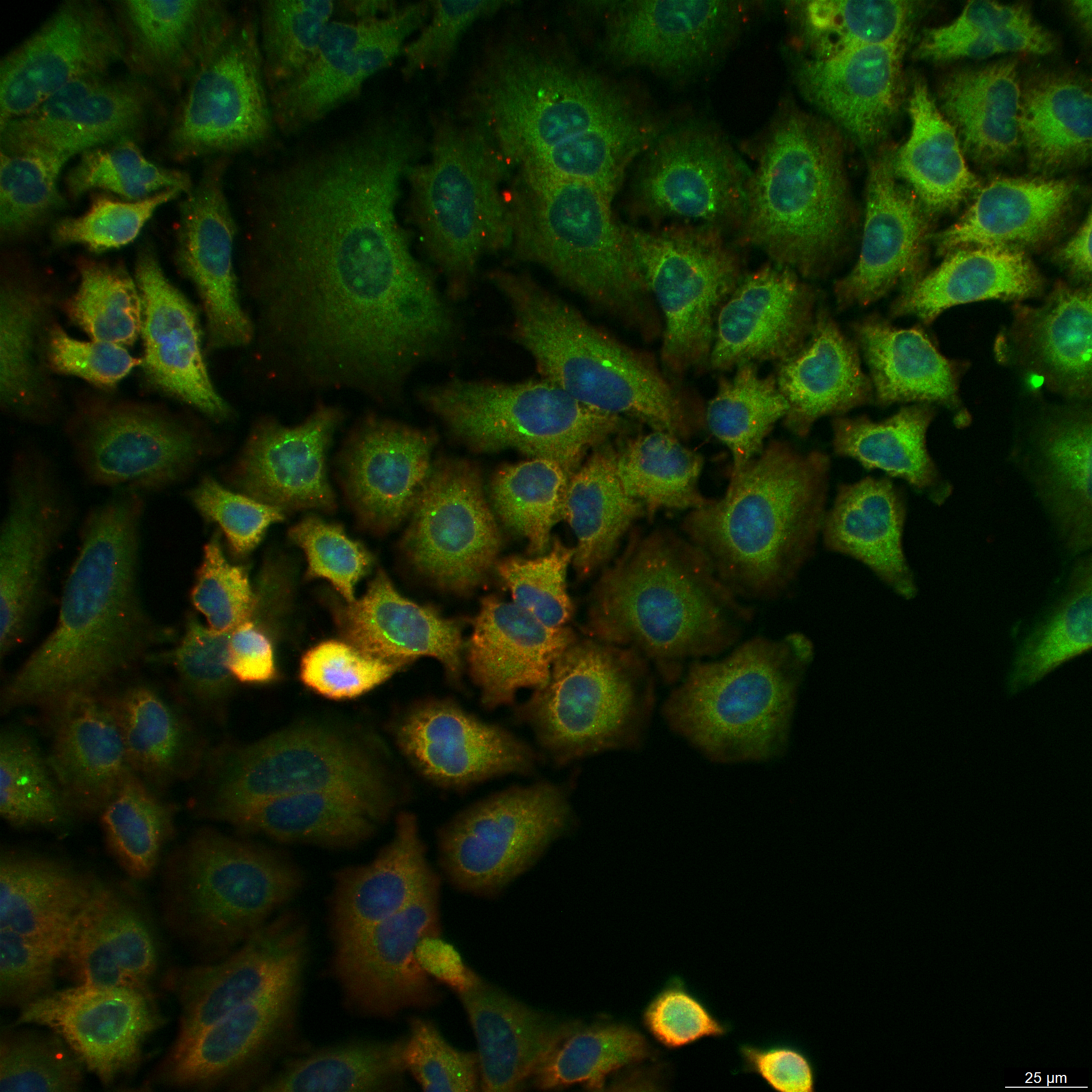

Supplement: Supplementary file 16 — Figure EV5 Source Data [file 44318_2025_421_MOESM16_ESM.zip › EV5/EV5N/lFNγ+Torin 1.tif]

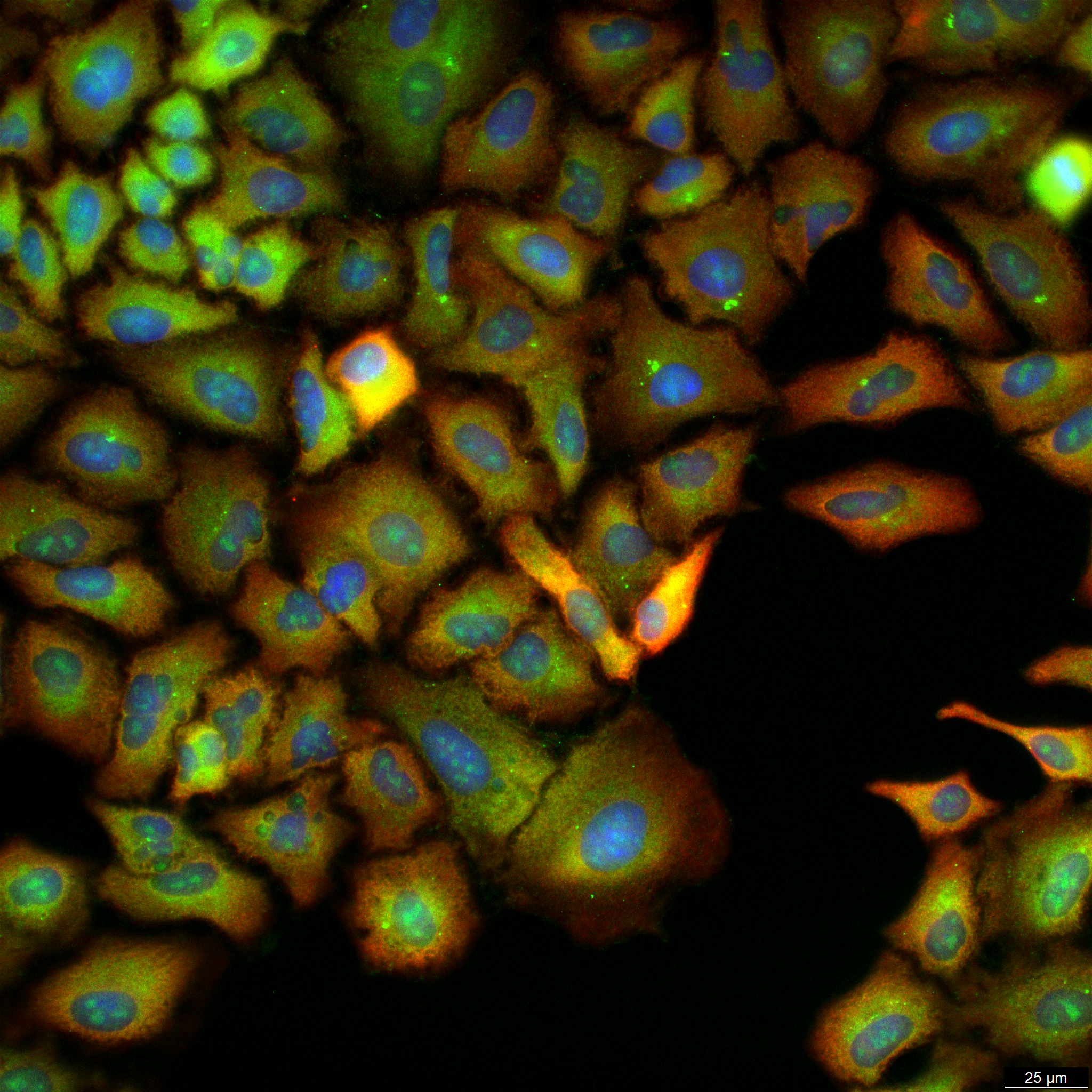

Supplement: Supplementary file 16 — Figure EV5 Source Data [file 44318_2025_421_MOESM16_ESM.zip › EV5/EV5N/lFNγ.tif]

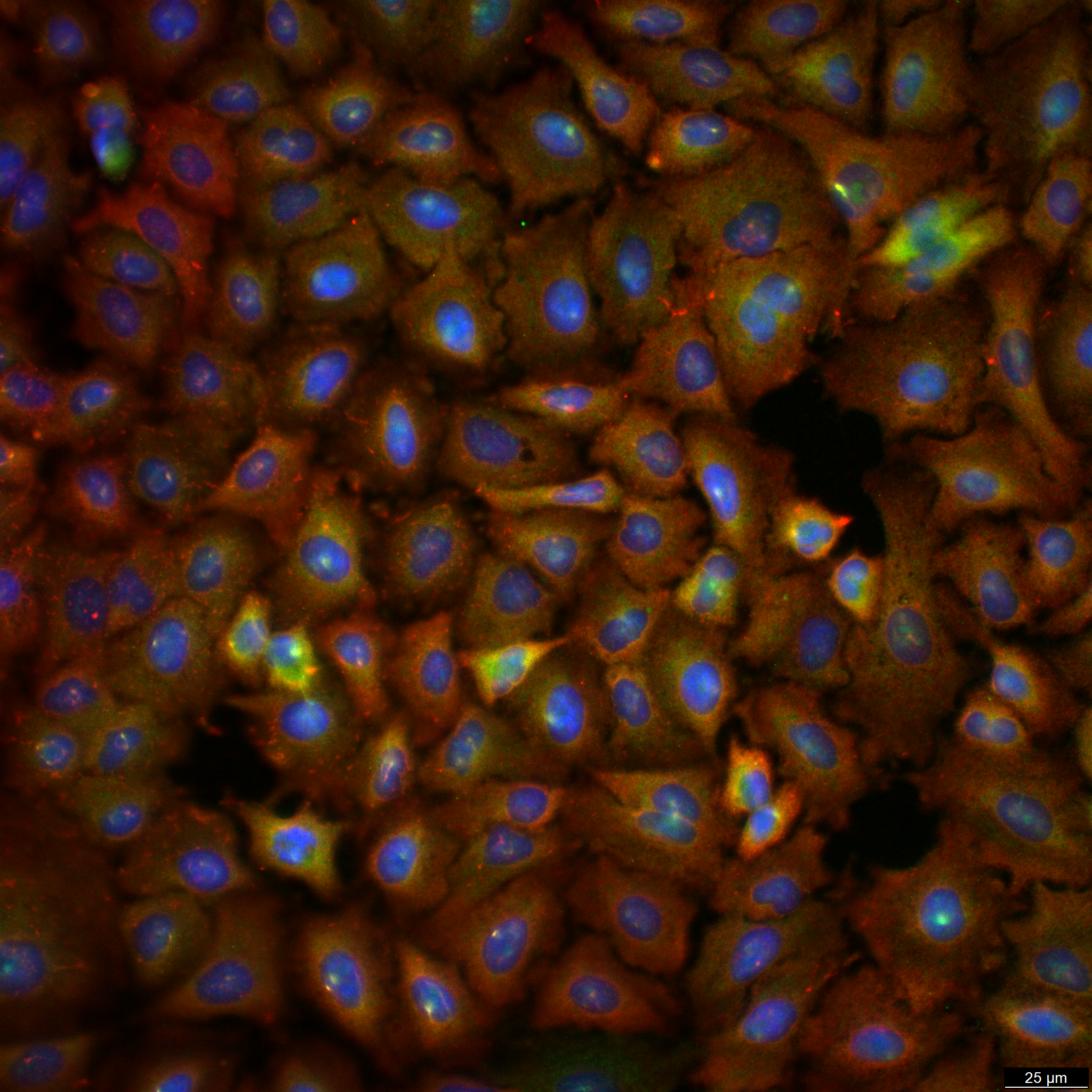

Supplement: Supplementary file 16 — Figure EV5 Source Data [file 44318_2025_421_MOESM16_ESM.zip › EV5/EV5N/Torin 1.tif]

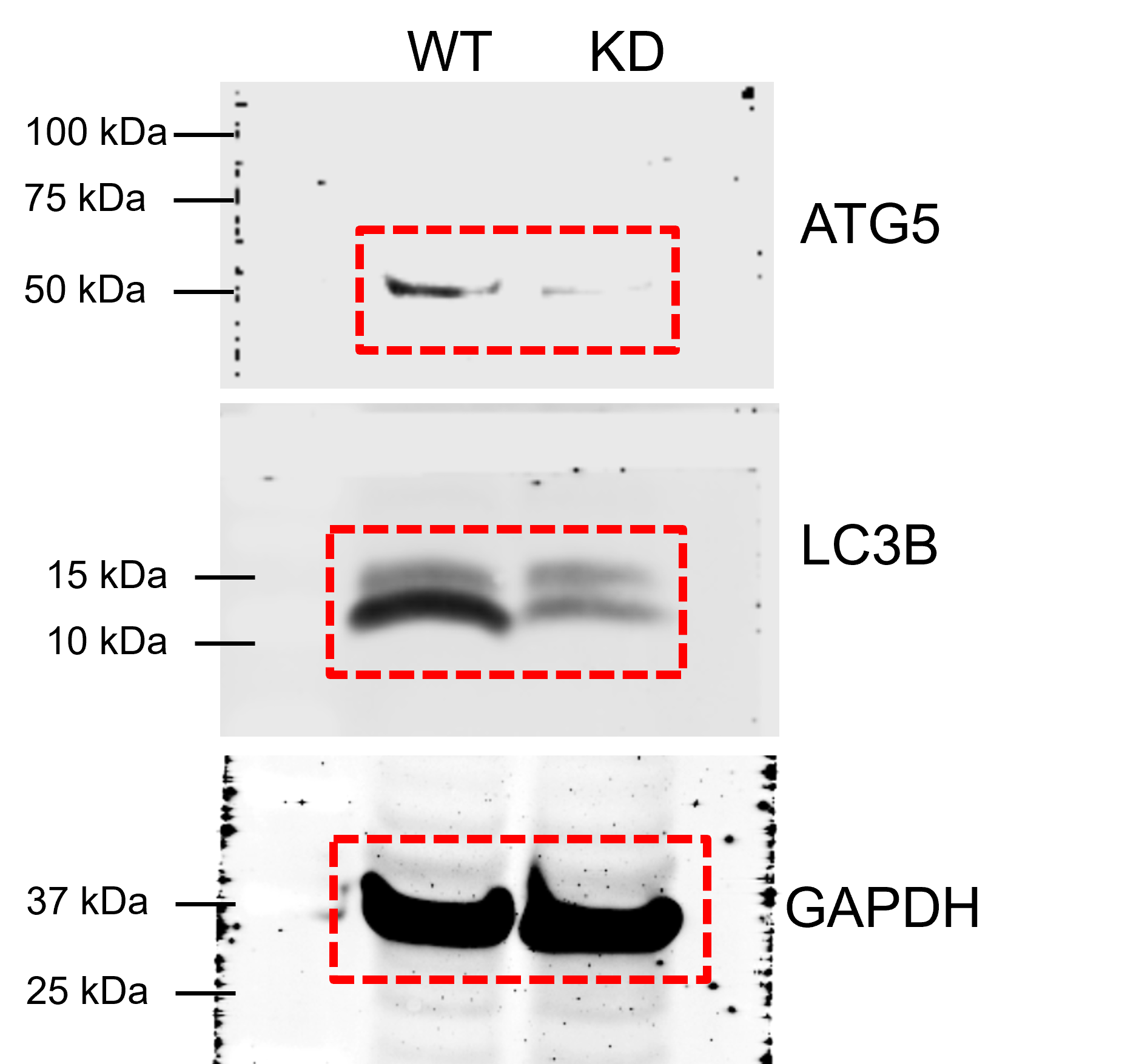

Supplement: Supplementary file 17 — Figure EV6 Source Data [file 44318_2025_421_MOESM17_ESM.zip › EV6/EV6A.tif]

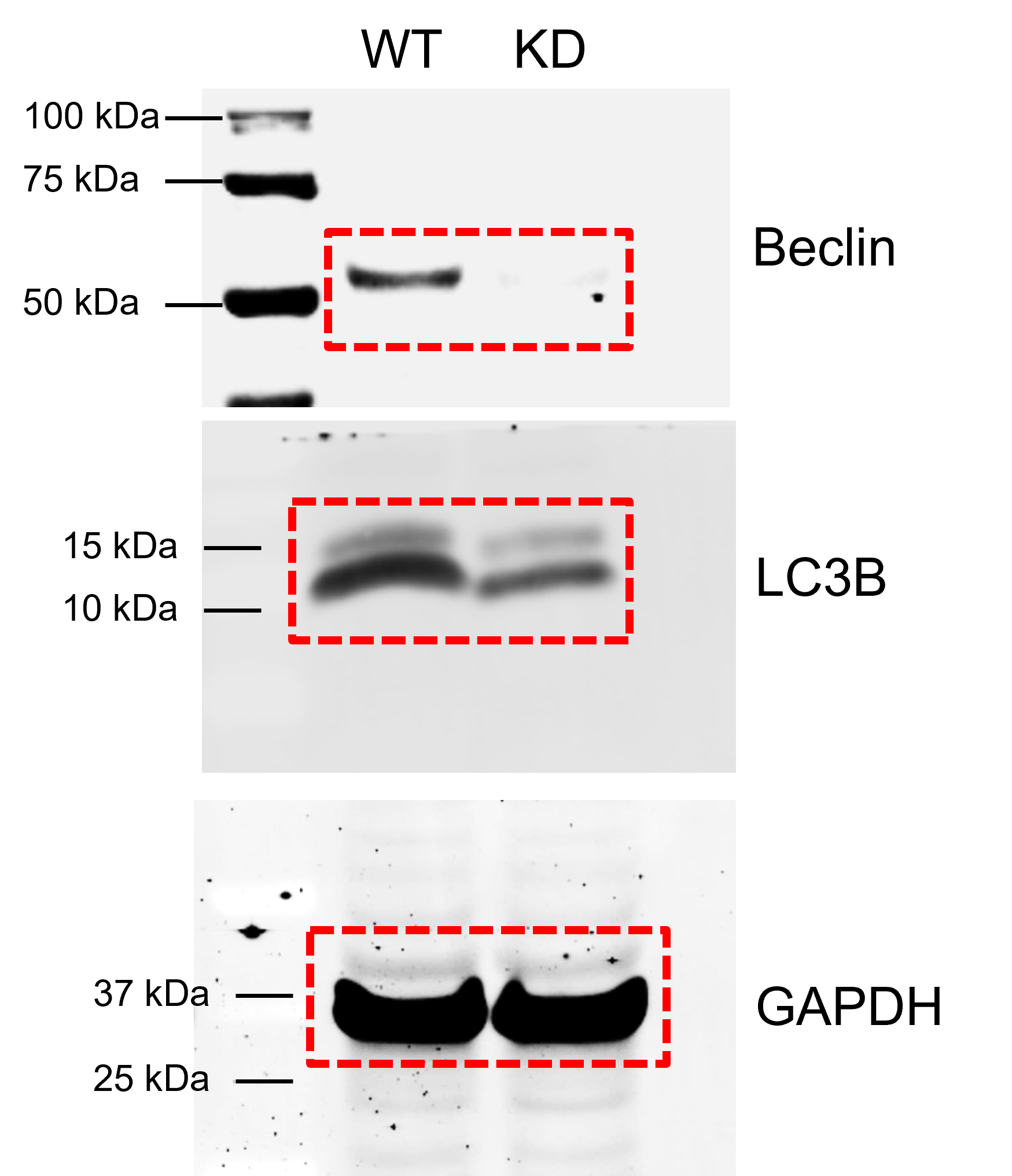

Supplement: Supplementary file 17 — Figure EV6 Source Data [file 44318_2025_421_MOESM17_ESM.zip › EV6/EV6B.tif]

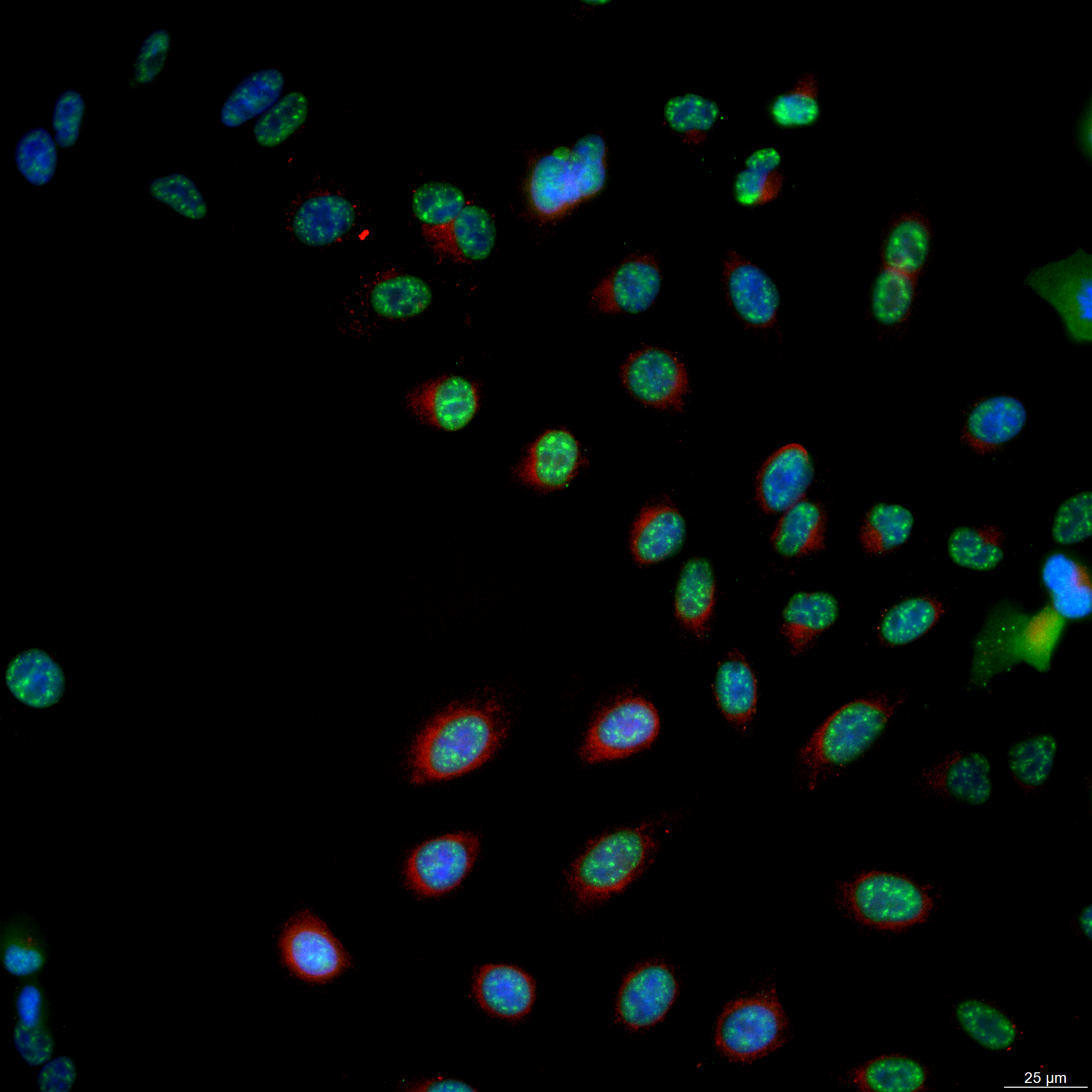

Supplement: Supplementary file 17 — Figure EV6 Source Data [file 44318_2025_421_MOESM17_ESM.zip › EV6/EV6C/CONTROL .tif]

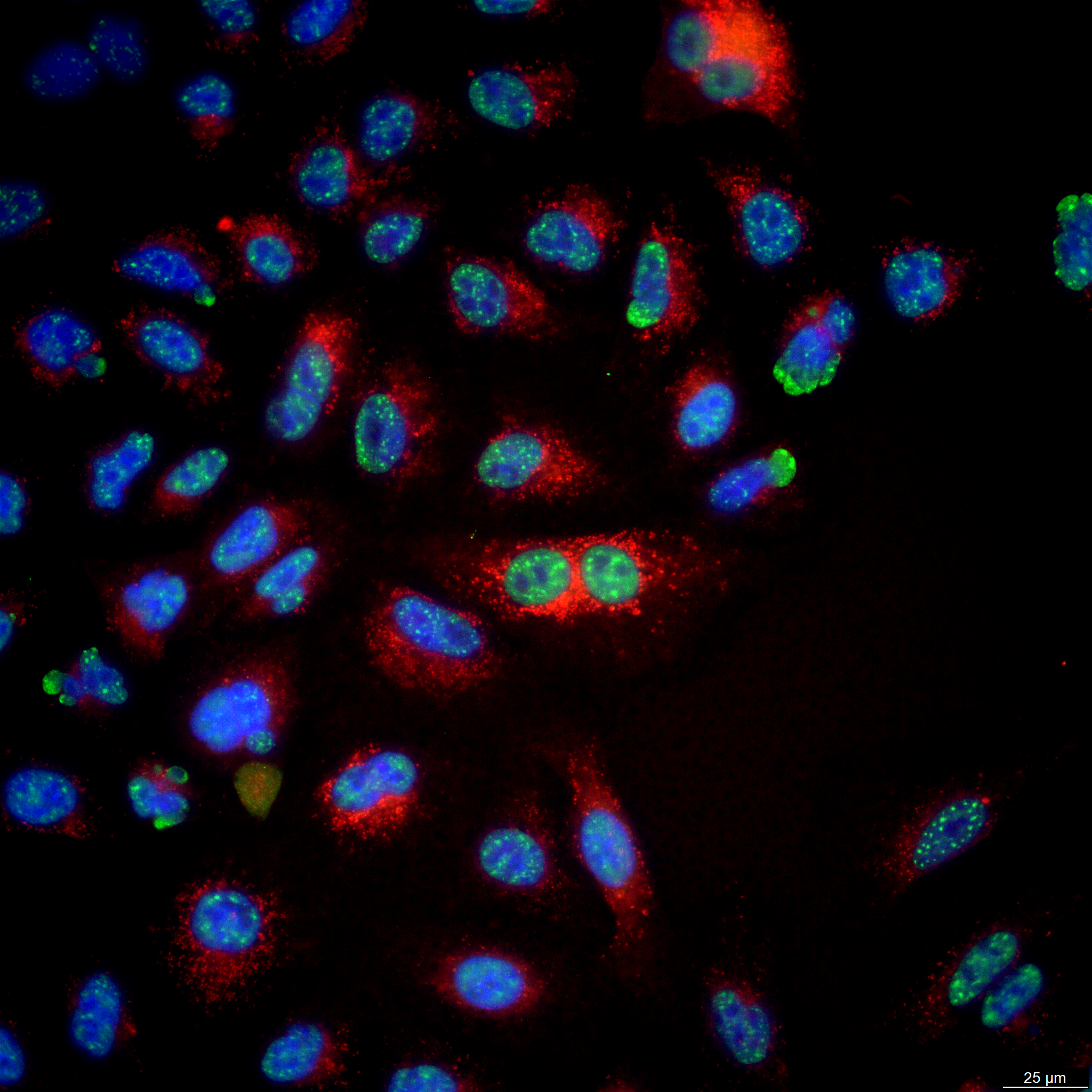

Supplement: Supplementary file 17 — Figure EV6 Source Data [file 44318_2025_421_MOESM17_ESM.zip › EV6/EV6C/lFNγ+MG132 (12 h).tif]

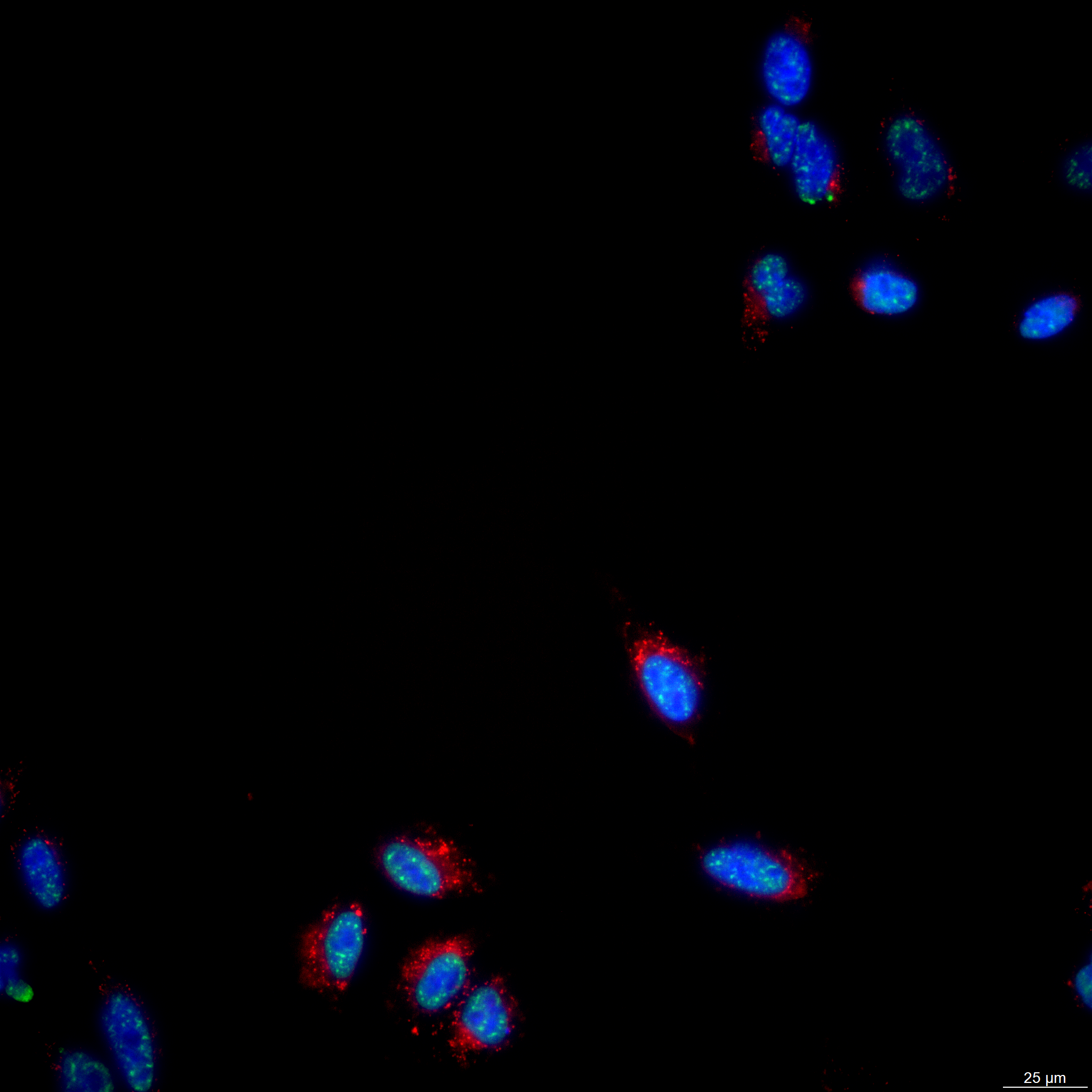

Supplement: Supplementary file 17 — Figure EV6 Source Data [file 44318_2025_421_MOESM17_ESM.zip › EV6/EV6C/lFNγ+MG132 (6 h).tif]

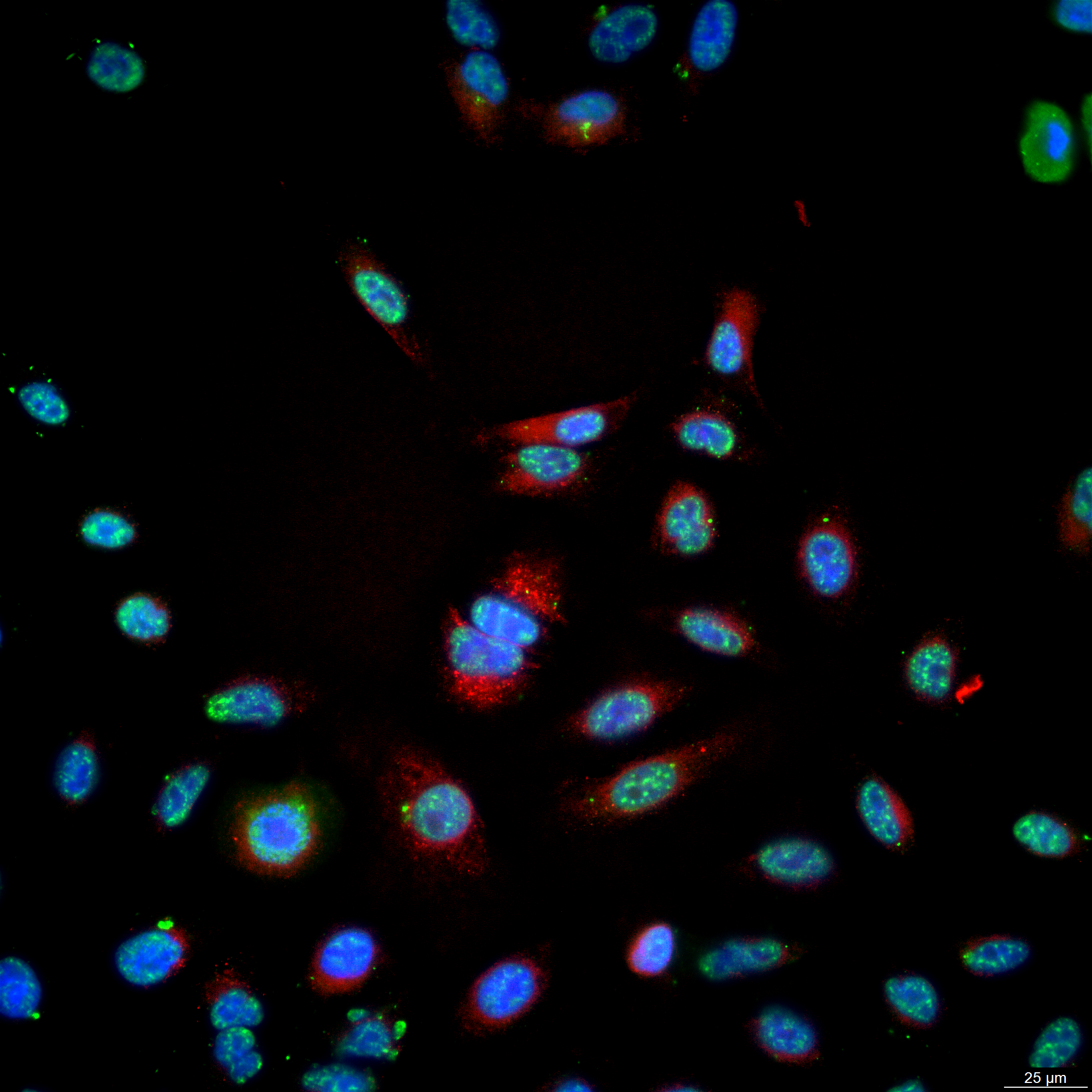

Supplement: Supplementary file 17 — Figure EV6 Source Data [file 44318_2025_421_MOESM17_ESM.zip › EV6/EV6C/lFNγ.tif]

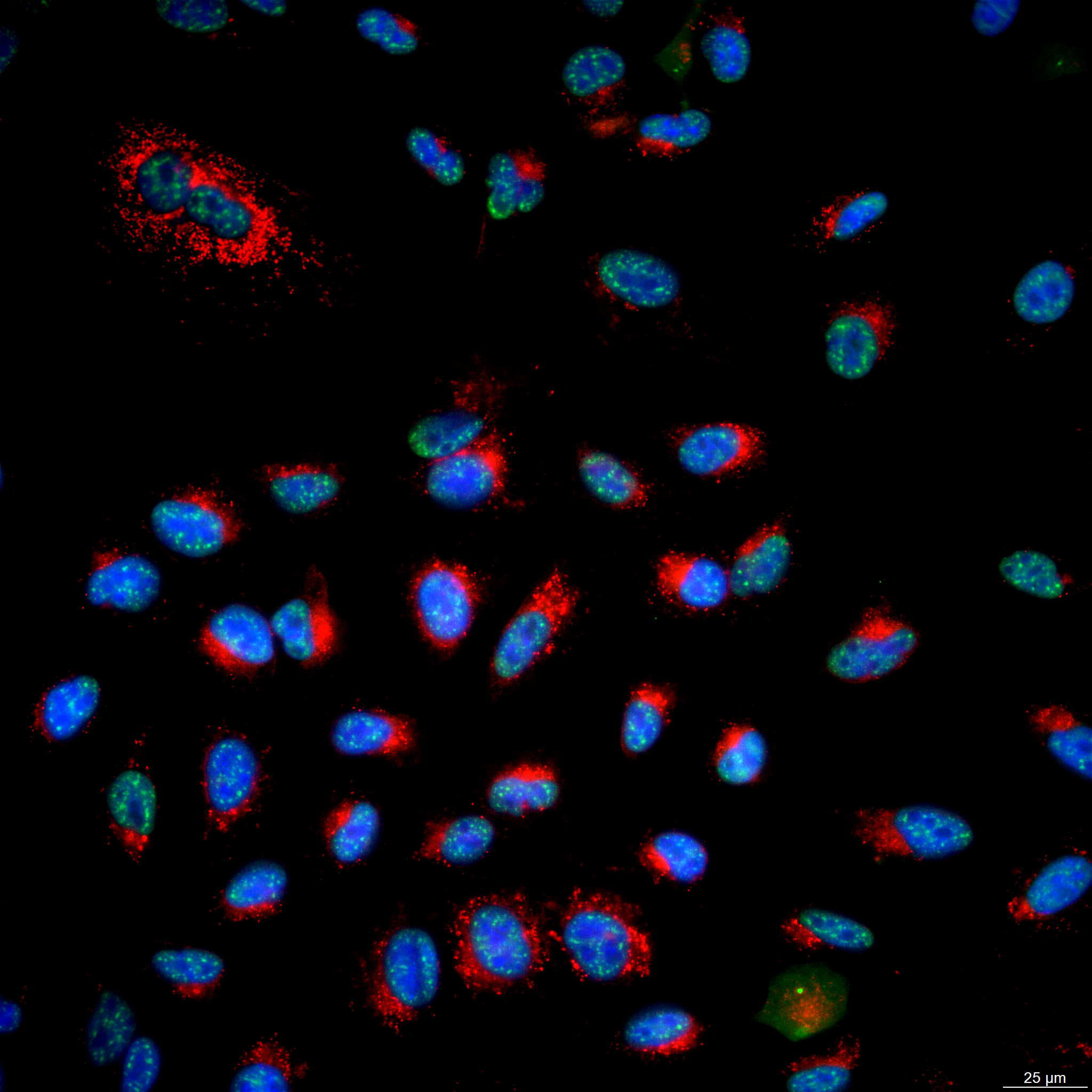

Supplement: Supplementary file 17 — Figure EV6 Source Data [file 44318_2025_421_MOESM17_ESM.zip › EV6/EV6C/MG132 (12 h).tif]

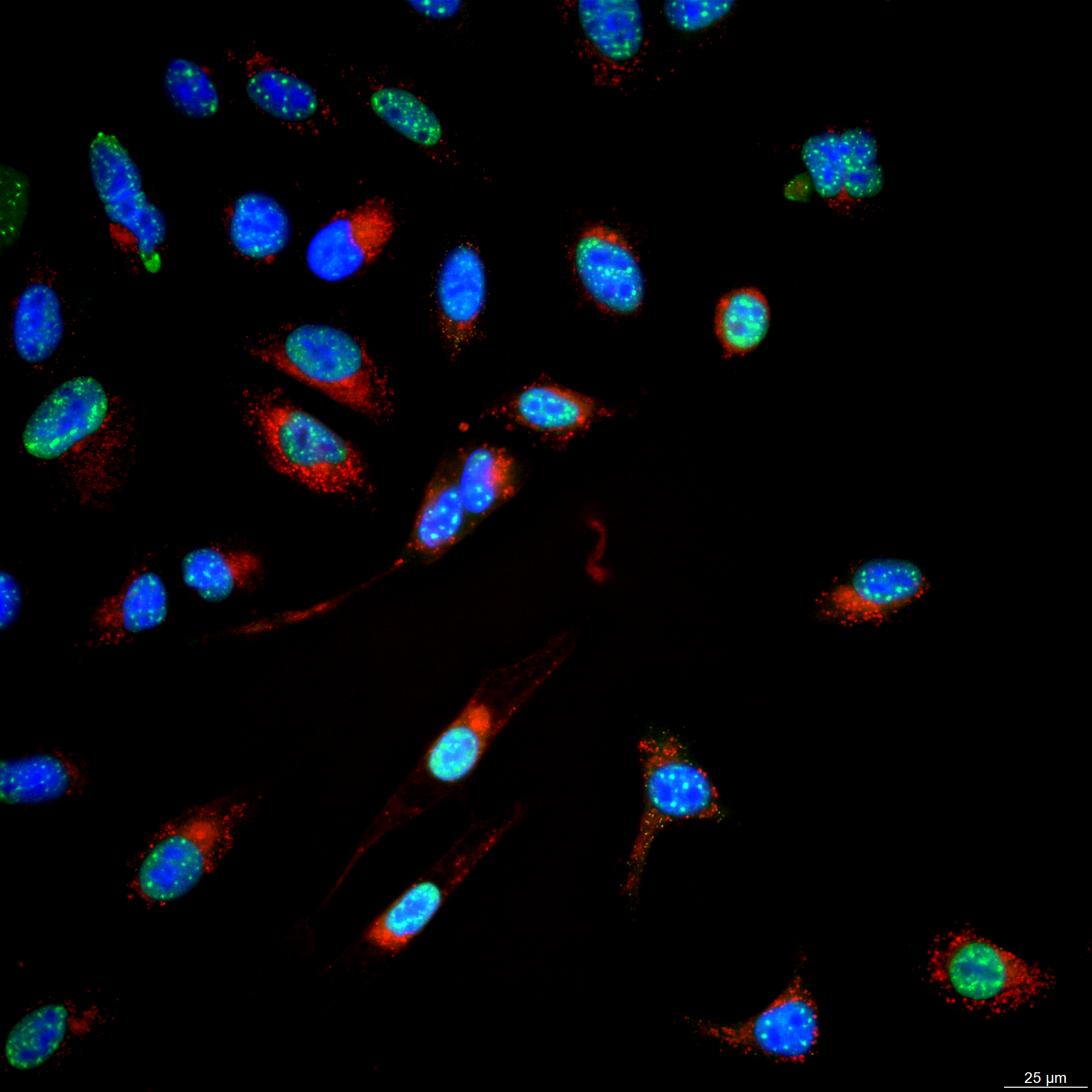

Supplement: Supplementary file 17 — Figure EV6 Source Data [file 44318_2025_421_MOESM17_ESM.zip › EV6/EV6C/MG132 (6 h).tif]

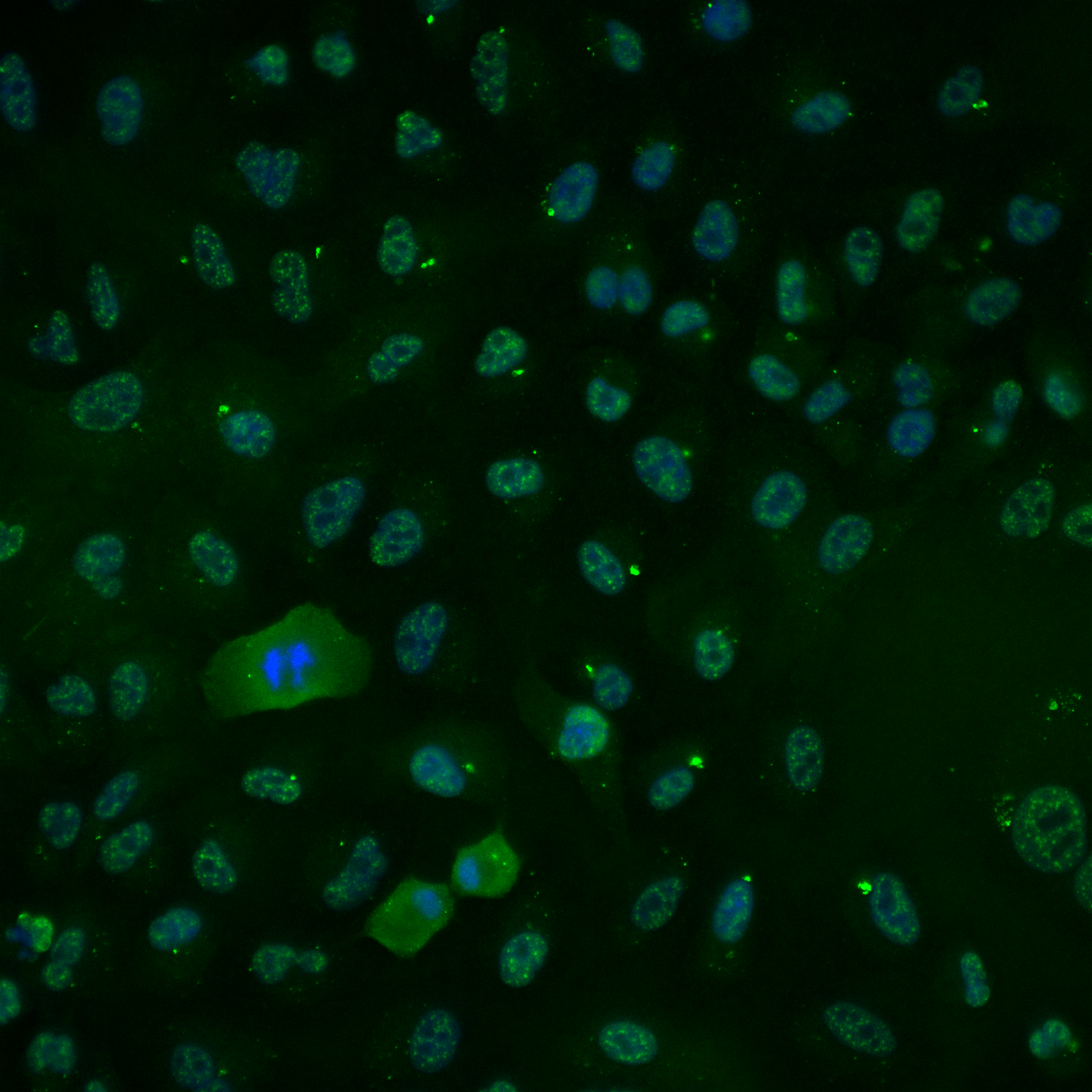

Supplement: Supplementary file 17 — Figure EV6 Source Data [file 44318_2025_421_MOESM17_ESM.zip › EV6/EV6D/0.1h MG132_IFNg.tif]

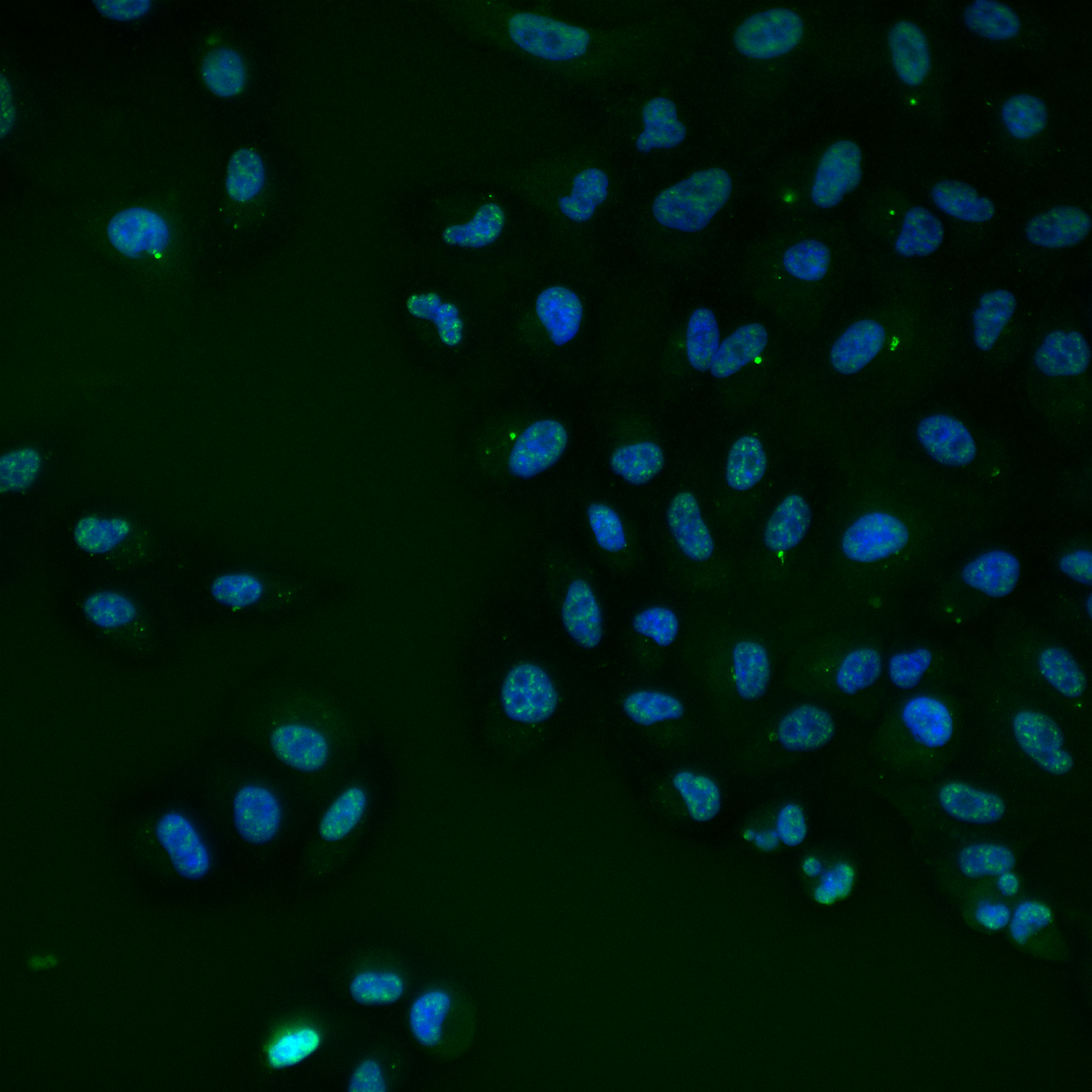

Supplement: Supplementary file 17 — Figure EV6 Source Data [file 44318_2025_421_MOESM17_ESM.zip › EV6/EV6D/0.5h MG132_IFNg.tif]

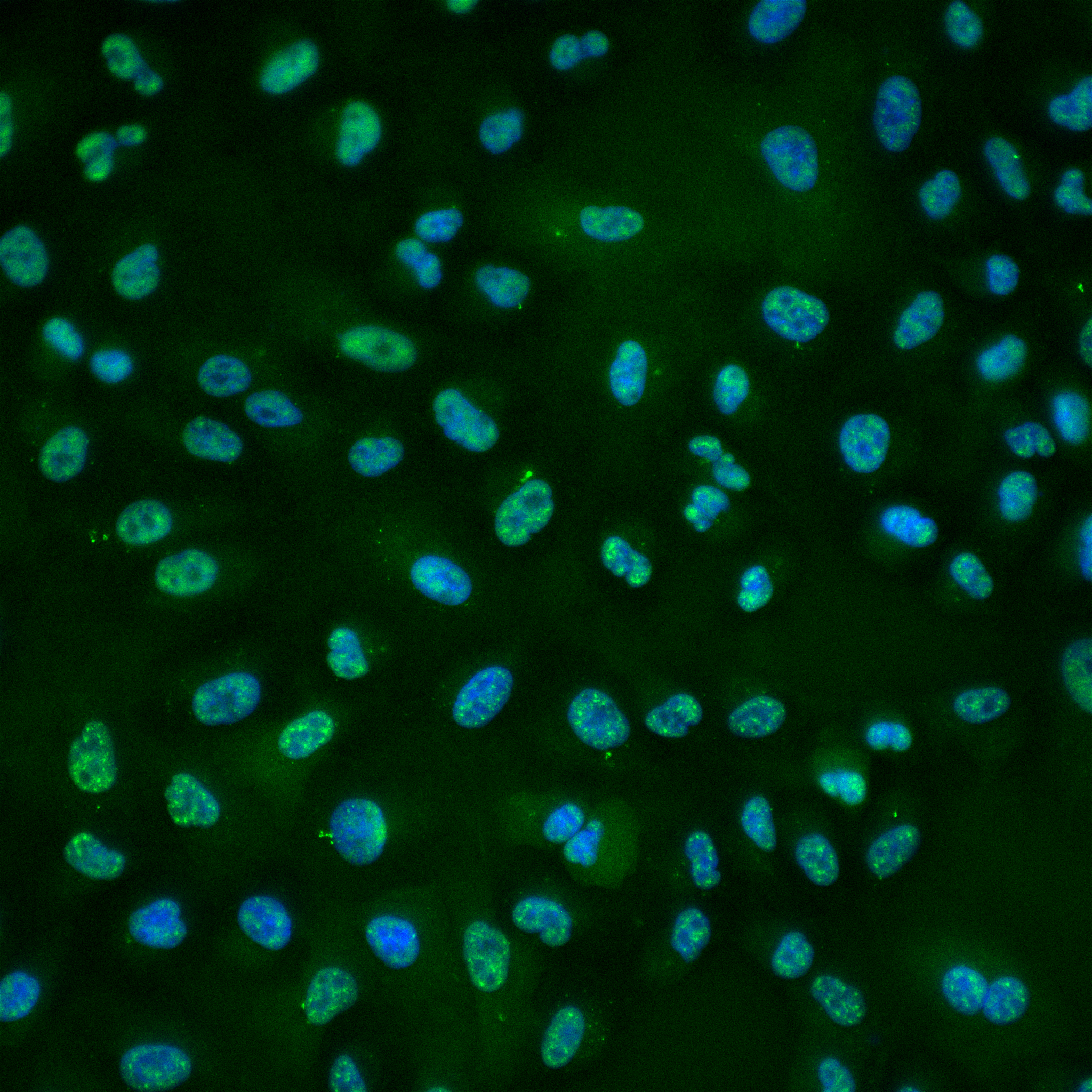

Supplement: Supplementary file 17 — Figure EV6 Source Data [file 44318_2025_421_MOESM17_ESM.zip › EV6/EV6D/1.5h MG132_IFNg.tif]

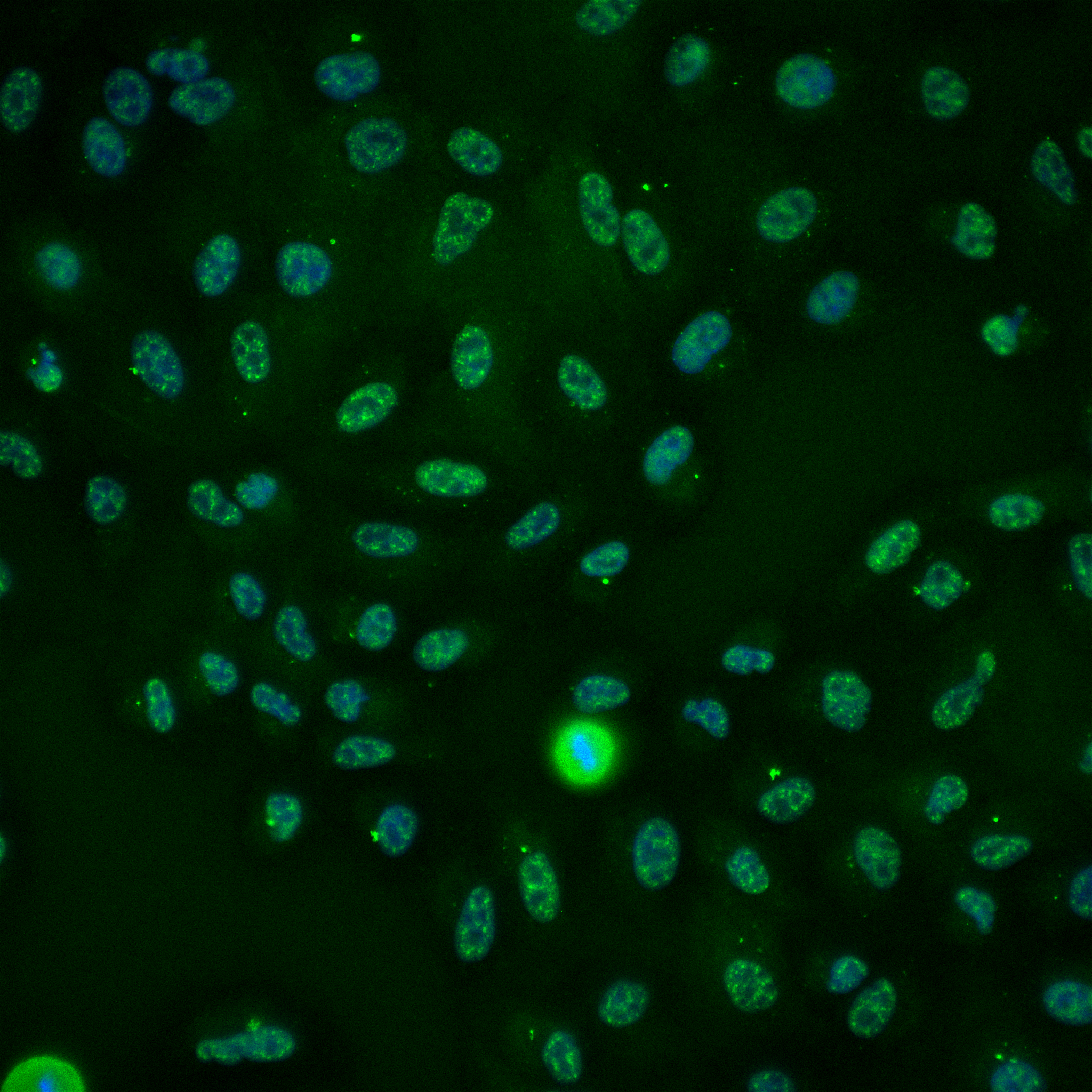

Supplement: Supplementary file 17 — Figure EV6 Source Data [file 44318_2025_421_MOESM17_ESM.zip › EV6/EV6D/1h MG132_IFNg.tif]

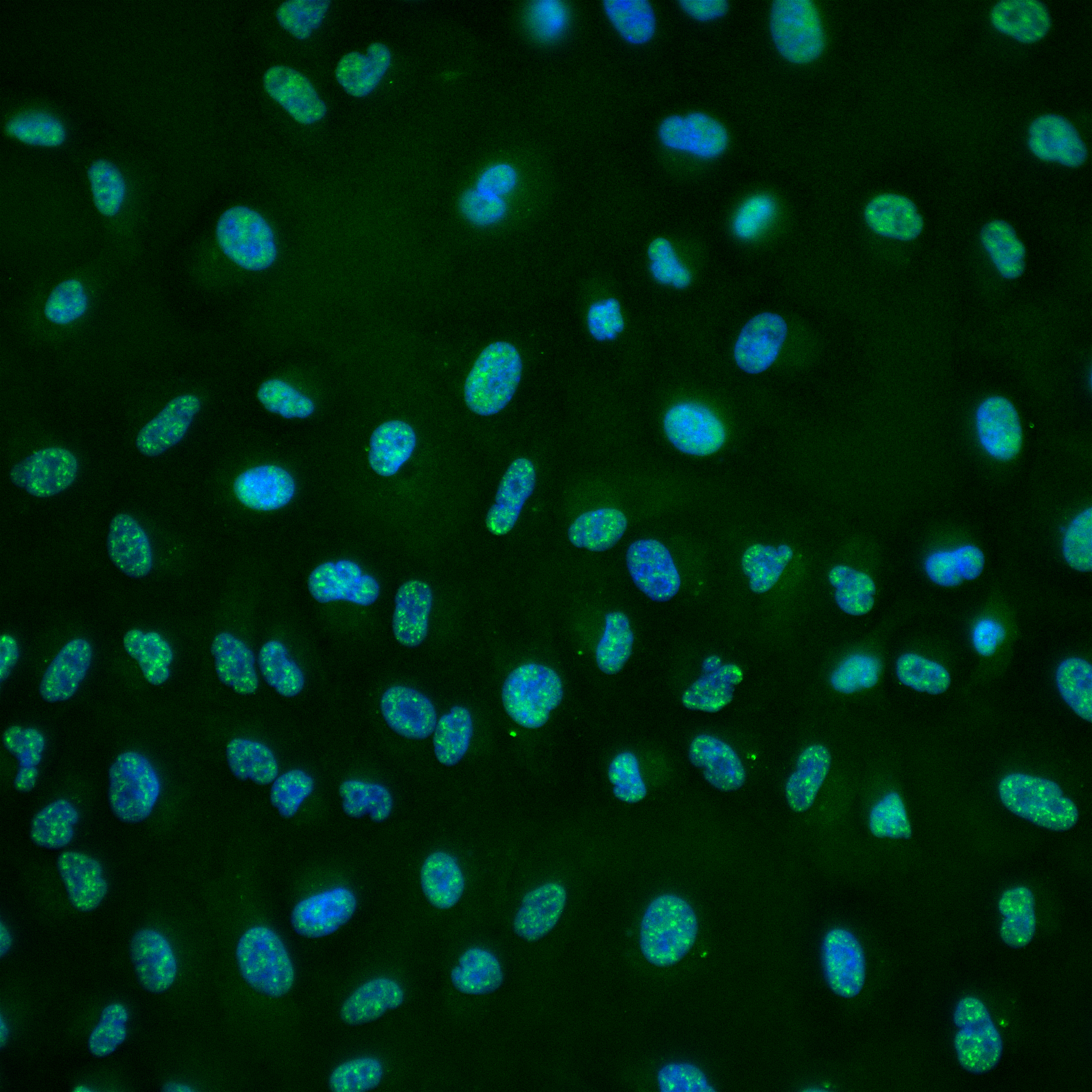

Supplement: Supplementary file 17 — Figure EV6 Source Data [file 44318_2025_421_MOESM17_ESM.zip › EV6/EV6D/3h MG132_IFNg.tif]

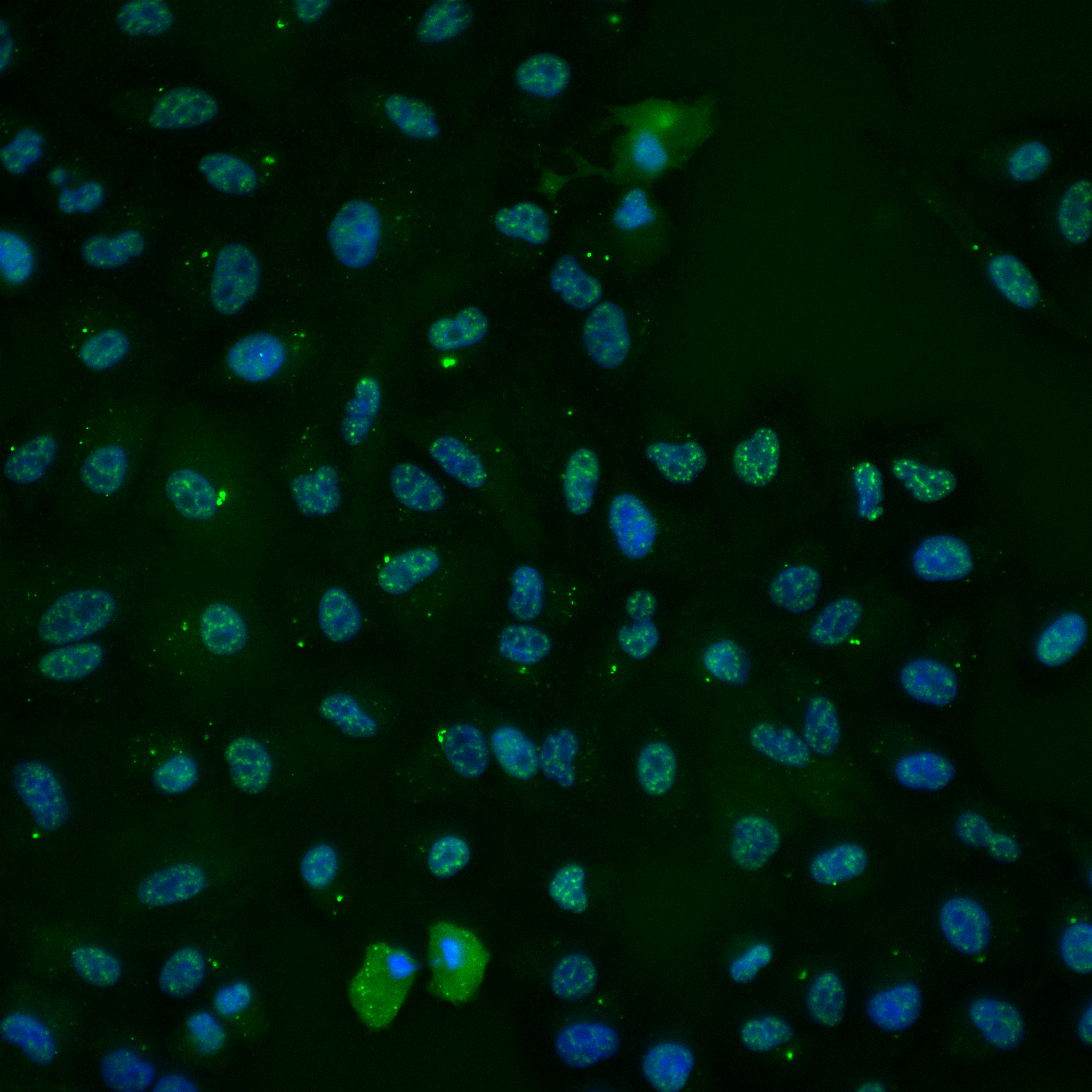

Supplement: Supplementary file 17 — Figure EV6 Source Data [file 44318_2025_421_MOESM17_ESM.zip › EV6/EV6D/6h DMSO_IFNg.tif]

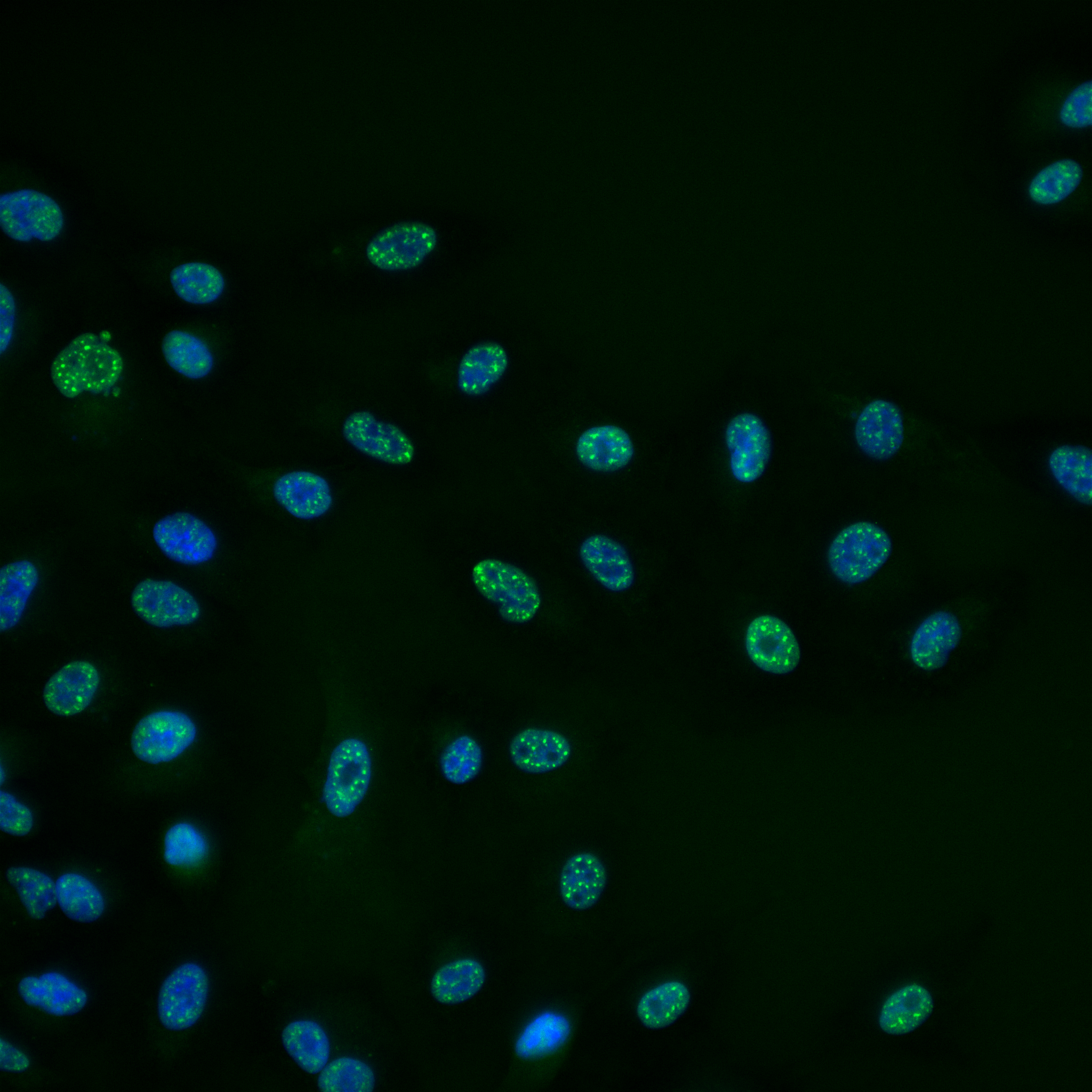

Supplement: Supplementary file 17 — Figure EV6 Source Data [file 44318_2025_421_MOESM17_ESM.zip › EV6/EV6D/6h MG132_IFNg.tif]

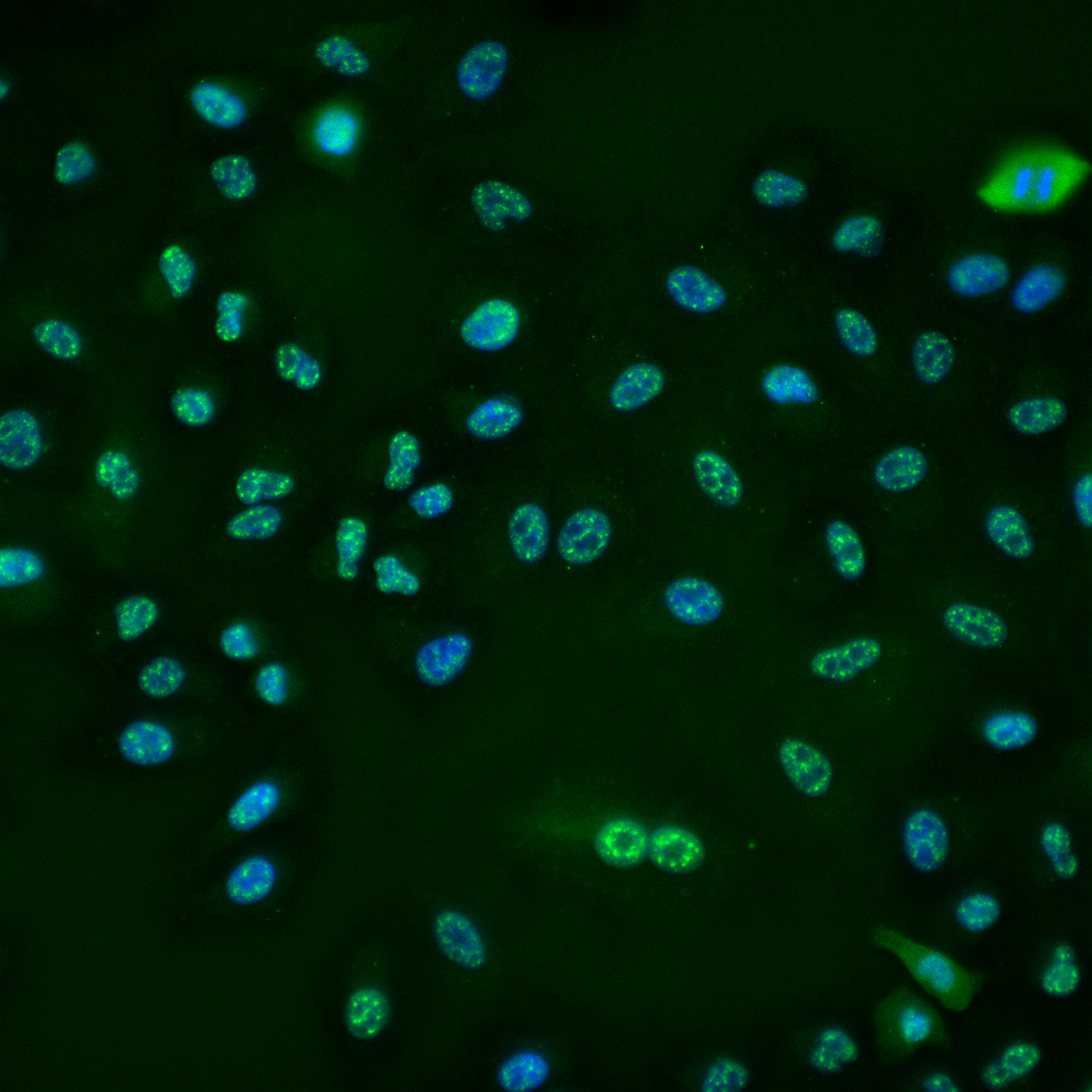

Supplement: Supplementary file 17 — Figure EV6 Source Data [file 44318_2025_421_MOESM17_ESM.zip › EV6/EV6D/6hr DMSO.tif]

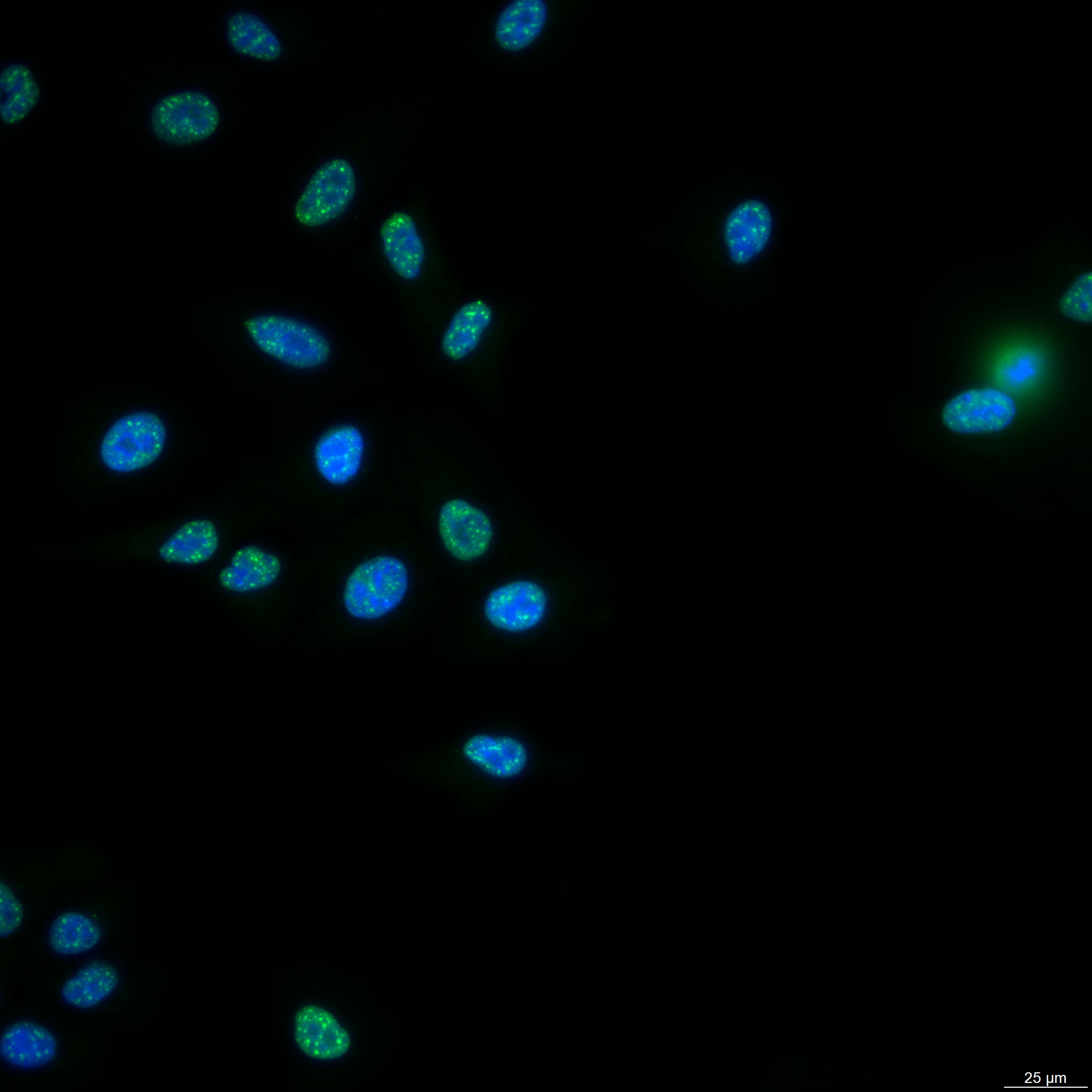

Supplement: Supplementary file 17 — Figure EV6 Source Data [file 44318_2025_421_MOESM17_ESM.zip › EV6/EV6E/CONTROL.tif]

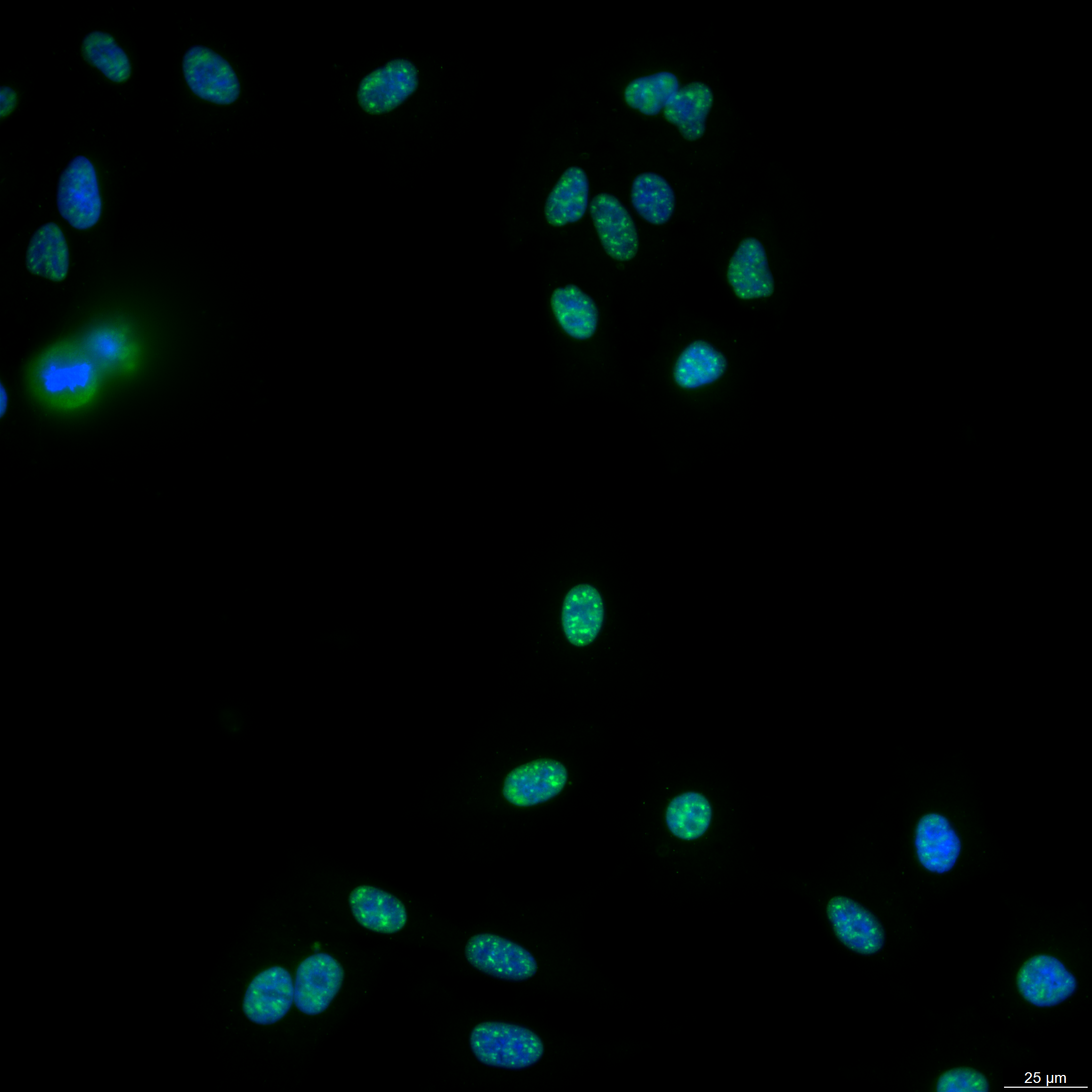

Supplement: Supplementary file 17 — Figure EV6 Source Data [file 44318_2025_421_MOESM17_ESM.zip › EV6/EV6E/lFNγ+BORT (6 h).tif]

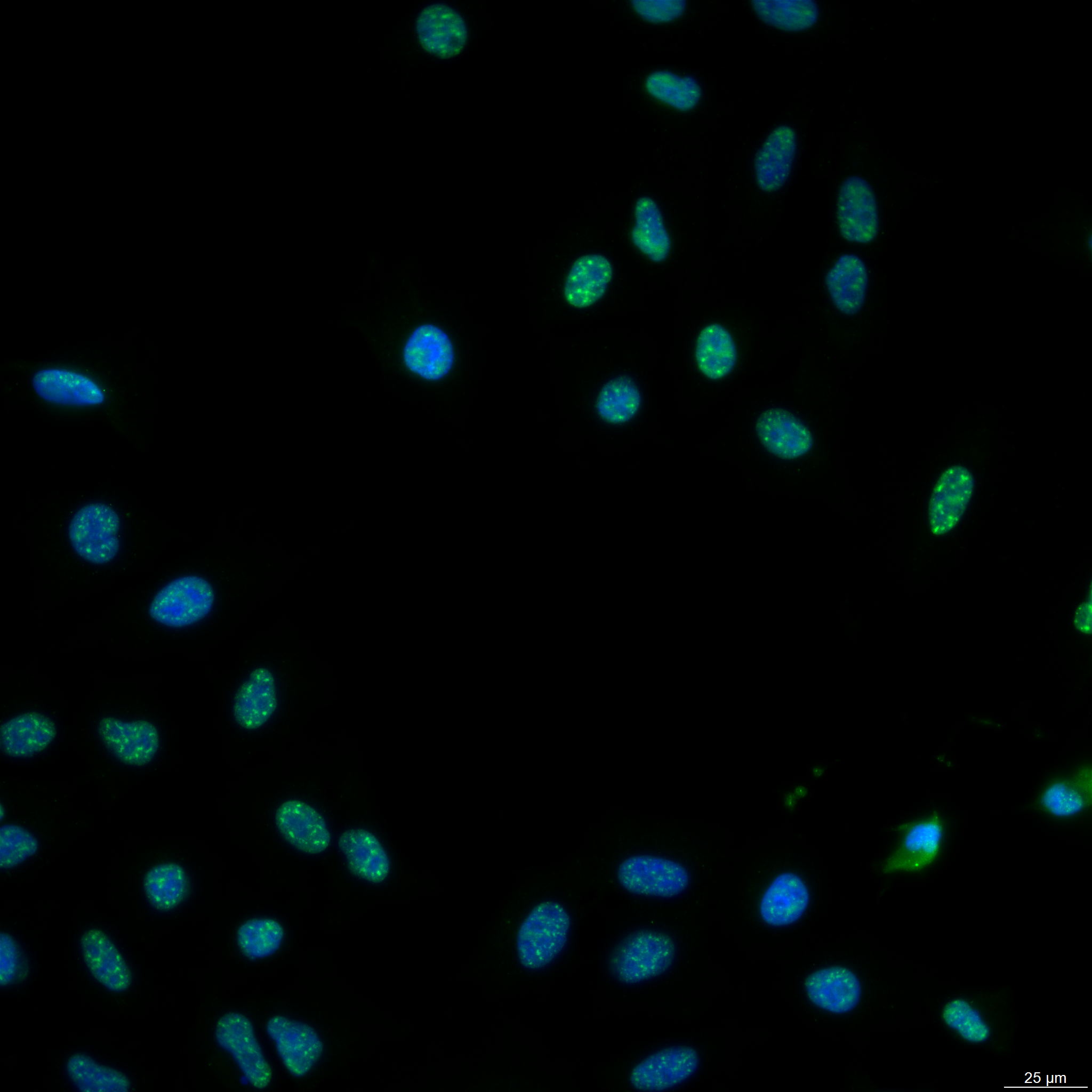

Supplement: Supplementary file 17 — Figure EV6 Source Data [file 44318_2025_421_MOESM17_ESM.zip › EV6/EV6E/lFNγ+Epox (6 h).tif]

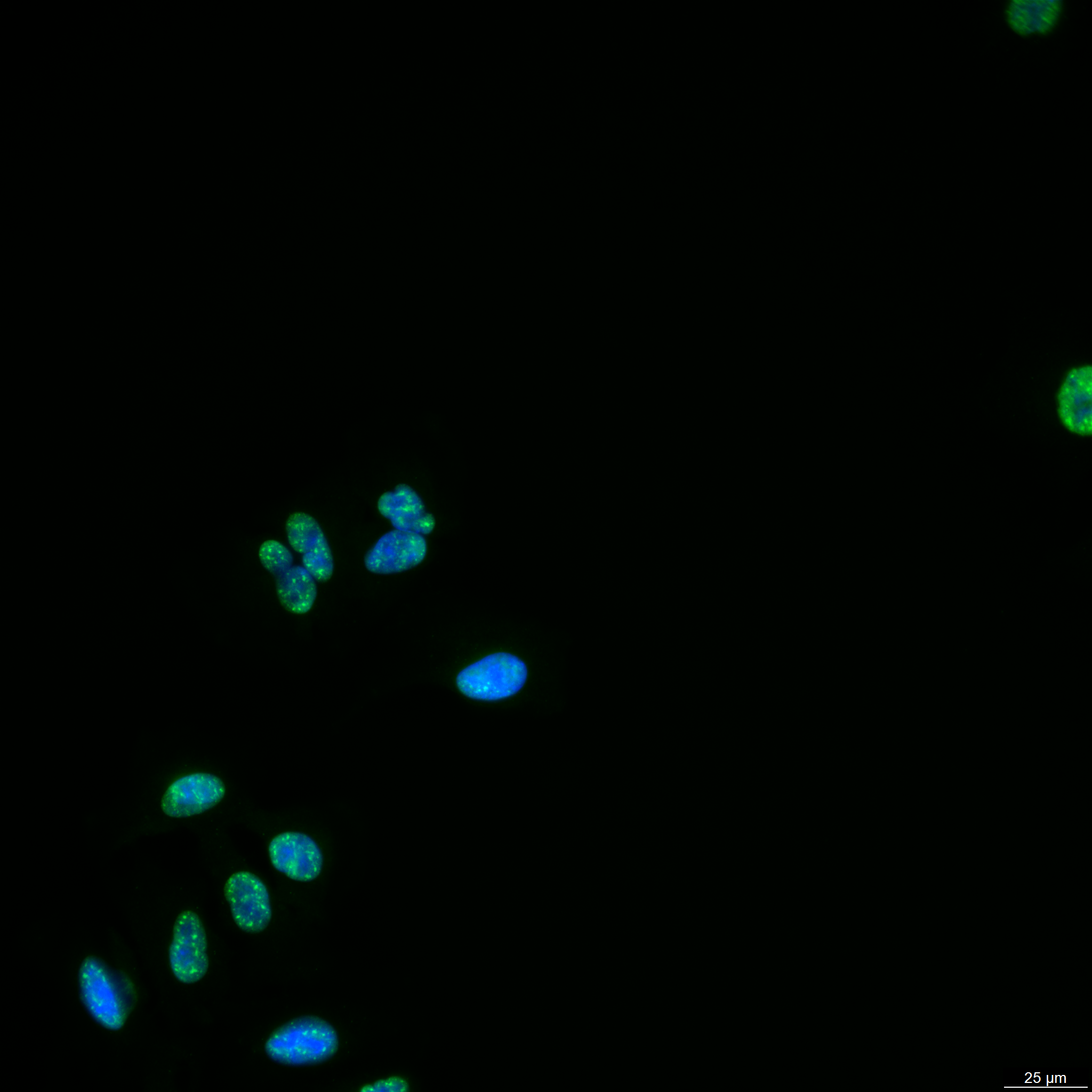

Supplement: Supplementary file 17 — Figure EV6 Source Data [file 44318_2025_421_MOESM17_ESM.zip › EV6/EV6E/lFNγ+MG132 (6 h).tif]

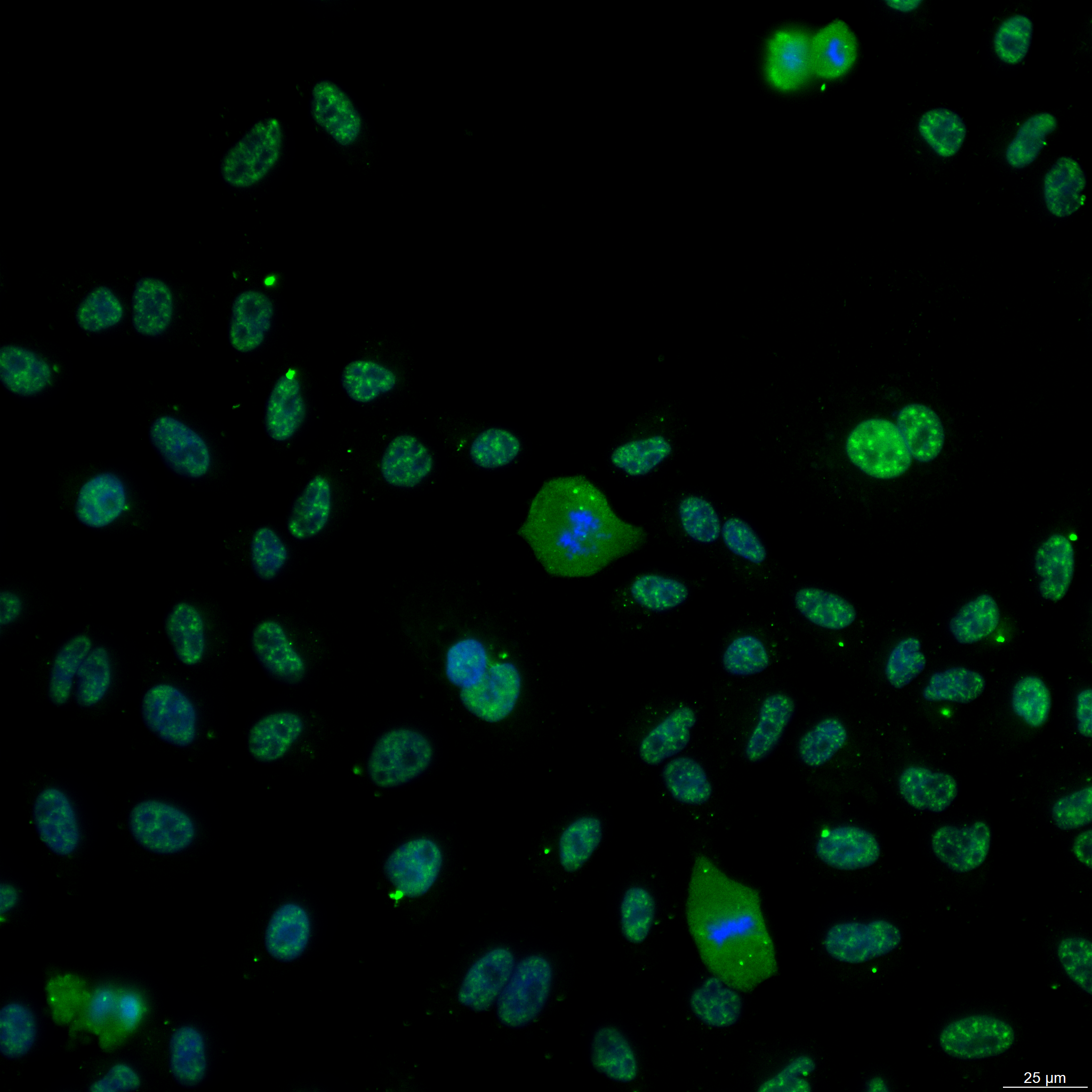

Supplement: Supplementary file 17 — Figure EV6 Source Data [file 44318_2025_421_MOESM17_ESM.zip › EV6/EV6E/lFNγ.tif]
