# Supplementary figures and images for: Interferon-induced PARP14-mediated ADP-ribosylation in p62 bodies requires the ubiquitin-proteasome system (part 4 of 4)
Source: EMBO J. 2025 Apr 7;44(10):2741–73. doi: 10.1038/s44318-025-00421-4 (PMC12084362; doi:10.1038/s44318-025-00421-4)

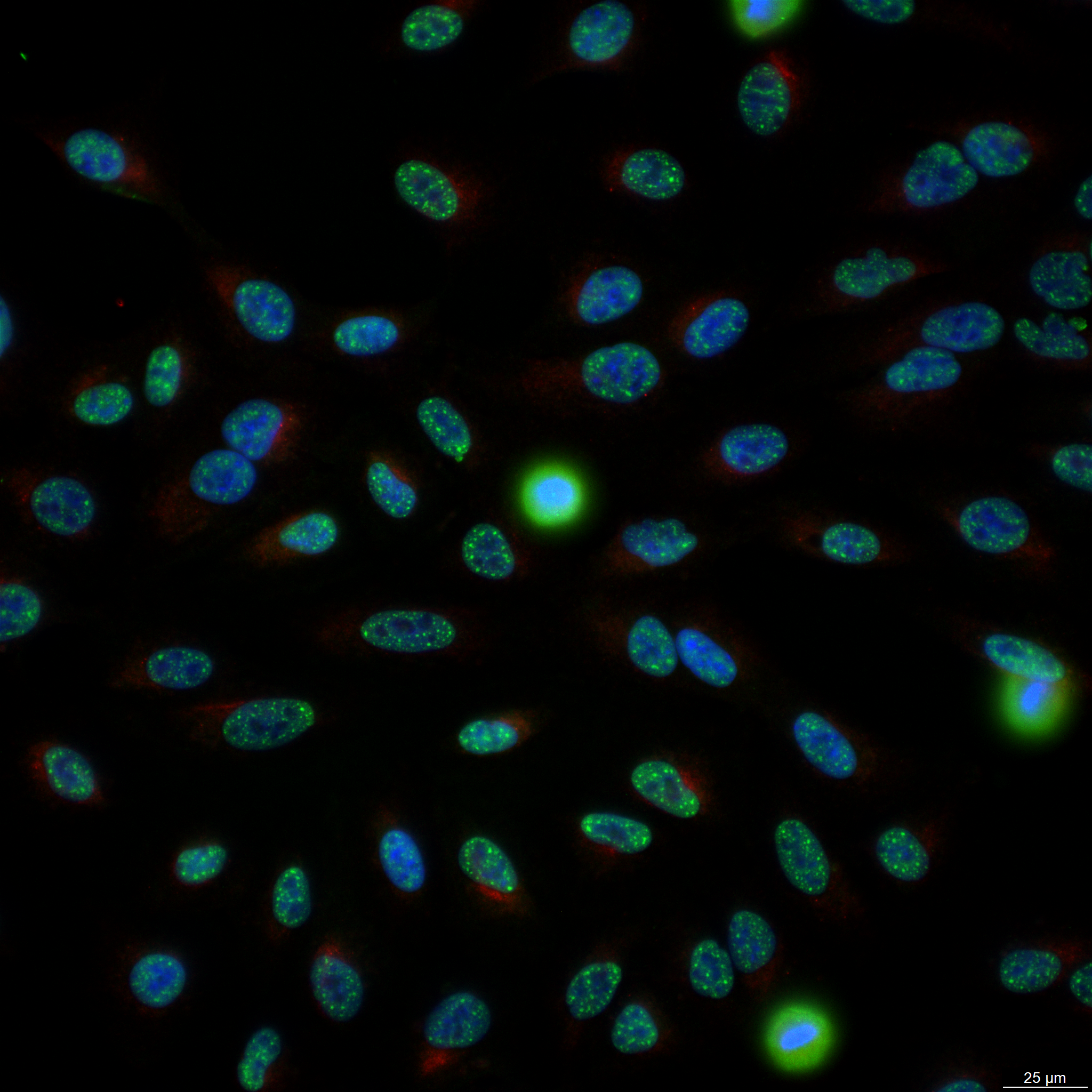

Supplement: Supplementary file 17 — Figure EV6 Source Data [file 44318_2025_421_MOESM17_ESM.zip › EV6/EV6F/Epox.tif]

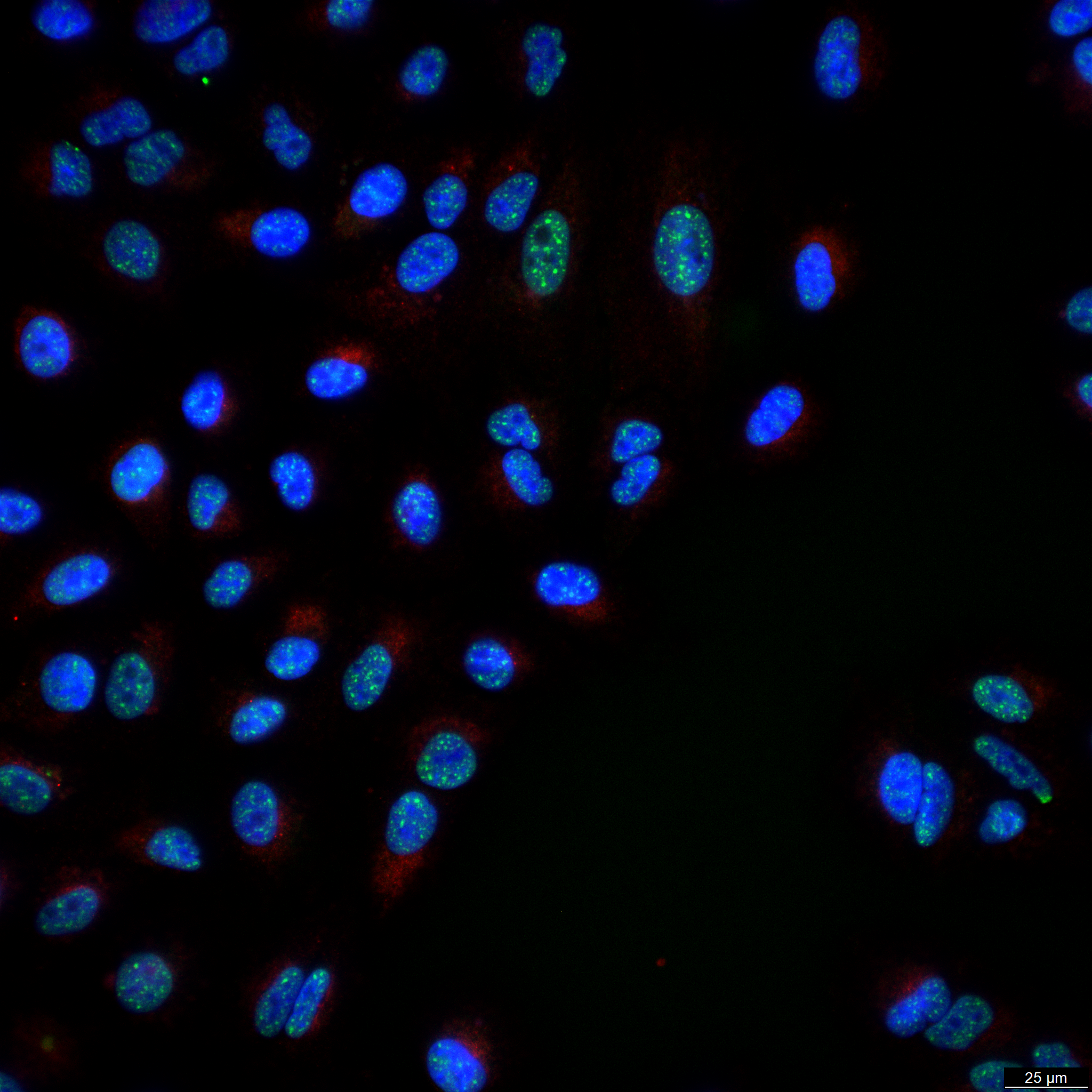

Supplement: Supplementary file 17 — Figure EV6 Source Data [file 44318_2025_421_MOESM17_ESM.zip › EV6/EV6F/MG132.tif]

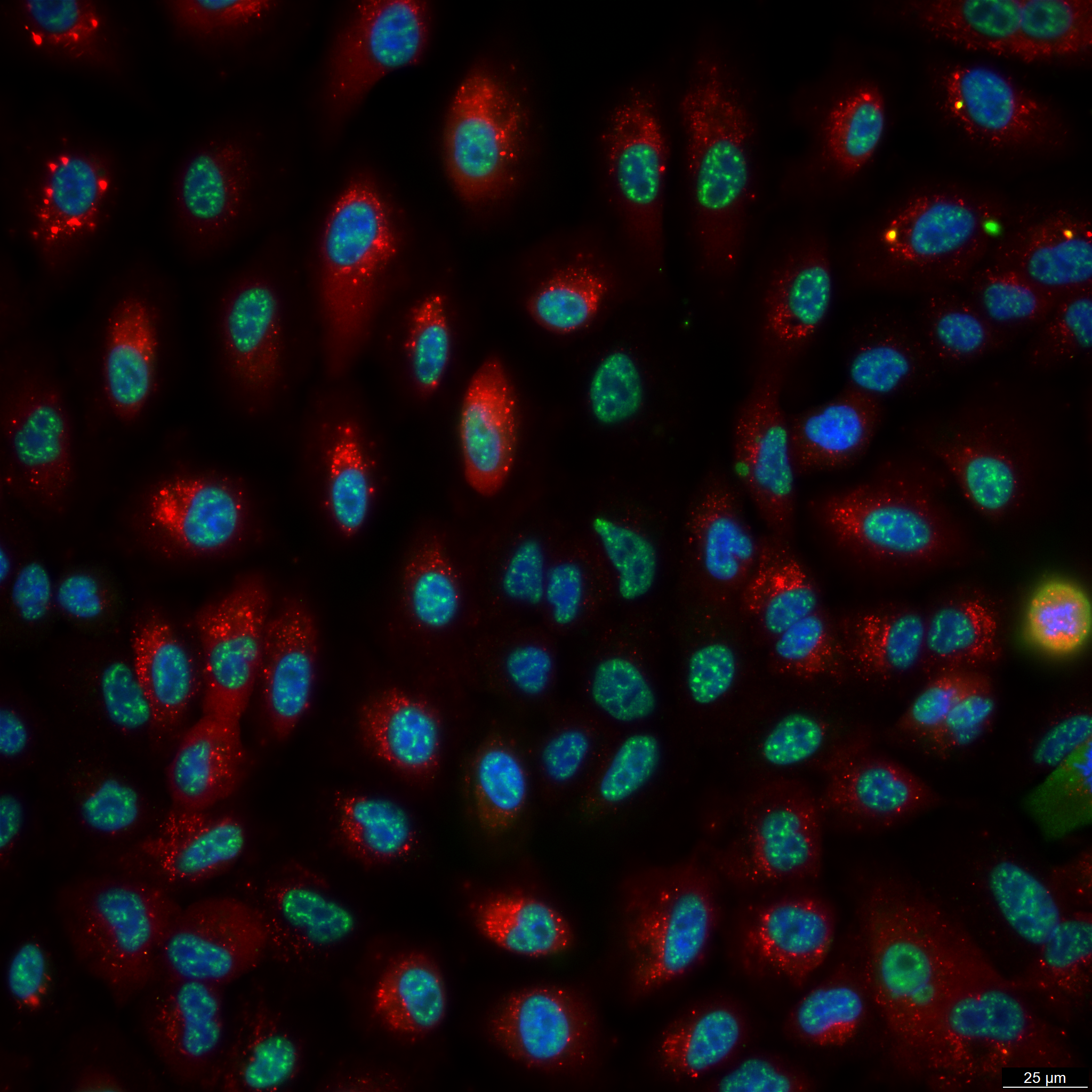

Supplement: Supplementary file 17 — Figure EV6 Source Data [file 44318_2025_421_MOESM17_ESM.zip › EV6/EV6G/Control.tif]

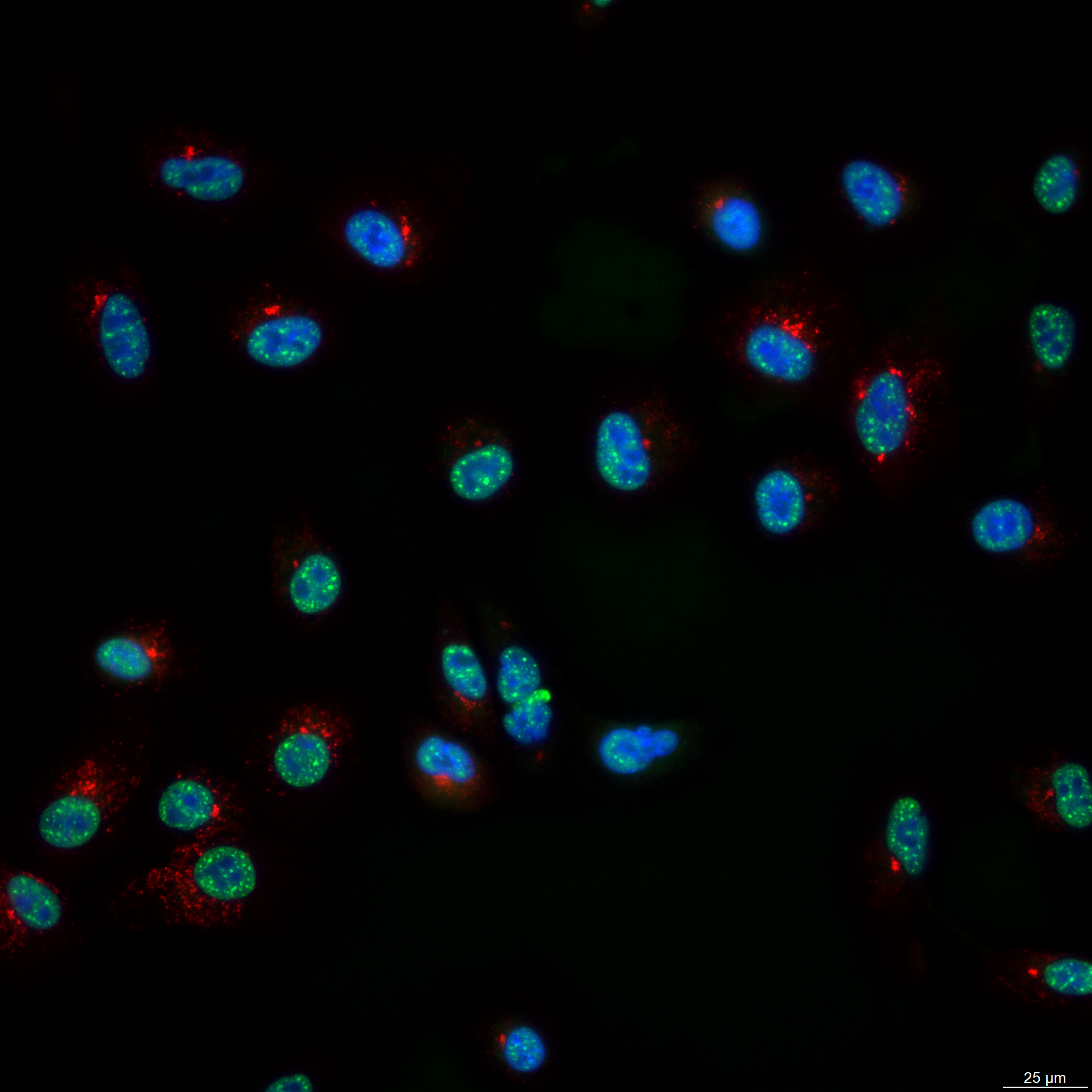

Supplement: Supplementary file 17 — Figure EV6 Source Data [file 44318_2025_421_MOESM17_ESM.zip › EV6/EV6G/Epox.tif]

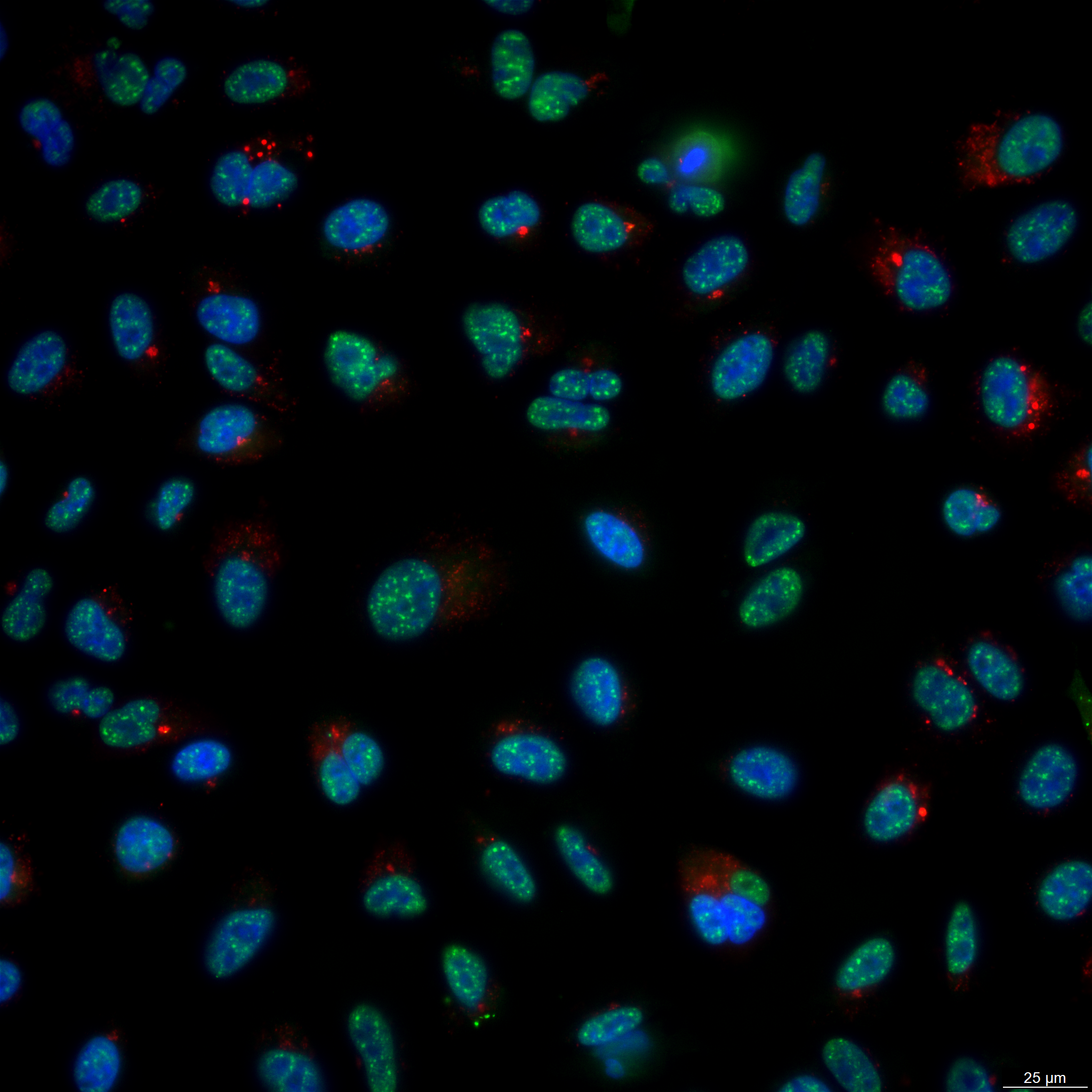

Supplement: Supplementary file 17 — Figure EV6 Source Data [file 44318_2025_421_MOESM17_ESM.zip › EV6/EV6G/lFNγ+Epox (6 h).tif]

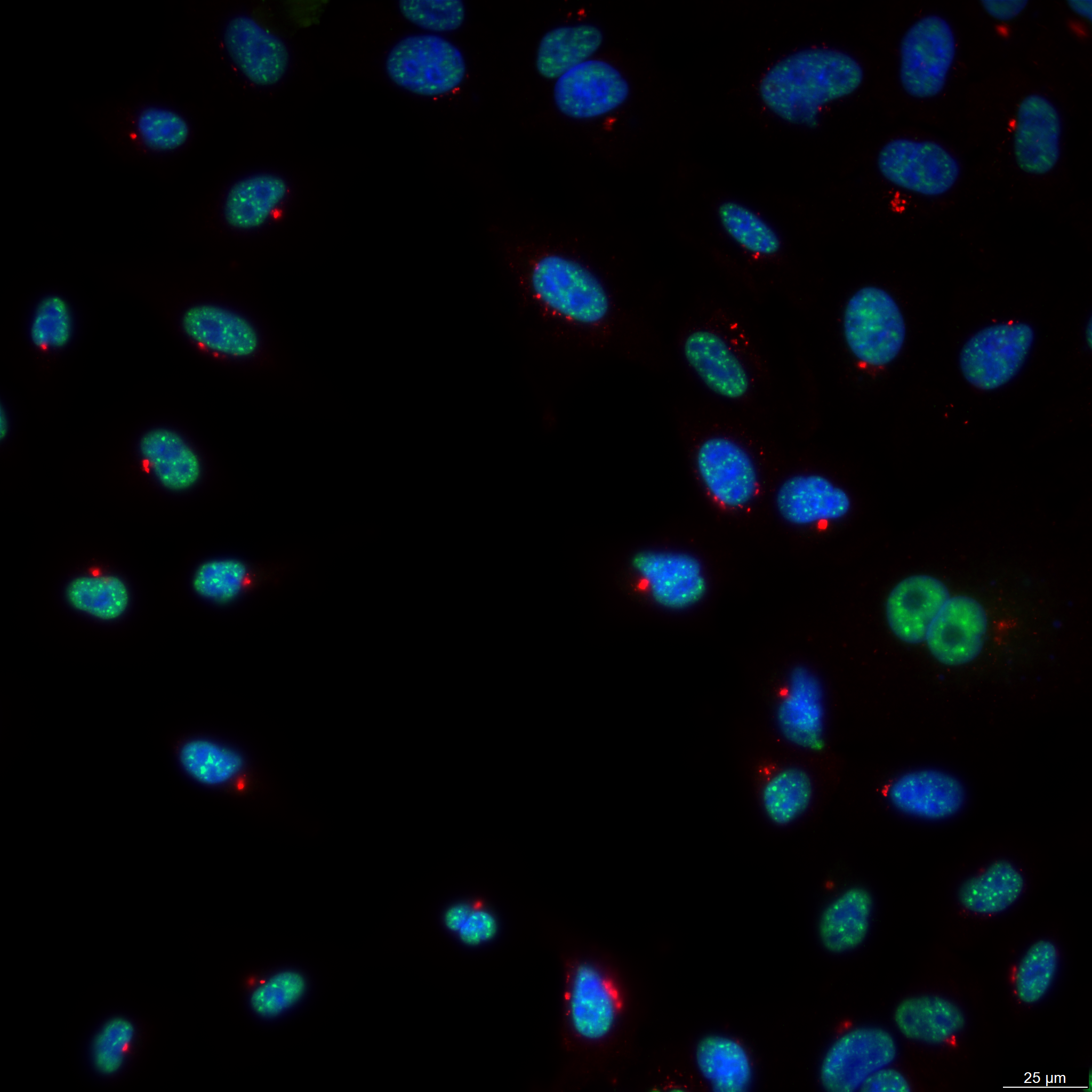

Supplement: Supplementary file 17 — Figure EV6 Source Data [file 44318_2025_421_MOESM17_ESM.zip › EV6/EV6G/lFNγ+MG132 (6 h).tif]

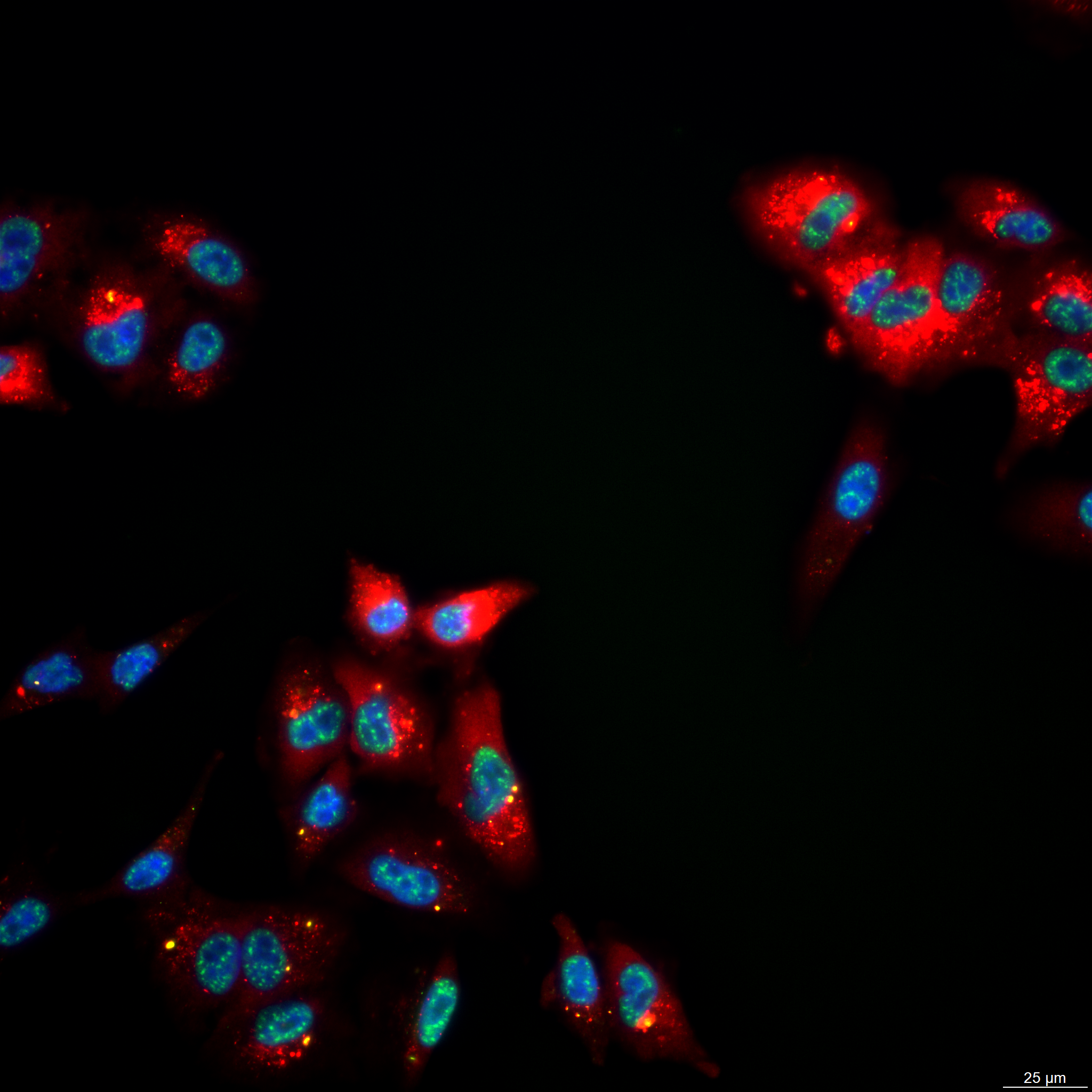

Supplement: Supplementary file 17 — Figure EV6 Source Data [file 44318_2025_421_MOESM17_ESM.zip › EV6/EV6G/lFNγ.tif]

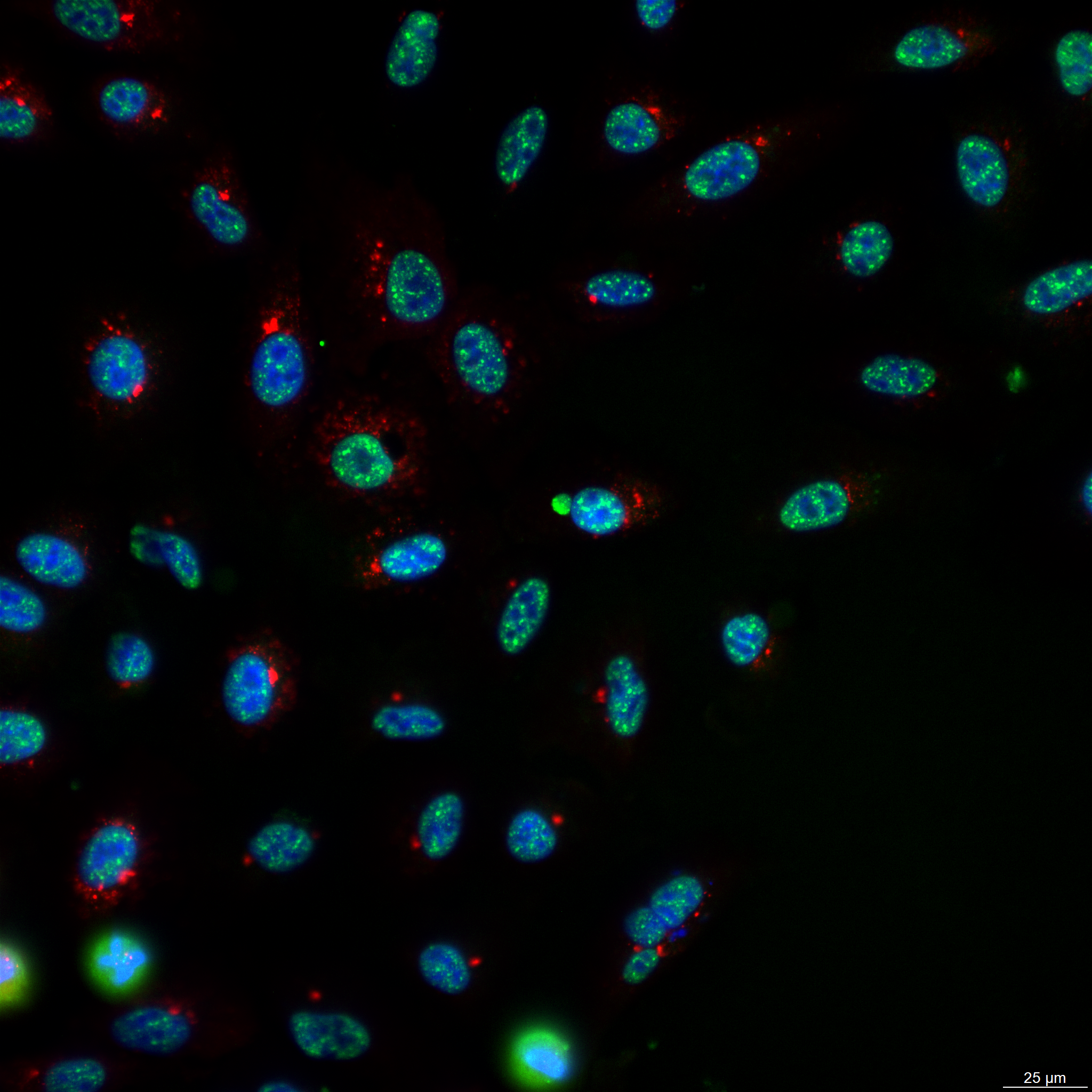

Supplement: Supplementary file 17 — Figure EV6 Source Data [file 44318_2025_421_MOESM17_ESM.zip › EV6/EV6G/MG132.tif]

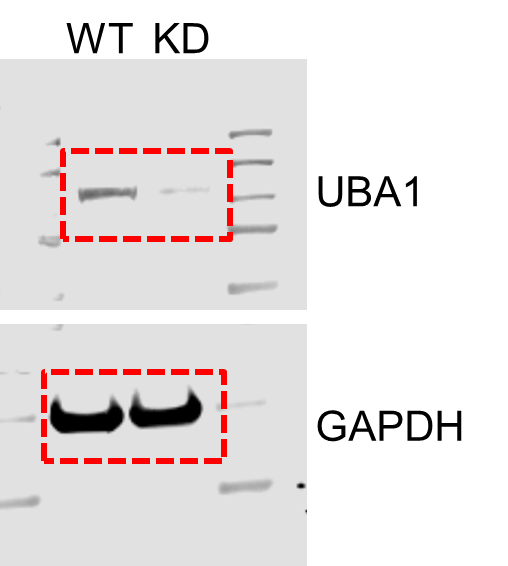

Supplement: Supplementary file 18 — Figure EV7 Source Data [file 44318_2025_421_MOESM18_ESM.zip › EV7/EV7A.tif]

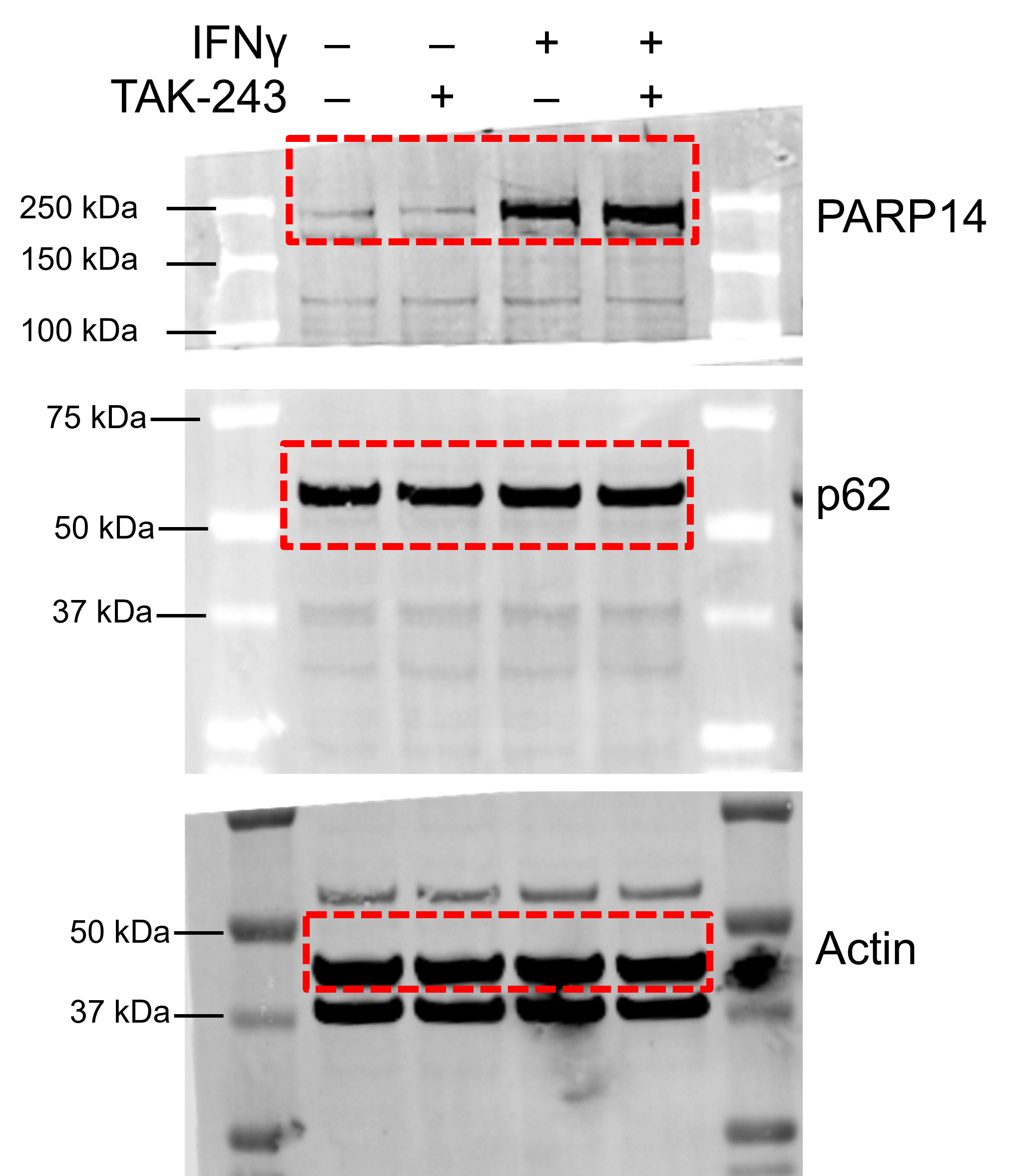

Supplement: Supplementary file 18 — Figure EV7 Source Data [file 44318_2025_421_MOESM18_ESM.zip › EV7/EV7B.tif]

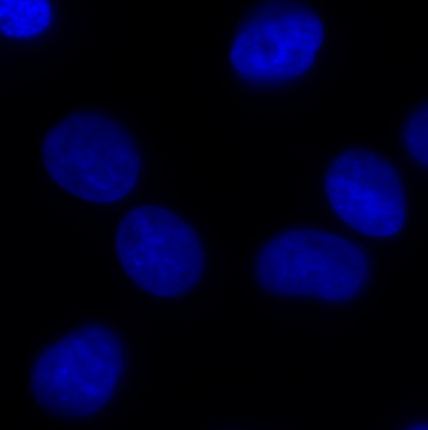

Supplement: Supplementary file 18 — Figure EV7 Source Data [file 44318_2025_421_MOESM18_ESM.zip › EV7/EV7C/Control 2_Crop001_ch00_SV.tif]

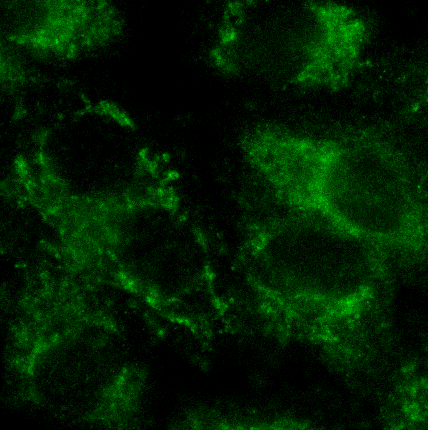

Supplement: Supplementary file 18 — Figure EV7 Source Data [file 44318_2025_421_MOESM18_ESM.zip › EV7/EV7C/Control 2_Crop001_ch01_SV.tif]

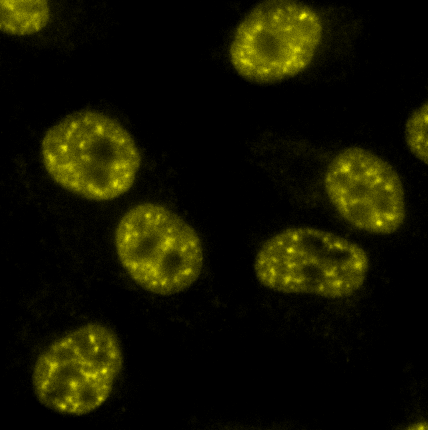

Supplement: Supplementary file 18 — Figure EV7 Source Data [file 44318_2025_421_MOESM18_ESM.zip › EV7/EV7C/Control 2_Crop001_ch02_SV.tif]

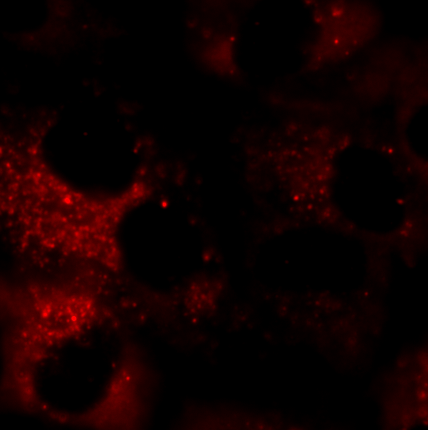

Supplement: Supplementary file 18 — Figure EV7 Source Data [file 44318_2025_421_MOESM18_ESM.zip › EV7/EV7C/Control 2_Crop001_ch03_SV.tif]

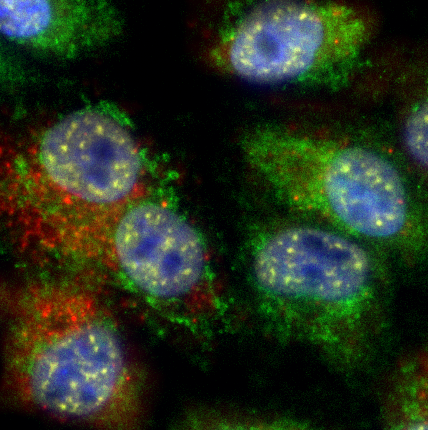

Supplement: Supplementary file 18 — Figure EV7 Source Data [file 44318_2025_421_MOESM18_ESM.zip › EV7/EV7C/Control 2_Crop001_overlay.tif]

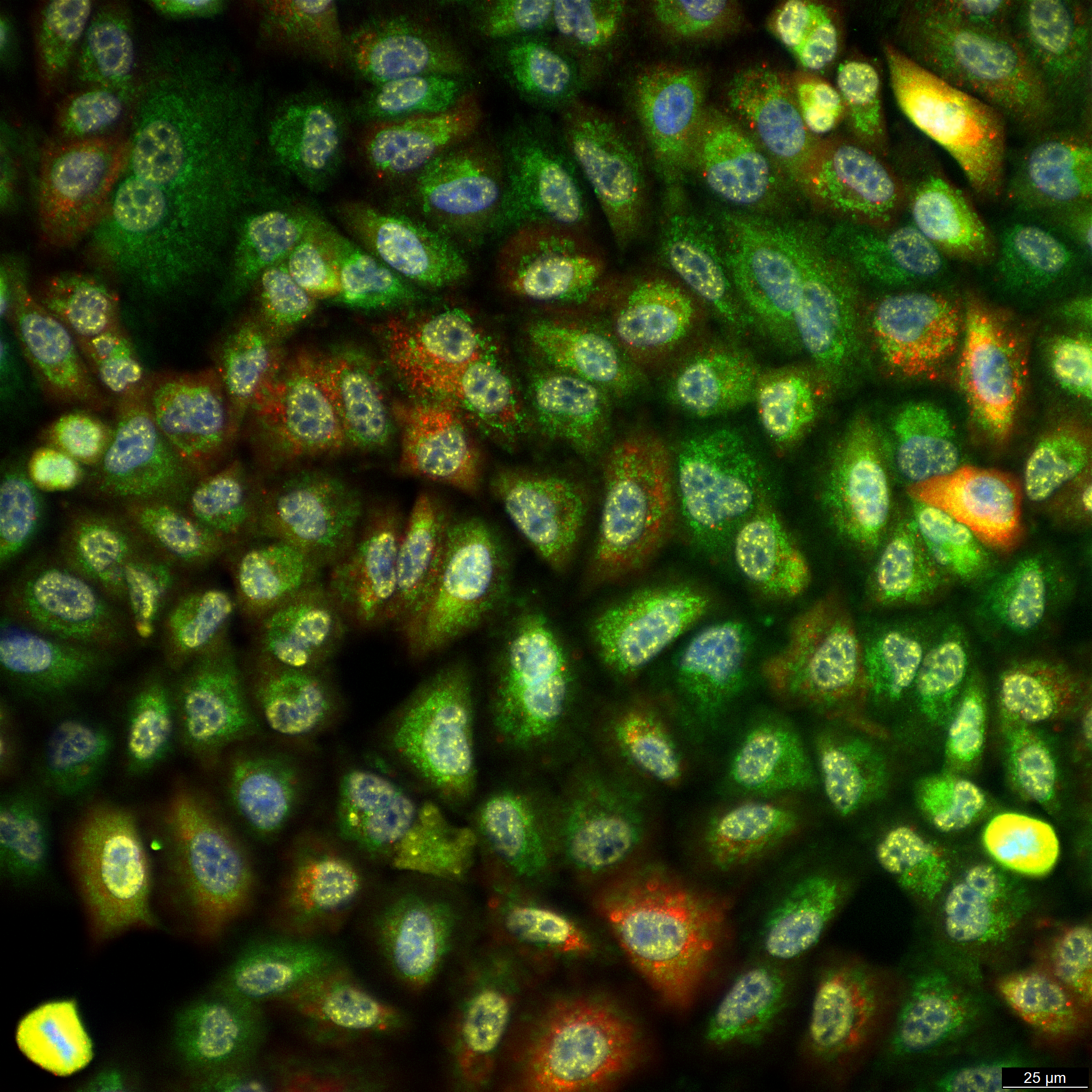

Supplement: Supplementary file 18 — Figure EV7 Source Data [file 44318_2025_421_MOESM18_ESM.zip › EV7/EV7C/Control Merge.tif]

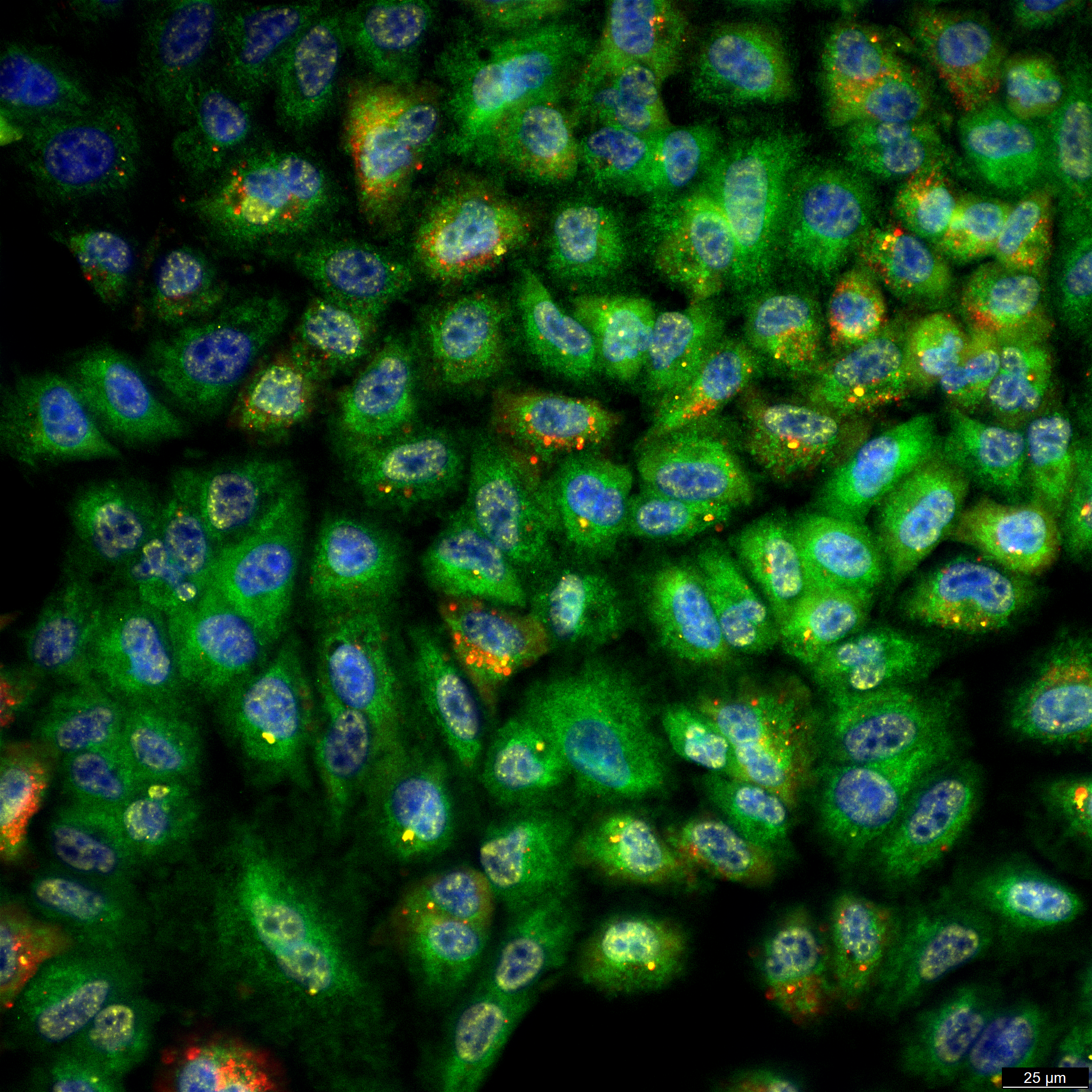

Supplement: Supplementary file 18 — Figure EV7 Source Data [file 44318_2025_421_MOESM18_ESM.zip › EV7/EV7C/IFN Merge.tif]

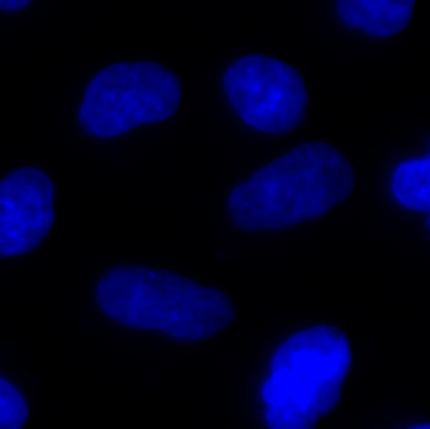

Supplement: Supplementary file 18 — Figure EV7 Source Data [file 44318_2025_421_MOESM18_ESM.zip › EV7/EV7C/IFN TRIM25 1_Crop004_ch00_SV.tif]

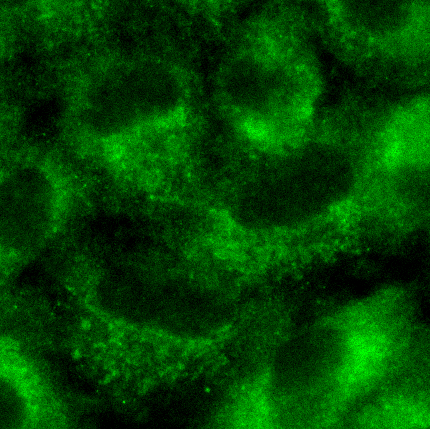

Supplement: Supplementary file 18 — Figure EV7 Source Data [file 44318_2025_421_MOESM18_ESM.zip › EV7/EV7C/IFN TRIM25 1_Crop004_ch01_SV.tif]

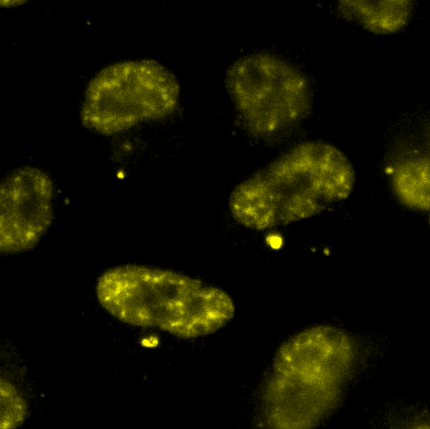

Supplement: Supplementary file 18 — Figure EV7 Source Data [file 44318_2025_421_MOESM18_ESM.zip › EV7/EV7C/IFN TRIM25 1_Crop004_ch02_SV.tif]

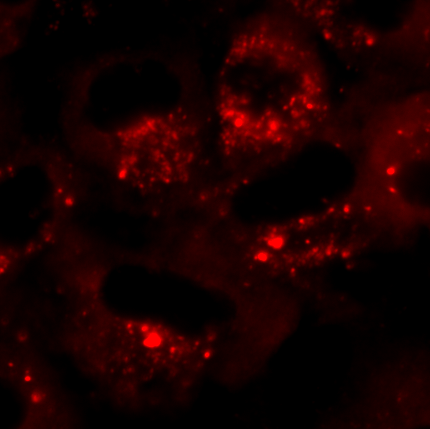

Supplement: Supplementary file 18 — Figure EV7 Source Data [file 44318_2025_421_MOESM18_ESM.zip › EV7/EV7C/IFN TRIM25 1_Crop004_ch03_SV.tif]

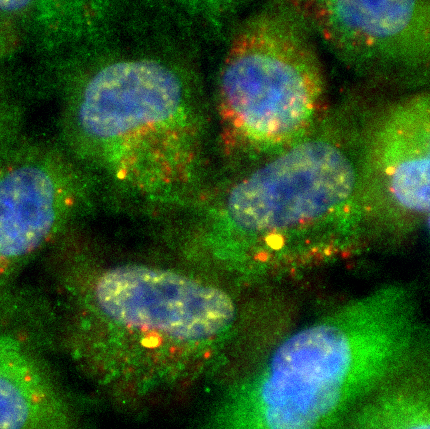

Supplement: Supplementary file 18 — Figure EV7 Source Data [file 44318_2025_421_MOESM18_ESM.zip › EV7/EV7C/IFN TRIM25 1_Crop004_overlay.tif]

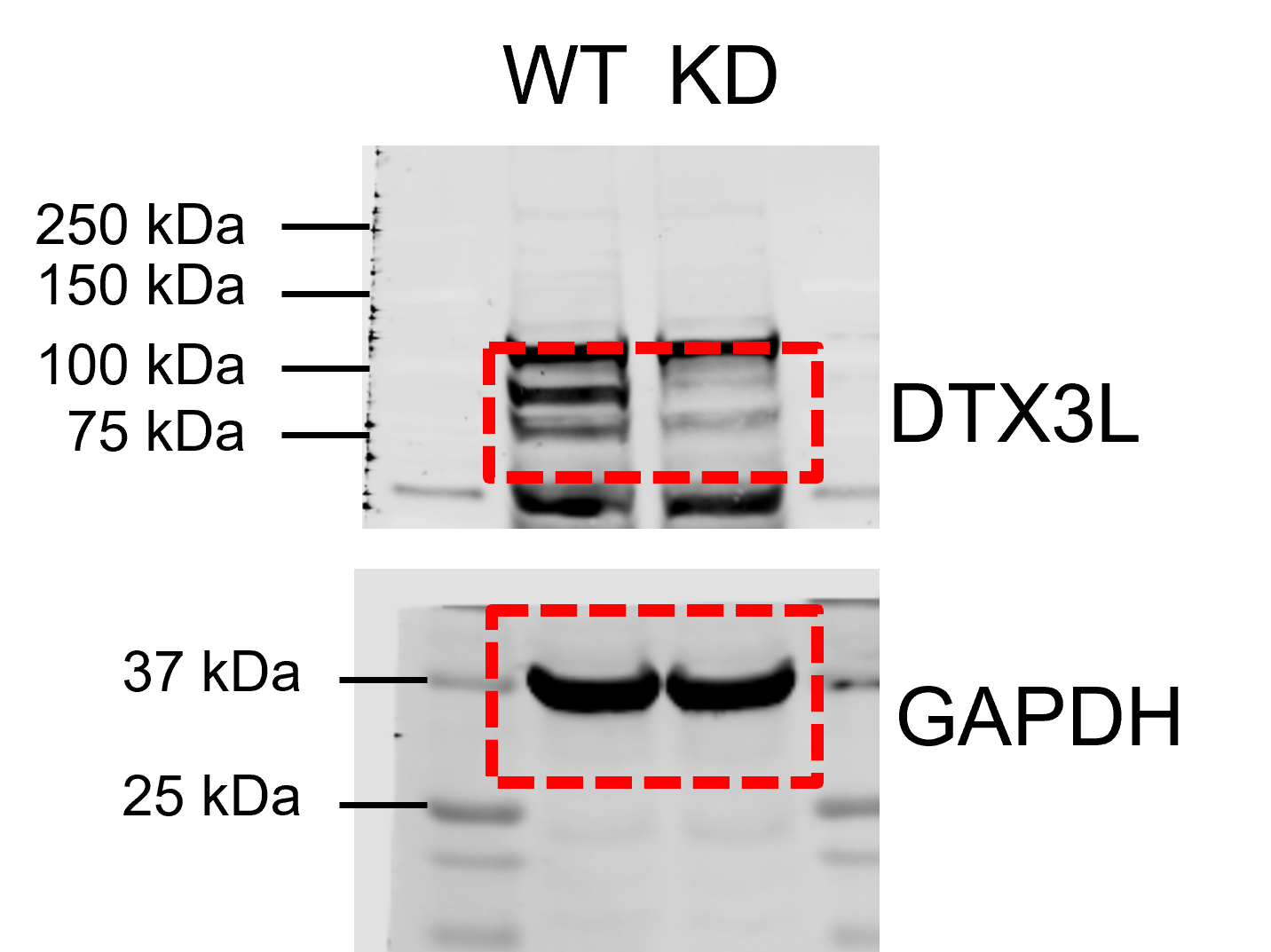

Supplement: Supplementary file 18 — Figure EV7 Source Data [file 44318_2025_421_MOESM18_ESM.zip › EV7/EV7D.tif]

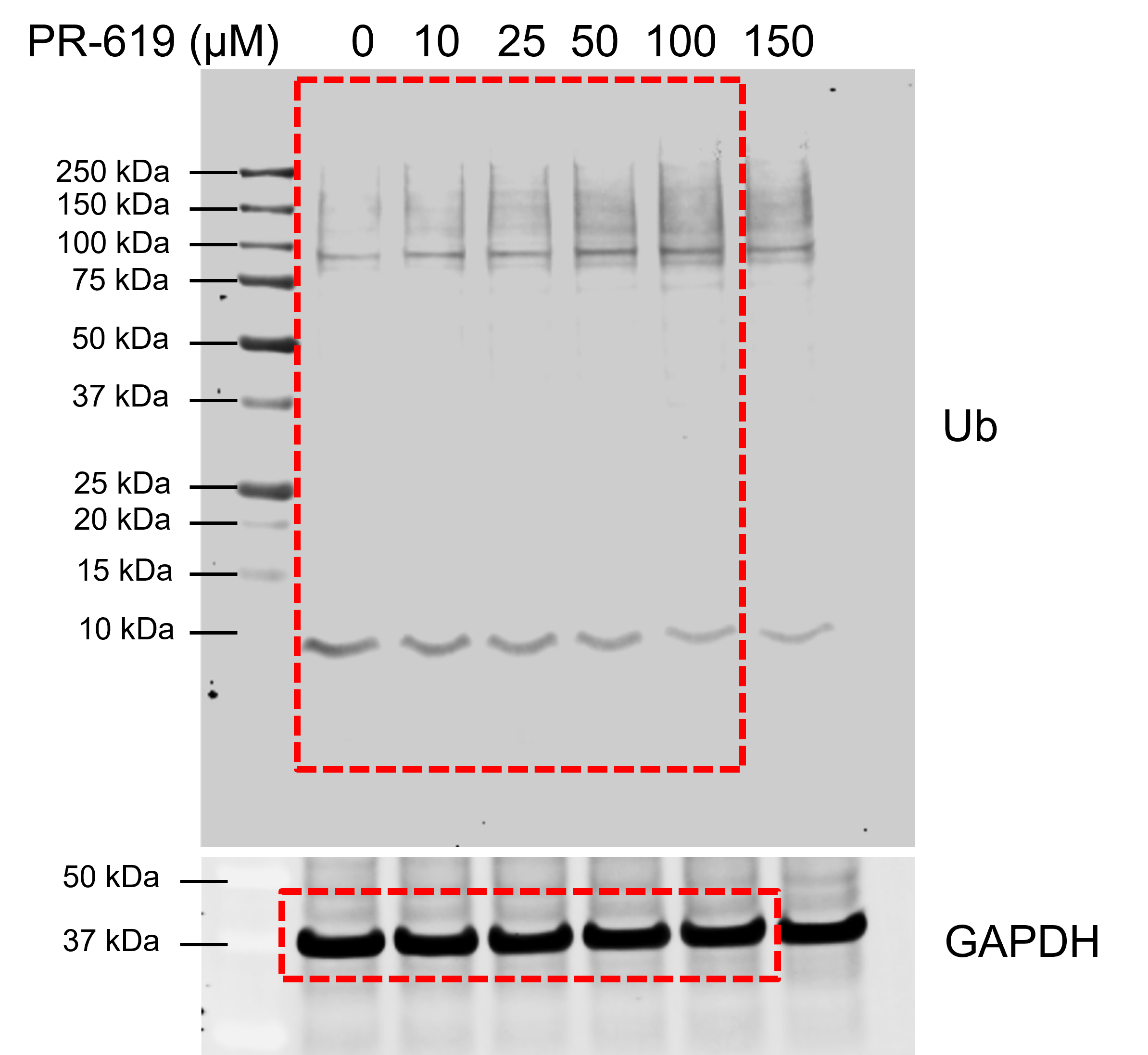

Supplement: Supplementary file 18 — Figure EV7 Source Data [file 44318_2025_421_MOESM18_ESM.zip › EV7/EV7E.tif]
