# Supplementary material for: Interferon-induced PARP14-mediated ADP-ribosylation in p62 bodies requires the ubiquitin-proteasome system
Source: EMBO J. 2025 Apr 7;44(10):2741–73. doi: 10.1038/s44318-025-00421-4 (PMC12084362; doi:10.1038/s44318-025-00421-4)
Supplement: Supplementary file 19 — Expanded View Figures [file 44318_2025_421_MOESM19_ESM.pdf]

Expanded View Figures

Figure EV1. Interferon-induced upregulation of PARP14 is critical for cytoplasmic ADPr condensate formation.

(**A**) ADPr condensates were monitored after cells treated with or without IFNα, β, γ (500 IU/ml or 1000 IU/ml) for 24 h, $n = 52$–112 cells. (**B**) ADPr condensate formation was monitored after cells were treated with IFNγ for the indicated time points and then replaced with fresh medium without IFNγ for a total duration of 24 h, $n = 339$–578 cells. (**C**) RT-qPCR analyses of all human PARP expression in cells upon treatment with or without IFNγ for 6 and 24 h. (**D**) PARP14 mRNA was measured by RT-qPCR in A549 cells pretreated with Actinomycin D (ActD 0.5 μg/ml) for 1 h, followed by 6-h treatment with or without IFNγ. (**E**) PARP9, PARP12, and PARP14 mRNA levels were measured by RT-qPCR in A549 cells treated with or without IFNγ for different time periods, followed by growing cells in media without IFNγ for a total of 24 h (same as panel S1B). Lower panel showing PARP14 protein levels measured by western blot. (**F**) PARP14 mRNA was measured by RT-qPCR after 48 h of siRNA transfection. (**G**) PARP14 protein levels were measured in A549 cells after 48 h of siPARP14 transfection. (**H**) PARP14 protein levels were assessed in cells transfected with si-PARP14 or control siRNA, with or without IFNγ for 24 h. (**I**) ADPr condensates were analyzed in either wild-type or si-PARP14 transfected cells with or without IFNγ treatment for 24 h, $n = 434$-547 cells. (**J**) PARP14 protein levels were measured in A549 stably transduced with shRNA against PARP14 using lentivirus. (**K**) ADPr condensates were analyzed in either wild-type or shPARP14 knockdown cells, after 24-h treatment with or without IFNγ, $n = 375$–449 cells. (**L**) PARP9 protein levels were measured in A549 wild-type (WT) or knockdown cells (KD) stably transduced with shRNA against PARP9 via lentivirus. (**M**) ADPr condensates were analyzed in either WT or shPARP9 KD cells, after 24-h treatment with or without IFNγ, $n = 131$–467 cells. (**N**) PARP12 protein levels were measured in A549 WT or KD cells stably transduced with shRNA against PARP12. (**O**) ADPr condensates were analyzed in either wild-type or shPARP12 knockdown cells after 24-h treatment with or without IFNγ, $n = 225$–267 cells. White arrows indicate the position of condensates, and white boxes represent the zoomed-in regions. The total area of condensates and Pearson correlation coefficient were analyzed by CellProfiler. Mean ± SEM, ns not significant, *$p < 0.05$, **$p < 0.01$, ***$p < 0.001$, ****$P < 0.0001$, unless otherwise stated. (**A**, **B**, **F**) One-way ANOVA, (**C**–**E**, **I**, **K**, **M**, **O**) Two-way ANOVA. All p values are provided in Dataset EV2. Data were representative of three biological replicates. Scale bar, 10 μm. Source data are available online for this figure.

© The Author(s)

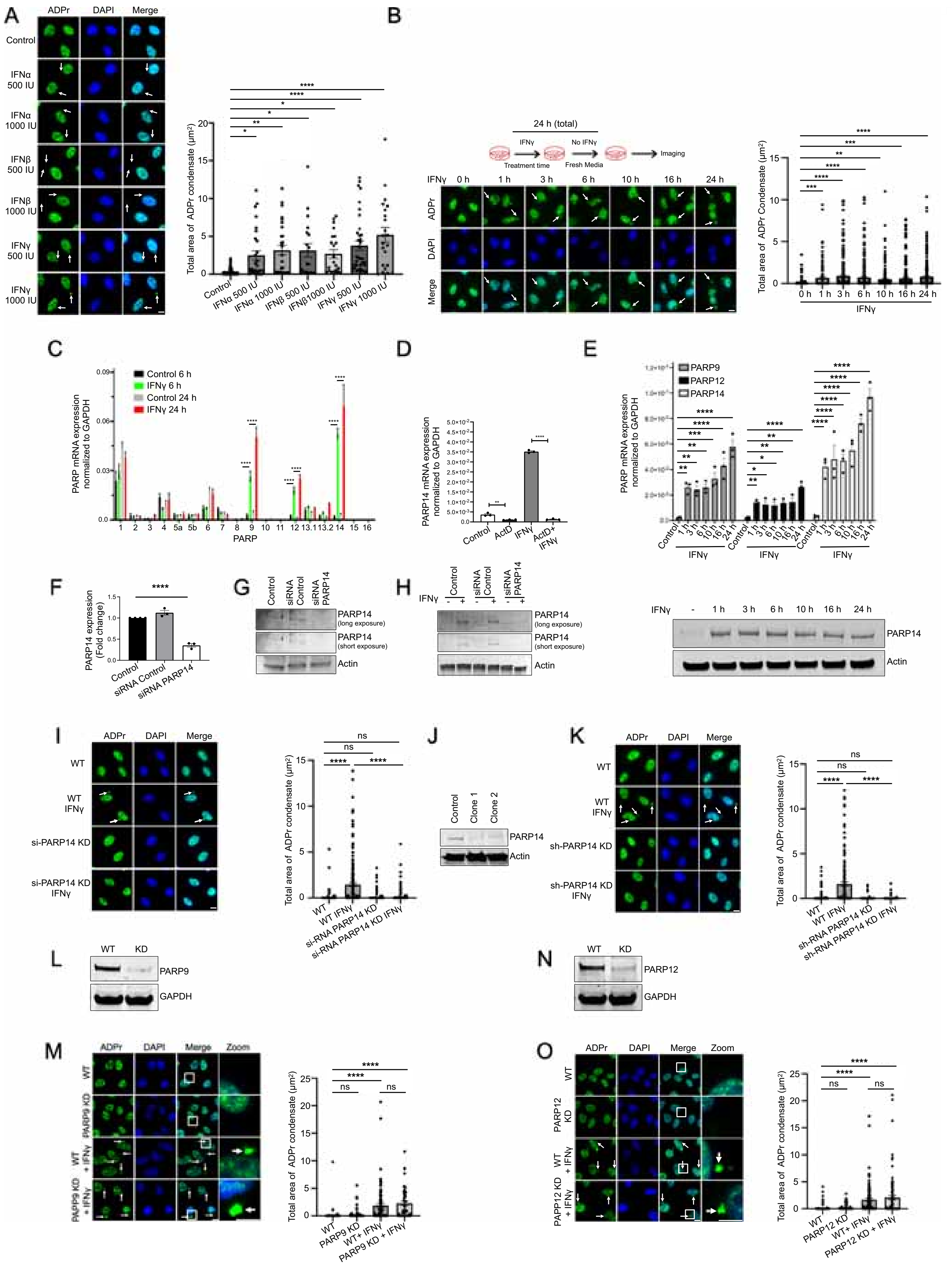

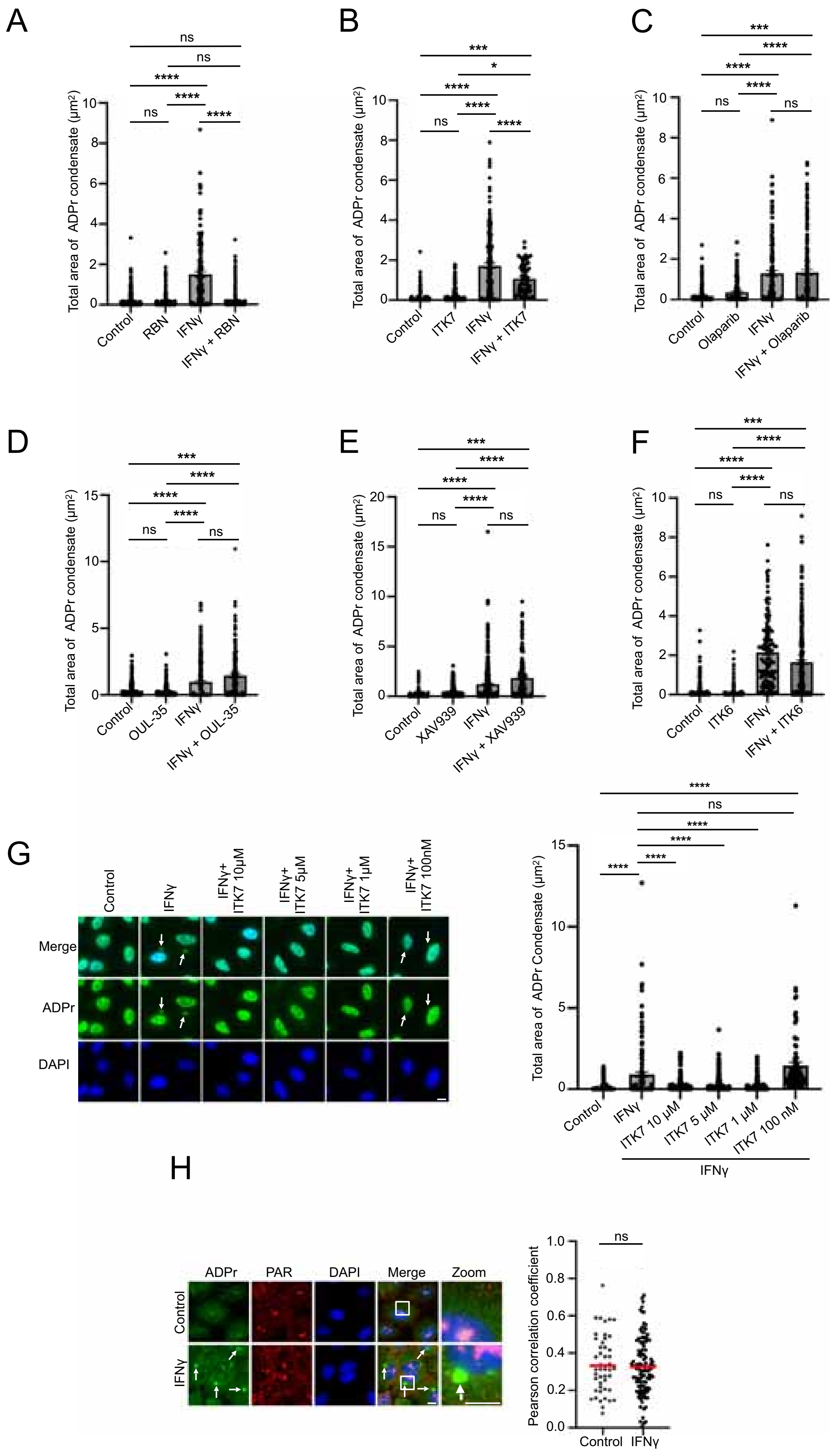

A
B
C
D
E
F
G
H
Total area of ADPr condensate (μm²)
Control
RBN
IFNγ
IFNγ + RBN
ITK7
IFNγ + ITK7
Olaparib
IFNγ + Olaparib
OUL-35
IFNγ + OUL-35
XAV939
IFNγ + XAV939
ITK6
IFNγ + ITK6
Total area of ADPr condensate
IFNγ+ ITK7 10μM
IFNγ+ ITK7 5μM
IFNγ+ ITK7 1μM
IFNγ+ ITK7 100nM
Merge
ADPr
DAPI
Total area of ADPr Condensate (μm²)
ITK7 10 μM
ITK7 5 μM
ITK7 1 μM
ITK7 100 nM
PAR
Zoom
Pearson correlation coefficient
ns

◀ **Figure EV2. Cytoplasmic ADPr condensate formation depends on PARP14 catalytic activity.**

Quantification of ADPr condensates in A549 cells pretreated with different PARP inhibitors for 1 h prior to 24-h treatment with or without IFNγ: (**A**) 10 μM RBN, $n = 236$–607 cells. (**B**) 10 μM ITK7, $n = 166$–506 cells. (**C**) 10 μM Olaparib, $n = 305$–485 cells. (**D**) 3 μM OUL-35, $n = 450$–596 cells. (**E**) 10 μM XAV939, $n = 460$–676 cells, and (**F**) 10 μM ITK6, $n = 301$–593 cells. (**G**) ADPr condensate formation was analyzed in cells pretreated with different doses of ITK7 for 1 h prior to 24-h IFNγ treatment, and compared to untreated control, $n = 163$–1049 cells. (**H**) PAR (Enzo life sciences, BML-SA216) and ADPr (Millipore, MABE1016) colocalization was assessed after 24-h treatment with or without IFNγ, $n = 48$–112 condensates. White arrows indicate the position of condensates, and white boxes represent the zoomed-in regions. The total area of condensates and Pearson correlation coefficient were analyzed by CellProfiler. Mean ± SEM, ns not significant, *$p < 0.05$, **$p < 0.01$, ***$p < 0.001$, ****$p < 0.0001$, unless otherwise stated. (**A**–**G**) Two-way ANOVA; (**H**) Unpaired *t*-test. All *p* values are provided in Dataset EV2. Data were representative of three biological replicates. Scale bar, 10 μm. Source data are available online for this figure.

 © The Author(s)

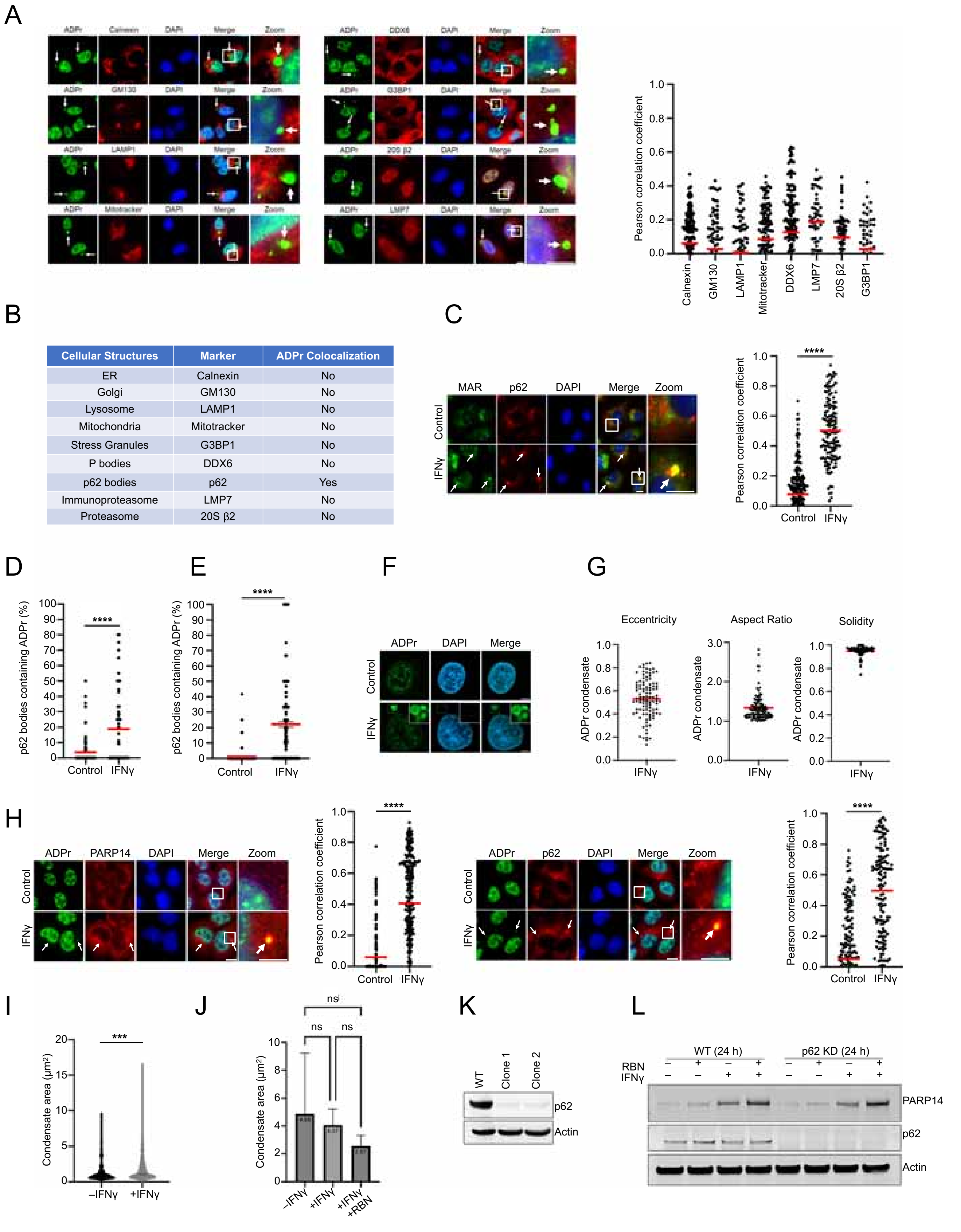

Cellular Structures	Marker	ADPr Colocalization
ER	Calnexin	No
Golgi	GM130	No
Lysosome	LAMP1	No
Mitochondria	Mitotracker	No
Stress Granules	G3BP1	No
P bodies	DDX6	No
p62 bodies	p62	Yes
Immunoproteasome	LMP7	No
Proteasome	20S β2	No

© The Author(s)

◀ **Figure EV3. ADPr condensates colocalize with p62 and exhibit distinct morphometric properties upon IFNγ treatment.**

(**A**) A549 cells were treated with IFNγ for 24 h and stained for colocalization of different markers of cellular structures (red) and ADPr (green), $n = 52–198$ condensates. The colocalization of ADPr condensates with the cellular structures was calculated by CellProfiler software using the Pearson correlation coefficient. (**B**) Table summarizing the colocalization of different cellular structure markers with ADPr condensates in A549 cells. (**C**) Colocalization of p62 with MAR was analyzed in cells with or without 24-h IFNγ treatment, $n = 153–198$ condensates. (**D**) Quantification showing the percentage of p62 bodies positive for ADPr for Fig. 3A, obtained by using a Leica microscope, $n = 70–112$ cells. (**E**) Quantification showing the percentage of p62 bodies positive for ADPr for Fig. 3D, obtained by using Zeiss Airyscan detector, $n = 250$ cells. (**F**, **G**) Representative immunofluorescence images using an Airyscan detector (Zeiss). Cells were imaged after 24-h IFNγ treatment: Morphometry analysis of ADPr condensates was performed by calculating eccentricity, aspect ratio, and solidity measured using the CellProfiler size/shape measurement module. Aspect ratio calculated by the ratio between major axis length and minor axis length of each region of interest, $n = 94–107$ condensates. Scale bars, 5 μm. (**H**) A375 cells were treated with or without IFNγ for 24 h and analyzed for colocalization of ADPr with PARP14 (left panel, $n = 179–188$ condensates) or p62 (right panel, $n = 148–182$ condensates). (**I**) Violin plot showing size quantification of p62 bodies in A549 cells treated with or without IFNγ for 24 h by using imageJ "Max Entropy" for setting threshold and identifying condensates with size greater than 1 pixel using the "Analyze Particle, $n = 182$ condensates (–IFNγ) versus 467 condensates (+IFNγ) from three different fields in each group. (**J**) Size comparison of p62 bodies in FRAP analyses under different conditions for Fig. 3H,I. (**K**) p62 protein levels were measured in shp62 knockdown (KD) A549 cell clones generated by lentiviral transduction. (**L**) PARP14 and p62 protein levels in wild-type (WT) and p62 KD cells pretreated with RBN (10 μM) for 1 h followed by 24-h treatment with or without IFNγ. White arrows indicate the position of condensates, and white boxes represent the zoomed-in regions. The total area of condensates and Pearson correlation coefficient were analyzed by CellProfiler. Mean ± SEM, ns not significant, $*p < 0.05$, $**p < 0.01$, $***p < 0.001$, $****p < 0.0001$, unless otherwise stated. (**C–E**, **H–I**) Unpaired *t*-test; (**J**) One-way ANOVA. All *p* values are provided in Dataset EV2. Data were representative of three biological replicates. Scale bar, 10 μm, unless otherwise indicated. Source data are available online for this figure.

 © The Author(s)

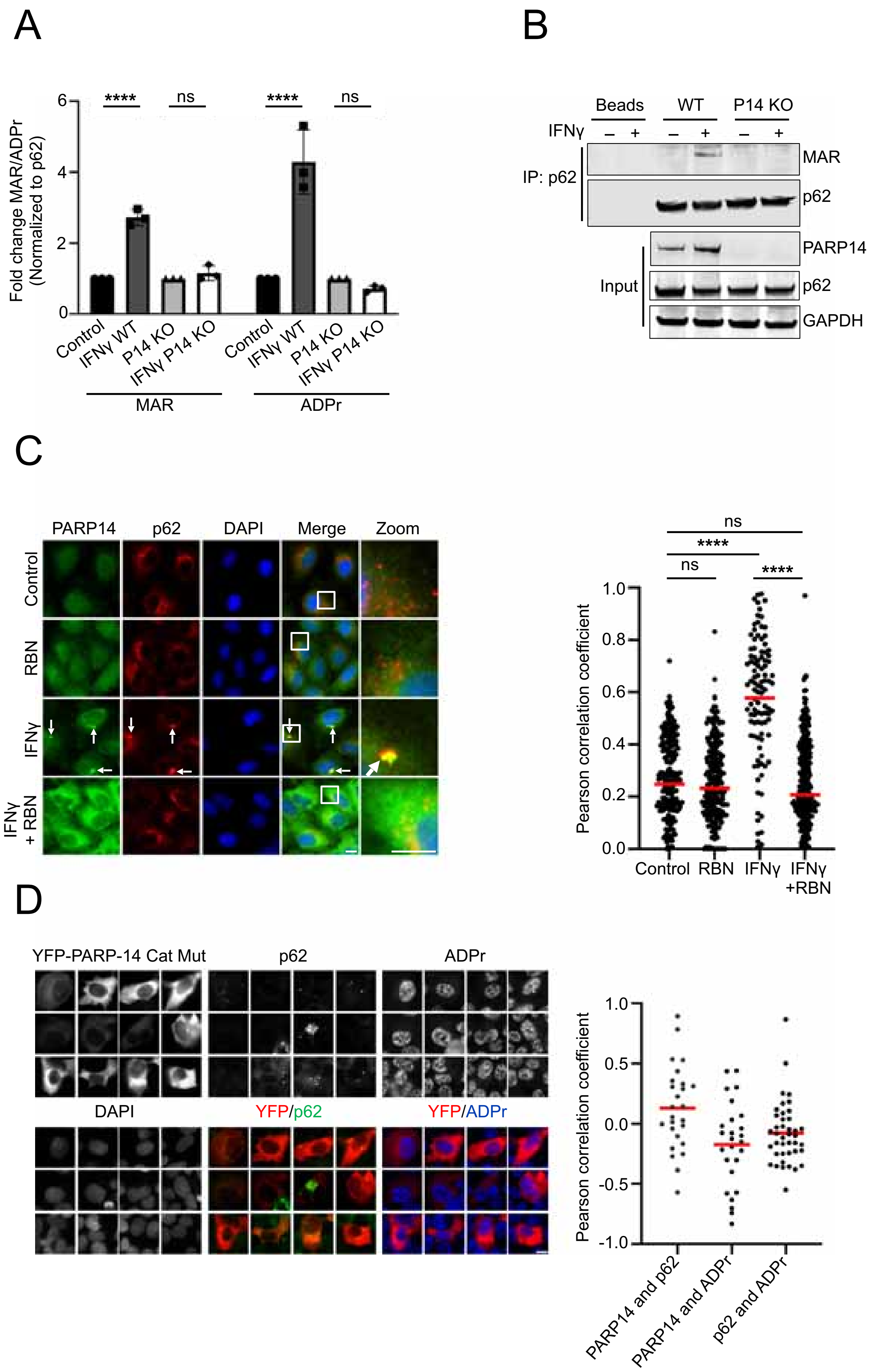
A
Fold change MAR/ADPr (Normalized to p62)
****
ns
Control
IFNγ WT
P14 KO
IFNγ P14 KO
MAR
ADPr
B
Beads
WT
P14 KO
IFNγ
IP: p62
MAR
p62
PARP14
Input
GAPDH
C
PARP14
p62
DAPI
Merge
Zoom
Control
RBN
IFNγ
IFNγ + RBN
Pearson correlation coefficient
IFNγ +RBN
D
YFP-PARP-14 Cat Mut
ADPr
YFP/p62
YFP/ADPr
PARP14 and p62
PARP14 and ADPr
p62 and ADPr

Figure EV4. PARP14-mediated p62 co-condensation depends on PARP14 catalytic activity.

(**A**) Quantification of MAR and ADPr signal on p62 immunoprecipitates, as shown in Fig. 4D, from wild-type (WT) and PARP14 knockout (KO) A549 cells after 24-h treatment with or without IFNγ. (**B**) Immunoprecipitates of p62 performed under denaturing conditions were probed for MAR (HCA-355) and p62 from WT and PARP14 KO A549 cells after 24-h treatment with or without IFNγ. (**C**) PARP14 (Abcam) and p62 colocalization was assessed in cells pretreated with or without RBN for 1 h, followed by 24-h treatment with or without IFNγ, $n = 106–321$ condensates. (**D**) U2OS cells were transiently transfected with YFP-PARP14 catalytic mutant and analyzed for ADPr condensate formation and PARP14 and p62 colocalization, $n = 27–44$ condensates. White arrows indicate the position of condensates, and white boxes represent the zoomed-in regions. The total area of condensates and Pearson correlation coefficient were analyzed by CellProfiler. Mean ± SEM, ns not significant, $*p < 0.05$, $**p < 0.01$, $***p < 0.001$, $****p < 0.0001$, unless otherwise stated. (**A**, **C**) Two-way ANOVA. All p values are provided in Dataset EV2. Data were representative of three biological replicates. Scale bar, 10 µm. Source data are available online for this figure.

 © The Author(s)

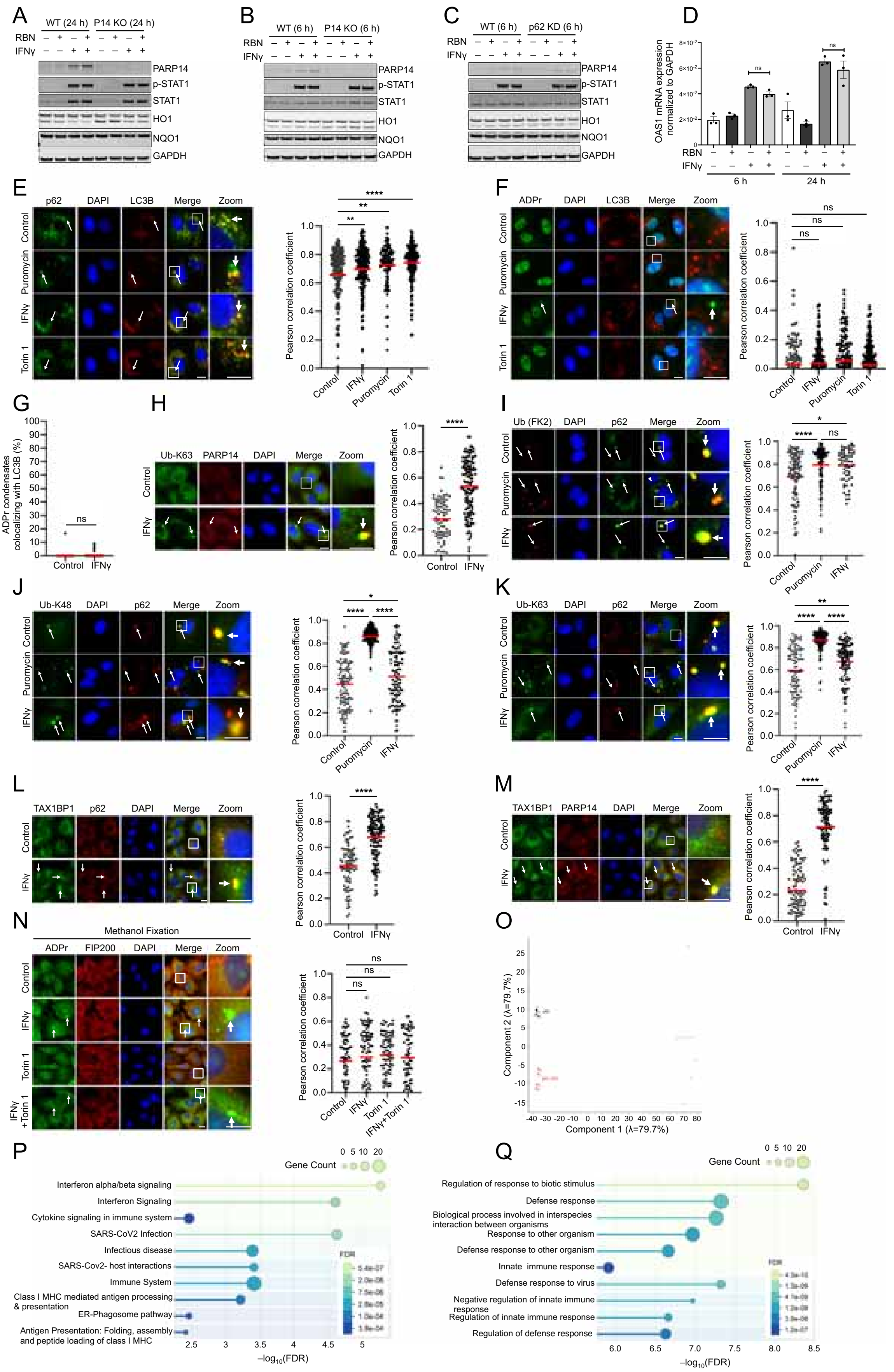

A
WT (24 h)
P14 KO (24 h)
RBN
IFNγ
PARP14
p-STAT1
STAT1
HO1
NQO1
GAPDH
B
WT (6 h)
P14 KO (6 h)
C
WT (6 h)
p62 KD (6 h)
D
OAS1 mRNA expression normalized to GAPDH
ns
6 h
24 h
E
p62
DAPI
LC3B
Merge
Zoom
Control
Puromycin
IFNγ
Torin 1
Pearson correlation coefficient
F
ADPr
G
ADPr condensates colocalizing with LC3B (%)
H
Ub-K63
PARP14
I
Ub (FK2)
J
Ub-K48
K
L
TAX1BP1
M
N
Methanol Fixation
FIP200
IFNγ+Torin 1
O
Component 2 (λ=79.7%)
Component 1 (λ=79.7%)
P
Gene Count
Interferon alpha/beta signaling
Interferon Signaling
Cytokine signaling in immune system
SARS-CoV2 Infection
Infectious disease
SARS-Cov2- host interactions
Immune System
Class I MHC mediated antigen processing & presentation
ER-Phagosome pathway
Antigen Presentation: Folding, assembly and peptide loading of class I MHC
FDR
−log10(FDR)
Q
Regulation of response to biotic stimulus
Defense response
Biological process involved in interspecies interaction between organisms
Response to other organism
Defense response to other organism
Innate immune response
Defense response to virus
Negative regulation of innate immune response
Regulation of innate immune response
Regulation of defense response

Figure EV5. ADPr-enriched p62 bodies selectively contain components of canonical p62 bodies.

(**A**, **B**) Western blot analyses of p-STAT1, STAT1, HO1, and NQO1 in A549 wild-type (WT) and PARP14 knockout (KO) cells treated with different combinations of 1-h RBN (10 µM) pretreatment and 6-h or 24-h IFNγ treatment. (**C**) Western blot analyses of p-STAT1, STAT1, HO1, and NQO1 in wild-type (WT) and p62 knockdown (KD) cells treated with different combinations of 1-h RBN (10 µM) pretreatment and 6-h IFNγ treatment. (**D**) RT-qPCR analyses of OAS1 mRNA levels in cells treated with different combinations of RBN (10 µM) pretreatment for 1 h, followed by IFNγ treatment for either 6 or 24 h. (**E**, **F**) Colocalization of LC3B with (E) p62, $n = 105–221$ condensates, and (**F**) ADPr, $n = 105–165$ condensates, was assessed in cells either treated with puromycin (5 µg/ml) for 3 h, IFNγ overnight or Torin 1 (1 µM) for 3 h, and compared to untreated control. Puromycin and Torin 1 treatment was given for 3 h before fixing cells. (**G**) Quantification showing the percentage of LC3B positive for ADPr for Fig. 5D, $n = 81–87$ condensates. (**H**) Colocalization of PARP14 and Ub-K63 was assessed in cells treated with or without IFNγ for 24 h, $n = 74–135$ condensates. (**I**–**K**) Colocalization of p62 with (**I**) Ubiquitin, $n = 64–124$ condensates, (**J**) Ub-K48, $n = 113–210$ condensates, and (**K**) Ub-K63, $n = 96–177$ condensates, was assessed in cells treated with or without IFNγ or puromycin. IFNγ was given overnight while puromycin (5 µg/ml) treatment was given for 3 h before fixation. (**L**) Colocalization of TAX1BP1 and p62 was assessed after 24-h treatment with or without IFNγ, same as Fig. 5H, 86–131 condensates. (**M**) Colocalization of TAX1BP1 and PARP14 was assessed after 24-h treatment with or without IFNγ, $n = 98–101$ condensates. (**N**) Colocalization of FIP200 and ADPr was assessed after treating A549 cells with IFNγ overnight, followed by either DMSO or Torin 1 (1 µM) treatment for 6-h using ice-cold methanol for fixation, $n = 55–108$ condensates. (**O**) Principal component analysis (PCA) plot of immunoprecipitated samples across four biological replicates: IgG Control (gray), p62 alone (black), and p62 in the presence of IFNγ (red). (**P**, **Q**) Term enrichment analysis showing (**P**) Reactome pathway, and (**Q**) gene ontology statistically enriched within the p62-specific network after IFNγ treatment. Statistical testing was conducted using the STRING database with default settings. White arrows indicate the position of condensates, and white boxes represent the zoomed-in regions. The total area of condensates and Pearson correlation coefficient were analyzed by CellProfiler. Mean ± SEM, ns not significant, *$p < 0.05$, **$p < 0.01$, ***$p < 0.001$, ****$p < 0.0001$, unless otherwise stated. (**D**) One-way ANOVA; (**E**, **F**, **I**–**K**, **N**) Two-way ANOVA; (**G**, **H**, **L**, **M**) Unpaired *t*-test. All *p* values are provided in Dataset EV2. Data were representative of three biological replicates. Scale bar, 10 µm. Source data are available online for this figure.

 © The Author(s)

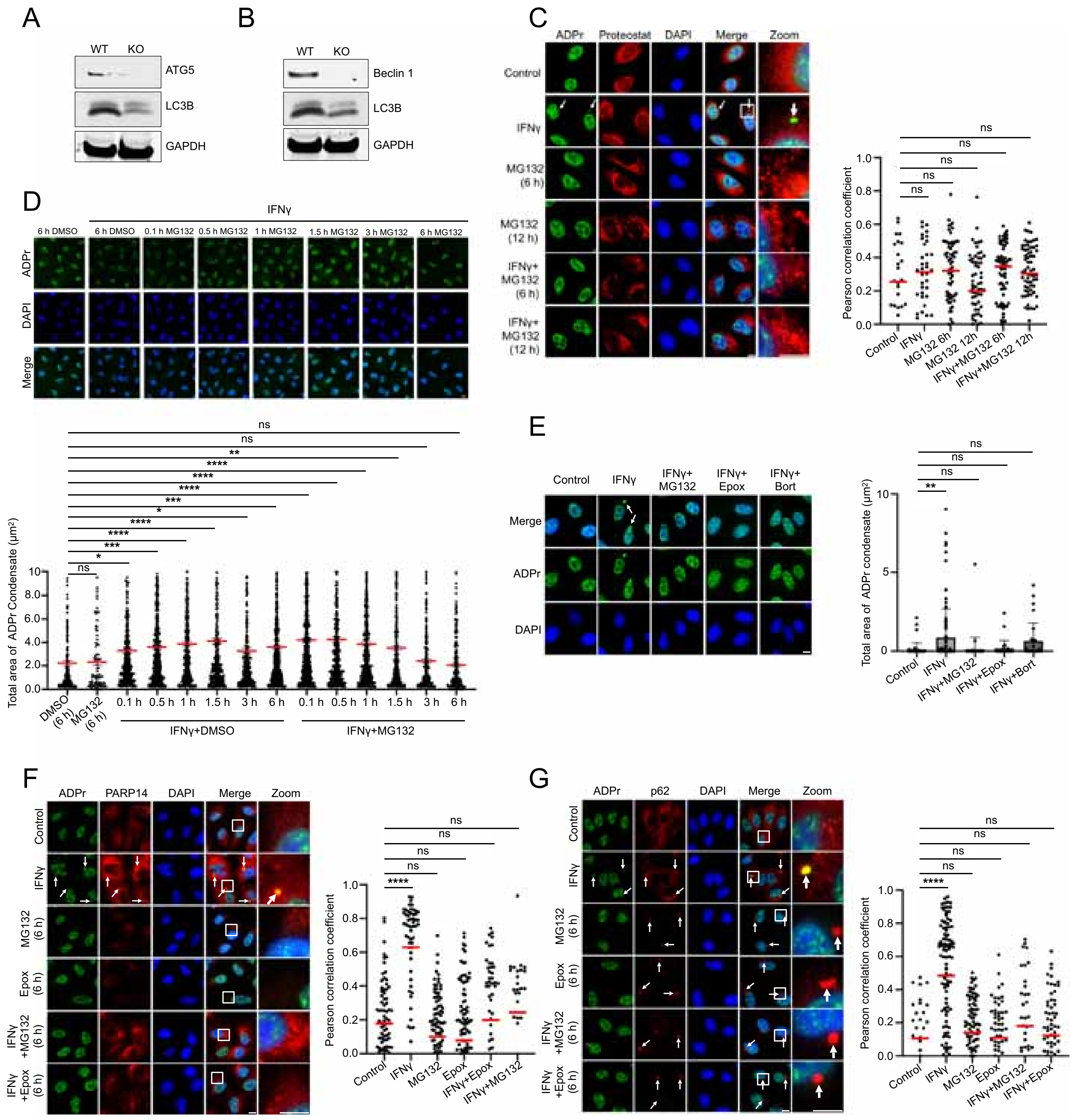
A
WT KO
ATG5
LC3B
GAPDH
B
WT KO
Beclin 1
LC3B
GAPDH
C
ADPr Proteostat DAPI Merge Zoom
Control
IFNγ
MG132 (6 h)
MG132 (12 h)
IFNγ+ MG132 (6 h)
IFNγ+ MG132 (12 h)
Pearson correlation coefficient
Control IFNγ MG132 6h MG132 12h IFNγ+MG132 6h IFNγ+MG132 12h
ns
D
IFNγ
6 h DMSO 6 h DMSO 0.1 h MG132 0.5 h MG132 1 h MG132 1.5 h MG132 3 h MG132 6 h MG132
ADPr
DAPI
Merge
Total area of ADPr Condensate (µm²)
DMSO (6 h) MG132 (6 h) 0.1 h 0.5 h 1 h 1.5 h 3 h 6 h 0.1 h 0.5 h 1 h 1.5 h 3 h 6 h
IFNγ+DMSO
IFNγ+MG132
E
Control IFNγ IFNγ+ MG132 IFNγ+ Epox IFNγ+ Bort
Merge
ADPr
DAPI
Total area of ADPr condensate (µm²)
Control IFNγ IFNγ+MG132 IFNγ+Epox IFNγ+Bort
F
ADPr PARP14 DAPI Merge Zoom
Control
IFNγ
MG132 (6 h)
Epox (6 h)
IFNγ +MG132 (6 h)
IFNγ +Epox (6 h)
Pearson correlation coefficient
Control IFNγ MG132 Epox IFNγ+Epox IFNγ+MG132
G
ADPr p62 DAPI Merge Zoom
Control
IFNγ
MG132 (6 h)
Epox (6 h)
IFNγ +MG132 (6 h)
IFNγ +Epox (6 h)
Pearson correlation coefficient
Control IFNγ MG132 Epox IFNγ+MG132 IFNγ+Epox

Figure EV6. Cytoplasmic ADPr condensates do not colocalize with aggresomes but depend on proteasome activity.

(**A**) Western blot showing ATG5 and LC3B protein levels in ATG5 knockout (KO) cells. (**B**) Western blot showing Beclin 1 and LC3B protein levels in Beclin 1 KO cells. (**C**) Colocalization of ADPr and aggresomes stained with PROTEOSTAT® dye was assessed in A549 cells treated with or without IFNγ overnight, followed by 10 µM MG132 treatment for either 6 or 12 h, $n = 26–71$ condensates. (**D**) ADPr condensates were monitored after cells were treated with IFNγ for 24 h, followed by 10 µM MG132 for different time periods as indicated, and compared to untreated control. The lower panel shows quantification of ADPr condensates in IFNγ-treated cells either in the presence of DMSO or 10 µM MG132, $n = 85–535$ cells. (**E**) ADPr condensate formation was assessed in cells treated with IFNγ overnight, followed by treatment with proteasome inhibitors 10 µM MG132 or 1 µM Epoxomicin (Epox) or Bortezomib (100 nM) for 6 h, $n = 88–227$ cells. (**F**) Colocalization of ADPr and PARP14 was analyzed in cells treated with IFNγ overnight, followed by the treatment with 10 µM MG132 or 1 µM Epoxomicin for 6 h, same as in Fig. 6D, and compared to untreated control, $n = 33–106$ condensates. (**G**) Colocalization of ADPr and p62 was analyzed in cells treated with MG132 or Epoxomicin, as in panel (**F**), $n = 33–109$ condensates. White arrows indicate the position of condensates, and white boxes represent the zoomed-in regions. The total area of condensates and Pearson correlation coefficient were analyzed by CellProfiler. Mean ± SEM, ns not significant, $^*p < 0.05$, $^{**}p < 0.01$, $^{***}p < 0.001$, $^{****}p < 0.0001$, unless otherwise stated. (**C–G**) One-way ANOVA. All p values are provided in Dataset EV2. Data were representative of three biological replicates. Scale bar, 10 µm. Source data are available online for this figure.

 © The Author(s)

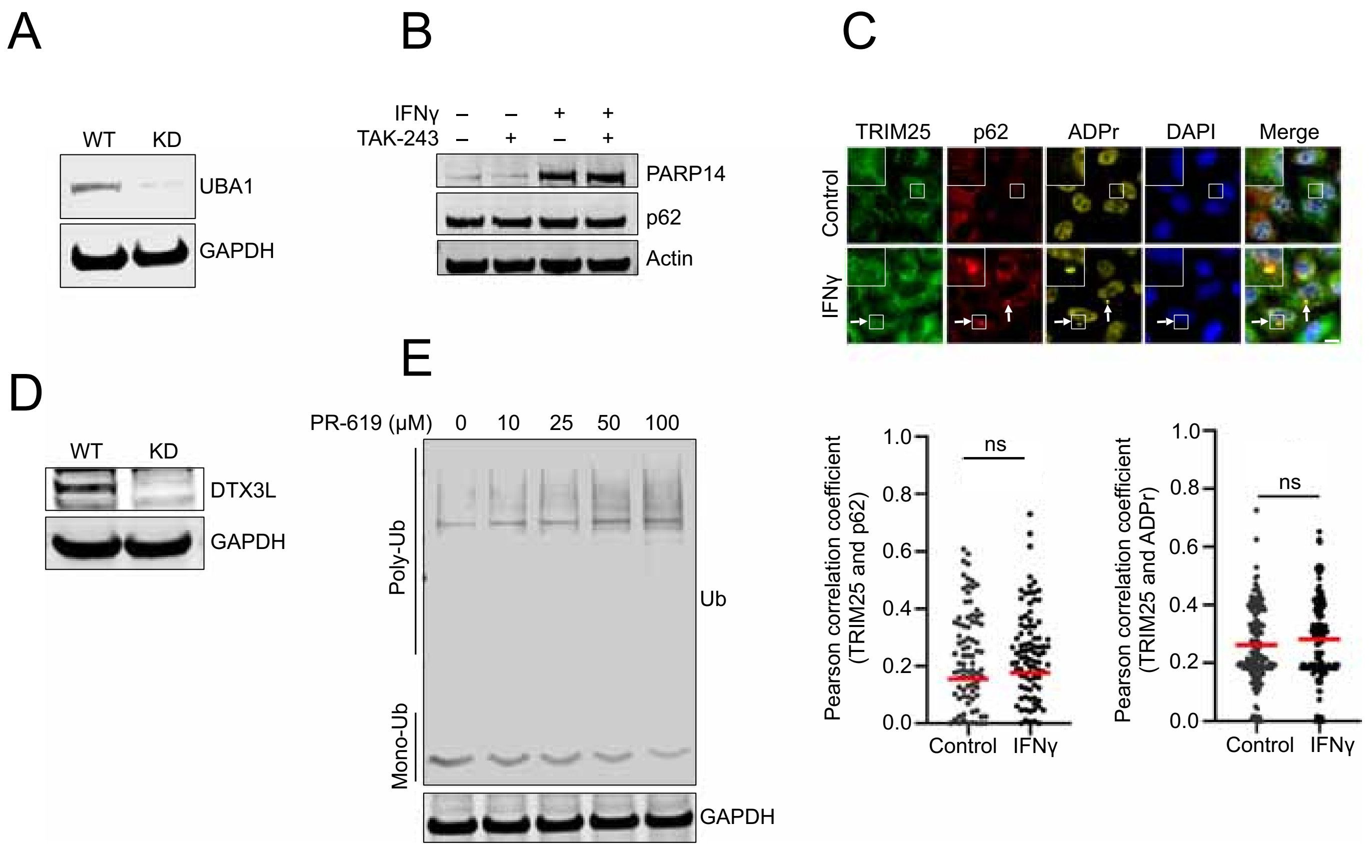

Figure EV7. Cytoplasmic ADPr condensate formation depends on specific components of the ubiquitination pathway.

(**A**) Western blot showing UBA1 protein levels in A549 wild-type (WT) and lentiviral shRNA-UBA1 knockdown (KD) cells. (**B**) PARP14 and p62 protein levels were analyzed from samples in Fig. 7B. (**C**) Colocalization of TRIM25 with p62 and ADPr was analyzed in A549 cells with or without 24-h IFNγ treatment, $n = 106$–108 condensates for the p62 panel and $n = 150$ condensates for the ADPr panel. (**D**) Western blot showing DTX3L protein levels in A549 WT and lentiviral shRNA-DTX3L KD cells. (**E**) The total ubiquitination profile was assessed in A549 cells treated with different doses of PR619 for 8 h. White arrows indicate the position of condensates, and white boxes represent the zoomed-in regions. The total area of condensates and Pearson correlation coefficient were analyzed by CellProfiler. Mean ± SEM, ns not significant. (**C**) Unpaired *t*-test. All *p* values are provided in Dataset EV2. Data were representative of three biological replicates. Scale bar, 10 μm. Source data are available online for this figure.
